# Exploring Barmah Forest virus pathogenesis: molecular tools to investigate non-structural protein 3 nuclear localization and viral genomic determinants of replication

**DOI:** 10.1128/mbio.00993-24

**Published:** 2024-07-02

**Authors:** Ailar Omler, Margit Mutso, Mihkel Vaher, Joseph R. Freitas, Adam Taylor, Cassandra T. David, Gregory W. Moseley, Xiang Liu, Andres Merits, Suresh Mahalingam

**Affiliations:** 1 Emerging Viruses, Inflammation and Therapeutics Group, Menzies Health Institute Queensland, Griffith University, Gold Coast, Queensland, Australia; 2 Institute of Bioengineering, University of Tartu, Tartu, Estonia; 3 Institute of Technology, University of Tartu, Tartu, Estonia; 4 The Institute of Molecular and Cell Biology, University of Tartu, Tartu, Estonia; 5 Global Virus Network (GVN) Centre for Excellence in Arboviruses, Griffith University, Gold Coast, Queensland, Australia; 6 School of Pharmacy and Medical Sciences, Griffith University, Gold Coast, Queensland, Australia; 7 Department of Microbiology, Biomedicine Discovery Institute, Monash University, Clayton, Victoria, Australia; Johns Hopkins Bloomberg School of Public Health, Baltimore, Maryland, USA

**Keywords:** Barmah Forest virus, immune evasion, alphavirus, nuclear transport, reverse genetic analysis, viral replication

## Abstract

Barmah Forest virus (BFV) is a mosquito-borne virus that causes arthralgia with accompanying rash, fever, and myalgia in humans. The virus is mainly found in Australia and has caused outbreaks associated with significant health concerns. As the sole representative of the Barmah Forest complex within the genus *Alphavirus*, BFV is not closely related genetically to other alphaviruses. Notably, basic knowledge of BFV molecular virology has not been well studied due to a lack of critical investigative tools such as an infectious clone. Here we describe the construction of an infectious BFV cDNA clone based on Genbank sequence and demonstrate that the clone-derived virus has *in vitro* and *in vivo* properties similar to naturally occurring virus, BFV field isolate 2193 (BFV2193-FI). A substitution in nsP4, V1911D, which was identified in the Genbank reference sequence, was found to inhibit virus rescue and replication. T1325P substitution in nsP2 selected during virus passaging was shown to be an adaptive mutation, compensating for the inhibitory effect of nsP4-V1911D. The two mutations were associated with changes in viral non-structural polyprotein processing and type I interferon (IFN) induction. Interestingly, a nuclear localization signal, active in mammalian but not mosquito cells, was identified in nsP3. A point mutation abolishing nsP3 nuclear localization attenuated BFV replication. This effect was more prominent in the presence of type I interferon signaling, suggesting nsP3 nuclear localization might be associated with IFN antagonism. Furthermore, abolishing nsP3 nuclear localization reduced virus replication in mice but did not significantly affect disease.

**IMPORTANCE:**

Barmah Forest virus (BFV) is Australia’s second most prevalent arbovirus, with approximately 1,000 cases reported annually. The clinical symptoms of BFV infection include rash, polyarthritis, arthralgia, and myalgia. As BFV is not closely related to other pathogenic alphaviruses or well-studied model viruses, our understanding of its molecular virology and mechanisms of pathogenesis is limited. There is also a lack of molecular tools essential for corresponding studies. Here we describe the construction of an infectious clone of BFV, variants harboring point mutations, and sequences encoding marker protein. In infected mammalian cells, nsP3 of BFV was located in the nuclei. This finding extends our understanding of the diverse mechanisms used by alphavirus replicase proteins to interact with host cells. Our novel observations highlight the complex synergy through which the viral replication machinery evolves to correct mutation errors within the viral genome.

## INTRODUCTION

Barmah Forest virus (BFV) belongs to the genus *Alphavirus* (family *Togaviridae*). The prototype strain of BFV (BFV2193-FI) was first isolated in 1974 from *Culex annulirostris* mosquitoes collected from Barmah Forest in the Murray Valley, Northern Victoria, Australia (1). Since then, multiple additional strains have been isolated from different mosquito species and geographical regions of Australia. In addition to *Culex* species, BFV transmission by *Aedes vigilax*, *Aedes normanensis, Aedes notoscriptu*s, and even *Culicoides* midges have been reported (2, 3). The transmission vectors are the same as described for the Ross River virus (RRV) (4); therefore, both of these viruses share the same endemic areas. Marsupials (such as kangaroos, wallabies, possums) and horses are shown to be the potential nonhuman vertebrate hosts for BFV (2).

BFV is the second most prevalent arbovirus in Australia, with approximately 1,000 cases reported per year and over 16,000 confirmed cases since 2000 (Australian Government Department of Health National Notifiable Diseases Surveillance System) (5). BFV infection in humans was first documented in 1986 in New South Wales, Australia (6). Subsequently, large outbreaks were identified over the next decade in the Northern Territory in 1992 (7), Western Australia in 1993–1994 (8), New South Wales in 1994–1995 (9), Victoria in 2002 (10), and Southeastern Queensland in 2002–2003 (11). BFV infection was believed to be exclusive to Australia, but a case of BFV infection in a child from Papua New Guinea (PNG) with no travel history was reported in 2014 and the PNG isolate was found to be phylogenetically divergent from the known Australian BFV strains (12).

Symptoms of BFV infection include fever, headache, tiredness or fatigue, joint pain or swelling, rash, muscle pain, and swollen lymph nodes, which in some cases may last for periods of 6 months or more. Symptoms are similar to those associated with other arthritogenic alphavirus infections, such as RRV, chikungunya virus (CHIKV), o’nyong-nyong (ONNV), and Mayaro (MAYV) viruses (13–15). BFV disease differs slightly from RRV disease in that there is less joint swelling, a more prominent and intense rash, and a lower prevalence of chronic diseases (16). A patient presenting with the aforementioned symptoms in endemic areas is usually tested serologically for both viruses due to overlapping symptoms (2, 16). An analysis of the symptoms of BFV infection and RRV infection in a mouse model revealed that BFV infection exhibited milder musculoskeletal inflammation (17, 18).

The BFV genome has a length of ~11.5 kb and contains two major open reading frames (ORFs). The first ORF encodes non-structural (ns) polyproteins P123 and P1234, which are precursors for ns-proteins (nsP1–nsP4). The second ORF encodes the structural polyprotein expressed from sub-genomic RNA synthesized in infected cells. BFV is a sole representative of the Barmah Forest complex within the *Alphavirus* (19). The amino acid sequence similarity of BFV nsPs is highest to those of RRV and Semliki Forest virus (SFV) (20, 21), 62% and 63%, respectively. However, the sequences of BFV E2, 6K, and E1 proteins exhibit a significant degree of similarity to the alphaviruses of Western, Eastern, and Venezuelan equine encephalitis complexes (19, 22). While some molecular characteristics of BFV have been described, such as lower glycosylation of E2 compared to RRV, SFV, and SINV (20), little information is currently available regarding virus-host interactions of BFV nsPs. In addition, molecular virology tools vital to identifying BFV replication and pathogenic mechanisms have been lacking.

Enzymatic functions of alphavirus nsPs, that are subunits of the RNA replicase complex (RC), are well conserved (23). In addition, nsPs possess less conserved functions (24). Similarly, the interactions of nsPs with host proteins vary for different alphaviruses (23). For example, in addition to being a component of the RC, nsP3 is a key interaction partner for host proteins (25–27). nsP3 has been shown to exploit components of stress granules to regulate viral replication and counteract host defense (28–33). Similar to the majority of arthritogenic alphaviruses, the activity of the BFV RNA replicase is dependent on host G3BP proteins (34). These interactions are mediated by the C-terminal hypervariable domain (HVD) of nsP3. In addition to HVD, nsP3 contains two structured domains: the N-terminal macro domain and the central alphavirus unique domain (AUD) (35). The nsP3 macro domain can hydrolyze ADP-ribose groups from mono-ADP-ribosylated proteins, and this feature is critical for CHIKV replication and virulence in mice (36–38). The nsP3 macro domain is also essential for the neurovirulence of SINV and SFV (39–41). In addition, nsP3 is also involved in determining alphavirus vector specificity (42).

Herein, we describe the construction of an infectious cDNA (icDNA) clone of BFV. The clone-derived virus (BFV-IC) and BFV field isolate (BFV2193-FI) showed similar *in vitro* and *in vivo* properties. During the construction of the infectious clone, a mistake leading to V1911D substitution in nsP4 was identified in the Genbank reference sequence. This specific mutation was found to result in low infectivity compared to BFV2193-FI. Notably, another mutation, T1325P in nsP2, emerged in the P_1_ passage of the virus harboring the nsP4-V1911D substitution. nsP2-T1325P was shown to be a second-site compensatory mutation counteracting the effects of nsP4-V1911D. These mutations were linked to alterations in the processing of viral ns polyproteins and the induction of type I interferon (IFN). In infected mammalian cells, BFV nsP3 was found to localize to the nucleus; however, this localization was not observed in infected mosquito cells. A point mutation abolishing nsP3 nuclear localization resulted in a viable virus that had attenuated growth in mouse embryonic fibroblasts (MEF) and *in vivo* compared to the BFV2193-FI. The infectious clone of BFV facilitated the identification of a nuclear localization signal in nsP3 associated with BFV pathogenicity, and two critical amino acids in nsP2 and nsP4 that are essential for virus replication.

## RESULTS

### Construction of icDNA of BFV

**Fig 1:**
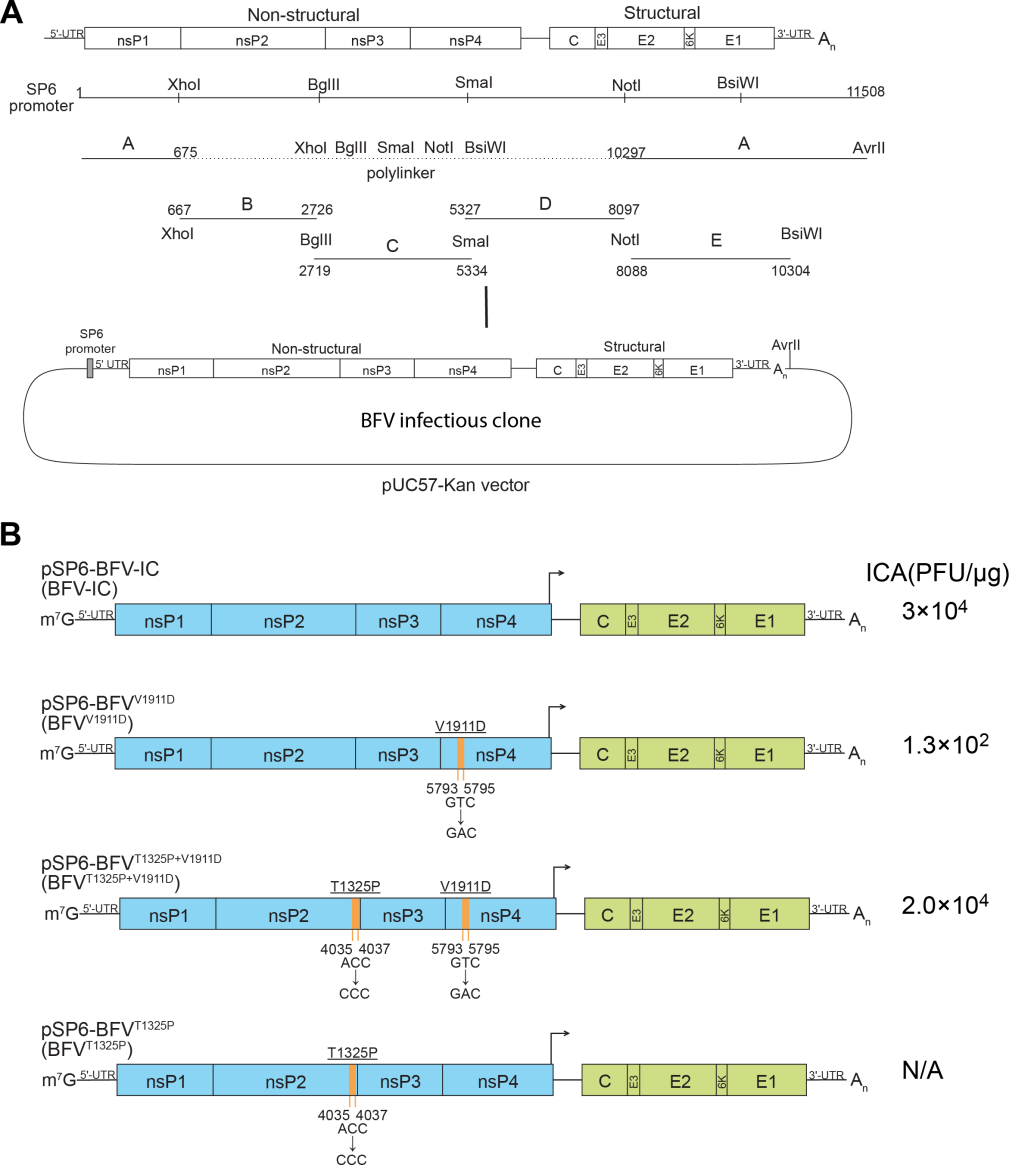
Construction of BFV icDNA clones. (**A**). Schematic presentation of a plasmid containing BFV icDNA. pUC57-Kan was used as a backbone, and five DNA fragments covering the complete BFV genome (11.5 kb) were synthesized and assembled using digestion with indicated restriction enzymes (XhoI, BgIII, Smal, NotI, and BsiWI). In fragment A, the 5′ end of the BFV genome was placed under an SP6 promotor, and a unique AvrII site, used for plasmid linearization, was placed immediately downstream of poly(A) sequence of virus genome. (**B**). Schematic presentation of recombinant BFV genomes, BFV-IC, BFV^V1911D^, BFV^T1325P+V1911D^, and BFV^T1325P^. Amino acid substitutions are shown above, and nucleotide substitutions are below the drawings; for amino acid residues, the numbers indicate their positions in P1234 of BFV-IC. Arrows indicate the sub-genomic promoter of BFV. The numbers at the right of the drawing indicate infectivity of corresponding RNA transcripts (PFU/μg of RNA) in the ICA, N/A—not analyzed.

BFV2193-FI, the prototype strain of BFV is the most virulent strain among all BFV variant isolates, with the highest mortality in newborn Swiss outbred mice (17). Therefore, it was chosen for constructing the icDNA clone of BFV. The sequence of BFV2193-FI was obtained from a complete genome sequence (GenBank: U73745.1) that was corrected by adding one missing residue based on the available partial sequence of the isolates 3′ untranslated region (UTR) (GenBank: AF339488.1). The infectious clone was assembled from five synthetic DNA fragments (Fig. 1A). Infectious center assay (ICA) revealed that infectivity of the corresponding RNA transcripts of the clone was low, ~100 PFU/μg of RNA (Fig. 1B; clone pSP6-BFV^V1911D^). The rescued virus was then amplified to generate P_1_ stock and sequenced. Compared to the icDNA sequence, a single substitution of T to P was detected at position 792 of nsP2 (corresponds to residue 1325 of ns polyprotein P1234, hereafter T1325P) in the P_1_ stock. Introduction of the T1325P substitution to the icDNA increased the infectivity of corresponding RNA transcripts by approximately 100-fold (Fig. 1B; clone pSP6-BFV^T1325P+V1911D^). The T1325P substitution is at residue P7 of the cleavage site between nsP2 and nsP3 (2/3 site). As the 2/3 site represents a hotspot of adaptive mutations generated by alphaviruses in response to various defects in the virus ns-proteins (43), it was considered that nsP2-T1325P most likely represents an adaptive mutation compensating for the low infectivity of the virus rescued from original icDNA clone.

Our data indicated that sequences available in GenBank had at least one mistake that, when present in the viral genome, drastically reduced its replication. To confirm this, we performed next-generation sequencing (NGS) of the BFV2193-FI genome and identified three synonymous and three non-synonymous differences: nsP4-V1911D, E2-H383N, and E1-A826G in the U73745.1 sequence (Table 1). We therefore hypothesized that the low infectivity of transcripts generated from the infectious clone might be related to the non-synonymous difference in the nsP4 region. To test whether the difference in the ns-protein region affects virus phenotype, we reversed the V1911D substitution in the infectious clone. We found that the presence of V1911 residue increased the infectivity of corresponding transcripts to ~3 × 10^4^ PFU/μg of RNA (Fig. 1B, clone pSP6-BFV-IC). The apparent high infectivity confirms that the mistake in U73745.1, resulting in a nsP4 residue substitution, was the reason for the low infectivity of original icDNA transcripts and suggests that the two differences in E2 and E1 were not essential to virus infectivity. This modified infectious clone, containing E2-H383N and E1-A826G compared to BFV2193-FI, was designated as pSP6-BFV-IC, the original icDNA clone as pSP6-BFV^V1911D^ and its variant with compensatory change in nsP2 as pSP6-BFV^T1325P+ V1911D^ (Fig. 1B).

**TABLE 1:** Comparison of the NGS-derived sequence of BFV2193-FI and the BFV genome sequence of U73745.1

NGS of BFV2193-FI	U73745.1	Nucleotide position(s)^*a*^	Amino acid position^*b*^	Protein	Substitution
GAG	GAA	3428	1122*	nsP2	None
TTC	TTT	5090	1676*	nsP3	None
GTC	GAC	5794	1911*	nsP4	V to D
TTT	TTC	6521	2154*	nsP4	None
CAC	AAC	8473	383**	E2	H to N
GCG	GGC	9803, 9804	826**	E1	A to G

^*a*^ Nucleotide positions are represented with respect to the full-length viral genome.

^*b*^ Amino acid positions mark the residues within the context of the unprocessed ns (*) and structural (**) polyproteins.

### BFV-IC and BFV2193-FI have similar *in vitro* and *in vivo* phenotypes

**Fig 2:**
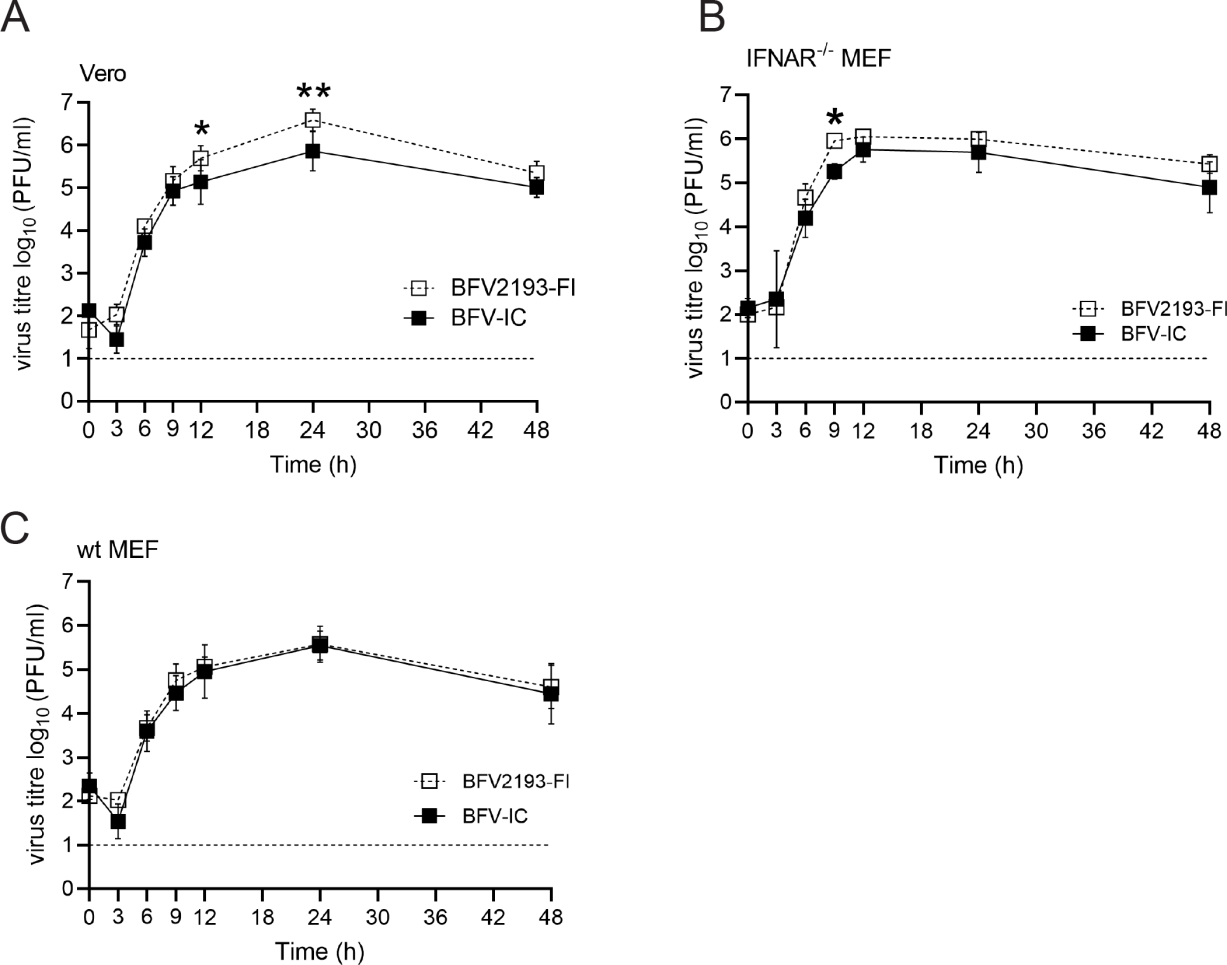
Comparison of *in vitro* phenotypes of BFV2193-FI and BFV-IC. Multi-step growth curves of BFV2193-FI and BFV-IC. Vero (**A**), IFNAR^-/-^ MEF (**B**), and WT MEF (**C**) cells were infected at an MOI of 0.1 and supernatants were collected at 0, 3, 6, 9, 12, 24, and 48 h p.i. Viral titers were determined by plaque assay and are represented as PFU/mL. The dotted line represents the limit of detection. Each data point represents the mean ± standard deviation (SD) from two independent experiments performed on triplicates. *P < 0.05 and **P < 0.01 using two-way ANOVA with a Bonferroni *post hoc* test.

**Fig 3:**
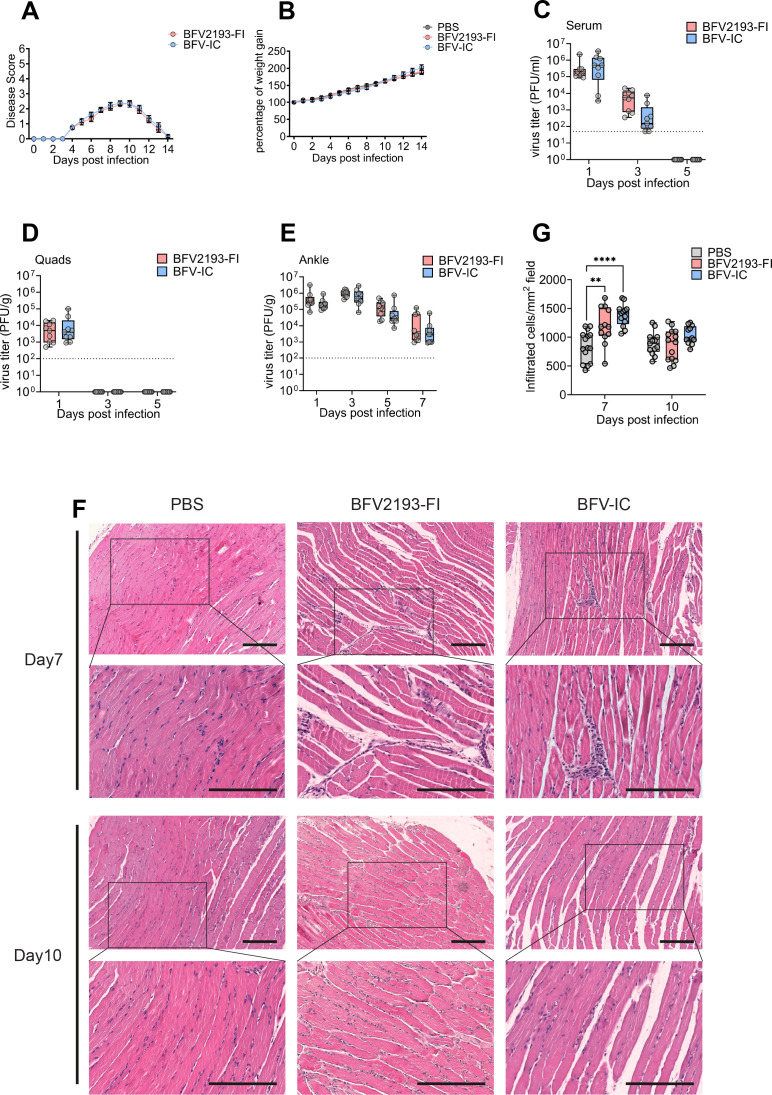
Comparison of *in vivo* phenotypes of BFV2193-FI and BFV-IC. C57BL/6 mice (*n* = 5) were infected subcutaneously with 10^5^ PFU of BFV2193-FI or BFV-IC or mock infected with PBS. Mice were assessed for disease score (**A**) and weight gain (**B**). Serum (**C**), quadriceps (**D**), and ankle (**E**) were collected on days 1, 3, and 5 p.i. Viral titers in the tissues were determined by plaque assay. The dotted line represents the limit of detection. H&E staining of longitudinal sections of quadriceps from the infected mice was performed at 7 and 10 days p.i. (**F**). Scale bar = 200 µm. The cell infiltrates were quantified using ImageScope (**G**). **P < 0.01; ****P < 0.0001 using two-way ANOVA with a Bonferroni *post hoc* test.

To reveal whether the phenotype of BFV-IC, the virus generated from pSP6-BFV-IC, matches that of BFV2193-FI, we compared the *in vitro* replication kinetics of these viruses. In Vero cells, BFV2193-FI showed a small growth advantage over BFV-IC (Fig. 2A) and to a lesser extent, this was also observed in interferon-alpha/beta receptor knockout (IFNAR^-/-^) MEF cells (Fig. 2B). However, no difference was observed in type I IFN-competent wild type (wt) MEF cells (Fig. 2C). The disease scores for C57BL/6 mice infected with BFV-IC and BFV2193-FI were highly similar, with disease onset at day 4 post-infection (p.i.), peak disease at day 10 p.i., and resolution by day 14 p.i. (Fig. 3A). BFV-IC or BFV2193-FI-infected mice did not exhibit a significant difference in weight gain (Fig. 3B), which confirmed the mild nature of BFV infection in a mouse model (17). No significant differences were found in virus replication kinetics in serum, quadriceps, and ankle joints of mice infected with BFV-IC or BFV2193-FI (Fig. 3C through E). Histological analysis showed a comparable level of tissue pathology and inflammatory cell infiltration in BFV-IC- and BFV2193-FI-infected mice (Fig. 3F and G). Based on these results, we conclude that BFV2193-FI and BFV-IC exhibit identical growth phenotypes in infected type I IFN-competent primary cells and disease phenotypes in WT-infected mice.

### A combination of nsP2-T1325P and nsP4-V1911D substitutions is associated with virus attenuation in mouse and mosquito cells

**Fig 4:**
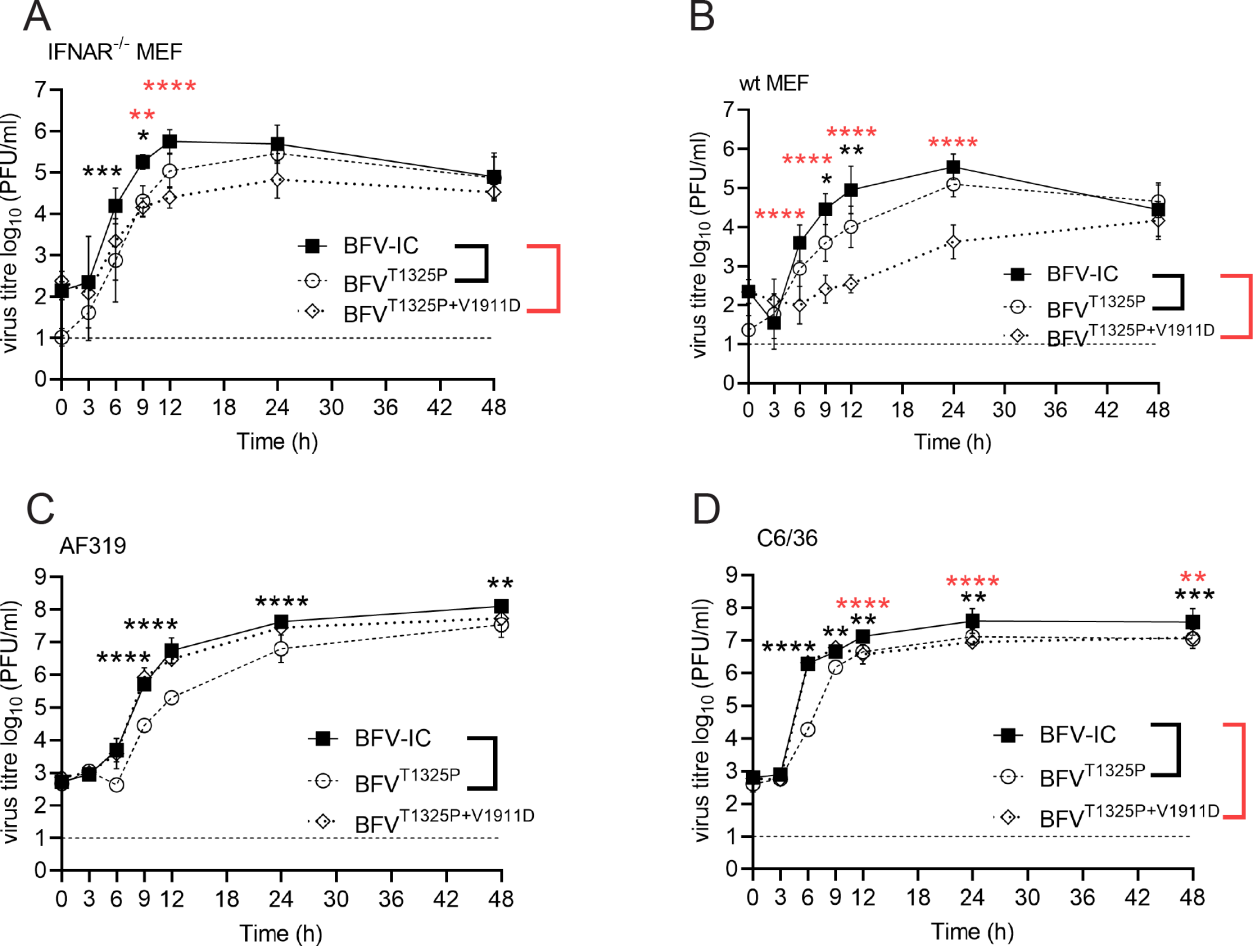
nsP2-T1325P and nsP4-V1911D substitutions attenuate BFV *in vitro*. IFNAR^-/-^ MEF (**A**), WT MEF (**B**), AF319 (**C**), and C6/36 (**D**) cells were infected with BFV-IC, BFV^T1325P^, and BFV^T1325P+V1911D^ at an MOI 0.1. Samples were collected and analyzed by plaque assay. The dotted line represents the limit of detection. Each data point represents the mean ± SD from two independent experiments performed in triplicates. *P < 0.05; **P < 0.01; ***P < 0.001, and ****P < 0.0001 using two-way ANOVA with a Bonferroni *post hoc* test.

As described previously, the mutation nsP4-V1911D was found to be the reason for the low infectivity of corresponding RNA transcripts (Fig. 1B). Interestingly, a spontaneously occurring second-site mutation, nsP2-T1325P, increased rescue efficiency by ~100-fold, suggesting that nsP2-T1325P could be an adaptive mutation when nsP4-V1911D is present. The significance of the two mutations, nsP2-T1325P and nsP4-V1911D, was therefore further investigated. Infectious clone pSP6-BFV^T1325P^ was generated by site-direct mutagenesis, producing BFV^T1325P^ (Fig. 1B). The nsP2-T1325P mutation occurs in the vicinity of the nsP2/3 cleavage site, that is, in a position associated with reduced replication of alphaviruses and their increased sensitivity to type I IFN responses (44, 45). To investigate whether this is also the case for BFV, the growth kinetics of BFV^T1325P^ and BFV^T1325P+V1911D^ were compared to that of BFV-IC in IFNAR^-/-^ MEF and WT MEF cells. It was observed that in IFNAR^-/-^ MEF cells, both BFV^T1325P^ and BFV^T1325P+V1911D^ grew slightly slower than BFV-IC; the difference was significant at early time points (Fig. 4A). From 12 h p.i. onward, BFV^T1325P^ titers exceeded those of BFV^T1325P+V1911D^, indicating an additional negative impact of the nsP4-V1911D substitution on BFV replication (Fig. 4A). This effect was even more prominent in WT MEFs where titers of BFV^T1325P+V1911D^ were consistently lower than BFV^T1325P^ from 6 h to 48 h p.i. (~1.5–2 log lower at 12 and 24 h p.i., statistically significant at both time points with *P* < 0.0001, Fig. 4B). The results indicate that nsP2-T1325P has, at least on its own, little impact on the type I IFN sensitivity of BFV. By contrast, the combination of nsP2-T1325P and nsP4-V1911D substitutions (e.g., the combination of mutations generated during virus rescue and adaptation) potentially increased the type I IFN sensitivity of BFV.

Slowdown of 2/3 site processing has been shown to increase CHIKV RNA replication in mosquito cells (46). To determine whether BFV^T1325P^ and BFV^T1325P+V1911D^ have similar phenotypes in mosquito cells, their growth curves in *Aedes aegypti* AF319 cells and *Aedes albopictus* C6/36 cells were compared to those of BFV-IC. No accelerated growth of mutant viruses was observed in either of these cell lines. In AF319 cells, BFV^T1325P^ was slightly attenuated, while the growth of BFV^T1325P+V1911D^ was almost identical to that of BFV-IC (Fig. 4C). In C6/36 cells, similar differences between the phenotypes of BFV-IC and the two mutant viruses were observed only at early time points. From 12 h p.i. onward, titers of both mutant viruses were similar but statistically significantly lower than those of BFV-IC (Fig. 4D). Thus, in contrast to MEFs, the combination of nsP2-T1325P and nsP4-V1911D mutation in mosquito cells was better tolerated than the nsP2-T1325P mutation alone.

### nsP3 of BFV contains a functional nuclear localization signal

**Fig 5:**
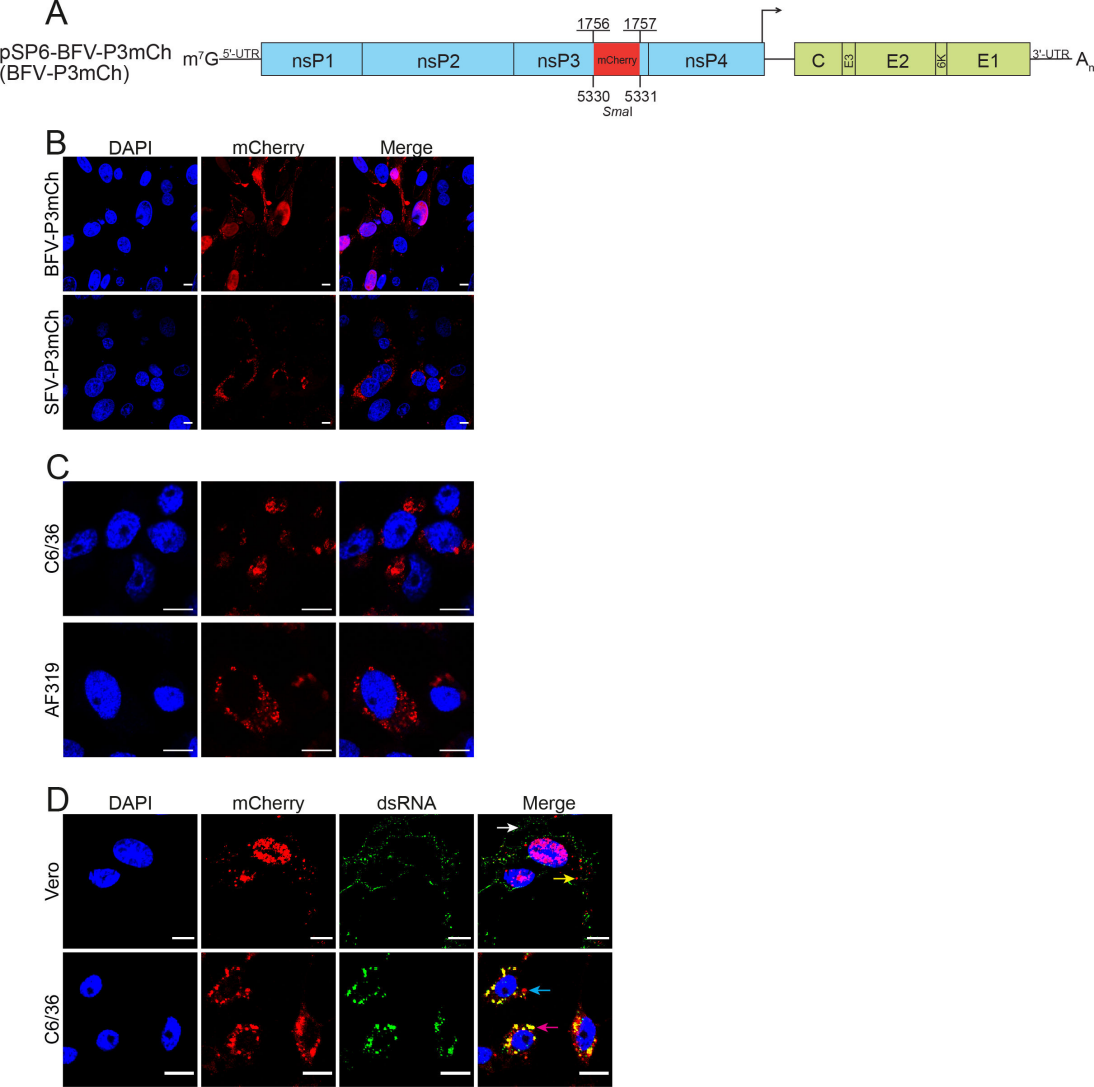
Subcellular localization of BFV nsP3. (**A**) Schematic presentation of the genome of BFV-P3mCh. Inserted sequence encoding for mCherry is shown in red. The arrow indicates the sub-genomic promoter of BFV. (**B**) BHK-21 cells were infected with BFV-P3mCh or SFV-P3mCh (as a control) at an MOI of 1.0. Cells were fixed 24 h p.i. Nuclei were counterstained with DAPI, and cells were analyzed for mCherry fluorescence using a Zeiss LSM710 confocal microscope. (**C**) C6/36 and AF319 cells were infected with the BFV-P3mCh at an MOI of 1.0. Cells were fixed 24 h p.i. Nuclei were counterstained with DAPI, and cells were analyzed for mCherry fluorescence using a Zeiss LSM710 confocal microscope. (**D**) Vero cells and C6/36 cells were infected with BFV-P3mCh at an MOI of 1.0, fixed 24 h p.i. and treated with dsRNA-specific mouse monoclonal J2 antibody as primary antibody and anti-mouse Alexa Fluor 488 conjugated antibody as the secondary antibody. In addition to mCherry and DAPI fluorescence, cells were analyzed for dsRNA signal using a Nikon N1R + confocal microscope. Scale bar = 10 µm.

Previous studies with alphaviruses have shown that marker proteins inserted into the HVD of nsP3 do not affect the subcellular localization of nsP3 in infected cells (47, 48). To enable visual detection of virus-infected cells, an infectious clone pSP6-BFV-P3mCh, encoding an nsP3-mCherry fusion protein (mCherry was inserted between residues 1756 and 1757 of P1234), was constructed (Fig. 5A). Consistent with previous observations (49), an analysis of BFV-P3mCh infected mammalian cells revealed that in addition to the usual focal cytoplasmic localization pattern, nsP3-mCherry also prominently localized into the nuclei of infected cells (Fig. 5B; Movie S1). To demonstrate that this is a phenomenon specific to BFV, recombinant SFV-P3mCh was constructed and used for comparison. No nuclear localization of nsP3-mCherry was observed in cells infected with this virus (Fig. 5B). Interestingly, in C6/36 and AF319, mosquito cells infected with BFV-P3mCh nuclear localization of nsP3 was not observed (Fig. 5C), indicating that localization and possibly the functions of BFV nsP3 are different in mammalian and mosquito cells.

In the cytoplasm of alphavirus-infected cells, nsP3 has been detected in different complexes, only part of which are involved in virus RNA replication (44, 50). Therefore, the localization of nsP3-mCherry was compared to that of double-stranded RNA (dsRNA), a marker for alphavirus RNA replication. As expected, in BFV-P3mCh-infected Vero and C6/36 cells, the dsRNA was localized in the cytoplasm (Fig. 5D). In Vero cells, dsRNA was detected in small foci primarily located at the cell periphery (white arrow). In general, the signal of nsP3-mCherry was not sufficiently strong enough to confirm its presence in these foci. Conversely, many nsP3-mCherry-positive foci in the cytoplasm did not contain detectable amounts of dsRNA (yellow arrow). This type of dsRNA and nsP3 localization resembles that previously observed in the cytoplasm of CHIKV-infected mammalian cells (50) and is consistent with the ability of nsP3 to form different cytoplasmic granules. Interestingly, however, in C6/36 cells, nsP3-mCherry and dsRNA localized in large cytoplasmic vesicles (magenta arrow). Virtually, all dsRNA-positive vesicles were also positive for nsP3-mCherry, indicating that these structures contain BFV replication complexes. At the same time, nsP3-mCherry foci, lacking detectable dsRNA staining, were also abundant (cyan arrow), indicating that BFV nsP3 forms different complexes in mosquito cells.

**Fig 6:**
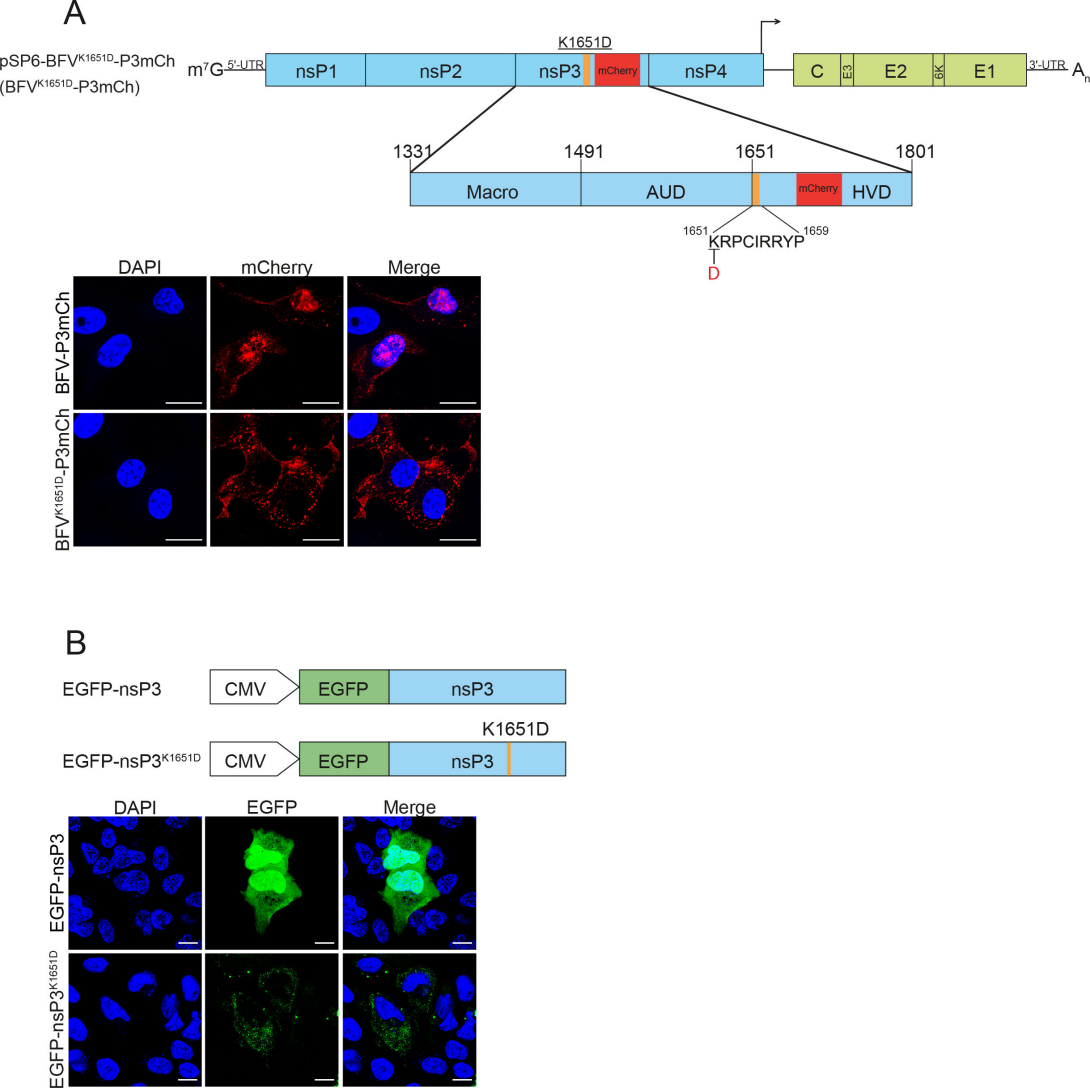
nsP3 of BFV contains a functional NLS. (**A**). Upper panel: Schematic presentation of recombinant BFV genomes. Macro-domain, AUD, HVD, and sequence of predicted NLS are shown. Amino acid positions of domain boundaries and NLS are according to these in P1234. Amino acid substitutions are shown above, and nucleotide substitutions are below the drawings. Inserted sequence encoding for mCherry is shown in red. Arrows indicate the sub-genomic promoter of BFV. Lower panel: Vero cells were infected with BFV-P3mCh or BFV^K1651D^-P3mCh at an MOI of 1.0. Cells were fixed 24 h p.i. Nuclei were counterstained with DAPI. Cells were analyzed for mCherry fluorescence using a Zeiss LSM710 confocal microscope. (**B**) Upper panel: Schematic presentation of constructs expressing EGFP-nsP3 fusion proteins. Lower panel: U2OS cells were transfected with expression constructs encoding for BFV EGFP-nsP3 and EGFP-nsP3^K1651D^ fusion proteins. Cells were fixed 24 h post-transfection. Nuclei were counterstained with DAPI. Cells were analyzed for EGFP fluorescence using a Zeiss LSM710 confocal microscope. Scale bar = 20 µm.

Analysis of BFV nsP3 using an NLS prediction software tool NLStradamus (http://www.moseslab.csb.utoronto.ca/NLStradamus) identified a potential NLS sequence at the boundary of the AUD and HVD. As Arg and Lys residues in NLS are crucial for the nuclear import of proteins (51), a mutation resulting in the K1651D substitution was introduced into pSP6-BFV-P3mCh (Fig. 6A). In Vero or MEF cells infected with BFV^K1651D^-P3mCh, no nuclear localization of nsP3^K1651D^-mCherry was observed (Fig. 6A; Fig. S1; Movie S2). This finding was also confirmed using a virus-free system. The WT nsP3 of BFV fused to EGFP (EGFP-nsP3) was found to localize prominently in the nuclei of transfected U2OS cells. By contrast, the localization of EGFP-nsP3^K1651D^ was cytoplasmic (Fig. 6B). Thus, BFV nsP3 contains an NLS functional in different mammalian cells.

### Disruption of BFV nsP3 nuclear localization leads to slow replication in type I IFN- competent cells

**Fig 7:**
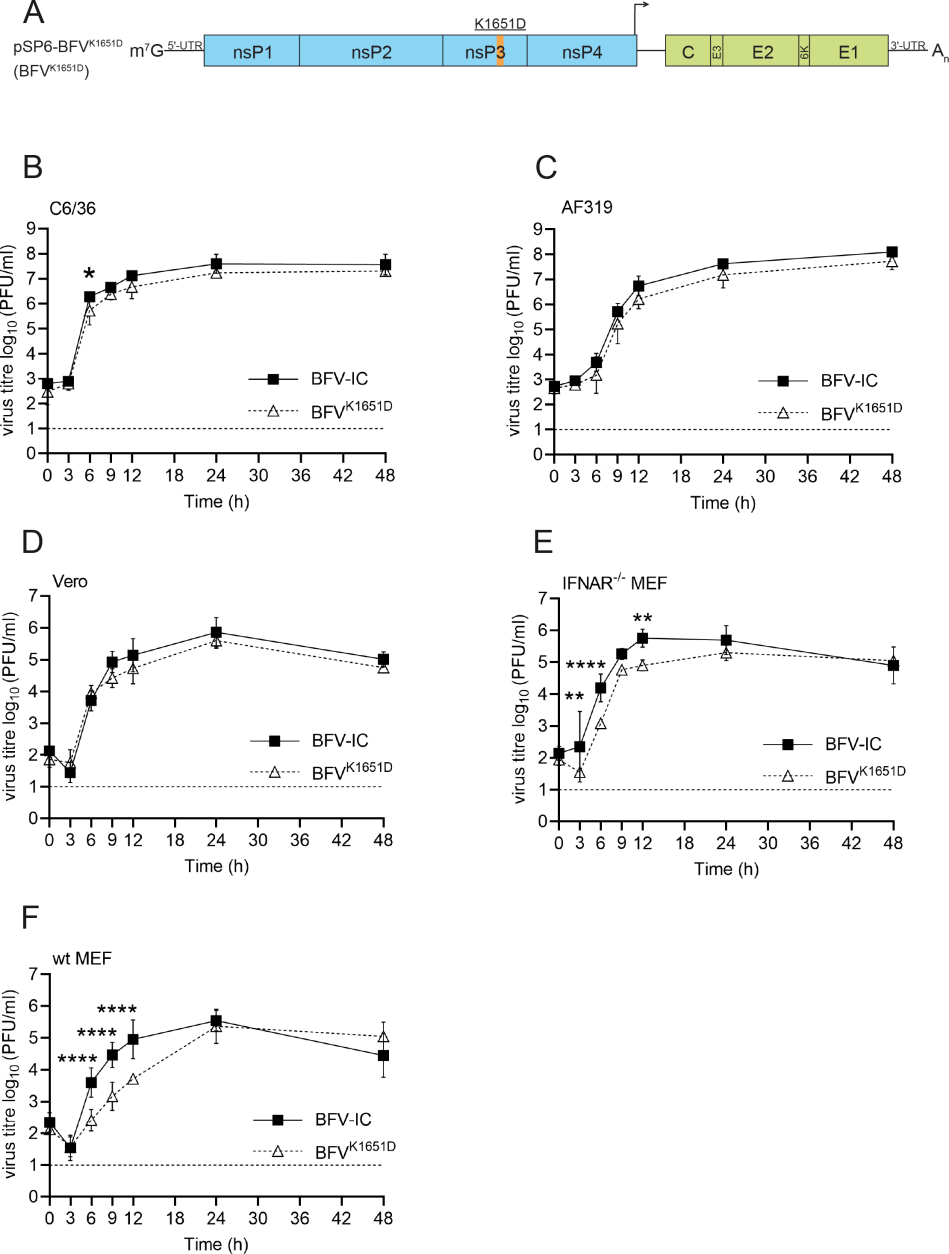
BFV^K1651D^ replicates well in mosquito and Vero cells but is attenuated in MEF cells. (**A**). Schematic presentation of recombinant BFV genomes. Amino acid substitutions are shown above the genome. Arrows indicate the sub-genomic promoter of BFV. C6/36 (**B**), AF319 (**C**), Vero (**D**), IFNAR^-/-^ MEF (**E**), and WT MEF (**F**) cells were infected with BFV-IC or BFV^K1651D^ at an MOI of 0.1. Viral titers in the cell culture were determined by plaque assay. The dotted line represents the limit of detection. Analysis was performed and data are presented as described in Fig. 2. *P < 0.05; **P < 0.01, and ****P < 0.0001 using two-way ANOVA with a Bonferroni *post hoc* test.

The nuclear localization of alphavirus nsP2 and capsid proteins are associated with the inhibition of host cell antiviral responses, including the type I IFN response (52, 53). Conversely, blocking the nuclear localization of these proteins results in the attenuation of alphaviruses in cells harboring an active type I IFN system (54–56). To determine whether the same may apply to nsP3 of BFV, the BFV^K1651D^ mutant virus (Fig. 7A), lacking mCherry but harboring nsP3-K1651D substitution, was constructed and compared to BFV-IC. In mosquito-derived C6/36 and AF319 cell lines, BFV^K1651D^ replicated similarly to BFV-IC. The titers of the mutant virus were marginally reduced, and the differences were largely statistically insignificant, except for the 6-h time point in C6/36 cells (Fig. 7B and C). This suggests that the nsP3-K1651D substitution has minimal effect on BFV replication in mosquito cells and reflects the lack of nsP3 nuclear localization in these cells (Fig. 5C). The mutation also had no significant effect on BFV replication in Vero cells, which cannot produce type I interferons (Fig. 7D). Surprisingly, the same was not observed in IFNAR^-/-^ MEFs. In these cells, BFV^K1651D^ titers were significantly lower at 3, 6, and 12 h p.i. (Fig. 7E); the difference was observed to be approximately sevenfold at 12 h p.i. The slower growth of BFV^K1651D^ may indicate that in primary murine cells, nuclear localization of nsP3 is important during the early stages of BFV replication. In WT MEFs, an additional attenuation of BFV^K1651D^ was observed: virus titers exhibited a slower increase, and the shape of the growth curve differed from that of BFV-IC. Consistently, the differences in titers were approximately 20-fold at 9 h p.i. and approximately 17-fold at 12 h p.i. (Fig. 7F). Thus, type I IFN signaling slowed down the accumulation of infectious BFV^K1651D^ in MEF cell culture. BFV^K1651D^ reached similar titers to BFV-IC in WT and IFNAR^-/-^ MEF cells after 24 h p.i. (Fig. 7E and F), indicating that the mutation slowed virus replication without affecting the number of virions produced from one cell.

### Disruption of nsP3 nuclear localization attenuated BFV replication in a mouse model

**Fig 8:**
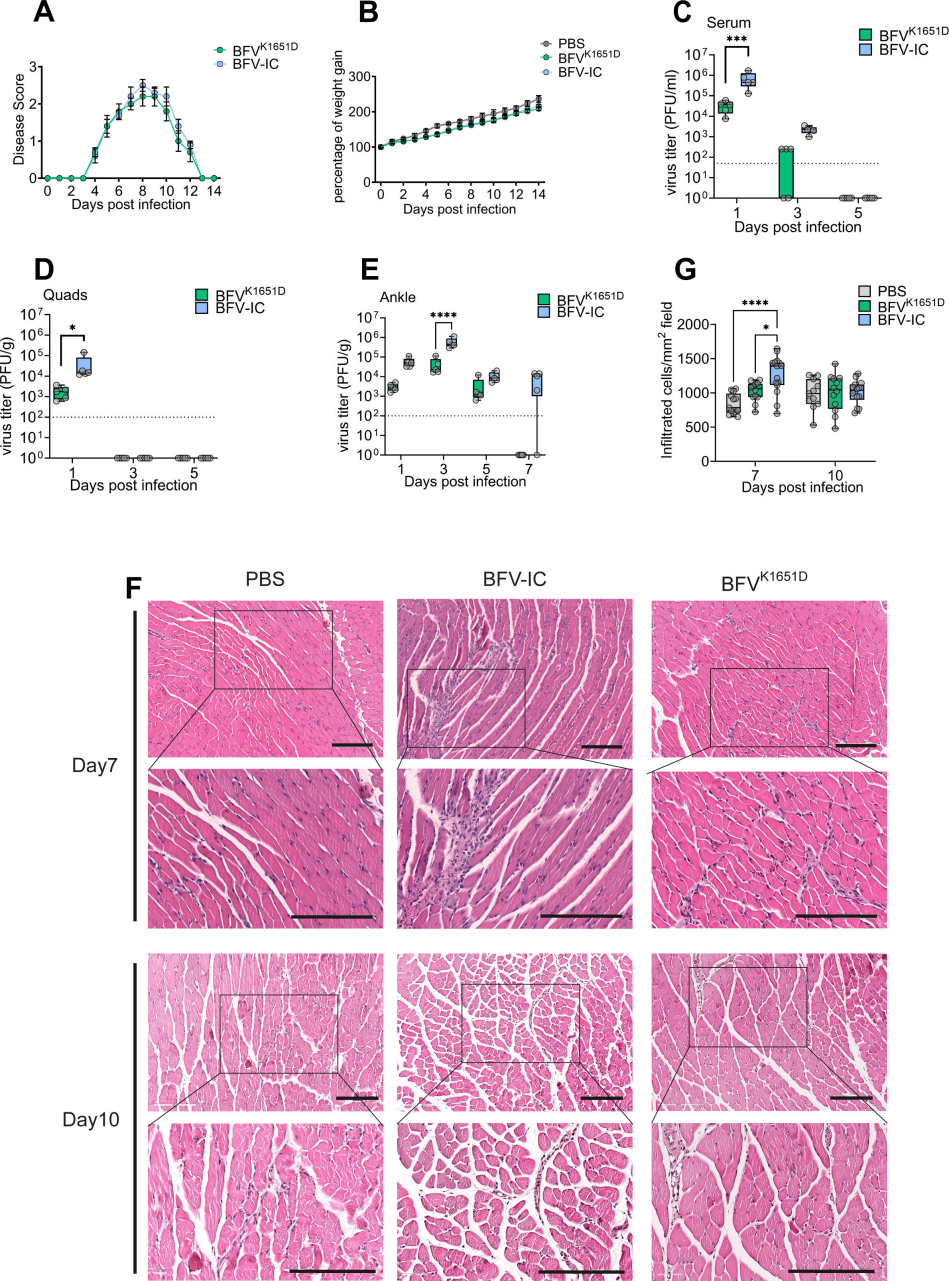
nsP3-K1651D substitution does not affect the disease severity but reduces BFV titers and cell infiltration in C57BL/6 mice. (**A and B**) C57BL/6 mice (*n* = 5) were infected subcutaneously with 10^5^ PFU of BFV-IC or BFV^K1651D^, and control mice (*n* = 5) were mock infected with PBS. Animals were monitored for (**A**) clinical disease symptoms or (**B**) weight gain. Mice (*n* = 4) were inoculated with 10^5^ PFU of BFV-IC or BFV^K1651D^. Serum (**C**), quadriceps (**D**), and ankle (**E**) were collected on days 1, 3, and 5 p.i. Viral titers in the tissues were determined by plaque assay. The dotted line represents the limit of detection. Statistical analysis was performed using two-way ANOVA with Bonferroni post-test (*P < 0.005; ***P < 0.001; ****P < 0.0001). H&E staining of longitudinal sections of quadriceps from the infected mice was performed at 7 and 10 days p.i. (**F**). Scale bar = 200 µm. The statistical analysis of cell infiltrates was quantified using ImageScope (**G**). *P < 0.05; ****P < 0.0001 using two-way ANOVA with Bonferroni *post hoc* test.

Mutations affecting alphavirus protein nuclear localization have been shown to result in attenuation *in vivo* (56). To verify whether this is also the case for BFV^K1651D^, the mutant virus was compared to BFV-IC in a mouse model (17). Disease in infected mice was first observed at day 4 p.i., peaked at day 8 p.i., and resolved by day 13 p.i. (Fig. 8A). No significant differences in the disease scores of BFV-IC- and BFV^K1651D^-infected mice were observed at any time (Fig. 8A). Similarly, no difference in weight gain between virus-infected and mock-infected animals was observed (Fig. 8B). By contrast, analysis of viremia revealed that at early stages of infection, BFV-IC titers were significantly higher than those of BFV^K1651D^ (Fig. 8C). Known target tissues for arthritogenic alphaviruses include the quadriceps and ankles. Therefore, corresponding samples were collected and assayed for viral titers. At day 1 p.i., BFV-IC titers in the quadriceps were significantly higher than those of BFV^K1651D^. Neither of the viruses was detectable at days 3 and 5 p.i. (Fig. 8D). In hind-limb ankle joints, BFV^K1651D^ was detectable up to day 5 p.i., while BFV-IC was still detectable at day 7 p.i. (Fig. 8E). The titers of BFV-IC were significantly higher at day 3 p.i. (Fig. 8E). Furthermore, BFV-IC induced higher levels of inflammatory cell infiltration in quadriceps than BFV^K1651D^ at day 7 p.i. (Fig. 8F and G). These findings suggest that despite causing disease with similar severity, replication of BFV lacking the nuclear localization of nsP3 was attenuated *in vivo*.

### Ns polyprotein processing and type I IFN induction by BFV replicases harboring nsP2-T1325P, nsP3-K1651D, and nsP4-V1911D substitutions

We have demonstrated that the three mutations nsP2-T1325P, nsP3-K1651D, and nsP4-V1911D are associated with reduced BFV replication. Our previous report showed that alphavirus replication is closely related to ns polyprotein processing and type I IFN induction (57). In this study, we used a trans-replication assay, viral protein pulse labeling assay, and co-transfection-based luciferase reporter gene assays to test whether these mutations lead to a change in ns polyprotein processing and type I IFN induction.

**Fig 9:**
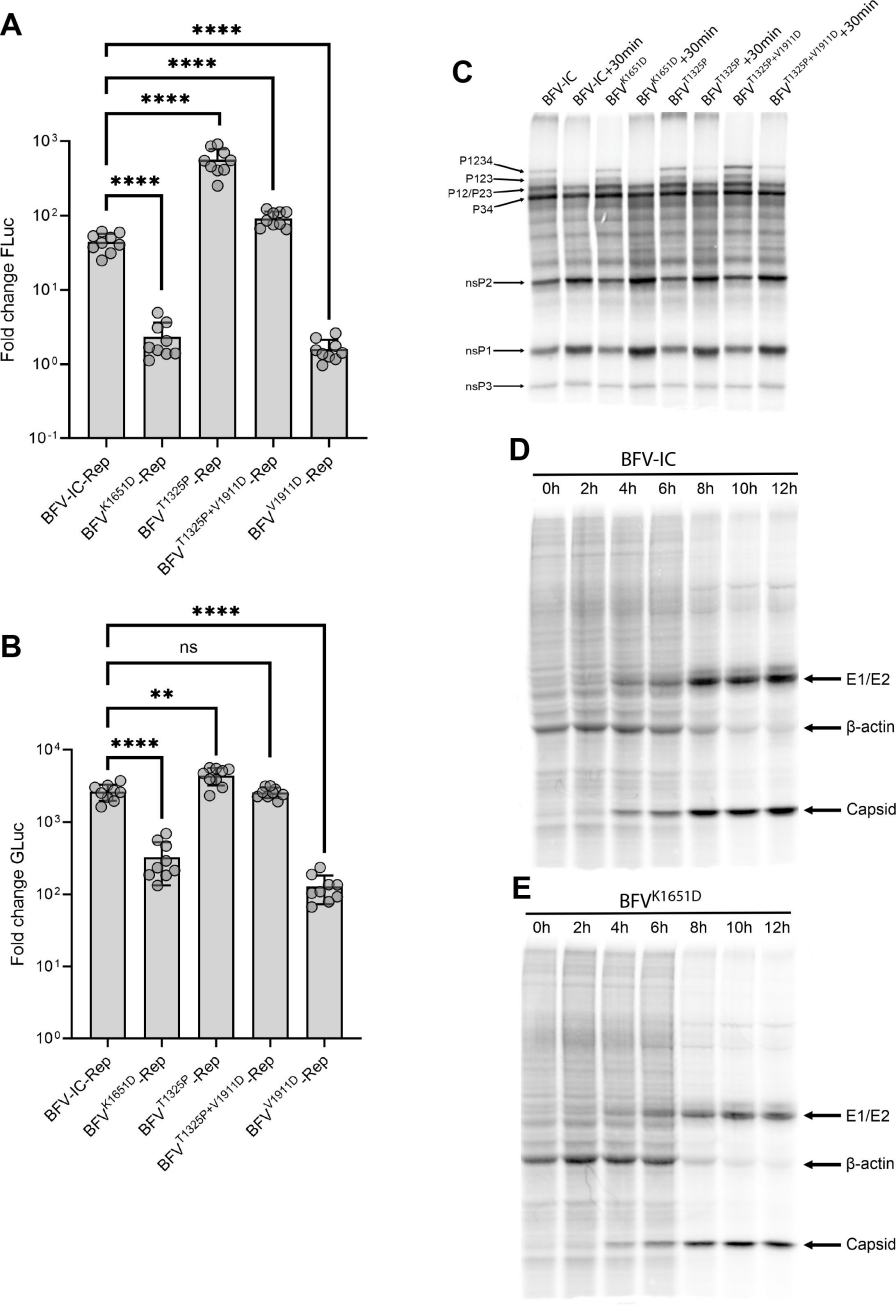
Impact of mutations in ns-polyprotein on the activity of BFV replicase, ns-polyprotein processing, and expression of viral structural proteins in infected cells. HEK293T cells were co-transfected with 0.5 µg of replicase expressing plasmids of BFV-IC, BFV^K1651D^, BFV^T1325P^, BFV^T1325P+V1911D^, and BFV^V1911D^ and 0.5 µg of HSPolI-FG-BFV; control cells were co-transfected with BFV-GAA and HSPolI-FG-BFV. The cells were collected at 18 h post-transfection, lysed, and the FLuc (**A**) and GLuc (**B**) signals were detected. The measured FLuc and GLuc values were normalized to those of the control transfection outputs. Each bar represents the mean ± SD from two independent experiments performed in triplicates. ns, not significant; **P < 0.01; ****P < 0.0001 using unpaired Student’s *t*-test. For *in vitro* translation assay (**C**), reactions were performed using 0.25 µg of icDNA plasmid of BFV-IC, BFV^K1651D^, BFV^T1325P^ or BFV^T1325P+V1911D^, 4 mM DTT, and 6 µCi of L-[^35^S]-methionine. The translation mixture was incubated for at 30°C for 45 min, then divided into two, where one part was denatured immediately, and the other was subjected to further incubation at 30°C for 30 min before being denatured. The proteins were resolved using SDS-PAGE and visualized using the Amersham Typhoon IP Biomolecular Imager. For pulse-labeling experiments, BHK-21 cells were infected with BFV-IC (**D**) or BFV^K1651D^ (**E**) at an MOI of 10. At indicated time points, cells were labeled by adding radioactive cysteine and methionine mixture at a final activity of 100 µCi/mL for 30 min. The collected samples were lysed and subjected to Benzonase nuclease treatment. Labeled proteins were separated by SDS-PAGE and visualized with the Amersham IP Biomolecular Imager.

The trans-replication assay used here is based on a previously published design (58) (Fig. S2) where the Firefly luciferase (Fluc) and Gaussia luciferase (Gluc) markers are encoded by the first and second ORFs of the template RNA, respectively. To differentiate between the expression of the two RNA transcripts, we refer to the replicase activity based on Fluc signal as RNA replication and Gluc signal as RNA transcription. As shown in Fig. 9A and B, the mutation nsP4-V1911D resulted in significantly lower levels of BFV replicase activity. nsP3-K1651D also strongly reduced RNA transcription and had a negative impact on RNA replication. Interestingly, compared to BFV-IC replicase, replicase harboring nsP2-T1325P substitution increased the efficiencies of BFV replicase. When this substitution was combined with nsP4-V1911D, the activities of the double-mutant replicase were similar to that of BFV-IC replicase (Fig. 9B), suggesting that the opposing effects of these substitutions canceled each other out. These results provided strong evidence, as we proposed based on the ICA results, that nsP2-T1325P is indeed an adaptive mutation compensating for the defect caused by nsP4-V1911D.

Processing of WT and mutant ns-polyproteins was analyzed using an *in vitro* translation assay. Expectedly, no noticeable differences in WT and K1615D ns polyprotein processing were observed. By contrast, ns polyproteins containing the T1325P substitution or a combination of T1325P and V1911D substitutions displayed slower processing, evidenced by slightly elevated levels of P1234 that remained detectable after a 30-min chase period (Fig. 9C). The data confirm that the T1325P mutation, located close to the 2/3 site, indeed slows processing of P1234. In addition, we performed pulse labeling of cells infected with BFV-IC and BFV-IC^K1651D^. No differences in the shutdown of host translation were observed as the expression of structural proteins of both viruses became detectable at the same time (4 h p.i.). However, the levels of E1/E2 glycoproteins and capsid protein synthesis were lower in BFV-IC^K1651D^-infected cells compared to BFV-IC-infected cells (Fig. 9D and E). These observations are in line with previous results showing the attenuated replication of BFV-IC^K1651D^ in MEF cells and in mice.

**Fig 10:**
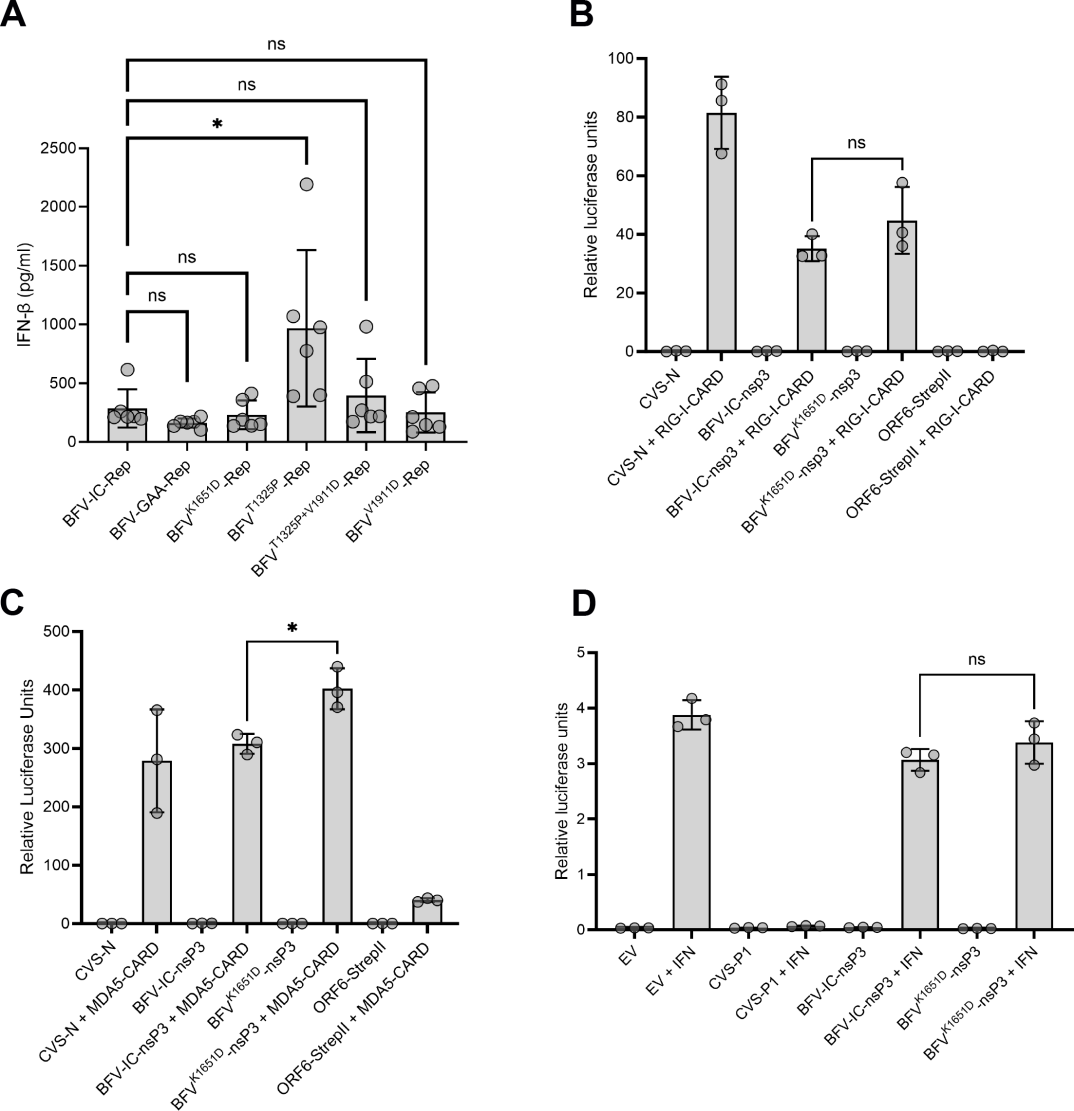
BFV nsp3 proteins lack substantial IFN-antagonist function. (**A**) IFN-β induction by non-classical viral PAMP RNA synthesized by BFV replicase. HEK293T cells were transfected with 1 µg of BFV replicase expression plasmids of BFV-IC, BFV^K1651D^, BFV^T1325P^, BFV^T1325P+V1911D^, and BFV^V1911D^. Control cells were co-transfected with BFV-GAA. Transfected cells were incubated at 37°C for 48 h and lysed for total RNA extraction. Five μg of RNA was used for another round of transfections in confluent COP5 cells. After 48 h of incubation, the supernatant was collected and assayed for IFN-β. Each bar represents the mean ± SD from two independent experiments performed in triplicates. *P < 0.05; **P < 0.01; ns, not significant using one-way ANOVA with a Bonferroni *post hoc* test. (**B–D**) Luciferase reporter gene assays for IFN induction (**B and C**) and IFN signaling (**D**). HEK-293T cells were transfected with pRL-TK, pGL3-IFNβ together with plasmids to express RIG I-CARD (**B**), MDA5-CARD (**C**), or empty vector (EV, non-stimulatory control) (**B and C**) and plasmid to express the indicated GFP-fused BFV nsP3 proteins or control proteins (GFP-CVS-N, ORF-6-StrepII), before analysis using a dual luciferase assays 40 h post-transfection. (**D**) HEK-293T cells transfected with pRL-TK plasmid, pISRE-luciferase plasmid, and plasmids to express the indicated GFP-nsP3 proteins or controls (GFP-CVS-P1 or EV). Cells were treated 8 h post-transfection with 1,000 U/mL IFN-α (18 h) before analysis using a dual luciferase assay. (**B–D**) FLuc activity (expressed from the IFNβ) (**B and C**) or ISRE (**D**) (promoter-containing plasmids) was normalized to RLuc activity (expressed from the TK promoter-containing plasmid) for each sample, as previously described (59). The data show mean ± SEM (*n* = 3 replicates) and are representative of three separate assays; statistical analysis was performed using Student’s *i*-test; *P < 0.05, ns, not significant.

To determine whether changes in ns polyprotein processing caused by these mutations could lead to a change in type I IFN induction, we tested the type I IFN induction capacity of BFV and mutant replicases. First, the ability of BFV replicases to induce type I IFN using the non-classical viral pathogen-associated molecular pattern (PAMP) RNAs was analyzed. As shown in Fig. 10A, the replicase of BFV IC induced type-I IFN at a significantly higher level compared to replicase with inactivated polymerase subunit (BFV-GAA) used as a negative control. The data indicate that similar to previously studied replicases of SFV, RRV, and SINV (57), the replicase of BFV also synthesizes non-classical viral PAMP RNAs. Interestingly, the replicase of BFV^T1325P^ induced significantly higher levels of IFN-β than the replicase of BFV-IC, indicating elevated synthesis of non-classical viral PAMP RNAs. By contrast, replicases of all the other mutants, including the nsP2-T1325*P* + nsP4-V1911D double mutant, induced IFN-β at levels comparable with the replicase of BFV-IC. The observation suggests that in addition to the activation of BFV replicase (Fig. 9A and B), the mutation nsP2-T1325P has an additional effect that could be a disadvantage in virus replication, which may explain the lower replication rates of BFV^T1325P^ in MEF cells.

As shown above, the replicase with nsP3-K1651D substitution did not induce higher levels of type I IFN compared to the BFV-IC replicase; however, the corresponding virus still has a significant growth defect in MEF cells (Fig. 7F) and *in vivo* (Fig. 8C through E). In an attempt to explain this phenotype, we further investigated whether the inactivation of BFV nsP3 nuclear localization by nsP3-K1651D substitution affects type I IFN induction or signaling. To assess this, we used luciferase reporter gene assays which measure activation of transcription by the IFNβ promoter (i.e., the IFN induction pathway) in response to overexpression of the CARD domains of RIG-I or MDA5 (Major pattern recognition receptors for cytoplasmic viral RNA PAMPs) which represent constitutively active RIG-I or MDA5 (Fig. 10B and C); or IFN-stimulated response element-dependent transcription (i.e., IFN signaling) in response to treatment of cells with IFNα (which activated STAT1-dependent pathways). N protein of rabies virus (RABV) strain CVS or empty vector was used as a negative control, while SARS-CoV-2 ORF6 and CVS P protein, defined antagonists of IFN induction and signaling, respectively, were used as positive controls and confirmed to strongly inhibit these pathways (Fig. 10B through D). Consistent with the previous observation, the reporter assays indicated that expression of BFV-nsP3 or BFV-nsP3^K1651D^ did not result in substantial inhibition of activation of the IFNβ promoter by RIG-I or MDA-5 (Fig. 10B and C). Furthermore, IFN-activated STAT1-dependent signaling was not significantly inhibited by BFV-nsP3 or BFV-nsP3^K1651D^ (Fig. 10D).

## DISCUSSION

Genetically, BFV is not closely related to the majority of alphaviruses. It does not belong to the alphavirus SF complex, which includes RRV, CHIKV, ONNV, and SFV. Our understanding of BFV replication and especially the interactions with host cells and organisms is very limited. Reverse genetics of alphaviruses represents an efficient approach for studying common and virus species-specific properties. Here we described the construction and use of a reverse genetics system for BFV.

The common caveat of developing infectious clones from synthetic DNA fragments is that manufacture is dependent on the quality of available sequence information. When the BFV infectious clone was designed, only one sequence of the complete genome and another partial sequence were available in GenBank. Abnormally low infectivity of the transcripts from the original BFV infectious clone indicated a possible mistake in the input sequences and forced us to perform an NGS analysis to identify the defect which turned out to be V1911D substitution in nsP4. Once the mistake in the original clone was corrected (V1911D), transcripts from BFV icDNA (pSP6-BFV-IC) became highly infectious and infectious virus with similar *in vitro* and *in vivo* phenotypes compared to BFV2193-FI was obtained. Moreover, the infectious construct showed good tolerance for genetic manipulation and reporter insertion, including mCherry, which will prove useful in various *in vivo* and *in vitro* studies.

The mCherry fused BFV nsP3 was found primarily in the cell nucleus, which is in line with previous observations (49). The significance of nsP3 nuclear localization on BFV replication was not universal, even in mammalian cells: in Vero cells unable to produce type I IFN, no significant difference in titers between BFV-IC and BFV^K1651D^ was observed. In murine primary cells, the differences were significant; however, the effect was temporary and detectable only at early time points. By 24 h p.i., BFV^K1651D^ reached titers very similar to BFV-IC, even in the presence of type I IFN signaling. Despite recent progress in understanding the functions of alphavirus nsP3 (38, 60, 61), many of its functions, especially those related to interactions with host cells, remain enigmatic. nsP3 interacts with many host factors, including several that are crucial for viral RNA replication. Interestingly, the essential host factors are virus- or virus-group-specific, including FHL1 (62) and factors normally associated with stress granule formation (30, 63). Thus far, the G3BP proteins remain the only host factors clearly shown to be essential for BFV RNA replication (34). In addition to enabling RNA replication, alphaviruses interact with host proteins to modify the cellular environment (64) and delay/prevent antiviral responses (65). Interestingly, many proteins shown to interact with alphavirus nsP3 or be present in virus replicase organelles are nuclear proteins (25, 66). Nuclear shuttling of alphavirus nsP2 and capsid proteins is essential for virus replication and pathogenesis (56, 67). It is not clear how nuclear localization of nsP3 facilitates virus replication. nsP3 of BFV does not, at least as an individual protein, have substantial ability to counteract type I IFN induction or signaling, and IFN induction and signaling in cells expressing nsP3 was not altered by mutation in the NLS (Fig. 10B through D). At the same time, *trans*-replication assays revealed that RNA replicase activities of BFV replicases harboring nsP3 with mutated NLS are reduced (Fig. 9A and B). This may imply that the NLS region is either directly important for RNA replication or that it facilitates replication in an indirect manner. Many host proteins, translocated from the nucleus to the cytoplasm during alphavirus infection, have antiviral properties (68). Thus, it is possible that nuclear localization of nsP3 of BFV counteracts the entry of these antiviral factors to the cytoplasm in the infected mammalian cells. Additional studies are required to find out whether or not this may be the case.

To our knowledge, BFV is the only alphavirus pathogenic to humans with a nsP3 that prominently localizes to nuclei. We have shown that nuclear localization is mediated by an NLS located at the boundary of AUD and HVD, occurring in mammals but not mosquito cells. The mechanistic reasons for the host-type specific activity of BFV nsP3 NLS remain unknown; possible reasons include cell-type-specific mode of action of the NLS, host-cell specific modifications of nsP3, and interaction of nsP3 with host-specific factors facilitating/interfering functioning of the NLS. The lack of nuclear localization in mosquito cells is not accidental, as the analysis of properties of BFV^K1651D^ revealed that in these cells, the NLS of nsP3 has minimal or no significance for BFV replication. BFV nsP3 nuclear localization was essential for *in vivo* replication and related to cell infiltration in inflamed tissues. However, a significant variation in overall disease symptoms was not observed due to the low disease scores. Nevertheless, the *in vivo* model produced data that are in good agreement with *in vitro* data and confirmed that replication of BFV^K1651D^ is slightly attenuated. To be specific, the model was sufficiently sensitive to reveal a reduction of viral titers due to the attenuating mutation and thus is applicable for *in vivo* analysis of attenuated BFV variants, including potential vaccine candidates.

The mistake in the input sequence from GenBank leading to V1911D substitution in nsP4 turned out to be helpful, as it allowed us to gain a unique insight into the molecular biology of BFV. This amino acid residue is located between the 97 amino acid residue long N-terminal domain of nsP4 and the classical RdRp fold that starts at approximately residue 150 (23, 69). To the best of our knowledge, the functions of the affected region have not been studied for any alphavirus. To note, it is likely that nsP4 of BFV has some unique properties as it is the only nsP4 of any alphavirus that cannot form a functional replicase complex with the P123 component of any heterologous alphaviruses (70, 71).

Interestingly, we found that a second-site adaptation in nsP2 compensated for the substitution in this region of nsP4. It has been previously observed that a mutation in the N-terminal region of nsP4 of SINV can be compensated with a substitution in the RNA helicase region of nsP2 (23). Furthermore, it has been shown that substitution in the catalytic region of CHIKV nsP4 (C483Y) and another in the protease region of nsP2 (G641D) are functionally linked (43, 72, 73). These reports suggested a functional interaction of alphavirus nsP2 and nsP4 which was later confirmed by a structural analysis of the alphavirus RNA replicase core (74). Interestingly, here in our study, the V1911D substitution in nsP4 was compensated for by a T1325P mutation that matches the P7 position of the 2/3 site, and the threonine is well conserved in the 38 BFV isolates collected over the past 50 years (Data S2). Within the replicase complex, this region is located distal from nsP4 (74) and the effect caused by the compensatory substitution should be indirect. Proline (P) is a rigid amino acid residue, and its presence was hypothesized to slow down the processing of the 2/3 site, an effect that was confirmed by *in vitro* translation assay (Fig. 9C). The slowdown of ns-polyprotein processing is one of the most common responses of alphaviruses to unfavorable mutations (43, 45); possibly because inhibition of the nsP2/3 site processing increases the stability of early replicase complex providing more time for the synthesis of negative-strand RNA (75, 76). Interestingly, while the mutation increased BFV replicase activity (Fig. 9A and B), the beneficial effects of nsP2-T1325P substitution alone on the virus infection were not observed in the cell lines used in this study. This may be attributed to the high IFN induction ability of this mutant due to elevated synthesis of non-classical viral PAMP RNAs (Fig. 10A). Instead, we obtained clear evidence for a functional linkage between nsP2-T1325P and nsP4-V1911D substitutions. In MEF cells, BFV harboring both nsP2-T1325P and nsP4-V1911D substitutions was more attenuated than a virus with nsP2-T1325P substitution alone; furthermore, the combination of nsP2-T1325P and nsP4-V1911D resulted in increased sensitivity to type I IFN signaling. Such effects are likely to be related to their replicase efficiency showing BFV^T1325P^ > BFV^T1325P+V1911D^. In mosquito cells, however, the combination of mutations was better tolerated than the nsP2-T1325P substitution alone; this observation also supports the hypothesis that these mutations complement each other. On the other hand, it was noted that BFV replication organelles in mammalian and mosquito cells have different morphology. This may indicate that their formation and functioning requirements are somewhat different.

For the first time, a BFV infectious clone was constructed, which provides an essential tool for future investigations of this understudied alphavirus. This single plasmid reverse genetics system has great potential for genetic manipulation. Utilizing the infectious clone, we found an NLS within BFV nsP3 that is highly correlated to BFV pathogenicity. Furthermore, two amino acids in nsP2 and nsP4 were demonstrated as critical modulators of virus replication. Altogether, our BFV infectious clone and the virulence determinates revealed in this study will aid BFV vaccine design and therapeutic interventions.

## MATERIALS AND METHODS

### Cells

HEK293T (ATCC, CRL-3216), Vero (Sigma-Aldrich, USA), wt MEF, and IFNAR^-/-^ MEF cells (gift from Andreas Suhrbier, QIMR Berghofer Medical Research Institute, Australia) were maintained in Dulbecco’s modified Eagle’s medium (DMEM, Corning) supplemented with 10% fetal bovine serum (FBS). BHK-21 (ATCC CCL-10) cells were maintained in Glasgow’s minimal essential medium (GMEM, Gibco) supplemented with 10% FBS, 2% tryptose phosphate broth (TPB), and 200 mM HEPES, pH 7.2. U2OS (ATCC HTB-96) and COP5 (77) cells were propagated in Iscove’s modified Eagle’s medium (IMDM, Corning) containing L-glutamine and supplemented with 10% FBS. *Aedes albopictus* C6/36 cells were propagated in Leibovitz’s L-15 medium (Corning) supplemented with 10% heat-inactivated FBS (Gibco) and 10% TPB. *Aedes aegypti* AF319 cells (gift from Kevin Maringer, University of Surrey, UK) were maintained in Leibovitz’s L-15 medium supplemented with 20% heat-inactivated FBS (Gibco), 10% TPB, and 10% dilution of stock of non-essential amino acids (Sigma-Aldrich). Penicillin (100 U/mL) and streptomycin (0.1 mg/mL) were added to all growth media. Mammalian cells were cultured in a humidified incubator at 37°C with 5% CO_2_; mosquito cells were cultured at 28°C with no additional CO_2_.

### Design, assembly and verification of BFV infectious clone

The sequence of the complete BFV2193-FI genome was obtained from GenBank (GenBank: U73745.1) and was compared with the sequence of the structural region of the same isolate (GenBank: AF339488.1). A sequencing mistake was detected in the structural region of U73745.1 and corrected. The resulting sequence was obtained as five synthetic DNA fragments (GenScript, USA). Fragment A, which contained sequences corresponding to both ends of the BFV genome (residues 1–675 and 10,297–11,508), was cloned into a pUC57-Kan plasmid. The bacteriophage SP6 RNA polymerase promoter was placed upstream of the residue corresponding to the 5′ end of the BFV genome, and a recognition site for restriction endonuclease *Avr*II was placed immediately downstream of a 60-residues long poly(A) sequence. The full-length infectious clone was obtained in a single step by ligating fragments B, C, D, and E to the plasmid containing fragment A (Fig. 1A); its sequence was verified by Sanger sequencing.

Virus rescue was performed as described below. The rescued virus was propagated once in BHK-21 cells, and obtained P_1_ stock was used to confirm its sequence. RNA was extracted from 100 µL of P_1_ stock using Quick-RNA MiniPrep kit (Zymo Research), reverse transcribed using the First Strand cDNA Synthesis Kit (Thermo Fisher Scientific), and PCR-amplified. The obtained set of amplicons covering the coding regions of the BFV genome was analyzed using Sanger sequencing.

### NGS of the BFV2193-FI isolate

BFV2193-FI was kindly provided by Dr Linda Hueston (Westmead) (20). To obtain the complete sequence of BFV2193-FI, Vero cells were infected at an MOI of 1.0. The medium was collected at 24 h p.i., and RNA was extracted with TRIzol reagent (Invitrogen, USA) according to the manufacturer’s protocol. Five micrograms of purified RNA was sent to the Australian Genome Research Facility (AGRF) for NGS analysis using Illumina MiSeq sequencing with 150 bp paired-end reads. Obtained data sets were analyzed by *de novo* assembly using the Trinity Transcriptome Assembly (78). The 5′- and 3′-UTR of BFV2193-FI were amplified with the SMARTer RACE 5′/3′ Kit (Takara) according to the manufacturer’s protocol. Obtained fragments were cloned into provided vectors, and for each end of the genome, three clones were analyzed using Sanger sequencing. The obtained complete sequence of BFV2193-FI is shown in Data S1.

### Construction of pSP6-BFV-IC, its infectious clone, and replicase derivatives and plasmids for expression of EGFP-nsP3 fusion proteins

The infectious clone of BFV was constructed as shown in Fig. 1A. To correct the mistake originating from incorrect GenBank sequence (GenBank: U73745.1), the ^5793^GAC^5795^ codon in the BFV original infectious clone (designated pSP6-BFV^V1911D^) was replaced with ^5793^GTC^5795^ codon; the acquired construct was designated as pSP6-BFV-IC. To introduce the potential compensatory substitution, the ^4035^ACC^4037^ codon of pSP6-BFV^V1911D^ was replaced with ^4035^CCC^4037^, and the acquired construct was designated as pSP6-BFV^T1325P+V1911D^. The same substitution introduced into pSP6-BFV-IC resulted in pSP6-BFV^T1325P^. To obtain recombinant BFV expressing mCherry reporter, the sequence encoding mCherry was inserted between codons 1756 and 1757 of the BFV ns ORF; the resulting construct was designated as pSP6-BFV-P3mCh. All these substitutions were made using PCR-based mutagenesis and subcloning procedures.

A potential NLS sequence in nsP3 was identified using a web-based NLS prediction tool NLStradamus (79) (http://www.moseslab.csb.utoronto.ca/NLStradamus). To disrupt the NLS, the ^5013^AAA^5015^ codon in pSP6-BFV-IC was replaced with ^5013^GAT^5015^ resulting in pSP6-BFV^K1651D^. A similar substitution introduced into pSP6-BFV-P3mCh resulted in pSP6-BFV^K1651D^-P3mCh. The clone pCMV-SFV6-P3mCh was obtained from pCMV-SFV6 (80) by inserting the mCherry marker gene into the region encoding for HVD of nsP3 using a naturally occurring XhoI restriction site.

The previously described plasmid for the expression of BFV replicase in mammalian cells (34) was used to produce replicases, harboring the aforementioned point mutations, *via* subcloning procedures. Here, the described IC replicase was named as BFV-IC-Rep, the polymerase inactive variant, where the nsP4 active site has been disrupted by a GDD to GAA substitution, is referred to as BFV-GAA-Rep and the acquired mutant replicases as BFV^T1325P^-Rep, BFV^T1325P+V1911D^-Rep, BFV^V1911D^-Rep, and BFV^K1651D^-Rep.

pSP6-BFV-IC and pSP6-BFV^K1651D^ were used as templates for the PCR amplification of sequences encoding for the corresponding nsP3s. The latter were ligated with the sequence encoding for the EGFP reporter in a pcDNA4/TO vector (Invitrogen) in the way that the nsP3s were fused to the C-terminus of EGFP. Sequences for all plasmids were verified by Sanger sequencing.

### Trans-replication assay

The principle of the trans-replication assay is shown in Fig. S2. In this assay, HEK293T cells grown to ~90% confluency on 24-well plates were co-transfected with 0.5 µg of a given BFV replicase expressing plasmid and 0.5 µg of the previously described human RNA-polymerase-I-promoter-driven replication-competent BFV RNA template encoding plasmid HSPolI-FG-BFV (34) using Lipofectamine LTX and PLUS reagent (Invitrogen) according to the manufacturer’s protocol. The transfected cells were then incubated at 37°C for 18 h; an optimal incubation time was selected based on previously published data (58), followed by lysis. The firefly luciferase (FLuc) and *Gaussia* luciferase (GLuc) signals were detected using the Dual-Luciferase Reporter Assay System (Promega) and the GloMax 20/20 luminometer. For data analysis, the measured FLuc and GLuc values were normalized to those of the control transfection outputs.

### *In vitro* translation

The TnT SP6 Quick Transcription/Translation System kit (Promega) was used for *in vitro* translation. Reactions were performed in a final volume of 10 µL containing 0.25 µg of plasmid, 4 mM DTT, and 6 µCi of L-[^35^S]-methionine (Perkin-Elmer). The mixtures were incubated for 45 min at 30°C and then stopped with cycloheximide (1 mM final concentration) and 10 ng of RNase A and incubated for 5 min at 30°C. The mixture was then divided into two, where one part was subjected to denaturing conditions by boiling the samples in Laemmli sample buffer for 1 min, and the other was subjected to further incubation at 30°C for 30 min before being denatured. The proteins were resolved in 8% polyacrylamide/SDS gel and gels fixed and vacuum-dried. Protein bands were visualized using the Amersham Typhoon IP Biomolecular Imager (GE Healthcare).

### Pulse-labelling experiments

BHK-21 cells grown to ~90% confluency on six-well plates were infected with an MOI of 10. After 1 h, the cell monolayer was washed with PBS and supplemented with a complete growth medium. 0 h timepoint collection was initiated immediately, where the growth medium was replaced with cysteine and methionine-free DMEM (Sigma-Aldrich) for 30 min, which was then supplemented with radioactive cysteine and methionine labels (EasyTag EXPRESS^35^S Protein Labelling Mix, Perkin-Elmer) at a final activity of 100 µCi/mL. After 30 min, the cells were washed with PBS, lysed with Laemmli sample buffer, and boiled for 1 min. The protocol was repeated for timepoints 2, 4, 6, 8, 10, and 12 h p.i. The collected samples were then subjected to Benzonase nuclease (Merck) treatment, and the proteins were separated by SDS-PAGE using 10% polyacrylamide/SDS gel. The gel was subsequently fixed, dried, and then used for protein band visualization with the Amersham IP Biomolecular Imager (GE Healthcare).

### PAMP RNA production, RNA extraction and IFN-β detection by ELISA assay

To induce the production of non-classical viral PAMP RNAs, HEK293T cells grown to ~90% confluency on 24-well plates were transfected with 1 µg of the above-described BFV replicase expression plasmids using the Lipofectamine LTX and PLUS reagent (Invitrogen) according to the manufacturer’s protocol. Control cells were transfected with BFV-GAA-Rep expressing a polymerase-negative BFV replicase (34). Transfected cells were incubated at 37°C for 48 h and lysed for total RNA extraction using the TRIzol reagent (Invitrogen) following the manufacturer’s protocol. Five micrograms of total RNA was used for another round of transfections in confluent COP5 cells using the Lipofectamine 2000 reagent (Invitrogen). After 48 h of incubation, the supernatant was collected and assayed for IFN-β levels using the VeriKine Mouse IFN Beta ELISA Kit (PBL Assay Science).

### Virus rescue, ICA, and titration

Five micrograms of pSP6-BFV^V1911D^, pSP6-BFV-IC, pSP6-BFV^T1325P^, pSP6-BFV^T1325P+V1911D^, pSP6-BFV^K1651D^, pSP6-BFV-P3mC, and pSP6-BFV^K1651D^-P3mC was linearized using *Avr*II restriction enzyme, purified using DNA Clean & Concentrator—5 columns (Zymo Research), and eluted in 6 µL water. *In vitro* transcription was performed with the mMESSAGE mMACHINE SP6 kit (Ambion) in a reaction volume of 5 µL using 0.5 µg of the linearized template. The reaction mixture was used directly to transfect BHK-21 cells using the Lipofectamine 2000 reagent (Invitrogen). SFV6-P3mC was rescued as previously described for SFV6 (80). Transfected cells were monitored for CPE development, after which virus stocks were collected and clarified by centrifugation at 3,000 × *g* for 10 min and then stored as aliquots at −80°C.

For the ICA assay, BHK-21 cells were transfected *via* electroporation (850 V, 25 µF, two pulses in a 4 mm electrode gap cuvette) with 10 µg of RNA transcripts of pSP6-BFV^V1911D^, pSP6-BFV^T1325P+V1911D^, or pSP6-BFV-IC. 10-fold dilutions of transfected cells were seeded onto sub-confluent BHK-21 monolayers grown in six-well cell culture plates. The plates were incubated at 37°C for 3 h, after which the culture medium was replaced with GMEM containing 0.8% carboxymethylcellulose (CMC) and 2% FBS. Cells were incubated at 37°C for 3 to 5 days and stained with crystal violet. Formed plaques were counted, and the infectivity in PFU per µg RNA was calculated.

Virus titers were determined by plaque assay as previously described (26). Briefly, 100 µL of 10-fold serial dilutions of virus samples were added to a confluent monolayer of Vero cells in a 24-well plate. Infected cells were incubated for 1 h with occasional shaking, followed by replacement of inoculum with overlay consisting of DMEM with 0.8% CMC and 2% FBS. After 4 days of incubation, the cells were fixed and stained with crystal violet. Visible plaques were counted, and viral titers were calculated and expressed as PFU per one mL (or PFU per gram of tissue).

### Confocal microscopy and timelapse fluorescence microscopy

BHK-21, Vero, MEF, C6/36, and AF319 cells were grown on coverslips in six-well plates to a 50% confluency and infected with BFV-P3mCh, BFV^K1651D^-P3mCh, or SFV6-PmCh at an MOI of 1.0. At 24 h p.i., the cells were washed with PBS and fixed with 4% PFA in PBS. For the dsRNA observation experiment, BFV-P3mCh-infected Vero and C6/36 cells were stained using mouse monoclonal anti-dsRNA J2 antibody (Scicons) as the primary antibody and anti-mouse Alexa Fluor 488 conjugated antibody (Invitrogen) as the secondary antibody. For the transfection experiment, U2OS cells were grown on coverslips in six-well plates to a 50% confluency and transfected with plasmids encoding for EGFP-nsP3 or EGFP-nsP3^K1651D^ fusion proteins using Lipofectamine LTX and PLUS reagent (Invitrogen). 24 h post-transfection, the cells were washed and fixed as described above. Nuclei were counterstained with DAPI, and coverslips were mounted in the Slowfade Gold mounting medium (Invitrogen). Images were obtained with a Zeiss LSM710 confocal microscope and processed using the ZEN 2.6 Blue software (Zeiss). Images for infected Vero and C6/36 cells stained for dsRNA were obtained using a Nikon A1R + confocal microscope and processed with ImageJ software.

For the timelapse fluorescence microscopy, Vero-E6 cells grown to ~70% confluency on 24-well plates were infected with BFV-IC or BFV^K1651D^ at an MOI of 1.0. The timelapse live cell imaging was conducted with the EVOSTM 7000 Imaging System using the EVOSTM Onstage Incubator to keep high humidity conditions at 37°C, 5% CO2. Images were taken every 30 min over a period of 24.5 h. The timelapse animations were compiled using ZEN Blue 3.3 software (Zeiss).

### Multi-step growth curve experiments

Vero, wt MEF, and IFNAR^-/-^ MEF cells and C6/36 and AF319 mosquito cells grown on 24-well plates to a confluency of ~80–90% were infected with BFV2193-FI, BFV-IC, BFV^T1325P^, BFV^T1325P+V1911D^, or pSP6-BFV^K1651D^-P3mC at an MOI of 0.1. After a 1-h incubation period with occasional shaking, the inoculum was removed, the cells were washed with media and covered with complete growth medium, and the sample, corresponding to 0 h time point, was collected. At 3, 6, 9, 12, 24, and 48 h p.i., whole media was collected and replaced with fresh media. Virus titers in collected samples were determined by plaque assay.

### Mice and animal experiments

All animal experiments were approved by the Animal Ethics Committee of Griffith University (Gly/12/18/AEC). All procedures conform to the National Health and Medical Research Council guidelines (81). The humane endpoint was defined as a clinical disease score of 6 (moribund) or weight loss greater than 15%. Inbred 21-day-old C57BL/6 mice were obtained from the Animal Resource Centre, Perth, Australia. Animals in the experimental group (*n* = 5) were infected with BFV2193-FI, BFV-IC, or BFV^K1651D^ (10^5^ PFU in 50 µL of PBS) *via* subcutaneous injections in the thorax below the right forelimb; control groups received an injection of 50 µL of PBS. Every 24 h, mice were weighed, monitored for disease symptoms, and scored accordingly throughout disease progression and recovery. Disease scores were assessed based on disease signs as follows: 0, no disease signs; 1, ruffled fur; 2, very mild hind limb weakness, some lethargy; 3, mild hind limb weakness, some signs of delicate walking, lethargy; 4, moderate hind limb weakness and occasional dragging, limited gripping ability; 5, severe hind limb weakness and dragging, limited movement, moderate lethargy, weight loss; and 6, loss of hind limb functions.

To determine viral titers in serum and selected tissues, mice (*n* = 4) were infected with BFV2193-FI or BFV^K1651D^ (10^5^ PFU in 50 µL of PBS) *via* subcutaneous injections in the thorax below the right forelimb. At selected days post-infection, mice were euthanized by CO_2_ asphyxiation and perfused with PBS following blood sample collection. Tissue samples were harvested for hind limb ankles, and hind limb quadriceps were weighed and homogenized in PBS. Viral titers in obtained samples were determined by plaque assay.

### Analysis of IFN antagonist activity of BFV nsP3 proteins using luciferase reporter gene assays

Reporter assays for IFN induction and IFN signaling have been described previously (59, 82–85). Briefly, to measure the activity of RIG-I and MDA5 activation of IFNβ induction pathways and effects thereon of expression of BFV nsp3, HEK-293T cells were co-transfected in triplicate with 40 ng pRL-TK (which expresses *Renilla* luciferase (RLuc) under the control of the constitutively active thymidine kinase promoter), 250 ng pGL3-IFNβ (in which firefly luciferase (Fluc) is under the control of the IFN-β promoter) and 250 ng plasmids to express the indicated GFP-fused nsP3 proteins or the control proteins ORF6-StrepII [expressing the SARS-CoV-2 ORF6 protein, a potent inhibitor of RIG-I/MDA5-activated IFN induction, fused to StrepII tag (82)] or pEGFP-CVS-N [expressing the nucleoprotein (N protein) of rabies virus, which lacks intrinsic antagonist activity to the IFN-induction pathway (86)]. To stimulate pGL3-IFNβ (i.e., activate the IFN induction pathway), cells were co-transfected with 125 ng MDA5-CARD, RIG-I-CARD (expressing only the N terminal CARD regions of MDA5 or RIG-I, respectively, which are constitutively active versions of the MDA5 and RIG-I pattern recognition receptors that detect cytoplasmic viral RNA products) or, as a non-stimulatory control, pUC18 (empty vector, EV) (82). At 40 h post-transfection, cells were lysed in passive lysis buffer (Promega), and cleared lysate was analyzed using a dual luciferase assay as previously described (59, 82).

To measure IFN-α-dependent signaling and effects thereon of expression nsP3 proteins, HEK 293T cells were transfected in triplicate with pRL-TK (40 ng, as above), pISRE-luc [250 ng, which expresses FLuc under the control of an IFN-activated ISRE-containing promoter (83)], and plasmid to express the indicated GFP-fused nsP3 proteins, EV negative control (250 ng), or pEGFP-C1-CVS-P1 plasmid [positive control expressing P protein of CVS rabies virus, a potent inhibitor of type I IFN signaling (83)]. Cells were treated 8 h post-transfection with or without 1,000 U/mL recombinant human IFN-α (PBL Interferon Source) for 18 h before harvesting and analysis using a dual luciferase assay as above.

Relative luciferase activity (RLU) was determined as previously described (59, 82, 83, 87) by normalizing values for FLuc activity to those for RLuc for each sample.

### Statistical analysis

The growth kinetics of viruses, mouse weight gain, disease score, and cell infiltration were analyzed using two-way ANOVA with a Bonferroni *post hoc* test. Results obtained from luciferase reporter gene assays were analyzed using Student’s *t*-test. All statistical analyses were performed using GraphPad Prism software.

## SUPPLEMENTAL MATERIAL

Data S1 mbio.00993-24-s0001.pdf NGS sequencing data for BFV2193-FI.

Data S2 mbio.00993-24-s0002.pdf BFV non-structural polyprotein alignment for mutation position comparison.

Figure S1 mbio.00993-24-s0003.tiff MEF cells infected with BFV-P3mCh or BFVK1651D-P3mCh.

Figure S2 mbio.00993-24-s0004.tiff Components and principle of BFV trans-replicase system.

Supplemental legends mbio.00993-24-s0005.docx Legends for supplemental movies, figures, and data.

Movie S1 mbio.00993-24-s0006.mov BFV nsP3-mCherry localization during BFV-P3mCh infection.

Movie S2 mbio.00993-24-s0007.mov BFV nsP3-mCherry localization during BFVK1651D-P3mCh infection.

ASM does not own the copyrights to Supplemental Material that may be linked to, or accessed through, an article. The authors have granted ASM a non-exclusive, world-wide license to publish the Supplemental Material files. Please contact the corresponding author directly for reuse.

## References

[1] Marshall ID, Woodroofe GM, Hirsch S. 1982. Viruses recovered from mosquitoes and wildlife serum collected in the Murray Valley of South-eastern Australia, February 1974, during an epidemic of encephalitis. Aust J Exp Biol Med Sci 60 (Pt 5):457–470. doi:10.1038/icb.1982.51 6299258

[2] Jacups SP, Whelan PI, Currie BJ. 2008. Ross River virus and Barmah Forest virus infections: a review of history, ecology, and predictive models, with implications for tropical northern Australia. Vector Borne Zoonotic Dis 8:283–297. doi:10.1089/vbz.2007.0152 18279007

[3] Liu W, Kizu JR, Matley DR, Grant R, McCallum FJ, Moller CG, Carthew TL, Hang J, Gubala AJ, Aaskov JG. 2020. Circulation of 2 Barmah Forest virus lineages in military training areas, Australia. Emerg Infect Dis 26:3061–3065. doi:10.3201/eid2612.191747 33219791 PMC7706964

[4] Pelecanos AM, Ryan PA, Gatton ML. 2011. Spatial-temporal epidemiological analyses of two sympatric, co-endemic alphaviral diseases in Queensland, Australia. Vector Borne Zoonotic Dis 11:375–382. doi:10.1089/vbz.2009.0256 21466385

[5] Zaid A, Burt FJ, Liu X, Poo YS, Zandi K, Suhrbier A, Weaver SC, Texeira MM, Mahalingam S. 2021. Arthritogenic alphaviruses: epidemiological and clinical perspective on emerging arboviruses. Lancet Infect Dis 21:e123–e133. doi:10.1016/S1473-3099(20)30491-6 33160445

[6] Vale TG, Carter IW, McPhie KA, James GS, Cloonan MJ. 1986. Human arbovirus infections along the south coast of New South Wales. Aust J Exp Biol Med Sci 64:307–309. doi:10.1038/icb.1986.32 3767767

[7] Merianos A, Farland A, Patel M, Currie B, Whelan P, Dentith H, Smith D. 1992. A concurrent outbreak of Barmah Forest and Ross River virus disease in Nhulunbuy, Northern Territory. Commun Dis Intell 16:110–111.

[8] Lindsay M, Johansen C, Broom AK, Smith DW, Mackenzie JS. 1995. Emergence of Barmah Forest virus in Western Australia. Emerg Infect Dis 1:22–26. doi:10.3201/eid0101.950104 8903150 PMC2626822

[9] Russell RC. Arboviruses and their vectors in Australia: an update on the ecology and epidemiology of some mosquito-borne arboviruses

[10] Passmore J, O’Grady KA, Moran R, Wishart E. 2002. An outbreak of Barmah Forest virus disease in Victoria. Commun Dis Intell Q Rep 26:600–604. 12549534 10.33321/cdi.2002.26.66

[11] Quinn HE, Gatton ML, Hall G, Young M, Ryan PA. 2005. Analysis of Barmah Forest virus disease activity in Queensland, Australia, 1993-2003: identification of a large, isolated outbreak of disease. J Med Entomol 42:882–890. doi:10.1093/jmedent/42.5.882 16363173

[12] Caly L, Horwood PF, Vijaykrishna D, Lynch S, Greenhill AR, Pomat W, Rai G, Kisa D, Bande G, Druce J, Abdad MY. 2019. Divergent Barmah Forest virus from Papua New Guinea. Emerg Infect Dis 25:2266–2269. doi:10.3201/eid2512.191070 31742504 PMC6874237

[13] Herrero LJ, Taylor A, Wolf S, Mahalingam S. 2015. Arthropod-borne arthritides. Best Pract Res Clin Rheumatol 29:259–274. doi:10.1016/j.berh.2015.04.003 26362743

[14] Bettadapura J, Herrero LJ, Taylor A, Mahalingam S. 2013. Approaches to the treatment of disease induced by chikungunya virus. Indian J Med Res 138:762–765. 24434329 PMC3928707

[15] Tharmarajah K, Mahalingam S, Zaid A. 2017. Chikungunya: vaccines and therapeutics. F1000Res 6:2114. doi:10.12688/f1000research.12461.1 29259782 PMC5728195

[16] Flaxman JP, Smith DW, Mackenzie JS, Fraser JRE, Bass SP, Hueston L, Lindsay MDA, Cunningham AL. 1998. A comparison of the diseases caused by Ross River virus and Barmah Forest virus. Med J Aust 169:159–163. doi:10.5694/j.1326-5377.1998.tb116019.x 9734514

[17] Herrero LJ, Lidbury BA, Bettadapura J, Jian P, Herring BL, Hey-Cunningham WJ, Sheng KC, Zakhary A, Mahalingam S. 2014. Characterization of Barmah Forest virus pathogenesis in a mouse model. J Gen Virol 95:2146–2154. doi:10.1099/vir.0.064733-0 24934444

[18] Taylor A, Herrero LJ, Rudd PA, Mahalingam S. 2015. Mouse models of alphavirus-induced inflammatory disease. J Gen Virol 96:221–238. doi:10.1099/vir.0.071282-0 25351726

[19] Chen R, Mukhopadhyay S, Merits A, Bolling B, Nasar F, Coffey LL, Powers A, Weaver SC, Ictv Report Consortium. 2018. ICTV virus taxonomy profile: Togaviridae. J Gen Virol 99:761–762. doi:10.1099/jgv.0.001072 29745869 PMC12662122

[20] Lee E, Stocks C, Lobigs P, Hislop A, Straub J, Marshall I, Weir R, Dalgarno L. 1997. Nucleotide sequence of the Barmah Forest virus genome. Virology 227:509–514. doi:10.1006/viro.1996.8343 9018152

[21] Kostyuchenko VA, Jakana J, Liu X, Haddow AD, Aung M, Weaver SC, Chiu W, Lok SM. 2011. The structure of Barmah Forest virus as revealed by cryo-electron microscopy at a 6-angstrom resolution has detailed transmembrane protein architecture and interactions. J Virol 85:9327–9333. doi:10.1128/JVI.05015-11 21752915 PMC3165765

[22] Taylor A, Melton JV, Herrero LJ, Thaa B, Karo-Astover L, Gage PW, Nelson MA, Sheng KC, Lidbury BA, Ewart GD, McInerney GM, Merits A, Mahalingam S. 2016. Effects of an in-frame deletion of the 6K gene locus from the genome of Ross River virus. J Virol 90:4150–4159. doi:10.1128/JVI.03192-15 26865723 PMC4810561

[23] Rupp JC, Sokoloski KJ, Gebhart NN, Hardy RW. 2015. Alphavirus RNA synthesis and non-structural protein functions. J Gen Virol 96:2483–2500. doi:10.1099/jgv.0.000249 26219641 PMC4635493

[24] Garmashova N, Gorchakov R, Volkova E, Paessler S, Frolova E, Frolov I. 2007. The Old World and New World alphaviruses use different virus-specific proteins for induction of transcriptional shutoff. J Virol 81:2472–2484. doi:10.1128/JVI.02073-06 17108023 PMC1865960

[25] Cristea IM, Carroll J-W, Rout MP, Rice CM, Chait BT, MacDonald MR. 2006. Tracking and elucidating alphavirus-host protein interactions. J Biol Chem 281:30269–30278. doi:10.1074/jbc.M603980200 16895903

[26] Mutso M, Morro AM, Smedberg C, Kasvandik S, Aquilimeba M, Teppor M, Tarve L, Lulla A, Lulla V, Saul S, Thaa B, McInerney GM, Merits A, Varjak M. 2018. Mutation of CD2AP and SH3KBP1 binding motif in alphavirus nsP3 hypervariable domain results in attenuated virus. Viruses 10:226. doi:10.3390/v10050226 29702546 PMC5977219

[27] Ng WH, Liu X, Ling ZL, Santos CNO, Magalhães LS, Kueh AJ, Herold MJ, Taylor A, Freitas JR, Koit S, Wang S, Lloyd AR, Teixeira MM, Merits A, Almeida RP, King NJC, Mahalingam S. 2023. FHL1 promotes chikungunya and o'nyong-nyong virus infection and pathogenesis with implications for alphavirus vaccine design. Nat Commun 14:6605. doi:10.1038/s41467-023-42330-2 37884534 PMC10603155

[28] Panas MD, Ahola T, McInerney GM. 2014. The C-terminal repeat domains of nsP3 from the Old World alphaviruses bind directly to G3BP. J Virol 88:5888–5893. doi:10.1128/JVI.00439-14 24623412 PMC4019107

[29] Thaa B, Biasiotto R, Eng K, Neuvonen M, Götte B, Rheinemann L, Mutso M, Utt A, Varghese F, Balistreri G, Merits A, Ahola T, McInerney GM. 2015. Differential phosphatidylinositol-3-kinase-Akt-mTOR activation by Semliki Forest and chikungunya viruses is dependent on nsP3 and connected to replication complex internalization. J Virol 89:11420–11437. doi:10.1128/JVI.01579-15 26339054 PMC4645633

[30] Kim DY, Reynaud JM, Rasalouskaya A, Akhrymuk I, Mobley JA, Frolov I, Frolova EI. 2016. New World and Old World alphaviruses have evolved to exploit different components of stress granules, FXR and G3BP proteins, for assembly of viral replication complexes. PLoS Pathog 12:e1005810. doi:10.1371/journal.ppat.1005810 27509095 PMC4980055

[31] Schulte T, Liu L, Panas MD, Thaa B, Dickson N, Götte B, Achour A, McInerney GM. 2016. Combined structural, biochemical and cellular evidence demonstrates that both FGDF motifs in alphavirus nsP3 are required for efficient replication. Open Biol 6:160078. doi:10.1098/rsob.160078 27383630 PMC4967826

[32] Frolov I, Kim DY, Akhrymuk M, Mobley JA, Frolova EI. 2017. Hypervariable domain of eastern equine encephalitis virus nsP3 redundantly utilizes multiple cellular proteins for replication complex assembly. J Virol 91:e00371-17. doi:10.1128/JVI.00371-17 28468889 PMC5487569

[33] Frolova EI, Palchevska O, Dominguez F, Frolov I. 2023. Alphavirus-induced transcriptional and translational shutoffs play major roles in blocking the formation of stress granules. J Virol 97:e0097923. doi:10.1128/jvi.00979-23 37902397 PMC10688339

[34] Götte B, Utt A, Fragkoudis R, Merits A, McInerney GM. 2020. Sensitivity of alphaviruses to G3BP deletion correlates with efficiency of replicase polyprotein processing. J Virol 94:e01681-19. doi:10.1128/JVI.01681-19 31941782 PMC7081891

[35] Shin G, Yost SA, Miller MT, Elrod EJ, Grakoui A, Marcotrigiano J. 2012. Structural and functional insights into alphavirus polyprotein processing and pathogenesis. Proc Natl Acad Sci U S A 109:16534–16539. doi:10.1073/pnas.1210418109 23010928 PMC3478664

[36] McPherson RL, Abraham R, Sreekumar E, Ong S-E, Cheng S-J, Baxter VK, Kistemaker HAV, Filippov DV, Griffin DE, Leung AKL. 2017. ADP-ribosylhydrolase activity of chikungunya virus macrodomain is critical for virus replication and virulence. Proc Natl Acad Sci U S A 114:1666–1671. doi:10.1073/pnas.1621485114 28143925 PMC5321000

[37] Abraham R, Hauer D, McPherson RL, Utt A, Kirby IT, Cohen MS, Merits A, Leung AKL, Griffin DE. 2018. ADP-ribosyl-binding and hydrolase activities of the alphavirus nsP3 macrodomain are critical for initiation of virus replication. Proc Natl Acad Sci U S A 115:E10457–E10466. doi:10.1073/pnas.1812130115 30322911 PMC6217424

[38] Abraham R, McPherson RL, Dasovich M, Badiee M, Leung AKL, Griffin DE. 2020. Both ADP-ribosyl-binding and hydrolase activities of the alphavirus nsP3 macrodomain affect neurovirulence in mice. mBio 11:e03253-19. doi:10.1128/mBio.03253-19 32047134 PMC7018654

[39] Tuittila M, Hinkkanen AE. 2003. Amino acid mutations in the replicase protein nsP3 of Semliki Forest virus cumulatively affect neurovirulence. J Gen Virol 84:1525–1533. doi:10.1099/vir.0.18936-0 12771422

[40] Park E, Griffin DE. 2009. The nsP3 macro domain is important for Sindbis virus replication in neurons and neurovirulence in mice. Virology 388:305–314. doi:10.1016/j.virol.2009.03.031 19395054 PMC2683903

[41] Saul S, Ferguson M, Cordonin C, Fragkoudis R, Ool M, Tamberg N, Sherwood K, Fazakerley JK, Merits A. 2015. Differences in processing determinants of nonstructural polyprotein and in the sequence of nonstructural protein 3 affect neurovirulence of Semliki Forest virus. J Virol 89:11030–11045. doi:10.1128/JVI.01186-15 26311875 PMC4621116

[42] Saxton-Shaw KD, Ledermann JP, Borland EM, Stovall JL, Mossel EC, Singh AJ, Wilusz J, Powers AM. 2013. O'nyong nyong virus molecular determinants of unique vector specificity reside in non-structural protein 3. PLoS Negl Trop Dis 7:e1931. doi:10.1371/journal.pntd.0001931 23359824 PMC3554527

[43] Teppor M, Žusinaite E, Karo-Astover L, Omler A, Rausalu K, Lulla V, Lulla A, Merits A. 2021. Semliki Forest virus chimeras with functional replicase modules from related alphaviruses survive by adaptive mutations in functionally important hot spots. J Virol 95:e0097321. doi:10.1128/JVI.00973-21 34319778 PMC8475518

[44] Gorchakov R, Frolova E, Sawicki S, Atasheva S, Sawicki D, Frolov I. 2008. A new role for ns polyprotein cleavage in Sindbis virus replication. J Virol 82:6218–6231. doi:10.1128/JVI.02624-07 18417571 PMC2447110

[45] Lulla V, Karo-Astover L, Rausalu K, Merits A, Lulla A. 2013. Presentation overrides specificity: probing the plasticity of alphaviral proteolytic activity through mutational analysis. J Virol 87:10207–10220. doi:10.1128/JVI.01485-13 23864614 PMC3754006

[46] Bartholomeeusen K, Utt A, Coppens S, Rausalu K, Vereecken K, Ariën KK, Merits A. 2018. A chikungunya virus trans-replicase system reveals the importance of delayed nonstructural polyprotein processing for efficient replication complex formation in mosquito cells. J Virol 92:e00152-18. doi:10.1128/JVI.00152-18 29695432 PMC6026725

[47] Tamberg N, Lulla V, Fragkoudis R, Lulla A, Fazakerley JK, Merits A. 2007. Insertion of EGFP into the replicase gene of Semliki Forest virus results in a novel, genetically stable marker virus. J Gen Virol 88:1225–1230. doi:10.1099/vir.0.82436-0 17374766 PMC2274952

[48] Remenyi R, Roberts GC, Zothner C, Merits A, Harris M. 2017. SNAP-tagged chikungunya virus replicons improve visualisation of non-structural protein 3 by fluorescence microscopy. Sci Rep 7:5682. doi:10.1038/s41598-017-05820-0 28720784 PMC5515888

[49] Nowee G, Bakker JW, Geertsema C, Ros VID, Göertz GP, Fros JJ, Pijlman GP. 2021. A tale of 20 alphaviruses; inter-species diversity and conserved interactions between viral non-structural protein 3 and stress granule proteins. Front Cell Dev Biol 9:625711. doi:10.3389/fcell.2021.625711 33644063 PMC7905232

[50] Remenyi R, Gao Y, Hughes RE, Curd A, Zothner C, Peckham M, Merits A, Harris M. 2018. Persistent replication of a chikungunya virus replicon in human cells is associated with presence of stable cytoplasmic granules containing nonstructural protein 3. J Virol 92:e00477-18. doi:10.1128/JVI.00477-18 29875241 PMC6069192

[51] Kosugi S, Hasebe M, Matsumura N, Takashima H, Miyamoto-Sato E, Tomita M, Yanagawa H. 2009. Six classes of nuclear localization signals specific to different binding grooves of importin alpha. J Biol Chem 284:478–485. doi:10.1074/jbc.M807017200 19001369

[52] Fros JJ, Pijlman GP. 2016. Alphavirus infection: host cell shut-off and inhibition of antiviral responses. Viruses 8:166. doi:10.3390/v8060166 27294951 PMC4926186

[53] Aguilar PV, Weaver SC, Basler CF. 2007. Capsid protein of eastern equine encephalitis virus inhibits host cell gene expression. J Virol 81:3866–3876. doi:10.1128/JVI.02075-06 17267491 PMC1866141

[54] Breakwell L, Dosenovic P, Karlsson Hedestam GB, D’Amato M, Liljeström P, Fazakerley J, McInerney GM. 2007. Semliki Forest virus nonstructural protein 2 is involved in suppression of the type I interferon response. J Virol 81:8677–8684. doi:10.1128/JVI.02411-06 17553895 PMC1951358

[55] Atasheva S, Krendelchtchikova V, Liopo A, Frolova E, Frolov I. 2010. Interplay of acute and persistent infections caused by Venezuelan equine encephalitis virus encoding mutated capsid protein. J Virol 84:10004–10015. doi:10.1128/JVI.01151-10 20668087 PMC2937817

[56] Taylor A, Liu X, Zaid A, Goh LYH, Hobson-Peters J, Hall RA, Merits A, Mahalingam S. 2017. Mutation of the N-terminal region of chikungunya virus capsid protein: implications for vaccine design. mBio 8:e01970-16. doi:10.1128/mBio.01970-16 28223458 PMC5358915

[57] Liu X, Mutso M, Utt A, Lepland A, Herrero LJ, Taylor A, Bettadapura J, Rudd PA, Merits A, Mahalingam S. 2018. Decreased virulence of Ross River virus harboring a mutation in the first cleavage site of nonstructural polyprotein is caused by a novel mechanism leading to increased production of interferon-inducing RNAs. mBio 9:e00044-18. doi:10.1128/mBio.00044-18 30131356 PMC6106088

[58] Lello LS, Utt A, Bartholomeeusen K, Wang S, Rausalu K, Kendall C, Coppens S, Fragkoudis R, Tuplin A, Alphey L, Ariën KK, Merits A. 2020. Cross-utilisation of template RNAs by alphavirus replicases. PLoS Pathog 16:e1008825. doi:10.1371/journal.ppat.1008825 32886709 PMC7498090

[59] Audsley MD, Marsh GA, Lieu KG, Tachedjian M, Joubert DA, Wang LF, Jans DA, Moseley GW. 2016. The immune evasion function of J and Beilong virus V proteins is distinct from that of other paramyxoviruses, consistent with their inclusion in the proposed genus Jeilongvirus. J Gen Virol 97:581–592. doi:10.1099/jgv.0.000388 26703878

[60] Gao Y, Goonawardane N, Ward J, Tuplin A, Harris M. 2019. Multiple roles of the non-structural protein 3 (nsP3) alphavirus unique domain (AUD) during chikungunya virus genome replication and transcription. PLoS Pathog 15:e1007239. doi:10.1371/journal.ppat.1007239 30668592 PMC6358111

[61] Meshram CD, Shiliaev N, Frolova EI, Frolov I. 2020. Hypervariable domain of nsP3 of eastern equine encephalitis virus is a critical determinant of viral virulence. J Virol 94:e00617-20. doi:10.1128/JVI.00617-20 32581106 PMC7431797

[62] Meertens L, Hafirassou ML, Couderc T, Bonnet-Madin L, Kril V, Kümmerer BM, Labeau A, Brugier A, Simon-Loriere E, Burlaud-Gaillard J, et al.. 2019. FHL1 is a major host factor for chikungunya virus infection. Nature 574:259–263. doi:10.1038/s41586-019-1578-4 31554973

[63] Götte B, Panas MD, Hellström K, Liu L, Samreen B, Larsson O, Ahola T, McInerney GM. 2019. Separate domains of G3BP promote efficient clustering of alphavirus replication complexes and recruitment of the translation initiation machinery. PLoS Pathog 15:e1007842. doi:10.1371/journal.ppat.1007842 31199850 PMC6594655

[64] Mazzon M, Castro C, Thaa B, Liu L, Mutso M, Liu X, Mahalingam S, Griffin JL, Marsh M, McInerney GM. 2018. Alphavirus-induced hyperactivation of PI3K/AKT directs pro-viral metabolic changes. PLoS Pathog 14:e1006835. doi:10.1371/journal.ppat.1006835 29377936 PMC5805360

[65] Foo SS, Reading PC, Jaillon S, Mantovani A, Mahalingam S. 2015. Pentraxins and collectins: friend or foe during pathogen invasion? Trends Microbiol 23:799–811. doi:10.1016/j.tim.2015.09.006 26482345 PMC7127210

[66] Varjak M, Saul S, Arike L, Lulla A, Peil L, Merits A. 2013. Magnetic fractionation and proteomic dissection of cellular organelles occupied by the late replication complexes of Semliki Forest virus. J Virol 87:10295–10312. doi:10.1128/JVI.01105-13 23864636 PMC3754020

[67] Meshram CD, Lukash T, Phillips AT, Akhrymuk I, Frolova EI, Frolov I. 2019. Lack of nsP2-specific nuclear functions attenuates chikungunya virus replication both in vitro and in vivo. Virology 534:14–24. doi:10.1016/j.virol.2019.05.016 31163352 PMC7204530

[68] Dickson AM, Anderson JR, Barnhart MD, Sokoloski KJ, Oko L, Opyrchal M, Galanis E, Wilusz CJ, Morrison TE, Wilusz J. 2012. Dephosphorylation of HuR protein during alphavirus infection is associated with HuR relocalization to the cytoplasm. J Biol Chem 287:36229–36238. doi:10.1074/jbc.M112.371203 22915590 PMC3476290

[69] Tomar S, Hardy RW, Smith JL, Kuhn RJ. 2006. Catalytic core of alphavirus nonstructural protein nsP4 possesses terminal adenylyltransferase activity. J Virol 80:9962–9969. doi:10.1128/JVI.01067-06 17005674 PMC1617302

[70] Lello LS, Bartholomeeusen K, Wang S, Coppens S, Fragkoudis R, Alphey L, Ariën KK, Merits A, Utt A. 2021. nsP4 is a major determinant of alphavirus replicase activity and template selectivity. J Virol 95:e0035521. doi:10.1128/JVI.00355-21 34319783 PMC8475546

[71] Lello LS, Miilimäe A, Cherkashchenko L, Omler A, Skilton R, Ireland R, Ulaeto D, Merits A. 2023. Activity, template preference, and compatibility of components of RNA replicase of eastern equine encephalitis virus. J Virol 97:e0136822. doi:10.1128/jvi.01368-22 36533950 PMC9888243

[72] Coffey LL, Beeharry Y, Bordería AV, Blanc H, Vignuzzi M. 2011. Arbovirus high fidelity variant loses fitness in mosquitoes and mice. Proc Natl Acad Sci U S A 108:16038–16043. doi:10.1073/pnas.1111650108 21896755 PMC3179076

[73] Stapleford KA, Rozen-Gagnon K, Das PK, Saul S, Poirier EZ, Blanc H, Vidalain PO, Merits A, Vignuzzi M. 2015. Viral polymerase-helicase complexes regulate replication fidelity to overcome intracellular nucleotide depletion. J Virol 89:11233–11244. doi:10.1128/JVI.01553-15 26311883 PMC4645662

[74] Tan YB, Chmielewski D, Law MCY, Zhang K, He Y, Chen M, Jin J, Luo D. 2022. Molecular architecture of the chikungunya virus replication complex. Sci Adv 8:eadd2536. doi:10.1126/sciadv.add2536 36449616 PMC9710867

[75] Hwang Kim K, Rümenapf T, Strauss EG, Strauss JH. 2004. Regulation of Semliki Forest virus RNA replication: a model for the control of alphavirus pathogenesis in invertebrate hosts. Virology 323:153–163. doi:10.1016/j.virol.2004.03.009 15165827

[76] Hellström K, Kallio K, Utt A, Quirin T, Jokitalo E, Merits A, Ahola T. 2017. Partially uncleaved alphavirus replicase forms spherule structures in the presence and absence of RNA template. J Virol 91:e00787-17. doi:10.1128/JVI.00787-17 28701392 PMC5571266

[77] Tyndall C, La Mantia G, Thacker CM, Favaloro J, Kamen R. 1981. A region of the polyoma virus genome between the replication origin and late protein coding sequences is required in cis for both early gene expression and viral DNA replication. Nucleic Acids Res 9:6231–6250. doi:10.1093/nar/9.23.6231 6275353 PMC327600

[78] Shi M, Lin XD, Tian JH, Chen LJ, Chen X, Li CX, Qin XC, Li J, Cao JP, Eden JS, Buchmann J, Wang W, Xu J, Holmes EC, Zhang YZ. 2016. Redefining the invertebrate RNA virosphere. Nature 540:539–543. doi:10.1038/nature20167 27880757

[79] Nguyen Ba AN, Pogoutse A, Provart N, Moses AM. 2009. NLStradamus: a simple hidden Markov model for nuclear localization signal prediction. BMC Bioinformatics 10:202. doi:10.1186/1471-2105-10-202 19563654 PMC2711084

[80] Ferguson MC, Saul S, Fragkoudis R, Weisheit S, Cox J, Patabendige A, Sherwood K, Watson M, Merits A, Fazakerley JK. 2015. Ability of the encephalitic arbovirus Semliki Forest virus to cross the blood-brain barrier is determined by the charge of the E2 glycoprotein. J Virol 89:7536–7549. doi:10.1128/JVI.03645-14 25972559 PMC4505677

[81] Anonymous. 2013. Australian code for the care and use of animals for scientific purposes. Australian Government. Available from: www.nhmrc.gov.au/_files_nhmrc/publications/attachments/ea28_code_care_use_animals_131209.pdf

[82] Shemesh M, Aktepe TE, Deerain JM, McAuley JL, Audsley MD, David CT, Purcell DFJ, Urin V, Hartmann R, Moseley GW, Mackenzie JM, Schreiber G, Harari D. 2021. SARS-CoV-2 suppresses IFNβ production mediated by NSP1, 5, 6, 15, ORF6 and ORF7b but does not suppress the effects of added interferon. PLoS Pathog 17:e1009800. doi:10.1371/journal.ppat.1009800 34437657 PMC8389490

[83] Hossain MA, Larrous F, Rawlinson SM, Zhan J, Sethi A, Ibrahim Y, Aloi M, Lieu KG, Mok YF, Griffin MDW, Ito N, Ose T, Bourhy H, Moseley GW, Gooley PR. 2019. Structural elucidation of viral antagonism of innate immunity at the STAT1 interface. Cell Rep 29:1934–1945. doi:10.1016/j.celrep.2019.10.020 31722208

[84] Brice A, Whelan DR, Ito N, Shimizu K, Wiltzer-Bach L, Lo CY, Blondel D, Jans DA, Bell TDM, Moseley GW. 2016. Quantitative analysis of the microtubule interaction of rabies virus P3 protein: roles in immune evasion and pathogenesis. Sci Rep 6:33493. doi:10.1038/srep33493 27649849 PMC5030706

[85] Manokaran G, Audsley MD, Funakoda H, David CT, Garnham KA, Rawlinson SM, Deffrasnes C, Ito N, Moseley GW. 2022. Deactivation of the antiviral state by rabies virus through targeting and accumulation of persistently phosphorylated STAT1. PLoS Pathog 18:e1010533. doi:10.1371/journal.ppat.1010533 35576230 PMC9135343

[86] Masatani T, Ito N, Shimizu K, Ito Y, Nakagawa K, Sawaki Y, Koyama H, Sugiyama M. 2010. Rabies virus nucleoprotein functions to evade activation of the RIG-I-mediated antiviral response. J Virol 84:4002–4012. doi:10.1128/JVI.02220-09 20130065 PMC2849511

[87] Wiltzer L, Larrous F, Oksayan S, Ito N, Marsh GA, Wang LF, Blondel D, Bourhy H, Jans DA, Moseley GW. 2012. Conservation of a unique mechanism of immune evasion across the Lyssavirus genus. J Virol 86:10194–10199. doi:10.1128/JVI.01249-12 22740405 PMC3446585

