# Supplementary material for: Exploring Barmah Forest virus pathogenesis: molecular tools to investigate non-structural protein 3 nuclear localization and viral genomic determinants of replication
Source: mBio. 2024 Jul 2;15(8):e00993-24. doi: 10.1128/mbio.00993-24 (PMC11323547; doi:10.1128/mbio.00993-24)
Supplement: Data S1 — NGS sequencing data for BFV2193-FI. [file mbio.00993-24-s0001.pdf]

NGS sequencing data for BFV2193-FI

AATGGGCGCCTGGTAACAAGTTAGACATAAACCACAGCGATCCATTGTCTCCAGATCCAAACATGGCGAAACC
AGTTGTGAAGATCGACGTGGAACCTGAAAGCCATTTCGCTAAGCAGGTCCAGAGTTGCTTCCCGCAGTTTGAG
ATCGAAGCAGTGCAGACCACACCAAACGATCATGCACACGCGAGGGCGTTTTCGCACCTTGCTACGAAGCTCA
TAGAAATGGAGACAGCAAAAGATCAGATCATCCTCGATATCGGAAGTGCACCCGCGAGGAGACTGTATTCAG
AACACAAGTACCACTGTGTTTGCCCAATGAAGTGCACGGAAGATCCAGAGAGAATGCTAGGATATGCACGTA
AGTTGATCGCAGGCTCTGCGAAAGGGAAGGCAGAAAAGTTACGCGATCTCAGGGATGTCTTGGCTACGCCAG
ACATCGAGACGCAGTCGCTATGTCTCCACACAGACGCATCCTGCAGATACCGCGGTGATGTTGCCGTGTATCA
AGACGTGTATGCCATTGACGCACCTACCACGCTGTACCACCAAGCGTTAAAGGGCGTCAGGACCGCATATTGG
ATAGGCTTTGATACAACGCCGTTCATGTACGATGCACTAGCAGGAGCTTACCCGCTCTACTCCACAAACTGGG
CTGATGAGCAAGTGCTCGAGTCCAGAAACATTGGGCTATGTTCAGACAAAGTTTCTGAAGGGGGAAAGAAAG
GGAGATCAATCCTCAGGAAGAAGTTCTTGAAGCAGTCAGACAGAGTCATGTTCTCTGTCGGCTCGACGTTGTA
TACGGAAAGCCGTAAATTACTGCAAAGTTGGCACCTGCCATCCACATTCCATCTCAAAGGCAAATCTTCGTTCA
CGTGCCGCTGCGACACTATCGTCAGCTGCGAAGGGTATGTTCTGAAGAAAATTACAATGTGTCCTGGAGTGAC
AGGCAAACCGATAGGATATGCCGTCACCCATCACAAAGAAGGATTCGTAGTCGGAAAAGTCACAGATACCAT
TCGCGGCGAGAGAGTCTCCTTCGCCGTGTGTACTTATGTACCAACAACACTCTGCGACCAGATGACCGGGATC
CTAGCAACAGAAGTAACAGCCGATGATGCCCAGAAACTGCTGGTGGGTTTGAACCAGAGAATAGTAGTTAAT
GGTAGGACCCAGAGAAATACCAATACTATGAAGAACTACCTGCTACCACTGGTTGCACAAGCGCTAGCAAAAT
GGGCGAAGGAAGCAAAACAGGATATGGAAGATGAAAGACCCCTGAACGAACGCCAACGAACGCTAACGTGC
CTCTGCTGCTGGGCATTTAAGCGAAACAAACGCCACGCCATTTACAAGAGACCAGACACACAGAGTATAGTCA
AGGTCCCTTGCGAATTCACAAGCTTTCCTTTGGTCAGCCTGTGGTCCGCTGGGATGTCTATATCTCTTAGGCAG
AAGTTGAAGATGATGCTGCAGGCGAGGCAGCCCACACAAATAGCAGCAGTGACTGAGGAACTCATACAAGA
AGCAGCTGCAGTAGAGCAAGAGGCCGTGGATACGGCCAATGCCGAGCTGGACCACGCCGCATGGCCCTCCAT
TGTGGATACGACAGAGCGCCATGTTGAGGTCGAAGTGGAAGAACTCGACCAGCGTGCAGGGGAAGGGGTA
GTGGAAACACCTCGAAACTCTATCAAAGTTTCAACACAGATCGGGGACGCGTTAATCGGCAGTTACCTGATCC
TATCACCCCAAGCAGTCCTACGCAGCGAAAAATTAGCCTGCATACATGATCTTGCAGAGCAGGTTAAGTTGGT
CACACACTCTGGCCGTAGTGGTAGGTACGCCGTCGACAAATACGACGGAAGAGTACTAGTCCCTACAGGAGT
GGCTATAGACATTCAATCGTTCCAGGCTCTCAGTGAGAGCGCGACCCTTGTGTACAACGAACGCGAGTTCGTT
AACAGGAAGCTGTGGCACATAGCAGTATACGGGGCAGCACTCAATACTGATGAAGAAGGATACGAGAAGGT
CCCGGTAGAGAGAGCAGAATCAGATTATGTGTTTGATGTAGACCAAAAAATGTGCCTAAAAAAAGAGCAGGC
ATCAGGTTGGGTACTCTGTGGCGAACTAGTCAACCCCCCATTCCACGAATTCGCATATGAAGGGCTCCGCACG
AGACCGTCAGCACCCTACAAGGTTCATACAGTAGGTGTGTACGGAGTGCCAGGATCAGGCAAATCCGCAATA
ATCAAGAACACGGTCACCATGTCTGACCTAGTATTGAGTGGTAAGAAAGAGAACTGCTTAGAAATTATGAAC
GATGTACTTAAACACAGAGCTCTACGTATCACAGCGAAGACCGTAGACTCAGTGTTATTAAACGGCGTGAAAC
ACACGCCTAACATACTATACATCGACGAAGCGTTCTCATGCCATGCAGGGACTCTGTTGGCCACTATAGCCATA
GTCAGGCCCAAACAGAAAGTGGTACTGTGCGGAGACCCGAAACAATGCGGATTCTTCAATATGATGCAACTG
AAAGTTAATTACAATCATGACATCTGCTCAGAAGTCTTCCACAAAAGTATCTCTAGACGGTGCACCCAGGATAT
CACGGCCATCGTTTCCAAATTACATTACCAGGACCGAATGAGGACCACAAACCCCCGAAAAGGAGACATCATT
ATAGACACTACCGGCACTACCAAACCAGCCAAAACAGATCTGATTCTGACGTGCTTCAGGGGATGGGTGAAA
CAGTTGCAGCAAGACTACAGAGGTAACGAAGTAATGACGGCTGCAGCGTCCCAAGGACTGACGAGGGCCTC
CGTATATGCGGTTCGAACTAAAGTCAATGAGAACCCGCTATATGCACAGACCTCCGAGCACGTGAACGTGTTG
TTAACACGCACAGAAAACAAGCTAGTATGGAAGACCTTGTCAACAGATCCCTGGATTAAAACACTGACTAACC
CACCTAGAGGGCACTATACCGCCACCATAGCAGAATGGGAAGCGGAACACCAGGGTATAATGAAGGCCATAC
AAGGGTATGCACCGCCCGTGAACACCTTCATGAACAAAGTAAATGTGTGCTGGGCAAAGACACTTACGCCTGT
GCTGGAAACTGCGGGTATCTCCCTGTCAGCAGAAGACTGGTCTGAACTGCTGCCCCCGTTTGCCCAGGACGTG
GCGTACTCACCCGAGGTGGCATTAAACATCATATGCACGAAAATGTATGGGTTTGACTTAGACACTGGTCTTTT
TTCCAGGCCATCAGTGCCAATGACATACACCAAAGACCATTGGGATAACAGAGTTGGAGGGAAAATGTATGG

ATTCAGCCAACAAGCATACGATCAGCTGGCAAGACGACATCCGTACCTTCGAGGTAGAGAGAAATCAGGAAT
GCAGATCGTAGTCACTGAGATGCGTATCCAGCGCCCAAGATCGGATGCCAACATCATCCCGATCAACCGCAG
GCTCCCTCACTCACTCGTAGCCACACACGAGTATAGGCGAGCTGCACGGGCCGAGGAATTCTTCACCACGACA
CGAGGGTACACTATGCTGCTGGTCTCTGAGTATAACATGAACTTACCAAACAAGAAGATCACCTGGCTGGCTC
CGATAGGGACGCAGGGGGCCCATCACACCGCCAACCTAAACTTGGGGATACCACCTCTGCTGGGCAGTTTTG
ATGCGGTGGTTGTGAACATGCCGACTCCATTCCGGAACCATCACTACCAGCAATGTGAAGACCACGCGATGAA
ACTCCAGATGCTGGCAGGCGACGCACTGAGGCACATTAAACCTGGCGGATCATTGTGGGTCAAGGCATACGG
CTACGCAGACCGGCACAGCGAGCACGTGGTCTTGGCATTGGCTAGAAAGTTTAAAAGCTTCAGAGTCACACA
ACCCTCATGCGTGACTTCCAACACCGAGGTGTTTCTCCACTTCTCAATTTTTGACAATGGCAAACGCGCGATAG
CCCTGCATTCAGCTAATAGGAAGGCTAACAGTATCTTCCAAAACACCTTCTTACCGGCGGGCAGTGCACCGGC
GTACAGAGTCAAACGTGGAGACATTTCGAACGCCCCAGAGGATGCAGTGGTCAATGCAGCAAACCAACAGG
GAGTGAAGGGTGCTGGAGTTTGCGGTGCAATTTACCGTAAGTGGCCGGACGCTTTCGGTGATGTCGCTACTC
CAACCGGAACAGCAGTTTCGAAATCCGTCCAAGATAAATTGGTGATCCACGCTGTCGGCCCGAATTTCTCAAA
ATGTTCAGAAGAGGAAGGGGACAGAGACCTAGCATCTGCTTACAGAGCTGCAGCAGAAATAGTGATGGATA
AAAAAATTACAACAGTGGCCGTCCCCTTACTCTCCACCGGCATTTATGCCGGAGGAAAAAACAGAGTAGAACA
GTCACTCAACCATCTCTTCACGGCATTCGACAATACTGATGCAGATGTGACCATATATTGCATGGACAAAACAT
GGGAAAAGAAGATTAAGGAGGCAATCGATCACCGGACTTCGGTTGAGATGGTGCAGGATGACGTGCAGTTG
GAGGAGGAACTGGTACGAGTACACCCTTTGAGTAGTTTAGCAGGTAGGAAGGGTTACAGTACGGACAGCGG
CCGAGTGTTTTCCTACCTGGAAGGTACCAAATTCCATCAGACTGCGGTGGACATAGCCGAAATGCAAGTGCTG
TGGCCCGCCCTCAAAGAGTCTAATGAGCAAATAGTGGCATACACCTTAGGAGAATCAATGGACCAGATACGT
GGCAAGTGCCCGACAGAAGATACTGACGCCTCCACACCTCCACGGACTGTGCCGTGCCTCTGTCGATACGCCA
TGACACCAGAGAGAGTGTACCGACTTAAATGCACGAACACTACCCAATTTACGGTTTGCTCATCTTTTGAGTTG
CCAAAGTATCACATTCAGGGAGTGCAGAGAGTAAAATGTGAAAGAATCATCATCTTAGATCCCACTGTTCCAC
CAACTTACAAACGGCCATGCATCAGACGGTACCCCTCCACAATCTCTTGTAACTCCTCTGAGGACTCCAGGAGC
TTGTCTACTTTCTCTGTCAGCTCCGACTCCTCGATTGGTTCTCTGCCGGTCGGAGACACGAGACCCATTCCAGC
CCCGAGGACCATTTTCAGACCCGTCCCTGCCCCGAGAGCACCCGTGCTCAGAACCACACCGCCTCCTAAACCA
CCGCGCACATTCACCGTGCGTGCAGAAGTGCACCAAGCACCCCCTACACCTGTACCTCCACCCAGACCGAAGA
GGGCTGCAAAGTTGGCTCGTGAGATGCACCCCGGGTTCACCTTCGGGGACTTCGGAGAGCACGAGGTTGAG
GAGCTTACGGCCTCTCCCTTAACCTTCGGAGATTTTGCTGAAGGAGAGATCCAGGGGATGGGAGTGGAGTTT
GAATGACTAGGCAGAGCCGGCGGGTACATTTTTTCGTCAGACACGGGTCCAGGCCACCTACAGCAGAGATCC
GTTTTACAAAATTGCACGGCAGAATGTATCTACGAACCGGCAAAACTAGAAAAAATTCATGCACCAAAGTTGG
ATAAAACCAAGGAAGATATCTTAAGGAGCAAGTACCAAATGAAACCGTCTGAAGCAAACAAAAGCAGGTACC
AATCTAGAAAAGTAGAAAATATGAAAGCAGAGATCGTAGGTAGACTCTTGGACGGACTGGGGGAGTATCTG
GGCACCGAGCATCCAGTTGAATGCTACCGAATAACGTACCCGGTGCCTATATACTCAACTAGTGTCCTCAGAG
GTCTGTCTAGTGCCAAAACAGCTGTTAGAGCTTGCAATGCATTTTTGGAAGCTAATTTTCCATCAGTCACTTCAT
ATAAAATTACTGATGAATACGACGCATACCTAGATATGGTAGATGGATCAGAGAGCTGTCTGGACAGATCCTC
CTTTTCGCCGTCTAGATTGCGTAGCTTTCCAAAAACACACTCATACTTGGACCCACAGATCAACAGTGCGGTAC
CGTCACCATTCCAAAACACCTTACAAAATGTATTGGCAGCGGCCACCAAAAGAAACTGTAATGTCACACAGAT
GAGAGAACTACCAACATATGATTCTGCAGTGCTAAATGTAGAGGCCTTCAGGAAATATGCGTGCAAGCCAGA
CGTATGGGATGAGTACAGGGATAATCCGATTTGCATAACCACCGAAAATGTCACCACTTACGTCGCCAAGTTG
AAAGGACCGAAAGCTGCGGCCTTGTTTGCAAAAACACATAACCTGATACCACTACACCAAGTTCCTATGGACA
AATTCACGGTAGATATGAAGAGAGATGTCAAAGTCACGCCCGGAACCAAGCACACCGAAGAGAGACCAAAG
GTACAGGTGATTCAAGCGGCAGAGCCACTAGCCACTGCCTACCTCTGCGGAATTCACCGTGAATTGGTGCGCC
GTCTCAACAACGCGCTTTTCCCAAACATCCACACTTTGTTTGATATGTCCGCAGAGGATTTTGATGCAATCATA
GCGGAACATTTTAAGCACGGTGACCATGTGTTGGAAACGGATATAGCCTCTTTTGACAAAAGTCAAGATGATT
CCATGGCACTCACTGCGTTAATGATCCTTGAGGACCTGGGAGTAGACCAAAACCTAATGAATTTGATAGAGGC
TGCATTCGGGGAAATCGTGAGTACACACTTGCCCACAGGTACTAGATTCAAATTTGGAGCTATGATGAAGTCT
GGAATGTTTTTGACGCTGTTCGTCAATACAATTCTTAATGTGGTTATTGCGTGCCGAGTGTTGGAGGATCAATT

GGCGCAGTCGCCGTGGCCTGCTTTCATAGGAGATGACAACATAATCCATGGTATAATATCAGACAAATTGATG
GCAGATAGATGTGCCACCTGGATGAACATGGAGGTCAAGATACTGGACTCTATAGTTGGAATACGGCCACCT
TACTTCTGTGGAGGATTTATTGTATGTGACGATGTAACAGGTACAGCCTGCCGCGTCGCAGACCCACTGAAGA
GATTGTTCAAGCTAGGTAAGCCATTGCCACTTGACGATGGCCAAGATGAAGACAGAAGACGTGCATTACATG
ATGAAGTGAAAACCTGGTCGCGCGTAGGGCTGCGACACAGAGTGTGTGAAGCCATCGAAGACCGTTATGCCG
TCCACTCATCAGAACTAGTTTTATTGGCACTGACTACTCTGTCTAAGAACTTGAAGTCCTTCAGAAACATAAGA
GGGAAACCAATACATCTCTACGGTGGTCCTAAATAGTTGCCGTTAGACAACTAGTCTCGGCAACATGGATTTC
ATCCCCACCCAAACCTTCTATGGTAGACGATGGAGACCAGCACCAGTCCAGAGATACATACCCCAACCCCAAC
CACCAGCGCCTCCACGCCGTAGGAGAGGACCATCTCAACTCCAACAGCTTGTGGCTGCATTGGGCGCACTAGC
TCTACAACCCAAGCAGAAACAAAAAAGAGCACAGAAGAAGCCCAAGAAGACACCACCACCAAAACCAAAAAA
GACCCAGAAGCCTAAGAAACCAACCCAAAAGAAGAAGTCCAAACCCGGCAAACGTATGCGTAACTGCATGAA
GATCGAGAATGACTGCATCTTTCCGGTGATGCTCGATGGAAAGGTTAACGGCTACGCTTGCTTAGTGGGGGA
TAAAGTCATGAAACCAGCTCATGTGAAGGGCACGATCGACAATCCAGAACTAGCCAAATTGACATTCAAGAA
ATCTAGCAAGTATGATCTAGAATGTGCTCAAGTGCCGGTATGCATGAAATCAGACGCATCCAAGTTCACCCAT
GAGAAACCAGAAGGACATTACAACTGGCACCATGGGGCAGTGCAATTTAGCAATGGTAGGTTTACCATTCCG
ACGGGCTCTGGCAAACCTGGAGACAGTGGTAGGCCTATTTTTGACAATACCGGCAAGGTAGTAGCCATAGTG
CTGGGAGGTGCAAATGAAGGGGCCCGGACAGCCCTATCCGTGGTCACCTGGAATAAGGATATGGTGACCCG
CATAACACCTGAAGAATCAGTGGAGTGGTCGGCGGCCGCACTGAATATAACAGCACTATGTGTCCTCCAGAA
CTTATCGTTCCCGTGTGATGCACCACCATGTGCACCATGCTGTTACGAAAAAGACCCTGCAGGGACCCTAAGA
TTGCTGTCTGACCACTACTACCACCCCAAGTATTATGAATTACTTGACTCGACGATGCACTGCCCACAAGGAAG
GAGACCTAAGAGGTCTGTTGCGCATTTCGAAGCCTACAAGGCTACGAGACCGTATATAGGGTGGTGCGCAGA
TTGTGGACTGGCAGGATCATGCCCATCCCCTGTGAGCATCGAGCACGTCTGGAGTGATGCCGACGACGGCGT
ACTGAAGATCCAAGTGTCCATGCAGATCGGTATAGCTAAAAGCAATACTATTCACCACGCTAAGATACGTTAC
ATGGGTGCCAATGGAGTACAGGAGGCTGAACGCTCTACCCTAAGTGTATCCACAACAGCACCATGTGACATCT
TGGCGACCATGGGCCATTTCATCTTGGCCCGCTGCCGACCCGGCAGTCAAGTTGAAGTATCACTAAGCACCGA
TCCAAAGCTGCTATGCCGTACACCATTCTCCCACAAGCCCAGGTTTATTGGCAATGAAAAGTCCCCAGCACCCA
CCGGGCACAAGACCCGAATTCCCTGCAAAACTTACTCCCATCAGACAGACTTAACGAGAGAAGAGATTACAAT
GCATGTACCGCCGGATGTCCCCATCCAAGGGCTAGTGTCCAATACAGGTAAGTCGTACTCATTAGACCCAAAG
ACGAAGACCATCAAGTACAAATGCACTTGCGGCGAGACTGTAAAAGAAGGTACTGCTACGAACAAAATCACA
CTGTTCAATTGTGACACCGCCCCAAAGTGTATTACATATGCAGTGGATAACACAGTGTGGCAGTACAACTCCC
AATACGTGCCCAGGTCCGAAGTTACGGAGGTGAAAGGAAAGATCCATGTGCCTTTCCCTCTGACCGACAGCA
CGTGTGCAGTCAGCGTAGCACCTGAACCGCAAGTGACATACAGACTGGGGGAAGTGGAGTTCCACTTCCACC
CTATGTACCCCACCCTCTTCTCCATTAGGAGCCTCGGAAAGGATCCGAGCCACAGTCAAGAATGGATAGATAC
ACCCATGAGCAAGACAATCCAAGTTGGGGCAGAAGGCGTGGAGTATGTCTGGGGAAACAACAACCCGGTAC
GACTATGGGCACAGAAGAGCTCATCGAGCAGCGCGCATGGTAACCCTATTAGCATAGTCTCACATTACTATGA
CCTGTACCCTTACTGGACCATCACAGTACTAGCGAGTCTAGGCTTGCTAATAGTGATTAGTTCCGGTTTTTCAT
GCTTTTTGTGTTCAGTCGCTCGAACCAAATGCCTTACACCCTATCAATTAGCACCAGGCGCCCAATTACCCACAT
TTATAGCACTCCTTTGCTGCGCTAAGTCTGCACGCGCAGACACTTTAGATGATTTTTCCTACCTGTGGACCAAC
AACCAAGCCATGTTTTGGCTCCAACTGGCATCTCCGGTTGCAGCGTTCTTGTGCTTATCCTATTGCTGTAGAAA
TCTAGCATGCTGTATGAAGATTTTTTTAGGGATAAGCGGCCTGTGTGTAATTGCCACGCAGGCCTACGAGCAC
TCAACCACGATGCCGAATCAGGTGGGAATACCGTTTAAAGCCTTGATAGAGCGACCAGGTTACGCAGCGCTC
CCGCTATCTTTAGTAGTGATTAAGTCAGAATTAGTCCCCTCATTAGTTCAGGATTATATTACCTGCAACTACAAG
ACTGTGGTCCCGTCTCCGTACATTAAATGTTGCGGAGGCGCTGAGTGTTCACACAAAAATGAAGCGGACTATA
AGTGCTCGGTGTTCACAGGCGTGTACCCGTTTATGTGGGGAGGCGCCTACTGCTTCTGTGACACCGAAAACAG
TCAGATGAGTGAAGTATACGTAACCAGAGGAGAATCATGCGAGGCTGACCATGCCATCGCTTATCAGGTACA
CACAGCATCGCTTAAGGCACAAGTAATGATATCGATTGGAGAACTGAACCAAACCGTCGACGTGTTTGTCAAC
GGAGACAGTCCAGCCAGAATCCAACAATCAAAGTTCATACTTGGGCCGATATCCAGTGCCTGGTCTCCTTTTG
ATCACAAGGTGATCGTATACAGGGATGAGGTGTACAATGAAGACTACGCACCGTACGGATCCGGCCAAGCAG

GCAGGTTCGGAGACATCCAAAGTAGAACTGTTAACAGCACTGATGTCTATGCCAACACCAATTTGAAGCTTAA
AAGACCGGCTTCAGGCAATGTTCATGTACCATACACGCAAACCCCTTCGGGTTTCTCGTACTGGAAAAAAGAG
AAGGGAGTACCATTGAATCGAAACGCCCCTTTTGGCTGTATCATCAAAGTCAATCCAGTACGTGCTGAAAACT
GCGTATATGGCAACATACCGATCAGTATGGATATTGCGGACGCGCACTTCACAAGGATCGATGAATCCCCGTC
TGTGTCCTTGAAGGCGTGTGAAGTGCAGTCCTGCACTTATTCATCGGATTTTGGCGGAGTAGCGAGCATTTCC
TACACATCTAATAAGGTAGGTAAGTGTGCCATCCACAGCCACTCGAACTCCGCAACGATGAAGGATTCTGTGC
AGGATGTCCAGGAAAGCGGCGCCTTGTCGCTTTTCTTTGCGACTTCCTCTGTCGAGCCGAACTTCGTGGTCCAA
GTGTGTAACGCGCGGATCACTTGCCATGGTAAGTGTGAACCACCGAAAGACCACATCGTACCATACGCAGCC
AAACACAACGACGCCGAGTTTCCATCCATCTCTACTACAGCTTGGCAATGGTTGGCACACACCACCTCAGGGC
CACTCACCATACTTGTGGTAGCTATTATAGTCGTTGTTGTAGTATCCATTGTAGTATGTGCAAGACACTAGCAG
AATAGTATGTATGATAGTATATTTATAAGATTAGTTTGTTATGTAAGTATGTAGTAGTAAGTATAGTATTATAG
GAATTAATATAGATTAGACCAATACGTAAGTATGTAGTATTAAGTATAGTATTACAGGAATTAGTATAGTCATA
GTTAGTGTAGAAATAGTTCAAAGGGCCATATAACCCCTGAATAGTAACAAAAGAGAAAAACACAAAAATAGT
AGTTCAAAGGGCCATATTACCCCTGAATAGTAACAAAAATCAAAAACTAAATAAAAATTTAGAAAAACTTAAT
ATCAGATCCCAAAATTTTTAAATTGAACTGTAGCCGAACTCTACGGAGATGTAGGCATCCGAACTCTACGGAG
ATGTAGGATCAAATTCTGCCGAACCCCAGAACACCGGGGACGTAGGCGTCTAATTTGTTTTTTAATATTTTAC
