# Supplementary material for: Exploring Barmah Forest virus pathogenesis: molecular tools to investigate non-structural protein 3 nuclear localization and viral genomic determinants of replication
Source: mBio. 2024 Jul 2;15(8):e00993-24. doi: 10.1128/mbio.00993-24 (PMC11323547; doi:10.1128/mbio.00993-24)
Supplement: Data S2 — BFV non-structural polyprotein alignment for mutation position comparison. [file mbio.00993-24-s0002.pdf]

Description for position indications:
Nucleotide triplet corresponding for amino acid position 1325 in the non-structural polyprotein is highlighted in yellow.
Nucleotide triplet corresponding for amino acid position 1325 in the non-structural polyprotein is highlighted in orange.
The nucleotide sequence encoding for the predicted NLS in nsP3 is highlighted in magenta.

RID: ZUTWFH37016
Job Title:U73745.1 non-structural protein region
Program: BLASTN
Query: U73745.1 non-structural protein region ID: lcl|Query_724219(dna) Length: 7236
Database: nt Nucleotide collection (nt)

Sequences producing significant alignments:

```
                                                                          Scientific
 Common                     Max     Total Query   E    Per.    Acc.

Description                                                               Name
 Name             Taxid       Score   Score cover Value Ident  Len         Accession

Barmah Forest virus isolate KO376-1, complete genome                      Barmah
Fores... NA                11020      13221  13221 100%  0.0   99.64  11394
MN689031.1
Barmah Forest virus isolate SW28057, complete genome                      Barmah
Fores... NA                11020      12778  12778 100%  0.0   98.54  11467
MN689033.1
Barmah Forest virus isolate SW26969, complete genome                      Barmah
Fores... NA                11020      12778  12778 100%  0.0   98.54  11541
MN689032.1
Barmah Forest virus isolate SW31286, complete genome                      Barmah
Fores... NA                11020      12767  12767 100%  0.0   98.51  11523
MN689034.1
Barmah Forest virus isolate SW35221, complete genome                      Barmah
Fores... NA                11020      12761  12761 100%  0.0   98.49  11498
MN689035.1
Barmah Forest virus isolate K60652, complete genome                       Barmah
Fores... NA                11020      12694  12694 100%  0.0   98.33  11454
MN689026.1
Barmah Forest virus isolate K67171, complete genome                       Barmah
Fores... NA                11020      12689  12689 100%  0.0   98.31  11464
MN689028.1
Barmah Forest virus isolate K67289, complete genome                       Barmah
Fores... NA                11020      12683  12683 100%  0.0   98.30  11254
MN689029.1
Barmah Forest virus isolate K61404, complete genome                       Barmah
Fores... NA                11020      12667  12667 100%  0.0   98.26  11626
MN689027.1
Barmah Forest virus strain SW94093, complete genome                       Barmah
Fores... NA                11020      12661  12661 100%  0.0   98.24  11535
MW835350.1
Barmah Forest virus strain SW94245, complete genome                       Barmah
```

Fores... NA 11020 12661 12661 100% 0.0 98.24 11500
MW835349.1
Barmah Forest virus isolate SW94393, complete genome Barmah
Fores... NA 11020 12661 12661 100% 0.0 98.24 11493
MN689043.1
Barmah Forest virus isolate SW94096, complete genome Barmah
Fores... NA 11020 12661 12661 100% 0.0 98.24 11513
MN689042.1
Barmah Forest virus isolate SW67821, complete genome Barmah
Fores... NA 11020 12661 12661 100% 0.0 98.24 11494
MN689036.1
Barmah Forest virus isolate DC56192, complete genome Barmah
Fores... NA 11020 12661 12661 100% 0.0 98.24 11499
MN689023.1
Barmah Forest virus isolate SW93518, complete genome Barmah
Fores... NA 11020 12656 12656 100% 0.0 98.23 11456
MN689041.1
Barmah Forest virus isolate DC30314, complete genome Barmah
Fores... NA 11020 12656 12656 100% 0.0 98.23 11498
MN689021.1
Barmah Forest virus strain SW94401, complete genome Barmah
Fores... NA 11020 12650 12650 100% 0.0 98.22 11468
MW835348.1
Barmah Forest virus isolate DC57911, complete genome Barmah
Fores... NA 11020 12650 12650 100% 0.0 98.22 11495
MN689024.1
Barmah Forest virus isolate SW97836, complete genome Barmah
Fores... NA 11020 12645 12645 100% 0.0 98.20 11298
MN689045.1
Barmah Forest virus isolate SW77318, complete genome Barmah
Fores... NA 11020 12639 12639 100% 0.0 98.19 11492
MN689040.1
Barmah Forest virus isolate SW75325, complete genome Barmah
Fores... NA 11020 12639 12639 100% 0.0 98.19 11461
MN689038.1
Barmah Forest virus isolate SW68009, complete genome Barmah
Fores... NA 11020 12639 12639 100% 0.0 98.19 11525
MN689037.1
Barmah Forest virus isolate SW76326, complete genome Barmah
Fores... NA 11020 12628 12628 100% 0.0 98.16 11493
MN689039.1
Barmah Forest virus isolate DC45960, complete genome Barmah
Fores... NA 11020 12628 12628 100% 0.0 98.16 11487
MN689022.1
Barmah Forest virus isolate EGR27629, complete genome Barmah
Fores... NA 11020 12622 12622 100% 0.0 98.15 11499
MN689025.1
Barmah Forest virus isolate MIDITullyA.2017, complete genome Barmah
Fores... NA 11020 12617 12617 100% 0.0 98.13 11574
MN064696.1
Barmah Forest virus isolate MIDITully.2017, complete genome Barmah
Fores... NA 11020 12617 12617 100% 0.0 98.13 11489
MK697273.1
Barmah Forest virus isolate K80639, complete genome Barmah

```
Fores... NA             11020      12595  12595 100%  0.0   98.08  11490
MN689030.1
Barmah Forest virus isolate ARBO304, complete genome            Barmah
Fores... NA             11020      12589  12589 100%  0.0   98.07  11526
MW556196.1
Barmah Forest virus isolate SW105045, complete genome           Barmah
Fores... NA             11020      12584  12584 100%  0.0   98.05  11486
MN689046.1
Barmah Forest virus isolate MIDIWBTA.2018, complete genome      Barmah
Fores... NA             11020      12578  12578 100%  0.0   98.04  11563
MN064697.1
Barmah Forest virus isolate MIDIWB78.2018, complete genome      Barmah
Fores... NA             11020      12578  12578 100%  0.0   98.04  11503
MK697274.1
Barmah Forest virus isolate ARBO318, complete genome            Barmah
Fores... NA             11020      12578  12578 100%  0.0   98.04  11550
MW556197.1
Barmah Forest virus isolate SW105961, complete genome           Barmah
Fores... NA             11020      12578  12578 100%  0.0   98.04  11486
MN689047.1
Barmah Forest virus isolate SW94457, complete genome            Barmah
Fores... NA             11020      12491  12491 100%  0.0   97.89  11366
MN689044.1
Barmah Forest virus isolate PNG, complete genome                Barmah
Fores... NA             11020      12152  12152 100%  0.0   96.99  11480
MN115377.1

Alignments:

>Barmah Forest virus isolate KO376-1, complete genome
Sequence ID: MN689031.1 Length: 11394
Range 1: 55 to 7290

Score:13221 bits(7159), Expect:0.0,
Identities:7210/7236(99%),  Gaps:0/7236(0%), Strand: Plus/Plus

Query  1     ATGGCGAAACCAGTTGTGAAGATCGACGTGGAACCTGAAAGCCATTTCGCTAAGCAGGTC  60
Sbjct  55    ...........T................................................  114

Query  61    CAGAGTTGCTTCCCGCAGTTTGAGATCGAAGCAGTGCAGACCACACCAAACGATCATGCA  120
Sbjct  115   ............................................................  174

Query  121   CACGCGAGGGCGTTTTCGCACCTTGCTACGAAGCTCATAGAAATGGAGACAGCAAAAGAT  180
Sbjct  175   ............................................................  234

Query  181   CAGATCATCCTCGATATCGGAAGTGCACCCGCGAGGAGACTGTATTCAGAACACAAGTAC  240
Sbjct  235   ............................................................  294

Query  241   CACTGTGTTTGCCCAATGAAGTGCACGGAAGATCCAGAGAGAATGCTAGGATATGCACGT  300
Sbjct  295   ............................................................  354

Query  301   AAGTTGATCGCAGGCTCTGCGAAAGGGAAGGCAGAAAAGTTACGCGATCTCAGGGATGTC  360
Sbjct  355   ............................................................  414
```

```
Query  361   TTGGCTACGCCAGACATCGAGACGCAGTCGCTATGTCTCCACACAGACGCATCCTGCAGA  420
Sbjct  415   ............................................................  474

Query  421   TACCGCGGTGATGTTGCCGTGTATCAAGACGTGTATGCCATTGACGCACCTACCACGCTG  480
Sbjct  475   ............................................................  534

Query  481   TACCACCAAGCGTTAAAGGGCGTCAGGACCGCATATTGGATAGGCTTTGATACAACGCCG  540
Sbjct  535   ...........................................................A  594

Query  541   TTCATGTACGATGCACTAGCAGGAGCTTACCCGCTCTACTCCACAAACTGGGCTGATGAG  600
Sbjct  595   ............................................................  654

Query  601   CAAGTGCTCGAGTCCAGAAACATTGGGCTATGTTCAGACAAAGTTTCTGAAGGGGGAAAG  660
Sbjct  655   ............................................................  714

Query  661   AAAGGGAGATCAATCCTCAGGAAGAAGTTCTTGAAGCAGTCAGACAGAGTCATGTTCTCT  720
Sbjct  715   ............................................................  774

Query  721   GTCGGCTCGACGTTGTATACGGAAAGCCGTAAATTACTGCAAAGTTGGCACCTGCCATCC  780
Sbjct  775   ............................................................  834

Query  781   ACATTCCATCTCAAAGGCAAATCTTCGTTCACGTGCCGCTGCGACACTATCGTCAGCTGC  840
Sbjct  835   ............................................................  894

Query  841   GAAGGGTATGTTCTGAAGAAAATTACAATGTGTCCTGGAGTGACAGGCAAACCGATAGGA  900
Sbjct  895   ............................................................  954

Query  901   TATGCCGTCACCCATCACAAAGAAGGATTCGTAGTCGGAAAAGTCACAGATACCATTCGC  960
Sbjct  955   ............................................................  1014

Query  961   GGCGAGAGAGTCTCCTTCGCCGTGTGTACTTATGTACCAACAACACTCTGCGACCAGATG  1020
Sbjct  1015  ............................................................  1074

Query  1021  ACCGGGATCCTAGCAACAGAAGTAACAGCCGATGATGCCCAGAAACTGCTGGTGGGTTTG  1080
Sbjct  1075  ............................................................  1134

Query  1081  AACCAGAGAATAGTAGTTAATGGTAGGACCCAGAGAAATACCAATACTATGAAGAACTAC  1140
Sbjct  1135  ............................................................  1194

Query  1141  CTGCTACCACTGGTTGCACAAGCGCTAGCAAAATGGGCGAAGGAAGCAAAACAGGATATG  1200
Sbjct  1195  ......................................A.....................  1254

Query  1201  GAAGATGAAAGACCCCTGAACGAACGCCAACGAACGCTAACGTGCCTCTGCTGCTGGGCA  1260
Sbjct  1255  .........................................A..................  1314

Query  1261  TTTAAGCGAAACAAACGCCACGCCATTTACAAGAGACCAGACACACAGAGTATAGTCAAG  1320
Sbjct  1315  ............................................................  1374

Query  1321  GTCCCTTGCGAATTCACAAGCTTTCCTTTGGTCAGCCTGTGGTCCGCTGGGATGTCTATA  1380
Sbjct  1375  ..............................A.............................  1434

Query  1381  TCTCTTAGGCAGAAGTTGAAGATGATGCTGCAGGCGAGGCAGCCCACACAAATAGCAGCA  1440
Sbjct  1435  ............................................................  1494
```

```
Query  1441  GTGACTGAGGAACTCATACAAGAAGCAGCTGCAGTAGAGCAAGAGGCCGTGGATACGGCC  1500
Sbjct  1495  ............................................................  1554

Query  1501  AATGCCGAGCTGGACCACGCCGCATGGCCCTCCATTGTGGATACGACAGAGCGCCATGTT  1560
Sbjct  1555  ............................................................  1614

Query  1561  GAGGTCGAAGTGGAAGAACTCGACCAGCGTGCAGGGGAAGGGGTAGTGGAAACACCTCGA  1620
Sbjct  1615  ............................................................  1674

Query  1621  AACTCTATCAAAGTTTCAACACAGATCGGGGACGCGTTAATCGGCAGTTACCTGATCCTA  1680
Sbjct  1675  ............................................................  1734

Query  1681  TCACCCCAAGCAGTCCTACGCAGCGAAAAATTAGCCTGCATACATGATCTTGCAGAGCAG  1740
Sbjct  1735  ............................................................  1794

Query  1741  GTTAAGTTGGTCACACACTCTGGCCGTAGTGGTAGGTACGCCGTCGACAAATACNACGGA  1800
Sbjct  1795  ......................................................G.....  1854

Query  1801  AGAGTACTAGTCCCTACAGGAGTGGCTATAGACATTCAATCGTTCCAGGCTCTCAGTGAG  1860
Sbjct  1855  ............................................................  1914

Query  1861  AGCGCGACCCTTGTGTACAACGAACGCGAGTTCGTTAACAGGAAGCTGTGGCACATAGCA  1920
Sbjct  1915  ..........................A.................................  1974

Query  1921  GTATACGGGGCAGCACTCAATACTGATGAAGAAGGATACGAGAAGGTCCCGGTAGAGAGA  1980
Sbjct  1975  ............................................................  2034

Query  1981  GCAGAATCAGATTATGTGTTTGATGTAGACCAAAAAATGTGCCTaaaaaaaGAGCAGGCA  2040
Sbjct  2035  ............................................................  2094

Query  2041  TCAGGTTGGGTACTCTGTGGCGAACTAGTCAACCCCCCATTCCACGAATTCGCATATGAA  2100
Sbjct  2095  ............................................................  2154

Query  2101  GGGCTCCGCACGAGACCGTCAGCACCCTACAAGGTTCATACAGTAGGTGTGTACGGAGTG  2160
Sbjct  2155  ............................................................  2214

Query  2161  CCAGGATCAGGCAAATCCGCAATAATCAAGAACACGGTCACCATGTCTGACCTAGTATTG  2220
Sbjct  2215  ............................................................  2274

Query  2221  AGTGGTAAGAAAGAGAACTGCTTAGAAATTATGAACGATGTACTTAAACACAGAGCTCTA  2280
Sbjct  2275  ............................................................  2334

Query  2281  CGTATCACAGCGAAGACCGTAGACTCAGTGTTATTAAACGGCGTGAAACACACGCCTAAC  2340
Sbjct  2335  ............................................................  2394

Query  2341  ATACTATACATCGACGAAGCGTTCTCATGCCATGCAGGGACTCTGTTGGCCACTATAGCC  2400
Sbjct  2395  ............................................................  2454

Query  2401  ATAGTCAGGCCCAAACAGAAAGTGGTACTGTGCGGAGACCCGAAACAATGCGGATTCTTC  2460
Sbjct  2455  .....T......................................................  2514

Query  2461  AATATGATGCAACTGAAAGTTAATTACAATCATGACATCTGCTCAGAAGTCTTCCACAAA  2520
Sbjct  2515  ............................................................  2574
```

```
Query  2521  AGTATCTCTAGACGGTGCACCCAGGATATCACGGCCATCGTTTCCAAATTACATTACCAG  2580
Sbjct  2575  ............................................................  2634

Query  2581  GACCGAATGAGGACCACAAACCCCCGAAAAGGAGACATCATTATAGACACTACCGGCACT  2640
Sbjct  2635  ............................................................  2694

Query  2641  ACCAAACCAGCCAAAACAGATCTGATTCTGACGTGCTTCAGGGGATGGGTGAAACAGTTG  2700
Sbjct  2695  ............................................................  2754

Query  2701  CAGCAAGACTACAGAGGTAACGAAGTAATGACGGCTGCAGCGTCCCAAGGACTGACGAGG  2760
Sbjct  2755  ............................................................  2814

Query  2761  GCCTCCGTATATGCGGTTCGAACTAAAGTCAATGAGAACCCGCTATATGCACAGACCTCC  2820
Sbjct  2815  ............................................................  2874

Query  2821  GAGCACGTGAACGTGTTGTTAACACGCACAGAAAACAAGCTAGTATGGAAGACCTTGTCA  2880
Sbjct  2875  ............................................................  2934

Query  2881  ACAGATCCCTGGATTAAAACACTGACTAACCCACCTAGAGGGCACTATACCGCCACCATA  2940
Sbjct  2935  ............................................................  2994

Query  2941  GCAGAATGGGAAGCGGAACACCAGGGTATAATGAAGGCCATACAAGGGTATGCACCGCCC  3000
Sbjct  2995  ....................T.......................................  3054

Query  3001  GTGAACACCTTCATGAACAAAGTAAATGTGTGCTGGGCAAAGACACTTACGCCTGTGCTG  3060
Sbjct  3055  ............................................................  3114

Query  3061  GAAACTGCGGGTATCTCCCTGTCAGCAGAAGACTGGTCTGAACTGCTGCCCCCGTTTGCC  3120
Sbjct  3115  ............................................................  3174

Query  3121  CAGGACGTGGCGTACTCACCCGAGGTGGCATTAAACATCATATGCACGAAAATGTATGGG  3180
Sbjct  3175  ............................................................  3234

Query  3181  TTTGACTTAGACACTGGTCTTTTTTCCAGGCCATCAGTGCCAATGACATACACCAAAGAC  3240
Sbjct  3235  ............................................................  3294

Query  3241  CATTGGGATAACAGAGTTGGAGGGAAAATGTATGGATTCAGCCAACAAGCATACGATCAG  3300
Sbjct  3295  ............................................................  3354

Query  3301  CTGGCAAGACGACATCCGTACCTTCGAGGTAGAGAGAAATCAGGAATGCAGATCGTAGTC  3360
Sbjct  3355  ............................................................  3414

Query  3361  ACTGAAATGCGTATCCAGCGCCCAAGATCGGATGCCAACATCATCCCGATCAACCGCAGG  3420
Sbjct  3415  .....G......................................................  3474

Query  3421  CTCCCTCACTCACTCGTAGCCACACACGAGTATAGGCGAGCTGCACGGGCCGAGGAATTC  3480
Sbjct  3475  ............................................................  3534

Query  3481  TTCACCACGACACGAGGGTACACTATGCTGCTGGTCTCTGAGTATAACATGAACTTACCA  3540
Sbjct  3535  ............................................................  3594

Query  3541  AACAAGAAGATCACCTGGCTGGCTCCGATAGGGACGCAGGGGGCCCATCACACCGCCAAC  3600
Sbjct  3595  ...............................................C............  3654
```

```
Query  3601  CTAAACTTGGGGATACCACCTCTGCTGGGCAGTTTTGATGCGGTGGTTGTGAACATGCCG  3660
Sbjct  3655  ............................................................  3714

Query  3661  ACTCCATTCCGGAACCATCACTACCAGCAATGTGAAGACCACGCGATGAAACTCCAGATG  3720
Sbjct  3715  ............................................................  3774

Query  3721  CTGGCAGGCGACGCACTGAGGCACATTAAACCTGGCGGATCATTGTGGGTCAAGGCATAC  3780
Sbjct  3775  ............................................................  3834

Query  3781  GGCTACGCAGACCGGCACAGCGAGCACGTGGTCTTGGCATTGGCTAGAAAGTTTAAAAGC  3840
Sbjct  3835  ............................................................  3894

Query  3841  TTCAGAGTCACACAACCCTCATGCGTGACTTCCAACACCGAGGTGTTTCTCCACTTCTCA  3900
Sbjct  3895  ............................................................  3954

Query  3901  ATTTTTGACAATGGCAAACGCGCGATAGCCCTGCATTCAGCTAATAGGAAGGCTAACAGT  3960
Sbjct  3955  ............................................................  4014

Query  3961  ATCTTCCAAAACACCTTCTTACCGGCGGGCAGTGCACCGGCGTACAGAGTCAAACGTGGA  4020
Sbjct  4015  ............................................................  4074

Query  4021  GACATTTCGAACGCCCCAGAGGATGCAGTGGTCAATGCAGCAAACCAACAGGGAGTGAAG  4080
Sbjct  4075  ............................................................  4134

Query  4081  GGTGCTGGAGTTTGCGGTGCAATTTACCGTAAGTGGCCGGACGCTTTCGGTGATGTCGCT  4140
Sbjct  4135  ............................................................  4194

Query  4141  ACTCCAACCGGAACAGCAGTTTCGAAATCCGTCCAAGATAAATTGGTGATCCACGCTGTC  4200
Sbjct  4195  ............................................................  4254

Query  4201  GGCCCGAATTTCTCAAAATGTTCAGAAGAGGAAGGGGACAGAGACCTAGCATCTGCTTAC  4260
Sbjct  4255  ............................................................  4314

Query  4261  AGAGCTGCAGCAGAAATAGTGATGGATaaaaaaaTTACAACAGTGGCCGTCCCCTTACTC  4320
Sbjct  4315  ..........................A.................................  4374

Query  4321  TCCACCGGCATTTATGCCGGAGGAAAAAACAGAGTAGAACAGTCACTCAACCATCTCTTC  4380
Sbjct  4375  ............................................................  4434

Query  4381  ACGGCATTCGACAATACTGATGCAGATGTGACCATATATTGCATGGACAAAACATGGGAA  4440
Sbjct  4435  ............................................................  4494

Query  4441  AAGAAGATTAAGGAGGCAATCGATCACCGGACTTCGGTTGAGATGGTGCAGGATGACGTG  4500
Sbjct  4495  .............................A..............................  4554

Query  4501  CAGTTGGAGGAGGAACTGGTACGAGTACACCCTTTGAGTAGTTTAGCAGGTAGGAAGGGT  4560
Sbjct  4555  ............................................................  4614

Query  4561  TACAGTACGGACAGCGGCCGAGTGTTTTCCTACCTGGAAGGTACCAAATTCCATCAGACT  4620
Sbjct  4615  ............................................................  4674

Query  4621  GCGGTGGACATAGCCGAAATGCAAGTGCTGTGGCCCGCCCTCAAAGAGTCTAATGAGCAA  4680
Sbjct  4675  ............................................................  4734
```

```
Query  4681  ATAGTGGCATACACCTTAGGAGAATCAATGGACCAGATACGTGGCAAGTGCCCGACAGAA  4740
Sbjct  4735  ............................................................  4794

Query  4741  GATACTGACGCCTCCACACCTCCACGGACTGTGCCGTGCCTCTGTCGATACGCCATGACA  4800
Sbjct  4795  ............................................................  4854

Query  4801  CCAGAGAGAGTGTACCGACTTAAATGCACGAACACTACCCAATTTACGGTTTGCTCATCT  4860
Sbjct  4855  ............................................................  4914

Query  4861  TTTGAGTTGCCAAAGTATCACATTCAGGGAGTGCAGAGAGTAAAATGTGAAAGAATCATC  4920
Sbjct  4915  ...........................................................T  4974

Query  4921  ATCTTAGATCCCACTGTTCCACCAACTTACAAACGGCCATGCATCAGACGGTACCCCTCC  4980
Sbjct  4975  ............................................................  5034

Query  4981  ACAATCTCTTGTAACTCCTCTGAGGACTCCAGGAGCTTGTCTACTTTTTCTGTCAGCTCC  5040
Sbjct  5035  ...............................................C............  5094

Query  5041  GACTCCTCGATTGGTTCTCTGCCGGTCGGAGACACGAGACCCATTCCAGCCCCGAGGACC  5100
Sbjct  5095  ............................................................  5154

Query  5101  ATTTTCAGACCCGTCCCTGCCCCGAGAGCACCCGTGCTCAGAACCACACCGCCTCCTAAA  5160
Sbjct  5155  ............................................................  5214

Query  5161  CCACCGCGCACATTCACCGTGCGTGCAGAAGTGCACCAAGCACCCCCTACACCTGTACCT  5220
Sbjct  5215  ............................................................  5274

Query  5221  CCACCCAGACCGAAGAGGGCTGCAAAGTTGGCTCGTGAGATGCACCCCGGGTTCACCTTC  5280
Sbjct  5275  ............................................................  5334

Query  5281  GGGGACTTCGGAGAGCACGAGGTTGAGGAGCTTACGGCCTCTCCCTTAACCTTCGGAGAT  5340
Sbjct  5335  ............................................................  5394

Query  5341  TTTGCTGAAGGAGAGATCCAGGGGATGGGAGTGGAGTTTGAATGACTAGGCAGAGCCGGC  5400
Sbjct  5395  ........G...................................................  5454

Query  5401  GGGTACATTTTTTCGTCAGACACGGGTCCAGGCCACCTACAGCAGAGATCCGTTTTACAA  5460
Sbjct  5455  ............................................................  5514

Query  5461  AATTGCACGGCAGAATGTATCTACGAACCGGCAAAACTAGAAAAAATTCATGCACCAAAG  5520
Sbjct  5515  ............................................................  5574

Query  5521  TTGGATAAAACCAAGGAAGATATCTTAAGGAGCAAGTACCAAATGAAACCGTCTGAAGCA  5580
Sbjct  5575  ............................................................  5634

Query  5581  AACAAAAGCAGGTACCAATCTAGAAAAGTAGAAAATATGAAAGCAGAGATCGTAGGTAGA  5640
Sbjct  5635  ..T..................................................G......  5694

Query  5641  CTCTTGGACGGACTGGGGGAGTATCTGGGCACCGAGCATCCAGTTGAATGCTACCGAATA  5700
Sbjct  5695  ............................................................  5754

Query  5701  ACGTACCCGGTGCCTATATACTCAACTAGTGACCTCAGAGGTCTGTCTAGTGCCAAAACA  5760
Sbjct  5755  ...............................T............................  5814
```

```
Query  5761  GCTGTTAGAGCTTGCAATGCATTTTTGGAAGCTAATTTTCCATCAGTCACTTCATATAAA  5820
Sbjct  5815  ............................................................  5874

Query  5821  ATTACTGATGAATACGACGCATACCTAGATATGGTAGATGGATCAGAGAGCTGTCTGGAC  5880
Sbjct  5875  .....C......................................................  5934

Query  5881  AGATCCTCCTTTTCGCCGTCTAGATTGCGTAGCTTTCCAAAAACACACTCATACTTGGAC  5940
Sbjct  5935  ............................................................  5994

Query  5941  CCACAGATCAACAGTGCGGTACCGTCACCATTCCAAAACACCTTACAAAATGTATTGGCA  6000
Sbjct  5995  ............................................................  6054

Query  6001  GCGGCCACCAAAAGAAACTGTAATGTCACACAGATGAGAGAACTACCAACATATGATTCT  6060
Sbjct  6055  ............................................................  6114

Query  6061  GCAGTGCTAAATGTAGAGGCCTTCAGGAAATATGCGTGCAAGCCAGACGTATGGGATGAG  6120
Sbjct  6115  ............................................................  6174

Query  6121  TACAGGGATAATCCGATTTGCATAACCACCGAAAATGTCACCACTTACGTCGCCAAGTTG  6180
Sbjct  6175  ............................................................  6234

Query  6181  AAAGGACCGAAAGCTGCGGCCTTGTTTGCAAAAACACATAACCTGATACCACTACACCAA  6240
Sbjct  6235  ...................................................T........  6294

Query  6241  GTTCCTATGGACAAATTCACGGTAGATATGAAGAGAGATGTCAAAGTCACGCCCGGAACC  6300
Sbjct  6295  ............................................................  6354

Query  6301  AAGCACACCGAAGAGAGACCAAAGGTACAGGTGATTCAAGCGGCAGAGCCACTAGCCACT  6360
Sbjct  6355  ............................................................  6414

Query  6361  GCCTACCTCTGCGGAATTCACCGTGAATTGGTGCGCCGTCTCAACAACGCGCTTTTCCCA  6420
Sbjct  6415  ........................................................T...  6474

Query  6421  AACATCCACACTTTGTTTGATATGTCCGCAGAGGATTTCGATGCAATCATAGCGGAACAT  6480
Sbjct  6475  ......................................T.....................  6534

Query  6481  TTTAAGCACGGTGACCATGTGTTGGAAACGGATATAGCCTCTTTTGACAAAAGTCAAGAT  6540
Sbjct  6535  ............................................................  6594

Query  6541  GATTCCATGGCACTCACTGCGTTAATGATCCTTGAGGACCTGGGAGTAGACCAAAACCTA  6600
Sbjct  6595  .................C..........................................  6654

Query  6601  ATGAATTTGATAGAGGCTGCATTCGGGGAAATCGTGAGTACACACTTGCCCACAGGTACT  6660
Sbjct  6655  ............................................................  6714

Query  6661  AGATTCAAATTTGGAGCTATGATGAAGTCTGGAATGTTTTTGACGCTGTTCGTCAATACA  6720
Sbjct  6715  ............................................................  6774

Query  6721  ATTCTTAATGTGGTTATTGCGTGCCGAGTGTTGGAGGATCAATTGGCGCAGTCGCCGTGG  6780
Sbjct  6775  ...........................................................C  6834

Query  6781  CCTGCTTTCATAGGAGATGACAACATAATCCATGGTATAATATCAGACAAATTGATGGCA  6840
Sbjct  6835  G...........................................................  6894
```

```
Query  6841  GATAGATGTGCCACCTGGATGAACATGGAGGTCAAGATACTGGACTCTATAGTTGGAATA  6900
Sbjct  6895  ............................................................  6954

Query  6901  CGGCCACCTTACTTCTGTGGAGGATTTATTGTATGTGACGATGTAACAGGTACAGCCTGC  6960
Sbjct  6955  ............................................................  7014

Query  6961  CGCGTCGCAGACCCACTGAAGAGATTGTTCAAGCTAGGTAAGCCATTGCCACTTGACGAT  7020
Sbjct  7015  ............................................................  7074

Query  7021  GGCCAAGATGAAGACAGAAGACGTGCATTACATGATGAAGTGAAAACCTGGTCGCGCGTA  7080
Sbjct  7075  ............................................................  7134

Query  7081  GGGCTGCGACACAGAGTGTGTGAAGCCATCGAAGACCGTTATGCCGTCCACTCATCAGAA  7140
Sbjct  7135  ............................................................  7194

Query  7141  CTAGTTTTATTGGCACTGACTACTCTGTCTAAGAACTTGAAGTCCTTCAGAAACATAAGA  7200
Sbjct  7195  ............................................................  7254

Query  7201  GGGAAACCAATACATCTCTACGGTGGTCCTAAATAG  7236
Sbjct  7255  ....................................  7290



>Barmah Forest virus isolate SW28057, complete genome
Sequence ID: MN689033.1 Length: 11467
Range 1: 54 to 7289

Score:12778 bits(6919), Expect:0.0,
Identities:7130/7236(99%),  Gaps:0/7236(0%), Strand: Plus/Plus

Query  1     ATGGCGAAACCAGTTGTGAAGATCGACGTGGAACCTGAAAGCCATTTCGCTAAGCAGGTC  60
Sbjct  54    ...............................................T............  113

Query  61    CAGAGTTGCTTCCCGCAGTTTGAGATCGAAGCAGTGCAGACCACACCAAACGATCATGCA  120
Sbjct  114   .........................................G..................  173

Query  121   CACGCGAGGGCGTTTTCGCACCTTGCTACGAAGCTCATAGAAATGGAGACAGCAAAAGAT  180
Sbjct  174   ............................................................  233

Query  181   CAGATCATCCTCGATATCGGAAGTGCACCCGCGAGGAGACTGTATTCAGAACACAAGTAC  240
Sbjct  234   ............................................................  293

Query  241   CACTGTGTTTGCCCAATGAAGTGCACGGAAGATCCAGAGAGAATGCTAGGATATGCACGT  300
Sbjct  294   ............................................................  353

Query  301   AAGTTGATCGCAGGCTCTGCGAAAGGGAAGGCAGAAAAGTTACGCGATCTCAGGGATGTC  360
Sbjct  354   ............................................................  413

Query  361   TTGGCTACGCCAGACATCGAGACGCAGTCGCTATGTCTCCACACAGACGCATCCTGCAGA  420
Sbjct  414   ............................................................  473

Query  421   TACCGCGGTGATGTTGCCGTGTATCAAGACGTGTATGCCATTGACGCACCTACCACGCTG  480
Sbjct  474   ..................................................C.........  533
```

```
Query  481   TACCACCAAGCGTTAAAGGGCGTCAGGACCGCATATTGGATAGGCTTTGATACAACGCCG  540
Sbjct  534   ...........................................................A  593

Query  541   TTCATGTACGATGCACTAGCAGGAGCTTACCCGCTCTACTCCACAAACTGGGCTGATGAG  600
Sbjct  594   .........A...........................................C......  653

Query  601   CAAGTGCTCGAGTCCAGAAACATTGGGCTATGTTCAGACAAAGTTTCTGAAGGGGGAAAG  660
Sbjct  654   ............................................................  713

Query  661   AAAGGGAGATCAATCCTCAGGAAGAAGTTCTTGAAGCAGTCAGACAGAGTCATGTTCTCT  720
Sbjct  714   ............................................................  773

Query  721   GTCGGCTCGACGTTGTATACGGAAAGCCGTAAATTACTGCAAAGTTGGCACCTGCCATCC  780
Sbjct  774   ............................................C...............  833

Query  781   ACATTCCATCTCAAAGGCAAATCTTCGTTCACGTGCCGCTGCGACACTATCGTCAGCTGC  840
Sbjct  834   ................................A..............C............  893

Query  841   GAAGGGTATGTTCTGAAGAAAATTACAATGTGTCCTGGAGTGACAGGCAAACCGATAGGA  900
Sbjct  894   ................................C..C........................  953

Query  901   TATGCCGTCACCCATCACAAAGAAGGATTCGTAGTCGGAAAAGTCACAGATACCATTCGC  960
Sbjct  954   ....................G.............................C.........  1013

Query  961   GGCGAGAGAGTCTCCTTCGCCGTGTGTACTTATGTACCAACAACACTCTGCGACCAGATG  1020
Sbjct  1014  ............................................................  1073

Query  1021  ACCGGGATCCTAGCAACAGAAGTAACAGCCGATGATGCCCAGAAACTGCTGGTGGGTTTG  1080
Sbjct  1074  ........T...................................................  1133

Query  1081  AACCAGAGAATAGTAGTTAATGGTAGGACCCAGAGAAATACCAATACTATGAAGAACTAC  1140
Sbjct  1134  ............................................C...............  1193

Query  1141  CTGCTACCACTGGTTGCACAAGCGCTAGCAAAATGGGCGAAGGAAGCAAAACAGGATATG  1200
Sbjct  1194  ......................................A.....................  1253

Query  1201  GAAGATGAAAGACCCCTGAACGAACGCCAACGAACGCTAACGTGCCTCTGCTGCTGGGCA  1260
Sbjct  1254  ............................................................  1313

Query  1261  TTTAAGCGAAACAAACGCCACGCCATTTACAAGAGACCAGACACACAGAGTATAGTCAAG  1320
Sbjct  1314  ............................................................  1373

Query  1321  GTCCCTTGCGAATTCACAAGCTTTCCTTTGGTCAGCCTGTGGTCCGCTGGGATGTCTATA  1380
Sbjct  1374  ............................................................  1433

Query  1381  TCTCTTAGGCAGAAGTTGAAGATGATGCTGCAGGCGAGGCAGCCCACACAAATAGCAGCA  1440
Sbjct  1434  ............................................................  1493

Query  1441  GTGACTGAGGAACTCATACAAGAAGCAGCTGCAGTAGAGCAAGAGGCCGTGGATACGGCC  1500
Sbjct  1494  ..................................C.........................  1553

Query  1501  AATGCCGAGCTGGACCACGCCGCATGGCCCTCCATTGTGGATACGACAGAGCGCCATGTT  1560
Sbjct  1554  ...............................................G............  1613
```

```
Query  1561  GAGGTCGAAGTGGAAGAACTCGACCAGCGTGCAGGGGAAGGGGTAGTGGAAACACCTCGA  1620
Sbjct  1614  ............................................................  1673

Query  1621  AACTCTATCAAAGTTTCAACACAGATCGGGGACGCGTTAATCGGCAGTTACCTGATCCTA  1680
Sbjct  1674  ........T...................................................  1733

Query  1681  TCACCCCAAGCAGTCCTACGCAGCGAAAAATTAGCCTGCATACATGATCTTGCAGAGCAG  1740
Sbjct  1734  ............................................................  1793

Query  1741  GTTAAGTTGGTCACACACTCTGGCCGTAGTGGTAGGTACGCCGTCGACAAATACNACGGA  1800
Sbjct  1794  ..C.................C..T..............................G.....  1853

Query  1801  AGAGTACTAGTCCCTACAGGAGTGGCTATAGACATTCAATCGTTCCAGGCTCTCAGTGAG  1860
Sbjct  1854  ............................................................  1913

Query  1861  AGCGCGACCCTTGTGTACAACGAACGCGAGTTCGTTAACAGGAAGCTGTGGCACATAGCA  1920
Sbjct  1914  ..T..........................................T..............  1973

Query  1921  GTATACGGGGCAGCACTCAATACTGATGAAGAAGGATACGAGAAGGTCCCGGTAGAGAGA  1980
Sbjct  1974  ............................................................  2033

Query  1981  GCAGAATCAGATTATGTGTTTGATGTAGACCAAAAAATGTGCCTaaaaaaaGAGCAGGCA  2040
Sbjct  2034  .................A............A.............C...............  2093

Query  2041  TCAGGTTGGGTACTCTGTGGCGAACTAGTCAACCCCCCATTCCACGAATTCGCATATGAA  2100
Sbjct  2094  .....A......................................................  2153

Query  2101  GGGCTCCGCACGAGACCGTCAGCACCCTACAAGGTTCATACAGTAGGTGTGTACGGAGTG  2160
Sbjct  2154  ........T...................................................  2213

Query  2161  CCAGGATCAGGCAAATCCGCAATAATCAAGAACACGGTCACCATGTCTGACCTAGTATTG  2220
Sbjct  2214  ............................................................  2273

Query  2221  AGTGGTAAGAAAGAGAACTGCTTAGAAATTATGAACGATGTACTTAAACACAGAGCTCTA  2280
Sbjct  2274  ............................................................  2333

Query  2281  CGTATCACAGCGAAGACCGTAGACTCAGTGTTATTAAACGGCGTGAAACACACGCCTAAC  2340
Sbjct  2334  .....T.............................G........................  2393

Query  2341  ATACTATACATCGACGAAGCGTTCTCATGCCATGCAGGGACTCTGTTGGCCACTATAGCC  2400
Sbjct  2394  ............................................................  2453

Query  2401  ATAGTCAGGCCCAAACAGAAAGTGGTACTGTGCGGAGACCCGAAACAATGCGGATTCTTC  2460
Sbjct  2454  T...........................................................  2513

Query  2461  AATATGATGCAACTGAAAGTTAATTACAATCATGACATCTGCTCAGAAGTCTTCCACAAA  2520
Sbjct  2514  .......................C....................................  2573

Query  2521  AGTATCTCTAGACGGTGCACCCAGGATATCACGGCCATCGTTTCCAAATTACATTACCAG  2580
Sbjct  2574  ............................................T...............  2633

Query  2581  GACCGAATGAGGACCACAAACCCCCGAAAAGGAGACATCATTATAGACACTACCGGCACT  2640
Sbjct  2634  ............................................................  2693
```

```
Query  2641  ACCAAACCAGCCAAAACAGATCTGATTCTGACGTGCTTCAGGGGATGGGTGAAACAGTTG  2700
Sbjct  2694  ....................C.......................................  2753

Query  2701  CAGCAAGACTACAGAGGTAACGAAGTAATGACGGCTGCAGCGTCCCAAGGACTGACGAGG  2760
Sbjct  2754  ............................................................  2813

Query  2761  GCCTCCGTATATGCGGTTCGAACTAAAGTCAATGAGAACCCGCTATATGCACAGACCTCC  2820
Sbjct  2814  ............................................................  2873

Query  2821  GAGCACGTGAACGTGTTGTTAACACGCACAGAAAACAAGCTAGTATGGAAGACCTTGTCA  2880
Sbjct  2874  .......................................T....................  2933

Query  2881  ACAGATCCCTGGATTAAAACACTGACTAACCCACCTAGAGGGCACTATACCGCCACCATA  2940
Sbjct  2934  ............................................................  2993

Query  2941  GCAGAATGGGAAGCGGAACACCAGGGTATAATGAAGGCCATACAAGGGTATGCACCGCCC  3000
Sbjct  2994  ..............A.............................................  3053

Query  3001  GTGAACACCTTCATGAACAAAGTAAATGTGTGCTGGGCAAAGACACTTACGCCTGTGCTG  3060
Sbjct  3054  ...............................................C..A.........  3113

Query  3061  GAAACTGCGGGTATCTCCCTGTCAGCAGAAGACTGGTCTGAACTGCTGCCCCCGTTTGCC  3120
Sbjct  3114  ............................................................  3173

Query  3121  CAGGACGTGGCGTACTCACCCGAGGTGGCATTAAACATCATATGCACGAAAATGTATGGG  3180
Sbjct  3174  ...............................................A............  3233

Query  3181  TTTGACTTAGACACTGGTCTTTTTTCCAGGCCATCAGTGCCAATGACATACACCAAAGAC  3240
Sbjct  3234  ........G.....C.............A.........A.....................  3293

Query  3241  CATTGGGATAACAGAGTTGGAGGGAAAATGTATGGATTCAGCCAACAAGCATACGATCAG  3300
Sbjct  3294  ............................................................  3353

Query  3301  CTGGCAAGACGACATCCGTACCTTCGAGGTAGAGAGAAATCAGGAATGCAGATCGTAGTC  3360
Sbjct  3354  ...................................A........................  3413

Query  3361  ACTGAAATGCGTATCCAGCGCCCAAGATCGGATGCCAACATCATCCCGATCAACCGCAGG  3420
Sbjct  3414  .....G......................................................  3473

Query  3421  CTCCCTCACTCACTCGTAGCCACACACGAGTATAGGCGAGCTGCACGGGCCGAGGAATTC  3480
Sbjct  3474  ............................................G...............  3533

Query  3481  TTCACCACGACACGAGGGTACACTATGCTGCTGGTCTCTGAGTATAACATGAACTTACCA  3540
Sbjct  3534  ..........T.................................................  3593

Query  3541  AACAAGAAGATCACCTGGCTGGCTCCGATAGGGACGCAGGGGGCCCATCACACCGCCAAC  3600
Sbjct  3594  ............................................................  3653

Query  3601  CTAAACTTGGGGATACCACCTCTGCTGGGCAGTTTTGATGCGGTGGTTGTGAACATGCCG  3660
Sbjct  3654  ............................................................  3713

Query  3661  ACTCCATTCCGGAACCATCACTACCAGCAATGTGAAGACCACGCGATGAAACTCCAGATG  3720
Sbjct  3714  ................................C...........................  3773
```

```
Query  3721  CTGGCAGGCGACGCACTGAGGCACATTAAACCTGGCGGATCATTGTGGGTCAAGGCATAC  3780
Sbjct  3774  ............................................................  3833

Query  3781  GGCTACGCAGACCGGCACAGCGAGCACGTGGTCTTGGCATTGGCTAGAAAGTTTAAAAGC  3840
Sbjct  3834  .......................................C....................  3893

Query  3841  TTCAGAGTCACACAACCCTCATGCGTGACTTCCAACACCGAGGTGTTTCTCCACTTCTCA  3900
Sbjct  3894  ...........G........G.....A.................................  3953

Query  3901  ATTTTTGACAATGGCAAACGCGCGATAGCCCTGCATTCAGCTAATAGGAAGGCTAACAGT  3960
Sbjct  3954  ............................................................  4013

Query  3961  ATCTTCCAAAACACCTTCTTACCGGCGGGCAGTGCACCGGCGTACAGAGTCAAACGTGGA  4020
Sbjct  4014  ..........................A....A............................  4073

Query  4021  GACATTTCGAACGCCCCAGAGGATGCAGTGGTCAATGCAGCAAACCAACAGGGAGTGAAG  4080
Sbjct  4074  ............................................................  4133

Query  4081  GGTGCTGGAGTTTGCGGTGCAATTTACCGTAAGTGGCCGGACGCTTTCGGTGATGTCGCT  4140
Sbjct  4134  ............................................................  4193

Query  4141  ACTCCAACCGGAACAGCAGTTTCGAAATCCGTCCAAGATAAATTGGTGATCCACGCTGTC  4200
Sbjct  4194  ............................................................  4253

Query  4201  GGCCCGAATTTCTCAAAATGTTCAGAAGAGGAAGGGGACAGAGACCTAGCATCTGCTTAC  4260
Sbjct  4254  ............................................................  4313

Query  4261  AGAGCTGCAGCAGAAATAGTGATGGATaaaaaaaTTACAACAGTGGCCGTCCCCTTACTC  4320
Sbjct  4314  ..........................A.................................  4373

Query  4321  TCCACCGGCATTTATGCCGGAGGAAAAAACAGAGTAGAACAGTCACTCAACCATCTCTTC  4380
Sbjct  4374  ..............C........G....................................  4433

Query  4381  ACGGCATTCGACAATACTGATGCAGATGTGACCATATATTGCATGGACAAAACATGGGAA  4440
Sbjct  4434  ............................................................  4493

Query  4441  AAGAAGATTAAGGAGGCAATCGATCACCGGACTTCGGTTGAGATGGTGCAGGATGACGTG  4500
Sbjct  4494  ............................................................  4553

Query  4501  CAGTTGGAGGAGGAACTGGTACGAGTACACCCTTTGAGTAGTTTAGCAGGTAGGAAGGGT  4560
Sbjct  4554  ............................................................  4613

Query  4561  TACAGTACGGACAGCGGCCGAGTGTTTTCCTACCTGGAAGGTACCAAATTCCATCAGACT  4620
Sbjct  4614  ............................................................  4673

Query  4621  GCGGTGGACATAGCCGAAATGCAAGTGCTGTGGCCCGCCCTCAAAGAGTCTAATGAGCAA  4680
Sbjct  4674  ........T.............................T.....................  4733

Query  4681  ATAGTGGCATACACCTTAGGAGAATCAATGGACCAGATACGTGGCAAGTGCCCGACAGAA  4740
Sbjct  4734  ............................................................  4793

Query  4741  GATACTGACGCCTCCACACCTCCACGGACTGTGCCGTGCCTCTGTCGATACGCCATGACA  4800
Sbjct  4794  ............................................................  4853
```

```
Query  4801  CCAGAGAGAGTGTACCGACTTAAATGCACGAACACTACCCAATTTACGGTTTGCTCATCT  4860
Sbjct  4854  ............................................C.....C.........  4913

Query  4861  TTTGAGTTGCCAAAGTATCACATTCAGGGAGTGCAGAGAGTAAAATGTGAAAGAATCATC  4920
Sbjct  4914  ............................................................  4973

Query  4921  ATCTTAGATCCCACTGTTCCACCAACTTACAAACGGCCATGCATCAGACGGTACCCCTCC  4980
Sbjct  4974  ............................................................  5033

Query  4981  ACAATCTCTTGTAACTCCTCTGAGGACTCCAGGAGCTTGTCTACTTTTTCTGTCAGCTCC  5040
Sbjct  5034  ........C......................................C............  5093

Query  5041  GACTCCTCGATTGGTTCTCTGCCGGTCGGAGACACGAGACCCATTCCAGCCCCGAGGACC  5100
Sbjct  5094  .................CG..A...................T...........A......  5153

Query  5101  ATTTTCAGACCCGTCCCTGCCCCGAGAGCACCCGTGCTCAGAACCACACCGCCTCCTAAA  5160
Sbjct  5154  G..........................................T................  5213

Query  5161  CCACCGCGCACATTCACCGTGCGTGCAGAAGTGCACCAAGCACCCCCTACACCTGTACCT  5220
Sbjct  5214  ...................................T........................  5273

Query  5221  CCACCCAGACCGAAGAGGGCTGCAAAGTTGGCTCGTGAGATGCACCCCGGGTTCACCTTC  5280
Sbjct  5274  ............................................................  5333

Query  5281  GGGGACTTCGGAGAGCACGAGGTTGAGGAGCTTACGGCCTCTCCCTTAACCTTCGGAGAT  5340
Sbjct  5334  ..A..................A.................G....................  5393

Query  5341  TTTGCTGAAGGAGAGATCCAGGGGATGGGAGTGGAGTTTGAATGACTAGGCAGAGCCGGC  5400
Sbjct  5394  ............................................................  5453

Query  5401  GGGTACATTTTTTCGTCAGACACGGGTCCAGGCCACCTACAGCAGAGATCCGTTTTACAA  5460
Sbjct  5454  .........................................A..................  5513

Query  5461  AATTGCACGGCAGAATGTATCTACGAACCGGCAAAACTAGAAAAAATTCATGCACCAAAG  5520
Sbjct  5514  ............................................................  5573

Query  5521  TTGGATAAAACCAAGGAAGATATCTTAAGGAGCAAGTACCAAATGAAACCGTCTGAAGCA  5580
Sbjct  5574  ............................................................  5633

Query  5581  AACAAAAGCAGGTACCAATCTAGAAAAGTAGAAAATATGAAAGCAGAGATCGTAGGTAGA  5640
Sbjct  5634  ........T.........................................T.........  5693

Query  5641  CTCTTGGACGGACTGGGGGAGTATCTGGGCACCGAGCATCCAGTTGAATGCTACCGAATA  5700
Sbjct  5694  ..............A.............................................  5753

Query  5701  ACGTACCCGGTGCCTATATACTCAACTAGTGACCTCAGAGGTCTGTCTAGTGCCAAAACA  5760
Sbjct  5754  .....T........................AT..G.........................  5813

Query  5761  GCTGTTAGAGCTTGCAATGCATTTTTGGAAGCTAATTTTCCATCAGTCACTTCATATAAA  5820
Sbjct  5814  ............................................................  5873

Query  5821  ATTACTGATGAATACGACGCATACCTAGATATGGTAGATGGATCAGAGAGCTGTCTGGAC  5880
Sbjct  5874  ............................................................  5933
```

```
Query  5881  AGATCCTCCTTTTCGCCGTCTAGATTGCGTAGCTTTCCAAAAACACACTCATACTTGGAC  5940
Sbjct  5934  .................A...........C..............................  5993

Query  5941  CCACAGATCAACAGTGCGGTACCGTCACCATTCCAAAACACCTTACAAAATGTATTGGCA  6000
Sbjct  5994  ..G.........................................................  6053

Query  6001  GCGGCCACCAAAAGAAACTGTAATGTCACACAGATGAGAGAACTACCAACATATGATTCT  6060
Sbjct  6054  ...............................................G............  6113

Query  6061  GCAGTGCTAAATGTAGAGGCCTTCAGGAAATATGCGTGCAAGCCAGACGTATGGGATGAG  6120
Sbjct  6114  ............................................................  6173

Query  6121  TACAGGGATAATCCGATTTGCATAACCACCGAAAATGTCACCACTTACGTCGCCAAGTTG  6180
Sbjct  6174  ..............A......................................T......  6233

Query  6181  AAAGGACCGAAAGCTGCGGCCTTGTTTGCAAAAACACATAACCTGATACCACTACACCAA  6240
Sbjct  6234  ............................................................  6293

Query  6241  GTTCCTATGGACAAATTCACGGTAGATATGAAGAGAGATGTCAAAGTCACGCCCGGAACC  6300
Sbjct  6294  ............................................................  6353

Query  6301  AAGCACACCGAAGAGAGACCAAAGGTACAGGTGATTCAAGCGGCAGAGCCACTAGCCACT  6360
Sbjct  6354  ............................................................  6413

Query  6361  GCCTACCTCTGCGGAATTCACCGTGAATTGGTGCGCCGTCTCAACAACGCGCTTTTCCCA  6420
Sbjct  6414  .....................................................C......  6473

Query  6421  AACATCCACACTTTGTTTGATATGTCCGCAGAGGATTTCGATGCAATCATAGCGGAACAT  6480
Sbjct  6474  ..T...................................T.....................  6533

Query  6481  TTTAAGCACGGTGACCATGTGTTGGAAACGGATATAGCCTCTTTTGACAAAAGTCAAGAT  6540
Sbjct  6534  .................C..........................................  6593

Query  6541  GATTCCATGGCACTCACTGCGTTAATGATCCTTGAGGACCTGGGAGTAGACCAAAACCTA  6600
Sbjct  6594  ............................................................  6653

Query  6601  ATGAATTTGATAGAGGCTGCATTCGGGGAAATCGTGAGTACACACTTGCCCACAGGTACT  6660
Sbjct  6654  ............................................................  6713

Query  6661  AGATTCAAATTTGGAGCTATGATGAAGTCTGGAATGTTTTTGACGCTGTTCGTCAATACA  6720
Sbjct  6714  .......G.....................C...............T..............  6773

Query  6721  ATTCTTAATGTGGTTATTGCGTGCCGAGTGTTGGAGGATCAATTGGCGCAGTCGCCGTGG  6780
Sbjct  6774  ........C..................................................C  6833

Query  6781  CCTGCTTTCATAGGAGATGACAACATAATCCATGGTATAATATCAGACAAATTGATGGCA  6840
Sbjct  6834  G..........................................................G  6893

Query  6841  GATAGATGTGCCACCTGGATGAACATGGAGGTCAAGATACTGGACTCTATAGTTGGAATA  6900
Sbjct  6894  ............................................T..C............  6953

Query  6901  CGGCCACCTTACTTCTGTGGAGGATTTATTGTATGTGACGATGTAACAGGTACAGCCTGC  6960
Sbjct  6954  ...........T................................................  7013
```

```
Query  6961  CGCGTCGCAGACCCACTGAAGAGATTGTTCAAGCTAGGTAAGCCATTGCCACTTGACGAT  7020
Sbjct  7014  ........................C...................................  7073

Query  7021  GGCCAAGATGAAGACAGAAGACGTGCATTACATGATGAAGTGAAAACCTGGTCGCGCGTA  7080
Sbjct  7074  ............................................................  7133

Query  7081  GGGCTGCGACACAGAGTGTGTGAAGCCATCGAAGACCGTTATGCCGTCCACTCATCAGAA  7140
Sbjct  7134  ..................................................T.........  7193

Query  7141  CTAGTTTTATTGGCACTGACTACTCTGTCTAAGAACTTGAAGTCCTTCAGAAACATAAGA  7200
Sbjct  7194  ............................................................  7253

Query  7201  GGGAAACCAATACATCTCTACGGTGGTCCTAAATAG  7236
Sbjct  7254  ....................................  7289
```

>Barmah Forest virus isolate SW26969, complete genome
Sequence ID: MN689032.1 Length: 11541
Range 1: 57 to 7292

Score:12778 bits(6919), Expect:0.0,
Identities:7130/7236(99%),  Gaps:0/7236(0%), Strand: Plus/Plus

```
Query  1     ATGGCGAAACCAGTTGTGAAGATCGACGTGGAACCTGAAAGCCATTTCGCTAAGCAGGTC  60
Sbjct  57    ...............................................T............  116

Query  61    CAGAGTTGCTTCCCGCAGTTTGAGATCGAAGCAGTGCAGACCACACCAAACGATCATGCA  120
Sbjct  117   .........................................G..................  176

Query  121   CACGCGAGGGCGTTTTCGCACCTTGCTACGAAGCTCATAGAAATGGAGACAGCAAAAGAT  180
Sbjct  177   ............................................................  236

Query  181   CAGATCATCCTCGATATCGGAAGTGCACCCGCGAGGAGACTGTATTCAGAACACAAGTAC  240
Sbjct  237   ............................................................  296

Query  241   CACTGTGTTTGCCCAATGAAGTGCACGGAAGATCCAGAGAGAATGCTAGGATATGCACGT  300
Sbjct  297   ............................................................  356

Query  301   AAGTTGATCGCAGGCTCTGCGAAAGGGAAGGCAGAAAAGTTACGCGATCTCAGGGATGTC  360
Sbjct  357   ............................................................  416

Query  361   TTGGCTACGCCAGACATCGAGACGCAGTCGCTATGTCTCCACACAGACGCATCCTGCAGA  420
Sbjct  417   ............................................................  476

Query  421   TACCGCGGTGATGTTGCCGTGTATCAAGACGTGTATGCCATTGACGCACCTACCACGCTG  480
Sbjct  477   ..................................................C.........  536

Query  481   TACCACCAAGCGTTAAAGGGCGTCAGGACCGCATATTGGATAGGCTTTGATACAACGCCG  540
Sbjct  537   ...........................................................A  596

Query  541   TTCATGTACGATGCACTAGCAGGAGCTTACCCGCTCTACTCCACAAACTGGGCTGATGAG  600
```

```
Sbjct  597    .........A...........................................C......  656

Query  601    CAAGTGCTCGAGTCCAGAAACATTGGGCTATGTTCAGACAAAGTTTCTGAAGGGGGAAAG  660
Sbjct  657    ............................................................  716

Query  661    AAAGGGAGATCAATCCTCAGGAAGAAGTTCTTGAAGCAGTCAGACAGAGTCATGTTCTCT  720
Sbjct  717    ............................................................  776

Query  721    GTCGGCTCGACGTTGTATACGGAAAGCCGTAAATTACTGCAAAGTTGGCACCTGCCATCC  780
Sbjct  777    ............................................C...............  836

Query  781    ACATTCCATCTCAAAGGCAAATCTTCGTTCACGTGCCGCTGCGACACTATCGTCAGCTGC  840
Sbjct  837    ................................A..............C............  896

Query  841    GAAGGGTATGTTCTGAAGAAAATTACAATGTGTCCTGGAGTGACAGGCAAACCGATAGGA  900
Sbjct  897    ................................C..C........................  956

Query  901    TATGCCGTCACCCATCACAAAGAAGGATTCGTAGTCGGAAAAGTCACAGATACCATTCGC  960
Sbjct  957    ....................G.............................C.........  1016

Query  961    GGCGAGAGAGTCTCCTTCGCCGTGTGTACTTATGTACCAACAACACTCTGCGACCAGATG  1020
Sbjct  1017   ............................................................  1076

Query  1021   ACCGGGATCCTAGCAACAGAAGTAACAGCCGATGATGCCCAGAAACTGCTGGTGGGTTTG  1080
Sbjct  1077   ........T...................................................  1136

Query  1081   AACCAGAGAATAGTAGTTAATGGTAGGACCCAGAGAAATACCAATACTATGAAGAACTAC  1140
Sbjct  1137   ............................................C...............  1196

Query  1141   CTGCTACCACTGGTTGCACAAGCGCTAGCAAAATGGGCGAAGGAAGCAAAACAGGATATG  1200
Sbjct  1197   ......................................A.....................  1256

Query  1201   GAAGATGAAAGACCCCTGAACGAACGCCAACGAACGCTAACGTGCCTCTGCTGCTGGGCA  1260
Sbjct  1257   ............................................................  1316

Query  1261   TTTAAGCGAAACAAACGCCACGCCATTTACAAGAGACCAGACACACAGAGTATAGTCAAG  1320
Sbjct  1317   ............................................................  1376

Query  1321   GTCCCTTGCGAATTCACAAGCTTTCCTTTGGTCAGCCTGTGGTCCGCTGGGATGTCTATA  1380
Sbjct  1377   ............................................................  1436

Query  1381   TCTCTTAGGCAGAAGTTGAAGATGATGCTGCAGGCGAGGCAGCCCACACAAATAGCAGCA  1440
Sbjct  1437   ............................................................  1496

Query  1441   GTGACTGAGGAACTCATACAAGAAGCAGCTGCAGTAGAGCAAGAGGCCGTGGATACGGCC  1500
Sbjct  1497   ..................................C.........................  1556

Query  1501   AATGCCGAGCTGGACCACGCCGCATGGCCCTCCATTGTGGATACGACAGAGCGCCATGTT  1560
Sbjct  1557   ...............................................G............  1616

Query  1561   GAGGTCGAAGTGGAAGAACTCGACCAGCGTGCAGGGGAAGGGGTAGTGGAAACACCTCGA  1620
Sbjct  1617   ............................................................  1676

Query  1621   AACTCTATCAAAGTTTCAACACAGATCGGGGACGCGTTAATCGGCAGTTACCTGATCCTA  1680
```

```
Sbjct  1677  ........T...................................................  1736

Query  1681  TCACCCCAAGCAGTCCTACGCAGCGAAAAATTAGCCTGCATACATGATCTTGCAGAGCAG  1740
Sbjct  1737  ............................................................  1796

Query  1741  GTTAAGTTGGTCACACACTCTGGCCGTAGTGGTAGGTACGCCGTCGACAAATACNACGGA  1800
Sbjct  1797  ..C................C..T............................G.....  1856

Query  1801  AGAGTACTAGTCCCTACAGGAGTGGCTATAGACATTCAATCGTTCCAGGCTCTCAGTGAG  1860
Sbjct  1857  ............................................................  1916

Query  1861  AGCGCGACCCTTGTGTACAACGAACGCGAGTTCGTTAACAGGAAGCTGTGGCACATAGCA  1920
Sbjct  1917  ..T.......................................T..............  1976

Query  1921  GTATACGGGGCAGCACTCAATACTGATGAAGAAGGATACGAGAAGGTCCCGGTAGAGAGA  1980
Sbjct  1977  ............................................................  2036

Query  1981  GCAGAATCAGATTATGTGTTTGATGTAGACCAAAAAATGTGCCTaaaaaaaGAGCAGGCA  2040
Sbjct  2037  ..................A............A.............C...............  2096

Query  2041  TCAGGTTGGGTACTCTGTGGCGAACTAGTCAACCCCCCATTCCACGAATTCGCATATGAA  2100
Sbjct  2097  .....A......................................................  2156

Query  2101  GGGCTCCGCACGAGACCGTCAGCACCCTACAAGGTTCATACAGTAGGTGTGTACGGAGTG  2160
Sbjct  2157  ........T...................................................  2216

Query  2161  CCAGGATCAGGCAAATCCGCAATAATCAAGAACACGGTCACCATGTCTGACCTAGTATTG  2220
Sbjct  2217  ............................................................  2276

Query  2221  AGTGGTAAGAAAGAGAACTGCTTAGAAATTATGAACGATGTACTTAAACACAGAGCTCTA  2280
Sbjct  2277  ............................................................  2336

Query  2281  CGTATCACAGCGAAGACCGTAGACTCAGTGTTATTAAACGGCGTGAAACACACGCCTAAC  2340
Sbjct  2337  .....T...........................G.........................  2396

Query  2341  ATACTATACATCGACGAAGCGTTCTCATGCCATGCAGGGACTCTGTTGGCCACTATAGCC  2400
Sbjct  2397  ............................................................  2456

Query  2401  ATAGTCAGGCCCAAACAGAAAGTGGTACTGTGCGGAGACCCGAAACAATGCGGATTCTTC  2460
Sbjct  2457  T...........................................................  2516

Query  2461  AATATGATGCAACTGAAAGTTAATTACAATCATGACATCTGCTCAGAAGTCTTCCACAAA  2520
Sbjct  2517  ......................C.....................................  2576

Query  2521  AGTATCTCTAGACGGTGCACCCAGGATATCACGGCCATCGTTTCCAAATTACATTACCAG  2580
Sbjct  2577  ..........................................T...............  2636

Query  2581  GACCGAATGAGGACCACAAACCCCCGAAAAGGAGACATCATTATAGACACTACCGGCACT  2640
Sbjct  2637  ............................................................  2696

Query  2641  ACCAAACCAGCCAAAACAGATCTGATTCTGACGTGCTTCAGGGGATGGGTGAAACAGTTG  2700
Sbjct  2697  ...................C........................................  2756

Query  2701  CAGCAAGACTACAGAGGTAACGAAGTAATGACGGCTGCAGCGTCCCAAGGACTGACGAGG  2760
```

```
Sbjct  2757  ............................................................  2816

Query  2761  GCCTCCGTATATGCGGTTCGAACTAAAGTCAATGAGAACCCGCTATATGCACAGACCTCC  2820
Sbjct  2817  ............................................................  2876

Query  2821  GAGCACGTGAACGTGTTGTTAACACGCACAGAAAACAAGCTAGTATGGAAGACCTTGTCA  2880
Sbjct  2877  .......................................T....................  2936

Query  2881  ACAGATCCCTGGATTAAAACACTGACTAACCCACCTAGAGGGCACTATACCGCCACCATA  2940
Sbjct  2937  ............................................................  2996

Query  2941  GCAGAATGGGAAGCGGAACACCAGGGTATAATGAAGGCCATACAAGGGTATGCACCGCCC  3000
Sbjct  2997  ..............A.............................................  3056

Query  3001  GTGAACACCTTCATGAACAAAGTAAATGTGTGCTGGGCAAAGACACTTACGCCTGTGCTG  3060
Sbjct  3057  ...............................................C..A.........  3116

Query  3061  GAAACTGCGGGTATCTCCCTGTCAGCAGAAGACTGGTCTGAACTGCTGCCCCCGTTTGCC  3120
Sbjct  3117  ............................................................  3176

Query  3121  CAGGACGTGGCGTACTCACCCGAGGTGGCATTAAACATCATATGCACGAAAATGTATGGG  3180
Sbjct  3177  ...............................................A............  3236

Query  3181  TTTGACTTAGACACTGGTCTTTTTTCCAGGCCATCAGTGCCAATGACATACACCAAAGAC  3240
Sbjct  3237  ........G.....C.............A.........A.....................  3296

Query  3241  CATTGGGATAACAGAGTTGGAGGGAAAATGTATGGATTCAGCCAACAAGCATACGATCAG  3300
Sbjct  3297  ............................................................  3356

Query  3301  CTGGCAAGACGACATCCGTACCTTCGAGGTAGAGAGAAATCAGGAATGCAGATCGTAGTC  3360
Sbjct  3357  ...................................A........................  3416

Query  3361  ACTGAAATGCGTATCCAGCGCCCAAGATCGGATGCCAACATCATCCCGATCAACCGCAGG  3420
Sbjct  3417  .....G......................................................  3476

Query  3421  CTCCCTCACTCACTCGTAGCCACACACGAGTATAGGCGAGCTGCACGGGCCGAGGAATTC  3480
Sbjct  3477  ............................................G...............  3536

Query  3481  TTCACCACGACACGAGGGTACACTATGCTGCTGGTCTCTGAGTATAACATGAACTTACCA  3540
Sbjct  3537  ..........T.................................................  3596

Query  3541  AACAAGAAGATCACCTGGCTGGCTCCGATAGGGACGCAGGGGGCCCATCACACCGCCAAC  3600
Sbjct  3597  ............................................................  3656

Query  3601  CTAAACTTGGGGATACCACCTCTGCTGGGCAGTTTTGATGCGGTGGTTGTGAACATGCCG  3660
Sbjct  3657  ............................................................  3716

Query  3661  ACTCCATTCCGGAACCATCACTACCAGCAATGTGAAGACCACGCGATGAAACTCCAGATG  3720
Sbjct  3717  ................................C...........................  3776

Query  3721  CTGGCAGGCGACGCACTGAGGCACATTAAACCTGGCGGATCATTGTGGGTCAAGGCATAC  3780
Sbjct  3777  ............................................................  3836

Query  3781  GGCTACGCAGACCGGCACAGCGAGCACGTGGTCTTGGCATTGGCTAGAAAGTTTAAAAGC  3840
```

```
Sbjct  3837  .......................................C....................  3896

Query  3841  TTCAGAGTCACACAACCCTCATGCGTGACTTCCAACACCGAGGTGTTTCTCCACTTCTCA  3900
Sbjct  3897  ...........G........G.....A.................................  3956

Query  3901  ATTTTTGACAATGGCAAACGCGCGATAGCCCTGCATTCAGCTAATAGGAAGGCTAACAGT  3960
Sbjct  3957  ............................................................  4016

Query  3961  ATCTTCCAAAACACCTTCTTACCGGCGGGCAGTGCACCGGCGTACAGAGTCAAACGTGGA  4020
Sbjct  4017  ..........................A....A............................  4076

Query  4021  GACATTTCGAACGCCCCAGAGGATGCAGTGGTCAATGCAGCAAACCAACAGGGAGTGAAG  4080
Sbjct  4077  ............................................................  4136

Query  4081  GGTGCTGGAGTTTGCGGTGCAATTTACCGTAAGTGGCCGGACGCTTTCGGTGATGTCGCT  4140
Sbjct  4137  ............................................................  4196

Query  4141  ACTCCAACCGGAACAGCAGTTTCGAAATCCGTCCAAGATAAATTGGTGATCCACGCTGTC  4200
Sbjct  4197  ............................................................  4256

Query  4201  GGCCCGAATTTCTCAAAATGTTCAGAAGAGGAAGGGGACAGAGACCTAGCATCTGCTTAC  4260
Sbjct  4257  ............................................................  4316

Query  4261  AGAGCTGCAGCAGAAATAGTGATGGATaaaaaaaTTACAACAGTGGCCGTCCCCTTACTC  4320
Sbjct  4317  ..........................A.................................  4376

Query  4321  TCCACCGGCATTTATGCCGGAGGAAAAAACAGAGTAGAACAGTCACTCAACCATCTCTTC  4380
Sbjct  4377  ..............C........G....................................  4436

Query  4381  ACGGCATTCGACAATACTGATGCAGATGTGACCATATATTGCATGGACAAAACATGGGAA  4440
Sbjct  4437  ............................................................  4496

Query  4441  AAGAAGATTAAGGAGGCAATCGATCACCGGACTTCGGTTGAGATGGTGCAGGATGACGTG  4500
Sbjct  4497  ............................................................  4556

Query  4501  CAGTTGGAGGAGGAACTGGTACGAGTACACCCTTTGAGTAGTTTAGCAGGTAGGAAGGGT  4560
Sbjct  4557  ............................................................  4616

Query  4561  TACAGTACGGACAGCGGCCGAGTGTTTTCCTACCTGGAAGGTACCAAATTCCATCAGACT  4620
Sbjct  4617  ............................................................  4676

Query  4621  GCGGTGGACATAGCCGAAATGCAAGTGCTGTGGCCCGCCCTCAAAGAGTCTAATGAGCAA  4680
Sbjct  4677  ........T.............................T.....................  4736

Query  4681  ATAGTGGCATACACCTTAGGAGAATCAATGGACCAGATACGTGGCAAGTGCCCGACAGAA  4740
Sbjct  4737  ............................................................  4796

Query  4741  GATACTGACGCCTCCACACCTCCACGGACTGTGCCGTGCCTCTGTCGATACGCCATGACA  4800
Sbjct  4797  ............................................................  4856

Query  4801  CCAGAGAGAGTGTACCGACTTAAATGCACGAACACTACCCAATTTACGGTTTGCTCATCT  4860
Sbjct  4857  ............................................C.....C.........  4916

Query  4861  TTTGAGTTGCCAAAGTATCACATTCAGGGAGTGCAGAGAGTAAAATGTGAAAGAATCATC  4920
```

```
Sbjct  4917  ............................................................  4976

Query  4921  ATCTTAGATCCCACTGTTCCACCAACTTACAAACGGCCATGCATCAGACGGTACCCCTCC  4980
Sbjct  4977  ............................................................  5036

Query  4981  ACAATCTCTTGTAACTCCTCTGAGGACTCCAGGAGCTTGTCTACTTTTTCTGTCAGCTCC  5040
Sbjct  5037  ........C......................................C............  5096

Query  5041  GACTCCTCGATTGGTTCTCTGCCGGTCGGAGACACGAGACCCATTCCAGCCCCGAGGACC  5100
Sbjct  5097  .................CG..A...................T...........A......  5156

Query  5101  ATTTTCAGACCCGTCCCTGCCCCGAGAGCACCCGTGCTCAGAACCACACCGCCTCCTAAA  5160
Sbjct  5157  G..........................................T................  5216

Query  5161  CCACCGCGCACATTCACCGTGCGTGCAGAAGTGCACCAAGCACCCCCTACACCTGTACCT  5220
Sbjct  5217  ...................................T........................  5276

Query  5221  CCACCCAGACCGAAGAGGGCTGCAAAGTTGGCTCGTGAGATGCACCCCGGGTTCACCTTC  5280
Sbjct  5277  ............................................................  5336

Query  5281  GGGGACTTCGGAGAGCACGAGGTTGAGGAGCTTACGGCCTCTCCCTTAACCTTCGGAGAT  5340
Sbjct  5337  ..A..................A.................G....................  5396

Query  5341  TTTGCTGAAGGAGAGATCCAGGGGATGGGAGTGGAGTTTGAATGACTAGGCAGAGCCGGC  5400
Sbjct  5397  ............................................................  5456

Query  5401  GGGTACATTTTTTCGTCAGACACGGGTCCAGGCCACCTACAGCAGAGATCCGTTTTACAA  5460
Sbjct  5457  .........................................A..................  5516

Query  5461  AATTGCACGGCAGAATGTATCTACGAACCGGCAAAACTAGAAAAAATTCATGCACCAAAG  5520
Sbjct  5517  ............................................................  5576

Query  5521  TTGGATAAAACCAAGGAAGATATCTTAAGGAGCAAGTACCAAATGAAACCGTCTGAAGCA  5580
Sbjct  5577  ............................................................  5636

Query  5581  AACAAAAGCAGGTACCAATCTAGAAAAGTAGAAAATATGAAAGCAGAGATCGTAGGTAGA  5640
Sbjct  5637  ........T.........................................T.........  5696

Query  5641  CTCTTGGACGGACTGGGGGAGTATCTGGGCACCGAGCATCCAGTTGAATGCTACCGAATA  5700
Sbjct  5697  ..............A.............................................  5756

Query  5701  ACGTACCCGGTGCCTATATACTCAACTAGTGACCTCAGAGGTCTGTCTAGTGCCAAAACA  5760
Sbjct  5757  .....T........................AT..G.........................  5816

Query  5761  GCTGTTAGAGCTTGCAATGCATTTTTGGAAGCTAATTTTCCATCAGTCACTTCATATAAA  5820
Sbjct  5817  ............................................................  5876

Query  5821  ATTACTGATGAATACGACGCATACCTAGATATGGTAGATGGATCAGAGAGCTGTCTGGAC  5880
Sbjct  5877  ............................................................  5936

Query  5881  AGATCCTCCTTTTCGCCGTCTAGATTGCGTAGCTTTCCAAAAACACACTCATACTTGGAC  5940
Sbjct  5937  .................A...........C..............................  5996

Query  5941  CCACAGATCAACAGTGCGGTACCGTCACCATTCCAAAACACCTTACAAAATGTATTGGCA  6000
```

```
Sbjct  5997  ..G.........................................................  6056

Query  6001  GCGGCCACCAAAAGAAACTGTAATGTCACACAGATGAGAGAACTACCAACATATGATTCT  6060
Sbjct  6057  ...............................................G............  6116

Query  6061  GCAGTGCTAAATGTAGAGGCCTTCAGGAAATATGCGTGCAAGCCAGACGTATGGGATGAG  6120
Sbjct  6117  ............................................................  6176

Query  6121  TACAGGGATAATCCGATTTGCATAACCACCGAAAATGTCACCACTTACGTCGCCAAGTTG  6180
Sbjct  6177  ..............A......................................T......  6236

Query  6181  AAAGGACCGAAAGCTGCGGCCTTGTTTGCAAAAACACATAACCTGATACCACTACACCAA  6240
Sbjct  6237  ............................................................  6296

Query  6241  GTTCCTATGGACAAATTCACGGTAGATATGAAGAGAGATGTCAAAGTCACGCCCGGAACC  6300
Sbjct  6297  ............................................................  6356

Query  6301  AAGCACACCGAAGAGAGACCAAAGGTACAGGTGATTCAAGCGGCAGAGCCACTAGCCACT  6360
Sbjct  6357  ............................................................  6416

Query  6361  GCCTACCTCTGCGGAATTCACCGTGAATTGGTGCGCCGTCTCAACAACGCGCTTTTCCCA  6420
Sbjct  6417  .....................................................C......  6476

Query  6421  AACATCCACACTTTGTTTGATATGTCCGCAGAGGATTTCGATGCAATCATAGCGGAACAT  6480
Sbjct  6477  ..T...................................T.....................  6536

Query  6481  TTTAAGCACGGTGACCATGTGTTGGAAACGGATATAGCCTCTTTTGACAAAAGTCAAGAT  6540
Sbjct  6537  .................C..........................................  6596

Query  6541  GATTCCATGGCACTCACTGCGTTAATGATCCTTGAGGACCTGGGAGTAGACCAAAACCTA  6600
Sbjct  6597  ............................................................  6656

Query  6601  ATGAATTTGATAGAGGCTGCATTCGGGGAAATCGTGAGTACACACTTGCCCACAGGTACT  6660
Sbjct  6657  ............................................................  6716

Query  6661  AGATTCAAATTTGGAGCTATGATGAAGTCTGGAATGTTTTTGACGCTGTTCGTCAATACA  6720
Sbjct  6717  .......G.....................C...............T..............  6776

Query  6721  ATTCTTAATGTGGTTATTGCGTGCCGAGTGTTGGAGGATCAATTGGCGCAGTCGCCGTGG  6780
Sbjct  6777  ........C..................................................C  6836

Query  6781  CCTGCTTTCATAGGAGATGACAACATAATCCATGGTATAATATCAGACAAATTGATGGCA  6840
Sbjct  6837  G..........................................................G  6896

Query  6841  GATAGATGTGCCACCTGGATGAACATGGAGGTCAAGATACTGGACTCTATAGTTGGAATA  6900
Sbjct  6897  ............................................T..C............  6956

Query  6901  CGGCCACCTTACTTCTGTGGAGGATTTATTGTATGTGACGATGTAACAGGTACAGCCTGC  6960
Sbjct  6957  ...........T................................................  7016

Query  6961  CGCGTCGCAGACCCACTGAAGAGATTGTTCAAGCTAGGTAAGCCATTGCCACTTGACGAT  7020
Sbjct  7017  ........................C...................................  7076

Query  7021  GGCCAAGATGAAGACAGAAGACGTGCATTACATGATGAAGTGAAAACCTGGTCGCGCGTA  7080
```

```
Sbjct  7077  ............................................................  7136

Query  7081  GGGCTGCGACACAGAGTGTGTGAAGCCATCGAAGACCGTTATGCCGTCCACTCATCAGAA  7140
Sbjct  7137  ..................................................T.........  7196

Query  7141  CTAGTTTTATTGGCACTGACTACTCTGTCTAAGAACTTGAAGTCCTTCAGAAACATAAGA  7200
Sbjct  7197  ............................................................  7256

Query  7201  GGGAAACCAATACATCTCTACGGTGGTCCTAAATAG  7236
Sbjct  7257  ....................................  7292
```

```
>Barmah Forest virus isolate SW31286, complete genome
Sequence ID: MN689034.1 Length: 11523
Range 1: 66 to 7301

Score:12767 bits(6913), Expect:0.0,
Identities:7128/7236(99%),  Gaps:0/7236(0%), Strand: Plus/Plus

Query  1     ATGGCGAAACCAGTTGTGAAGATCGACGTGGAACCTGAAAGCCATTTCGCTAAGCAGGTC  60
Sbjct  66    ...............................................T............  125

Query  61    CAGAGTTGCTTCCCGCAGTTTGAGATCGAAGCAGTGCAGACCACACCAAACGATCATGCA  120
Sbjct  126   .........................................G..................  185

Query  121   CACGCGAGGGCGTTTTCGCACCTTGCTACGAAGCTCATAGAAATGGAGACAGCAAAAGAT  180
Sbjct  186   ............................................................  245

Query  181   CAGATCATCCTCGATATCGGAAGTGCACCCGCGAGGAGACTGTATTCAGAACACAAGTAC  240
Sbjct  246   ............................................................  305

Query  241   CACTGTGTTTGCCCAATGAAGTGCACGGAAGATCCAGAGAGAATGCTAGGATATGCACGT  300
Sbjct  306   ............................................................  365

Query  301   AAGTTGATCGCAGGCTCTGCGAAAGGGAAGGCAGAAAAGTTACGCGATCTCAGGGATGTC  360
Sbjct  366   ............................................................  425

Query  361   TTGGCTACGCCAGACATCGAGACGCAGTCGCTATGTCTCCACACAGACGCATCCTGCAGA  420
Sbjct  426   ............................................................  485

Query  421   TACCGCGGTGATGTTGCCGTGTATCAAGACGTGTATGCCATTGACGCACCTACCACGCTG  480
Sbjct  486   ..................................................C.........  545

Query  481   TACCACCAAGCGTTAAAGGGCGTCAGGACCGCATATTGGATAGGCTTTGATACAACGCCG  540
Sbjct  546   ...........................................................A  605

Query  541   TTCATGTACGATGCACTAGCAGGAGCTTACCCGCTCTACTCCACAAACTGGGCTGATGAG  600
Sbjct  606   .........A...........................................C......  665

Query  601   CAAGTGCTCGAGTCCAGAAACATTGGGCTATGTTCAGACAAAGTTTCTGAAGGGGGAAAG  660
Sbjct  666   ............................................................  725
```

```
Query  661   AAAGGGAGATCAATCCTCAGGAAGAAGTTCTTGAAGCAGTCAGACAGAGTCATGTTCTCT  720
Sbjct  726   ............................................................  785

Query  721   GTCGGCTCGACGTTGTATACGGAAAGCCGTAAATTACTGCAAAGTTGGCACCTGCCATCC  780
Sbjct  786   ............................................C...............  845

Query  781   ACATTCCATCTCAAAGGCAAATCTTCGTTCACGTGCCGCTGCGACACTATCGTCAGCTGC  840
Sbjct  846   ................................A..............C............  905

Query  841   GAAGGGTATGTTCTGAAGAAAATTACAATGTGTCCTGGAGTGACAGGCAAACCGATAGGA  900
Sbjct  906   ................................C..C........................  965

Query  901   TATGCCGTCACCCATCACAAAGAAGGATTCGTAGTCGGAAAAGTCACAGATACCATTCGC  960
Sbjct  966   ....................G.............................C.........  1025

Query  961   GGCGAGAGAGTCTCCTTCGCCGTGTGTACTTATGTACCAACAACACTCTGCGACCAGATG  1020
Sbjct  1026  ............................................................  1085

Query  1021  ACCGGGATCCTAGCAACAGAAGTAACAGCCGATGATGCCCAGAAACTGCTGGTGGGTTTG  1080
Sbjct  1086  ........T...................................................  1145

Query  1081  AACCAGAGAATAGTAGTTAATGGTAGGACCCAGAGAAATACCAATACTATGAAGAACTAC  1140
Sbjct  1146  ............................................C...............  1205

Query  1141  CTGCTACCACTGGTTGCACAAGCGCTAGCAAAATGGGCGAAGGAAGCAAAACAGGATATG  1200
Sbjct  1206  ......................................A.....................  1265

Query  1201  GAAGATGAAAGACCCCTGAACGAACGCCAACGAACGCTAACGTGCCTCTGCTGCTGGGCA  1260
Sbjct  1266  ............................................................  1325

Query  1261  TTTAAGCGAAACAAACGCCACGCCATTTACAAGAGACCAGACACACAGAGTATAGTCAAG  1320
Sbjct  1326  ............................................................  1385

Query  1321  GTCCCTTGCGAATTCACAAGCTTTCCTTTGGTCAGCCTGTGGTCCGCTGGGATGTCTATA  1380
Sbjct  1386  ............................................................  1445

Query  1381  TCTCTTAGGCAGAAGTTGAAGATGATGCTGCAGGCGAGGCAGCCCACACAAATAGCAGCA  1440
Sbjct  1446  ............................................................  1505

Query  1441  GTGACTGAGGAACTCATACAAGAAGCAGCTGCAGTAGAGCAAGAGGCCGTGGATACGGCC  1500
Sbjct  1506  ..................................C.........................  1565

Query  1501  AATGCCGAGCTGGACCACGCCGCATGGCCCTCCATTGTGGATACGACAGAGCGCCATGTT  1560
Sbjct  1566  ...............................................G............  1625

Query  1561  GAGGTCGAAGTGGAAGAACTCGACCAGCGTGCAGGGGAAGGGGTAGTGGAAACACCTCGA  1620
Sbjct  1626  ............................................................  1685

Query  1621  AACTCTATCAAAGTTTCAACACAGATCGGGGACGCGTTAATCGGCAGTTACCTGATCCTA  1680
Sbjct  1686  ........T...................................................  1745

Query  1681  TCACCCCAAGCAGTCCTACGCAGCGAAAAATTAGCCTGCATACATGATCTTGCAGAGCAG  1740
Sbjct  1746  ............................................................  1805
```

```
Query  1741  GTTAAGTTGGTCACACACTCTGGCCGTAGTGGTAGGTACGCCGTCGACAAATACNACGGA  1800
Sbjct  1806  ..C.................C..T..............................G.....  1865

Query  1801  AGAGTACTAGTCCCTACAGGAGTGGCTATAGACATTCAATCGTTCCAGGCTCTCAGTGAG  1860
Sbjct  1866  ............................................T...............  1925

Query  1861  AGCGCGACCCTTGTGTACAACGAACGCGAGTTCGTTAACAGGAAGCTGTGGCACATAGCA  1920
Sbjct  1926  ..T..........................................T..............  1985

Query  1921  GTATACGGGGCAGCACTCAATACTGATGAAGAAGGATACGAGAAGGTCCCGGTAGAGAGA  1980
Sbjct  1986  ............................................................  2045

Query  1981  GCAGAATCAGATTATGTGTTTGATGTAGACCAAAAAATGTGCCTaaaaaaaGAGCAGGCA  2040
Sbjct  2046  .................A............A.............C...............  2105

Query  2041  TCAGGTTGGGTACTCTGTGGCGAACTAGTCAACCCCCCATTCCACGAATTCGCATATGAA  2100
Sbjct  2106  .....A......................................................  2165

Query  2101  GGGCTCCGCACGAGACCGTCAGCACCCTACAAGGTTCATACAGTAGGTGTGTACGGAGTG  2160
Sbjct  2166  ........T...................................................  2225

Query  2161  CCAGGATCAGGCAAATCCGCAATAATCAAGAACACGGTCACCATGTCTGACCTAGTATTG  2220
Sbjct  2226  ............................................................  2285

Query  2221  AGTGGTAAGAAAGAGAACTGCTTAGAAATTATGAACGATGTACTTAAACACAGAGCTCTA  2280
Sbjct  2286  ............................................................  2345

Query  2281  CGTATCACAGCGAAGACCGTAGACTCAGTGTTATTAAACGGCGTGAAACACACGCCTAAC  2340
Sbjct  2346  .....T.............................G........................  2405

Query  2341  ATACTATACATCGACGAAGCGTTCTCATGCCATGCAGGGACTCTGTTGGCCACTATAGCC  2400
Sbjct  2406  ............................................................  2465

Query  2401  ATAGTCAGGCCCAAACAGAAAGTGGTACTGTGCGGAGACCCGAAACAATGCGGATTCTTC  2460
Sbjct  2466  T...........................................................  2525

Query  2461  AATATGATGCAACTGAAAGTTAATTACAATCATGACATCTGCTCAGAAGTCTTCCACAAA  2520
Sbjct  2526  .......................C....................................  2585

Query  2521  AGTATCTCTAGACGGTGCACCCAGGATATCACGGCCATCGTTTCCAAATTACATTACCAG  2580
Sbjct  2586  ............................................T...............  2645

Query  2581  GACCGAATGAGGACCACAAACCCCCGAAAAGGAGACATCATTATAGACACTACCGGCACT  2640
Sbjct  2646  ............................................................  2705

Query  2641  ACCAAACCAGCCAAAACAGATCTGATTCTGACGTGCTTCAGGGGATGGGTGAAACAGTTG  2700
Sbjct  2706  ....................C.......................................  2765

Query  2701  CAGCAAGACTACAGAGGTAACGAAGTAATGACGGCTGCAGCGTCCCAAGGACTGACGAGG  2760
Sbjct  2766  ............................................................  2825

Query  2761  GCCTCCGTATATGCGGTTCGAACTAAAGTCAATGAGAACCCGCTATATGCACAGACCTCC  2820
Sbjct  2826  ............................................................  2885
```

```
Query  2821  GAGCACGTGAACGTGTTGTTAACACGCACAGAAAACAAGCTAGTATGGAAGACCTTGTCA  2880
Sbjct  2886  .......................................T....................  2945

Query  2881  ACAGATCCCTGGATTAAAACACTGACTAACCCACCTAGAGGGCACTATACCGCCACCATA  2940
Sbjct  2946  ............................................................  3005

Query  2941  GCAGAATGGGAAGCGGAACACCAGGGTATAATGAAGGCCATACAAGGGTATGCACCGCCC  3000
Sbjct  3006  ..............A.............................................  3065

Query  3001  GTGAACACCTTCATGAACAAAGTAAATGTGTGCTGGGCAAAGACACTTACGCCTGTGCTG  3060
Sbjct  3066  ...............................................C..A.........  3125

Query  3061  GAAACTGCGGGTATCTCCCTGTCAGCAGAAGACTGGTCTGAACTGCTGCCCCCGTTTGCC  3120
Sbjct  3126  ............................................................  3185

Query  3121  CAGGACGTGGCGTACTCACCCGAGGTGGCATTAAACATCATATGCACGAAAATGTATGGG  3180
Sbjct  3186  ...............................................A............  3245

Query  3181  TTTGACTTAGACACTGGTCTTTTTCCAGGCCATCAGTGCCAATGACATACACCAAAGAC  3240
Sbjct  3246  ........G.....C.............A.........A.....................  3305

Query  3241  CATTGGGATAACAGAGTTGGAGGGAAAATGTATGGATTCAGCCAACAAGCATACGATCAG  3300
Sbjct  3306  ............................................................  3365

Query  3301  CTGGCAAGACGACATCCGTACCTTCGAGGTAGAGAGAAATCAGGAATGCAGATCGTAGTC  3360
Sbjct  3366  ...................................A........................  3425

Query  3361  ACTGAAATGCGTATCCAGCGCCCAAGATCGGATGCCAACATCATCCCGATCAACCGCAGG  3420
Sbjct  3426  .....G......................................................  3485

Query  3421  CTCCCTCACTCACTCGTAGCCACACACGAGTATAGGCGAGCTGCACGGGCCGAGGAATTC  3480
Sbjct  3486  ............................................G...............  3545

Query  3481  TTCACCACGACACGAGGGTACACTATGCTGCTGGTCTCTGAGTATAACATGAACTTACCA  3540
Sbjct  3546  ..........T.................................................  3605

Query  3541  AACAAGAAGATCACCTGGCTGGCTCCGATAGGGACGCAGGGGGCCCATCACACCGCCAAC  3600
Sbjct  3606  ............................................................  3665

Query  3601  CTAAACTTGGGGATACCACCTCTGCTGGGCAGTTTTGATGCGGTGGTTGTGAACATGCCG  3660
Sbjct  3666  ............................................................  3725

Query  3661  ACTCCATTCCGGAACCATCACTACCAGCAATGTGAAGACCACGCGATGAAACTCCAGATG  3720
Sbjct  3726  ................................C...........................  3785

Query  3721  CTGGCAGGCGACGCACTGAGGCACATTAAACCTGGCGGATCATTGTGGGTCAAGGCATAC  3780
Sbjct  3786  ............................................................  3845

Query  3781  GGCTACGCAGACCGGCACAGCGAGCACGTGGTCTTGGCATTGGCTAGAAAGTTTAAAAGC  3840
Sbjct  3846  .......................................C....................  3905

Query  3841  TTCAGAGTCACACAACCCTCATGCGTGACTTCCAACACCGAGGTGTTTCTCCACTTCTCA  3900
Sbjct  3906  ...........G........G.....A.................................  3965
```

```
Query  3901  ATTTTTGACAATGGCAAACGCGCGATAGCCCTGCATTCAGCTAATAGGAAGGCTAACAGT  3960
Sbjct  3966  ............................................................  4025

Query  3961  ATCTTCCAAAACACCTTCTTACCGGCGGGCAGTGCACCGGCGTACAGAGTCAAACGTGGA  4020
Sbjct  4026  ..........................A....A............................  4085

Query  4021  GACATTTCGAACGCCCCAGAGGATGCAGTGGTCAATGCAGCAAACCAACAGGGAGTGAAG  4080
Sbjct  4086  ............................................................  4145

Query  4081  GGTGCTGGAGTTTGCGGTGCAATTTACCGTAAGTGGCCGGACGCTTTCGGTGATGTCGCT  4140
Sbjct  4146  ............................................................  4205

Query  4141  ACTCCAACCGGAACAGCAGTTTCGAAATCCGTCCAAGATAAATTGGTGATCCACGCTGTC  4200
Sbjct  4206  ............................................................  4265

Query  4201  GGCCCGAATTTCTCAAAATGTTCAGAAGAGGAAGGGGACAGAGACCTAGCATCTGCTTAC  4260
Sbjct  4266  ............................................................  4325

Query  4261  AGAGCTGCAGCAGAAATAGTGATGGATaaaaaaTTACAACAGTGGCCGTCCCCTTACTC  4320
Sbjct  4326  ..........................A.................................  4385

Query  4321  TCCACCGGCATTTATGCCGGAGGAAAAAACAGAGTAGAACAGTCACTCAACCATCTCTTC  4380
Sbjct  4386  ..............C........G....................................  4445

Query  4381  ACGGCATTCGACAATACTGATGCAGATGTGACCATATATTGCATGGACAAAACATGGGAA  4440
Sbjct  4446  ............................................................  4505

Query  4441  AAGAAGATTAAGGAGGCAATCGATCACCGGACTTCGGTTGAGATGGTGCAGGATGACGTG  4500
Sbjct  4506  ............................................................  4565

Query  4501  CAGTTGGAGGAGGAACTGGTACGAGTACACCCTTTGAGTAGTTTAGCAGGTAGGAAGGGT  4560
Sbjct  4566  ............................................................  4625

Query  4561  TACAGTACGGACAGCGGCCGAGTGTTTTCCTACCTGGAAGGTACCAAATTCCATCAGACT  4620
Sbjct  4626  ............................................................  4685

Query  4621  GCGGTGGACATAGCCGAAATGCAAGTGCTGTGGCCCGCCCTCAAAGAGTCTAATGAGCAA  4680
Sbjct  4686  ........T.............................T.....................  4745

Query  4681  ATAGTGGCATACACCTTAGGAGAATCAATGGACCAGATACGTGGCAAGTGCCCGACAGAA  4740
Sbjct  4746  ............................................................  4805

Query  4741  GATACTGACGCCTCCACACCTCCACGGACTGTGCCGTGCCTCTGTCGATACGCCATGACA  4800
Sbjct  4806  ............................................................  4865

Query  4801  CCAGAGAGAGTGTACCGACTTAAATGCACGAACACTACCCAATTTACGGTTTGCTCATCT  4860
Sbjct  4866  ............................................C.....C.........  4925

Query  4861  TTTGAGTTGCCAAAGTATCACATTCAGGGAGTGCAGAGAGTAAAATGTGAAAGAATCATC  4920
Sbjct  4926  ............................................................  4985

Query  4921  ATCTTAGATCCCACTGTTCCACCAACTTACAAACGGCCATGCATCAGACGGTACCCCTCC  4980
Sbjct  4986  ............................................................  5045
```

```
Query  4981  ACAATCTCTTGTAACTCCTCTGAGGACTCCAGGAGCTTGTCTACTTTTTCTGTCAGCTCC  5040
Sbjct  5046  ........C......................................C............  5105

Query  5041  GACTCCTCGATTGGTTCTCTGCCGGTCGGAGACACGAGACCCATTCCAGCCCCGAGGACC  5100
Sbjct  5106  .................CG..A...................T...........A......  5165

Query  5101  ATTTTCAGACCCGTCCCTGCCCCGAGAGCACCCGTGCTCAGAACCACACCGCCTCCTAAA  5160
Sbjct  5166  G..........................................T................  5225

Query  5161  CCACCGCGCACATTCACCGTGCGTGCAGAAGTGCACCAAGCACCCCCTACACCTGTACCT  5220
Sbjct  5226  ...................................T........................  5285

Query  5221  CCACCCAGACCGAAGAGGGCTGCAAAGTTGGCTCGTGAGATGCACCCCGGGTTCACCTTC  5280
Sbjct  5286  ............................................................  5345

Query  5281  GGGGACTTCGGAGAGCACGAGGTTGAGGAGCTTACGGCCTCTCCCTTAACCTTCGGAGAT  5340
Sbjct  5346  ..A..................A.................G....................  5405

Query  5341  TTTGCTGAAGGAGAGATCCAGGGGATGGGAGTGGAGTTTGAATGACTAGGCAGAGCCGGC  5400
Sbjct  5406  ............................................G...............  5465

Query  5401  GGGTACATTTTTTCGTCAGACACGGGTCCAGGCCACCTACAGCAGAGATCCGTTTTACAA  5460
Sbjct  5466  .........................................A..................  5525

Query  5461  AATTGCACGGCAGAATGTATCTACGAACCGGCAAAACTAGAAAAAATTCATGCACCAAAG  5520
Sbjct  5526  ............................................................  5585

Query  5521  TTGGATAAAACCAAGGAAGATATCTTAAGGAGCAAGTACCAAATGAAACCGTCTGAAGCA  5580
Sbjct  5586  ............................................................  5645

Query  5581  AACAAAAGCAGGTACCAATCTAGAAAAGTAGAAAATATGAAAGCAGAGATCGTAGGTAGA  5640
Sbjct  5646  ........T.........................................T.........  5705

Query  5641  CTCTTGGACGGACTGGGGGAGTATCTGGGCACCGAGCATCCAGTTGAATGCTACCGAATA  5700
Sbjct  5706  ..............A.............................................  5765

Query  5701  ACGTACCCGGTGCCTATATACTCAACTAGTGACCTCAGAGGTCTGTCTAGTGCCAAAACA  5760
Sbjct  5766  .....T........................AT..G.........................  5825

Query  5761  GCTGTTAGAGCTTGCAATGCATTTTTGGAAGCTAATTTTCCATCAGTCACTTCATATAAA  5820
Sbjct  5826  ............................................................  5885

Query  5821  ATTACTGATGAATACGACGCATACCTAGATATGGTAGATGGATCAGAGAGCTGTCTGGAC  5880
Sbjct  5886  ............................................................  5945

Query  5881  AGATCCTCCTTTTCGCCGTCTAGATTGCGTAGCTTTCCAAAAACACACTCATACTTGGAC  5940
Sbjct  5946  .................A...........C..............................  6005

Query  5941  CCACAGATCAACAGTGCGGTACCGTCACCATTCCAAAACACCTTACAAAATGTATTGGCA  6000
Sbjct  6006  ..G.........................................................  6065

Query  6001  GCGGCCACCAAAAGAAACTGTAATGTCACACAGATGAGAGAACTACCAACATATGATTCT  6060
Sbjct  6066  ...............................................G............  6125
```

```
Query  6061  GCAGTGCTAAATGTAGAGGCCTTCAGGAAATATGCGTGCAAGCCAGACGTATGGGATGAG  6120
Sbjct  6126  ............................................................  6185

Query  6121  TACAGGGATAATCCGATTTGCATAACCACCGAAAATGTCACCACTTACGTCGCCAAGTTG  6180
Sbjct  6186  ..............A......................................T......  6245

Query  6181  AAAGGACCGAAAGCTGCGGCCTTGTTTGCAAAAACACATAACCTGATACCACTACACCAA  6240
Sbjct  6246  ............................................................  6305

Query  6241  GTTCCTATGGACAAATTCACGGTAGATATGAAGAGAGATGTCAAAGTCACGCCCGGAACC  6300
Sbjct  6306  ............................................................  6365

Query  6301  AAGCACACCGAAGAGAGACCAAAGGTACAGGTGATTCAAGCGGCAGAGCCACTAGCCACT  6360
Sbjct  6366  ............................................................  6425

Query  6361  GCCTACCTCTGCGGAATTCACCGTGAATTGGTGCGCCGTCTCAACAACGCGCTTTTCCCA  6420
Sbjct  6426  .....................................................C......  6485

Query  6421  AACATCCACACTTTGTTTGATATGTCCGCAGAGGATTTCGATGCAATCATAGCGGAACAT  6480
Sbjct  6486  ..T...................................T.....................  6545

Query  6481  TTTAAGCACGGTGACCATGTGTTGGAAACGGATATAGCCTCTTTTGACAAAAGTCAAGAT  6540
Sbjct  6546  .................C..........................................  6605

Query  6541  GATTCCATGGCACTCACTGCGTTAATGATCCTTGAGGACCTGGGAGTAGACCAAAACCTA  6600
Sbjct  6606  ............................................................  6665

Query  6601  ATGAATTTGATAGAGGCTGCATTCGGGGAAATCGTGAGTACACACTTGCCCACAGGTACT  6660
Sbjct  6666  ............................................................  6725

Query  6661  AGATTCAAATTTGGAGCTATGATGAAGTCTGGAATGTTTTTGACGCTGTTCGTCAATACA  6720
Sbjct  6726  .......G.....................C...............T..............  6785

Query  6721  ATTCTTAATGTGGTTATTGCGTGCCGAGTGTTGGAGGATCAATTGGCGCAGTCGCCGTGG  6780
Sbjct  6786  ........C..................................................C  6845

Query  6781  CCTGCTTTCATAGGAGATGACAACATAATCCATGGTATAATATCAGACAAATTGATGGCA  6840
Sbjct  6846  G..........................................................G  6905

Query  6841  GATAGATGTGCCACCTGGATGAACATGGAGGTCAAGATACTGGACTCTATAGTTGGAATA  6900
Sbjct  6906  ............................................T..C............  6965

Query  6901  CGGCCACCTTACTTCTGTGGAGGATTTATTGTATGTGACGATGTAACAGGTACAGCCTGC  6960
Sbjct  6966  ...........T................................................  7025

Query  6961  CGCGTCGCAGACCCACTGAAGAGATTGTTCAAGCTAGGTAAGCCATTGCCACTTGACGAT  7020
Sbjct  7026  ........................C...................................  7085

Query  7021  GGCCAAGATGAAGACAGAAGACGTGCATTACATGATGAAGTGAAAACCTGGTCGCGCGTA  7080
Sbjct  7086  ............................................................  7145

Query  7081  GGGCTGCGACACAGAGTGTGTGAAGCCATCGAAGACCGTTATGCCGTCCACTCATCAGAA  7140
Sbjct  7146  ..................................................T.........  7205
```

```
Query  7141  CTAGTTTTATTGGCACTGACTACTCTGTCTAAGAACTTGAAGTCCTTCAGAAACATAAGA  7200
Sbjct  7206  ............................................................  7265

Query  7201  GGGAAACCAATACATCTCTACGGTGGTCCTAAATAG  7236
Sbjct  7266  ....................................  7301
```

>Barmah Forest virus isolate SW35221, complete genome
Sequence ID: MN689035.1 Length: 11498
Range 1: 54 to 7289

Score:12761 bits(6910), Expect:0.0,
Identities:7127/7236(98%),  Gaps:0/7236(0%), Strand: Plus/Plus

```
Query  1     ATGGCGAAACCAGTTGTGAAGATCGACGTGGAACCTGAAAGCCATTTCGCTAAGCAGGTC  60
Sbjct  54    ...............................................T............  113

Query  61    CAGAGTTGCTTCCCGCAGTTTGAGATCGAAGCAGTGCAGACCACACCAAACGATCATGCA  120
Sbjct  114   .........................................G..................  173

Query  121   CACGCGAGGGCGTTTTCGCACCTTGCTACGAAGCTCATAGAAATGGAGACAGCAAAAGAT  180
Sbjct  174   .....................................................C......  233

Query  181   CAGATCATCCTCGATATCGGAAGTGCACCCGCGAGGAGACTGTATTCAGAACACAAGTAC  240
Sbjct  234   ............................................................  293

Query  241   CACTGTGTTTGCCCAATGAAGTGCACGGAAGATCCAGAGAGAATGCTAGGATATGCACGT  300
Sbjct  294   ............................................................  353

Query  301   AAGTTGATCGCAGGCTCTGCGAAAGGGAAGGCAGAAAAGTTACGCGATCTCAGGGATGTC  360
Sbjct  354   ............................................................  413

Query  361   TTGGCTACGCCAGACATCGAGACGCAGTCGCTATGTCTCCACACAGACGCATCCTGCAGA  420
Sbjct  414   ............................................................  473

Query  421   TACCGCGGTGATGTTGCCGTGTATCAAGACGTGTATGCCATTGACGCACCTACCACGCTG  480
Sbjct  474   ..................................................C.........  533

Query  481   TACCACCAAGCGTTAAAGGGCGTCAGGACCGCATATTGGATAGGCTTTGATACAACGCCG  540
Sbjct  534   ...........................................................A  593

Query  541   TTCATGTACGATGCACTAGCAGGAGCTTACCCGCTCTACTCCACAAACTGGGCTGATGAG  600
Sbjct  594   .........A...........................................C......  653

Query  601   CAAGTGCTCGAGTCCAGAAACATTGGGCTATGTTCAGACAAAGTTTCTGAAGGGGGAAAG  660
Sbjct  654   ............................................................  713

Query  661   AAAGGGAGATCAATCCTCAGGAAGAAGTTCTTGAAGCAGTCAGACAGAGTCATGTTCTCT  720
Sbjct  714   ............................................................  773

Query  721   GTCGGCTCGACGTTGTATACGGAAAGCCGTAAATTACTGCAAAGTTGGCACCTGCCATCC  780
Sbjct  774   ............................................C...............  833
```

```
Query  781   ACATTCCATCTCAAAGGCAAATCTTCGTTCACGTGCCGCTGCGACACTATCGTCAGCTGC  840
Sbjct  834   ................................A..............C............  893

Query  841   GAAGGGTATGTTCTGAAGAAAATTACAATGTGTCCTGGAGTGACAGGCAAACCGATAGGA  900
Sbjct  894   ................................C..C........................  953

Query  901   TATGCCGTCACCCATCACAAAGAAGGATTCGTAGTCGGAAAAGTCACAGATACCATTCGC  960
Sbjct  954   ....................G.............................C.........  1013

Query  961   GGCGAGAGAGTCTCCTTCGCCGTGTGTACTTATGTACCAACAACACTCTGCGACCAGATG  1020
Sbjct  1014  ............................................................  1073

Query  1021  ACCGGGATCCTAGCAACAGAAGTAACAGCCGATGATGCCCAGAAACTGCTGGTGGGTTTG  1080
Sbjct  1074  ........T...................................................  1133

Query  1081  AACCAGAGAATAGTAGTTAATGGTAGGACCCAGAGAAATACCAATACTATGAAGAACTAC  1140
Sbjct  1134  ............................................C...............  1193

Query  1141  CTGCTACCACTGGTTGCACAAGCGCTAGCAAAATGGGCGAAGGAAGCAAAACAGGATATG  1200
Sbjct  1194  ......................................A.....................  1253

Query  1201  GAAGATGAAAGACCCCTGAACGAACGCCAACGAACGCTAACGTGCCTCTGCTGCTGGGCA  1260
Sbjct  1254  ............................................................  1313

Query  1261  TTTAAGCGAAACAAACGCCACGCCATTTACAAGAGACCAGACACACAGAGTATAGTCAAG  1320
Sbjct  1314  ............................................................  1373

Query  1321  GTCCCTTGCGAATTCACAAGCTTTCCTTTGGTCAGCCTGTGGTCCGCTGGGATGTCTATA  1380
Sbjct  1374  ............................................................  1433

Query  1381  TCTCTTAGGCAGAAGTTGAAGATGATGCTGCAGGCGAGGCAGCCCACACAAATAGCAGCA  1440
Sbjct  1434  ............................................................  1493

Query  1441  GTGACTGAGGAACTCATACAAGAAGCAGCTGCAGTAGAGCAAGAGGCCGTGGATACGGCC  1500
Sbjct  1494  ..................................C.........................  1553

Query  1501  AATGCCGAGCTGGACCACGCCGCATGGCCCTCCATTGTGGATACGACAGAGCGCCATGTT  1560
Sbjct  1554  ...............................................G............  1613

Query  1561  GAGGTCGAAGTGGAAGAACTCGACCAGCGTGCAGGGGAAGGGGTAGTGGAAACACCTCGA  1620
Sbjct  1614  ............................................................  1673

Query  1621  AACTCTATCAAAGTTTCAACACAGATCGGGGACGCGTTAATCGGCAGTTACCTGATCCTA  1680
Sbjct  1674  ........T...................................................  1733

Query  1681  TCACCCCAAGCAGTCCTACGCAGCGAAAAATTAGCCTGCATACATGATCTTGCAGAGCAG  1740
Sbjct  1734  ............................................................  1793

Query  1741  GTTAAGTTGGTCACACACTCTGGCCGTAGTGGTAGGTACGCCGTCGACAAATACNACGGA  1800
Sbjct  1794  ..C.................C..T..............................G.....  1853

Query  1801  AGAGTACTAGTCCCTACAGGAGTGGCTATAGACATTCAATCGTTCCAGGCTCTCAGTGAG  1860
Sbjct  1854  ............................................................  1913
```

```
Query  1861  AGCGCGACCCTTGTGTACAACGAACGCGAGTTCGTTAACAGGAAGCTGTGGCACATAGCA  1920
Sbjct  1914  ..T..........................................T..............  1973

Query  1921  GTATACGGGGCAGCACTCAATACTGATGAAGAAGGATACGAGAAGGTCCCGGTAGAGAGA  1980
Sbjct  1974  ............................................................  2033

Query  1981  GCAGAATCAGATTATGTGTTTGATGTAGACCAAAAAATGTGCCTaaaaaaaGAGCAGGCA  2040
Sbjct  2034  .................A............A.............C...............  2093

Query  2041  TCAGGTTGGGTACTCTGTGGCGAACTAGTCAACCCCCCATTCCACGAATTCGCATATGAA  2100
Sbjct  2094  .....A......................................................  2153

Query  2101  GGGCTCCGCACGAGACCGTCAGCACCCTACAAGGTTCATACAGTAGGTGTGTACGGAGTG  2160
Sbjct  2154  ........T...................................................  2213

Query  2161  CCAGGATCAGGCAAATCCGCAATAATCAAGAACACGGTCACCATGTCTGACCTAGTATTG  2220
Sbjct  2214  ............................................................  2273

Query  2221  AGTGGTAAGAAAGAGAACTGCTTAGAAATTATGAACGATGTACTTAAACACAGAGCTCTA  2280
Sbjct  2274  ............................................................  2333

Query  2281  CGTATCACAGCGAAGACCGTAGACTCAGTGTTATTAAACGGCGTGAAACACACGCCTAAC  2340
Sbjct  2334  .....T.............................G........................  2393

Query  2341  ATACTATACATCGACGAAGCGTTCTCATGCCATGCAGGGACTCTGTTGGCCACTATAGCC  2400
Sbjct  2394  ............................................................  2453

Query  2401  ATAGTCAGGCCCAAACAGAAAGTGGTACTGTGCGGAGACCCGAAACAATGCGGATTCTTC  2460
Sbjct  2454  T...........................................................  2513

Query  2461  AATATGATGCAACTGAAAGTTAATTACAATCATGACATCTGCTCAGAAGTCTTCCACAAA  2520
Sbjct  2514  .......................C....................................  2573

Query  2521  AGTATCTCTAGACGGTGCACCCAGGATATCACGGCCATCGTTTCCAAATTACATTACCAG  2580
Sbjct  2574  ............................................T...............  2633

Query  2581  GACCGAATGAGGACCACAAACCCCCGAAAAGGAGACATCATTATAGACACTACCGGCACT  2640
Sbjct  2634  ............................................................  2693

Query  2641  ACCAAACCAGCCAAAACAGATCTGATTCTGACGTGCTTCAGGGGATGGGTGAAACAGTTG  2700
Sbjct  2694  ....................C.......................................  2753

Query  2701  CAGCAAGACTACAGAGGTAACGAAGTAATGACGGCTGCAGCGTCCCAAGGACTGACGAGG  2760
Sbjct  2754  ............................................................  2813

Query  2761  GCCTCCGTATATGCGGTTCGAACTAAAGTCAATGAGAACCCGCTATATGCACAGACCTCC  2820
Sbjct  2814  ............................................................  2873

Query  2821  GAGCACGTGAACGTGTTGTTAACACGCACAGAAAACAAGCTAGTATGGAAGACCTTGTCA  2880
Sbjct  2874  .......................................T....................  2933

Query  2881  ACAGATCCCTGGATTAAAACACTGACTAACCCACCTAGAGGGCACTATACCGCCACCATA  2940
Sbjct  2934  ............................................................  2993
```

```
Query  2941  GCAGAATGGGAAGCGGAACACCAGGGTATAATGAAGGCCATACAAGGGTATGCACCGCCC  3000
Sbjct  2994  ..............A.............................................  3053

Query  3001  GTGAACACCTTCATGAACAAAGTAAATGTGTGCTGGGCAAAGACACTTACGCCTGTGCTG  3060
Sbjct  3054  ...............................................C..A.........  3113

Query  3061  GAAACTGCGGGTATCTCCCTGTCAGCAGAAGACTGGTCTGAACTGCTGCCCCCGTTTGCC  3120
Sbjct  3114  ............................................................  3173

Query  3121  CAGGACGTGGCGTACTCACCCGAGGTGGCATTAAACATCATATGCACGAAAATGTATGGG  3180
Sbjct  3174  ...............................................A............  3233

Query  3181  TTTGACTTAGACACTGGTCTTTTTTCCAGGCCATCAGTGCCAATGACATACACCAAAGAC  3240
Sbjct  3234  ........G.....C.............A.........A.....................  3293

Query  3241  CATTGGGATAACAGAGTTGGAGGGAAAATGTATGGATTCAGCCAACAAGCATACGATCAG  3300
Sbjct  3294  ............................................................  3353

Query  3301  CTGGCAAGACGACATCCGTACCTTCGAGGTAGAGAGAAATCAGGAATGCAGATCGTAGTC  3360
Sbjct  3354  ...................................A........................  3413

Query  3361  ACTGAAATGCGTATCCAGCGCCCAAGATCGGATGCCAACATCATCCCGATCAACCGCAGG  3420
Sbjct  3414  .....G.........................................A............  3473

Query  3421  CTCCCTCACTCACTCGTAGCCACACACGAGTATAGGCGAGCTGCACGGCCGAGGAATTC  3480
Sbjct  3474  ............................................G...............  3533

Query  3481  TTCACCACGACACGAGGGTACACTATGCTGCTGGTCTCTGAGTATAACATGAACTTACCA  3540
Sbjct  3534  ..........T.................................................  3593

Query  3541  AACAAGAAGATCACCTGGCTGGCTCCGATAGGGACGCAGGGGGCCCATCACACCGCCAAC  3600
Sbjct  3594  ............................................................  3653

Query  3601  CTAAACTTGGGGATACCACCTCTGCTGGGCAGTTTTGATGCGGTGGTTGTGAACATGCCG  3660
Sbjct  3654  ............................................................  3713

Query  3661  ACTCCATTCCGGAACCATCACTACCAGCAATGTGAAGACCACGCGATGAAACTCCAGATG  3720
Sbjct  3714  ................................C...........................  3773

Query  3721  CTGGCAGGCGACGCACTGAGGCACATTAAACCTGGCGGATCATTGTGGGTCAAGGCATAC  3780
Sbjct  3774  ............................................................  3833

Query  3781  GGCTACGCAGACCGGCACAGCGAGCACGTGGTCTTGGCATTGGCTAGAAAGTTTAAAAGC  3840
Sbjct  3834  .......................................C....................  3893

Query  3841  TTCAGAGTCACACAACCCTCATGCGTGACTTCCAACACCGAGGTGTTTCTCCACTTCTCA  3900
Sbjct  3894  ...........G........G.....A.................................  3953

Query  3901  ATTTTTGACAATGGCAAACGCGCGATAGCCCTGCATTCAGCTAATAGGAAGGCTAACAGT  3960
Sbjct  3954  ............................................................  4013

Query  3961  ATCTTCCAAAACACCTTCTTACCGGCGGGCAGTGCACCGGCGTACAGAGTCAAACGTGGA  4020
Sbjct  4014  ..........................A....A............................  4073
```

```
Query  4021  GACATTTCGAACGCCCCAGAGGATGCAGTGGTCAATGCAGCAAACCAACAGGGAGTGAAG  4080
Sbjct  4074  ............................................................  4133

Query  4081  GGTGCTGGAGTTTGCGGTGCAATTTACCGTAAGTGGCCGGACGCTTTCGGTGATGTCGCT  4140
Sbjct  4134  ............................................................  4193

Query  4141  ACTCCAACCGGAACAGCAGTTTCGAAATCCGTCCAAGATAAATTGGTGATCCACGCTGTC  4200
Sbjct  4194  ...............................C............................  4253

Query  4201  GGCCCGAATTTCTCAAAATGTTCAGAAGAGGAAGGGGACAGAGACCTAGCATCTGCTTAC  4260
Sbjct  4254  ............................................................  4313

Query  4261  AGAGCTGCAGCAGAAATAGTGATGGATaaaaaaaTTACAACAGTGGCCGTCCCCTTACTC  4320
Sbjct  4314  ..........................A.................................  4373

Query  4321  TCCACCGGCATTTATGCCGGAGGAAAAAACAGAGTAGAACAGTCACTCAACCATCTCTTC  4380
Sbjct  4374  ..............C........G....................................  4433

Query  4381  ACGGCATTCGACAATACTGATGCAGATGTGACCATATATTGCATGGACAAAACATGGGAA  4440
Sbjct  4434  ............................................................  4493

Query  4441  AAGAAGATTAAGGAGGCAATCGATCACCGGACTTCGGTTGAGATGGTGCAGGATGACGTG  4500
Sbjct  4494  ............................................................  4553

Query  4501  CAGTTGGAGGAGGAACTGGTACGAGTACACCCTTTGAGTAGTTTAGCAGGTAGGAAGGGT  4560
Sbjct  4554  ............................................................  4613

Query  4561  TACAGTACGGACAGCGGCCGAGTGTTTTCCTACCTGGAAGGTACCAAATTCCATCAGACT  4620
Sbjct  4614  ............................................................  4673

Query  4621  GCGGTGGACATAGCCGAAATGCAAGTGCTGTGGCCCGCCCTCAAAGAGTCTAATGAGCAA  4680
Sbjct  4674  ........T.............................T.....................  4733

Query  4681  ATAGTGGCATACACCTTAGGAGAATCAATGGACCAGATACGTGGCAAGTGCCCGACAGAA  4740
Sbjct  4734  ............................................................  4793

Query  4741  GATACTGACGCCTCCACACCTCCACGGACTGTGCCGTGCCTCTGTCGATACGCCATGACA  4800
Sbjct  4794  ............................................................  4853

Query  4801  CCAGAGAGAGTGTACCGACTTAAATGCACGAACACTACCCAATTTACGGTTTGCTCATCT  4860
Sbjct  4854  ............................................C.....C.........  4913

Query  4861  TTTGAGTTGCCAAAGTATCACATTCAGGGAGTGCAGAGAGTAAAATGTGAAAGAATCATC  4920
Sbjct  4914  ............................................................  4973

Query  4921  ATCTTAGATCCCACTGTTCCACCAACTTACAAACGGCCATGCATCAGACGGTACCCCTCC  4980
Sbjct  4974  ............................................................  5033

Query  4981  ACAATCTCTTGTAACTCCTCTGAGGACTCCAGGAGCTTGTCTACTTTTTCTGTCAGCTCC  5040
Sbjct  5034  ........C......................................C............  5093

Query  5041  GACTCCTCGATTGGTTCTCTGCCGGTCGGAGACACGAGACCCATTCCAGCCCCGAGGACC  5100
Sbjct  5094  .................CG..A...................T...........A......  5153
```

```
Query  5101  ATTTTCAGACCCGTCCCTGCCCCGAGAGCACCCGTGCTCAGAACCACACCGCCTCCTAAA  5160
Sbjct  5154  G..........................................T................  5213

Query  5161  CCACCGCGCACATTCACCGTGCGTGCAGAAGTGCACCAAGCACCCCCTACACCTGTACCT  5220
Sbjct  5214  ...................................T........................  5273

Query  5221  CCACCCAGACCGAAGAGGGCTGCAAAGTTGGCTCGTGAGATGCACCCCGGGTTCACCTTC  5280
Sbjct  5274  ............................................................  5333

Query  5281  GGGGACTTCGGAGAGCACGAGGTTGAGGAGCTTACGGCCTCTCCCTTAACCTTCGGAGAT  5340
Sbjct  5334  ..A..................A.................G....................  5393

Query  5341  TTTGCTGAAGGAGAGATCCAGGGGATGGGAGTGGAGTTTGAATGACTAGGCAGAGCCGGC  5400
Sbjct  5394  ............................................................  5453

Query  5401  GGGTACATTTTTTCGTCAGACACGGGTCCAGGCCACCTACAGCAGAGATCCGTTTTACAA  5460
Sbjct  5454  .........................................A..................  5513

Query  5461  AATTGCACGGCAGAATGTATCTACGAACCGGCAAAACTAGAAAAAATTCATGCACCAAAG  5520
Sbjct  5514  ............................................................  5573

Query  5521  TTGGATAAAACCAAGGAAGATATCTTAAGGAGCAAGTACCAAATGAAACCGTCTGAAGCA  5580
Sbjct  5574  ............................................................  5633

Query  5581  AACAAAAGCAGGTACCAATCTAGAAAAGTAGAAAATATGAAAGCAGAGATCGTAGGTAGA  5640
Sbjct  5634  ........T.........................................T.........  5693

Query  5641  CTCTTGGACGGACTGGGGGAGTATCTGGGCACCGAGCATCCAGTTGAATGCTACCGAATA  5700
Sbjct  5694  ..............A.............................................  5753

Query  5701  ACGTACCCGGTGCCTATATACTCAACTAGTGACCTCAGAGGTCTGTCTAGTGCCAAAACA  5760
Sbjct  5754  .....T........................AT..G.........................  5813

Query  5761  GCTGTTAGAGCTTGCAATGCATTTTTGGAAGCTAATTTTCCATCAGTCACTTCATATAAA  5820
Sbjct  5814  ............................................................  5873

Query  5821  ATTACTGATGAATACGACGCATACCTAGATATGGTAGATGGATCAGAGAGCTGTCTGGAC  5880
Sbjct  5874  ............................................................  5933

Query  5881  AGATCCTCCTTTTCGCCGTCTAGATTGCGTAGCTTTCCAAAAACACACTCATACTTGGAC  5940
Sbjct  5934  .................A...........C..............................  5993

Query  5941  CCACAGATCAACAGTGCGGTACCGTCACCATTCCAAAACACCTTACAAAATGTATTGGCA  6000
Sbjct  5994  ..G.........................................................  6053

Query  6001  GCGGCCACCAAAAGAAACTGTAATGTCACACAGATGAGAGAACTACCAACATATGATTCT  6060
Sbjct  6054  ...............................................G............  6113

Query  6061  GCAGTGCTAAATGTAGAGGCCTTCAGGAAATATGCGTGCAAGCCAGACGTATGGGATGAG  6120
Sbjct  6114  ............................................................  6173

Query  6121  TACAGGGATAATCCGATTTGCATAACCACCGAAAATGTCACCACTTACGTCGCCAAGTTG  6180
Sbjct  6174  ..............A......................................T......  6233
```

```
Query  6181  AAAGGACCGAAAGCTGCGGCCTTGTTTGCAAAAACACATAACCTGATACCACTACACCAA  6240
Sbjct  6234  ............................................................  6293

Query  6241  GTTCCTATGGACAAATTCACGGTAGATATGAAGAGAGATGTCAAAGTCACGCCCGGAACC  6300
Sbjct  6294  ............................................................  6353

Query  6301  AAGCACACCGAAGAGAGACCAAAGGTACAGGTGATTCAAGCGGCAGAGCCACTAGCCACT  6360
Sbjct  6354  ............................................................  6413

Query  6361  GCCTACCTCTGCGGAATTCACCGTGAATTGGTGCGCCGTCTCAACAACGCGCTTTTCCCA  6420
Sbjct  6414  .....................................................C......  6473

Query  6421  AACATCCACACTTTGTTTGATATGTCCGCAGAGGATTTCGATGCAATCATAGCGGAACAT  6480
Sbjct  6474  ..T...................................T.....................  6533

Query  6481  TTTAAGCACGGTGACCATGTGTTGGAAACGGATATAGCCTCTTTTGACAAAAGTCAAGAT  6540
Sbjct  6534  .................C..........................................  6593

Query  6541  GATTCCATGGCACTCACTGCGTTAATGATCCTTGAGGACCTGGGAGTAGACCAAAACCTA  6600
Sbjct  6594  ............................................................  6653

Query  6601  ATGAATTTGATAGAGGCTGCATTCGGGGAAATCGTGAGTACACACTTGCCCACAGGTACT  6660
Sbjct  6654  ............................................................  6713

Query  6661  AGATTCAAATTTGGAGCTATGATGAAGTCTGGAATGTTTTTGACGCTGTTCGTCAATACA  6720
Sbjct  6714  .......G.....................C...............T..............  6773

Query  6721  ATTCTTAATGTGGTTATTGCGTGCCGAGTGTTGGAGGATCAATTGGCGCAGTCGCCGTGG  6780
Sbjct  6774  ........C..................................................C  6833

Query  6781  CCTGCTTTCATAGGAGATGACAACATAATCCATGGTATAATATCAGACAAATTGATGGCA  6840
Sbjct  6834  G..........................................................G  6893

Query  6841  GATAGATGTGCCACCTGGATGAACATGGAGGTCAAGATACTGGACTCTATAGTTGGAATA  6900
Sbjct  6894  ............................................T..C............  6953

Query  6901  CGGCCACCTTACTTCTGTGGAGGATTTATTGTATGTGACGATGTAACAGGTACAGCCTGC  6960
Sbjct  6954  ...........T................................................  7013

Query  6961  CGCGTCGCAGACCCACTGAAGAGATTGTTCAAGCTAGGTAAGCCATTGCCACTTGACGAT  7020
Sbjct  7014  ........................C...................................  7073

Query  7021  GGCCAAGATGAAGACAGAAGACGTGCATTACATGATGAAGTGAAAACCTGGTCGCGCGTA  7080
Sbjct  7074  ............................................................  7133

Query  7081  GGGCTGCGACACAGAGTGTGTGAAGCCATCGAAGACCGTTATGCCGTCCACTCATCAGAA  7140
Sbjct  7134  ..................................................T.........  7193

Query  7141  CTAGTTTTATTGGCACTGACTACTCTGTCTAAGAACTTGAAGTCCTTCAGAAACATAAGA  7200
Sbjct  7194  ............................................................  7253

Query  7201  GGGAAACCAATACATCTCTACGGTGGTCCTAAATAG  7236
Sbjct  7254  ....................................  7289
```

```
>Barmah Forest virus isolate K60652, complete genome
Sequence ID: MN689026.1 Length: 11454
Range 1: 56 to 7291

Score:12694 bits(6874), Expect:0.0,
Identities:7115/7236(98%),  Gaps:0/7236(0%), Strand: Plus/Plus

Query  1     ATGGCGAAACCAGTTGTGAAGATCGACGTGGAACCTGAAAGCCATTTCGCTAAGCAGGTC  60
Sbjct  56    ...............................................T............  115

Query  61    CAGAGTTGCTTCCCGCAGTTTGAGATCGAAGCAGTGCAGACCACACCAAACGATCATGCA  120
Sbjct  116   .........................................G..................  175

Query  121   CACGCGAGGGCGTTTTCGCACCTTGCTACGAAGCTCATAGAAATGGAGACAGCAAAAGAT  180
Sbjct  176   ............................................................  235

Query  181   CAGATCATCCTCGATATCGGAAGTGCACCCGCGAGGAGACTGTATTCAGAACACAAGTAC  240
Sbjct  236   ..........................G.................................  295

Query  241   CACTGTGTTTGCCCAATGAAGTGCACGGAAGATCCAGAGAGAATGCTAGGATATGCACGT  300
Sbjct  296   ............................................................  355

Query  301   AAGTTGATCGCAGGCTCTGCGAAAGGGAAGGCAGAAAAGTTACGCGATCTCAGGGATGTC  360
Sbjct  356   ............................................................  415

Query  361   TTGGCTACGCCAGACATCGAGACGCAGTCGCTATGTCTCCACACAGACGCATCCTGCAGA  420
Sbjct  416   ............................................................  475

Query  421   TACCGCGGTGATGTTGCCGTGTATCAAGACGTGTATGCCATTGACGCACCTACCACGCTG  480
Sbjct  476   ..................................................C.........  535

Query  481   TACCACCAAGCGTTAAAGGGCGTCAGGACCGCATATTGGATAGGCTTTGATACAACGCCG  540
Sbjct  536   ...........................................................A  595

Query  541   TTCATGTACGATGCACTAGCAGGAGCTTACCCGCTCTACTCCACAAACTGGGCTGATGAG  600
Sbjct  596   .........A..................................G........C......  655

Query  601   CAAGTGCTCGAGTCCAGAAACATTGGGCTATGTTCAGACAAAGTTTCTGAAGGGGGAAAG  660
Sbjct  656   ............................................................  715

Query  661   AAAGGGAGATCAATCCTCAGGAAGAAGTTCTTGAAGCAGTCAGACAGAGTCATGTTCTCT  720
Sbjct  716   ............................................................  775

Query  721   GTCGGCTCGACGTTGTATACGGAAAGCCGTAAATTACTGCAAAGTTGGCACCTGCCATCC  780
Sbjct  776   ............................................C...............  835

Query  781   ACATTCCATCTCAAAGGCAAATCTTCGTTCACGTGCCGCTGCGACACTATCGTCAGCTGC  840
Sbjct  836   ................................A..............C............  895

Query  841   GAAGGGTATGTTCTGAAGAAAATTACAATGTGTCCTGGAGTGACAGGCAAACCGATAGGA  900
```

```
Sbjct  896   ................................C..C........................  955

Query  901   TATGCCGTCACCCATCACAAAGAAGGATTCGTAGTCGGAAAAGTCACAGATACCATTCGC  960
Sbjct  956   ....................G.............................C.........  1015

Query  961   GGCGAGAGAGTCTCCTTCGCCGTGTGTACTTATGTACCAACAACACTCTGCGACCAGATG  1020
Sbjct  1016  ............................................................  1075

Query  1021  ACCGGGATCCTAGCAACAGAAGTAACAGCCGATGATGCCCAGAAACTGCTGGTGGGTTTG  1080
Sbjct  1076  ........T...................................................  1135

Query  1081  AACCAGAGAATAGTAGTTAATGGTAGGACCCAGAGAAATACCAATACTATGAAGAACTAC  1140
Sbjct  1136  ............................................C...............  1195

Query  1141  CTGCTACCACTGGTTGCACAAGCGCTAGCAAAATGGGCGAAGGAAGCAAAACAGGATATG  1200
Sbjct  1196  ..........................T...........A.....................  1255

Query  1201  GAAGATGAAAGACCCCTGAACGAACGCCAACGAACGCTAACGTGCCTCTGCTGCTGGGCA  1260
Sbjct  1256  ............................................................  1315

Query  1261  TTTAAGCGAAACAAACGCCACGCCATTTACAAGAGACCAGACACACAGAGTATAGTCAAG  1320
Sbjct  1316  ............................................................  1375

Query  1321  GTCCCTTGCGAATTCACAAGCTTTCCTTTGGTCAGCCTGTGGTCCGCTGGGATGTCTATA  1380
Sbjct  1376  ............................................................  1435

Query  1381  TCTCTTAGGCAGAAGTTGAAGATGATGCTGCAGGCGAGGCAGCCCACACAAATAGCAGCA  1440
Sbjct  1436  ............................................................  1495

Query  1441  GTGACTGAGGAACTCATACAAGAAGCAGCTGCAGTAGAGCAAGAGGCCGTGGATACGGCC  1500
Sbjct  1496  ..................................C.........................  1555

Query  1501  AATGCCGAGCTGGACCACGCCGCATGGCCCTCCATTGTGGATACGACAGAGCGCCATGTT  1560
Sbjct  1556  ...............................................G............  1615

Query  1561  GAGGTCGAAGTGGAAGAACTCGACCAGCGTGCAGGGGAAGGGGTAGTGGAAACACCTCGA  1620
Sbjct  1616  ............................................................  1675

Query  1621  AACTCTATCAAAGTTTCAACACAGATCGGGGACGCGTTAATCGGCAGTTACCTGATCCTA  1680
Sbjct  1676  ........T...................................................  1735

Query  1681  TCACCCCAAGCAGTCCTACGCAGCGAAAAATTAGCCTGCATACATGATCTTGCAGAGCAG  1740
Sbjct  1736  ............................................................  1795

Query  1741  GTTAAGTTGGTCACACACTCTGGCCGTAGTGGTAGGTACGCCGTCGACAAATACNACGGA  1800
Sbjct  1796  ..C.................C..T..............................G.....  1855

Query  1801  AGAGTACTAGTCCCTACAGGAGTGGCTATAGACATTCAATCGTTCCAGGCTCTCAGTGAG  1860
Sbjct  1856  ...................................C........................  1915

Query  1861  AGCGCGACCCTTGTGTACAACGAACGCGAGTTCGTTAACAGGAAGCTGTGGCACATAGCA  1920
Sbjct  1916  ..T..........................................T..............  1975

Query  1921  GTATACGGGGCAGCACTCAATACTGATGAAGAAGGATACGAGAAGGTCCCGGTAGAGAGA  1980
```

```
Sbjct  1976  ............................................................  2035

Query  1981  GCAGAATCAGATTATGTGTTTGATGTAGACCAAAAAATGTGCCTaaaaaaaGAGCAGGCA  2040
Sbjct  2036  .................A............A.............C...............  2095

Query  2041  TCAGGTTGGGTACTCTGTGGCGAACTAGTCAACCCCCCATTCCACGAATTCGCATATGAA  2100
Sbjct  2096  .....A......................................................  2155

Query  2101  GGGCTCCGCACGAGACCGTCAGCACCCTACAAGGTTCATACAGTAGGTGTGTACGGAGTG  2160
Sbjct  2156  ........T..T................................................  2215

Query  2161  CCAGGATCAGGCAAATCCGCAATAATCAAGAACACGGTCACCATGTCTGACCTAGTATTG  2220
Sbjct  2216  ............................................................  2275

Query  2221  AGTGGTAAGAAAGAGAACTGCTTAGAAATTATGAACGATGTACTTAAACACAGAGCTCTA  2280
Sbjct  2276  ............................................................  2335

Query  2281  CGTATCACAGCGAAGACCGTAGACTCAGTGTTATTAAACGGCGTGAAACACACGCCTAAC  2340
Sbjct  2336  .....T.............................G........................  2395

Query  2341  ATACTATACATCGACGAAGCGTTCTCATGCCATGCAGGGACTCTGTTGGCCACTATAGCC  2400
Sbjct  2396  ............................................................  2455

Query  2401  ATAGTCAGGCCCAAACAGAAAGTGGTACTGTGCGGAGACCCGAAACAATGCGGATTCTTC  2460
Sbjct  2456  T...........................................................  2515

Query  2461  AATATGATGCAACTGAAAGTTAATTACAATCATGACATCTGCTCAGAAGTCTTCCACAAA  2520
Sbjct  2516  .......................C....................................  2575

Query  2521  AGTATCTCTAGACGGTGCACCCAGGATATCACGGCCATCGTTTCCAAATTACATTACCAG  2580
Sbjct  2576  ............................................T...............  2635

Query  2581  GACCGAATGAGGACCACAAACCCCCGAAAAGGAGACATCATTATAGACACTACCGGCACT  2640
Sbjct  2636  ..................................................C.........  2695

Query  2641  ACCAAACCAGCCAAAACAGATCTGATTCTGACGTGCTTCAGGGGATGGGTGAAACAGTTG  2700
Sbjct  2696  ....................C.......................................  2755

Query  2701  CAGCAAGACTACAGAGGTAACGAAGTAATGACGGCTGCAGCGTCCCAAGGACTGACGAGG  2760
Sbjct  2756  ............................................................  2815

Query  2761  GCCTCCGTATATGCGGTTCGAACTAAAGTCAATGAGAACCCGCTATATGCACAGACCTCC  2820
Sbjct  2816  ............................................................  2875

Query  2821  GAGCACGTGAACGTGTTGTTAACACGCACAGAAAACAAGCTAGTATGGAAGACCTTGTCA  2880
Sbjct  2876  .......................................T....................  2935

Query  2881  ACAGATCCCTGGATTAAAACACTGACTAACCCACCTAGAGGGCACTATACCGCCACCATA  2940
Sbjct  2936  ............................................................  2995

Query  2941  GCAGAATGGGAAGCGGAACACCAGGGTATAATGAAGGCCATACAAGGGTATGCACCGCCC  3000
Sbjct  2996  ..............A.............................................  3055

Query  3001  GTGAACACCTTCATGAACAAAGTAAATGTGTGCTGGGCAAAGACACTTACGCCTGTGCTG  3060
```

```
Sbjct  3056  ...............................................C..A.........  3115

Query  3061  GAAACTGCGGGTATCTCCCTGTCAGCAGAAGACTGGTCTGAACTGCTGCCCCCGTTTGCC  3120
Sbjct  3116  ............................................................  3175

Query  3121  CAGGACGTGGCGTACTCACCCGAGGTGGCATTAAACATCATATGCACGAAAATGTATGGG  3180
Sbjct  3176  ...............................................A............  3235

Query  3181  TTTGACTTAGACACTGGTCTTTTTTCCAGGCCATCAGTGCCAATGACATACACCAAAGAC  3240
Sbjct  3236  ........G.....C.............A.........A.....................  3295

Query  3241  CATTGGGATAACAGAGTTGGAGGGAAAATGTATGGATTCAGCCAACAAGCATACGATCAG  3300
Sbjct  3296  ............................................................  3355

Query  3301  CTGGCAAGACGACATCCGTACCTTCGAGGTAGAGAGAAATCAGGAATGCAGATCGTAGTC  3360
Sbjct  3356  ...................................A........................  3415

Query  3361  ACTGAAATGCGTATCCAGCGCCCAAGATCGGATGCCAACATCATCCCGATCAACCGCAGG  3420
Sbjct  3416  .....G......................................................  3475

Query  3421  CTCCCTCACTCACTCGTAGCCACACACGAGTATAGGCGAGCTGCACGGGCCGAGGAATTC  3480
Sbjct  3476  ............................................G...............  3535

Query  3481  TTCACCACGACACGAGGGTACACTATGCTGCTGGTCTCTGAGTATAACATGAACTTACCA  3540
Sbjct  3536  ..........T.................................................  3595

Query  3541  AACAAGAAGATCACCTGGCTGGCTCCGATAGGGACGCAGGGGGCCCATCACACCGCCAAC  3600
Sbjct  3596  ........................................T...................  3655

Query  3601  CTAAACTTGGGGATACCACCTCTGCTGGGCAGTTTTGATGCGGTGGTTGTGAACATGCCG  3660
Sbjct  3656  ........................................T...................  3715

Query  3661  ACTCCATTCCGGAACCATCACTACCAGCAATGTGAAGACCACGCGATGAAACTCCAGATG  3720
Sbjct  3716  ................................C...........................  3775

Query  3721  CTGGCAGGCGACGCACTGAGGCACATTAAACCTGGCGGATCATTGTGGGTCAAGGCATAC  3780
Sbjct  3776  ............................................................  3835

Query  3781  GGCTACGCAGACCGGCACAGCGAGCACGTGGTCTTGGCATTGGCTAGAAAGTTTAAAAGC  3840
Sbjct  3836  .......................................C....................  3895

Query  3841  TTCAGAGTCACACAACCCTCATGCGTGACTTCCAACACCGAGGTGTTTCTCCACTTCTCA  3900
Sbjct  3896  ...........G........G.....A.................................  3955

Query  3901  ATTTTTGACAATGGCAAACGCGCGATAGCCCTGCATTCAGCTAATAGGAAGGCTAACAGT  3960
Sbjct  3956  ............................................................  4015

Query  3961  ATCTTCCAAAACACCTTCTTACCGGCGGGCAGTGCACCGGCGTACAGAGTCAAACGTGGA  4020
Sbjct  4016  ..........................A....A............................  4075

Query  4021  GACATTTCGAACGCCCCAGAGGATGCAGTGGTCAATGCAGCAAACCAACAGGGAGTGAAG  4080
Sbjct  4076  ............................................................  4135

Query  4081  GGTGCTGGAGTTTGCGGTGCAATTTACCGTAAGTGGCCGGACGCTTTCGGTGATGTCGCT  4140
```

```
Sbjct  4136  ............................................................  4195

Query  4141  ACTCCAACCGGAACAGCAGTTTCGAAATCCGTCCAAGATAAATTGGTGATCCACGCTGTC  4200
Sbjct  4196  ............................................................  4255

Query  4201  GGCCCGAATTTCTCAAAATGTTCAGAAGAGGAAGGGGACAGAGACCTAGCATCTGCTTAC  4260
Sbjct  4256  ............................................................  4315

Query  4261  AGAGCTGCAGCAGAAATAGTGATGGATaaaaaaaTTACAACAGTGGCCGTCCCCTTACTC  4320
Sbjct  4316  ..........................A.................................  4375

Query  4321  TCCACCGGCATTTATGCCGGAGGAAAAAACAGAGTAGAACAGTCACTCAACCATCTCTTC  4380
Sbjct  4376  ..............C........G....................................  4435

Query  4381  ACGGCATTCGACAATACTGATGCAGATGTGACCATATATTGCATGGACAAAACATGGGAA  4440
Sbjct  4436  ........T...................................................  4495

Query  4441  AAGAAGATTAAGGAGGCAATCGATCACCGGACTTCGGTTGAGATGGTGCAGGATGACGTG  4500
Sbjct  4496  ............................................................  4555

Query  4501  CAGTTGGAGGAGGAACTGGTACGAGTACACCCTTTGAGTAGTTTAGCAGGTAGGAAGGGT  4560
Sbjct  4556  ......................................C.....................  4615

Query  4561  TACAGTACGGACAGCGGCCGAGTGTTTTCCTACCTGGAAGGTACCAAATTCCATCAGACT  4620
Sbjct  4616  ............................................................  4675

Query  4621  GCGGTGGACATAGCCGAAATGCAAGTGCTGTGGCCCGCCCTCAAAGAGTCTAATGAGCAA  4680
Sbjct  4676  ........T.............................T.....................  4735

Query  4681  ATAGTGGCATACACCTTAGGAGAATCAATGGACCAGATACGTGGCAAGTGCCCGACAGAA  4740
Sbjct  4736  ............................................................  4795

Query  4741  GATACTGACGCCTCCACACCTCCACGGACTGTGCCGTGCCTCTGTCGATACGCCATGACA  4800
Sbjct  4796  ............................................................  4855

Query  4801  CCAGAGAGAGTGTACCGACTTAAATGCACGAACACTACCCAATTTACGGTTTGCTCATCT  4860
Sbjct  4856  ............................................C.....C.........  4915

Query  4861  TTTGAGTTGCCAAAGTATCACATTCAGGGAGTGCAGAGAGTAAAATGTGAAAGAATCATC  4920
Sbjct  4916  ............................................................  4975

Query  4921  ATCTTAGATCCCACTGTTCCACCAACTTACAAACGGCCATGCATCAGACGGTACCCCTCC  4980
Sbjct  4976  ............................................................  5035

Query  4981  ACAATCTCTTGTAACTCCTCTGAGGACTCCAGGAGCTTGTCTACTTTTTCTGTCAGCTCC  5040
Sbjct  5036  ........C......................................C............  5095

Query  5041  GACTCCTCGATTGGTTCTCTGCCGGTCGGAGACACGAGACCCATTCCAGCCCCGAGGACC  5100
Sbjct  5096  .................CG..A...................T...........A......  5155

Query  5101  ATTTTCAGACCCGTCCCTGCCCCGAGAGCACCCGTGCTCAGAACCACACCGCCTCCTAAA  5160
Sbjct  5156  G..........................................T................  5215

Query  5161  CCACCGCGCACATTCACCGTGCGTGCAGAAGTGCACCAAGCACCCCCTACACCTGTACCT  5220
```

```
Sbjct  5216  ...................................T........................  5275

Query  5221  CCACCCAGACCGAAGAGGGCTGCAAAGTTGGCTCGTGAGATGCACCCCGGGTTCACCTTC  5280
Sbjct  5276  .....T......................................................  5335

Query  5281  GGGGACTTCGGAGAGCACGAGGTTGAGGAGCTTACGGCCTCTCCCTTAACCTTCGGAGAT  5340
Sbjct  5336  ..A..................A.................G....................  5395

Query  5341  TTTGCTGAAGGAGAGATCCAGGGGATGGGAGTGGAGTTTGAATGACTAGGCAGAGCCGGC  5400
Sbjct  5396  ....................A.......................................  5455

Query  5401  GGGTACATTTTTTCGTCAGACACGGGTCCAGGCCACCTACAGCAGAGATCCGTTTTACAA  5460
Sbjct  5456  .........................................A..................  5515

Query  5461  AATTGCACGGCAGAATGTATCTACGAACCGGCAAAACTAGAAAAAATTCATGCACCAAAG  5520
Sbjct  5516  ............................................................  5575

Query  5521  TTGGATAAAACCAAGGAAGATATCTTAAGGAGCAAGTACCAAATGAAACCGTCTGAAGCA  5580
Sbjct  5576  ............................................................  5635

Query  5581  AACAAAAGCAGGTACCAATCTAGAAAAGTAGAAAATATGAAAGCAGAGATCGTAGGTAGA  5640
Sbjct  5636  ........T.........................................T.........  5695

Query  5641  CTCTTGGACGGACTGGGGGAGTATCTGGGCACCGAGCATCCAGTTGAATGCTACCGAATA  5700
Sbjct  5696  ..............A.............................................  5755

Query  5701  ACGTACCCGGTGCCTATATACTCAACTAGTGACCTCAGAGGTCTGTCTAGTGCCAAAACA  5760
Sbjct  5756  .....T....C...................AT..G.........................  5815

Query  5761  GCTGTTAGAGCTTGCAATGCATTTTTGGAAGCTAATTTTCCATCAGTCACTTCATATAAA  5820
Sbjct  5816  ......................................C.....................  5875

Query  5821  ATTACTGATGAATACGACGCATACCTAGATATGGTAGATGGATCAGAGAGCTGTCTGGAC  5880
Sbjct  5876  ............................................................  5935

Query  5881  AGATCCTCCTTTTCGCCGTCTAGATTGCGTAGCTTTCCAAAAACACACTCATACTTGGAC  5940
Sbjct  5936  .................A...........C..............................  5995

Query  5941  CCACAGATCAACAGTGCGGTACCGTCACCATTCCAAAACACCTTACAAAATGTATTGGCA  6000
Sbjct  5996  ..G.........................................................  6055

Query  6001  GCGGCCACCAAAAGAAACTGTAATGTCACACAGATGAGAGAACTACCAACATATGATTCT  6060
Sbjct  6056  ...............................................G............  6115

Query  6061  GCAGTGCTAAATGTAGAGGCCTTCAGGAAATATGCGTGCAAGCCAGACGTATGGGATGAG  6120
Sbjct  6116  ............................................................  6175

Query  6121  TACAGGGATAATCCGATTTGCATAACCACCGAAAATGTCACCACTTACGTCGCCAAGTTG  6180
Sbjct  6176  ..............A...................................T..T......  6235

Query  6181  AAAGGACCGAAAGCTGCGGCCTTGTTTGCAAAAACACATAACCTGATACCACTACACCAA  6240
Sbjct  6236  ............................................................  6295

Query  6241  GTTCCTATGGACAAATTCACGGTAGATATGAAGAGAGATGTCAAAGTCACGCCCGGAACC  6300
```

```
Sbjct  6296  ............................................................  6355

Query  6301  AAGCACACCGAAGAGAGACCAAAGGTACAGGTGATTCAAGCGGCAGAGCCACTAGCCACT  6360
Sbjct  6356  ............................................................  6415

Query  6361  GCCTACCTCTGCGGAATTCACCGTGAATTGGTGCGCCGTCTCAACAACGCGCTTTTCCCA  6420
Sbjct  6416  .....................................................C......  6475

Query  6421  AACATCCACACTTTGTTTGATATGTCCGCAGAGGATTTCGATGCAATCATAGCGGAACAT  6480
Sbjct  6476  ..T...................................T.....................  6535

Query  6481  TTTAAGCACGGTGACCATGTGTTGGAAACGGATATAGCCTCTTTTGACAAAAGTCAAGAT  6540
Sbjct  6536  .................C..........................................  6595

Query  6541  GATTCCATGGCACTCACTGCGTTAATGATCCTTGAGGACCTGGGAGTAGACCAAAACCTA  6600
Sbjct  6596  ............................................................  6655

Query  6601  ATGAATTTGATAGAGGCTGCATTCGGGGAAATCGTGAGTACACACTTGCCCACAGGTACT  6660
Sbjct  6656  ............................................................  6715

Query  6661  AGATTCAAATTTGGAGCTATGATGAAGTCTGGAATGTTTTTGACGCTGTTCGTCAATACA  6720
Sbjct  6716  .......G.....................C...............T..............  6775

Query  6721  ATTCTTAATGTGGTTATTGCGTGCCGAGTGTTGGAGGATCAATTGGCGCAGTCGCCGTGG  6780
Sbjct  6776  ........C..................................................C  6835

Query  6781  CCTGCTTTCATAGGAGATGACAACATAATCCATGGTATAATATCAGACAAATTGATGGCA  6840
Sbjct  6836  G...........................................................  6895

Query  6841  GATAGATGTGCCACCTGGATGAACATGGAGGTCAAGATACTGGACTCTATAGTTGGAATA  6900
Sbjct  6896  ............................................T..C............  6955

Query  6901  CGGCCACCTTACTTCTGTGGAGGATTTATTGTATGTGACGATGTAACAGGTACAGCCTGC  6960
Sbjct  6956  ...........T................................................  7015

Query  6961  CGCGTCGCAGACCCACTGAAGAGATTGTTCAAGCTAGGTAAGCCATTGCCACTTGACGAT  7020
Sbjct  7016  ........................C...................................  7075

Query  7021  GGCCAAGATGAAGACAGAAGACGTGCATTACATGATGAAGTGAAAACCTGGTCGCGCGTA  7080
Sbjct  7076  .............................G..............................  7135

Query  7081  GGGCTGCGACACAGAGTGTGTGAAGCCATCGAAGACCGTTATGCCGTCCACTCATCAGAA  7140
Sbjct  7136  ..................................................T.........  7195

Query  7141  CTAGTTTTATTGGCACTGACTACTCTGTCTAAGAACTTGAAGTCCTTCAGAAACATAAGA  7200
Sbjct  7196  ............................................................  7255

Query  7201  GGGAAACCAATACATCTCTACGGTGGTCCTAAATAG  7236
Sbjct  7256  ....................................  7291
```

>Barmah Forest virus isolate K67171, complete genome

```
Sequence ID: MN689028.1 Length: 11464
Range 1: 51 to 7286

Score:12689 bits(6871), Expect:0.0,
Identities:7114/7236(98%),  Gaps:0/7236(0%), Strand: Plus/Plus

Query  1      ATGGCGAAACCAGTTGTGAAGATCGACGTGGAACCTGAAAGCCATTTCGCTAAGCAGGTC  60
Sbjct  51     ...............................................T............  110

Query  61     CAGAGTTGCTTCCCGCAGTTTGAGATCGAAGCAGTGCAGACCACACCAAACGATCATGCA  120
Sbjct  111    .........................................G..................  170

Query  121    CACGCGAGGGCGTTTTCGCACCTTGCTACGAAGCTCATAGAAATGGAGACAGCAAAAGAT  180
Sbjct  171    ............................................................  230

Query  181    CAGATCATCCTCGATATCGGAAGTGCACCCGCGAGGAGACTGTATTCAGAACACAAGTAC  240
Sbjct  231    ............................................................  290

Query  241    CACTGTGTTTGCCCAATGAAGTGCACGGAAGATCCAGAGAGAATGCTAGGATATGCACGT  300
Sbjct  291    ............................................................  350

Query  301    AAGTTGATCGCAGGCTCTGCGAAAGGGAAGGCAGAAAAGTTACGCGATCTCAGGGATGTC  360
Sbjct  351    ............................................................  410

Query  361    TTGGCTACGCCAGACATCGAGACGCAGTCGCTATGTCTCCACACAGACGCATCCTGCAGA  420
Sbjct  411    ............................................................  470

Query  421    TACCGCGGTGATGTTGCCGTGTATCAAGACGTGTATGCCATTGACGCACCTACCACGCTG  480
Sbjct  471    ..................................................C.........  530

Query  481    TACCACCAAGCGTTAAAGGGCGTCAGGACCGCATATTGGATAGGCTTTGATACAACGCCG  540
Sbjct  531    ...........................................................A  590

Query  541    TTCATGTACGATGCACTAGCAGGAGCTTACCCGCTCTACTCCACAAACTGGGCTGATGAG  600
Sbjct  591    .........A...........................................C......  650

Query  601    CAAGTGCTCGAGTCCAGAAACATTGGGCTATGTTCAGACAAAGTTTCTGAAGGGGGAAAG  660
Sbjct  651    ............................................................  710

Query  661    AAAGGGAGATCAATCCTCAGGAAGAAGTTCTTGAAGCAGTCAGACAGAGTCATGTTCTCT  720
Sbjct  711    ............................................................  770

Query  721    GTCGGCTCGACGTTGTATACGGAAAGCCGTAAATTACTGCAAAGTTGGCACCTGCCATCC  780
Sbjct  771    ............................................C...............  830

Query  781    ACATTCCATCTCAAAGGCAAATCTTCGTTCACGTGCCGCTGCGACACTATCGTCAGCTGC  840
Sbjct  831    ................................A..............C............  890

Query  841    GAAGGGTATGTTCTGAAGAAAATTACAATGTGTCCTGGAGTGACAGGCAAACCGATAGGA  900
Sbjct  891    ................................C..C........................  950

Query  901    TATGCCGTCACCCATCACAAAGAAGGATTCGTAGTCGGAAAAGTCACAGATACCATTCGC  960
Sbjct  951    ....................G.............................C.........  1010
```

```
Query  961   GGCGAGAGAGTCTCCTTCGCCGTGTGTACTTATGTACCAACAACACTCTGCGACCAGATG  1020
Sbjct  1011  ............................................................  1070

Query  1021  ACCGGGATCCTAGCAACAGAAGTAACAGCCGATGATGCCCAGAAACTGCTGGTGGGTTTG  1080
Sbjct  1071  ........T...................................................  1130

Query  1081  AACCAGAGAATAGTAGTTAATGGTAGGACCCAGAGAAATACCAATACTATGAAGAACTAC  1140
Sbjct  1131  ............................................C...............  1190

Query  1141  CTGCTACCACTGGTTGCACAAGCGCTAGCAAAATGGGCGAAGGAAGCAAAACAGGATATG  1200
Sbjct  1191  ......................................A.....................  1250

Query  1201  GAAGATGAAAGACCCCTGAACGAACGCCAACGAACGCTAACGTGCCTCTGCTGCTGGGCA  1260
Sbjct  1251  ............................................................  1310

Query  1261  TTTAAGCGAAACAAACGCCACGCCATTTACAAGAGACCAGACACACAGAGTATAGTCAAG  1320
Sbjct  1311  ............................................................  1370

Query  1321  GTCCCTTGCGAATTCACAAGCTTTCCTTTGGTCAGCCTGTGGTCCGCTGGGATGTCTATA  1380
Sbjct  1371  ............................................................  1430

Query  1381  TCTCTTAGGCAGAAGTTGAAGATGATGCTGCAGGCGAGGCAGCCCACACAAATAGCAGCA  1440
Sbjct  1431  ............................................................  1490

Query  1441  GTGACTGAGGAACTCATACAAGAAGCAGCTGCAGTAGAGCAAGAGGCCGTGGATACGGCC  1500
Sbjct  1491  ..................................C.........................  1550

Query  1501  AATGCCGAGCTGGACCACGCCGCATGGCCCTCCATTGTGGATACGACAGAGCGCCATGTT  1560
Sbjct  1551  ...............................................G............  1610

Query  1561  GAGGTCGAAGTGGAAGAACTCGACCAGCGTGCAGGGGAAGGGGTAGTGGAAACACCTCGA  1620
Sbjct  1611  ............................................................  1670

Query  1621  AACTCTATCAAAGTTTCAACACAGATCGGGGACGCGTTAATCGGCAGTTACCTGATCCTA  1680
Sbjct  1671  ........T........T..........................................  1730

Query  1681  TCACCCCAAGCAGTCCTACGCAGCGAAAAATTAGCCTGCATACATGATCTTGCAGAGCAG  1740
Sbjct  1731  ............................................................  1790

Query  1741  GTTAAGTTGGTCACACACTCTGGCCGTAGTGGTAGGTACGCCGTCGACAAATACNACGGA  1800
Sbjct  1791  ..C.................C..T..............................G.....  1850

Query  1801  AGAGTACTAGTCCCTACAGGAGTGGCTATAGACATTCAATCGTTCCAGGCTCTCAGTGAG  1860
Sbjct  1851  ...................................C........................  1910

Query  1861  AGCGCGACCCTTGTGTACAACGAACGCGAGTTCGTTAACAGGAAGCTGTGGCACATAGCA  1920
Sbjct  1911  ..T..........................................T..............  1970

Query  1921  GTATACGGGGCAGCACTCAATACTGATGAAGAAGGATACGAGAAGGTCCCGGTAGAGAGA  1980
Sbjct  1971  ............................................................  2030

Query  1981  GCAGAATCAGATTATGTGTTTGATGTAGACCAAAAAATGTGCCTaaaaaaaGAGCAGGCA  2040
Sbjct  2031  .................A............A.............C...............  2090
```

```
Query  2041  TCAGGTTGGGTACTCTGTGGCGAACTAGTCAACCCCCCATTCCACGAATTCGCATATGAA  2100
Sbjct  2091  .....A......................................................  2150

Query  2101  GGGCTCCGCACGAGACCGTCAGCACCCTACAAGGTTCATACAGTAGGTGTGTACGGAGTG  2160
Sbjct  2151  ........T..T................................................  2210

Query  2161  CCAGGATCAGGCAAATCCGCAATAATCAAGAACACGGTCACCATGTCTGACCTAGTATTG  2220
Sbjct  2211  ............................................................  2270

Query  2221  AGTGGTAAGAAAGAGAACTGCTTAGAAATTATGAACGATGTACTTAAACACAGAGCTCTA  2280
Sbjct  2271  ............................................................  2330

Query  2281  CGTATCACAGCGAAGACCGTAGACTCAGTGTTATTAAACGGCGTGAAACACACGCCTAAC  2340
Sbjct  2331  .....T.............................G........................  2390

Query  2341  ATACTATACATCGACGAAGCGTTCTCATGCCATGCAGGGACTCTGTTGGCCACTATAGCC  2400
Sbjct  2391  ............................................................  2450

Query  2401  ATAGTCAGGCCCAAACAGAAAGTGGTACTGTGCGGAGACCCGAAACAATGCGGATTCTTC  2460
Sbjct  2451  T...........................................................  2510

Query  2461  AATATGATGCAACTGAAAGTTAATTACAATCATGACATCTGCTCAGAAGTCTTCCACAAA  2520
Sbjct  2511  .......................C....................................  2570

Query  2521  AGTATCTCTAGACGGTGCACCCAGGATATCACGGCCATCGTTTCCAAATTACATTACCAG  2580
Sbjct  2571  ............................................T...............  2630

Query  2581  GACCGAATGAGGACCACAAACCCCCGAAAAGGAGACATCATTATAGACACTACCGGCACT  2640
Sbjct  2631  ..................................................C.........  2690

Query  2641  ACCAAACCAGCCAAAACAGATCTGATTCTGACGTGCTTCAGGGGATGGGTGAAACAGTTG  2700
Sbjct  2691  ....................C.......................................  2750

Query  2701  CAGCAAGACTACAGAGGTAACGAAGTAATGACGGCTGCAGCGTCCCAAGGACTGACGAGG  2760
Sbjct  2751  ............................................................  2810

Query  2761  GCCTCCGTATATGCGGTTCGAACTAAAGTCAATGAGAACCCGCTATATGCACAGACCTCC  2820
Sbjct  2811  ............................................................  2870

Query  2821  GAGCACGTGAACGTGTTGTTAACACGCACAGAAAACAAGCTAGTATGGAAGACCTTGTCA  2880
Sbjct  2871  .......................................T....................  2930

Query  2881  ACAGATCCCTGGATTAAAACACTGACTAACCCACCTAGAGGGCACTATACCGCCACCATA  2940
Sbjct  2931  ............................................................  2990

Query  2941  GCAGAATGGGAAGCGGAACACCAGGGTATAATGAAGGCCATACAAGGGTATGCACCGCCC  3000
Sbjct  2991  ..............A.............................................  3050

Query  3001  GTGAACACCTTCATGAACAAAGTAAATGTGTGCTGGGCAAAGACACTTACGCCTGTGCTG  3060
Sbjct  3051  ............................................G..C..A.........  3110

Query  3061  GAAACTGCGGGTATCTCCCTGTCAGCAGAAGACTGGTCTGAACTGCTGCCCCCGTTTGCC  3120
Sbjct  3111  ............................................................  3170
```

```
Query  3121  CAGGACGTGGCGTACTCACCCGAGGTGGCATTAAACATCATATGCACGAAAATGTATGGG  3180
Sbjct  3171  ...............................................A............  3230

Query  3181  TTTGACTTAGACACTGGTCTTTTTTCCAGGCCATCAGTGCCAATGACATACACCAAAGAC  3240
Sbjct  3231  ........G.....C.............A.........A.....................  3290

Query  3241  CATTGGGATAACAGAGTTGGAGGGAAAATGTATGGATTCAGCCAACAAGCATACGATCAG  3300
Sbjct  3291  ............................................................  3350

Query  3301  CTGGCAAGACGACATCCGTACCTTCGAGGTAGAGAGAAATCAGGAATGCAGATCGTAGTC  3360
Sbjct  3351  .................A.................A........................  3410

Query  3361  ACTGAAATGCGTATCCAGCGCCCAAGATCGGATGCCAACATCATCCCGATCAACCGCAGG  3420
Sbjct  3411  .....G......................................................  3470

Query  3421  CTCCCTCACTCACTCGTAGCCACACACGAGTATAGGCGAGCTGCACGGGCCGAGGAATTC  3480
Sbjct  3471  ............................................G...............  3530

Query  3481  TTCACCACGACACGAGGGTACACTATGCTGCTGGTCTCTGAGTATAACATGAACTTACCA  3540
Sbjct  3531  ..........T.................................................  3590

Query  3541  AACAAGAAGATCACCTGGCTGGCTCCGATAGGGACGCAGGGGGCCCATCACACCGCCAAC  3600
Sbjct  3591  ............................................................  3650

Query  3601  CTAAACTTGGGGATACCACCTCTGCTGGGCAGTTTTGATGCGGTGGTTGTGAACATGCCG  3660
Sbjct  3651  ........................................T...................  3710

Query  3661  ACTCCATTCCGGAACCATCACTACCAGCAATGTGAAGACCACGCGATGAAACTCCAGATG  3720
Sbjct  3711  ................................C...........................  3770

Query  3721  CTGGCAGGCGACGCACTGAGGCACATTAAACCTGGCGGATCATTGTGGGTCAAGGCATAC  3780
Sbjct  3771  ............................................................  3830

Query  3781  GGCTACGCAGACCGGCACAGCGAGCACGTGGTCTTGGCATTGGCTAGAAAGTTTAAAAGC  3840
Sbjct  3831  .......................................C....................  3890

Query  3841  TTCAGAGTCACACAACCCTCATGCGTGACTTCCAACACCGAGGTGTTTCTCCACTTCTCA  3900
Sbjct  3891  ...........G........G.....A.................................  3950

Query  3901  ATTTTTGACAATGGCAAACGCGCGATAGCCCTGCATTCAGCTAATAGGAAGGCTAACAGT  3960
Sbjct  3951  ............................................................  4010

Query  3961  ATCTTCCAAAACACCTTCTTACCGGCGGGCAGTGCACCGGCGTACAGAGTCAAACGTGGA  4020
Sbjct  4011  ..........................A....A............................  4070

Query  4021  GACATTTCGAACGCCCCAGAGGATGCAGTGGTCAATGCAGCAAACCAACAGGGAGTGAAG  4080
Sbjct  4071  ............................................................  4130

Query  4081  GGTGCTGGAGTTTGCGGTGCAATTTACCGTAAGTGGCCGGACGCTTTCGGTGATGTCGCT  4140
Sbjct  4131  ............................................................  4190

Query  4141  ACTCCAACCGGAACAGCAGTTTCGAAATCCGTCCAAGATAAATTGGTGATCCACGCTGTC  4200
Sbjct  4191  ............................................................  4250
```

```
Query  4201  GGCCCGAATTTCTCAAAATGTTCAGAAGAGGAAGGGGACAGAGACCTAGCATCTGCTTAC  4260
Sbjct  4251  ............................................................  4310

Query  4261  AGAGCTGCAGCAGAAATAGTGATGGATaaaaaaTTACAACAGTGGCCGTCCCCTTACTC  4320
Sbjct  4311  ..........................A.................................  4370

Query  4321  TCCACCGGCATTTATGCCGGAGGAAAAAACAGAGTAGAACAGTCACTCAACCATCTCTTC  4380
Sbjct  4371  ..............C........G....................................  4430

Query  4381  ACGGCATTCGACAATACTGATGCAGATGTGACCATATATTGCATGGACAAAACATGGGAA  4440
Sbjct  4431  ........T...................................................  4490

Query  4441  AAGAAGATTAAGGAGGCAATCGATCACCGGACTTCGGTTGAGATGGTGCAGGATGACGTG  4500
Sbjct  4491  ............................................................  4550

Query  4501  CAGTTGGAGGAGGAACTGGTACGAGTACACCCTTTGAGTAGTTTAGCAGGTAGGAAGGGT  4560
Sbjct  4551  ......................................C.....................  4610

Query  4561  TACAGTACGGACAGCGGCCGAGTGTTTTCCTACCTGGAAGGTACCAAATTCCATCAGACT  4620
Sbjct  4611  ............................................................  4670

Query  4621  GCGGTGGACATAGCCGAAATGCAAGTGCTGTGGCCCGCCCTCAAAGAGTCTAATGAGCAA  4680
Sbjct  4671  ........T.............................T.....................  4730

Query  4681  ATAGTGGCATACACCTTAGGAGAATCAATGGACCAGATACGTGGCAAGTGCCCGACAGAA  4740
Sbjct  4731  ...........T................................................  4790

Query  4741  GATACTGACGCCTCCACACCTCCACGGACTGTGCCGTGCCTCTGTCGATACGCCATGACA  4800
Sbjct  4791  ............................................................  4850

Query  4801  CCAGAGAGAGTGTACCGACTTAAATGCACGAACACTACCCAATTTACGGTTTGCTCATCT  4860
Sbjct  4851  ............................................C.....C.........  4910

Query  4861  TTTGAGTTGCCAAAGTATCACATTCAGGGAGTGCAGAGAGTAAAATGTGAAAGAATCATC  4920
Sbjct  4911  ............................................................  4970

Query  4921  ATCTTAGATCCCACTGTTCCACCAACTTACAAACGGCCATGCATCAGACGGTACCCCTCC  4980
Sbjct  4971  ............................................................  5030

Query  4981  ACAATCTCTTGTAACTCCTCTGAGGACTCCAGGAGCTTGTCTACTTTTTCTGTCAGCTCC  5040
Sbjct  5031  ........C......................................C............  5090

Query  5041  GACTCCTCGATTGGTTCTCTGCCGGTCGGAGACACGAGACCCATTCCAGCCCCGAGGACC  5100
Sbjct  5091  .................CG..A...................T...........A......  5150

Query  5101  ATTTTCAGACCCGTCCCTGCCCCGAGAGCACCCGTGCTCAGAACCACACCGCCTCCTAAA  5160
Sbjct  5151  G...............................T..........T................  5210

Query  5161  CCACCGCGCACATTCACCGTGCGTGCAGAAGTGCACCAAGCACCCCCTACACCTGTACCT  5220
Sbjct  5211  ...................................T........................  5270

Query  5221  CCACCCAGACCGAAGAGGGCTGCAAAGTTGGCTCGTGAGATGCACCCCGGGTTCACCTTC  5280
Sbjct  5271  .....T......................................................  5330
```

```
Query  5281  GGGGACTTCGGAGAGCACGAGGTTGAGGAGCTTACGGCCTCTCCCTTAACCTTCGGAGAT  5340
Sbjct  5331  ..A..................A.................G....................  5390

Query  5341  TTTGCTGAAGGAGAGATCCAGGGGATGGGAGTGGAGTTTGAATGACTAGGCAGAGCCGGC  5400
Sbjct  5391  ....................A.......................................  5450

Query  5401  GGGTACATTTTTTCGTCAGACACGGGTCCAGGCCACCTACAGCAGAGATCCGTTTTACAA  5460
Sbjct  5451  .........................................A..................  5510

Query  5461  AATTGCACGGCAGAATGTATCTACGAACCGGCAAAACTAGAAAAAATTCATGCACCAAAG  5520
Sbjct  5511  ............................................................  5570

Query  5521  TTGGATAAAACCAAGGAAGATATCTTAAGGAGCAAGTACCAAATGAAACCGTCTGAAGCA  5580
Sbjct  5571  ............................................................  5630

Query  5581  AACAAAAGCAGGTACCAATCTAGAAAAGTAGAAAATATGAAAGCAGAGATCGTAGGTAGA  5640
Sbjct  5631  ........T.........................................T.........  5690

Query  5641  CTCTTGGACGGACTGGGGGAGTATCTGGGCACCGAGCATCCAGTTGAATGCTACCGAATA  5700
Sbjct  5691  ..............A.............................................  5750

Query  5701  ACGTACCCGGTGCCTATATACTCAACTAGTGACCTCAGAGGTCTGTCTAGTGCCAAAACA  5760
Sbjct  5751  .....T....C...................AT..G.........................  5810

Query  5761  GCTGTTAGAGCTTGCAATGCATTTTTGGAAGCTAATTTTCCATCAGTCACTTCATATAAA  5820
Sbjct  5811  ............................................................  5870

Query  5821  ATTACTGATGAATACGACGCATACCTAGATATGGTAGATGGATCAGAGAGCTGTCTGGAC  5880
Sbjct  5871  ............................................................  5930

Query  5881  AGATCCTCCTTTTCGCCGTCTAGATTGCGTAGCTTTCCAAAAACACACTCATACTTGGAC  5940
Sbjct  5931  .................A...........C..............................  5990

Query  5941  CCACAGATCAACAGTGCGGTACCGTCACCATTCCAAAACACCTTACAAAATGTATTGGCA  6000
Sbjct  5991  ..G.........................................................  6050

Query  6001  GCGGCCACCAAAAGAAACTGTAATGTCACACAGATGAGAGAACTACCAACATATGATTCT  6060
Sbjct  6051  ...............................................G............  6110

Query  6061  GCAGTGCTAAATGTAGAGGCCTTCAGGAAATATGCGTGCAAGCCAGACGTATGGGATGAG  6120
Sbjct  6111  ............................................................  6170

Query  6121  TACAGGGATAATCCGATTTGCATAACCACCGAAAATGTCACCACTTACGTCGCCAAGTTG  6180
Sbjct  6171  ..............A...................................T..T......  6230

Query  6181  AAAGGACCGAAAGCTGCGGCCTTGTTTGCAAAAACACATAACCTGATACCACTACACCAA  6240
Sbjct  6231  ............................................................  6290

Query  6241  GTTCCTATGGACAAATTCACGGTAGATATGAAGAGAGATGTCAAAGTCACGCCCGGAACC  6300
Sbjct  6291  ............................................................  6350

Query  6301  AAGCACACCGAAGAGAGACCAAAGGTACAGGTGATTCAAGCGGCAGAGCCACTAGCCACT  6360
Sbjct  6351  ............................................................  6410
```

```
Query  6361  GCCTACCTCTGCGGAATTCACCGTGAATTGGTGCGCCGTCTCAACAACGCGCTTTTCCCA  6420
Sbjct  6411  .....................................................C......  6470

Query  6421  AACATCCACACTTTGTTTGATATGTCCGCAGAGGATTTCGATGCAATCATAGCGGAACAT  6480
Sbjct  6471  ..T...................................T.....................  6530

Query  6481  TTTAAGCACGGTGACCATGTGTTGGAAACGGATATAGCCTCTTTTGACAAAAGTCAAGAT  6540
Sbjct  6531  .................C..........................................  6590

Query  6541  GATTCCATGGCACTCACTGCGTTAATGATCCTTGAGGACCTGGGAGTAGACCAAAACCTA  6600
Sbjct  6591  ............................................................  6650

Query  6601  ATGAATTTGATAGAGGCTGCATTCGGGGAAATCGTGAGTACACACTTGCCCACAGGTACT  6660
Sbjct  6651  ............................................................  6710

Query  6661  AGATTCAAATTTGGAGCTATGATGAAGTCTGGAATGTTTTTGACGCTGTTCGTCAATACA  6720
Sbjct  6711  .......G.....................C...............T..............  6770

Query  6721  ATTCTTAATGTGGTTATTGCGTGCCGAGTGTTGGAGGATCAATTGGCGCAGTCGCCGTGG  6780
Sbjct  6771  ........C..................................................C  6830

Query  6781  CCTGCTTTCATAGGAGATGACAACATAATCCATGGTATAATATCAGACAAATTGATGGCA  6840
Sbjct  6831  G..........................................................G  6890

Query  6841  GATAGATGTGCCACCTGGATGAACATGGAGGTCAAGATACTGGACTCTATAGTTGGAATA  6900
Sbjct  6891  ............................................T..C............  6950

Query  6901  CGGCCACCTTACTTCTGTGGAGGATTTATTGTATGTGACGATGTAACAGGTACAGCCTGC  6960
Sbjct  6951  ...........T................................................  7010

Query  6961  CGCGTCGCAGACCCACTGAAGAGATTGTTCAAGCTAGGTAAGCCATTGCCACTTGACGAT  7020
Sbjct  7011  ........................C...................................  7070

Query  7021  GGCCAAGATGAAGACAGAAGACGTGCATTACATGATGAAGTGAAAACCTGGTCGCGCGTA  7080
Sbjct  7071  .............................G..............................  7130

Query  7081  GGGCTGCGACACAGAGTGTGTGAAGCCATCGAAGACCGTTATGCCGTCCACTCATCAGAA  7140
Sbjct  7131  ..................................................T.........  7190

Query  7141  CTAGTTTTATTGGCACTGACTACTCTGTCTAAGAACTTGAAGTCCTTCAGAAACATAAGA  7200
Sbjct  7191  ............................................................  7250

Query  7201  GGGAAACCAATACATCTCTACGGTGGTCCTAAATAG  7236
Sbjct  7251  ....................................  7286
```

>Barmah Forest virus isolate K67289, complete genome
Sequence ID: MN689029.1 Length: 11254
Range 1: 49 to 7284

Score:12683 bits(6868), Expect:0.0,
Identities:7113/7236(98%),  Gaps:0/7236(0%), Strand: Plus/Plus

```
Query  1     ATGGCGAAACCAGTTGTGAAGATCGACGTGGAACCTGAAAGCCATTTCGCTAAGCAGGTC  60
Sbjct  49    ...............................................T............  108

Query  61    CAGAGTTGCTTCCCGCAGTTTGAGATCGAAGCAGTGCAGACCACACCAAACGATCATGCA  120
Sbjct  109   .........................................G..................  168

Query  121   CACGCGAGGGCGTTTTCGCACCTTGCTACGAAGCTCATAGAAATGGAGACAGCAAAAGAT  180
Sbjct  169   ............................................................  228

Query  181   CAGATCATCCTCGATATCGGAAGTGCACCCGCGAGGAGACTGTATTCAGAACACAAGTAC  240
Sbjct  229   ............................................................  288

Query  241   CACTGTGTTTGCCCAATGAAGTGCACGGAAGATCCAGAGAGAATGCTAGGATATGCACGT  300
Sbjct  289   ............................................................  348

Query  301   AAGTTGATCGCAGGCTCTGCGAAAGGGAAGGCAGAAAAGTTACGCGATCTCAGGGATGTC  360
Sbjct  349   ............................................................  408

Query  361   TTGGCTACGCCAGACATCGAGACGCAGTCGCTATGTCTCCACACAGACGCATCCTGCAGA  420
Sbjct  409   ............................................................  468

Query  421   TACCGCGGTGATGTTGCCGTGTATCAAGACGTGTATGCCATTGACGCACCTACCACGCTG  480
Sbjct  469   ..................................................C.........  528

Query  481   TACCACCAAGCGTTAAAGGGCGTCAGGACCGCATATTGGATAGGCTTTGATACAACGCCG  540
Sbjct  529   ...........................................................A  588

Query  541   TTCATGTACGATGCACTAGCAGGAGCTTACCCGCTCTACTCCACAAACTGGGCTGATGAG  600
Sbjct  589   .........A...........................................C......  648

Query  601   CAAGTGCTCGAGTCCAGAAACATTGGGCTATGTTCAGACAAAGTTTCTGAAGGGGGAAAG  660
Sbjct  649   ............................................................  708

Query  661   AAAGGGAGATCAATCCTCAGGAAGAAGTTCTTGAAGCAGTCAGACAGAGTCATGTTCTCT  720
Sbjct  709   ............................................................  768

Query  721   GTCGGCTCGACGTTGTATACGGAAAGCCGTAAATTACTGCAAAGTTGGCACCTGCCATCC  780
Sbjct  769   ............................................C...............  828

Query  781   ACATTCCATCTCAAAGGCAAATCTTCGTTCACGTGCCGCTGCGACACTATCGTCAGCTGC  840
Sbjct  829   ................................A..............C............  888

Query  841   GAAGGGTATGTTCTGAAGAAAATTACAATGTGTCCTGGAGTGACAGGCAAACCGATAGGA  900
Sbjct  889   ........C.......................C..C........................  948

Query  901   TATGCCGTCACCCATCACAAAGAAGGATTCGTAGTCGGAAAAGTCACAGATACCATTCGC  960
Sbjct  949   ....................G.............................C.........  1008

Query  961   GGCGAGAGAGTCTCCTTCGCCGTGTGTACTTATGTACCAACAACACTCTGCGACCAGATG  1020
Sbjct  1009  ............................................................  1068

Query  1021  ACCGGGATCCTAGCAACAGAAGTAACAGCCGATGATGCCCAGAAACTGCTGGTGGGTTTG  1080
Sbjct  1069  ........T...................................................  1128
```

```
Query  1081  AACCAGAGAATAGTAGTTAATGGTAGGACCCAGAGAAATACCAATACTATGAAGAACTAC  1140
Sbjct  1129  ............................................C...............  1188

Query  1141  CTGCTACCACTGGTTGCACAAGCGCTAGCAAAATGGGCGAAGGAAGCAAAACAGGATATG  1200
Sbjct  1189  ......................................A.....................  1248

Query  1201  GAAGATGAAAGACCCCTGAACGAACGCCAACGAACGCTAACGTGCCTCTGCTGCTGGGCA  1260
Sbjct  1249  ............................................................  1308

Query  1261  TTTAAGCGAAACAAACGCCACGCCATTTACAAGAGACCAGACACACAGAGTATAGTCAAG  1320
Sbjct  1309  ............................................................  1368

Query  1321  GTCCCTTGCGAATTCACAAGCTTTCCTTTGGTCAGCCTGTGGTCCGCTGGGATGTCTATA  1380
Sbjct  1369  ............................................................  1428

Query  1381  TCTCTTAGGCAGAAGTTGAAGATGATGCTGCAGGCGAGGCAGCCCACACAAATAGCAGCA  1440
Sbjct  1429  ............................................................  1488

Query  1441  GTGACTGAGGAACTCATACAAGAAGCAGCTGCAGTAGAGCAAGAGGCCGTGGATACGGCC  1500
Sbjct  1489  ..................................C.........................  1548

Query  1501  AATGCCGAGCTGGACCACGCCGCATGGCCCTCCATTGTGGATACGACAGAGCGCCATGTT  1560
Sbjct  1549  ...............................................G............  1608

Query  1561  GAGGTCGAAGTGGAAGAACTCGACCAGCGTGCAGGGGAAGGGGTAGTGGAAACACCTCGA  1620
Sbjct  1609  ............................................................  1668

Query  1621  AACTCTATCAAAGTTTCAACACAGATCGGGGACGCGTTAATCGGCAGTTACCTGATCCTA  1680
Sbjct  1669  ........T........T..........................................  1728

Query  1681  TCACCCCAAGCAGTCCTACGCAGCGAAAAATTAGCCTGCATACATGATCTTGCAGAGCAG  1740
Sbjct  1729  ............................................................  1788

Query  1741  GTTAAGTTGGTCACACACTCTGGCCGTAGTGGTAGGTACGCCGTCGACAAATACNACGGA  1800
Sbjct  1789  ..C.................C..T..............................G.....  1848

Query  1801  AGAGTACTAGTCCCTACAGGAGTGGCTATAGACATTCAATCGTTCCAGGCTCTCAGTGAG  1860
Sbjct  1849  ...................................C........................  1908

Query  1861  AGCGCGACCCTTGTGTACAACGAACGCGAGTTCGTTAACAGGAAGCTGTGGCACATAGCA  1920
Sbjct  1909  ..T..........................................T..............  1968

Query  1921  GTATACGGGGCAGCACTCAATACTGATGAAGAAGGATACGAGAAGGTCCCGGTAGAGAGA  1980
Sbjct  1969  ............................................................  2028

Query  1981  GCAGAATCAGATTATGTGTTTGATGTAGACCAAAAAATGTGCCTaaaaaaaGAGCAGGCA  2040
Sbjct  2029  .................A............A.............C...............  2088

Query  2041  TCAGGTTGGGTACTCTGTGGCGAACTAGTCAACCCCCCATTCCACGAATTCGCATATGAA  2100
Sbjct  2089  .....A......................................................  2148

Query  2101  GGGCTCCGCACGAGACCGTCAGCACCCTACAAGGTTCATACAGTAGGTGTGTACGGAGTG  2160
Sbjct  2149  ........T..T................................................  2208
```

```
Query  2161  CCAGGATCAGGCAAATCCGCAATAATCAAGAACACGGTCACCATGTCTGACCTAGTATTG  2220
Sbjct  2209  ............................................................  2268

Query  2221  AGTGGTAAGAAAGAGAACTGCTTAGAAATTATGAACGATGTACTTAAACACAGAGCTCTA  2280
Sbjct  2269  ............................................................  2328

Query  2281  CGTATCACAGCGAAGACCGTAGACTCAGTGTTATTAAACGGCGTGAAACACACGCCTAAC  2340
Sbjct  2329  .....T.............................G........................  2388

Query  2341  ATACTATACATCGACGAAGCGTTCTCATGCCATGCAGGGACTCTGTTGGCCACTATAGCC  2400
Sbjct  2389  ............................................................  2448

Query  2401  ATAGTCAGGCCCAAACAGAAAGTGGTACTGTGCGGAGACCCGAAACAATGCGGATTCTTC  2460
Sbjct  2449  T...........................................................  2508

Query  2461  AATATGATGCAACTGAAAGTTAATTACAATCATGACATCTGCTCAGAAGTCTTCCACAAA  2520
Sbjct  2509  .......................C....................................  2568

Query  2521  AGTATCTCTAGACGGTGCACCCAGGATATCACGGCCATCGTTTCCAAATTACATTACCAG  2580
Sbjct  2569  ............................................T...............  2628

Query  2581  GACCGAATGAGGACCACAAACCCCCGAAAAGGAGACATCATTATAGACACTACCGGCACT  2640
Sbjct  2629  ..................................................C.........  2688

Query  2641  ACCAAACCAGCCAAAACAGATCTGATTCTGACGTGCTTCAGGGGATGGGTGAAACAGTTG  2700
Sbjct  2689  ....................C.......................................  2748

Query  2701  CAGCAAGACTACAGAGGTAACGAAGTAATGACGGCTGCAGCGTCCCAAGGACTGACGAGG  2760
Sbjct  2749  ............................................................  2808

Query  2761  GCCTCCGTATATGCGGTTCGAACTAAAGTCAATGAGAACCCGCTATATGCACAGACCTCC  2820
Sbjct  2809  ............................................................  2868

Query  2821  GAGCACGTGAACGTGTTGTTAACACGCACAGAAAACAAGCTAGTATGGAAGACCTTGTCA  2880
Sbjct  2869  .......................................T....................  2928

Query  2881  ACAGATCCCTGGATTAAAACACTGACTAACCCACCTAGAGGGCACTATACCGCCACCATA  2940
Sbjct  2929  ............................................................  2988

Query  2941  GCAGAATGGGAAGCGGAACACCAGGGTATAATGAAGGCCATACAAGGGTATGCACCGCCC  3000
Sbjct  2989  ..............A.............................................  3048

Query  3001  GTGAACACCTTCATGAACAAAGTAAATGTGTGCTGGGCAAAGACACTTACGCCTGTGCTG  3060
Sbjct  3049  ............................................G..C..A.........  3108

Query  3061  GAAACTGCGGGTATCTCCCTGTCAGCAGAAGACTGGTCTGAACTGCTGCCCCCGTTTGCC  3120
Sbjct  3109  ............................................................  3168

Query  3121  CAGGACGTGGCGTACTCACCCGAGGTGGCATTAAACATCATATGCACGAAAATGTATGGG  3180
Sbjct  3169  ...............................................A............  3228

Query  3181  TTTGACTTAGACACTGGTCTTTTTTCCAGGCCATCAGTGCCAATGACATACACCAAAGAC  3240
Sbjct  3229  ........G.....C.............A.........A.....................  3288
```

```
Query  3241  CATTGGGATAACAGAGTTGGAGGGAAAATGTATGGATTCAGCCAACAAGCATACGATCAG  3300
Sbjct  3289  ............................................................  3348

Query  3301  CTGGCAAGACGACATCCGTACCTTCGAGGTAGAGAGAAATCAGGAATGCAGATCGTAGTC  3360
Sbjct  3349  ...................T...............A........................  3408

Query  3361  ACTGAAATGCGTATCCAGCGCCCAAGATCGGATGCCAACATCATCCCGATCAACCGCAGG  3420
Sbjct  3409  .....G......................................................  3468

Query  3421  CTCCCTCACTCACTCGTAGCCACACACGAGTATAGGCGAGCTGCACGGGCCGAGGAATTC  3480
Sbjct  3469  ............................................G...............  3528

Query  3481  TTCACCACGACACGAGGGTACACTATGCTGCTGGTCTCTGAGTATAACATGAACTTACCA  3540
Sbjct  3529  ..........T.................................................  3588

Query  3541  AACAAGAAGATCACCTGGCTGGCTCCGATAGGGACGCAGGGGGCCCATCACACCGCCAAC  3600
Sbjct  3589  ............................................................  3648

Query  3601  CTAAACTTGGGGATACCACCTCTGCTGGGCAGTTTTGATGCGGTGGTTGTGAACATGCCG  3660
Sbjct  3649  ........................................T...................  3708

Query  3661  ACTCCATTCCGGAACCATCACTACCAGCAATGTGAAGACCACGCGATGAAACTCCAGATG  3720
Sbjct  3709  ................................C...........................  3768

Query  3721  CTGGCAGGCGACGCACTGAGGCACATTAAACCTGGCGGATCATTGTGGGTCAAGGCATAC  3780
Sbjct  3769  ............................................................  3828

Query  3781  GGCTACGCAGACCGGCACAGCGAGCACGTGGTCTTGGCATTGGCTAGAAAGTTTAAAAGC  3840
Sbjct  3829  .......................................C....................  3888

Query  3841  TTCAGAGTCACACAACCCTCATGCGTGACTTCCAACACCGAGGTGTTTCTCCACTTCTCA  3900
Sbjct  3889  ...........G........G.....A.................................  3948

Query  3901  ATTTTTGACAATGGCAAACGCGCGATAGCCCTGCATTCAGCTAATAGGAAGGCTAACAGT  3960
Sbjct  3949  ............................................................  4008

Query  3961  ATCTTCCAAAACACCTTCTTACCGGCGGGCAGTGCACCGGCGTACAGAGTCAAACGTGGA  4020
Sbjct  4009  ..........................A....A............................  4068

Query  4021  GACATTTCGAACGCCCCAGAGGATGCAGTGGTCAATGCAGCAAACCAACAGGGAGTGAAG  4080
Sbjct  4069  .................................................G..........  4128

Query  4081  GGTGCTGGAGTTTGCGGTGCAATTTACCGTAAGTGGCCGGACGCTTTCGGTGATGTCGCT  4140
Sbjct  4129  ............................................................  4188

Query  4141  ACTCCAACCGGAACAGCAGTTTCGAAATCCGTCCAAGATAAATTGGTGATCCACGCTGTC  4200
Sbjct  4189  ............................................................  4248

Query  4201  GGCCCGAATTTCTCAAAATGTTCAGAAGAGGAAGGGGACAGAGACCTAGCATCTGCTTAC  4260
Sbjct  4249  ............................................................  4308

Query  4261  AGAGCTGCAGCAGAAATAGTGATGGATaaaaaaaTTACAACAGTGGCCGTCCCCTTACTC  4320
Sbjct  4309  ..........................A.................................  4368
```

```
Query  4321  TCCACCGGCATTTATGCCGGAGGAAAAAACAGAGTAGAACAGTCACTCAACCATCTCTTC  4380
Sbjct  4369  ..............C........G....................................  4428

Query  4381  ACGGCATTCGACAATACTGATGCAGATGTGACCATATATTGCATGGACAAAACATGGGAA  4440
Sbjct  4429  ........T...................................................  4488

Query  4441  AAGAAGATTAAGGAGGCAATCGATCACCGGACTTCGGTTGAGATGGTGCAGGATGACGTG  4500
Sbjct  4489  ............................................................  4548

Query  4501  CAGTTGGAGGAGGAACTGGTACGAGTACACCCTTTGAGTAGTTTAGCAGGTAGGAAGGGT  4560
Sbjct  4549  ......................................C.....................  4608

Query  4561  TACAGTACGGACAGCGGCCGAGTGTTTTCCTACCTGGAAGGTACCAAATTCCATCAGACT  4620
Sbjct  4609  ............................................................  4668

Query  4621  GCGGTGGACATAGCCGAAATGCAAGTGCTGTGGCCCGCCCTCAAAGAGTCTAATGAGCAA  4680
Sbjct  4669  ........T.............................T.....................  4728

Query  4681  ATAGTGGCATACACCTTAGGAGAATCAATGGACCAGATACGTGGCAAGTGCCCGACAGAA  4740
Sbjct  4729  ............................................................  4788

Query  4741  GATACTGACGCCTCCACACCTCCACGGACTGTGCCGTGCCTCTGTCGATACGCCATGACA  4800
Sbjct  4789  ............................................................  4848

Query  4801  CCAGAGAGAGTGTACCGACTTAAATGCACGAACACTACCCAATTTACGGTTTGCTCATCT  4860
Sbjct  4849  ............................................C.....C.........  4908

Query  4861  TTTGAGTTGCCAAAGTATCACATTCAGGGAGTGCAGAGAGTAAAATGTGAAAGAATCATC  4920
Sbjct  4909  ............................................................  4968

Query  4921  ATCTTAGATCCCACTGTTCCACCAACTTACAAACGGCCATGCATCAGACGGTACCCCTCC  4980
Sbjct  4969  ............................................................  5028

Query  4981  ACAATCTCTTGTAACTCCTCTGAGGACTCCAGGAGCTTGTCTACTTTTTCTGTCAGCTCC  5040
Sbjct  5029  ........C......................................C............  5088

Query  5041  GACTCCTCGATTGGTTCTCTGCCGGTCGGAGACACGAGACCCATTCCAGCCCCGAGGACC  5100
Sbjct  5089  .................CG..A...................T...........A......  5148

Query  5101  ATTTTCAGACCCGTCCCTGCCCCGAGAGCACCCGTGCTCAGAACCACACCGCCTCCTAAA  5160
Sbjct  5149  G...............................T..........T................  5208

Query  5161  CCACCGCGCACATTCACCGTGCGTGCAGAAGTGCACCAAGCACCCCCTACACCTGTACCT  5220
Sbjct  5209  ...................................T........................  5268

Query  5221  CCACCCAGACCGAAGAGGGCTGCAAAGTTGGCTCGTGAGATGCACCCCGGGTTCACCTTC  5280
Sbjct  5269  .....T......................................................  5328

Query  5281  GGGGACTTCGGAGAGCACGAGGTTGAGGAGCTTACGGCCTCTCCCTTAACCTTCGGAGAT  5340
Sbjct  5329  ..A..................A.................G....................  5388

Query  5341  TTTGCTGAAGGAGAGATCCAGGGGATGGGAGTGGAGTTTGAATGACTAGGCAGAGCCGGC  5400
Sbjct  5389  ....................A.......................................  5448
```

```
Query  5401  GGGTACATTTTTTCGTCAGACACGGGTCCAGGCCACCTACAGCAGAGATCCGTTTTACAA  5460
Sbjct  5449  .........................................A..................  5508

Query  5461  AATTGCACGGCAGAATGTATCTACGAACCGGCAAAACTAGAAAAAATTCATGCACCAAAG  5520
Sbjct  5509  ............................................................  5568

Query  5521  TTGGATAAAACCAAGGAAGATATCTTAAGGAGCAAGTACCAAATGAAACCGTCTGAAGCA  5580
Sbjct  5569  ............................................................  5628

Query  5581  AACAAAAGCAGGTACCAATCTAGAAAAGTAGAAAATATGAAAGCAGAGATCGTAGGTAGA  5640
Sbjct  5629  ........T.........................................T.........  5688

Query  5641  CTCTTGGACGGACTGGGGGAGTATCTGGGCACCGAGCATCCAGTTGAATGCTACCGAATA  5700
Sbjct  5689  ..............A.............................................  5748

Query  5701  ACGTACCCGGTGCCTATATACTCAACTAGTGACCTCAGAGGTCTGTCTAGTGCCAAAACA  5760
Sbjct  5749  .....T....C...................AT..G.........................  5808

Query  5761  GCTGTTAGAGCTTGCAATGCATTTTTGGAAGCTAATTTTCCATCAGTCACTTCATATAAA  5820
Sbjct  5809  ............................................................  5868

Query  5821  ATTACTGATGAATACGACGCATACCTAGATATGGTAGATGGATCAGAGAGCTGTCTGGAC  5880
Sbjct  5869  ............................................................  5928

Query  5881  AGATCCTCCTTTTCGCCGTCTAGATTGCGTAGCTTTCCAAAAACACACTCATACTTGGAC  5940
Sbjct  5929  .................A...........C..............................  5988

Query  5941  CCACAGATCAACAGTGCGGTACCGTCACCATTCCAAAACACCTTACAAAATGTATTGGCA  6000
Sbjct  5989  ..G.........................................................  6048

Query  6001  GCGGCCACCAAAAGAAACTGTAATGTCACACAGATGAGAGAACTACCAACATATGATTCT  6060
Sbjct  6049  ...............................................G............  6108

Query  6061  GCAGTGCTAAATGTAGAGGCCTTCAGGAAATATGCGTGCAAGCCAGACGTATGGGATGAG  6120
Sbjct  6109  ............................................................  6168

Query  6121  TACAGGGATAATCCGATTTGCATAACCACCGAAAATGTCACCACTTACGTCGCCAAGTTG  6180
Sbjct  6169  ..............A...................................T..T......  6228

Query  6181  AAAGGACCGAAAGCTGCGGCCTTGTTTGCAAAAACACATAACCTGATACCACTACACCAA  6240
Sbjct  6229  ............................................................  6288

Query  6241  GTTCCTATGGACAAATTCACGGTAGATATGAAGAGAGATGTCAAAGTCACGCCCGGAACC  6300
Sbjct  6289  ............................................................  6348

Query  6301  AAGCACACCGAAGAGAGACCAAAGGTACAGGTGATTCAAGCGGCAGAGCCACTAGCCACT  6360
Sbjct  6349  ............................................................  6408

Query  6361  GCCTACCTCTGCGGAATTCACCGTGAATTGGTGCGCCGTCTCAACAACGCGCTTTTCCCA  6420
Sbjct  6409  .....................................................C......  6468

Query  6421  AACATCCACACTTTGTTTGATATGTCCGCAGAGGATTTCGATGCAATCATAGCGGAACAT  6480
Sbjct  6469  ..T...................................T.....................  6528
```

```
Query  6481  TTTAAGCACGGTGACCATGTGTTGGAAACGGATATAGCCTCTTTTGACAAAAGTCAAGAT  6540
Sbjct  6529  .................C..........................................  6588

Query  6541  GATTCCATGGCACTCACTGCGTTAATGATCCTTGAGGACCTGGGAGTAGACCAAAACCTA  6600
Sbjct  6589  ............................................................  6648

Query  6601  ATGAATTTGATAGAGGCTGCATTCGGGGAAATCGTGAGTACACACTTGCCCACAGGTACT  6660
Sbjct  6649  ............................................................  6708

Query  6661  AGATTCAAATTTGGAGCTATGATGAAGTCTGGAATGTTTTTGACGCTGTTCGTCAATACA  6720
Sbjct  6709  .......G.....................C...............T..............  6768

Query  6721  ATTCTTAATGTGGTTATTGCGTGCCGAGTGTTGGAGGATCAATTGGCGCAGTCGCCGTGG  6780
Sbjct  6769  ........C..................................................C  6828

Query  6781  CCTGCTTTCATAGGAGATGACAACATAATCCATGGTATAATATCAGACAAATTGATGGCA  6840
Sbjct  6829  G..........................................................G  6888

Query  6841  GATAGATGTGCCACCTGGATGAACATGGAGGTCAAGATACTGGACTCTATAGTTGGAATA  6900
Sbjct  6889  ............................................T..C............  6948

Query  6901  CGGCCACCTTACTTCTGTGGAGGATTTATTGTATGTGACGATGTAACAGGTACAGCCTGC  6960
Sbjct  6949  ...........T................................................  7008

Query  6961  CGCGTCGCAGACCCACTGAAGAGATTGTTCAAGCTAGGTAAGCCATTGCCACTTGACGAT  7020
Sbjct  7009  ........................C...................................  7068

Query  7021  GGCCAAGATGAAGACAGAAGACGTGCATTACATGATGAAGTGAAAACCTGGTCGCGCGTA  7080
Sbjct  7069  .............................G..............................  7128

Query  7081  GGGCTGCGACACAGAGTGTGTGAAGCCATCGAAGACCGTTATGCCGTCCACTCATCAGAA  7140
Sbjct  7129  ..................................................T.........  7188

Query  7141  CTAGTTTTATTGGCACTGACTACTCTGTCTAAGAACTTGAAGTCCTTCAGAAACATAAGA  7200
Sbjct  7189  ............................................................  7248

Query  7201  GGGAAACCAATACATCTCTACGGTGGTCCTAAATAG  7236
Sbjct  7249  ....................................  7284
```

>Barmah Forest virus isolate K61404, complete genome
Sequence ID: MN689027.1 Length: 11626
Range 1: 61 to 7296

Score:12667 bits(6859), Expect:0.0,
Identities:7110/7236(98%),  Gaps:0/7236(0%), Strand: Plus/Plus

```
Query  1     ATGGCGAAACCAGTTGTGAAGATCGACGTGGAACCTGAAAGCCATTTCGCTAAGCAGGTC  60
Sbjct  61    ...............................................T............  120

Query  61    CAGAGTTGCTTCCCGCAGTTTGAGATCGAAGCAGTGCAGACCACACCAAACGATCATGCA  120
```

```
Sbjct  121   .........................................G..................  180

Query  121   CACGCGAGGGCGTTTTCGCACCTTGCTACGAAGCTCATAGAAATGGAGACAGCAAAAGAT  180
Sbjct  181   ............................................................  240

Query  181   CAGATCATCCTCGATATCGGAAGTGCACCCGCGAGGAGACTGTATTCAGAACACAAGTAC  240
Sbjct  241   ............................................................  300

Query  241   CACTGTGTTTGCCCAATGAAGTGCACGGAAGATCCAGAGAGAATGCTAGGATATGCACGT  300
Sbjct  301   ............................................................  360

Query  301   AAGTTGATCGCAGGCTCTGCGAAAGGGAAGGCAGAAAAGTTACGCGATCTCAGGGATGTC  360
Sbjct  361   ............................................................  420

Query  361   TTGGCTACGCCAGACATCGAGACGCAGTCGCTATGTCTCCACACAGACGCATCCTGCAGA  420
Sbjct  421   ............................................................  480

Query  421   TACCGCGGTGATGTTGCCGTGTATCAAGACGTGTATGCCATTGACGCACCTACCACGCTG  480
Sbjct  481   ..................................................C.........  540

Query  481   TACCACCAAGCGTTAAAGGGCGTCAGGACCGCATATTGGATAGGCTTTGATACAACGCCG  540
Sbjct  541   ...........................................................A  600

Query  541   TTCATGTACGATGCACTAGCAGGAGCTTACCCGCTCTACTCCACAAACTGGGCTGATGAG  600
Sbjct  601   .........A..................................G........C......  660

Query  601   CAAGTGCTCGAGTCCAGAAACATTGGGCTATGTTCAGACAAAGTTTCTGAAGGGGGAAAG  660
Sbjct  661   ............................................................  720

Query  661   AAAGGGAGATCAATCCTCAGGAAGAAGTTCTTGAAGCAGTCAGACAGAGTCATGTTCTCT  720
Sbjct  721   ............................................................  780

Query  721   GTCGGCTCGACGTTGTATACGGAAAGCCGTAAATTACTGCAAAGTTGGCACCTGCCATCC  780
Sbjct  781   ............................................C...............  840

Query  781   ACATTCCATCTCAAAGGCAAATCTTCGTTCACGTGCCGCTGCGACACTATCGTCAGCTGC  840
Sbjct  841   ...................C............A..............C............  900

Query  841   GAAGGGTATGTTCTGAAGAAAATTACAATGTGTCCTGGAGTGACAGGCAAACCGATAGGA  900
Sbjct  901   ................................C..C........................  960

Query  901   TATGCCGTCACCCATCACAAAGAAGGATTCGTAGTCGGAAAAGTCACAGATACCATTCGC  960
Sbjct  961   ....................G.............................C.........  1020

Query  961   GGCGAGAGAGTCTCCTTCGCCGTGTGTACTTATGTACCAACAACACTCTGCGACCAGATG  1020
Sbjct  1021  ............................................................  1080

Query  1021  ACCGGGATCCTAGCAACAGAAGTAACAGCCGATGATGCCCAGAAACTGCTGGTGGGTTTG  1080
Sbjct  1081  ........T...................................................  1140

Query  1081  AACCAGAGAATAGTAGTTAATGGTAGGACCCAGAGAAATACCAATACTATGAAGAACTAC  1140
Sbjct  1141  ............................................C...............  1200

Query  1141  CTGCTACCACTGGTTGCACAAGCGCTAGCAAAATGGGCGAAGGAAGCAAAACAGGATATG  1200
```

```
Sbjct  1201  ..........................T...........A.....................  1260

Query  1201  GAAGATGAAAGACCCCTGAACGAACGCCAACGAACGCTAACGTGCCTCTGCTGCTGGGCA  1260
Sbjct  1261  ............................................................  1320

Query  1261  TTTAAGCGAAACAAACGCCACGCCATTTACAAGAGACCAGACACACAGAGTATAGTCAAG  1320
Sbjct  1321  ............................................................  1380

Query  1321  GTCCCTTGCGAATTCACAAGCTTTCCTTTGGTCAGCCTGTGGTCCGCTGGGATGTCTATA  1380
Sbjct  1381  ............................................................  1440

Query  1381  TCTCTTAGGCAGAAGTTGAAGATGATGCTGCAGGCGAGGCAGCCCACACAAATAGCAGCA  1440
Sbjct  1441  ............................................................  1500

Query  1441  GTGACTGAGGAACTCATACAAGAAGCAGCTGCAGTAGAGCAAGAGGCCGTGGATACGGCC  1500
Sbjct  1501  ..................................C.........................  1560

Query  1501  AATGCCGAGCTGGACCACGCCGCATGGCCCTCCATTGTGGATACGACAGAGCGCCATGTT  1560
Sbjct  1561  ...............................................G...........C  1620

Query  1561  GAGGTCGAAGTGGAAGAACTCGACCAGCGTGCAGGGGAAGGGGTAGTGGAAACACCTCGA  1620
Sbjct  1621  ............................................................  1680

Query  1621  AACTCTATCAAAGTTTCAACACAGATCGGGGACGCGTTAATCGGCAGTTACCTGATCCTA  1680
Sbjct  1681  ........T...................................................  1740

Query  1681  TCACCCCAAGCAGTCCTACGCAGCGAAAAATTAGCCTGCATACATGATCTTGCAGAGCAG  1740
Sbjct  1741  .......................................C....................  1800

Query  1741  GTTAAGTTGGTCACACACTCTGGCCGTAGTGGTAGGTACGCCGTCGACAAATACNACGGA  1800
Sbjct  1801  ..C.................C..T..............................G.....  1860

Query  1801  AGAGTACTAGTCCCTACAGGAGTGGCTATAGACATTCAATCGTTCCAGGCTCTCAGTGAG  1860
Sbjct  1861  ...................................C........................  1920

Query  1861  AGCGCGACCCTTGTGTACAACGAACGCGAGTTCGTTAACAGGAAGCTGTGGCACATAGCA  1920
Sbjct  1921  ..T..........................................T..............  1980

Query  1921  GTATACGGGGCAGCACTCAATACTGATGAAGAAGGATACGAGAAGGTCCCGGTAGAGAGA  1980
Sbjct  1981  ............................................................  2040

Query  1981  GCAGAATCAGATTATGTGTTTGATGTAGACCAAAAAATGTGCCTaaaaaaaGAGCAGGCA  2040
Sbjct  2041  .................A............A.............C...............  2100

Query  2041  TCAGGTTGGGTACTCTGTGGCGAACTAGTCAACCCCCCATTCCACGAATTCGCATATGAA  2100
Sbjct  2101  .....A......................................................  2160

Query  2101  GGGCTCCGCACGAGACCGTCAGCACCCTACAAGGTTCATACAGTAGGTGTGTACGGAGTG  2160
Sbjct  2161  ........T..T................................................  2220

Query  2161  CCAGGATCAGGCAAATCCGCAATAATCAAGAACACGGTCACCATGTCTGACCTAGTATTG  2220
Sbjct  2221  ............................................................  2280

Query  2221  AGTGGTAAGAAAGAGAACTGCTTAGAAATTATGAACGATGTACTTAAACACAGAGCTCTA  2280
```

```
Sbjct  2281  ............................................................  2340

Query  2281  CGTATCACAGCGAAGACCGTAGACTCAGTGTTATTAAACGGCGTGAAACACACGCCTAAC  2340
Sbjct  2341  .....T.............................G........................  2400

Query  2341  ATACTATACATCGACGAAGCGTTCTCATGCCATGCAGGGACTCTGTTGGCCACTATAGCC  2400
Sbjct  2401  ............................................................  2460

Query  2401  ATAGTCAGGCCCAAACAGAAAGTGGTACTGTGCGGAGACCCGAAACAATGCGGATTCTTC  2460
Sbjct  2461  T...........................................................  2520

Query  2461  AATATGATGCAACTGAAAGTTAATTACAATCATGACATCTGCTCAGAAGTCTTCCACAAA  2520
Sbjct  2521  .......................C....................................  2580

Query  2521  AGTATCTCTAGACGGTGCACCCAGGATATCACGGCCATCGTTTCCAAATTACATTACCAG  2580
Sbjct  2581  ............................................T...............  2640

Query  2581  GACCGAATGAGGACCACAAACCCCCGAAAAGGAGACATCATTATAGACACTACCGGCACT  2640
Sbjct  2641  ..................................................C.........  2700

Query  2641  ACCAAACCAGCCAAAACAGATCTGATTCTGACGTGCTTCAGGGGATGGGTGAAACAGTTG  2700
Sbjct  2701  ....................C.......................................  2760

Query  2701  CAGCAAGACTACAGAGGTAACGAAGTAATGACGGCTGCAGCGTCCCAAGGACTGACGAGG  2760
Sbjct  2761  ............................................................  2820

Query  2761  GCCTCCGTATATGCGGTTCGAACTAAAGTCAATGAGAACCCGCTATATGCACAGACCTCC  2820
Sbjct  2821  ............................................................  2880

Query  2821  GAGCACGTGAACGTGTTGTTAACACGCACAGAAAACAAGCTAGTATGGAAGACCTTGTCA  2880
Sbjct  2881  .......................................T....................  2940

Query  2881  ACAGATCCCTGGATTAAAACACTGACTAACCCACCTAGAGGGCACTATACCGCCACCATA  2940
Sbjct  2941  ............................................................  3000

Query  2941  GCAGAATGGGAAGCGGAACACCAGGGTATAATGAAGGCCATACAAGGGTATGCACCGCCC  3000
Sbjct  3001  ..............A.............................................  3060

Query  3001  GTGAACACCTTCATGAACAAAGTAAATGTGTGCTGGGCAAAGACACTTACGCCTGTGCTG  3060
Sbjct  3061  ...............................................C..A.........  3120

Query  3061  GAAACTGCGGGTATCTCCCTGTCAGCAGAAGACTGGTCTGAACTGCTGCCCCCGTTTGCC  3120
Sbjct  3121  ............................................................  3180

Query  3121  CAGGACGTGGCGTACTCACCCGAGGTGGCATTAAACATCATATGCACGAAAATGTATGGG  3180
Sbjct  3181  ...............................................A............  3240

Query  3181  TTTGACTTAGACACTGGTCTTTTTTCCAGGCCATCAGTGCCAATGACATACACCAAAGAC  3240
Sbjct  3241  ........G.....C.............A.........A........T............  3300

Query  3241  CATTGGGATAACAGAGTTGGAGGGAAAATGTATGGATTCAGCCAACAAGCATACGATCAG  3300
Sbjct  3301  ............................................................  3360

Query  3301  CTGGCAAGACGACATCCGTACCTTCGAGGTAGAGAGAAATCAGGAATGCAGATCGTAGTC  3360
```

```
Sbjct  3361  ...................................A........................  3420

Query  3361  ACTGAAATGCGTATCCAGCGCCCAAGATCGGATGCCAACATCATCCCGATCAACCGCAGG  3420
Sbjct  3421  .....G......................................................  3480

Query  3421  CTCCCTCACTCACTCGTAGCCACACACGAGTATAGGCGAGCTGCACGGGCCGAGGAATTC  3480
Sbjct  3481  ............................................G...............  3540

Query  3481  TTCACCACGACACGAGGGTACACTATGCTGCTGGTCTCTGAGTATAACATGAACTTACCA  3540
Sbjct  3541  ..........T.................................................  3600

Query  3541  AACAAGAAGATCACCTGGCTGGCTCCGATAGGGACGCAGGGGGCCCATCACACCGCCAAC  3600
Sbjct  3601  ............................................................  3660

Query  3601  CTAAACTTGGGGATACCACCTCTGCTGGGCAGTTTTGATGCGGTGGTTGTGAACATGCCG  3660
Sbjct  3661  ........................................T...................  3720

Query  3661  ACTCCATTCCGGAACCATCACTACCAGCAATGTGAAGACCACGCGATGAAACTCCAGATG  3720
Sbjct  3721  ................................C...........................  3780

Query  3721  CTGGCAGGCGACGCACTGAGGCACATTAAACCTGGCGGATCATTGTGGGTCAAGGCATAC  3780
Sbjct  3781  ............................................................  3840

Query  3781  GGCTACGCAGACCGGCACAGCGAGCACGTGGTCTTGGCATTGGCTAGAAAGTTTAAAAGC  3840
Sbjct  3841  .......................................C....................  3900

Query  3841  TTCAGAGTCACACAACCCTCATGCGTGACTTCCAACACCGAGGTGTTTCTCCACTTCTCA  3900
Sbjct  3901  ...........G........G.....A.................................  3960

Query  3901  ATTTTTGACAATGGCAAACGCGCGATAGCCCTGCATTCAGCTAATAGGAAGGCTAACAGT  3960
Sbjct  3961  ............................................................  4020

Query  3961  ATCTTCCAAAACACCTTCTTACCGGCGGGCAGTGCACCGGCGTACAGAGTCAAACGTGGA  4020
Sbjct  4021  ..........................A....A............................  4080

Query  4021  GACATTTCGAACGCCCCAGAGGATGCAGTGGTCAATGCAGCAAACCAACAGGGAGTGAAG  4080
Sbjct  4081  .................................................G..........  4140

Query  4081  GGTGCTGGAGTTTGCGGTGCAATTTACCGTAAGTGGCCGGACGCTTTCGGTGATGTCGCT  4140
Sbjct  4141  ............................................................  4200

Query  4141  ACTCCAACCGGAACAGCAGTTTCGAAATCCGTCCAAGATAAATTGGTGATCCACGCTGTC  4200
Sbjct  4201  ............................................................  4260

Query  4201  GGCCCGAATTTCTCAAAATGTTCAGAAGAGGAAGGGGACAGAGACCTAGCATCTGCTTAC  4260
Sbjct  4261  ............................................................  4320

Query  4261  AGAGCTGCAGCAGAAATAGTGATGGATaaaaaaaTTACAACAGTGGCCGTCCCCTTACTC  4320
Sbjct  4321  ..........................A.................................  4380

Query  4321  TCCACCGGCATTTATGCCGGAGGAAAAAACAGAGTAGAACAGTCACTCAACCATCTCTTC  4380
Sbjct  4381  ..............C........G....................................  4440

Query  4381  ACGGCATTCGACAATACTGATGCAGATGTGACCATATATTGCATGGACAAAACATGGGAA  4440
```

```
Sbjct  4441  ........T...................................................  4500

Query  4441  AAGAAGATTAAGGAGGCAATCGATCACCGGACTTCGGTTGAGATGGTGCAGGATGACGTG  4500
Sbjct  4501  ............................................................  4560

Query  4501  CAGTTGGAGGAGGAACTGGTACGAGTACACCCTTTGAGTAGTTTAGCAGGTAGGAAGGGT  4560
Sbjct  4561  ......................................C.....................  4620

Query  4561  TACAGTACGGACAGCGGCCGAGTGTTTTCCTACCTGGAAGGTACCAAATTCCATCAGACT  4620
Sbjct  4621  ............................................................  4680

Query  4621  GCGGTGGACATAGCCGAAATGCAAGTGCTGTGGCCCGCCCTCAAAGAGTCTAATGAGCAA  4680
Sbjct  4681  ........T.............................T.....................  4740

Query  4681  ATAGTGGCATACACCTTAGGAGAATCAATGGACCAGATACGTGGCAAGTGCCCGACAGAA  4740
Sbjct  4741  ............................................................  4800

Query  4741  GATACTGACGCCTCCACACCTCCACGGACTGTGCCGTGCCTCTGTCGATACGCCATGACA  4800
Sbjct  4801  ............................................................  4860

Query  4801  CCAGAGAGAGTGTACCGACTTAAATGCACGAACACTACCCAATTTACGGTTTGCTCATCT  4860
Sbjct  4861  ............................................C.....C.........  4920

Query  4861  TTTGAGTTGCCAAAGTATCACATTCAGGGAGTGCAGAGAGTAAAATGTGAAAGAATCATC  4920
Sbjct  4921  ............................................................  4980

Query  4921  ATCTTAGATCCCACTGTTCCACCAACTTACAAACGGCCATGCATCAGACGGTACCCCTCC  4980
Sbjct  4981  ............................................................  5040

Query  4981  ACAATCTCTTGTAACTCCTCTGAGGACTCCAGGAGCTTGTCTACTTTTTCTGTCAGCTCC  5040
Sbjct  5041  ........C...................................C...........  5100

Query  5041  GACTCCTCGATTGGTTCTCTGCCGGTCGGAGACACGAGACCCATTCCAGCCCCGAGGACC  5100
Sbjct  5101  .................CG..A..................T...........A......  5160

Query  5101  ATTTTCAGACCCGTCCCTGCCCCGAGAGCACCCGTGCTCAGAACCACACCGCCTCCTAAA  5160
Sbjct  5161  G.C.........................................T................  5220

Query  5161  CCACCGCGCACATTCACCGTGCGTGCAGAAGTGCACCAAGCACCCCCTACACCTGTACCT  5220
Sbjct  5221  ...................................T........................  5280

Query  5221  CCACCCAGACCGAAGAGGGCTGCAAAGTTGGCTCGTGAGATGCACCCCGGGTTCACCTTC  5280
Sbjct  5281  .....T......................................................  5340

Query  5281  GGGGACTTCGGAGAGCACGAGGTTGAGGAGCTTACGGCCTCTCCCTTAACCTTCGGAGAT  5340
Sbjct  5341  ..A..................A.................G....................  5400

Query  5341  TTTGCTGAAGGAGAGATCCAGGGGATGGGAGTGGAGTTTGAATGACTAGGCAGAGCCGGC  5400
Sbjct  5401  ....................A.......................................  5460

Query  5401  GGGTACATTTTTTCGTCAGACACGGGTCCAGGCCACCTACAGCAGAGATCCGTTTTACAA  5460
Sbjct  5461  ........................................A.................  5520

Query  5461  AATTGCACGGCAGAATGTATCTACGAACCGGCAAAACTAGAAAAAATTCATGCACCAAAG  5520
```

```
Sbjct  5521  ............................................................  5580

Query  5521  TTGGATAAAACCAAGGAAGATATCTTAAGGAGCAAGTACCAAATGAAACCGTCTGAAGCA  5580
Sbjct  5581  ............................................................  5640

Query  5581  AACAAAAGCAGGTACCAATCTAGAAAAGTAGAAAATATGAAAGCAGAGATCGTAGGTAGA  5640
Sbjct  5641  ........T.........................................T.........  5700

Query  5641  CTCTTGGACGGACTGGGGGAGTATCTGGGCACCGAGCATCCAGTTGAATGCTACCGAATA  5700
Sbjct  5701  ..............A.............................................  5760

Query  5701  ACGTACCCGGTGCCTATATACTCAACTAGTGACCTCAGAGGTCTGTCTAGTGCCAAAACA  5760
Sbjct  5761  .....T....C...................AT..G.........................  5820

Query  5761  GCTGTTAGAGCTTGCAATGCATTTTTGGAAGCTAATTTTCCATCAGTCACTTCATATAAA  5820
Sbjct  5821  ......................................C.....................  5880

Query  5821  ATTACTGATGAATACGACGCATACCTAGATATGGTAGATGGATCAGAGAGCTGTCTGGAC  5880
Sbjct  5881  ............................................................  5940

Query  5881  AGATCCTCCTTTTCGCCGTCTAGATTGCGTAGCTTTCCAAAAACACACTCATACTTGGAC  5940
Sbjct  5941  .................A...........C..............................  6000

Query  5941  CCACAGATCAACAGTGCGGTACCGTCACCATTCCAAAACACCTTACAAAATGTATTGGCA  6000
Sbjct  6001  ..G.........................................................  6060

Query  6001  GCGGCCACCAAAAGAAACTGTAATGTCACACAGATGAGAGAACTACCAACATATGATTCT  6060
Sbjct  6061  ...............................................G............  6120

Query  6061  GCAGTGCTAAATGTAGAGGCCTTCAGGAAATATGCGTGCAAGCCAGACGTATGGGATGAG  6120
Sbjct  6121  ............................................................  6180

Query  6121  TACAGGGATAATCCGATTTGCATAACCACCGAAAATGTCACCACTTACGTCGCCAAGTTG  6180
Sbjct  6181  ..............A...................................T..T......  6240

Query  6181  AAAGGACCGAAAGCTGCGGCCTTGTTTGCAAAAACACATAACCTGATACCACTACACCAA  6240
Sbjct  6241  ............................................................  6300

Query  6241  GTTCCTATGGACAAATTCACGGTAGATATGAAGAGAGATGTCAAAGTCACGCCCGGAACC  6300
Sbjct  6301  ............................................................  6360

Query  6301  AAGCACACCGAAGAGAGACCAAAGGTACAGGTGATTCAAGCGGCAGAGCCACTAGCCACT  6360
Sbjct  6361  ....................G.......................................  6420

Query  6361  GCCTACCTCTGCGGAATTCACCGTGAATTGGTGCGCCGTCTCAACAACGCGCTTTTCCCA  6420
Sbjct  6421  .....................................................C......  6480

Query  6421  AACATCCACACTTTGTTTGATATGTCCGCAGAGGATTTCGATGCAATCATAGCGGAACAT  6480
Sbjct  6481  ..T...................................T.....................  6540

Query  6481  TTTAAGCACGGTGACCATGTGTTGGAAACGGATATAGCCTCTTTTGACAAAAGTCAAGAT  6540
Sbjct  6541  .................C..........................................  6600

Query  6541  GATTCCATGGCACTCACTGCGTTAATGATCCTTGAGGACCTGGGAGTAGACCAAAACCTA  6600
```

```
Sbjct  6601  ............................................................  6660

Query  6601  ATGAATTTGATAGAGGCTGCATTCGGGGAAATCGTGAGTACACACTTGCCCACAGGTACT  6660
Sbjct  6661  ............................................................  6720

Query  6661  AGATTCAAATTTGGAGCTATGATGAAGTCTGGAATGTTTTTGACGCTGTTCGTCAATACA  6720
Sbjct  6721  .......G.....................C...............T..............  6780

Query  6721  ATTCTTAATGTGGTTATTGCGTGCCGAGTGTTGGAGGATCAATTGGCGCAGTCGCCGTGG  6780
Sbjct  6781  ........C..................................................C  6840

Query  6781  CCTGCTTTCATAGGAGATGACAACATAATCCATGGTATAATATCAGACAAATTGATGGCA  6840
Sbjct  6841  G...........................................................  6900

Query  6841  GATAGATGTGCCACCTGGATGAACATGGAGGTCAAGATACTGGACTCTATAGTTGGAATA  6900
Sbjct  6901  ............................................T..C............  6960

Query  6901  CGGCCACCTTACTTCTGTGGAGGATTTATTGTATGTGACGATGTAACAGGTACAGCCTGC  6960
Sbjct  6961  ...........T................................................  7020

Query  6961  CGCGTCGCAGACCCACTGAAGAGATTGTTCAAGCTAGGTAAGCCATTGCCACTTGACGAT  7020
Sbjct  7021  ........................C...................................  7080

Query  7021  GGCCAAGATGAAGACAGAAGACGTGCATTACATGATGAAGTGAAAACCTGGTCGCGCGTA  7080
Sbjct  7081  .............................G..............................  7140

Query  7081  GGGCTGCGACACAGAGTGTGTGAAGCCATCGAAGACCGTTATGCCGTCCACTCATCAGAA  7140
Sbjct  7141  ..................................................T.........  7200

Query  7141  CTAGTTTTATTGGCACTGACTACTCTGTCTAAGAACTTGAAGTCCTTCAGAAACATAAGA  7200
Sbjct  7201  ............................................................  7260

Query  7201  GGGAAACCAATACATCTCTACGGTGGTCCTAAATAG  7236
Sbjct  7261  ....................................  7296
```

>Barmah Forest virus strain SW94093, complete genome
Sequence ID: MW835350.1 Length: 11535
Range 1: 51 to 7286

Score:12661 bits(6856), Expect:0.0,
Identities:7109/7236(98%),  Gaps:0/7236(0%), Strand: Plus/Plus

```
Query  1     ATGGCGAAACCAGTTGTGAAGATCGACGTGGAACCTGAAAGCCATTTCGCTAAGCAGGTC  60
Sbjct  51    ...............................................T............  110

Query  61    CAGAGTTGCTTCCCGCAGTTTGAGATCGAAGCAGTGCAGACCACACCAAACGATCATGCA  120
Sbjct  111   .........................................G..................  170

Query  121   CACGCGAGGGCGTTTTCGCACCTTGCTACGAAGCTCATAGAAATGGAGACAGCAAAAGAT  180
Sbjct  171   ............................................................  230
```

```
Query  181   CAGATCATCCTCGATATCGGAAGTGCACCCGCGAGGAGACTGTATTCAGAACACAAGTAC  240
Sbjct  231   ............................................................  290

Query  241   CACTGTGTTTGCCCAATGAAGTGCACGGAAGATCCAGAGAGAATGCTAGGATATGCACGT  300
Sbjct  291   ............................................................  350

Query  301   AAGTTGATCGCAGGCTCTGCGAAAGGGAAGGCAGAAAAGTTACGCGATCTCAGGGATGTC  360
Sbjct  351   ............................................................  410

Query  361   TTGGCTACGCCAGACATCGAGACGCAGTCGCTATGTCTCCACACAGACGCATCCTGCAGA  420
Sbjct  411   ............................................................  470

Query  421   TACCGCGGTGATGTTGCCGTGTATCAAGACGTGTATGCCATTGACGCACCTACCACGCTG  480
Sbjct  471   ..................................................C.........  530

Query  481   TACCACCAAGCGTTAAAGGGCGTCAGGACCGCATATTGGATAGGCTTTGATACAACGCCG  540
Sbjct  531   ...........................................................A  590

Query  541   TTCATGTACGATGCACTAGCAGGAGCTTACCCGCTCTACTCCACAAACTGGGCTGATGAG  600
Sbjct  591   .........A...........................................C......  650

Query  601   CAAGTGCTCGAGTCCAGAAACATTGGGCTATGTTCAGACAAAGTTTCTGAAGGGGGAAAG  660
Sbjct  651   ............................................................  710

Query  661   AAAGGGAGATCAATCCTCAGGAAGAAGTTCTTGAAGCAGTCAGACAGAGTCATGTTCTCT  720
Sbjct  711   ............................................................  770

Query  721   GTCGGCTCGACGTTGTATACGGAAAGCCGTAAATTACTGCAAAGTTGGCACCTGCCATCC  780
Sbjct  771   ............................................C...............  830

Query  781   ACATTCCATCTCAAAGGCAAATCTTCGTTCACGTGCCGCTGCGACACTATCGTCAGCTGC  840
Sbjct  831   ................................A..............C............  890

Query  841   GAAGGGTATGTTCTGAAGAAAATTACAATGTGTCCTGGAGTGACAGGCAAACCGATAGGA  900
Sbjct  891   ................................C..C........................  950

Query  901   TATGCCGTCACCCATCACAAAGAAGGATTCGTAGTCGGAAAAGTCACAGATACCATTCGC  960
Sbjct  951   ....................G.............................C.........  1010

Query  961   GGCGAGAGAGTCTCCTTCGCCGTGTGTACTTATGTACCAACAACACTCTGCGACCAGATG  1020
Sbjct  1011  ............................................................  1070

Query  1021  ACCGGGATCCTAGCAACAGAAGTAACAGCCGATGATGCCCAGAAACTGCTGGTGGGTTTG  1080
Sbjct  1071  ........T...................................................  1130

Query  1081  AACCAGAGAATAGTAGTTAATGGTAGGACCCAGAGAAATACCAATACTATGAAGAACTAC  1140
Sbjct  1131  ............................................C...............  1190

Query  1141  CTGCTACCACTGGTTGCACAAGCGCTAGCAAAATGGGCGAAGGAAGCAAAACAGGATATG  1200
Sbjct  1191  ......................................A.....................  1250

Query  1201  GAAGATGAAAGACCCCTGAACGAACGCCAACGAACGCTAACGTGCCTCTGCTGCTGGGCA  1260
Sbjct  1251  ............................................................  1310
```

```
Query  1261  TTTAAGCGAAACAAACGCCACGCCATTTACAAGAGACCAGACACACAGAGTATAGTCAAG  1320
Sbjct  1311  ............................................................  1370

Query  1321  GTCCCTTGCGAATTCACAAGCTTTCCTTTGGTCAGCCTGTGGTCCGCTGGGATGTCTATA  1380
Sbjct  1371  ............................................................  1430

Query  1381  TCTCTTAGGCAGAAGTTGAAGATGATGCTGCAGGCGAGGCAGCCCACACAAATAGCAGCA  1440
Sbjct  1431  ............................................................  1490

Query  1441  GTGACTGAGGAACTCATACAAGAAGCAGCTGCAGTAGAGCAAGAGGCCGTGGATACGGCC  1500
Sbjct  1491  ..................................C.........................  1550

Query  1501  AATGCCGAGCTGGACCACGCCGCATGGCCCTCCATTGTGGATACGACAGAGCGCCATGTT  1560
Sbjct  1551  ...............................................G............  1610

Query  1561  GAGGTCGAAGTGGAAGAACTCGACCAGCGTGCAGGGGAAGGGGTAGTGGAAACACCTCGA  1620
Sbjct  1611  ............................................................  1670

Query  1621  AACTCTATCAAAGTTTCAACACAGATCGGGGACGCGTTAATCGGCAGTTACCTGATCCTA  1680
Sbjct  1671  ........T........T..........................................  1730

Query  1681  TCACCCCAAGCAGTCCTACGCAGCGAAAAATTAGCCTGCATACATGATCTTGCAGAGCAG  1740
Sbjct  1731  ............................................................  1790

Query  1741  GTTAAGTTGGTCACACACTCTGGCCGTAGTGGTAGGTACGCCGTCGACAAATACNACGGA  1800
Sbjct  1791  ..C.................C..T..............................G.....  1850

Query  1801  AGAGTACTAGTCCCTACAGGAGTGGCTATAGACATTCAATCGTTCCAGGCTCTCAGTGAG  1860
Sbjct  1851  ...................................C........................  1910

Query  1861  AGCGCGACCCTTGTGTACAACGAACGCGAGTTCGTTAACAGGAAGCTGTGGCACATAGCA  1920
Sbjct  1911  ..T..........................................T..............  1970

Query  1921  GTATACGGGGCAGCACTCAATACTGATGAAGAAGGATACGAGAAGGTCCCGGTAGAGAGA  1980
Sbjct  1971  ............................................................  2030

Query  1981  GCAGAATCAGATTATGTGTTTGATGTAGACCAAAAAATGTGCCTaaaaaaaGAGCAGGCA  2040
Sbjct  2031  .................A............A.............C...............  2090

Query  2041  TCAGGTTGGGTACTCTGTGGCGAACTAGTCAACCCCCCATTCCACGAATTCGCATATGAA  2100
Sbjct  2091  .....A......................................................  2150

Query  2101  GGGCTCCGCACGAGACCGTCAGCACCCTACAAGGTTCATACAGTAGGTGTGTACGGAGTG  2160
Sbjct  2151  ........T..T................................................  2210

Query  2161  CCAGGATCAGGCAAATCCGCAATAATCAAGAACACGGTCACCATGTCTGACCTAGTATTG  2220
Sbjct  2211  ............................................................  2270

Query  2221  AGTGGTAAGAAAGAGAACTGCTTAGAAATTATGAACGATGTACTTAAACACAGAGCTCTA  2280
Sbjct  2271  ............................................................  2330

Query  2281  CGTATCACAGCGAAGACCGTAGACTCAGTGTTATTAAACGGCGTGAAACACACGCCTAAC  2340
Sbjct  2331  .....T.............................G........................  2390
```

```
Query  2341  ATACTATACATCGACGAAGCGTTCTCATGCCATGCAGGGACTCTGTTGGCCACTATAGCC  2400
Sbjct  2391  ............................................................  2450

Query  2401  ATAGTCAGGCCCAAACAGAAAGTGGTACTGTGCGGAGACCCGAAACAATGCGGATTCTTC  2460
Sbjct  2451  T...........................................................  2510

Query  2461  AATATGATGCAACTGAAAGTTAATTACAATCATGACATCTGCTCAGAAGTCTTCCACAAA  2520
Sbjct  2511  .......................C....................................  2570

Query  2521  AGTATCTCTAGACGGTGCACCCAGGATATCACGGCCATCGTTTCCAAATTACATTACCAG  2580
Sbjct  2571  ............................................T...............  2630

Query  2581  GACCGAATGAGGACCACAAACCCCCGAAAAGGAGACATCATTATAGACACTACCGGCACT  2640
Sbjct  2631  ..................................................C.........  2690

Query  2641  ACCAAACCAGCCAAAACAGATCTGATTCTGACGTGCTTCAGGGGATGGGTGAAACAGTTG  2700
Sbjct  2691  ....................C.......................................  2750

Query  2701  CAGCAAGACTACAGAGGTAACGAAGTAATGACGGCTGCAGCGTCCCAAGGACTGACGAGG  2760
Sbjct  2751  ............................................................  2810

Query  2761  GCCTCCGTATATGCGGTTCGAACTAAAGTCAATGAGAACCCGCTATATGCACAGACCTCC  2820
Sbjct  2811  ............................................................  2870

Query  2821  GAGCACGTGAACGTGTTGTTAACACGCACAGAAAACAAGCTAGTATGGAAGACCTTGTCA  2880
Sbjct  2871  .......................................T....................  2930

Query  2881  ACAGATCCCTGGATTAAAACACTGACTAACCCACCTAGAGGGCACTATACCGCCACCATA  2940
Sbjct  2931  ............................................................  2990

Query  2941  GCAGAATGGGAAGCGGAACACCAGGGTATAATGAAGGCCATACAAGGGTATGCACCGCCC  3000
Sbjct  2991  ..............A.............................................  3050

Query  3001  GTGAACACCTTCATGAACAAAGTAAATGTGTGCTGGGCAAAGACACTTACGCCTGTGCTG  3060
Sbjct  3051  ...........T................................G..C..A.........  3110

Query  3061  GAAACTGCGGGTATCTCCCTGTCAGCAGAAGACTGGTCTGAACTGCTGCCCCCGTTTGCC  3120
Sbjct  3111  ............................................................  3170

Query  3121  CAGGACGTGGCGTACTCACCCGAGGTGGCATTAAACATCATATGCACGAAAATGTATGGG  3180
Sbjct  3171  ...............................................A............  3230

Query  3181  TTTGACTTAGACACTGGTCTTTTTTCCAGGCCATCAGTGCCAATGACATACACCAAAGAC  3240
Sbjct  3231  ........G.....C.............A.........A.....................  3290

Query  3241  CATTGGGATAACAGAGTTGGAGGGAAAATGTATGGATTCAGCCAACAAGCATACGATCAG  3300
Sbjct  3291  ............................................................  3350

Query  3301  CTGGCAAGACGACATCCGTACCTTCGAGGTAGAGAGAAATCAGGAATGCAGATCGTAGTC  3360
Sbjct  3351  ...................................A........................  3410

Query  3361  ACTGAAATGCGTATCCAGCGCCCAAGATCGGATGCCAACATCATCCCGATCAACCGCAGG  3420
Sbjct  3411  .....G......................................................  3470
```

```
Query  3421  CTCCCTCACTCACTCGTAGCCACACACGAGTATAGGCGAGCTGCACGGGCCGAGGAATTC  3480
Sbjct  3471  ............................................G...............  3530

Query  3481  TTCACCACGACACGAGGGTACACTATGCTGCTGGTCTCTGAGTATAACATGAACTTACCA  3540
Sbjct  3531  ..........T.................................................  3590

Query  3541  AACAAGAAGATCACCTGGCTGGCTCCGATAGGGACGCAGGGGGCCCATCACACCGCCAAC  3600
Sbjct  3591  ............................................................  3650

Query  3601  CTAAACTTGGGGATACCACCTCTGCTGGGCAGTTTTGATGCGGTGGTTGTGAACATGCCG  3660
Sbjct  3651  ........................................T...................  3710

Query  3661  ACTCCATTCCGGAACCATCACTACCAGCAATGTGAAGACCACGCGATGAAACTCCAGATG  3720
Sbjct  3711  ................................C...........................  3770

Query  3721  CTGGCAGGCGACGCACTGAGGCACATTAAACCTGGCGGATCATTGTGGGTCAAGGCATAC  3780
Sbjct  3771  .............................G..............................  3830

Query  3781  GGCTACGCAGACCGGCACAGCGAGCACGTGGTCTTGGCATTGGCTAGAAAGTTTAAAAGC  3840
Sbjct  3831  .......................................C....................  3890

Query  3841  TTCAGAGTCACACAACCCTCATGCGTGACTTCCAACACCGAGGTGTTTCTCCACTTCTCA  3900
Sbjct  3891  ...........G........G.....A.................................  3950

Query  3901  ATTTTTGACAATGGCAAACGCGCGATAGCCCTGCATTCAGCTAATAGGAAGGCTAACAGT  3960
Sbjct  3951  ............................................................  4010

Query  3961  ATCTTCCAAAACACCTTCTTACCGGCGGGCAGTGCACCGGCGTACAGAGTCAAACGTGGA  4020
Sbjct  4011  ..........................A....A............................  4070

Query  4021  GACATTTCGAACGCCCCAGAGGATGCAGTGGTCAATGCAGCAAACCAACAGGGAGTGAAG  4080
Sbjct  4071  ............................................................  4130

Query  4081  GGTGCTGGAGTTTGCGGTGCAATTTACCGTAAGTGGCCGGACGCTTTCGGTGATGTCGCT  4140
Sbjct  4131  ............................................................  4190

Query  4141  ACTCCAACCGGAACAGCAGTTTCGAAATCCGTCCAAGATAAATTGGTGATCCACGCTGTC  4200
Sbjct  4191  ............................................................  4250

Query  4201  GGCCCGAATTTCTCAAAATGTTCAGAAGAGGAAGGGGACAGAGACCTAGCATCTGCTTAC  4260
Sbjct  4251  ...........T................................................  4310

Query  4261  AGAGCTGCAGCAGAAATAGTGATGGATaaaaaaaTTACAACAGTGGCCGTCCCCTTACTC  4320
Sbjct  4311  ..........................A...........................C.....  4370

Query  4321  TCCACCGGCATTTATGCCGGAGGAAAAAACAGAGTAGAACAGTCACTCAACCATCTCTTC  4380
Sbjct  4371  ..............C........G....................................  4430

Query  4381  ACGGCATTCGACAATACTGATGCAGATGTGACCATATATTGCATGGACAAAACATGGGAA  4440
Sbjct  4431  ........T...................................................  4490

Query  4441  AAGAAGATTAAGGAGGCAATCGATCACCGGACTTCGGTTGAGATGGTGCAGGATGACGTG  4500
Sbjct  4491  ............................................................  4550
```

```
Query  4501  CAGTTGGAGGAGGAACTGGTACGAGTACACCCTTTGAGTAGTTTAGCAGGTAGGAAGGGT  4560
Sbjct  4551  ......................................C.....................  4610

Query  4561  TACAGTACGGACAGCGGCCGAGTGTTTTCCTACCTGGAAGGTACCAAATTCCATCAGACT  4620
Sbjct  4611  ............................................................  4670

Query  4621  GCGGTGGACATAGCCGAAATGCAAGTGCTGTGGCCCGCCCTCAAAGAGTCTAATGAGCAA  4680
Sbjct  4671  ........T.............................T.....................  4730

Query  4681  ATAGTGGCATACACCTTAGGAGAATCAATGGACCAGATACGTGGCAAGTGCCCGACAGAA  4740
Sbjct  4731  ............................................................  4790

Query  4741  GATACTGACGCCTCCACACCTCCACGGACTGTGCCGTGCCTCTGTCGATACGCCATGACA  4800
Sbjct  4791  ............................................................  4850

Query  4801  CCAGAGAGAGTGTACCGACTTAAATGCACGAACACTACCCAATTTACGGTTTGCTCATCT  4860
Sbjct  4851  ............................................C.....C.........  4910

Query  4861  TTTGAGTTGCCAAAGTATCACATTCAGGGAGTGCAGAGAGTAAAATGTGAAAGAATCATC  4920
Sbjct  4911  ............................................................  4970

Query  4921  ATCTTAGATCCCACTGTTCCACCAACTTACAAACGGCCATGCATCAGACGGTACCCCTCC  4980
Sbjct  4971  ............................................................  5030

Query  4981  ACAATCTCTTGTAACTCCTCTGAGGACTCCAGGAGCTTGTCTACTTTTTCTGTCAGCTCC  5040
Sbjct  5031  ........C.......................................C...........  5090

Query  5041  GACTCCTCGATTGGTTCTCTGCCGGTCGGAGACACGAGACCCATTCCAGCCCCGAGGACC  5100
Sbjct  5091  .................CG..A...................T...........A......  5150

Query  5101  ATTTTCAGACCCGTCCCTGCCCCGAGAGCACCCGTGCTCAGAACCACACCGCCTCCTAAA  5160
Sbjct  5151  G...............................T..........T................  5210

Query  5161  CCACCGCGCACATTCACCGTGCGTGCAGAAGTGCACCAAGCACCCCCTACACCTGTACCT  5220
Sbjct  5211  ...................................T........................  5270

Query  5221  CCACCCAGACCGAAGAGGGCTGCAAAGTTGGCTCGTGAGATGCACCCCGGGTTCACCTTC  5280
Sbjct  5271  .....T......................................................  5330

Query  5281  GGGGACTTCGGAGAGCACGAGGTTGAGGAGCTTACGGCCTCTCCCTTAACCTTCGGAGAT  5340
Sbjct  5331  ..A..................A.................G....................  5390

Query  5341  TTTGCTGAAGGAGAGATCCAGGGGATGGGAGTGGAGTTTGAATGACTAGGCAGAGCCGGC  5400
Sbjct  5391  ....................A....C..................................  5450

Query  5401  GGGTACATTTTTTCGTCAGACACGGGTCCAGGCCACCTACAGCAGAGATCCGTTTTACAA  5460
Sbjct  5451  .........................................A.....G............  5510

Query  5461  AATTGCACGGCAGAATGTATCTACGAACCGGCAAAACTAGAAAAAATTCATGCACCAAAG  5520
Sbjct  5511  ............................................................  5570

Query  5521  TTGGATAAAACCAAGGAAGATATCTTAAGGAGCAAGTACCAAATGAAACCGTCTGAAGCA  5580
Sbjct  5571  ............................................................  5630
```

```
Query  5581  AACAAAAGCAGGTACCAATCTAGAAAAGTAGAAAATATGAAAGCAGAGATCGTAGGTAGA  5640
Sbjct  5631  ........T.........................................T.........  5690

Query  5641  CTCTTGGACGGACTGGGGGAGTATCTGGGCACCGAGCATCCAGTTGAATGCTACCGAATA  5700
Sbjct  5691  ..............A.............................................  5750

Query  5701  ACGTACCCGGTGCCTATATACTCAACTAGTGACCTCAGAGGTCTGTCTAGTGCCAAAACA  5760
Sbjct  5751  .....T....C...................AT..G.........................  5810

Query  5761  GCTGTTAGAGCTTGCAATGCATTTTTGGAAGCTAATTTTCCATCAGTCACTTCATATAAA  5820
Sbjct  5811  ............................................................  5870

Query  5821  ATTACTGATGAATACGACGCATACCTAGATATGGTAGATGGATCAGAGAGCTGTCTGGAC  5880
Sbjct  5871  ............................................................  5930

Query  5881  AGATCCTCCTTTTCGCCGTCTAGATTGCGTAGCTTTCCAAAAACACACTCATACTTGGAC  5940
Sbjct  5931  .................A...........C..............................  5990

Query  5941  CCACAGATCAACAGTGCGGTACCGTCACCATTCCAAAACACCTTACAAAATGTATTGGCA  6000
Sbjct  5991  ..G.........................................................  6050

Query  6001  GCGGCCACCAAAAGAAACTGTAATGTCACACAGATGAGAGAACTACCAACATATGATTCT  6060
Sbjct  6051  ...............................................G............  6110

Query  6061  GCAGTGCTAAATGTAGAGGCCTTCAGGAAATATGCGTGCAAGCCAGACGTATGGGATGAG  6120
Sbjct  6111  ............................................................  6170

Query  6121  TACAGGGATAATCCGATTTGCATAACCACCGAAAATGTCACCACTTACGTCGCCAAGTTG  6180
Sbjct  6171  ..............A...................................T..T......  6230

Query  6181  AAAGGACCGAAAGCTGCGGCCTTGTTTGCAAAAACACATAACCTGATACCACTACACCAA  6240
Sbjct  6231  ............................................................  6290

Query  6241  GTTCCTATGGACAAATTCACGGTAGATATGAAGAGAGATGTCAAAGTCACGCCCGGAACC  6300
Sbjct  6291  ............................................................  6350

Query  6301  AAGCACACCGAAGAGAGACCAAAGGTACAGGTGATTCAAGCGGCAGAGCCACTAGCCACT  6360
Sbjct  6351  ............................................................  6410

Query  6361  GCCTACCTCTGCGGAATTCACCGTGAATTGGTGCGCCGTCTCAACAACGCGCTTTTCCCA  6420
Sbjct  6411  .....................................................C.....T  6470

Query  6421  AACATCCACACTTTGTTTGATATGTCCGCAGAGGATTTCGATGCAATCATAGCGGAACAT  6480
Sbjct  6471  ..T...................................T.....................  6530

Query  6481  TTTAAGCACGGTGACCATGTGTTGGAAACGGATATAGCCTCTTTTGACAAAAGTCAAGAT  6540
Sbjct  6531  .................C..........................................  6590

Query  6541  GATTCCATGGCACTCACTGCGTTAATGATCCTTGAGGACCTGGGAGTAGACCAAAACCTA  6600
Sbjct  6591  ............................................................  6650

Query  6601  ATGAATTTGATAGAGGCTGCATTCGGGGAAATCGTGAGTACACACTTGCCCACAGGTACT  6660
Sbjct  6651  ............................................................  6710
```

```
Query  6661  AGATTCAAATTTGGAGCTATGATGAAGTCTGGAATGTTTTTGACGCTGTTCGTCAATACA  6720
Sbjct  6711  .......G....................C..............T..............  6770

Query  6721  ATTCTTAATGTGGTTATTGCGTGCCGAGTGTTGGAGGATCAATTGGCGCAGTCGCCGTGG  6780
Sbjct  6771  ........C..................................................C  6830

Query  6781  CCTGCTTTCATAGGAGATGACAACATAATCCATGGTATAATATCAGACAAATTGATGGCA  6840
Sbjct  6831  G..........................................................G  6890

Query  6841  GATAGATGTGCCACCTGGATGAACATGGAGGTCAAGATACTGGACTCTATAGTTGGAATA  6900
Sbjct  6891  ............................................T..C............  6950

Query  6901  CGGCCACCTTACTTCTGTGGAGGATTTATTGTATGTGACGATGTAACAGGTACAGCCTGC  6960
Sbjct  6951  ...........T................................................  7010

Query  6961  CGCGTCGCAGACCCACTGAAGAGATTGTTCAAGCTAGGTAAGCCATTGCCACTTGACGAT  7020
Sbjct  7011  .......................C....................................  7070

Query  7021  GGCCAAGATGAAGACAGAAGACGTGCATTACATGATGAAGTGAAAACCTGGTCGCGCGTA  7080
Sbjct  7071  ............................G...............................  7130

Query  7081  GGGCTGCGACACAGAGTGTGTGAAGCCATCGAAGACCGTTATGCCGTCCACTCATCAGAA  7140
Sbjct  7131  ..................................................T.........  7190

Query  7141  CTAGTTTTATTGGCACTGACTACTCTGTCTAAGAACTTGAAGTCCTTCAGAAACATAAGA  7200
Sbjct  7191  ............................................................  7250

Query  7201  GGGAAACCAATACATCTCTACGGTGGTCCTAAATAG  7236
Sbjct  7251  ....................................  7286
```

>Barmah Forest virus strain SW94245, complete genome
Sequence ID: MW835349.1 Length: 11500
Range 1: 57 to 7292

Score:12661 bits(6856), Expect:0.0,
Identities:7109/7236(98%),  Gaps:0/7236(0%), Strand: Plus/Plus

```
Query  1     ATGGCGAAACCAGTTGTGAAGATCGACGTGGAACCTGAAAGCCATTTCGCTAAGCAGGTC  60
Sbjct  57    ................................................T...........  116

Query  61    CAGAGTTGCTTCCCGCAGTTTGAGATCGAAGCAGTGCAGACCACACCAAACGATCATGCA  120
Sbjct  117   .........................................G..................  176

Query  121   CACGCGAGGGCGTTTTCGCACCTTGCTACGAAGCTCATAGAAATGGAGACAGCAAAAGAT  180
Sbjct  177   ............................................................  236

Query  181   CAGATCATCCTCGATATCGGAAGTGCACCCGCGAGGAGACTGTATTCAGAACACAAGTAC  240
Sbjct  237   ............................................................  296

Query  241   CACTGTGTTTGCCCAATGAAGTGCACGGAAGATCCAGAGAGAATGCTAGGATATGCACGT  300
Sbjct  297   ............................................................  356
```

```
Query  301   AAGTTGATCGCAGGCTCTGCGAAAGGGAAGGCAGAAAAGTTACGCGATCTCAGGGATGTC  360
Sbjct  357   ............................................................  416

Query  361   TTGGCTACGCCAGACATCGAGACGCAGTCGCTATGTCTCCACACAGACGCATCCTGCAGA  420
Sbjct  417   ............................................................  476

Query  421   TACCGCGGTGATGTTGCCGTGTATCAAGACGTGTATGCCATTGACGCACCTACCACGCTG  480
Sbjct  477   ..................................................C.........  536

Query  481   TACCACCAAGCGTTAAAGGGCGTCAGGACCGCATATTGGATAGGCTTTGATACAACGCCG  540
Sbjct  537   ...........................................................A  596

Query  541   TTCATGTACGATGCACTAGCAGGAGCTTACCCGCTCTACTCCACAAACTGGGCTGATGAG  600
Sbjct  597   .........A...........................................C......  656

Query  601   CAAGTGCTCGAGTCCAGAAACATTGGGCTATGTTCAGACAAAGTTTCTGAAGGGGGAAAG  660
Sbjct  657   ............................................................  716

Query  661   AAAGGGAGATCAATCCTCAGGAAGAAGTTCTTGAAGCAGTCAGACAGAGTCATGTTCTCT  720
Sbjct  717   ............................................................  776

Query  721   GTCGGCTCGACGTTGTATACGGAAAGCCGTAAATTACTGCAAAGTTGGCACCTGCCATCC  780
Sbjct  777   ............................................C...............  836

Query  781   ACATTCCATCTCAAAGGCAAATCTTCGTTCACGTGCCGCTGCGACACTATCGTCAGCTGC  840
Sbjct  837   ................................A..............C............  896

Query  841   GAAGGGTATGTTCTGAAGAAAATTACAATGTGTCCTGGAGTGACAGGCAAACCGATAGGA  900
Sbjct  897   ................................C..C........................  956

Query  901   TATGCCGTCACCCATCACAAAGAAGGATTCGTAGTCGGAAAAGTCACAGATACCATTCGC  960
Sbjct  957   ....................G.............................C.........  1016

Query  961   GGCGAGAGAGTCTCCTTCGCCGTGTGTACTTATGTACCAACAACACTCTGCGACCAGATG  1020
Sbjct  1017  ............................................................  1076

Query  1021  ACCGGGATCCTAGCAACAGAAGTAACAGCCGATGATGCCCAGAAACTGCTGGTGGGTTTG  1080
Sbjct  1077  ........T...................................................  1136

Query  1081  AACCAGAGAATAGTAGTTAATGGTAGGACCCAGAGAAATACCAATACTATGAAGAACTAC  1140
Sbjct  1137  ............................................C...............  1196

Query  1141  CTGCTACCACTGGTTGCACAAGCGCTAGCAAAATGGGCGAAGGAAGCAAAACAGGATATG  1200
Sbjct  1197  ......................................A.....................  1256

Query  1201  GAAGATGAAAGACCCCTGAACGAACGCCAACGAACGCTAACGTGCCTCTGCTGCTGGGCA  1260
Sbjct  1257  ............................................................  1316

Query  1261  TTTAAGCGAAACAAACGCCACGCCATTTACAAGAGACCAGACACACAGAGTATAGTCAAG  1320
Sbjct  1317  ............................................................  1376

Query  1321  GTCCCTTGCGAATTCACAAGCTTTCCTTTGGTCAGCCTGTGGTCCGCTGGGATGTCTATA  1380
Sbjct  1377  ............................................................  1436
```

```
Query  1381  TCTCTTAGGCAGAAGTTGAAGATGATGCTGCAGGCGAGGCAGCCCACACAAATAGCAGCA  1440
Sbjct  1437  ............................................................  1496

Query  1441  GTGACTGAGGAACTCATACAAGAAGCAGCTGCAGTAGAGCAAGAGGCCGTGGATACGGCC  1500
Sbjct  1497  ..................................C.........................  1556

Query  1501  AATGCCGAGCTGGACCACGCCGCATGGCCCTCCATTGTGGATACGACAGAGCGCCATGTT  1560
Sbjct  1557  ...............................................G............  1616

Query  1561  GAGGTCGAAGTGGAAGAACTCGACCAGCGTGCAGGGGAAGGGGTAGTGGAAACACCTCGA  1620
Sbjct  1617  ............................................................  1676

Query  1621  AACTCTATCAAAGTTTCAACACAGATCGGGGACGCGTTAATCGGCAGTTACCTGATCCTA  1680
Sbjct  1677  ........T........T..........................................  1736

Query  1681  TCACCCCAAGCAGTCCTACGCAGCGAAAAATTAGCCTGCATACATGATCTTGCAGAGCAG  1740
Sbjct  1737  ............................................................  1796

Query  1741  GTTAAGTTGGTCACACACTCTGGCCGTAGTGGTAGGTACGCCGTCGACAAATACNACGGA  1800
Sbjct  1797  ..C.................C..T..............................G.....  1856

Query  1801  AGAGTACTAGTCCCTACAGGAGTGGCTATAGACATTCAATCGTTCCAGGCTCTCAGTGAG  1860
Sbjct  1857  ...................................C........................  1916

Query  1861  AGCGCGACCCTTGTGTACAACGAACGCGAGTTCGTTAACAGGAAGCTGTGGCACATAGCA  1920
Sbjct  1917  ..T..........................................T..............  1976

Query  1921  GTATACGGGGCAGCACTCAATACTGATGAAGAAGGATACGAGAAGGTCCCGGTAGAGAGA  1980
Sbjct  1977  ............................................................  2036

Query  1981  GCAGAATCAGATTATGTGTTTGATGTAGACCAAAAAATGTGCCTaaaaaaaGAGCAGGCA  2040
Sbjct  2037  .................A............A.............C...............  2096

Query  2041  TCAGGTTGGGTACTCTGTGGCGAACTAGTCAACCCCCCATTCCACGAATTCGCATATGAA  2100
Sbjct  2097  .....A......................................................  2156

Query  2101  GGGCTCCGCACGAGACCGTCAGCACCCTACAAGGTTCATACAGTAGGTGTGTACGGAGTG  2160
Sbjct  2157  ........T..T................................................  2216

Query  2161  CCAGGATCAGGCAAATCCGCAATAATCAAGAACACGGTCACCATGTCTGACCTAGTATTG  2220
Sbjct  2217  ............................................................  2276

Query  2221  AGTGGTAAGAAAGAGAACTGCTTAGAAATTATGAACGATGTACTTAAACACAGAGCTCTA  2280
Sbjct  2277  ............................................................  2336

Query  2281  CGTATCACAGCGAAGACCGTAGACTCAGTGTTATTAAACGGCGTGAAACACACGCCTAAC  2340
Sbjct  2337  .....T.............................G........................  2396

Query  2341  ATACTATACATCGACGAAGCGTTCTCATGCCATGCAGGGACTCTGTTGGCCACTATAGCC  2400
Sbjct  2397  ............................................................  2456

Query  2401  ATAGTCAGGCCCAAACAGAAAGTGGTACTGTGCGGAGACCCGAAACAATGCGGATTCTTC  2460
Sbjct  2457  T...........................................................  2516
```

```
Query  2461  AATATGATGCAACTGAAAGTTAATTACAATCATGACATCTGCTCAGAAGTCTTCCACAAA  2520
Sbjct  2517  .......................C....................................  2576

Query  2521  AGTATCTCTAGACGGTGCACCCAGGATATCACGGCCATCGTTTCCAAATTACATTACCAG  2580
Sbjct  2577  ............................................T...............  2636

Query  2581  GACCGAATGAGGACCACAAACCCCGAAAAGGAGACATCATTATAGACACTACCGGCACT  2640
Sbjct  2637  ..................................................C.........  2696

Query  2641  ACCAAACCAGCCAAAACAGATCTGATTCTGACGTGCTTCAGGGGATGGGTGAAACAGTTG  2700
Sbjct  2697  ....................C.......................................  2756

Query  2701  CAGCAAGACTACAGAGGTAACGAAGTAATGACGGCTGCAGCGTCCCAAGGACTGACGAGG  2760
Sbjct  2757  ............................................................  2816

Query  2761  GCCTCCGTATATGCGGTTCGAACTAAAGTCAATGAGAACCCGCTATATGCACAGACCTCC  2820
Sbjct  2817  ............................................................  2876

Query  2821  GAGCACGTGAACGTGTTGTTAACACGCACAGAAAACAAGCTAGTATGGAAGACCTTGTCA  2880
Sbjct  2877  .......................................T....................  2936

Query  2881  ACAGATCCCTGGATTAAAACACTGACTAACCCACCTAGAGGGCACTATACCGCCACCATA  2940
Sbjct  2937  ............................................................  2996

Query  2941  GCAGAATGGGAAGCGGAACACCAGGGTATAATGAAGGCCATACAAGGGTATGCACCGCCC  3000
Sbjct  2997  ..............A.............................................  3056

Query  3001  GTGAACACCTTCATGAACAAAGTAAATGTGTGCTGGGCAAAGACACTTACGCCTGTGCTG  3060
Sbjct  3057  ...........T................................G..C..A.........  3116

Query  3061  GAAACTGCGGGTATCTCCCTGTCAGCAGAAGACTGGTCTGAACTGCTGCCCCCGTTTGCC  3120
Sbjct  3117  ............................................................  3176

Query  3121  CAGGACGTGGCGTACTCACCCGAGGTGGCATTAAACATCATATGCACGAAAATGTATGGG  3180
Sbjct  3177  ...............................................A............  3236

Query  3181  TTTGACTTAGACACTGGTCTTTTTCCAGGCCATCAGTGCCAATGACATACACCAAAGAC  3240
Sbjct  3237  ........G.....C.............A.........A.....................  3296

Query  3241  CATTGGGATAACAGAGTTGGAGGGAAAATGTATGGATTCAGCCAACAAGCATACGATCAG  3300
Sbjct  3297  ............................................................  3356

Query  3301  CTGGCAAGACGACATCCGTACCTTCGAGGTAGAGAGAAATCAGGAATGCAGATCGTAGTC  3360
Sbjct  3357  ...................................A........................  3416

Query  3361  ACTGAAATGCGTATCCAGCGCCCAAGATCGGATGCCAACATCATCCCGATCAACCGCAGG  3420
Sbjct  3417  .....G......................................................  3476

Query  3421  CTCCCTCACTCACTCGTAGCCACACACGAGTATAGGCGAGCTGCACGGGCCGAGGAATTC  3480
Sbjct  3477  ............................................G...............  3536

Query  3481  TTCACCACGACACGAGGGTACACTATGCTGCTGGTCTCTGAGTATAACATGAACTTACCA  3540
Sbjct  3537  ..........T.................................................  3596
```

```
Query  3541  AACAAGAAGATCACCTGGCTGGCTCCGATAGGGACGCAGGGGGCCCATCACACCGCCAAC  3600
Sbjct  3597  ............................................................  3656

Query  3601  CTAAACTTGGGGATACCACCTCTGCTGGGCAGTTTTGATGCGGTGGTTGTGAACATGCCG  3660
Sbjct  3657  ........................................T...................  3716

Query  3661  ACTCCATTCCGGAACCATCACTACCAGCAATGTGAAGACCACGCGATGAAACTCCAGATG  3720
Sbjct  3717  ................................C...........................  3776

Query  3721  CTGGCAGGCGACGCACTGAGGCACATTAAACCTGGCGGATCATTGTGGGTCAAGGCATAC  3780
Sbjct  3777  .............................G..............................  3836

Query  3781  GGCTACGCAGACCGGCACAGCGAGCACGTGGTCTTGGCATTGGCTAGAAAGTTTAAAAGC  3840
Sbjct  3837  .......................................C....................  3896

Query  3841  TTCAGAGTCACACAACCCTCATGCGTGACTTCCAACACCGAGGTGTTTCTCCACTTCTCA  3900
Sbjct  3897  ...........G........G.....A.................................  3956

Query  3901  ATTTTTGACAATGGCAAACGCGCGATAGCCCTGCATTCAGCTAATAGGAAGGCTAACAGT  3960
Sbjct  3957  ............................................................  4016

Query  3961  ATCTTCCAAAACACCTTCTTACCGGCGGGCAGTGCACCGGCGTACAGAGTCAAACGTGGA  4020
Sbjct  4017  ..........................A....A............................  4076

Query  4021  GACATTTCGAACGCCCCAGAGGATGCAGTGGTCAATGCAGCAAACCAACAGGGAGTGAAG  4080
Sbjct  4077  ............................................................  4136

Query  4081  GGTGCTGGAGTTTGCGGTGCAATTTACCGTAAGTGGCCGGACGCTTTCGGTGATGTCGCT  4140
Sbjct  4137  ............................................................  4196

Query  4141  ACTCCAACCGGAACAGCAGTTTCGAAATCCGTCCAAGATAAATTGGTGATCCACGCTGTC  4200
Sbjct  4197  ............................................................  4256

Query  4201  GGCCCGAATTTCTCAAAATGTTCAGAAGAGGAAGGGGACAGAGACCTAGCATCTGCTTAC  4260
Sbjct  4257  ...........T................................................  4316

Query  4261  AGAGCTGCAGCAGAAATAGTGATGGATaaaaaaaTTACAACAGTGGCCGTCCCCTTACTC  4320
Sbjct  4317  ..........................A...........................C.....  4376

Query  4321  TCCACCGGCATTTATGCCGGAGGAAAAAACAGAGTAGAACAGTCACTCAACCATCTCTTC  4380
Sbjct  4377  ..............C........G....................................  4436

Query  4381  ACGGCATTCGACAATACTGATGCAGATGTGACCATATATTGCATGGACAAAACATGGGAA  4440
Sbjct  4437  ........T...................................................  4496

Query  4441  AAGAAGATTAAGGAGGCAATCGATCACCGGACTTCGGTTGAGATGGTGCAGGATGACGTG  4500
Sbjct  4497  ............................................................  4556

Query  4501  CAGTTGGAGGAGGAACTGGTACGAGTACACCCTTTGAGTAGTTTAGCAGGTAGGAAGGGT  4560
Sbjct  4557  ......................................C.....................  4616

Query  4561  TACAGTACGGACAGCGGCCGAGTGTTTTCCTACCTGGAAGGTACCAAATTCCATCAGACT  4620
Sbjct  4617  ............................................................  4676
```

```
Query  4621  GCGGTGGACATAGCCGAAATGCAAGTGCTGTGGCCCGCCCTCAAAGAGTCTAATGAGCAA  4680
Sbjct  4677  ........T.............................T.....................  4736

Query  4681  ATAGTGGCATACACCTTAGGAGAATCAATGGACCAGATACGTGGCAAGTGCCCGACAGAA  4740
Sbjct  4737  ............................................................  4796

Query  4741  GATACTGACGCCTCCACACCTCCACGGACTGTGCCGTGCCTCTGTCGATACGCCATGACA  4800
Sbjct  4797  ............................................................  4856

Query  4801  CCAGAGAGAGTGTACCGACTTAAATGCACGAACACTACCCAATTTACGGTTTGCTCATCT  4860
Sbjct  4857  ............................................C.....C.........  4916

Query  4861  TTTGAGTTGCCAAAGTATCACATTCAGGGAGTGCAGAGAGTAAAATGTGAAAGAATCATC  4920
Sbjct  4917  ............................................................  4976

Query  4921  ATCTTAGATCCCACTGTTCCACCAACTTACAAACGGCCATGCATCAGACGGTACCCCTCC  4980
Sbjct  4977  ............................................................  5036

Query  4981  ACAATCTCTTGTAACTCCTCTGAGGACTCCAGGAGCTTGTCTACTTTTTCTGTCAGCTCC  5040
Sbjct  5037  ........C......................................C............  5096

Query  5041  GACTCCTCGATTGGTTCTCTGCCGGTCGGAGACACGAGACCCATTCCAGCCCCGAGGACC  5100
Sbjct  5097  .................CG..A...................T...........A......  5156

Query  5101  ATTTTCAGACCCGTCCCTGCCCCGAGAGCACCCGTGCTCAGAACCACACCGCCTCCTAAA  5160
Sbjct  5157  G...............................T..........T................  5216

Query  5161  CCACCGCGCACATTCACCGTGCGTGCAGAAGTGCACCAAGCACCCCCTACACCTGTACCT  5220
Sbjct  5217  ...................................T........................  5276

Query  5221  CCACCCAGACCGAAGAGGGCTGCAAAGTTGGCTCGTGAGATGCACCCCGGGTTCACCTTC  5280
Sbjct  5277  .....T......................................................  5336

Query  5281  GGGGACTTCGGAGAGCACGAGGTTGAGGAGCTTACGGCCTCTCCCTTAACCTTCGGAGAT  5340
Sbjct  5337  ..A..................A.................G....................  5396

Query  5341  TTTGCTGAAGGAGAGATCCAGGGGATGGGAGTGGAGTTTGAATGACTAGGCAGAGCCGGC  5400
Sbjct  5397  ....................A....C..................................  5456

Query  5401  GGGTACATTTTTTCGTCAGACACGGGTCCAGGCCACCTACAGCAGAGATCCGTTTTACAA  5460
Sbjct  5457  .........................................A.....G............  5516

Query  5461  AATTGCACGGCAGAATGTATCTACGAACCGGCAAAACTAGAAAAAATTCATGCACCAAAG  5520
Sbjct  5517  ............................................................  5576

Query  5521  TTGGATAAAACCAAGGAAGATATCTTAAGGAGCAAGTACCAAATGAAACCGTCTGAAGCA  5580
Sbjct  5577  ............................................................  5636

Query  5581  AACAAAAGCAGGTACCAATCTAGAAAAGTAGAAAATATGAAAGCAGAGATCGTAGGTAGA  5640
Sbjct  5637  ........T.........................................T.........  5696

Query  5641  CTCTTGGACGGACTGGGGGAGTATCTGGGCACCGAGCATCCAGTTGAATGCTACCGAATA  5700
Sbjct  5697  ..............A.............................................  5756
```

```
Query  5701  ACGTACCCGGTGCCTATATACTCAACTAGTGACCTCAGAGGTCTGTCTAGTGCCAAAACA  5760
Sbjct  5757  .....T....C...................AT..G.........................  5816

Query  5761  GCTGTTAGAGCTTGCAATGCATTTTTGGAAGCTAATTTTCCATCAGTCACTTCATATAAA  5820
Sbjct  5817  ............................................................  5876

Query  5821  ATTACTGATGAATACGACGCATACCTAGATATGGTAGATGGATCAGAGAGCTGTCTGGAC  5880
Sbjct  5877  ............................................................  5936

Query  5881  AGATCCTCCTTTTCGCCGTCTAGATTGCGTAGCTTTCCAAAAACACACTCATACTTGGAC  5940
Sbjct  5937  .................A...........C..............................  5996

Query  5941  CCACAGATCAACAGTGCGGTACCGTCACCATTCCAAAACACCTTACAAAATGTATTGGCA  6000
Sbjct  5997  ..G.........................................................  6056

Query  6001  GCGGCCACCAAAAGAAACTGTAATGTCACACAGATGAGAGAACTACCAACATATGATTCT  6060
Sbjct  6057  ...............................................G............  6116

Query  6061  GCAGTGCTAAATGTAGAGGCCTTCAGGAAATATGCGTGCAAGCCAGACGTATGGGATGAG  6120
Sbjct  6117  ............................................................  6176

Query  6121  TACAGGGATAATCCGATTTGCATAACCACCGAAAATGTCACCACTTACGTCGCCAAGTTG  6180
Sbjct  6177  ..............A...................................T..T......  6236

Query  6181  AAAGGACCGAAAGCTGCGGCCTTGTTTGCAAAAACACATAACCTGATACCACTACACCAA  6240
Sbjct  6237  ............................................................  6296

Query  6241  GTTCCTATGGACAAATTCACGGTAGATATGAAGAGAGATGTCAAAGTCACGCCCGGAACC  6300
Sbjct  6297  ............................................................  6356

Query  6301  AAGCACACCGAAGAGAGACCAAAGGTACAGGTGATTCAAGCGGCAGAGCCACTAGCCACT  6360
Sbjct  6357  ............................................................  6416

Query  6361  GCCTACCTCTGCGGAATTCACCGTGAATTGGTGCGCCGTCTCAACAACGCGCTTTTCCCA  6420
Sbjct  6417  .....................................................C.....T  6476

Query  6421  AACATCCACACTTTGTTTGATATGTCCGCAGAGGATTTCGATGCAATCATAGCGGAACAT  6480
Sbjct  6477  ..T...................................T.....................  6536

Query  6481  TTTAAGCACGGTGACCATGTGTTGGAAACGGATATAGCCTCTTTTGACAAAAGTCAAGAT  6540
Sbjct  6537  .................C..........................................  6596

Query  6541  GATTCCATGGCACTCACTGCGTTAATGATCCTTGAGGACCTGGGAGTAGACCAAAACCTA  6600
Sbjct  6597  ............................................................  6656

Query  6601  ATGAATTTGATAGAGGCTGCATTCGGGGAAATCGTGAGTACACACTTGCCCACAGGTACT  6660
Sbjct  6657  ............................................................  6716

Query  6661  AGATTCAAATTTGGAGCTATGATGAAGTCTGGAATGTTTTTGACGCTGTTCGTCAATACA  6720
Sbjct  6717  .......G.....................C...............T..............  6776

Query  6721  ATTCTTAATGTGGTTATTGCGTGCCGAGTGTTGGAGGATCAATTGGCGCAGTCGCCGTGG  6780
Sbjct  6777  ........C..................................................C  6836
```

```
Query  6781   CCTGCTTTCATAGGAGATGACAACATAATCCATGGTATAATATCAGACAAATTGATGGCA  6840
Sbjct  6837   G..........................................................G  6896

Query  6841   GATAGATGTGCCACCTGGATGAACATGGAGGTCAAGATACTGGACTCTATAGTTGGAATA  6900
Sbjct  6897   ............................................T..C............  6956

Query  6901   CGGCCACCTTACTTCTGTGGAGGATTTATTGTATGTGACGATGTAACAGGTACAGCCTGC  6960
Sbjct  6957   ...........T................................................  7016

Query  6961   CGCGTCGCAGACCCACTGAAGAGATTGTTCAAGCTAGGTAAGCCATTGCCACTTGACGAT  7020
Sbjct  7017   ........................C...................................  7076

Query  7021   GGCCAAGATGAAGACAGAAGACGTGCATTACATGATGAAGTGAAAACCTGGTCGCGCGTA  7080
Sbjct  7077   .............................G..............................  7136

Query  7081   GGGCTGCGACACAGAGTGTGTGAAGCCATCGAAGACCGTTATGCCGTCCACTCATCAGAA  7140
Sbjct  7137   ..................................................T.........  7196

Query  7141   CTAGTTTTATTGGCACTGACTACTCTGTCTAAGAACTTGAAGTCCTTCAGAAACATAAGA  7200
Sbjct  7197   ............................................................  7256

Query  7201   GGGAAACCAATACATCTCTACGGTGGTCCTAAATAG  7236
Sbjct  7257   ....................................  7292
```

>Barmah Forest virus isolate SW94393, complete genome
Sequence ID: MN689043.1 Length: 11493
Range 1: 54 to 7289

Score:12661 bits(6856), Expect:0.0,
Identities:7109/7236(98%), Gaps:0/7236(0%), Strand: Plus/Plus

```
Query  1      ATGGCGAAACCAGTTGTGAAGATCGACGTGGAACCTGAAAGCCATTTCGCTAAGCAGGTC  60
Sbjct  54     ...............................................T............  113

Query  61     CAGAGTTGCTTCCCGCAGTTTGAGATCGAAGCAGTGCAGACCACACCAAACGATCATGCA  120
Sbjct  114    .........................................G..................  173

Query  121    CACGCGAGGGCGTTTTCGCACCTTGCTACGAAGCTCATAGAAATGGAGACAGCAAAAGAT  180
Sbjct  174    ............................................................  233

Query  181    CAGATCATCCTCGATATCGGAAGTGCACCCGCGAGGAGACTGTATTCAGAACACAAGTAC  240
Sbjct  234    ............................................................  293

Query  241    CACTGTGTTTGCCCAATGAAGTGCACGGAAGATCCAGAGAGAATGCTAGGATATGCACGT  300
Sbjct  294    ............................................................  353

Query  301    AAGTTGATCGCAGGCTCTGCGAAAGGGAAGGCAGAAAAGTTACGCGATCTCAGGGATGTC  360
Sbjct  354    ............................................................  413

Query  361    TTGGCTACGCCAGACATCGAGACGCAGTCGCTATGTCTCCACACAGACGCATCCTGCAGA  420
```

```
Sbjct  414   ............................................................  473

Query  421   TACCGCGGTGATGTTGCCGTGTATCAAGACGTGTATGCCATTGACGCACCTACCACGCTG  480
Sbjct  474   ..................................................C.........  533

Query  481   TACCACCAAGCGTTAAAGGGCGTCAGGACCGCATATTGGATAGGCTTTGATACAACGCCG  540
Sbjct  534   ...........................................................A  593

Query  541   TTCATGTACGATGCACTAGCAGGAGCTTACCCGCTCTACTCCACAAACTGGGCTGATGAG  600
Sbjct  594   .........A...........................................C......  653

Query  601   CAAGTGCTCGAGTCCAGAAACATTGGGCTATGTTCAGACAAAGTTTCTGAAGGGGGAAAG  660
Sbjct  654   ............................................................  713

Query  661   AAAGGGAGATCAATCCTCAGGAAGAAGTTCTTGAAGCAGTCAGACAGAGTCATGTTCTCT  720
Sbjct  714   ............................................................  773

Query  721   GTCGGCTCGACGTTGTATACGGAAAGCCGTAAATTACTGCAAAGTTGGCACCTGCCATCC  780
Sbjct  774   ............................................C...............  833

Query  781   ACATTCCATCTCAAAGGCAAATCTTCGTTCACGTGCCGCTGCGACACTATCGTCAGCTGC  840
Sbjct  834   ................................A..............C............  893

Query  841   GAAGGGTATGTTCTGAAGAAAATTACAATGTGTCCTGGAGTGACAGGCAAACCGATAGGA  900
Sbjct  894   ................................C..C........................  953

Query  901   TATGCCGTCACCCATCACAAAGAAGGATTCGTAGTCGGAAAAGTCACAGATACCATTCGC  960
Sbjct  954   ....................G.............................C.........  1013

Query  961   GGCGAGAGAGTCTCCTTCGCCGTGTGTACTTATGTACCAACAACACTCTGCGACCAGATG  1020
Sbjct  1014  ............................................................  1073

Query  1021  ACCGGGATCCTAGCAACAGAAGTAACAGCCGATGATGCCCAGAAACTGCTGGTGGGTTTG  1080
Sbjct  1074  ........T...................................................  1133

Query  1081  AACCAGAGAATAGTAGTTAATGGTAGGACCCAGAGAAATACCAATACTATGAAGAACTAC  1140
Sbjct  1134  ............................................C...............  1193

Query  1141  CTGCTACCACTGGTTGCACAAGCGCTAGCAAAATGGGCGAAGGAAGCAAAACAGGATATG  1200
Sbjct  1194  ......................................A.....................  1253

Query  1201  GAAGATGAAAGACCCCTGAACGAACGCCAACGAACGCTAACGTGCCTCTGCTGCTGGGCA  1260
Sbjct  1254  ............................................................  1313

Query  1261  TTTAAGCGAAACAAACGCCACGCCATTTACAAGAGACCAGACACACAGAGTATAGTCAAG  1320
Sbjct  1314  ............................................................  1373

Query  1321  GTCCCTTGCGAATTCACAAGCTTTCCTTTGGTCAGCCTGTGGTCCGCTGGGATGTCTATA  1380
Sbjct  1374  ............................................................  1433

Query  1381  TCTCTTAGGCAGAAGTTGAAGATGATGCTGCAGGCGAGGCAGCCCACACAAATAGCAGCA  1440
Sbjct  1434  ............................................................  1493

Query  1441  GTGACTGAGGAACTCATACAAGAAGCAGCTGCAGTAGAGCAAGAGGCCGTGGATACGGCC  1500
```

```
Sbjct  1494  ..................................C.........................  1553

Query  1501  AATGCCGAGCTGGACCACGCCGCATGGCCCTCCATTGTGGATACGACAGAGCGCCATGTT  1560
Sbjct  1554  ...............................................G............  1613

Query  1561  GAGGTCGAAGTGGAAGAACTCGACCAGCGTGCAGGGGAAGGGGTAGTGGAAACACCTCGA  1620
Sbjct  1614  ............................................................  1673

Query  1621  AACTCTATCAAAGTTTCAACACAGATCGGGGACGCGTTAATCGGCAGTTACCTGATCCTA  1680
Sbjct  1674  ........T........T..........................................  1733

Query  1681  TCACCCCAAGCAGTCCTACGCAGCGAAAAATTAGCCTGCATACATGATCTTGCAGAGCAG  1740
Sbjct  1734  ............................................................  1793

Query  1741  GTTAAGTTGGTCACACACTCTGGCCGTAGTGGTAGGTACGCCGTCGACAAATACNACGGA  1800
Sbjct  1794  ..C.................C..T..............................G.....  1853

Query  1801  AGAGTACTAGTCCCTACAGGAGTGGCTATAGACATTCAATCGTTCCAGGCTCTCAGTGAG  1860
Sbjct  1854  ...................................C........................  1913

Query  1861  AGCGCGACCCTTGTGTACAACGAACGCGAGTTCGTTAACAGGAAGCTGTGGCACATAGCA  1920
Sbjct  1914  ..T..........................................T..............  1973

Query  1921  GTATACGGGGCAGCACTCAATACTGATGAAGAAGGATACGAGAAGGTCCCGGTAGAGAGA  1980
Sbjct  1974  ............................................................  2033

Query  1981  GCAGAATCAGATTATGTGTTTGATGTAGACCAAAAAATGTGCCTaaaaaaaGAGCAGGCA  2040
Sbjct  2034  .................A............A.............C...............  2093

Query  2041  TCAGGTTGGGTACTCTGTGGCGAACTAGTCAACCCCCCATTCCACGAATTCGCATATGAA  2100
Sbjct  2094  .....A......................................................  2153

Query  2101  GGGCTCCGCACGAGACCGTCAGCACCCTACAAGGTTCATACAGTAGGTGTGTACGGAGTG  2160
Sbjct  2154  ........T..T................................................  2213

Query  2161  CCAGGATCAGGCAAATCCGCAATAATCAAGAACACGGTCACCATGTCTGACCTAGTATTG  2220
Sbjct  2214  ............................................................  2273

Query  2221  AGTGGTAAGAAAGAGAACTGCTTAGAAATTATGAACGATGTACTTAAACACAGAGCTCTA  2280
Sbjct  2274  ............................................................  2333

Query  2281  CGTATCACAGCGAAGACCGTAGACTCAGTGTTATTAAACGGCGTGAAACACACGCCTAAC  2340
Sbjct  2334  .....T.............................G........................  2393

Query  2341  ATACTATACATCGACGAAGCGTTCTCATGCCATGCAGGGACTCTGTTGGCCACTATAGCC  2400
Sbjct  2394  ............................................................  2453

Query  2401  ATAGTCAGGCCCAAACAGAAAGTGGTACTGTGCGGAGACCCGAAACAATGCGGATTCTTC  2460
Sbjct  2454  T...........................................................  2513

Query  2461  AATATGATGCAACTGAAAGTTAATTACAATCATGACATCTGCTCAGAAGTCTTCCACAAA  2520
Sbjct  2514  .......................C....................................  2573

Query  2521  AGTATCTCTAGACGGTGCACCCAGGATATCACGGCCATCGTTTCCAAATTACATTACCAG  2580
```

```
Sbjct  2574  ............................................T...............  2633

Query  2581  GACCGAATGAGGACCACAAACCCCCGAAAAGGAGACATCATTATAGACACTACCGGCACT  2640
Sbjct  2634  ..................................................C.........  2693

Query  2641  ACCAAACCAGCCAAAACAGATCTGATTCTGACGTGCTTCAGGGGATGGGTGAAACAGTTG  2700
Sbjct  2694  ....................C.......................................  2753

Query  2701  CAGCAAGACTACAGAGGTAACGAAGTAATGACGGCTGCAGCGTCCCAAGGACTGACGAGG  2760
Sbjct  2754  ............................................................  2813

Query  2761  GCCTCCGTATATGCGGTTCGAACTAAAGTCAATGAGAACCCGCTATATGCACAGACCTCC  2820
Sbjct  2814  ............................................................  2873

Query  2821  GAGCACGTGAACGTGTTGTTAACACGCACAGAAAACAAGCTAGTATGGAAGACCTTGTCA  2880
Sbjct  2874  .......................................T....................  2933

Query  2881  ACAGATCCCTGGATTAAAACACTGACTAACCCACCTAGAGGGCACTATACCGCCACCATA  2940
Sbjct  2934  ............................................................  2993

Query  2941  GCAGAATGGGAAGCGGAACACCAGGGTATAATGAAGGCCATACAAGGGTATGCACCGCCC  3000
Sbjct  2994  ..............A.............................................  3053

Query  3001  GTGAACACCTTCATGAACAAAGTAAATGTGTGCTGGGCAAAGACACTTACGCCTGTGCTG  3060
Sbjct  3054  ...........T................................G..C..A.........  3113

Query  3061  GAAACTGCGGGTATCTCCCTGTCAGCAGAAGACTGGTCTGAACTGCTGCCCCCGTTTGCC  3120
Sbjct  3114  ............................................................  3173

Query  3121  CAGGACGTGGCGTACTCACCCGAGGTGGCATTAAACATCATATGCACGAAAATGTATGGG  3180
Sbjct  3174  ...............................................A............  3233

Query  3181  TTTGACTTAGACACTGGTCTTTTTTCCAGGCCATCAGTGCCAATGACATACACCAAAGAC  3240
Sbjct  3234  ........G.....C.............A.........A.....................  3293

Query  3241  CATTGGGATAACAGAGTTGGAGGGAAAATGTATGGATTCAGCCAACAAGCATACGATCAG  3300
Sbjct  3294  ............................................................  3353

Query  3301  CTGGCAAGACGACATCCGTACCTTCGAGGTAGAGAGAAATCAGGAATGCAGATCGTAGTC  3360
Sbjct  3354  ...................................A........................  3413

Query  3361  ACTGAAATGCGTATCCAGCGCCCAAGATCGGATGCCAACATCATCCCGATCAACCGCAGG  3420
Sbjct  3414  .....G......................................................  3473

Query  3421  CTCCCTCACTCACTCGTAGCCACACACGAGTATAGGCGAGCTGCACGGGCCGAGGAATTC  3480
Sbjct  3474  ............................................G...............  3533

Query  3481  TTCACCACGACACGAGGGTACACTATGCTGCTGGTCTCTGAGTATAACATGAACTTACCA  3540
Sbjct  3534  ..........T.................................................  3593

Query  3541  AACAAGAAGATCACCTGGCTGGCTCCGATAGGGACGCAGGGGGCCCATCACACCGCCAAC  3600
Sbjct  3594  ............................................................  3653

Query  3601  CTAAACTTGGGGATACCACCTCTGCTGGGCAGTTTTGATGCGGTGGTTGTGAACATGCCG  3660
```

```
Sbjct  3654  ........................................T...................  3713

Query  3661  ACTCCATTCCGGAACCATCACTACCAGCAATGTGAAGACCACGCGATGAAACTCCAGATG  3720
Sbjct  3714  ................................C...........................  3773

Query  3721  CTGGCAGGCGACGCACTGAGGCACATTAAACCTGGCGGATCATTGTGGGTCAAGGCATAC  3780
Sbjct  3774  .............................G..............................  3833

Query  3781  GGCTACGCAGACCGGCACAGCGAGCACGTGGTCTTGGCATTGGCTAGAAAGTTTAAAAGC  3840
Sbjct  3834  .......................................C....................  3893

Query  3841  TTCAGAGTCACACAACCCTCATGCGTGACTTCCAACACCGAGGTGTTTCTCCACTTCTCA  3900
Sbjct  3894  ...........G........G.....A.................................  3953

Query  3901  ATTTTTGACAATGGCAAACGCGCGATAGCCCTGCATTCAGCTAATAGGAAGGCTAACAGT  3960
Sbjct  3954  ............................................................  4013

Query  3961  ATCTTCCAAAACACCTTCTTACCGGCGGGCAGTGCACCGGCGTACAGAGTCAAACGTGGA  4020
Sbjct  4014  ..........................A....A............................  4073

Query  4021  GACATTTCGAACGCCCCAGAGGATGCAGTGGTCAATGCAGCAAACCAACAGGGAGTGAAG  4080
Sbjct  4074  ............................................................  4133

Query  4081  GGTGCTGGAGTTTGCGGTGCAATTTACCGTAAGTGGCCGGACGCTTTCGGTGATGTCGCT  4140
Sbjct  4134  ............................................................  4193

Query  4141  ACTCCAACCGGAACAGCAGTTTCGAAATCCGTCCAAGATAAATTGGTGATCCACGCTGTC  4200
Sbjct  4194  ............................................................  4253

Query  4201  GGCCCGAATTTCTCAAAATGTTCAGAAGAGGAAGGGGACAGAGACCTAGCATCTGCTTAC  4260
Sbjct  4254  ...........T................................................  4313

Query  4261  AGAGCTGCAGCAGAAATAGTGATGGATaaaaaaaTTACAACAGTGGCCGTCCCCTTACTC  4320
Sbjct  4314  ..........................A...........................C.....  4373

Query  4321  TCCACCGGCATTTATGCCGGAGGAAAAAACAGAGTAGAACAGTCACTCAACCATCTCTTC  4380
Sbjct  4374  ..............C........G....................................  4433

Query  4381  ACGGCATTCGACAATACTGATGCAGATGTGACCATATATTGCATGGACAAAACATGGGAA  4440
Sbjct  4434  ........T...................................................  4493

Query  4441  AAGAAGATTAAGGAGGCAATCGATCACCGGACTTCGGTTGAGATGGTGCAGGATGACGTG  4500
Sbjct  4494  ............................................................  4553

Query  4501  CAGTTGGAGGAGGAACTGGTACGAGTACACCCTTTGAGTAGTTTAGCAGGTAGGAAGGGT  4560
Sbjct  4554  ......................................C.....................  4613

Query  4561  TACAGTACGGACAGCGGCCGAGTGTTTTCCTACCTGGAAGGTACCAAATTCCATCAGACT  4620
Sbjct  4614  ............................................................  4673

Query  4621  GCGGTGGACATAGCCGAAATGCAAGTGCTGTGGCCCGCCCTCAAAGAGTCTAATGAGCAA  4680
Sbjct  4674  ........T.............................T.....................  4733

Query  4681  ATAGTGGCATACACCTTAGGAGAATCAATGGACCAGATACGTGGCAAGTGCCCGACAGAA  4740
```

```
Sbjct  4734  ............................................................  4793

Query  4741  GATACTGACGCCTCCACACCTCCACGGACTGTGCCGTGCCTCTGTCGATACGCCATGACA  4800
Sbjct  4794  ............................................................  4853

Query  4801  CCAGAGAGAGTGTACCGACTTAAATGCACGAACACTACCCAATTTACGGTTTGCTCATCT  4860
Sbjct  4854  ............................................C.....C.........  4913

Query  4861  TTTGAGTTGCCAAAGTATCACATTCAGGGAGTGCAGAGAGTAAAATGTGAAAGAATCATC  4920
Sbjct  4914  ............................................................  4973

Query  4921  ATCTTAGATCCCACTGTTCCACCAACTTACAAACGGCCATGCATCAGACGGTACCCCTCC  4980
Sbjct  4974  ............................................................  5033

Query  4981  ACAATCTCTTGTAACTCCTCTGAGGACTCCAGGAGCTTGTCTACTTTTTCTGTCAGCTCC  5040
Sbjct  5034  ........C......................................C............  5093

Query  5041  GACTCCTCGATTGGTTCTCTGCCGGTCGGAGACACGAGACCCATTCCAGCCCCGAGGACC  5100
Sbjct  5094  .................CG..A...................T...........A......  5153

Query  5101  ATTTTCAGACCCGTCCCTGCCCCGAGAGCACCCGTGCTCAGAACCACACCGCCTCCTAAA  5160
Sbjct  5154  G...............................T..........T................  5213

Query  5161  CCACCGCGCACATTCACCGTGCGTGCAGAAGTGCACCAAGCACCCCCTACACCTGTACCT  5220
Sbjct  5214  ...................................T........................  5273

Query  5221  CCACCCAGACCGAAGAGGGCTGCAAAGTTGGCTCGTGAGATGCACCCCGGGTTCACCTTC  5280
Sbjct  5274  .....T......................................................  5333

Query  5281  GGGGACTTCGGAGAGCACGAGGTTGAGGAGCTTACGGCCTCTCCCTTAACCTTCGGAGAT  5340
Sbjct  5334  ..A..................A.................G....................  5393

Query  5341  TTTGCTGAAGGAGAGATCCAGGGGATGGGAGTGGAGTTTGAATGACTAGGCAGAGCCGGC  5400
Sbjct  5394  ....................A....C..................................  5453

Query  5401  GGGTACATTTTTTCGTCAGACACGGGTCCAGGCCACCTACAGCAGAGATCCGTTTTACAA  5460
Sbjct  5454  .........................................A.....G............  5513

Query  5461  AATTGCACGGCAGAATGTATCTACGAACCGGCAAAACTAGAAAAAATTCATGCACCAAAG  5520
Sbjct  5514  ............................................................  5573

Query  5521  TTGGATAAAACCAAGGAAGATATCTTAAGGAGCAAGTACCAAATGAAACCGTCTGAAGCA  5580
Sbjct  5574  ............................................................  5633

Query  5581  AACAAAAGCAGGTACCAATCTAGAAAAGTAGAAAATATGAAAGCAGAGATCGTAGGTAGA  5640
Sbjct  5634  ........T.........................................T.........  5693

Query  5641  CTCTTGGACGGACTGGGGGAGTATCTGGGCACCGAGCATCCAGTTGAATGCTACCGAATA  5700
Sbjct  5694  ..............A.............................................  5753

Query  5701  ACGTACCCGGTGCCTATATACTCAACTAGTGACCTCAGAGGTCTGTCTAGTGCCAAAACA  5760
Sbjct  5754  .....T....C...................AT..G.........................  5813

Query  5761  GCTGTTAGAGCTTGCAATGCATTTTTGGAAGCTAATTTTCCATCAGTCACTTCATATAAA  5820
```

```
Sbjct  5814  ............................................................  5873

Query  5821  ATTACTGATGAATACGACGCATACCTAGATATGGTAGATGGATCAGAGAGCTGTCTGGAC  5880
Sbjct  5874  ............................................................  5933

Query  5881  AGATCCTCCTTTTCGCCGTCTAGATTGCGTAGCTTTCCAAAAACACACTCATACTTGGAC  5940
Sbjct  5934  .................A...........C..............................  5993

Query  5941  CCACAGATCAACAGTGCGGTACCGTCACCATTCCAAAACACCTTACAAAATGTATTGGCA  6000
Sbjct  5994  ..G.........................................................  6053

Query  6001  GCGGCCACCAAAAGAAACTGTAATGTCACACAGATGAGAGAACTACCAACATATGATTCT  6060
Sbjct  6054  ...............................................G............  6113

Query  6061  GCAGTGCTAAATGTAGAGGCCTTCAGGAAATATGCGTGCAAGCCAGACGTATGGGATGAG  6120
Sbjct  6114  ............................................................  6173

Query  6121  TACAGGGATAATCCGATTTGCATAACCACCGAAAATGTCACCACTTACGTCGCCAAGTTG  6180
Sbjct  6174  ..............A...................................T..T......  6233

Query  6181  AAAGGACCGAAAGCTGCGGCCTTGTTTGCAAAAACACATAACCTGATACCACTACACCAA  6240
Sbjct  6234  ............................................................  6293

Query  6241  GTTCCTATGGACAAATTCACGGTAGATATGAAGAGAGATGTCAAAGTCACGCCCGGAACC  6300
Sbjct  6294  ............................................................  6353

Query  6301  AAGCACACCGAAGAGAGACCAAAGGTACAGGTGATTCAAGCGGCAGAGCCACTAGCCACT  6360
Sbjct  6354  ............................................................  6413

Query  6361  GCCTACCTCTGCGGAATTCACCGTGAATTGGTGCGCCGTCTCAACAACGCGCTTTTCCCA  6420
Sbjct  6414  .....................................................C.....T  6473

Query  6421  AACATCCACACTTTGTTTGATATGTCCGCAGAGGATTTCGATGCAATCATAGCGGAACAT  6480
Sbjct  6474  ..T...................................T.....................  6533

Query  6481  TTTAAGCACGGTGACCATGTGTTGGAAACGGATATAGCCTCTTTTGACAAAAGTCAAGAT  6540
Sbjct  6534  .................C..........................................  6593

Query  6541  GATTCCATGGCACTCACTGCGTTAATGATCCTTGAGGACCTGGGAGTAGACCAAAACCTA  6600
Sbjct  6594  ............................................................  6653

Query  6601  ATGAATTTGATAGAGGCTGCATTCGGGGAAATCGTGAGTACACACTTGCCCACAGGTACT  6660
Sbjct  6654  ............................................................  6713

Query  6661  AGATTCAAATTTGGAGCTATGATGAAGTCTGGAATGTTTTTGACGCTGTTCGTCAATACA  6720
Sbjct  6714  .......G.....................C...............T..............  6773

Query  6721  ATTCTTAATGTGGTTATTGCGTGCCGAGTGTTGGAGGATCAATTGGCGCAGTCGCCGTGG  6780
Sbjct  6774  ........C..................................................C  6833

Query  6781  CCTGCTTTCATAGGAGATGACAACATAATCCATGGTATAATATCAGACAAATTGATGGCA  6840
Sbjct  6834  G..........................................................G  6893

Query  6841  GATAGATGTGCCACCTGGATGAACATGGAGGTCAAGATACTGGACTCTATAGTTGGAATA  6900
```

```
Sbjct  6894  ............................................T..C............  6953

Query  6901  CGGCCACCTTACTTCTGTGGAGGATTTATTGTATGTGACGATGTAACAGGTACAGCCTGC  6960
Sbjct  6954  ...........T................................................  7013

Query  6961  CGCGTCGCAGACCCACTGAAGAGATTGTTCAAGCTAGGTAAGCCATTGCCACTTGACGAT  7020
Sbjct  7014  ........................C...................................  7073

Query  7021  GGCCAAGATGAAGACAGAAGACGTGCATTACATGATGAAGTGAAAACCTGGTCGCGCGTA  7080
Sbjct  7074  .............................G..............................  7133

Query  7081  GGGCTGCGACACAGAGTGTGTGAAGCCATCGAAGACCGTTATGCCGTCCACTCATCAGAA  7140
Sbjct  7134  ..................................................T.........  7193

Query  7141  CTAGTTTTATTGGCACTGACTACTCTGTCTAAGAACTTGAAGTCCTTCAGAAACATAAGA  7200
Sbjct  7194  ............................................................  7253

Query  7201  GGGAAACCAATACATCTCTACGGTGGTCCTAAATAG  7236
Sbjct  7254  ....................................  7289
```

>Barmah Forest virus isolate SW94096, complete genome
Sequence ID: MN689042.1 Length: 11513
Range 1: 53 to 7288

Score:12661 bits(6856), Expect:0.0,
Identities:7109/7236(98%),  Gaps:0/7236(0%), Strand: Plus/Plus

```
Query  1     ATGGCGAAACCAGTTGTGAAGATCGACGTGGAACCTGAAAGCCATTTCGCTAAGCAGGTC  60
Sbjct  53    ...............................................T............  112

Query  61    CAGAGTTGCTTCCCGCAGTTTGAGATCGAAGCAGTGCAGACCACACCAAACGATCATGCA  120
Sbjct  113   .........................................G..................  172

Query  121   CACGCGAGGGCGTTTTCGCACCTTGCTACGAAGCTCATAGAAATGGAGACAGCAAAAGAT  180
Sbjct  173   ............................................................  232

Query  181   CAGATCATCCTCGATATCGGAAGTGCACCCGCGAGGAGACTGTATTCAGAACACAAGTAC  240
Sbjct  233   ............................................................  292

Query  241   CACTGTGTTTGCCCAATGAAGTGCACGGAAGATCCAGAGAGAATGCTAGGATATGCACGT  300
Sbjct  293   ............................................................  352

Query  301   AAGTTGATCGCAGGCTCTGCGAAAGGGAAGGCAGAAAAGTTACGCGATCTCAGGGATGTC  360
Sbjct  353   ............................................................  412

Query  361   TTGGCTACGCCAGACATCGAGACGCAGTCGCTATGTCTCCACACAGACGCATCCTGCAGA  420
Sbjct  413   ............................................................  472

Query  421   TACCGCGGTGATGTTGCCGTGTATCAAGACGTGTATGCCATTGACGCACCTACCACGCTG  480
Sbjct  473   ..................................................C.........  532
```

```
Query  481   TACCACCAAGCGTTAAAGGGCGTCAGGACCGCATATTGGATAGGCTTTGATACAACGCCG  540
Sbjct  533   ...........................................................A  592

Query  541   TTCATGTACGATGCACTAGCAGGAGCTTACCCGCTCTACTCCACAAACTGGGCTGATGAG  600
Sbjct  593   .........A...........................................C......  652

Query  601   CAAGTGCTCGAGTCCAGAAACATTGGGCTATGTTCAGACAAAGTTTCTGAAGGGGGAAAG  660
Sbjct  653   ............................................................  712

Query  661   AAAGGGAGATCAATCCTCAGGAAGAAGTTCTTGAAGCAGTCAGACAGAGTCATGTTCTCT  720
Sbjct  713   ............................................................  772

Query  721   GTCGGCTCGACGTTGTATACGGAAAGCCGTAAATTACTGCAAAGTTGGCACCTGCCATCC  780
Sbjct  773   ............................................C...............  832

Query  781   ACATTCCATCTCAAAGGCAAATCTTCGTTCACGTGCCGCTGCGACACTATCGTCAGCTGC  840
Sbjct  833   ................................A..............C............  892

Query  841   GAAGGGTATGTTCTGAAGAAAATTACAATGTGTCCTGGAGTGACAGGCAAACCGATAGGA  900
Sbjct  893   ................................C..C........................  952

Query  901   TATGCCGTCACCCATCACAAAGAAGGATTCGTAGTCGGAAAAGTCACAGATACCATTCGC  960
Sbjct  953   ....................G.............................C.........  1012

Query  961   GGCGAGAGAGTCTCCTTCGCCGTGTGTACTTATGTACCAACAACACTCTGCGACCAGATG  1020
Sbjct  1013  ............................................................  1072

Query  1021  ACCGGGATCCTAGCAACAGAAGTAACAGCCGATGATGCCCAGAAACTGCTGGTGGGTTTG  1080
Sbjct  1073  ........T...................................................  1132

Query  1081  AACCAGAGAATAGTAGTTAATGGTAGGACCCAGAGAAATACCAATACTATGAAGAACTAC  1140
Sbjct  1133  ............................................C...............  1192

Query  1141  CTGCTACCACTGGTTGCACAAGCGCTAGCAAAATGGGCGAAGGAAGCAAAACAGGATATG  1200
Sbjct  1193  ......................................A.....................  1252

Query  1201  GAAGATGAAAGACCCCTGAACGAACGCCAACGAACGCTAACGTGCCTCTGCTGCTGGGCA  1260
Sbjct  1253  ............................................................  1312

Query  1261  TTTAAGCGAAACAAACGCCACGCCATTTACAAGAGACCAGACACACAGAGTATAGTCAAG  1320
Sbjct  1313  ............................................................  1372

Query  1321  GTCCCTTGCGAATTCACAAGCTTTCCTTTGGTCAGCCTGTGGTCCGCTGGGATGTCTATA  1380
Sbjct  1373  ............................................................  1432

Query  1381  TCTCTTAGGCAGAAGTTGAAGATGATGCTGCAGGCGAGGCAGCCCACACAAATAGCAGCA  1440
Sbjct  1433  ............................................................  1492

Query  1441  GTGACTGAGGAACTCATACAAGAAGCAGCTGCAGTAGAGCAAGAGGCCGTGGATACGGCC  1500
Sbjct  1493  ..................................C.........................  1552

Query  1501  AATGCCGAGCTGGACCACGCCGCATGGCCCTCCATTGTGGATACGACAGAGCGCCATGTT  1560
Sbjct  1553  ...............................................G............  1612
```

```
Query  1561  GAGGTCGAAGTGGAAGAACTCGACCAGCGTGCAGGGGAAGGGGTAGTGGAAACACCTCGA  1620
Sbjct  1613  ............................................................  1672

Query  1621  AACTCTATCAAAGTTTCAACACAGATCGGGGACGCGTTAATCGGCAGTTACCTGATCCTA  1680
Sbjct  1673  ........T........T..........................................  1732

Query  1681  TCACCCCAAGCAGTCCTACGCAGCGAAAAATTAGCCTGCATACATGATCTTGCAGAGCAG  1740
Sbjct  1733  ............................................................  1792

Query  1741  GTTAAGTTGGTCACACACTCTGGCCGTAGTGGTAGGTACGCCGTCGACAAATACNACGGA  1800
Sbjct  1793  ..C.................C..T..............................G.....  1852

Query  1801  AGAGTACTAGTCCCTACAGGAGTGGCTATAGACATTCAATCGTTCCAGGCTCTCAGTGAG  1860
Sbjct  1853  ...................................C........................  1912

Query  1861  AGCGCGACCCTTGTGTACAACGAACGCGAGTTCGTTAACAGGAAGCTGTGGCACATAGCA  1920
Sbjct  1913  ..T..........................................T..............  1972

Query  1921  GTATACGGGGCAGCACTCAATACTGATGAAGAAGGATACGAGAAGGTCCCGGTAGAGAGA  1980
Sbjct  1973  ............................................................  2032

Query  1981  GCAGAATCAGATTATGTGTTTGATGTAGACCAAAAAATGTGCCTaaaaaaaGAGCAGGCA  2040
Sbjct  2033  .................A............A.............C...............  2092

Query  2041  TCAGGTTGGGTACTCTGTGGCGAACTAGTCAACCCCCCATTCCACGAATTCGCATATGAA  2100
Sbjct  2093  .....A......................................................  2152

Query  2101  GGGCTCCGCACGAGACCGTCAGCACCCTACAAGGTTCATACAGTAGGTGTGTACGGAGTG  2160
Sbjct  2153  ........T..T................................................  2212

Query  2161  CCAGGATCAGGCAAATCCGCAATAATCAAGAACACGGTCACCATGTCTGACCTAGTATTG  2220
Sbjct  2213  ............................................................  2272

Query  2221  AGTGGTAAGAAAGAGAACTGCTTAGAAATTATGAACGATGTACTTAAACACAGAGCTCTA  2280
Sbjct  2273  ............................................................  2332

Query  2281  CGTATCACAGCGAAGACCGTAGACTCAGTGTTATTAAACGGCGTGAAACACACGCCTAAC  2340
Sbjct  2333  .....T.............................G........................  2392

Query  2341  ATACTATACATCGACGAAGCGTTCTCATGCCATGCAGGGACTCTGTTGGCCACTATAGCC  2400
Sbjct  2393  ............................................................  2452

Query  2401  ATAGTCAGGCCCAAACAGAAAGTGGTACTGTGCGGAGACCCGAAACAATGCGGATTCTTC  2460
Sbjct  2453  T...........................................................  2512

Query  2461  AATATGATGCAACTGAAAGTTAATTACAATCATGACATCTGCTCAGAAGTCTTCCACAAA  2520
Sbjct  2513  .......................C....................................  2572

Query  2521  AGTATCTCTAGACGGTGCACCCAGGATATCACGGCCATCGTTTCCAAATTACATTACCAG  2580
Sbjct  2573  ............................................T...............  2632

Query  2581  GACCGAATGAGGACCACAAACCCCCGAAAAGGAGACATCATTATAGACACTACCGGCACT  2640
Sbjct  2633  ..................................................C.........  2692
```

```
Query  2641  ACCAAACCAGCCAAAACAGATCTGATTCTGACGTGCTTCAGGGGATGGGTGAAACAGTTG  2700
Sbjct  2693  ....................C.......................................  2752

Query  2701  CAGCAAGACTACAGAGGTAACGAAGTAATGACGGCTGCAGCGTCCCAAGGACTGACGAGG  2760
Sbjct  2753  ............................................................  2812

Query  2761  GCCTCCGTATATGCGGTTCGAACTAAAGTCAATGAGAACCCGCTATATGCACAGACCTCC  2820
Sbjct  2813  ............................................................  2872

Query  2821  GAGCACGTGAACGTGTTGTTAACACGCACAGAAAACAAGCTAGTATGGAAGACCTTGTCA  2880
Sbjct  2873  .......................................T....................  2932

Query  2881  ACAGATCCCTGGATTAAAACACTGACTAACCCACCTAGAGGGCACTATACCGCCACCATA  2940
Sbjct  2933  ............................................................  2992

Query  2941  GCAGAATGGGAAGCGGAACACCAGGGTATAATGAAGGCCATACAAGGGTATGCACCGCCC  3000
Sbjct  2993  ..............A.............................................  3052

Query  3001  GTGAACACCTTCATGAACAAAGTAAATGTGTGCTGGGCAAAGACACTTACGCCTGTGCTG  3060
Sbjct  3053  ...........T................................G..C..A.........  3112

Query  3061  GAAACTGCGGGTATCTCCCTGTCAGCAGAAGACTGGTCTGAACTGCTGCCCCCGTTTGCC  3120
Sbjct  3113  ............................................................  3172

Query  3121  CAGGACGTGGCGTACTCACCCGAGGTGGCATTAAACATCATATGCACGAAAATGTATGGG  3180
Sbjct  3173  ...............................................A............  3232

Query  3181  TTTGACTTAGACACTGGTCTTTTTCCAGGCCATCAGTGCCAATGACATACACCAAAGAC  3240
Sbjct  3233  ........G.....C.............A.........A.....................  3292

Query  3241  CATTGGGATAACAGAGTTGGAGGGAAAATGTATGGATTCAGCCAACAAGCATACGATCAG  3300
Sbjct  3293  ............................................................  3352

Query  3301  CTGGCAAGACGACATCCGTACCTTCGAGGTAGAGAGAAATCAGGAATGCAGATCGTAGTC  3360
Sbjct  3353  ...................................A........................  3412

Query  3361  ACTGAAATGCGTATCCAGCGCCCAAGATCGGATGCCAACATCATCCCGATCAACCGCAGG  3420
Sbjct  3413  .....G......................................................  3472

Query  3421  CTCCCTCACTCACTCGTAGCCACACACGAGTATAGGCGAGCTGCACGGGCCGAGGAATTC  3480
Sbjct  3473  ............................................G...............  3532

Query  3481  TTCACCACGACACGAGGGTACACTATGCTGCTGGTCTCTGAGTATAACATGAACTTACCA  3540
Sbjct  3533  ..........T.................................................  3592

Query  3541  AACAAGAAGATCACCTGGCTGGCTCCGATAGGGACGCAGGGGGCCCATCACACCGCCAAC  3600
Sbjct  3593  ............................................................  3652

Query  3601  CTAAACTTGGGGATACCACCTCTGCTGGGCAGTTTTGATGCGGTGGTTGTGAACATGCCG  3660
Sbjct  3653  ........................................T...................  3712

Query  3661  ACTCCATTCCGGAACCATCACTACCAGCAATGTGAAGACCACGCGATGAAACTCCAGATG  3720
Sbjct  3713  ................................C...........................  3772
```

```
Query  3721  CTGGCAGGCGACGCACTGAGGCACATTAAACCTGGCGGATCATTGTGGGTCAAGGCATAC  3780
Sbjct  3773  .............................G..............................  3832

Query  3781  GGCTACGCAGACCGGCACAGCGAGCACGTGGTCTTGGCATTGGCTAGAAAGTTTAAAAGC  3840
Sbjct  3833  .......................................C....................  3892

Query  3841  TTCAGAGTCACACAACCCTCATGCGTGACTTCCAACACCGAGGTGTTTCTCCACTTCTCA  3900
Sbjct  3893  ...........G........G.....A.................................  3952

Query  3901  ATTTTTGACAATGGCAAACGCGCGATAGCCCTGCATTCAGCTAATAGGAAGGCTAACAGT  3960
Sbjct  3953  ............................................................  4012

Query  3961  ATCTTCCAAAACACCTTCTTACCGGCGGGCAGTGCACCGGCGTACAGAGTCAAACGTGGA  4020
Sbjct  4013  ..........................A....A............................  4072

Query  4021  GACATTTCGAACGCCCCAGAGGATGCAGTGGTCAATGCAGCAAACCAACAGGGAGTGAAG  4080
Sbjct  4073  ............................................................  4132

Query  4081  GGTGCTGGAGTTTGCGGTGCAATTTACCGTAAGTGGCCGGACGCTTTCGGTGATGTCGCT  4140
Sbjct  4133  ............................................................  4192

Query  4141  ACTCCAACCGGAACAGCAGTTTCGAAATCCGTCCAAGATAAATTGGTGATCCACGCTGTC  4200
Sbjct  4193  ............................................................  4252

Query  4201  GGCCCGAATTTCTCAAAATGTTCAGAAGAGGAAGGGGACAGAGACCTAGCATCTGCTTAC  4260
Sbjct  4253  ...........T................................................  4312

Query  4261  AGAGCTGCAGCAGAAATAGTGATGGATaaaaaaaTTACAACAGTGGCCGTCCCCTTACTC  4320
Sbjct  4313  ..........................A...........................C.....  4372

Query  4321  TCCACCGGCATTTATGCCGGAGGAAAAAACAGAGTAGAACAGTCACTCAACCATCTCTTC  4380
Sbjct  4373  ..............C........G....................................  4432

Query  4381  ACGGCATTCGACAATACTGATGCAGATGTGACCATATATTGCATGGACAAAACATGGGAA  4440
Sbjct  4433  ........T...................................................  4492

Query  4441  AAGAAGATTAAGGAGGCAATCGATCACCGGACTTCGGTTGAGATGGTGCAGGATGACGTG  4500
Sbjct  4493  ............................................................  4552

Query  4501  CAGTTGGAGGAGGAACTGGTACGAGTACACCCTTTGAGTAGTTTAGCAGGTAGGAAGGGT  4560
Sbjct  4553  ......................................C.....................  4612

Query  4561  TACAGTACGGACAGCGGCCGAGTGTTTTCCTACCTGGAAGGTACCAAATTCCATCAGACT  4620
Sbjct  4613  ............................................................  4672

Query  4621  GCGGTGGACATAGCCGAAATGCAAGTGCTGTGGCCCGCCCTCAAAGAGTCTAATGAGCAA  4680
Sbjct  4673  ........T.............................T.....................  4732

Query  4681  ATAGTGGCATACACCTTAGGAGAATCAATGGACCAGATACGTGGCAAGTGCCCGACAGAA  4740
Sbjct  4733  ............................................................  4792

Query  4741  GATACTGACGCCTCCACACCTCCACGGACTGTGCCGTGCCTCTGTCGATACGCCATGACA  4800
Sbjct  4793  ............................................................  4852
```

```
Query  4801  CCAGAGAGAGTGTACCGACTTAAATGCACGAACACTACCCAATTTACGGTTTGCTCATCT  4860
Sbjct  4853  ............................................C.....C.........  4912

Query  4861  TTTGAGTTGCCAAAGTATCACATTCAGGGAGTGCAGAGAGTAAAATGTGAAAGAATCATC  4920
Sbjct  4913  ............................................................  4972

Query  4921  ATCTTAGATCCCACTGTTCCACCAACTTACAAACGGCCATGCATCAGACGGTACCCCTCC  4980
Sbjct  4973  ............................................................  5032

Query  4981  ACAATCTCTTGTAACTCCTCTGAGGACTCCAGGAGCTTGTCTACTTTTTCTGTCAGCTCC  5040
Sbjct  5033  ........C......................................C............  5092

Query  5041  GACTCCTCGATTGGTTCTCTGCCGGTCGGAGACACGAGACCCATTCCAGCCCCGAGGACC  5100
Sbjct  5093  .................CG..A...................T...........A......  5152

Query  5101  ATTTTCAGACCCGTCCCTGCCCCGAGAGCACCCGTGCTCAGAACCACACCGCCTCCTAAA  5160
Sbjct  5153  G...............................T..........T................  5212

Query  5161  CCACCGCGCACATTCACCGTGCGTGCAGAAGTGCACCAAGCACCCCCTACACCTGTACCT  5220
Sbjct  5213  ...................................T........................  5272

Query  5221  CCACCCAGACCGAAGAGGGCTGCAAAGTTGGCTCGTGAGATGCACCCCGGGTTCACCTTC  5280
Sbjct  5273  .....T......................................................  5332

Query  5281  GGGGACTTCGGAGAGCACGAGGTTGAGGAGCTTACGGCCTCTCCCTTAACCTTCGGAGAT  5340
Sbjct  5333  ..A..................A.................G....................  5392

Query  5341  TTTGCTGAAGGAGAGATCCAGGGGATGGGAGTGGAGTTTGAATGACTAGGCAGAGCCGGC  5400
Sbjct  5393  ....................A....C..................................  5452

Query  5401  GGGTACATTTTTTCGTCAGACACGGGTCCAGGCCACCTACAGCAGAGATCCGTTTTACAA  5460
Sbjct  5453  .........................................A.....G............  5512

Query  5461  AATTGCACGGCAGAATGTATCTACGAACCGGCAAAACTAGAAAAAATTCATGCACCAAAG  5520
Sbjct  5513  ............................................................  5572

Query  5521  TTGGATAAAACCAAGGAAGATATCTTAAGGAGCAAGTACCAAATGAAACCGTCTGAAGCA  5580
Sbjct  5573  ............................................................  5632

Query  5581  AACAAAAGCAGGTACCAATCTAGAAAAGTAGAAAATATGAAAGCAGAGATCGTAGGTAGA  5640
Sbjct  5633  ........T.........................................T.........  5692

Query  5641  CTCTTGGACGGACTGGGGGAGTATCTGGGCACCGAGCATCCAGTTGAATGCTACCGAATA  5700
Sbjct  5693  ..............A.............................................  5752

Query  5701  ACGTACCCGGTGCCTATATACTCAACTAGTGACCTCAGAGGTCTGTCTAGTGCCAAAACA  5760
Sbjct  5753  .....T....C...................AT..G.........................  5812

Query  5761  GCTGTTAGAGCTTGCAATGCATTTTTGGAAGCTAATTTTCCATCAGTCACTTCATATAAA  5820
Sbjct  5813  ............................................................  5872

Query  5821  ATTACTGATGAATACGACGCATACCTAGATATGGTAGATGGATCAGAGAGCTGTCTGGAC  5880
Sbjct  5873  ............................................................  5932
```

```
Query  5881  AGATCCTCCTTTTCGCCGTCTAGATTGCGTAGCTTTCCAAAAACACACTCATACTTGGAC  5940
Sbjct  5933  .................A...........C..............................  5992

Query  5941  CCACAGATCAACAGTGCGGTACCGTCACCATTCCAAAACACCTTACAAAATGTATTGGCA  6000
Sbjct  5993  ..G.........................................................  6052

Query  6001  GCGGCCACCAAAAGAAACTGTAATGTCACACAGATGAGAGAACTACCAACATATGATTCT  6060
Sbjct  6053  ...............................................G............  6112

Query  6061  GCAGTGCTAAATGTAGAGGCCTTCAGGAAATATGCGTGCAAGCCAGACGTATGGGATGAG  6120
Sbjct  6113  ............................................................  6172

Query  6121  TACAGGGATAATCCGATTTGCATAACCACCGAAAATGTCACCACTTACGTCGCCAAGTTG  6180
Sbjct  6173  ..............A...................................T..T......  6232

Query  6181  AAAGGACCGAAAGCTGCGGCCTTGTTTGCAAAAACACATAACCTGATACCACTACACCAA  6240
Sbjct  6233  ............................................................  6292

Query  6241  GTTCCTATGGACAAATTCACGGTAGATATGAAGAGAGATGTCAAAGTCACGCCCGGAACC  6300
Sbjct  6293  ............................................................  6352

Query  6301  AAGCACACCGAAGAGAGACCAAAGGTACAGGTGATTCAAGCGGCAGAGCCACTAGCCACT  6360
Sbjct  6353  ............................................................  6412

Query  6361  GCCTACCTCTGCGGAATTCACCGTGAATTGGTGCGCCGTCTCAACAACGCGCTTTTCCCA  6420
Sbjct  6413  .....................................................C.....T  6472

Query  6421  AACATCCACACTTTGTTTGATATGTCCGCAGAGGATTTCGATGCAATCATAGCGGAACAT  6480
Sbjct  6473  ..T...................................T.....................  6532

Query  6481  TTTAAGCACGGTGACCATGTGTTGGAAACGGATATAGCCTCTTTTGACAAAAGTCAAGAT  6540
Sbjct  6533  .................C..........................................  6592

Query  6541  GATTCCATGGCACTCACTGCGTTAATGATCCTTGAGGACCTGGGAGTAGACCAAAACCTA  6600
Sbjct  6593  ............................................................  6652

Query  6601  ATGAATTTGATAGAGGCTGCATTCGGGGAAATCGTGAGTACACACTTGCCCACAGGTACT  6660
Sbjct  6653  ............................................................  6712

Query  6661  AGATTCAAATTTGGAGCTATGATGAAGTCTGGAATGTTTTTGACGCTGTTCGTCAATACA  6720
Sbjct  6713  .......G.....................C...............T..............  6772

Query  6721  ATTCTTAATGTGGTTATTGCGTGCCGAGTGTTGGAGGATCAATTGGCGCAGTCGCCGTGG  6780
Sbjct  6773  ........C..................................................C  6832

Query  6781  CCTGCTTTCATAGGAGATGACAACATAATCCATGGTATAATATCAGACAAATTGATGGCA  6840
Sbjct  6833  G..........................................................G  6892

Query  6841  GATAGATGTGCCACCTGGATGAACATGGAGGTCAAGATACTGGACTCTATAGTTGGAATA  6900
Sbjct  6893  ............................................T..C............  6952

Query  6901  CGGCCACCTTACTTCTGTGGAGGATTTATTGTATGTGACGATGTAACAGGTACAGCCTGC  6960
Sbjct  6953  ...........T................................................  7012
```

```
Query  6961  CGCGTCGCAGACCCACTGAAGAGATTGTTCAAGCTAGGTAAGCCATTGCCACTTGACGAT  7020
Sbjct  7013  ........................C...................................  7072

Query  7021  GGCCAAGATGAAGACAGAAGACGTGCATTACATGATGAAGTGAAAACCTGGTCGCGCGTA  7080
Sbjct  7073  .............................G..............................  7132

Query  7081  GGGCTGCGACACAGAGTGTGTGAAGCCATCGAAGACCGTTATGCCGTCCACTCATCAGAA  7140
Sbjct  7133  ..................................................T.........  7192

Query  7141  CTAGTTTTATTGGCACTGACTACTCTGTCTAAGAACTTGAAGTCCTTCAGAAACATAAGA  7200
Sbjct  7193  ............................................................  7252

Query  7201  GGGAAACCAATACATCTCTACGGTGGTCCTAAATAG  7236
Sbjct  7253  ....................................  7288
```

>Barmah Forest virus isolate SW67821, complete genome
Sequence ID: MN689036.1 Length: 11494
Range 1: 51 to 7286

Score:12661 bits(6856), Expect:0.0,
Identities:7109/7236(98%),  Gaps:0/7236(0%), Strand: Plus/Plus

```
Query  1     ATGGCGAAACCAGTTGTGAAGATCGACGTGGAACCTGAAAGCCATTTCGCTAAGCAGGTC  60
Sbjct  51    ...............................................T............  110

Query  61    CAGAGTTGCTTCCCGCAGTTTGAGATCGAAGCAGTGCAGACCACACCAAACGATCATGCA  120
Sbjct  111   .........................................G..................  170

Query  121   CACGCGAGGGCGTTTTCGCACCTTGCTACGAAGCTCATAGAAATGGAGACAGCAAAAGAT  180
Sbjct  171   ............................................................  230

Query  181   CAGATCATCCTCGATATCGGAAGTGCACCCGCGAGGAGACTGTATTCAGAACACAAGTAC  240
Sbjct  231   ............................................................  290

Query  241   CACTGTGTTTGCCCAATGAAGTGCACGGAAGATCCAGAGAGAATGCTAGGATATGCACGT  300
Sbjct  291   ............................................................  350

Query  301   AAGTTGATCGCAGGCTCTGCGAAAGGGAAGGCAGAAAAGTTACGCGATCTCAGGGATGTC  360
Sbjct  351   ............................................................  410

Query  361   TTGGCTACGCCAGACATCGAGACGCAGTCGCTATGTCTCCACACAGACGCATCCTGCAGA  420
Sbjct  411   ............................................................  470

Query  421   TACCGCGGTGATGTTGCCGTGTATCAAGACGTGTATGCCATTGACGCACCTACCACGCTG  480
Sbjct  471   ..................................................C.........  530

Query  481   TACCACCAAGCGTTAAAGGGCGTCAGGACCGCATATTGGATAGGCTTTGATACAACGCCG  540
Sbjct  531   ...........................................................A  590

Query  541   TTCATGTACGATGCACTAGCAGGAGCTTACCCGCTCTACTCCACAAACTGGGCTGATGAG  600
Sbjct  591   .........A...........................................C......  650
```

```
Query  601   CAAGTGCTCGAGTCCAGAAACATTGGGCTATGTTCAGACAAAGTTTCTGAAGGGGGAAAG  660
Sbjct  651   ............................................................  710

Query  661   AAAGGGAGATCAATCCTCAGGAAGAAGTTCTTGAAGCAGTCAGACAGAGTCATGTTCTCT  720
Sbjct  711   ............................................................  770

Query  721   GTCGGCTCGACGTTGTATACGGAAAGCCGTAAATTACTGCAAAGTTGGCACCTGCCATCC  780
Sbjct  771   ............................................C...............  830

Query  781   ACATTCCATCTCAAAGGCAAATCTTCGTTCACGTGCCGCTGCGACACTATCGTCAGCTGC  840
Sbjct  831   ................................A..............C............  890

Query  841   GAAGGGTATGTTCTGAAGAAAATTACAATGTGTCCTGGAGTGACAGGCAAACCGATAGGA  900
Sbjct  891   ................................C..C........................  950

Query  901   TATGCCGTCACCCATCACAAAGAAGGATTCGTAGTCGGAAAAGTCACAGATACCATTCGC  960
Sbjct  951   ....................G.............................C.........  1010

Query  961   GGCGAGAGAGTCTCCTTCGCCGTGTGTACTTATGTACCAACAACACTCTGCGACCAGATG  1020
Sbjct  1011  ............................................................  1070

Query  1021  ACCGGGATCCTAGCAACAGAAGTAACAGCCGATGATGCCCAGAAACTGCTGGTGGGTTTG  1080
Sbjct  1071  ........T...................................................  1130

Query  1081  AACCAGAGAATAGTAGTTAATGGTAGGACCCAGAGAAATACCAATACTATGAAGAACTAC  1140
Sbjct  1131  ............................................C...............  1190

Query  1141  CTGCTACCACTGGTTGCACAAGCGCTAGCAAAATGGGCGAAGGAAGCAAAACAGGATATG  1200
Sbjct  1191  ......................................A.....................  1250

Query  1201  GAAGATGAAAGACCCCTGAACGAACGCCAACGAACGCTAACGTGCCTCTGCTGCTGGGCA  1260
Sbjct  1251  ............................................................  1310

Query  1261  TTTAAGCGAAACAAACGCCACGCCATTTACAAGAGACCAGACACACAGAGTATAGTCAAG  1320
Sbjct  1311  ............................................................  1370

Query  1321  GTCCCTTGCGAATTCACAAGCTTTCCTTTGGTCAGCCTGTGGTCCGCTGGGATGTCTATA  1380
Sbjct  1371  ............................................................  1430

Query  1381  TCTCTTAGGCAGAAGTTGAAGATGATGCTGCAGGCGAGGCAGCCCACACAAATAGCAGCA  1440
Sbjct  1431  ............................................................  1490

Query  1441  GTGACTGAGGAACTCATACAAGAAGCAGCTGCAGTAGAGCAAGAGGCCGTGGATACGGCC  1500
Sbjct  1491  ............T.....................C.........................  1550

Query  1501  AATGCCGAGCTGGACCACGCCGCATGGCCCTCCATTGTGGATACGACAGAGCGCCATGTT  1560
Sbjct  1551  ...............................................G............  1610

Query  1561  GAGGTCGAAGTGGAAGAACTCGACCAGCGTGCAGGGGAAGGGGTAGTGGAAACACCTCGA  1620
Sbjct  1611  ............................................................  1670

Query  1621  AACTCTATCAAAGTTTCAACACAGATCGGGGACGCGTTAATCGGCAGTTACCTGATCCTA  1680
Sbjct  1671  ........T...................................................  1730
```

```
Query  1681  TCACCCCAAGCAGTCCTACGCAGCGAAAAATTAGCCTGCATACATGATCTTGCAGAGCAG  1740
Sbjct  1731  ............................................................  1790

Query  1741  GTTAAGTTGGTCACACACTCTGGCCGTAGTGGTAGGTACGCCGTCGACAAATACNACGGA  1800
Sbjct  1791  ..C.................C..T..............................G.....  1850

Query  1801  AGAGTACTAGTCCCTACAGGAGTGGCTATAGACATTCAATCGTTCCAGGCTCTCAGTGAG  1860
Sbjct  1851  ...................................C........................  1910

Query  1861  AGCGCGACCCTTGTGTACAACGAACGCGAGTTCGTTAACAGGAAGCTGTGGCACATAGCA  1920
Sbjct  1911  ..T..........................................T..............  1970

Query  1921  GTATACGGGGCAGCACTCAATACTGATGAAGAAGGATACGAGAAGGTCCCGGTAGAGAGA  1980
Sbjct  1971  ............................................................  2030

Query  1981  GCAGAATCAGATTATGTGTTTGATGTAGACCAAAAAATGTGCCTaaaaaaaGAGCAGGCA  2040
Sbjct  2031  .................A............A.............C...............  2090

Query  2041  TCAGGTTGGGTACTCTGTGGCGAACTAGTCAACCCCCCATTCCACGAATTCGCATATGAA  2100
Sbjct  2091  .....A......................................................  2150

Query  2101  GGGCTCCGCACGAGACCGTCAGCACCCTACAAGGTTCATACAGTAGGTGTGTACGGAGTG  2160
Sbjct  2151  ........T..T................................................  2210

Query  2161  CCAGGATCAGGCAAATCCGCAATAATCAAGAACACGGTCACCATGTCTGACCTAGTATTG  2220
Sbjct  2211  ............................................................  2270

Query  2221  AGTGGTAAGAAAGAGAACTGCTTAGAAATTATGAACGATGTACTTAAACACAGAGCTCTA  2280
Sbjct  2271  ............................................................  2330

Query  2281  CGTATCACAGCGAAGACCGTAGACTCAGTGTTATTAAACGGCGTGAAACACACGCCTAAC  2340
Sbjct  2331  .....T.............................G........................  2390

Query  2341  ATACTATACATCGACGAAGCGTTCTCATGCCATGCAGGGACTCTGTTGGCCACTATAGCC  2400
Sbjct  2391  ............................................................  2450

Query  2401  ATAGTCAGGCCCAAACAGAAAGTGGTACTGTGCGGAGACCCGAAACAATGCGGATTCTTC  2460
Sbjct  2451  T..........................................................T  2510

Query  2461  AATATGATGCAACTGAAAGTTAATTACAATCATGACATCTGCTCAGAAGTCTTCCACAAA  2520
Sbjct  2511  .......................C....................................  2570

Query  2521  AGTATCTCTAGACGGTGCACCCAGGATATCACGGCCATCGTTTCCAAATTACATTACCAG  2580
Sbjct  2571  ............................................T...............  2630

Query  2581  GACCGAATGAGGACCACAAACCCCCGAAAAGGAGACATCATTATAGACACTACCGGCACT  2640
Sbjct  2631  ..................................................C.........  2690

Query  2641  ACCAAACCAGCCAAAACAGATCTGATTCTGACGTGCTTCAGGGGATGGGTGAAACAGTTG  2700
Sbjct  2691  ....................C.......................................  2750

Query  2701  CAGCAAGACTACAGAGGTAACGAAGTAATGACGGCTGCAGCGTCCCAAGGACTGACGAGG  2760
Sbjct  2751  ............................................................  2810
```

```
Query  2761  GCCTCCGTATATGCGGTTCGAACTAAAGTCAATGAGAACCCGCTATATGCACAGACCTCC  2820
Sbjct  2811  ............................................................  2870

Query  2821  GAGCACGTGAACGTGTTGTTAACACGCACAGAAAACAAGCTAGTATGGAAGACCTTGTCA  2880
Sbjct  2871  ..................C....................T....................  2930

Query  2881  ACAGATCCCTGGATTAAAACACTGACTAACCCACCTAGAGGGCACTATACCGCCACCATA  2940
Sbjct  2931  ............................................................  2990

Query  2941  GCAGAATGGGAAGCGGAACACCAGGGTATAATGAAGGCCATACAAGGGTATGCACCGCCC  3000
Sbjct  2991  ..............A.......................T.....................  3050

Query  3001  GTGAACACCTTCATGAACAAAGTAAATGTGTGCTGGGCAAAGACACTTACGCCTGTGCTG  3060
Sbjct  3051  ...............................................C..A.........  3110

Query  3061  GAAACTGCGGGTATCTCCCTGTCAGCAGAAGACTGGTCTGAACTGCTGCCCCCGTTTGCC  3120
Sbjct  3111  ............................................................  3170

Query  3121  CAGGACGTGGCGTACTCACCCGAGGTGGCATTAAACATCATATGCACGAAAATGTATGGG  3180
Sbjct  3171  ...............................................A............  3230

Query  3181  TTTGACTTAGACACTGGTCTTTTTCCAGGCCATCAGTGCCAATGACATACACCAAAGAC  3240
Sbjct  3231  ........G.....C.............A.........A.....................  3290

Query  3241  CATTGGGATAACAGAGTTGGAGGGAAAATGTATGGATTCAGCCAACAAGCATACGATCAG  3300
Sbjct  3291  ............................................................  3350

Query  3301  CTGGCAAGACGACATCCGTACCTTCGAGGTAGAGAGAAATCAGGAATGCAGATCGTAGTC  3360
Sbjct  3351  ...................................A........................  3410

Query  3361  ACTGAAATGCGTATCCAGCGCCCAAGATCGGATGCCAACATCATCCCGATCAACCGCAGG  3420
Sbjct  3411  .....G.................C....................................  3470

Query  3421  CTCCCTCACTCACTCGTAGCCACACACGAGTATAGGCGAGCTGCACGGGCCGAGGAATTC  3480
Sbjct  3471  ............................................G...............  3530

Query  3481  TTCACCACGACACGAGGGTACACTATGCTGCTGGTCTCTGAGTATAACATGAACTTACCA  3540
Sbjct  3531  ..........T.................................................  3590

Query  3541  AACAAGAAGATCACCTGGCTGGCTCCGATAGGGACGCAGGGGGCCCATCACACCGCCAAC  3600
Sbjct  3591  ............................................................  3650

Query  3601  CTAAACTTGGGGATACCACCTCTGCTGGGCAGTTTTGATGCGGTGGTTGTGAACATGCCG  3660
Sbjct  3651  ............................................................  3710

Query  3661  ACTCCATTCCGGAACCATCACTACCAGCAATGTGAAGACCACGCGATGAAACTCCAGATG  3720
Sbjct  3711  ................................C...........................  3770

Query  3721  CTGGCAGGCGACGCACTGAGGCACATTAAACCTGGCGGATCATTGTGGGTCAAGGCATAC  3780
Sbjct  3771  ............................................................  3830

Query  3781  GGCTACGCAGACCGGCACAGCGAGCACGTGGTCTTGGCATTGGCTAGAAAGTTTAAAAGC  3840
Sbjct  3831  .......................................C....................  3890
```

```
Query  3841  TTCAGAGTCACACAACCCTCATGCGTGACTTCCAACACCGAGGTGTTTCTCCACTTCTCA  3900
Sbjct  3891  ...........G.....A..G.....A.................................  3950

Query  3901  ATTTTTGACAATGGCAAACGCGCGATAGCCCTGCATTCAGCTAATAGGAAGGCTAACAGT  3960
Sbjct  3951  ............................................................  4010

Query  3961  ATCTTCCAAAACACCTTCTTACCGGCGGGCAGTGCACCGGCGTACAGAGTCAAACGTGGA  4020
Sbjct  4011  ..........................A....A............................  4070

Query  4021  GACATTTCGAACGCCCCAGAGGATGCAGTGGTCAATGCAGCAAACCAACAGGGAGTGAAG  4080
Sbjct  4071  ............................................................  4130

Query  4081  GGTGCTGGAGTTTGCGGTGCAATTTACCGTAAGTGGCCGGACGCTTTCGGTGATGTCGCT  4140
Sbjct  4131  ............................................................  4190

Query  4141  ACTCCAACCGGAACAGCAGTTTCGAAATCCGTCCAAGATAAATTGGTGATCCACGCTGTC  4200
Sbjct  4191  ............................................................  4250

Query  4201  GGCCCGAATTTCTCAAAATGTTCAGAAGAGGAAGGGGACAGAGACCTAGCATCTGCTTAC  4260
Sbjct  4251  ............................................................  4310

Query  4261  AGAGCTGCAGCAGAAATAGTGATGGATaaaaaaaTTACAACAGTGGCCGTCCCCTTACTC  4320
Sbjct  4311  ..........................A.................................  4370

Query  4321  TCCACCGGCATTTATGCCGGAGGAAAAAACAGAGTAGAACAGTCACTCAACCATCTCTTC  4380
Sbjct  4371  ..............C........G....................................  4430

Query  4381  ACGGCATTCGACAATACTGATGCAGATGTGACCATATATTGCATGGACAAAACATGGGAA  4440
Sbjct  4431  ........T...................................................  4490

Query  4441  AAGAAGATTAAGGAGGCAATCGATCACCGGACTTCGGTTGAGATGGTGCAGGATGACGTG  4500
Sbjct  4491  ............................................................  4550

Query  4501  CAGTTGGAGGAGGAACTGGTACGAGTACACCCTTTGAGTAGTTTAGCAGGTAGGAAGGGT  4560
Sbjct  4551  ............................................................  4610

Query  4561  TACAGTACGGACAGCGGCCGAGTGTTTTCCTACCTGGAAGGTACCAAATTCCATCAGACT  4620
Sbjct  4611  ............................................................  4670

Query  4621  GCGGTGGACATAGCCGAAATGCAAGTGCTGTGGCCCGCCCTCAAAGAGTCTAATGAGCAA  4680
Sbjct  4671  ........T.............................T.....................  4730

Query  4681  ATAGTGGCATACACCTTAGGAGAATCAATGGACCAGATACGTGGCAAGTGCCCGACAGAA  4740
Sbjct  4731  ............................................................  4790

Query  4741  GATACTGACGCCTCCACACCTCCACGGACTGTGCCGTGCCTCTGTCGATACGCCATGACA  4800
Sbjct  4791  ..C.........................................................  4850

Query  4801  CCAGAGAGAGTGTACCGACTTAAATGCACGAACACTACCCAATTTACGGTTTGCTCATCT  4860
Sbjct  4851  ............................................C.....C.........  4910

Query  4861  TTTGAGTTGCCAAAGTATCACATTCAGGGAGTGCAGAGAGTAAAATGTGAAAGAATCATC  4920
Sbjct  4911  ............................................................  4970
```

```
Query  4921  ATCTTAGATCCCACTGTTCCACCAACTTACAAACGGCCATGCATCAGACGGTACCCCTCC  4980
Sbjct  4971  ............................................................  5030

Query  4981  ACAATCTCTTGTAACTCCTCTGAGGACTCCAGGAGCTTGTCTACTTTTTCTGTCAGCTCC  5040
Sbjct  5031  ........C......................................C............  5090

Query  5041  GACTCCTCGATTGGTTCTCTGCCGGTCGGAGACACGAGACCCATTCCAGCCCCGAGGACC  5100
Sbjct  5091  .................CG..A...................T...........A.....T  5150

Query  5101  ATTTTCAGACCCGTCCCTGCCCCGAGAGCACCCGTGCTCAGAACCACACCGCCTCCTAAA  5160
Sbjct  5151  G..........................................T................  5210

Query  5161  CCACCGCGCACATTCACCGTGCGTGCAGAAGTGCACCAAGCACCCCCTACACCTGTACCT  5220
Sbjct  5211  ...................................T........................  5270

Query  5221  CCACCCAGACCGAAGAGGGCTGCAAAGTTGGCTCGTGAGATGCACCCCGGGTTCACCTTC  5280
Sbjct  5271  ............................................................  5330

Query  5281  GGGGACTTCGGAGAGCACGAGGTTGAGGAGCTTACGGCCTCTCCCTTAACCTTCGGAGAT  5340
Sbjct  5331  ..A..................A.................G....................  5390

Query  5341  TTTGCTGAAGGAGAGATCCAGGGGATGGGAGTGGAGTTTGAATGACTAGGCAGAGCCGGC  5400
Sbjct  5391  ............................................................  5450

Query  5401  GGGTACATTTTTTCGTCAGACACGGGTCCAGGCCACCTACAGCAGAGATCCGTTTTACAA  5460
Sbjct  5451  .........................................A..................  5510

Query  5461  AATTGCACGGCAGAATGTATCTACGAACCGGCAAAACTAGAAAAAATTCATGCACCAAAG  5520
Sbjct  5511  ............................................................  5570

Query  5521  TTGGATAAAACCAAGGAAGATATCTTAAGGAGCAAGTACCAAATGAAACCGTCTGAAGCA  5580
Sbjct  5571  ........G...................................................  5630

Query  5581  AACAAAAGCAGGTACCAATCTAGAAAAGTAGAAAATATGAAAGCAGAGATCGTAGGTAGA  5640
Sbjct  5631  ........T.........................................T.........  5690

Query  5641  CTCTTGGACGGACTGGGGGAGTATCTGGGCACCGAGCATCCAGTTGAATGCTACCGAATA  5700
Sbjct  5691  ..............A.......................C.....................  5750

Query  5701  ACGTACCCGGTGCCTATATACTCAACTAGTGACCTCAGAGGTCTGTCTAGTGCCAAAACA  5760
Sbjct  5751  .....T........................AT..G.........................  5810

Query  5761  GCTGTTAGAGCTTGCAATGCATTTTTGGAAGCTAATTTTCCATCAGTCACTTCATATAAA  5820
Sbjct  5811  ............................................................  5870

Query  5821  ATTACTGATGAATACGACGCATACCTAGATATGGTAGATGGATCAGAGAGCTGTCTGGAC  5880
Sbjct  5871  ............................................................  5930

Query  5881  AGATCCTCCTTTTCGCCGTCTAGATTGCGTAGCTTTCCAAAAACACACTCATACTTGGAC  5940
Sbjct  5931  .................A...........C..............................  5990

Query  5941  CCACAGATCAACAGTGCGGTACCGTCACCATTCCAAAACACCTTACAAAATGTATTGGCA  6000
Sbjct  5991  ..G.......................G.................................  6050
```

```
Query  6001  GCGGCCACCAAAAGAAACTGTAATGTCACACAGATGAGAGAACTACCAACATATGATTCT  6060
Sbjct  6051  ...............................................G............  6110

Query  6061  GCAGTGCTAAATGTAGAGGCCTTCAGGAAATATGCGTGCAAGCCAGACGTATGGGATGAG  6120
Sbjct  6111  ............................................................  6170

Query  6121  TACAGGGATAATCCGATTTGCATAACCACCGAAAATGTCACCACTTACGTCGCCAAGTTG  6180
Sbjct  6171  ..............A......................................T......  6230

Query  6181  AAAGGACCGAAAGCTGCGGCCTTGTTTGCAAAAACACATAACCTGATACCACTACACCAA  6240
Sbjct  6231  ............................................................  6290

Query  6241  GTTCCTATGGACAAATTCACGGTAGATATGAAGAGAGATGTCAAAGTCACGCCCGGAACC  6300
Sbjct  6291  ............................................................  6350

Query  6301  AAGCACACCGAAGAGAGACCAAAGGTACAGGTGATTCAAGCGGCAGAGCCACTAGCCACT  6360
Sbjct  6351  ...................................................T........  6410

Query  6361  GCCTACCTCTGCGGAATTCACCGTGAATTGGTGCGCCGTCTCAACAACGCGCTTTTCCCA  6420
Sbjct  6411  .....................................................C......  6470

Query  6421  AACATCCACACTTTGTTTGATATGTCCGCAGAGGATTTCGATGCAATCATAGCGGAACAT  6480
Sbjct  6471  ..T................................C..T.....................  6530

Query  6481  TTTAAGCACGGTGACCATGTGTTGGAAACGGATATAGCCTCTTTTGACAAAAGTCAAGAT  6540
Sbjct  6531  .................C..........................................  6590

Query  6541  GATTCCATGGCACTCACTGCGTTAATGATCCTTGAGGACCTGGGAGTAGACCAAAACCTA  6600
Sbjct  6591  ............................................................  6650

Query  6601  ATGAATTTGATAGAGGCTGCATTCGGGGAAATCGTGAGTACACACTTGCCCACAGGTACT  6660
Sbjct  6651  ............................................................  6710

Query  6661  AGATTCAAATTTGGAGCTATGATGAAGTCTGGAATGTTTTTGACGCTGTTCGTCAATACA  6720
Sbjct  6711  .......G.....................C...............T..............  6770

Query  6721  ATTCTTAATGTGGTTATTGCGTGCCGAGTGTTGGAGGATCAATTGGCGCAGTCGCCGTGG  6780
Sbjct  6771  ........C......................................A...........C  6830

Query  6781  CCTGCTTTCATAGGAGATGACAACATAATCCATGGTATAATATCAGACAAATTGATGGCA  6840
Sbjct  6831  G..........................................................G  6890

Query  6841  GATAGATGTGCCACCTGGATGAACATGGAGGTCAAGATACTGGACTCTATAGTTGGAATA  6900
Sbjct  6891  ..............T.............................T..C............  6950

Query  6901  CGGCCACCTTACTTCTGTGGAGGATTTATTGTATGTGACGATGTAACAGGTACAGCCTGC  6960
Sbjct  6951  ...........T................................................  7010

Query  6961  CGCGTCGCAGACCCACTGAAGAGATTGTTCAAGCTAGGTAAGCCATTGCCACTTGACGAT  7020
Sbjct  7011  ..............G.........C...................................  7070

Query  7021  GGCCAAGATGAAGACAGAAGACGTGCATTACATGATGAAGTGAAAACCTGGTCGCGCGTA  7080
Sbjct  7071  ........C...................................................  7130
```

```
Query  7081  GGGCTGCGACACAGAGTGTGTGAAGCCATCGAAGACCGTTATGCCGTCCACTCATCAGAA  7140
Sbjct  7131  ..................................................T.........  7190

Query  7141  CTAGTTTTATTGGCACTGACTACTCTGTCTAAGAACTTGAAGTCCTTCAGAAACATAAGA  7200
Sbjct  7191  ............................................................  7250

Query  7201  GGGAAACCAATACATCTCTACGGTGGTCCTAAATAG  7236
Sbjct  7251  ....................................  7286
```

>Barmah Forest virus isolate DC56192, complete genome
Sequence ID: MN689023.1 Length: 11499
Range 1: 56 to 7291

Score:12661 bits(6856), Expect:0.0,
Identities:7109/7236(98%), Gaps:0/7236(0%), Strand: Plus/Plus

```
Query  1      ATGGCGAAACCAGTTGTGAAGATCGACGTGGAACCTGAAAGCCATTTCGCTAAGCAGGTC  60
Sbjct  56     ...............................................T............  115

Query  61     CAGAGTTGCTTCCCGCAGTTTGAGATCGAAGCAGTGCAGACCACACCAAACGATCATGCA  120
Sbjct  116    .........................................G..................  175

Query  121    CACGCGAGGGCGTTTTCGCACCTTGCTACGAAGCTCATAGAAATGGAGACAGCAAAAGAT  180
Sbjct  176    ............................................................  235

Query  181    CAGATCATCCTCGATATCGGAAGTGCACCCGCGAGGAGACTGTATTCAGAACACAAGTAC  240
Sbjct  236    ............................................................  295

Query  241    CACTGTGTTTGCCCAATGAAGTGCACGGAAGATCCAGAGAGAATGCTAGGATATGCACGT  300
Sbjct  296    ............................................................  355

Query  301    AAGTTGATCGCAGGCTCTGCGAAAGGGAAGGCAGAAAAGTTACGCGATCTCAGGGATGTC  360
Sbjct  356    ............................................................  415

Query  361    TTGGCTACGCCAGACATCGAGACGCAGTCGCTATGTCTCCACACAGACGCATCCTGCAGA  420
Sbjct  416    ............................................................  475

Query  421    TACCGCGGTGATGTTGCCGTGTATCAAGACGTGTATGCCATTGACGCACCTACCACGCTG  480
Sbjct  476    ..................................................C.........  535

Query  481    TACCACCAAGCGTTAAAGGGCGTCAGGACCGCATATTGGATAGGCTTTGATACAACGCCG  540
Sbjct  536    ...........................................................A  595

Query  541    TTCATGTACGATGCACTAGCAGGAGCTTACCCGCTCTACTCCACAAACTGGGCTGATGAG  600
Sbjct  596    .........A...........................................C......  655

Query  601    CAAGTGCTCGAGTCCAGAAACATTGGGCTATGTTCAGACAAAGTTTCTGAAGGGGGAAAG  660
Sbjct  656    ............................................................  715

Query  661    AAAGGGAGATCAATCCTCAGGAAGAAGTTCTTGAAGCAGTCAGACAGAGTCATGTTCTCT  720
```

```
Sbjct  716   ............................................................  775

Query  721   GTCGGCTCGACGTTGTATACGGAAAGCCGTAAATTACTGCAAAGTTGGCACCTGCCATCC  780
Sbjct  776   ............................................C...............  835

Query  781   ACATTCCATCTCAAAGGCAAATCTTCGTTCACGTGCCGCTGCGACACTATCGTCAGCTGC  840
Sbjct  836   ................................A..............C............  895

Query  841   GAAGGGTATGTTCTGAAGAAAATTACAATGTGTCCTGGAGTGACAGGCAAACCGATAGGA  900
Sbjct  896   ................................C..C........................  955

Query  901   TATGCCGTCACCCATCACAAAGAAGGATTCGTAGTCGGAAAAGTCACAGATACCATTCGC  960
Sbjct  956   ....................G.............................C.........  1015

Query  961   GGCGAGAGAGTCTCCTTCGCCGTGTGTACTTATGTACCAACAACACTCTGCGACCAGATG  1020
Sbjct  1016  ............................................................  1075

Query  1021  ACCGGGATCCTAGCAACAGAAGTAACAGCCGATGATGCCCAGAAACTGCTGGTGGGTTTG  1080
Sbjct  1076  ........T...................................................  1135

Query  1081  AACCAGAGAATAGTAGTTAATGGTAGGACCCAGAGAAATACCAATACTATGAAGAACTAC  1140
Sbjct  1136  ............................................C...............  1195

Query  1141  CTGCTACCACTGGTTGCACAAGCGCTAGCAAAATGGGCGAAGGAAGCAAAACAGGATATG  1200
Sbjct  1196  ......................................A.....................  1255

Query  1201  GAAGATGAAAGACCCCTGAACGAACGCCAACGAACGCTAACGTGCCTCTGCTGCTGGGCA  1260
Sbjct  1256  ............................................................  1315

Query  1261  TTTAAGCGAAACAAACGCCACGCCATTTACAAGAGACCAGACACACAGAGTATAGTCAAG  1320
Sbjct  1316  ............................................................  1375

Query  1321  GTCCCTTGCGAATTCACAAGCTTTCCTTTGGTCAGCCTGTGGTCCGCTGGGATGTCTATA  1380
Sbjct  1376  ............................................................  1435

Query  1381  TCTCTTAGGCAGAAGTTGAAGATGATGCTGCAGGCGAGGCAGCCCACACAAATAGCAGCA  1440
Sbjct  1436  ............................................................  1495

Query  1441  GTGACTGAGGAACTCATACAAGAAGCAGCTGCAGTAGAGCAAGAGGCCGTGGATACGGCC  1500
Sbjct  1496  ..................................C.........................  1555

Query  1501  AATGCCGAGCTGGACCACGCCGCATGGCCCTCCATTGTGGATACGACAGAGCGCCATGTT  1560
Sbjct  1556  ...............................................G............  1615

Query  1561  GAGGTCGAAGTGGAAGAACTCGACCAGCGTGCAGGGGAAGGGGTAGTGGAAACACCTCGA  1620
Sbjct  1616  ............................................................  1675

Query  1621  AACTCTATCAAAGTTTCAACACAGATCGGGGACGCGTTAATCGGCAGTTACCTGATCCTA  1680
Sbjct  1676  ........T........T..........................................  1735

Query  1681  TCACCCCAAGCAGTCCTACGCAGCGAAAAATTAGCCTGCATACATGATCTTGCAGAGCAG  1740
Sbjct  1736  ............................................................  1795

Query  1741  GTTAAGTTGGTCACACACTCTGGCCGTAGTGGTAGGTACGCCGTCGACAAATACNACGGA  1800
```

```
Sbjct  1796  ..C.................C..T..............................G.....  1855

Query  1801  AGAGTACTAGTCCCTACAGGAGTGGCTATAGACATTCAATCGTTCCAGGCTCTCAGTGAG  1860
Sbjct  1856  ...................................C........................  1915

Query  1861  AGCGCGACCCTTGTGTACAACGAACGCGAGTTCGTTAACAGGAAGCTGTGGCACATAGCA  1920
Sbjct  1916  ..T..........................................T..............  1975

Query  1921  GTATACGGGGCAGCACTCAATACTGATGAAGAAGGATACGAGAAGGTCCCGGTAGAGAGA  1980
Sbjct  1976  ............................................................  2035

Query  1981  GCAGAATCAGATTATGTGTTTGATGTAGACCAAAAAATGTGCCTaaaaaaaGAGCAGGCA  2040
Sbjct  2036  .................A............A.............C...............  2095

Query  2041  TCAGGTTGGGTACTCTGTGGCGAACTAGTCAACCCCCCATTCCACGAATTCGCATATGAA  2100
Sbjct  2096  .....A......................................................  2155

Query  2101  GGGCTCCGCACGAGACCGTCAGCACCCTACAAGGTTCATACAGTAGGTGTGTACGGAGTG  2160
Sbjct  2156  ........T..T................................................  2215

Query  2161  CCAGGATCAGGCAAATCCGCAATAATCAAGAACACGGTCACCATGTCTGACCTAGTATTG  2220
Sbjct  2216  ............................................................  2275

Query  2221  AGTGGTAAGAAAGAGAACTGCTTAGAAATTATGAACGATGTACTTAAACACAGAGCTCTA  2280
Sbjct  2276  ............................................................  2335

Query  2281  CGTATCACAGCGAAGACCGTAGACTCAGTGTTATTAAACGGCGTGAAACACACGCCTAAC  2340
Sbjct  2336  .....T.............................G........................  2395

Query  2341  ATACTATACATCGACGAAGCGTTCTCATGCCATGCAGGGACTCTGTTGGCCACTATAGCC  2400
Sbjct  2396  ............................................................  2455

Query  2401  ATAGTCAGGCCCAAACAGAAAGTGGTACTGTGCGGAGACCCGAAACAATGCGGATTCTTC  2460
Sbjct  2456  T...........................................................  2515

Query  2461  AATATGATGCAACTGAAAGTTAATTACAATCATGACATCTGCTCAGAAGTCTTCCACAAA  2520
Sbjct  2516  .......................C....................................  2575

Query  2521  AGTATCTCTAGACGGTGCACCCAGGATATCACGGCCATCGTTTCCAAATTACATTACCAG  2580
Sbjct  2576  ............................................T...............  2635

Query  2581  GACCGAATGAGGACCACAAACCCCCGAAAAGGAGACATCATTATAGACACTACCGGCACT  2640
Sbjct  2636  ..................................................C.........  2695

Query  2641  ACCAAACCAGCCAAAACAGATCTGATTCTGACGTGCTTCAGGGGATGGGTGAAACAGTTG  2700
Sbjct  2696  ....................C.......................................  2755

Query  2701  CAGCAAGACTACAGAGGTAACGAAGTAATGACGGCTGCAGCGTCCCAAGGACTGACGAGG  2760
Sbjct  2756  ............................................................  2815

Query  2761  GCCTCCGTATATGCGGTTCGAACTAAAGTCAATGAGAACCCGCTATATGCACAGACCTCC  2820
Sbjct  2816  ............................................................  2875

Query  2821  GAGCACGTGAACGTGTTGTTAACACGCACAGAAAACAAGCTAGTATGGAAGACCTTGTCA  2880
```

```
Sbjct  2876  .......................................T....................  2935

Query  2881  ACAGATCCCTGGATTAAAACACTGACTAACCCACCTAGAGGGCACTATACCGCCACCATA  2940
Sbjct  2936  ............................................................  2995

Query  2941  GCAGAATGGGAAGCGGAACACCAGGGTATAATGAAGGCCATACAAGGGTATGCACCGCCC  3000
Sbjct  2996  ..............A.............................................  3055

Query  3001  GTGAACACCTTCATGAACAAAGTAAATGTGTGCTGGGCAAAGACACTTACGCCTGTGCTG  3060
Sbjct  3056  ...........T................................G..C..A.........  3115

Query  3061  GAAACTGCGGGTATCTCCCTGTCAGCAGAAGACTGGTCTGAACTGCTGCCCCCGTTTGCC  3120
Sbjct  3116  ............................................................  3175

Query  3121  CAGGACGTGGCGTACTCACCCGAGGTGGCATTAAACATCATATGCACGAAAATGTATGGG  3180
Sbjct  3176  ...............................................A............  3235

Query  3181  TTTGACTTAGACACTGGTCTTTTTTCCAGGCCATCAGTGCCAATGACATACACCAAAGAC  3240
Sbjct  3236  ........G.....C.............A.........A.....................  3295

Query  3241  CATTGGGATAACAGAGTTGGAGGGAAAATGTATGGATTCAGCCAACAAGCATACGATCAG  3300
Sbjct  3296  ............................................................  3355

Query  3301  CTGGCAAGACGACATCCGTACCTTCGAGGTAGAGAGAAATCAGGAATGCAGATCGTAGTC  3360
Sbjct  3356  ...................................A........................  3415

Query  3361  ACTGAAATGCGTATCCAGCGCCCAAGATCGGATGCCAACATCATCCCGATCAACCGCAGG  3420
Sbjct  3416  .....G......................................................  3475

Query  3421  CTCCCTCACTCACTCGTAGCCACACACGAGTATAGGCGAGCTGCACGGGCCGAGGAATTC  3480
Sbjct  3476  ............................................G...............  3535

Query  3481  TTCACCACGACACGAGGGTACACTATGCTGCTGGTCTCTGAGTATAACATGAACTTACCA  3540
Sbjct  3536  ..........T.................................................  3595

Query  3541  AACAAGAAGATCACCTGGCTGGCTCCGATAGGGACGCAGGGGGCCCATCACACCGCCAAC  3600
Sbjct  3596  ............................................................  3655

Query  3601  CTAAACTTGGGGATACCACCTCTGCTGGGCAGTTTTGATGCGGTGGTTGTGAACATGCCG  3660
Sbjct  3656  ........................................T...................  3715

Query  3661  ACTCCATTCCGGAACCATCACTACCAGCAATGTGAAGACCACGCGATGAAACTCCAGATG  3720
Sbjct  3716  ................................C...........................  3775

Query  3721  CTGGCAGGCGACGCACTGAGGCACATTAAACCTGGCGGATCATTGTGGGTCAAGGCATAC  3780
Sbjct  3776  .............................G..............................  3835

Query  3781  GGCTACGCAGACCGGCACAGCGAGCACGTGGTCTTGGCATTGGCTAGAAAGTTTAAAAGC  3840
Sbjct  3836  .......................................C....................  3895

Query  3841  TTCAGAGTCACACAACCCTCATGCGTGACTTCCAACACCGAGGTGTTTCTCCACTTCTCA  3900
Sbjct  3896  ...........G........G.....A.................................  3955

Query  3901  ATTTTTGACAATGGCAAACGCGCGATAGCCCTGCATTCAGCTAATAGGAAGGCTAACAGT  3960
```

```
Sbjct  3956  ............................................................  4015

Query  3961  ATCTTCCAAAACACCTTCTTACCGGCGGGCAGTGCACCGGCGTACAGAGTCAAACGTGGA  4020
Sbjct  4016  ..........................A....A............................  4075

Query  4021  GACATTTCGAACGCCCCAGAGGATGCAGTGGTCAATGCAGCAAACCAACAGGGAGTGAAG  4080
Sbjct  4076  ............................................................  4135

Query  4081  GGTGCTGGAGTTTGCGGTGCAATTTACCGTAAGTGGCCGGACGCTTTCGGTGATGTCGCT  4140
Sbjct  4136  ............................................................  4195

Query  4141  ACTCCAACCGGAACAGCAGTTTCGAAATCCGTCCAAGATAAATTGGTGATCCACGCTGTC  4200
Sbjct  4196  ............................................................  4255

Query  4201  GGCCCGAATTTCTCAAAATGTTCAGAAGAGGAAGGGGACAGAGACCTAGCATCTGCTTAC  4260
Sbjct  4256  ...........T................................................  4315

Query  4261  AGAGCTGCAGCAGAAATAGTGATGGATaaaaaaaTTACAACAGTGGCCGTCCCCTTACTC  4320
Sbjct  4316  ..........................A...........................C.....  4375

Query  4321  TCCACCGGCATTTATGCCGGAGGAAAAAACAGAGTAGAACAGTCACTCAACCATCTCTTC  4380
Sbjct  4376  ..............C........G....................................  4435

Query  4381  ACGGCATTCGACAATACTGATGCAGATGTGACCATATATTGCATGGACAAAACATGGGAA  4440
Sbjct  4436  ........T...................................................  4495

Query  4441  AAGAAGATTAAGGAGGCAATCGATCACCGGACTTCGGTTGAGATGGTGCAGGATGACGTG  4500
Sbjct  4496  ............................................................  4555

Query  4501  CAGTTGGAGGAGGAACTGGTACGAGTACACCCTTTGAGTAGTTTAGCAGGTAGGAAGGGT  4560
Sbjct  4556  ......................................C.....................  4615

Query  4561  TACAGTACGGACAGCGGCCGAGTGTTTTCCTACCTGGAAGGTACCAAATTCCATCAGACT  4620
Sbjct  4616  ............................................................  4675

Query  4621  GCGGTGGACATAGCCGAAATGCAAGTGCTGTGGCCCGCCCTCAAAGAGTCTAATGAGCAA  4680
Sbjct  4676  ........T.............................T.....................  4735

Query  4681  ATAGTGGCATACACCTTAGGAGAATCAATGGACCAGATACGTGGCAAGTGCCCGACAGAA  4740
Sbjct  4736  ............................................................  4795

Query  4741  GATACTGACGCCTCCACACCTCCACGGACTGTGCCGTGCCTCTGTCGATACGCCATGACA  4800
Sbjct  4796  ............................................................  4855

Query  4801  CCAGAGAGAGTGTACCGACTTAAATGCACGAACACTACCCAATTTACGGTTTGCTCATCT  4860
Sbjct  4856  ............................................C.....C.........  4915

Query  4861  TTTGAGTTGCCAAAGTATCACATTCAGGGAGTGCAGAGAGTAAAATGTGAAAGAATCATC  4920
Sbjct  4916  ............................................................  4975

Query  4921  ATCTTAGATCCCACTGTTCCACCAACTTACAAACGGCCATGCATCAGACGGTACCCCTCC  4980
Sbjct  4976  ............................................................  5035

Query  4981  ACAATCTCTTGTAACTCCTCTGAGGACTCCAGGAGCTTGTCTACTTTTTCTGTCAGCTCC  5040
```

```
Sbjct  5036  ........C......................................C............  5095

Query  5041  GACTCCTCGATTGGTTCTCTGCCGGTCGGAGACACGAGACCCATTCCAGCCCCGAGGACC  5100
Sbjct  5096  .................CG..A...................T...........A......  5155

Query  5101  ATTTTCAGACCCGTCCCTGCCCCGAGAGCACCCGTGCTCAGAACCACACCGCCTCCTAAA  5160
Sbjct  5156  G...............................T..........T................  5215

Query  5161  CCACCGCGCACATTCACCGTGCGTGCAGAAGTGCACCAAGCACCCCCTACACCTGTACCT  5220
Sbjct  5216  ...................................T........................  5275

Query  5221  CCACCCAGACCGAAGAGGGCTGCAAAGTTGGCTCGTGAGATGCACCCCGGGTTCACCTTC  5280
Sbjct  5276  .....T......................................................  5335

Query  5281  GGGGACTTCGGAGAGCACGAGGTTGAGGAGCTTACGGCCTCTCCCTTAACCTTCGGAGAT  5340
Sbjct  5336  ..A..................A.................G....................  5395

Query  5341  TTTGCTGAAGGAGAGATCCAGGGGATGGGAGTGGAGTTTGAATGACTAGGCAGAGCCGGC  5400
Sbjct  5396  ....................A....C..................................  5455

Query  5401  GGGTACATTTTTTCGTCAGACACGGGTCCAGGCCACCTACAGCAGAGATCCGTTTTACAA  5460
Sbjct  5456  .........................................A.....G............  5515

Query  5461  AATTGCACGGCAGAATGTATCTACGAACCGGCAAAACTAGAAAAAATTCATGCACCAAAG  5520
Sbjct  5516  ............................................................  5575

Query  5521  TTGGATAAAACCAAGGAAGATATCTTAAGGAGCAAGTACCAAATGAAACCGTCTGAAGCA  5580
Sbjct  5576  ............................................................  5635

Query  5581  AACAAAAGCAGGTACCAATCTAGAAAAGTAGAAAATATGAAAGCAGAGATCGTAGGTAGA  5640
Sbjct  5636  ........T.........................................T.........  5695

Query  5641  CTCTTGGACGGACTGGGGGAGTATCTGGGCACCGAGCATCCAGTTGAATGCTACCGAATA  5700
Sbjct  5696  ..............A.............................................  5755

Query  5701  ACGTACCCGGTGCCTATATACTCAACTAGTGACCTCAGAGGTCTGTCTAGTGCCAAAACA  5760
Sbjct  5756  .....T....C...................AT..G.........................  5815

Query  5761  GCTGTTAGAGCTTGCAATGCATTTTTGGAAGCTAATTTTCCATCAGTCACTTCATATAAA  5820
Sbjct  5816  ............................................................  5875

Query  5821  ATTACTGATGAATACGACGCATACCTAGATATGGTAGATGGATCAGAGAGCTGTCTGGAC  5880
Sbjct  5876  ............................................................  5935

Query  5881  AGATCCTCCTTTTCGCCGTCTAGATTGCGTAGCTTTCCAAAAACACACTCATACTTGGAC  5940
Sbjct  5936  .................A...........C..............................  5995

Query  5941  CCACAGATCAACAGTGCGGTACCGTCACCATTCCAAAACACCTTACAAAATGTATTGGCA  6000
Sbjct  5996  ..G.........................................................  6055

Query  6001  GCGGCCACCAAAAGAAACTGTAATGTCACACAGATGAGAGAACTACCAACATATGATTCT  6060
Sbjct  6056  ...............................................G............  6115

Query  6061  GCAGTGCTAAATGTAGAGGCCTTCAGGAAATATGCGTGCAAGCCAGACGTATGGGATGAG  6120
```

```
Sbjct  6116  ............................................................  6175

Query  6121  TACAGGGATAATCCGATTTGCATAACCACCGAAAATGTCACCACTTACGTCGCCAAGTTG  6180
Sbjct  6176  ..............A..................................T..T......  6235

Query  6181  AAAGGACCGAAAGCTGCGGCCTTGTTTGCAAAAACACATAACCTGATACCACTACACCAA  6240
Sbjct  6236  ............................................................  6295

Query  6241  GTTCCTATGGACAAATTCACGGTAGATATGAAGAGAGATGTCAAAGTCACGCCCGGAACC  6300
Sbjct  6296  ............................................................  6355

Query  6301  AAGCACACCGAAGAGAGACCAAAGGTACAGGTGATTCAAGCGGCAGAGCCACTAGCCACT  6360
Sbjct  6356  ............................................................  6415

Query  6361  GCCTACCTCTGCGGAATTCACCGTGAATTGGTGCGCCGTCTCAACAACGCGCTTTTCCCA  6420
Sbjct  6416  ......................................................C.....T  6475

Query  6421  AACATCCACACTTTGTTTGATATGTCCGCAGAGGATTTCGATGCAATCATAGCGGAACAT  6480
Sbjct  6476  ..T.................................T.......................  6535

Query  6481  TTTAAGCACGGTGACCATGTGTTGGAAACGGATATAGCCTCTTTTGACAAAAGTCAAGAT  6540
Sbjct  6536  .................C..........................................  6595

Query  6541  GATTCCATGGCACTCACTGCGTTAATGATCCTTGAGGACCTGGGAGTAGACCAAAACCTA  6600
Sbjct  6596  ............................................................  6655

Query  6601  ATGAATTTGATAGAGGCTGCATTCGGGGAAATCGTGAGTACACACTTGCCCACAGGTACT  6660
Sbjct  6656  ............................................................  6715

Query  6661  AGATTCAAATTTGGAGCTATGATGAAGTCTGGAATGTTTTTGACGCTGTTCGTCAATACA  6720
Sbjct  6716  .......G....................C...............T.............  6775

Query  6721  ATTCTTAATGTGGTTATTGCGTGCCGAGTGTTGGAGGATCAATTGGCGCAGTCGCCGTGG  6780
Sbjct  6776  ........C..................................................C  6835

Query  6781  CCTGCTTTCATAGGAGATGACAACATAATCCATGGTATAATATCAGACAAATTGATGGCA  6840
Sbjct  6836  G..........................................................G  6895

Query  6841  GATAGATGTGCCACCTGGATGAACATGGAGGTCAAGATACTGGACTCTATAGTTGGAATA  6900
Sbjct  6896  ..............................................T..C...........  6955

Query  6901  CGGCCACCTTACTTCTGTGGAGGATTTATTGTATGTGACGATGTAACAGGTACAGCCTGC  6960
Sbjct  6956  ...........T................................................  7015

Query  6961  CGCGTCGCAGACCCACTGAAGAGATTGTTCAAGCTAGGTAAGCCATTGCCACTTGACGAT  7020
Sbjct  7016  ........................C...................................  7075

Query  7021  GGCCAAGATGAAGACAGAAGACGTGCATTACATGATGAAGTGAAAACCTGGTCGCGCGTA  7080
Sbjct  7076  ............................G...............................  7135

Query  7081  GGGCTGCGACACAGAGTGTGTGAAGCCATCGAAGACCGTTATGCCGTCCACTCATCAGAA  7140
Sbjct  7136  ..................................................T.........  7195

Query  7141  CTAGTTTTATTGGCACTGACTACTCTGTCTAAGAACTTGAAGTCCTTCAGAAACATAAGA  7200
```

```
Sbjct  7196  ............................................................  7255

Query  7201  GGGAAACCAATACATCTCTACGGTGGTCCTAAATAG  7236
Sbjct  7256  ....................................  7291
```

>Barmah Forest virus isolate SW93518, complete genome
Sequence ID: MN689041.1 Length: 11456
Range 1: 44 to 7279

Score:12656 bits(6853), Expect:0.0,
Identities:7108/7236(98%),  Gaps:0/7236(0%), Strand: Plus/Plus

```
Query  1     ATGGCGAAACCAGTTGTGAAGATCGACGTGGAACCTGAAAGCCATTTCGCTAAGCAGGTC  60
Sbjct  44    ..............................................T.............  103

Query  61    CAGAGTTGCTTCCCGCAGTTTGAGATCGAAGCAGTGCAGACCACACCAAACGATCATGCA  120
Sbjct  104   .........................................G..................  163

Query  121   CACGCGAGGGCGTTTTCGCACCTTGCTACGAAGCTCATAGAAATGGAGACAGCAAAAGAT  180
Sbjct  164   ............................................................  223

Query  181   CAGATCATCCTCGATATCGGAAGTGCACCCGCGAGGAGACTGTATTCAGAACACAAGTAC  240
Sbjct  224   ............................................................  283

Query  241   CACTGTGTTTGCCCAATGAAGTGCACGGAAGATCCAGAGAGAATGCTAGGATATGCACGT  300
Sbjct  284   ............................................................  343

Query  301   AAGTTGATCGCAGGCTCTGCGAAAGGGAAGGCAGAAAAGTTACGCGATCTCAGGGATGTC  360
Sbjct  344   ............................................................  403

Query  361   TTGGCTACGCCAGACATCGAGACGCAGTCGCTATGTCTCCACACAGACGCATCCTGCAGA  420
Sbjct  404   ............................................................  463

Query  421   TACCGCGGTGATGTTGCCGTGTATCAAGACGTGTATGCCATTGACGCACCTACCACGCTG  480
Sbjct  464   ..................................................C.........  523

Query  481   TACCACCAAGCGTTAAAGGGCGTCAGGACCGCATATTGGATAGGCTTTGATACAACGCCG  540
Sbjct  524   ...........................................................A  583

Query  541   TTCATGTACGATGCACTAGCAGGAGCTTACCCGCTCTACTCCACAAACTGGGCTGATGAG  600
Sbjct  584   .........A...........................................C......  643

Query  601   CAAGTGCTCGAGTCCAGAAACATTGGGCTATGTTCAGACAAAGTTTCTGAAGGGGGAAAG  660
Sbjct  644   ............................................................  703

Query  661   AAAGGGAGATCAATCCTCAGGAAGAAGTTCTTGAAGCAGTCAGACAGAGTCATGTTCTCT  720
Sbjct  704   ............................................................  763

Query  721   GTCGGCTCGACGTTGTATACGGAAAGCCGTAAATTACTGCAAAGTTGGCACCTGCCATCC  780
Sbjct  764   ............................................C...............  823
```

```
Query  781   ACATTCCATCTCAAAGGCAAATCTTCGTTCACGTGCCGCTGCGACACTATCGTCAGCTGC  840
Sbjct  824   ................................A..............C............  883

Query  841   GAAGGGTATGTTCTGAAGAAAATTACAATGTGTCCTGGAGTGACAGGCAAACCGATAGGA  900
Sbjct  884   ................................C..C..............T.........  943

Query  901   TATGCCGTCACCCATCACAAAGAAGGATTCGTAGTCGGAAAAGTCACAGATACCATTCGC  960
Sbjct  944   ....................G.............................C.........  1003

Query  961   GGCGAGAGAGTCTCCTTCGCCGTGTGTACTTATGTACCAACAACACTCTGCGACCAGATG  1020
Sbjct  1004  ............................................................  1063

Query  1021  ACCGGGATCCTAGCAACAGAAGTAACAGCCGATGATGCCCAGAAACTGCTGGTGGGTTTG  1080
Sbjct  1064  ........T...................................................  1123

Query  1081  AACCAGAGAATAGTAGTTAATGGTAGGACCCAGAGAAATACCAATACTATGAAGAACTAC  1140
Sbjct  1124  ............................................C...............  1183

Query  1141  CTGCTACCACTGGTTGCACAAGCGCTAGCAAAATGGGCGAAGGAAGCAAAACAGGATATG  1200
Sbjct  1184  ......................................A.....................  1243

Query  1201  GAAGATGAAAGACCCCTGAACGAACGCCAACGAACGCTAACGTGCCTCTGCTGCTGGGCA  1260
Sbjct  1244  ............................................................  1303

Query  1261  TTTAAGCGAAACAAACGCCACGCCATTTACAAGAGACCAGACACACAGAGTATAGTCAAG  1320
Sbjct  1304  ............................................................  1363

Query  1321  GTCCCTTGCGAATTCACAAGCTTTCCTTTGGTCAGCCTGTGGTCCGCTGGGATGTCTATA  1380
Sbjct  1364  ............................................................  1423

Query  1381  TCTCTTAGGCAGAAGTTGAAGATGATGCTGCAGGCGAGGCAGCCCACACAAATAGCAGCA  1440
Sbjct  1424  ............................................................  1483

Query  1441  GTGACTGAGGAACTCATACAAGAAGCAGCTGCAGTAGAGCAAGAGGCCGTGGATACGGCC  1500
Sbjct  1484  ..................................C.........................  1543

Query  1501  AATGCCGAGCTGGACCACGCCGCATGGCCCTCCATTGTGGATACGACAGAGCGCCATGTT  1560
Sbjct  1544  ...............................................G............  1603

Query  1561  GAGGTCGAAGTGGAAGAACTCGACCAGCGTGCAGGGGAAGGGGTAGTGGAAACACCTCGA  1620
Sbjct  1604  ............................................................  1663

Query  1621  AACTCTATCAAAGTTTCAACACAGATCGGGGACGCGTTAATCGGCAGTTACCTGATCCTA  1680
Sbjct  1664  ........T........T..........................................  1723

Query  1681  TCACCCCAAGCAGTCCTACGCAGCGAAAAATTAGCCTGCATACATGATCTTGCAGAGCAG  1740
Sbjct  1724  ............................................................  1783

Query  1741  GTTAAGTTGGTCACACACTCTGGCCGTAGTGGTAGGTACGCCGTCGACAAATACNACGGA  1800
Sbjct  1784  ..C.................C..T..............................G.....  1843

Query  1801  AGAGTACTAGTCCCTACAGGAGTGGCTATAGACATTCAATCGTTCCAGGCTCTCAGTGAG  1860
Sbjct  1844  ...................................C........................  1903
```

```
Query  1861  AGCGCGACCCTTGTGTACAACGAACGCGAGTTCGTTAACAGGAAGCTGTGGCACATAGCA  1920
Sbjct  1904  ..T..........................................T..............  1963

Query  1921  GTATACGGGGCAGCACTCAATACTGATGAAGAAGGATACGAGAAGGTCCCGGTAGAGAGA  1980
Sbjct  1964  ............................................................  2023

Query  1981  GCAGAATCAGATTATGTGTTTGATGTAGACCAAAAAATGTGCCTaaaaaaaGAGCAGGCA  2040
Sbjct  2024  .................A............A.............C...............  2083

Query  2041  TCAGGTTGGGTACTCTGTGGCGAACTAGTCAACCCCCCATTCCACGAATTCGCATATGAA  2100
Sbjct  2084  .....A......................................................  2143

Query  2101  GGGCTCCGCACGAGACCGTCAGCACCCTACAAGGTTCATACAGTAGGTGTGTACGGAGTG  2160
Sbjct  2144  ........T..T................................................  2203

Query  2161  CCAGGATCAGGCAAATCCGCAATAATCAAGAACACGGTCACCATGTCTGACCTAGTATTG  2220
Sbjct  2204  ............................................................  2263

Query  2221  AGTGGTAAGAAAGAGAACTGCTTAGAAATTATGAACGATGTACTTAAACACAGAGCTCTA  2280
Sbjct  2264  ............................................................  2323

Query  2281  CGTATCACAGCGAAGACCGTAGACTCAGTGTTATTAAACGGCGTGAAACACACGCCTAAC  2340
Sbjct  2324  .....T.............................G........................  2383

Query  2341  ATACTATACATCGACGAAGCGTTCTCATGCCATGCAGGGACTCTGTTGGCCACTATAGCC  2400
Sbjct  2384  ............................................................  2443

Query  2401  ATAGTCAGGCCCAAACAGAAAGTGGTACTGTGCGGAGACCCGAAACAATGCGGATTCTTC  2460
Sbjct  2444  T...........................................................  2503

Query  2461  AATATGATGCAACTGAAAGTTAATTACAATCATGACATCTGCTCAGAAGTCTTCCACAAA  2520
Sbjct  2504  .......................C....................................  2563

Query  2521  AGTATCTCTAGACGGTGCACCCAGGATATCACGGCCATCGTTTCCAAATTACATTACCAG  2580
Sbjct  2564  ............................................T...............  2623

Query  2581  GACCGAATGAGGACCACAAACCCCCGAAAAGGAGACATCATTATAGACACTACCGGCACT  2640
Sbjct  2624  ..................................................C.........  2683

Query  2641  ACCAAACCAGCCAAAACAGATCTGATTCTGACGTGCTTCAGGGGATGGGTGAAACAGTTG  2700
Sbjct  2684  ....................C.......................................  2743

Query  2701  CAGCAAGACTACAGAGGTAACGAAGTAATGACGGCTGCAGCGTCCCAAGGACTGACGAGG  2760
Sbjct  2744  ............................................................  2803

Query  2761  GCCTCCGTATATGCGGTTCGAACTAAAGTCAATGAGAACCCGCTATATGCACAGACCTCC  2820
Sbjct  2804  ............................................................  2863

Query  2821  GAGCACGTGAACGTGTTGTTAACACGCACAGAAAACAAGCTAGTATGGAAGACCTTGTCA  2880
Sbjct  2864  .......................................T....................  2923

Query  2881  ACAGATCCCTGGATTAAAACACTGACTAACCCACCTAGAGGGCACTATACCGCCACCATA  2940
Sbjct  2924  ............................................................  2983
```

```
Query  2941  GCAGAATGGGAAGCGGAACACCAGGGTATAATGAAGGCCATACAAGGGTATGCACCGCCC  3000
Sbjct  2984  ..............A.............................................  3043

Query  3001  GTGAACACCTTCATGAACAAAGTAAATGTGTGCTGGGCAAAGACACTTACGCCTGTGCTG  3060
Sbjct  3044  ...........T................................G..C..A.........  3103

Query  3061  GAAACTGCGGGTATCTCCCTGTCAGCAGAAGACTGGTCTGAACTGCTGCCCCCGTTTGCC  3120
Sbjct  3104  ............................................................  3163

Query  3121  CAGGACGTGGCGTACTCACCCGAGGTGGCATTAAACATCATATGCACGAAAATGTATGGG  3180
Sbjct  3164  ...............................................A............  3223

Query  3181  TTTGACTTAGACACTGGTCTTTTTTCCAGGCCATCAGTGCCAATGACATACACCAAAGAC  3240
Sbjct  3224  ........G.....C.............A.........A.....................  3283

Query  3241  CATTGGGATAACAGAGTTGGAGGGAAAATGTATGGATTCAGCCAACAAGCATACGATCAG  3300
Sbjct  3284  ............................................................  3343

Query  3301  CTGGCAAGACGACATCCGTACCTTCGAGGTAGAGAGAAATCAGGAATGCAGATCGTAGTC  3360
Sbjct  3344  ...................................A........................  3403

Query  3361  ACTGAAATGCGTATCCAGCGCCCAAGATCGGATGCCAACATCATCCCGATCAACCGCAGG  3420
Sbjct  3404  .....G......................................................  3463

Query  3421  CTCCCTCACTCACTCGTAGCCACACACGAGTATAGGCGAGCTGCACGGGCCGAGGAATTC  3480
Sbjct  3464  ............................................G...............  3523

Query  3481  TTCACCACGACACGAGGGTACACTATGCTGCTGGTCTCTGAGTATAACATGAACTTACCA  3540
Sbjct  3524  ..........T.................................................  3583

Query  3541  AACAAGAAGATCACCTGGCTGGCTCCGATAGGGACGCAGGGGGCCCATCACACCGCCAAC  3600
Sbjct  3584  ............................................................  3643

Query  3601  CTAAACTTGGGGATACCACCTCTGCTGGGCAGTTTTGATGCGGTGGTTGTGAACATGCCG  3660
Sbjct  3644  ........................................T...................  3703

Query  3661  ACTCCATTCCGGAACCATCACTACCAGCAATGTGAAGACCACGCGATGAAACTCCAGATG  3720
Sbjct  3704  ................................C...........................  3763

Query  3721  CTGGCAGGCGACGCACTGAGGCACATTAAACCTGGCGGATCATTGTGGGTCAAGGCATAC  3780
Sbjct  3764  .............................G..............................  3823

Query  3781  GGCTACGCAGACCGGCACAGCGAGCACGTGGTCTTGGCATTGGCTAGAAAGTTTAAAAGC  3840
Sbjct  3824  .......................................C....................  3883

Query  3841  TTCAGAGTCACACAACCCTCATGCGTGACTTCCAACACCGAGGTGTTTCTCCACTTCTCA  3900
Sbjct  3884  ...........G........G.....A.................................  3943

Query  3901  ATTTTTGACAATGGCAAACGCGCGATAGCCCTGCATTCAGCTAATAGGAAGGCTAACAGT  3960
Sbjct  3944  ............................................................  4003

Query  3961  ATCTTCCAAAACACCTTCTTACCGGCGGGCAGTGCACCGGCGTACAGAGTCAAACGTGGA  4020
Sbjct  4004  ..........................A....A............................  4063
```

```
Query  4021  GACATTTCGAACGCCCCAGAGGATGCAGTGGTCAATGCAGCAAACCAACAGGGAGTGAAG  4080
Sbjct  4064  ............................................................  4123

Query  4081  GGTGCTGGAGTTTGCGGTGCAATTTACCGTAAGTGGCCGGACGCTTTCGGTGATGTCGCT  4140
Sbjct  4124  ............................................................  4183

Query  4141  ACTCCAACCGGAACAGCAGTTTCGAAATCCGTCCAAGATAAATTGGTGATCCACGCTGTC  4200
Sbjct  4184  ............................................................  4243

Query  4201  GGCCCGAATTTCTCAAAATGTTCAGAAGAGGAAGGGGACAGAGACCTAGCATCTGCTTAC  4260
Sbjct  4244  ...........T................................................  4303

Query  4261  AGAGCTGCAGCAGAAATAGTGATGGATaaaaaaaTTACAACAGTGGCCGTCCCCTTACTC  4320
Sbjct  4304  ..........................A...........................C.....  4363

Query  4321  TCCACCGGCATTTATGCCGGAGGAAAAAACAGAGTAGAACAGTCACTCAACCATCTCTTC  4380
Sbjct  4364  ..............C........G....................................  4423

Query  4381  ACGGCATTCGACAATACTGATGCAGATGTGACCATATATTGCATGGACAAAACATGGGAA  4440
Sbjct  4424  ........T...................................................  4483

Query  4441  AAGAAGATTAAGGAGGCAATCGATCACCGGACTTCGGTTGAGATGGTGCAGGATGACGTG  4500
Sbjct  4484  ............................................................  4543

Query  4501  CAGTTGGAGGAGGAACTGGTACGAGTACACCCTTTGAGTAGTTTAGCAGGTAGGAAGGGT  4560
Sbjct  4544  ......................................C.....................  4603

Query  4561  TACAGTACGGACAGCGGCCGAGTGTTTTCCTACCTGGAAGGTACCAAATTCCATCAGACT  4620
Sbjct  4604  ............................................................  4663

Query  4621  GCGGTGGACATAGCCGAAATGCAAGTGCTGTGGCCCGCCCTCAAAGAGTCTAATGAGCAA  4680
Sbjct  4664  ........T.............................T.....................  4723

Query  4681  ATAGTGGCATACACCTTAGGAGAATCAATGGACCAGATACGTGGCAAGTGCCCGACAGAA  4740
Sbjct  4724  ............................................................  4783

Query  4741  GATACTGACGCCTCCACACCTCCACGGACTGTGCCGTGCCTCTGTCGATACGCCATGACA  4800
Sbjct  4784  ............................................................  4843

Query  4801  CCAGAGAGAGTGTACCGACTTAAATGCACGAACACTACCCAATTTACGGTTTGCTCATCT  4860
Sbjct  4844  ............................................C.....C.........  4903

Query  4861  TTTGAGTTGCCAAAGTATCACATTCAGGGAGTGCAGAGAGTAAAATGTGAAAGAATCATC  4920
Sbjct  4904  ............................................................  4963

Query  4921  ATCTTAGATCCCACTGTTCCACCAACTTACAAACGGCCATGCATCAGACGGTACCCCTCC  4980
Sbjct  4964  ............................................................  5023

Query  4981  ACAATCTCTTGTAACTCCTCTGAGGACTCCAGGAGCTTGTCTACTTTTTCTGTCAGCTCC  5040
Sbjct  5024  ........C......................................C............  5083

Query  5041  GACTCCTCGATTGGTTCTCTGCCGGTCGGAGACACGAGACCCATTCCAGCCCCGAGGACC  5100
Sbjct  5084  .................CG..A...................T...........A......  5143
```

```
Query  5101  ATTTTCAGACCCGTCCCTGCCCCGAGAGCACCCGTGCTCAGAACCACACCGCCTCCTAAA  5160
Sbjct  5144  G...............................T..........T................  5203

Query  5161  CCACCGCGCACATTCACCGTGCGTGCAGAAGTGCACCAAGCACCCCCTACACCTGTACCT  5220
Sbjct  5204  ...................................T........................  5263

Query  5221  CCACCCAGACCGAAGAGGGCTGCAAAGTTGGCTCGTGAGATGCACCCCGGGTTCACCTTC  5280
Sbjct  5264  .....T......................................................  5323

Query  5281  GGGGACTTCGGAGAGCACGAGGTTGAGGAGCTTACGGCCTCTCCCTTAACCTTCGGAGAT  5340
Sbjct  5324  ..A..................A.................G....................  5383

Query  5341  TTTGCTGAAGGAGAGATCCAGGGGATGGGAGTGGAGTTTGAATGACTAGGCAGAGCCGGC  5400
Sbjct  5384  ....................A....C..................................  5443

Query  5401  GGGTACATTTTTTCGTCAGACACGGGTCCAGGCCACCTACAGCAGAGATCCGTTTTACAA  5460
Sbjct  5444  .........................................A.....G............  5503

Query  5461  AATTGCACGGCAGAATGTATCTACGAACCGGCAAAACTAGAAAAAATTCATGCACCAAAG  5520
Sbjct  5504  ............................................................  5563

Query  5521  TTGGATAAAACCAAGGAAGATATCTTAAGGAGCAAGTACCAAATGAAACCGTCTGAAGCA  5580
Sbjct  5564  ............................................................  5623

Query  5581  AACAAAAGCAGGTACCAATCTAGAAAAGTAGAAAATATGAAAGCAGAGATCGTAGGTAGA  5640
Sbjct  5624  ........T.........................................T.........  5683

Query  5641  CTCTTGGACGGACTGGGGGAGTATCTGGGCACCGAGCATCCAGTTGAATGCTACCGAATA  5700
Sbjct  5684  ..............A.............................................  5743

Query  5701  ACGTACCCGGTGCCTATATACTCAACTAGTGACCTCAGAGGTCTGTCTAGTGCCAAAACA  5760
Sbjct  5744  .....T....C...................AT..G.........................  5803

Query  5761  GCTGTTAGAGCTTGCAATGCATTTTTGGAAGCTAATTTTCCATCAGTCACTTCATATAAA  5820
Sbjct  5804  ............................................................  5863

Query  5821  ATTACTGATGAATACGACGCATACCTAGATATGGTAGATGGATCAGAGAGCTGTCTGGAC  5880
Sbjct  5864  ............................................................  5923

Query  5881  AGATCCTCCTTTTCGCCGTCTAGATTGCGTAGCTTTCCAAAAACACACTCATACTTGGAC  5940
Sbjct  5924  .................A...........C..............................  5983

Query  5941  CCACAGATCAACAGTGCGGTACCGTCACCATTCCAAAACACCTTACAAAATGTATTGGCA  6000
Sbjct  5984  ..G.........................................................  6043

Query  6001  GCGGCCACCAAAAGAAACTGTAATGTCACACAGATGAGAGAACTACCAACATATGATTCT  6060
Sbjct  6044  ...............................................G............  6103

Query  6061  GCAGTGCTAAATGTAGAGGCCTTCAGGAAATATGCGTGCAAGCCAGACGTATGGGATGAG  6120
Sbjct  6104  ............................................................  6163

Query  6121  TACAGGGATAATCCGATTTGCATAACCACCGAAAATGTCACCACTTACGTCGCCAAGTTG  6180
Sbjct  6164  ..............A...................................T..T......  6223
```

```
Query  6181  AAAGGACCGAAAGCTGCGGCCTTGTTTGCAAAAACACATAACCTGATACCACTACACCAA  6240
Sbjct  6224  ............................................................  6283

Query  6241  GTTCCTATGGACAAATTCACGGTAGATATGAAGAGAGATGTCAAAGTCACGCCCGGAACC  6300
Sbjct  6284  ............................................................  6343

Query  6301  AAGCACACCGAAGAGAGACCAAAGGTACAGGTGATTCAAGCGGCAGAGCCACTAGCCACT  6360
Sbjct  6344  ............................................................  6403

Query  6361  GCCTACCTCTGCGGAATTCACCGTGAATTGGTGCGCCGTCTCAACAACGCGCTTTTCCCA  6420
Sbjct  6404  .....................................................C.....T  6463

Query  6421  AACATCCACACTTTGTTTGATATGTCCGCAGAGGATTTCGATGCAATCATAGCGGAACAT  6480
Sbjct  6464  ..T...................................T.....................  6523

Query  6481  TTTAAGCACGGTGACCATGTGTTGGAAACGGATATAGCCTCTTTTGACAAAAGTCAAGAT  6540
Sbjct  6524  .................C..........................................  6583

Query  6541  GATTCCATGGCACTCACTGCGTTAATGATCCTTGAGGACCTGGGAGTAGACCAAAACCTA  6600
Sbjct  6584  ............................................................  6643

Query  6601  ATGAATTTGATAGAGGCTGCATTCGGGGAAATCGTGAGTACACACTTGCCCACAGGTACT  6660
Sbjct  6644  ............................................................  6703

Query  6661  AGATTCAAATTTGGAGCTATGATGAAGTCTGGAATGTTTTTGACGCTGTTCGTCAATACA  6720
Sbjct  6704  .......G.....................C...............T..............  6763

Query  6721  ATTCTTAATGTGGTTATTGCGTGCCGAGTGTTGGAGGATCAATTGGCGCAGTCGCCGTGG  6780
Sbjct  6764  ........C..................................................C  6823

Query  6781  CCTGCTTTCATAGGAGATGACAACATAATCCATGGTATAATATCAGACAAATTGATGGCA  6840
Sbjct  6824  G..........................................................G  6883

Query  6841  GATAGATGTGCCACCTGGATGAACATGGAGGTCAAGATACTGGACTCTATAGTTGGAATA  6900
Sbjct  6884  ............................................T..C............  6943

Query  6901  CGGCCACCTTACTTCTGTGGAGGATTTATTGTATGTGACGATGTAACAGGTACAGCCTGC  6960
Sbjct  6944  ...........T................................................  7003

Query  6961  CGCGTCGCAGACCCACTGAAGAGATTGTTCAAGCTAGGTAAGCCATTGCCACTTGACGAT  7020
Sbjct  7004  ........................C...................................  7063

Query  7021  GGCCAAGATGAAGACAGAAGACGTGCATTACATGATGAAGTGAAAACCTGGTCGCGCGTA  7080
Sbjct  7064  .............................G..............................  7123

Query  7081  GGGCTGCGACACAGAGTGTGTGAAGCCATCGAAGACCGTTATGCCGTCCACTCATCAGAA  7140
Sbjct  7124  ..................................................T.........  7183

Query  7141  CTAGTTTTATTGGCACTGACTACTCTGTCTAAGAACTTGAAGTCCTTCAGAAACATAAGA  7200
Sbjct  7184  ............................................................  7243

Query  7201  GGGAAACCAATACATCTCTACGGTGGTCCTAAATAG  7236
Sbjct  7244  ....................................  7279
```

```
>Barmah Forest virus isolate DC30314, complete genome
Sequence ID: MN689021.1 Length: 11498
Range 1: 55 to 7290

Score:12656 bits(6853), Expect:0.0,
Identities:7108/7236(98%),  Gaps:0/7236(0%), Strand: Plus/Plus

Query  1     ATGGCGAAACCAGTTGTGAAGATCGACGTGGAACCTGAAAGCCATTTCGCTAAGCAGGTC  60
Sbjct  55    ...............................................T............  114

Query  61    CAGAGTTGCTTCCCGCAGTTTGAGATCGAAGCAGTGCAGACCACACCAAACGATCATGCA  120
Sbjct  115   .........................................G..................  174

Query  121   CACGCGAGGGCGTTTTCGCACCTTGCTACGAAGCTCATAGAAATGGAGACAGCAAAAGAT  180
Sbjct  175   ............................................................  234

Query  181   CAGATCATCCTCGATATCGGAAGTGCACCCGCGAGGAGACTGTATTCAGAACACAAGTAC  240
Sbjct  235   ............................................................  294

Query  241   CACTGTGTTTGCCCAATGAAGTGCACGGAAGATCCAGAGAGAATGCTAGGATATGCACGT  300
Sbjct  295   ............................................................  354

Query  301   AAGTTGATCGCAGGCTCTGCGAAAGGGAAGGCAGAAAAGTTACGCGATCTCAGGGATGTC  360
Sbjct  355   ............................................................  414

Query  361   TTGGCTACGCCAGACATCGAGACGCAGTCGCTATGTCTCCACACAGACGCATCCTGCAGA  420
Sbjct  415   ............................................................  474

Query  421   TACCGCGGTGATGTTGCCGTGTATCAAGACGTGTATGCCATTGACGCACCTACCACGCTG  480
Sbjct  475   ..................................................C.........  534

Query  481   TACCACCAAGCGTTAAAGGGCGTCAGGACCGCATATTGGATAGGCTTTGATACAACGCCG  540
Sbjct  535   ...........................................................A  594

Query  541   TTCATGTACGATGCACTAGCAGGAGCTTACCCGCTCTACTCCACAAACTGGGCTGATGAG  600
Sbjct  595   .........A...........................................C......  654

Query  601   CAAGTGCTCGAGTCCAGAAACATTGGGCTATGTTCAGACAAAGTTTCTGAAGGGGGAAAG  660
Sbjct  655   ............................................................  714

Query  661   AAAGGGAGATCAATCCTCAGGAAGAAGTTCTTGAAGCAGTCAGACAGAGTCATGTTCTCT  720
Sbjct  715   ............................................................  774

Query  721   GTCGGCTCGACGTTGTATACGGAAAGCCGTAAATTACTGCAAAGTTGGCACCTGCCATCC  780
Sbjct  775   ............................................C...............  834

Query  781   ACATTCCATCTCAAAGGCAAATCTTCGTTCACGTGCCGCTGCGACACTATCGTCAGCTGC  840
Sbjct  835   ................................A..............C............  894

Query  841   GAAGGGTATGTTCTGAAGAAAATTACAATGTGTCCTGGAGTGACAGGCAAACCGATAGGA  900
Sbjct  895   ................................C..C........................  954
```

```
Query  901   TATGCCGTCACCCATCACAAAGAAGGATTCGTAGTCGGAAAAGTCACAGATACCATTCGC  960
Sbjct  955   ....................G.............................C.........  1014

Query  961   GGCGAGAGAGTCTCCTTCGCCGTGTGTACTTATGTACCAACAACACTCTGCGACCAGATG  1020
Sbjct  1015  ............................................................  1074

Query  1021  ACCGGGATCCTAGCAACAGAAGTAACAGCCGATGATGCCCAGAAACTGCTGGTGGGTTTG  1080
Sbjct  1075  ........T...................................................  1134

Query  1081  AACCAGAGAATAGTAGTTAATGGTAGGACCCAGAGAAATACCAATACTATGAAGAACTAC  1140
Sbjct  1135  ............................................C...............  1194

Query  1141  CTGCTACCACTGGTTGCACAAGCGCTAGCAAAATGGGCGAAGGAAGCAAAACAGGATATG  1200
Sbjct  1195  ......................................A.....................  1254

Query  1201  GAAGATGAAAGACCCCTGAACGAACGCCAACGAACGCTAACGTGCCTCTGCTGCTGGGCA  1260
Sbjct  1255  ............................................................  1314

Query  1261  TTTAAGCGAAACAAACGCCACGCCATTTACAAGAGACCAGACACACAGAGTATAGTCAAG  1320
Sbjct  1315  ............................................................  1374

Query  1321  GTCCCTTGCGAATTCACAAGCTTTCCTTTGGTCAGCCTGTGGTCCGCTGGGATGTCTATA  1380
Sbjct  1375  ............................................................  1434

Query  1381  TCTCTTAGGCAGAAGTTGAAGATGATGCTGCAGGCGAGGCAGCCCACACAAATAGCAGCA  1440
Sbjct  1435  ............................................................  1494

Query  1441  GTGACTGAGGAACTCATACAAGAAGCAGCTGCAGTAGAGCAAGAGGCCGTGGATACGGCC  1500
Sbjct  1495  ............T.....................C.........................  1554

Query  1501  AATGCCGAGCTGGACCACGCCGCATGGCCCTCCATTGTGGATACGACAGAGCGCCATGTT  1560
Sbjct  1555  ...............................................G............  1614

Query  1561  GAGGTCGAAGTGGAAGAACTCGACCAGCGTGCAGGGGAAGGGGTAGTGGAAACACCTCGA  1620
Sbjct  1615  ............................................................  1674

Query  1621  AACTCTATCAAAGTTTCAACACAGATCGGGGACGCGTTAATCGGCAGTTACCTGATCCTA  1680
Sbjct  1675  ........T...................................................  1734

Query  1681  TCACCCCAAGCAGTCCTACGCAGCGAAAAATTAGCCTGCATACATGATCTTGCAGAGCAG  1740
Sbjct  1735  ............................................................  1794

Query  1741  GTTAAGTTGGTCACACACTCTGGCCGTAGTGGTAGGTACGCCGTCGACAAATACNACGGA  1800
Sbjct  1795  ..C.................C..T..............................G.....  1854

Query  1801  AGAGTACTAGTCCCTACAGGAGTGGCTATAGACATTCAATCGTTCCAGGCTCTCAGTGAG  1860
Sbjct  1855  ...................................C........................  1914

Query  1861  AGCGCGACCCTTGTGTACAACGAACGCGAGTTCGTTAACAGGAAGCTGTGGCACATAGCA  1920
Sbjct  1915  ..T..........................................T..............  1974

Query  1921  GTATACGGGGCAGCACTCAATACTGATGAAGAAGGATACGAGAAGGTCCCGGTAGAGAGA  1980
Sbjct  1975  ............................................................  2034
```

```
Query  1981  GCAGAATCAGATTATGTGTTTGATGTAGACCAAAAAATGTGCCTaaaaaaaGAGCAGGCA  2040
Sbjct  2035  .................A............A.............C...............  2094

Query  2041  TCAGGTTGGGTACTCTGTGGCGAACTAGTCAACCCCCCATTCCACGAATTCGCATATGAA  2100
Sbjct  2095  .....A......................................................  2154

Query  2101  GGGCTCCGCACGAGACCGTCAGCACCCTACAAGGTTCATACAGTAGGTGTGTACGGAGTG  2160
Sbjct  2155  ........T..T................................................  2214

Query  2161  CCAGGATCAGGCAAATCCGCAATAATCAAGAACACGGTCACCATGTCTGACCTAGTATTG  2220
Sbjct  2215  ............................................................  2274

Query  2221  AGTGGTAAGAAAGAGAACTGCTTAGAAATTATGAACGATGTACTTAAACACAGAGCTCTA  2280
Sbjct  2275  ............................................................  2334

Query  2281  CGTATCACAGCGAAGACCGTAGACTCAGTGTTATTAAACGGCGTGAAACACACGCCTAAC  2340
Sbjct  2335  .....T.............................G........................  2394

Query  2341  ATACTATACATCGACGAAGCGTTCTCATGCCATGCAGGGACTCTGTTGGCCACTATAGCC  2400
Sbjct  2395  ............................................................  2454

Query  2401  ATAGTCAGGCCCAAACAGAAAGTGGTACTGTGCGGAGACCCGAAACAATGCGGATTCTTC  2460
Sbjct  2455  T..........................................................T  2514

Query  2461  AATATGATGCAACTGAAAGTTAATTACAATCATGACATCTGCTCAGAAGTCTTCCACAAA  2520
Sbjct  2515  .......................C....................................  2574

Query  2521  AGTATCTCTAGACGGTGCACCCAGGATATCACGGCCATCGTTTCCAAATTACATTACCAG  2580
Sbjct  2575  ............................................T...............  2634

Query  2581  GACCGAATGAGGACCACAAACCCCCGAAAAGGAGACATCATTATAGACACTACCGGCACT  2640
Sbjct  2635  ..................................................C.........  2694

Query  2641  ACCAAACCAGCCAAAACAGATCTGATTCTGACGTGCTTCAGGGGATGGGTGAAACAGTTG  2700
Sbjct  2695  ....................C.......................................  2754

Query  2701  CAGCAAGACTACAGAGGTAACGAAGTAATGACGGCTGCAGCGTCCCAAGGACTGACGAGG  2760
Sbjct  2755  ............................................................  2814

Query  2761  GCCTCCGTATATGCGGTTCGAACTAAAGTCAATGAGAACCCGCTATATGCACAGACCTCC  2820
Sbjct  2815  ............................................................  2874

Query  2821  GAGCACGTGAACGTGTTGTTAACACGCACAGAAAACAAGCTAGTATGGAAGACCTTGTCA  2880
Sbjct  2875  ..................C....................T....................  2934

Query  2881  ACAGATCCCTGGATTAAAACACTGACTAACCCACCTAGAGGGCACTATACCGCCACCATA  2940
Sbjct  2935  ............................................................  2994

Query  2941  GCAGAATGGGAAGCGGAACACCAGGGTATAATGAAGGCCATACAAGGGTATGCACCGCCC  3000
Sbjct  2995  ..............A.......................T.....................  3054

Query  3001  GTGAACACCTTCATGAACAAAGTAAATGTGTGCTGGGCAAAGACACTTACGCCTGTGCTG  3060
Sbjct  3055  ...............................................C..A.........  3114
```

```
Query  3061  GAAACTGCGGGTATCTCCCTGTCAGCAGAAGACTGGTCTGAACTGCTGCCCCCGTTTGCC  3120
Sbjct  3115  ............................................................  3174

Query  3121  CAGGACGTGGCGTACTCACCCGAGGTGGCATTAAACATCATATGCACGAAAATGTATGGG  3180
Sbjct  3175  ...............................................A............  3234

Query  3181  TTTGACTTAGACACTGGTCTTTTTTCCAGGCCATCAGTGCCAATGACATACACCAAAGAC  3240
Sbjct  3235  ........G.....C.............A.........A.....................  3294

Query  3241  CATTGGGATAACAGAGTTGGAGGGAAAATGTATGGATTCAGCCAACAAGCATACGATCAG  3300
Sbjct  3295  ............................................................  3354

Query  3301  CTGGCAAGACGACATCCGTACCTTCGAGGTAGAGAGAAATCAGGAATGCAGATCGTAGTC  3360
Sbjct  3355  ...................................A........................  3414

Query  3361  ACTGAAATGCGTATCCAGCGCCCAAGATCGGATGCCAACATCATCCCGATCAACCGCAGG  3420
Sbjct  3415  .....G......................................................  3474

Query  3421  CTCCCTCACTCACTCGTAGCCACACACGAGTATAGGCGAGCTGCACGGGCCGAGGAATTC  3480
Sbjct  3475  ............................................G...............  3534

Query  3481  TTCACCACGACACGAGGGTACACTATGCTGCTGGTCTCTGAGTATAACATGAACTTACCA  3540
Sbjct  3535  ..........T.................................................  3594

Query  3541  AACAAGAAGATCACCTGGCTGGCTCCGATAGGGACGCAGGGGGCCCATCACACCGCCAAC  3600
Sbjct  3595  ............................................................  3654

Query  3601  CTAAACTTGGGGATACCACCTCTGCTGGGCAGTTTTGATGCGGTGGTTGTGAACATGCCG  3660
Sbjct  3655  ............................................................  3714

Query  3661  ACTCCATTCCGGAACCATCACTACCAGCAATGTGAAGACCACGCGATGAAACTCCAGATG  3720
Sbjct  3715  ................................C...........................  3774

Query  3721  CTGGCAGGCGACGCACTGAGGCACATTAAACCTGGCGGATCATTGTGGGTCAAGGCATAC  3780
Sbjct  3775  ............................................................  3834

Query  3781  GGCTACGCAGACCGGCACAGCGAGCACGTGGTCTTGGCATTGGCTAGAAAGTTTAAAAGC  3840
Sbjct  3835  .......................................C....................  3894

Query  3841  TTCAGAGTCACACAACCCTCATGCGTGACTTCCAACACCGAGGTGTTTCTCCACTTCTCA  3900
Sbjct  3895  ...........G.....A..G.....A.................................  3954

Query  3901  ATTTTTGACAATGGCAAACGCGCGATAGCCCTGCATTCAGCTAATAGGAAGGCTAACAGT  3960
Sbjct  3955  ............................................................  4014

Query  3961  ATCTTCCAAAACACCTTCTTACCGGCGGGCAGTGCACCGGCGTACAGAGTCAAACGTGGA  4020
Sbjct  4015  ..........................A....A............................  4074

Query  4021  GACATTTCGAACGCCCCAGAGGATGCAGTGGTCAATGCAGCAAACCAACAGGGAGTGAAG  4080
Sbjct  4075  ............................................................  4134

Query  4081  GGTGCTGGAGTTTGCGGTGCAATTTACCGTAAGTGGCCGGACGCTTTCGGTGATGTCGCT  4140
Sbjct  4135  ............................................................  4194
```

```
Query  4141  ACTCCAACCGGAACAGCAGTTTCGAAATCCGTCCAAGATAAATTGGTGATCCACGCTGTC  4200
Sbjct  4195  ............................................................  4254

Query  4201  GGCCCGAATTTCTCAAAATGTTCAGAAGAGGAAGGGGACAGAGACCTAGCATCTGCTTAC  4260
Sbjct  4255  ............................................................  4314

Query  4261  AGAGCTGCAGCAGAAATAGTGATGGATaaaaaaTTACAACAGTGGCCGTCCCCTTACTC  4320
Sbjct  4315  ..........................A.................................  4374

Query  4321  TCCACCGGCATTTATGCCGGAGGAAAAAACAGAGTAGAACAGTCACTCAACCATCTCTTC  4380
Sbjct  4375  ..............C........G....................................  4434

Query  4381  ACGGCATTCGACAATACTGATGCAGATGTGACCATATATTGCATGGACAAAACATGGGAA  4440
Sbjct  4435  ........T...................................................  4494

Query  4441  AAGAAGATTAAGGAGGCAATCGATCACCGGACTTCGGTTGAGATGGTGCAGGATGACGTG  4500
Sbjct  4495  ............................................................  4554

Query  4501  CAGTTGGAGGAGGAACTGGTACGAGTACACCCTTTGAGTAGTTTAGCAGGTAGGAAGGGT  4560
Sbjct  4555  ............................................................  4614

Query  4561  TACAGTACGGACAGCGGCCGAGTGTTTTCCTACCTGGAAGGTACCAAATTCCATCAGACT  4620
Sbjct  4615  ............................................................  4674

Query  4621  GCGGTGGACATAGCCGAAATGCAAGTGCTGTGGCCCGCCCTCAAAGAGTCTAATGAGCAA  4680
Sbjct  4675  ........T.............................T.....................  4734

Query  4681  ATAGTGGCATACACCTTAGGAGAATCAATGGACCAGATACGTGGCAAGTGCCCGACAGAA  4740
Sbjct  4735  ............................................................  4794

Query  4741  GATACTGACGCCTCCACACCTCCACGGACTGTGCCGTGCCTCTGTCGATACGCCATGACA  4800
Sbjct  4795  ..C.........A...............................................  4854

Query  4801  CCAGAGAGAGTGTACCGACTTAAATGCACGAACACTACCCAATTTACGGTTTGCTCATCT  4860
Sbjct  4855  ............................................C.....C.........  4914

Query  4861  TTTGAGTTGCCAAAGTATCACATTCAGGGAGTGCAGAGAGTAAAATGTGAAAGAATCATC  4920
Sbjct  4915  ............................................................  4974

Query  4921  ATCTTAGATCCCACTGTTCCACCAACTTACAAACGGCCATGCATCAGACGGTACCCCTCC  4980
Sbjct  4975  ............................................................  5034

Query  4981  ACAATCTCTTGTAACTCCTCTGAGGACTCCAGGAGCTTGTCTACTTTTTCTGTCAGCTCC  5040
Sbjct  5035  ........C......................................C............  5094

Query  5041  GACTCCTCGATTGGTTCTCTGCCGGTCGGAGACACGAGACCCATTCCAGCCCCGAGGACC  5100
Sbjct  5095  .................CG..A...................T...........A.....T  5154

Query  5101  ATTTTCAGACCCGTCCCTGCCCCGAGAGCACCCGTGCTCAGAACCACACCGCCTCCTAAA  5160
Sbjct  5155  G..........................................T................  5214

Query  5161  CCACCGCGCACATTCACCGTGCGTGCAGAAGTGCACCAAGCACCCCCTACACCTGTACCT  5220
Sbjct  5215  ...................................T........................  5274
```

```
Query  5221  CCACCCAGACCGAAGAGGGCTGCAAAGTTGGCTCGTGAGATGCACCCCGGGTTCACCTTC  5280
Sbjct  5275  ............................................................  5334

Query  5281  GGGGACTTCGGAGAGCACGAGGTTGAGGAGCTTACGGCCTCTCCCTTAACCTTCGGAGAT  5340
Sbjct  5335  ..A..................A.................G....................  5394

Query  5341  TTTGCTGAAGGAGAGATCCAGGGGATGGGAGTGGAGTTTGAATGACTAGGCAGAGCCGGC  5400
Sbjct  5395  ............................................................  5454

Query  5401  GGGTACATTTTTTCGTCAGACACGGGTCCAGGCCACCTACAGCAGAGATCCGTTTTACAA  5460
Sbjct  5455  .........................................A..................  5514

Query  5461  AATTGCACGGCAGAATGTATCTACGAACCGGCAAAACTAGAAAAAATTCATGCACCAAAG  5520
Sbjct  5515  ............................................................  5574

Query  5521  TTGGATAAAACCAAGGAAGATATCTTAAGGAGCAAGTACCAAATGAAACCGTCTGAAGCA  5580
Sbjct  5575  ........G...................................................  5634

Query  5581  AACAAAAGCAGGTACCAATCTAGAAAAGTAGAAAATATGAAAGCAGAGATCGTAGGTAGA  5640
Sbjct  5635  ........T.........................................T.........  5694

Query  5641  CTCTTGGACGGACTGGGGGAGTATCTGGGCACCGAGCATCCAGTTGAATGCTACCGAATA  5700
Sbjct  5695  ..............A.......................C.....................  5754

Query  5701  ACGTACCCGGTGCCTATATACTCAACTAGTGACCTCAGAGGTCTGTCTAGTGCCAAAACA  5760
Sbjct  5755  .....T........................AT..G.........................  5814

Query  5761  GCTGTTAGAGCTTGCAATGCATTTTTGGAAGCTAATTTTCCATCAGTCACTTCATATAAA  5820
Sbjct  5815  ............................................................  5874

Query  5821  ATTACTGATGAATACGACGCATACCTAGATATGGTAGATGGATCAGAGAGCTGTCTGGAC  5880
Sbjct  5875  ............................................................  5934

Query  5881  AGATCCTCCTTTTCGCCGTCTAGATTGCGTAGCTTTCCAAAAACACACTCATACTTGGAC  5940
Sbjct  5935  .................A...........C..............................  5994

Query  5941  CCACAGATCAACAGTGCGGTACCGTCACCATTCCAAAACACCTTACAAAATGTATTGGCA  6000
Sbjct  5995  ..G.......................G.................................  6054

Query  6001  GCGGCCACCAAAAGAAACTGTAATGTCACACAGATGAGAGAACTACCAACATATGATTCT  6060
Sbjct  6055  ...............................................G............  6114

Query  6061  GCAGTGCTAAATGTAGAGGCCTTCAGGAAATATGCGTGCAAGCCAGACGTATGGGATGAG  6120
Sbjct  6115  ............................................................  6174

Query  6121  TACAGGGATAATCCGATTTGCATAACCACCGAAAATGTCACCACTTACGTCGCCAAGTTG  6180
Sbjct  6175  ..............A......................................T......  6234

Query  6181  AAAGGACCGAAAGCTGCGGCCTTGTTTGCAAAAACACATAACCTGATACCACTACACCAA  6240
Sbjct  6235  ............................................................  6294

Query  6241  GTTCCTATGGACAAATTCACGGTAGATATGAAGAGAGATGTCAAAGTCACGCCCGGAACC  6300
Sbjct  6295  ............................................................  6354
```

```
Query  6301  AAGCACACCGAAGAGAGACCAAAGGTACAGGTGATTCAAGCGGCAGAGCCACTAGCCACT  6360
Sbjct  6355  ...................................................T........  6414

Query  6361  GCCTACCTCTGCGGAATTCACCGTGAATTGGTGCGCCGTCTCAACAACGCGCTTTTCCCA  6420
Sbjct  6415  .....................................................C......  6474

Query  6421  AACATCCACACTTTGTTTGATATGTCCGCAGAGGATTTCGATGCAATCATAGCGGAACAT  6480
Sbjct  6475  ..T.............................A..C..T.....................  6534

Query  6481  TTTAAGCACGGTGACCATGTGTTGGAAACGGATATAGCCTCTTTTGACAAAAGTCAAGAT  6540
Sbjct  6535  .................C..........................................  6594

Query  6541  GATTCCATGGCACTCACTGCGTTAATGATCCTTGAGGACCTGGGAGTAGACCAAAACCTA  6600
Sbjct  6595  ............................................................  6654

Query  6601  ATGAATTTGATAGAGGCTGCATTCGGGGAAATCGTGAGTACACACTTGCCCACAGGTACT  6660
Sbjct  6655  ............................................................  6714

Query  6661  AGATTCAAATTTGGAGCTATGATGAAGTCTGGAATGTTTTTGACGCTGTTCGTCAATACA  6720
Sbjct  6715  .......G.....................C...............T..............  6774

Query  6721  ATTCTTAATGTGGTTATTGCGTGCCGAGTGTTGGAGGATCAATTGGCGCAGTCGCCGTGG  6780
Sbjct  6775  ........C......................................A...........C  6834

Query  6781  CCTGCTTTCATAGGAGATGACAACATAATCCATGGTATAATATCAGACAAATTGATGGCA  6840
Sbjct  6835  G..........................................................G  6894

Query  6841  GATAGATGTGCCACCTGGATGAACATGGAGGTCAAGATACTGGACTCTATAGTTGGAATA  6900
Sbjct  6895  ..............T.............................T..C............  6954

Query  6901  CGGCCACCTTACTTCTGTGGAGGATTTATTGTATGTGACGATGTAACAGGTACAGCCTGC  6960
Sbjct  6955  ...........T................................................  7014

Query  6961  CGCGTCGCAGACCCACTGAAGAGATTGTTCAAGCTAGGTAAGCCATTGCCACTTGACGAT  7020
Sbjct  7015  ..............G.........C...................................  7074

Query  7021  GGCCAAGATGAAGACAGAAGACGTGCATTACATGATGAAGTGAAAACCTGGTCGCGCGTA  7080
Sbjct  7075  ........C...................................................  7134

Query  7081  GGGCTGCGACACAGAGTGTGTGAAGCCATCGAAGACCGTTATGCCGTCCACTCATCAGAA  7140
Sbjct  7135  ..................................................T.........  7194

Query  7141  CTAGTTTTATTGGCACTGACTACTCTGTCTAAGAACTTGAAGTCCTTCAGAAACATAAGA  7200
Sbjct  7195  ............................................................  7254

Query  7201  GGGAAACCAATACATCTCTACGGTGGTCCTAAATAG  7236
Sbjct  7255  ....................................  7290
```

>Barmah Forest virus strain SW94401, complete genome
Sequence ID: MW835348.1 Length: 11468

```
Range 1: 54 to 7289

Score:12650 bits(6850), Expect:0.0,
Identities:7107/7236(98%),  Gaps:0/7236(0%), Strand: Plus/Plus

Query  1     ATGGCGAAACCAGTTGTGAAGATCGACGTGGAACCTGAAAGCCATTTCGCTAAGCAGGTC  60
Sbjct  54    ...............................................T............  113

Query  61    CAGAGTTGCTTCCCGCAGTTTGAGATCGAAGCAGTGCAGACCACACCAAACGATCATGCA  120
Sbjct  114   .........................................G..................  173

Query  121   CACGCGAGGGCGTTTTCGCACCTTGCTACGAAGCTCATAGAAATGGAGACAGCAAAAGAT  180
Sbjct  174   ............................................................  233

Query  181   CAGATCATCCTCGATATCGGAAGTGCACCCGCGAGGAGACTGTATTCAGAACACAAGTAC  240
Sbjct  234   ............................................................  293

Query  241   CACTGTGTTTGCCCAATGAAGTGCACGGAAGATCCAGAGAGAATGCTAGGATATGCACGT  300
Sbjct  294   ............................................................  353

Query  301   AAGTTGATCGCAGGCTCTGCGAAAGGGAAGGCAGAAAAGTTACGCGATCTCAGGGATGTC  360
Sbjct  354   ............................................................  413

Query  361   TTGGCTACGCCAGACATCGAGACGCAGTCGCTATGTCTCCACACAGACGCATCCTGCAGA  420
Sbjct  414   ............................................................  473

Query  421   TACCGCGGTGATGTTGCCGTGTATCAAGACGTGTATGCCATTGACGCACCTACCACGCTG  480
Sbjct  474   ..................................................C.........  533

Query  481   TACCACCAAGCGTTAAAGGGCGTCAGGACCGCATATTGGATAGGCTTTGATACAACGCCG  540
Sbjct  534   ...........................................................A  593

Query  541   TTCATGTACGATGCACTAGCAGGAGCTTACCCGCTCTACTCCACAAACTGGGCTGATGAG  600
Sbjct  594   .........A...........................................C......  653

Query  601   CAAGTGCTCGAGTCCAGAAACATTGGGCTATGTTCAGACAAAGTTTCTGAAGGGGGAAAG  660
Sbjct  654   ............................................................  713

Query  661   AAAGGGAGATCAATCCTCAGGAAGAAGTTCTTGAAGCAGTCAGACAGAGTCATGTTCTCT  720
Sbjct  714   .................................G..........................  773

Query  721   GTCGGCTCGACGTTGTATACGGAAAGCCGTAAATTACTGCAAAGTTGGCACCTGCCATCC  780
Sbjct  774   ............................................C......T........  833

Query  781   ACATTCCATCTCAAAGGCAAATCTTCGTTCACGTGCCGCTGCGACACTATCGTCAGCTGC  840
Sbjct  834   ................................A..............C............  893

Query  841   GAAGGGTATGTTCTGAAGAAAATTACAATGTGTCCTGGAGTGACAGGCAAACCGATAGGA  900
Sbjct  894   ................................C..C........................  953

Query  901   TATGCCGTCACCCATCACAAAGAAGGATTCGTAGTCGGAAAAGTCACAGATACCATTCGC  960
Sbjct  954   ....................G.............................C.........  1013

Query  961   GGCGAGAGAGTCTCCTTCGCCGTGTGTACTTATGTACCAACAACACTCTGCGACCAGATG  1020
```

```
Sbjct  1014  ............................................................  1073

Query  1021  ACCGGGATCCTAGCAACAGAAGTAACAGCCGATGATGCCCAGAAACTGCTGGTGGGTTTG  1080
Sbjct  1074  ........T...................................................  1133

Query  1081  AACCAGAGAATAGTAGTTAATGGTAGGACCCAGAGAAATACCAATACTATGAAGAACTAC  1140
Sbjct  1134  ............................................C...............  1193

Query  1141  CTGCTACCACTGGTTGCACAAGCGCTAGCAAAATGGGCGAAGGAAGCAAAACAGGATATG  1200
Sbjct  1194  ......................................A.....................  1253

Query  1201  GAAGATGAAAGACCCCTGAACGAACGCCAACGAACGCTAACGTGCCTCTGCTGCTGGGCA  1260
Sbjct  1254  ............................................................  1313

Query  1261  TTTAAGCGAAACAAACGCCACGCCATTTACAAGAGACCAGACACACAGAGTATAGTCAAG  1320
Sbjct  1314  ............................................................  1373

Query  1321  GTCCCTTGCGAATTCACAAGCTTTCCTTTGGTCAGCCTGTGGTCCGCTGGGATGTCTATA  1380
Sbjct  1374  ............................................................  1433

Query  1381  TCTCTTAGGCAGAAGTTGAAGATGATGCTGCAGGCGAGGCAGCCCACACAAATAGCAGCA  1440
Sbjct  1434  ............................................................  1493

Query  1441  GTGACTGAGGAACTCATACAAGAAGCAGCTGCAGTAGAGCAAGAGGCCGTGGATACGGCC  1500
Sbjct  1494  ..................................C.........................  1553

Query  1501  AATGCCGAGCTGGACCACGCCGCATGGCCCTCCATTGTGGATACGACAGAGCGCCATGTT  1560
Sbjct  1554  ...............................................G............  1613

Query  1561  GAGGTCGAAGTGGAAGAACTCGACCAGCGTGCAGGGGAAGGGGTAGTGGAAACACCTCGA  1620
Sbjct  1614  ............................................................  1673

Query  1621  AACTCTATCAAAGTTTCAACACAGATCGGGGACGCGTTAATCGGCAGTTACCTGATCCTA  1680
Sbjct  1674  ........T........T..........................................  1733

Query  1681  TCACCCCAAGCAGTCCTACGCAGCGAAAAATTAGCCTGCATACATGATCTTGCAGAGCAG  1740
Sbjct  1734  ............................................................  1793

Query  1741  GTTAAGTTGGTCACACACTCTGGCCGTAGTGGTAGGTACGCCGTCGACAAATACNACGGA  1800
Sbjct  1794  ..C.................C..T..............................G.....  1853

Query  1801  AGAGTACTAGTCCCTACAGGAGTGGCTATAGACATTCAATCGTTCCAGGCTCTCAGTGAG  1860
Sbjct  1854  ...................................C........................  1913

Query  1861  AGCGCGACCCTTGTGTACAACGAACGCGAGTTCGTTAACAGGAAGCTGTGGCACATAGCA  1920
Sbjct  1914  ..T..........................................T..............  1973

Query  1921  GTATACGGGGCAGCACTCAATACTGATGAAGAAGGATACGAGAAGGTCCCGGTAGAGAGA  1980
Sbjct  1974  ............................................................  2033

Query  1981  GCAGAATCAGATTATGTGTTTGATGTAGACCAAAAAATGTGCCTaaaaaaaGAGCAGGCA  2040
Sbjct  2034  .................A............A.............C...............  2093

Query  2041  TCAGGTTGGGTACTCTGTGGCGAACTAGTCAACCCCCCATTCCACGAATTCGCATATGAA  2100
```

```
Sbjct  2094  .....A......................................................  2153

Query  2101  GGGCTCCGCACGAGACCGTCAGCACCCTACAAGGTTCATACAGTAGGTGTGTACGGAGTG  2160
Sbjct  2154  ........T..T................................................  2213

Query  2161  CCAGGATCAGGCAAATCCGCAATAATCAAGAACACGGTCACCATGTCTGACCTAGTATTG  2220
Sbjct  2214  ............................................................  2273

Query  2221  AGTGGTAAGAAAGAGAACTGCTTAGAAATTATGAACGATGTACTTAAACACAGAGCTCTA  2280
Sbjct  2274  ............................................................  2333

Query  2281  CGTATCACAGCGAAGACCGTAGACTCAGTGTTATTAAACGGCGTGAAACACACGCCTAAC  2340
Sbjct  2334  .....T.............................G........................  2393

Query  2341  ATACTATACATCGACGAAGCGTTCTCATGCCATGCAGGGACTCTGTTGGCCACTATAGCC  2400
Sbjct  2394  ............................................................  2453

Query  2401  ATAGTCAGGCCCAAACAGAAAGTGGTACTGTGCGGAGACCCGAAACAATGCGGATTCTTC  2460
Sbjct  2454  T...........................................................  2513

Query  2461  AATATGATGCAACTGAAAGTTAATTACAATCATGACATCTGCTCAGAAGTCTTCCACAAA  2520
Sbjct  2514  .......................C....................................  2573

Query  2521  AGTATCTCTAGACGGTGCACCCAGGATATCACGGCCATCGTTTCCAAATTACATTACCAG  2580
Sbjct  2574  ............................................T...............  2633

Query  2581  GACCGAATGAGGACCACAAACCCCCGAAAAGGAGACATCATTATAGACACTACCGGCACT  2640
Sbjct  2634  ..................................................C.........  2693

Query  2641  ACCAAACCAGCCAAAACAGATCTGATTCTGACGTGCTTCAGGGGATGGGTGAAACAGTTG  2700
Sbjct  2694  ....................C.......................................  2753

Query  2701  CAGCAAGACTACAGAGGTAACGAAGTAATGACGGCTGCAGCGTCCCAAGGACTGACGAGG  2760
Sbjct  2754  ............................................................  2813

Query  2761  GCCTCCGTATATGCGGTTCGAACTAAAGTCAATGAGAACCCGCTATATGCACAGACCTCC  2820
Sbjct  2814  ............................................................  2873

Query  2821  GAGCACGTGAACGTGTTGTTAACACGCACAGAAAACAAGCTAGTATGGAAGACCTTGTCA  2880
Sbjct  2874  .......................................T....................  2933

Query  2881  ACAGATCCCTGGATTAAAACACTGACTAACCCACCTAGAGGGCACTATACCGCCACCATA  2940
Sbjct  2934  ............................................................  2993

Query  2941  GCAGAATGGGAAGCGGAACACCAGGGTATAATGAAGGCCATACAAGGGTATGCACCGCCC  3000
Sbjct  2994  ..............A.............................................  3053

Query  3001  GTGAACACCTTCATGAACAAAGTAAATGTGTGCTGGGCAAAGACACTTACGCCTGTGCTG  3060
Sbjct  3054  ...........T................................G..C..A.........  3113

Query  3061  GAAACTGCGGGTATCTCCCTGTCAGCAGAAGACTGGTCTGAACTGCTGCCCCCGTTTGCC  3120
Sbjct  3114  ............................................................  3173

Query  3121  CAGGACGTGGCGTACTCACCCGAGGTGGCATTAAACATCATATGCACGAAAATGTATGGG  3180
```

```
Sbjct  3174  ...............................................A............  3233

Query  3181  TTTGACTTAGACACTGGTCTTTTTTCCAGGCCATCAGTGCCAATGACATACACCAAAGAC  3240
Sbjct  3234  ........G.....C.............A.........A.....................  3293

Query  3241  CATTGGGATAACAGAGTTGGAGGGAAAATGTATGGATTCAGCCAACAAGCATACGATCAG  3300
Sbjct  3294  ............................................................  3353

Query  3301  CTGGCAAGACGACATCCGTACCTTCGAGGTAGAGAGAAATCAGGAATGCAGATCGTAGTC  3360
Sbjct  3354  ...................................A........................  3413

Query  3361  ACTGAAATGCGTATCCAGCGCCCAAGATCGGATGCCAACATCATCCCGATCAACCGCAGG  3420
Sbjct  3414  .....G......................................................  3473

Query  3421  CTCCCTCACTCACTCGTAGCCACACACGAGTATAGGCGAGCTGCACGGGCCGAGGAATTC  3480
Sbjct  3474  ............................................G...............  3533

Query  3481  TTCACCACGACACGAGGGTACACTATGCTGCTGGTCTCTGAGTATAACATGAACTTACCA  3540
Sbjct  3534  ..........T.................................................  3593

Query  3541  AACAAGAAGATCACCTGGCTGGCTCCGATAGGGACGCAGGGGGCCCATCACACCGCCAAC  3600
Sbjct  3594  ............................................................  3653

Query  3601  CTAAACTTGGGGATACCACCTCTGCTGGGCAGTTTTGATGCGGTGGTTGTGAACATGCCG  3660
Sbjct  3654  ........................................T...................  3713

Query  3661  ACTCCATTCCGGAACCATCACTACCAGCAATGTGAAGACCACGCGATGAAACTCCAGATG  3720
Sbjct  3714  ................................C...........................  3773

Query  3721  CTGGCAGGCGACGCACTGAGGCACATTAAACCTGGCGGATCATTGTGGGTCAAGGCATAC  3780
Sbjct  3774  .............................G..............................  3833

Query  3781  GGCTACGCAGACCGGCACAGCGAGCACGTGGTCTTGGCATTGGCTAGAAAGTTTAAAAGC  3840
Sbjct  3834  .......................................C....................  3893

Query  3841  TTCAGAGTCACACAACCCTCATGCGTGACTTCCAACACCGAGGTGTTTCTCCACTTCTCA  3900
Sbjct  3894  ...........G........G.....A.................................  3953

Query  3901  ATTTTTGACAATGGCAAACGCGCGATAGCCCTGCATTCAGCTAATAGGAAGGCTAACAGT  3960
Sbjct  3954  ............................................................  4013

Query  3961  ATCTTCCAAAACACCTTCTTACCGGCGGGCAGTGCACCGGCGTACAGAGTCAAACGTGGA  4020
Sbjct  4014  ..........................A....A............................  4073

Query  4021  GACATTTCGAACGCCCCAGAGGATGCAGTGGTCAATGCAGCAAACCAACAGGGAGTGAAG  4080
Sbjct  4074  ............................................................  4133

Query  4081  GGTGCTGGAGTTTGCGGTGCAATTTACCGTAAGTGGCCGGACGCTTTCGGTGATGTCGCT  4140
Sbjct  4134  ............................................................  4193

Query  4141  ACTCCAACCGGAACAGCAGTTTCGAAATCCGTCCAAGATAAATTGGTGATCCACGCTGTC  4200
Sbjct  4194  ............................................................  4253

Query  4201  GGCCCGAATTTCTCAAAATGTTCAGAAGAGGAAGGGGACAGAGACCTAGCATCTGCTTAC  4260
```

```
Sbjct  4254  ...........T................................................  4313

Query  4261  AGAGCTGCAGCAGAAATAGTGATGGATaaaaaaaTTACAACAGTGGCCGTCCCCTTACTC  4320
Sbjct  4314  ..........................A...........................C.....  4373

Query  4321  TCCACCGGCATTTATGCCGGAGGAAAAAACAGAGTAGAACAGTCACTCAACCATCTCTTC  4380
Sbjct  4374  ..............C........G....................................  4433

Query  4381  ACGGCATTCGACAATACTGATGCAGATGTGACCATATATTGCATGGACAAAACATGGGAA  4440
Sbjct  4434  ........T...................................................  4493

Query  4441  AAGAAGATTAAGGAGGCAATCGATCACCGGACTTCGGTTGAGATGGTGCAGGATGACGTG  4500
Sbjct  4494  ............................................................  4553

Query  4501  CAGTTGGAGGAGGAACTGGTACGAGTACACCCTTTGAGTAGTTTAGCAGGTAGGAAGGGT  4560
Sbjct  4554  ......................................C.....................  4613

Query  4561  TACAGTACGGACAGCGGCCGAGTGTTTTCCTACCTGGAAGGTACCAAATTCCATCAGACT  4620
Sbjct  4614  ............................................................  4673

Query  4621  GCGGTGGACATAGCCGAAATGCAAGTGCTGTGGCCCGCCCTCAAAGAGTCTAATGAGCAA  4680
Sbjct  4674  ........T.............................T.....................  4733

Query  4681  ATAGTGGCATACACCTTAGGAGAATCAATGGACCAGATACGTGGCAAGTGCCCGACAGAA  4740
Sbjct  4734  ............................................................  4793

Query  4741  GATACTGACGCCTCCACACCTCCACGGACTGTGCCGTGCCTCTGTCGATACGCCATGACA  4800
Sbjct  4794  ............................................................  4853

Query  4801  CCAGAGAGAGTGTACCGACTTAAATGCACGAACACTACCCAATTTACGGTTTGCTCATCT  4860
Sbjct  4854  ............................................C.....C.........  4913

Query  4861  TTTGAGTTGCCAAAGTATCACATTCAGGGAGTGCAGAGAGTAAAATGTGAAAGAATCATC  4920
Sbjct  4914  ............................................................  4973

Query  4921  ATCTTAGATCCCACTGTTCCACCAACTTACAAACGGCCATGCATCAGACGGTACCCCTCC  4980
Sbjct  4974  ............................................................  5033

Query  4981  ACAATCTCTTGTAACTCCTCTGAGGACTCCAGGAGCTTGTCTACTTTTTCTGTCAGCTCC  5040
Sbjct  5034  ........C......................................C............  5093

Query  5041  GACTCCTCGATTGGTTCTCTGCCGGTCGGAGACACGAGACCCATTCCAGCCCCGAGGACC  5100
Sbjct  5094  .................CG..A...................T...........A......  5153

Query  5101  ATTTTCAGACCCGTCCCTGCCCCGAGAGCACCCGTGCTCAGAACCACACCGCCTCCTAAA  5160
Sbjct  5154  G...............................T..........T................  5213

Query  5161  CCACCGCGCACATTCACCGTGCGTGCAGAAGTGCACCAAGCACCCCCTACACCTGTACCT  5220
Sbjct  5214  ...................................T........................  5273

Query  5221  CCACCCAGACCGAAGAGGGCTGCAAAGTTGGCTCGTGAGATGCACCCCGGGTTCACCTTC  5280
Sbjct  5274  .....T......................................................  5333

Query  5281  GGGGACTTCGGAGAGCACGAGGTTGAGGAGCTTACGGCCTCTCCCTTAACCTTCGGAGAT  5340
```

```
Sbjct  5334  ..A..................A.................G....................  5393

Query  5341  TTTGCTGAAGGAGAGATCCAGGGGATGGGAGTGGAGTTTGAATGACTAGGCAGAGCCGGC  5400
Sbjct  5394  ....................A....C..................................  5453

Query  5401  GGGTACATTTTTTCGTCAGACACGGGTCCAGGCCACCTACAGCAGAGATCCGTTTTACAA  5460
Sbjct  5454  .........................................A.....G............  5513

Query  5461  AATTGCACGGCAGAATGTATCTACGAACCGGCAAAACTAGAAAAAATTCATGCACCAAAG  5520
Sbjct  5514  ............................................................  5573

Query  5521  TTGGATAAAACCAAGGAAGATATCTTAAGGAGCAAGTACCAAATGAAACCGTCTGAAGCA  5580
Sbjct  5574  ............................................................  5633

Query  5581  AACAAAAGCAGGTACCAATCTAGAAAAGTAGAAAATATGAAAGCAGAGATCGTAGGTAGA  5640
Sbjct  5634  ........T.........................................T.........  5693

Query  5641  CTCTTGGACGGACTGGGGGAGTATCTGGGCACCGAGCATCCAGTTGAATGCTACCGAATA  5700
Sbjct  5694  ..............A.............................................  5753

Query  5701  ACGTACCCGGTGCCTATATACTCAACTAGTGACCTCAGAGGTCTGTCTAGTGCCAAAACA  5760
Sbjct  5754  .....T....C...................AT..G.........................  5813

Query  5761  GCTGTTAGAGCTTGCAATGCATTTTTGGAAGCTAATTTTCCATCAGTCACTTCATATAAA  5820
Sbjct  5814  ............................................................  5873

Query  5821  ATTACTGATGAATACGACGCATACCTAGATATGGTAGATGGATCAGAGAGCTGTCTGGAC  5880
Sbjct  5874  ............................................................  5933

Query  5881  AGATCCTCCTTTTCGCCGTCTAGATTGCGTAGCTTTCCAAAAACACACTCATACTTGGAC  5940
Sbjct  5934  .................A...........C..............................  5993

Query  5941  CCACAGATCAACAGTGCGGTACCGTCACCATTCCAAAACACCTTACAAAATGTATTGGCA  6000
Sbjct  5994  ..G.........................................................  6053

Query  6001  GCGGCCACCAAAAGAAACTGTAATGTCACACAGATGAGAGAACTACCAACATATGATTCT  6060
Sbjct  6054  ...............................................G............  6113

Query  6061  GCAGTGCTAAATGTAGAGGCCTTCAGGAAATATGCGTGCAAGCCAGACGTATGGGATGAG  6120
Sbjct  6114  ............................................................  6173

Query  6121  TACAGGGATAATCCGATTTGCATAACCACCGAAAATGTCACCACTTACGTCGCCAAGTTG  6180
Sbjct  6174  ..............A...................................T..T......  6233

Query  6181  AAAGGACCGAAAGCTGCGGCCTTGTTTGCAAAAACACATAACCTGATACCACTACACCAA  6240
Sbjct  6234  ............................................................  6293

Query  6241  GTTCCTATGGACAAATTCACGGTAGATATGAAGAGAGATGTCAAAGTCACGCCCGGAACC  6300
Sbjct  6294  ............................................................  6353

Query  6301  AAGCACACCGAAGAGAGACCAAAGGTACAGGTGATTCAAGCGGCAGAGCCACTAGCCACT  6360
Sbjct  6354  ............................................................  6413

Query  6361  GCCTACCTCTGCGGAATTCACCGTGAATTGGTGCGCCGTCTCAACAACGCGCTTTTCCCA  6420
```

```
Sbjct  6414  .....................................................C.....T  6473

Query  6421  AACATCCACACTTTGTTTGATATGTCCGCAGAGGATTTCGATGCAATCATAGCGGAACAT  6480
Sbjct  6474  ..T...................................T.....................  6533

Query  6481  TTTAAGCACGGTGACCATGTGTTGGAAACGGATATAGCCTCTTTTGACAAAAGTCAAGAT  6540
Sbjct  6534  .................C..........................................  6593

Query  6541  GATTCCATGGCACTCACTGCGTTAATGATCCTTGAGGACCTGGGAGTAGACCAAAACCTA  6600
Sbjct  6594  ............................................................  6653

Query  6601  ATGAATTTGATAGAGGCTGCATTCGGGGAAATCGTGAGTACACACTTGCCCACAGGTACT  6660
Sbjct  6654  ............................................................  6713

Query  6661  AGATTCAAATTTGGAGCTATGATGAAGTCTGGAATGTTTTTGACGCTGTTCGTCAATACA  6720
Sbjct  6714  .......G.....................C...............T..............  6773

Query  6721  ATTCTTAATGTGGTTATTGCGTGCCGAGTGTTGGAGGATCAATTGGCGCAGTCGCCGTGG  6780
Sbjct  6774  ........C..................................................C  6833

Query  6781  CCTGCTTTCATAGGAGATGACAACATAATCCATGGTATAATATCAGACAAATTGATGGCA  6840
Sbjct  6834  G..........................................................G  6893

Query  6841  GATAGATGTGCCACCTGGATGAACATGGAGGTCAAGATACTGGACTCTATAGTTGGAATA  6900
Sbjct  6894  ............................................T..C............  6953

Query  6901  CGGCCACCTTACTTCTGTGGAGGATTTATTGTATGTGACGATGTAACAGGTACAGCCTGC  6960
Sbjct  6954  ...........T................................................  7013

Query  6961  CGCGTCGCAGACCCACTGAAGAGATTGTTCAAGCTAGGTAAGCCATTGCCACTTGACGAT  7020
Sbjct  7014  ........................C...................................  7073

Query  7021  GGCCAAGATGAAGACAGAAGACGTGCATTACATGATGAAGTGAAAACCTGGTCGCGCGTA  7080
Sbjct  7074  .............................G..............................  7133

Query  7081  GGGCTGCGACACAGAGTGTGTGAAGCCATCGAAGACCGTTATGCCGTCCACTCATCAGAA  7140
Sbjct  7134  ..................................................T.........  7193

Query  7141  CTAGTTTTATTGGCACTGACTACTCTGTCTAAGAACTTGAAGTCCTTCAGAAACATAAGA  7200
Sbjct  7194  ............................................................  7253

Query  7201  GGGAAACCAATACATCTCTACGGTGGTCCTAAATAG  7236
Sbjct  7254  ....................................  7289
```

>Barmah Forest virus isolate DC57911, complete genome
Sequence ID: MN689024.1 Length: 11495
Range 1: 52 to 7287

Score:12650 bits(6850), Expect:0.0,
Identities:7107/7236(98%),  Gaps:0/7236(0%), Strand: Plus/Plus

```
Query  1     ATGGCGAAACCAGTTGTGAAGATCGACGTGGAACCTGAAAGCCATTTCGCTAAGCAGGTC  60
Sbjct  52    ...............................................T............  111

Query  61    CAGAGTTGCTTCCCGCAGTTTGAGATCGAAGCAGTGCAGACCACACCAAACGATCATGCA  120
Sbjct  112   .........................................G..................  171

Query  121   CACGCGAGGGCGTTTTCGCACCTTGCTACGAAGCTCATAGAAATGGAGACAGCAAAAGAT  180
Sbjct  172   ............................................................  231

Query  181   CAGATCATCCTCGATATCGGAAGTGCACCCGCGAGGAGACTGTATTCAGAACACAAGTAC  240
Sbjct  232   ............................................................  291

Query  241   CACTGTGTTTGCCCAATGAAGTGCACGGAAGATCCAGAGAGAATGCTAGGATATGCACGT  300
Sbjct  292   ............................................................  351

Query  301   AAGTTGATCGCAGGCTCTGCGAAAGGGAAGGCAGAAAAGTTACGCGATCTCAGGGATGTC  360
Sbjct  352   ............................................................  411

Query  361   TTGGCTACGCCAGACATCGAGACGCAGTCGCTATGTCTCCACACAGACGCATCCTGCAGA  420
Sbjct  412   ............................................................  471

Query  421   TACCGCGGTGATGTTGCCGTGTATCAAGACGTGTATGCCATTGACGCACCTACCACGCTG  480
Sbjct  472   ..................................................C.........  531

Query  481   TACCACCAAGCGTTAAAGGGCGTCAGGACCGCATATTGGATAGGCTTTGATACAACGCCG  540
Sbjct  532   ...........................................................A  591

Query  541   TTCATGTACGATGCACTAGCAGGAGCTTACCCGCTCTACTCCACAAACTGGGCTGATGAG  600
Sbjct  592   .........A...........................................C......  651

Query  601   CAAGTGCTCGAGTCCAGAAACATTGGGCTATGTTCAGACAAAGTTTCTGAAGGGGGAAAG  660
Sbjct  652   ............................................................  711

Query  661   AAAGGGAGATCAATCCTCAGGAAGAAGTTCTTGAAGCAGTCAGACAGAGTCATGTTCTCT  720
Sbjct  712   ............................................................  771

Query  721   GTCGGCTCGACGTTGTATACGGAAAGCCGTAAATTACTGCAAAGTTGGCACCTGCCATCC  780
Sbjct  772   ............................................C...............  831

Query  781   ACATTCCATCTCAAAGGCAAATCTTCGTTCACGTGCCGCTGCGACACTATCGTCAGCTGC  840
Sbjct  832   ................................A..............C............  891

Query  841   GAAGGGTATGTTCTGAAGAAAATTACAATGTGTCCTGGAGTGACAGGCAAACCGATAGGA  900
Sbjct  892   ................................C..C............G...........  951

Query  901   TATGCCGTCACCCATCACAAAGAAGGATTCGTAGTCGGAAAAGTCACAGATACCATTCGC  960
Sbjct  952   ....................G.............................C.........  1011

Query  961   GGCGAGAGAGTCTCCTTCGCCGTGTGTACTTATGTACCAACAACACTCTGCGACCAGATG  1020
Sbjct  1012  ............................................................  1071

Query  1021  ACCGGGATCCTAGCAACAGAAGTAACAGCCGATGATGCCCAGAAACTGCTGGTGGGTTTG  1080
Sbjct  1072  ........T...................................................  1131
```

```
Query  1081  AACCAGAGAATAGTAGTTAATGGTAGGACCCAGAGAAATACCAATACTATGAAGAACTAC  1140
Sbjct  1132  ............................................C...............  1191

Query  1141  CTGCTACCACTGGTTGCACAAGCGCTAGCAAAATGGGCGAAGGAAGCAAAACAGGATATG  1200
Sbjct  1192  ......................................A.....................  1251

Query  1201  GAAGATGAAAGACCCCTGAACGAACGCCAACGAACGCTAACGTGCCTCTGCTGCTGGGCA  1260
Sbjct  1252  ............................................................  1311

Query  1261  TTTAAGCGAAACAAACGCCACGCCATTTACAAGAGACCAGACACACAGAGTATAGTCAAG  1320
Sbjct  1312  ............................................................  1371

Query  1321  GTCCCTTGCGAATTCACAAGCTTTCCTTTGGTCAGCCTGTGGTCCGCTGGGATGTCTATA  1380
Sbjct  1372  ............................................................  1431

Query  1381  TCTCTTAGGCAGAAGTTGAAGATGATGCTGCAGGCGAGGCAGCCCACACAAATAGCAGCA  1440
Sbjct  1432  ............................................................  1491

Query  1441  GTGACTGAGGAACTCATACAAGAAGCAGCTGCAGTAGAGCAAGAGGCCGTGGATACGGCC  1500
Sbjct  1492  ..................................C.........................  1551

Query  1501  AATGCCGAGCTGGACCACGCCGCATGGCCCTCCATTGTGGATACGACAGAGCGCCATGTT  1560
Sbjct  1552  ...............................................G............  1611

Query  1561  GAGGTCGAAGTGGAAGAACTCGACCAGCGTGCAGGGGAAGGGGTAGTGGAAACACCTCGA  1620
Sbjct  1612  ............................................................  1671

Query  1621  AACTCTATCAAAGTTTCAACACAGATCGGGGACGCGTTAATCGGCAGTTACCTGATCCTA  1680
Sbjct  1672  ........T........T..........................................  1731

Query  1681  TCACCCCAAGCAGTCCTACGCAGCGAAAAATTAGCCTGCATACATGATCTTGCAGAGCAG  1740
Sbjct  1732  ............................................................  1791

Query  1741  GTTAAGTTGGTCACACACTCTGGCCGTAGTGGTAGGTACGCCGTCGACAAATACNACGGA  1800
Sbjct  1792  ..C.................C..T..............................G.....  1851

Query  1801  AGAGTACTAGTCCCTACAGGAGTGGCTATAGACATTCAATCGTTCCAGGCTCTCAGTGAG  1860
Sbjct  1852  ...................................C........................  1911

Query  1861  AGCGCGACCCTTGTGTACAACGAACGCGAGTTCGTTAACAGGAAGCTGTGGCACATAGCA  1920
Sbjct  1912  ..T..........................................T..............  1971

Query  1921  GTATACGGGGCAGCACTCAATACTGATGAAGAAGGATACGAGAAGGTCCCGGTAGAGAGA  1980
Sbjct  1972  ............................................................  2031

Query  1981  GCAGAATCAGATTATGTGTTTGATGTAGACCAAAAAATGTGCCTaaaaaaaGAGCAGGCA  2040
Sbjct  2032  .................A............A.............C...............  2091

Query  2041  TCAGGTTGGGTACTCTGTGGCGAACTAGTCAACCCCCCATTCCACGAATTCGCATATGAA  2100
Sbjct  2092  .....A......................................................  2151

Query  2101  GGGCTCCGCACGAGACCGTCAGCACCCTACAAGGTTCATACAGTAGGTGTGTACGGAGTG  2160
Sbjct  2152  ........T..T................................................  2211
```

```
Query  2161  CCAGGATCAGGCAAATCCGCAATAATCAAGAACACGGTCACCATGTCTGACCTAGTATTG  2220
Sbjct  2212  ............................................................  2271

Query  2221  AGTGGTAAGAAAGAGAACTGCTTAGAAATTATGAACGATGTACTTAAACACAGAGCTCTA  2280
Sbjct  2272  ............................................................  2331

Query  2281  CGTATCACAGCGAAGACCGTAGACTCAGTGTTATTAAACGGCGTGAAACACACGCCTAAC  2340
Sbjct  2332  .....T.............................G........................  2391

Query  2341  ATACTATACATCGACGAAGCGTTCTCATGCCATGCAGGGACTCTGTTGGCCACTATAGCC  2400
Sbjct  2392  ............................................................  2451

Query  2401  ATAGTCAGGCCCAAACAGAAAGTGGTACTGTGCGGAGACCCGAAACAATGCGGATTCTTC  2460
Sbjct  2452  T...........................................................  2511

Query  2461  AATATGATGCAACTGAAAGTTAATTACAATCATGACATCTGCTCAGAAGTCTTCCACAAA  2520
Sbjct  2512  .......................C....................................  2571

Query  2521  AGTATCTCTAGACGGTGCACCCAGGATATCACGGCCATCGTTTCCAAATTACATTACCAG  2580
Sbjct  2572  ............................................T...............  2631

Query  2581  GACCGAATGAGGACCACAAACCCCCGAAAAGGAGACATCATTATAGACACTACCGGCACT  2640
Sbjct  2632  ..................................................C.........  2691

Query  2641  ACCAAACCAGCCAAAACAGATCTGATTCTGACGTGCTTCAGGGGATGGGTGAAACAGTTG  2700
Sbjct  2692  ....................C.......................................  2751

Query  2701  CAGCAAGACTACAGAGGTAACGAAGTAATGACGGCTGCAGCGTCCCAAGGACTGACGAGG  2760
Sbjct  2752  ............................................................  2811

Query  2761  GCCTCCGTATATGCGGTTCGAACTAAAGTCAATGAGAACCCGCTATATGCACAGACCTCC  2820
Sbjct  2812  ............................................................  2871

Query  2821  GAGCACGTGAACGTGTTGTTAACACGCACAGAAAACAAGCTAGTATGGAAGACCTTGTCA  2880
Sbjct  2872  .......................................T....................  2931

Query  2881  ACAGATCCCTGGATTAAAACACTGACTAACCCACCTAGAGGGCACTATACCGCCACCATA  2940
Sbjct  2932  ............................................................  2991

Query  2941  GCAGAATGGGAAGCGGAACACCAGGGTATAATGAAGGCCATACAAGGGTATGCACCGCCC  3000
Sbjct  2992  ..............A.............................................  3051

Query  3001  GTGAACACCTTCATGAACAAAGTAAATGTGTGCTGGGCAAAGACACTTACGCCTGTGCTG  3060
Sbjct  3052  ...........T................................G..C..A.........  3111

Query  3061  GAAACTGCGGGTATCTCCCTGTCAGCAGAAGACTGGTCTGAACTGCTGCCCCCGTTTGCC  3120
Sbjct  3112  ............................................................  3171

Query  3121  CAGGACGTGGCGTACTCACCCGAGGTGGCATTAAACATCATATGCACGAAAATGTATGGG  3180
Sbjct  3172  ...............................................A............  3231

Query  3181  TTTGACTTAGACACTGGTCTTTTTTCCAGGCCATCAGTGCCAATGACATACACCAAAGAC  3240
Sbjct  3232  ........G.....C.............A.........A.....................  3291
```

```
Query  3241  CATTGGGATAACAGAGTTGGAGGGAAAATGTATGGATTCAGCCAACAAGCATACGATCAG  3300
Sbjct  3292  ............................................................  3351

Query  3301  CTGGCAAGACGACATCCGTACCTTCGAGGTAGAGAGAAATCAGGAATGCAGATCGTAGTC  3360
Sbjct  3352  ...................................A........................  3411

Query  3361  ACTGAAATGCGTATCCAGCGCCCAAGATCGGATGCCAACATCATCCCGATCAACCGCAGG  3420
Sbjct  3412  .....G......................................................  3471

Query  3421  CTCCCTCACTCACTCGTAGCCACACACGAGTATAGGCGAGCTGCACGGGCCGAGGAATTC  3480
Sbjct  3472  ............................................G...............  3531

Query  3481  TTCACCACGACACGAGGGTACACTATGCTGCTGGTCTCTGAGTATAACATGAACTTACCA  3540
Sbjct  3532  ..........T.................................................  3591

Query  3541  AACAAGAAGATCACCTGGCTGGCTCCGATAGGGACGCAGGGGGCCCATCACACCGCCAAC  3600
Sbjct  3592  ............................................................  3651

Query  3601  CTAAACTTGGGGATACCACCTCTGCTGGGCAGTTTTGATGCGGTGGTTGTGAACATGCCG  3660
Sbjct  3652  ........................................T...................  3711

Query  3661  ACTCCATTCCGGAACCATCACTACCAGCAATGTGAAGACCACGCGATGAAACTCCAGATG  3720
Sbjct  3712  ................................C...........................  3771

Query  3721  CTGGCAGGCGACGCACTGAGGCACATTAAACCTGGCGGATCATTGTGGGTCAAGGCATAC  3780
Sbjct  3772  .............................G..............................  3831

Query  3781  GGCTACGCAGACCGGCACAGCGAGCACGTGGTCTTGGCATTGGCTAGAAAGTTTAAAAGC  3840
Sbjct  3832  .......................................C....................  3891

Query  3841  TTCAGAGTCACACAACCCTCATGCGTGACTTCCAACACCGAGGTGTTTCTCCACTTCTCA  3900
Sbjct  3892  ...........G........G.....A.................................  3951

Query  3901  ATTTTTGACAATGGCAAACGCGCGATAGCCCTGCATTCAGCTAATAGGAAGGCTAACAGT  3960
Sbjct  3952  ............................................................  4011

Query  3961  ATCTTCCAAAACACCTTCTTACCGGCGGGCAGTGCACCGGCGTACAGAGTCAAACGTGGA  4020
Sbjct  4012  ..........................A....A............................  4071

Query  4021  GACATTTCGAACGCCCCAGAGGATGCAGTGGTCAATGCAGCAAACCAACAGGGAGTGAAG  4080
Sbjct  4072  ............................................................  4131

Query  4081  GGTGCTGGAGTTTGCGGTGCAATTTACCGTAAGTGGCCGGACGCTTTCGGTGATGTCGCT  4140
Sbjct  4132  ............................................................  4191

Query  4141  ACTCCAACCGGAACAGCAGTTTCGAAATCCGTCCAAGATAAATTGGTGATCCACGCTGTC  4200
Sbjct  4192  ............................................................  4251

Query  4201  GGCCCGAATTTCTCAAAATGTTCAGAAGAGGAAGGGGACAGAGACCTAGCATCTGCTTAC  4260
Sbjct  4252  ...........T................................................  4311

Query  4261  AGAGCTGCAGCAGAAATAGTGATGGATaaaaaaaTTACAACAGTGGCCGTCCCCTTACTC  4320
Sbjct  4312  ..........................A...........................C.....  4371
```

```
Query  4321  TCCACCGGCATTTATGCCGGAGGAAAAAACAGAGTAGAACAGTCACTCAACCATCTCTTC  4380
Sbjct  4372  ..............C........G....................................  4431

Query  4381  ACGGCATTCGACAATACTGATGCAGATGTGACCATATATTGCATGGACAAAACATGGGAA  4440
Sbjct  4432  ........T...................................................  4491

Query  4441  AAGAAGATTAAGGAGGCAATCGATCACCGGACTTCGGTTGAGATGGTGCAGGATGACGTG  4500
Sbjct  4492  ............................................................  4551

Query  4501  CAGTTGGAGGAGGAACTGGTACGAGTACACCCTTTGAGTAGTTTAGCAGGTAGGAAGGGT  4560
Sbjct  4552  ......................................C.....................  4611

Query  4561  TACAGTACGGACAGCGGCCGAGTGTTTTCCTACCTGGAAGGTACCAAATTCCATCAGACT  4620
Sbjct  4612  ............................................................  4671

Query  4621  GCGGTGGACATAGCCGAAATGCAAGTGCTGTGGCCCGCCCTCAAAGAGTCTAATGAGCAA  4680
Sbjct  4672  ........T.............................T.....................  4731

Query  4681  ATAGTGGCATACACCTTAGGAGAATCAATGGACCAGATACGTGGCAAGTGCCCGACAGAA  4740
Sbjct  4732  ............................................................  4791

Query  4741  GATACTGACGCCTCCACACCTCCACGGACTGTGCCGTGCCTCTGTCGATACGCCATGACA  4800
Sbjct  4792  ............................................................  4851

Query  4801  CCAGAGAGAGTGTACCGACTTAAATGCACGAACACTACCCAATTTACGGTTTGCTCATCT  4860
Sbjct  4852  ............................................C.....C.........  4911

Query  4861  TTTGAGTTGCCAAAGTATCACATTCAGGGAGTGCAGAGAGTAAAATGTGAAAGAATCATC  4920
Sbjct  4912  ............................................................  4971

Query  4921  ATCTTAGATCCCACTGTTCCACCAACTTACAAACGGCCATGCATCAGACGGTACCCCTCC  4980
Sbjct  4972  ............................................................  5031

Query  4981  ACAATCTCTTGTAACTCCTCTGAGGACTCCAGGAGCTTGTCTACTTTTTCTGTCAGCTCC  5040
Sbjct  5032  ........C......................................C............  5091

Query  5041  GACTCCTCGATTGGTTCTCTGCCGGTCGGAGACACGAGACCCATTCCAGCCCCGAGGACC  5100
Sbjct  5092  .................CG..A.............A.....T...........A......  5151

Query  5101  ATTTTCAGACCCGTCCCTGCCCCGAGAGCACCCGTGCTCAGAACCACACCGCCTCCTAAA  5160
Sbjct  5152  G...............................T..........T................  5211

Query  5161  CCACCGCGCACATTCACCGTGCGTGCAGAAGTGCACCAAGCACCCCCTACACCTGTACCT  5220
Sbjct  5212  ...................................T........................  5271

Query  5221  CCACCCAGACCGAAGAGGGCTGCAAAGTTGGCTCGTGAGATGCACCCCGGGTTCACCTTC  5280
Sbjct  5272  .....T......................................................  5331

Query  5281  GGGGACTTCGGAGAGCACGAGGTTGAGGAGCTTACGGCCTCTCCCTTAACCTTCGGAGAT  5340
Sbjct  5332  ..A..................A.................G....................  5391

Query  5341  TTTGCTGAAGGAGAGATCCAGGGGATGGGAGTGGAGTTTGAATGACTAGGCAGAGCCGGC  5400
Sbjct  5392  ....................A....C..................................  5451
```

```
Query  5401  GGGTACATTTTTTCGTCAGACACGGGTCCAGGCCACCTACAGCAGAGATCCGTTTTACAA  5460
Sbjct  5452  .........................................A.....G............  5511

Query  5461  AATTGCACGGCAGAATGTATCTACGAACCGGCAAAACTAGAAAAAATTCATGCACCAAAG  5520
Sbjct  5512  ............................................................  5571

Query  5521  TTGGATAAAACCAAGGAAGATATCTTAAGGAGCAAGTACCAAATGAAACCGTCTGAAGCA  5580
Sbjct  5572  ............................................................  5631

Query  5581  AACAAAAGCAGGTACCAATCTAGAAAAGTAGAAAATATGAAAGCAGAGATCGTAGGTAGA  5640
Sbjct  5632  ........T.........................................T.........  5691

Query  5641  CTCTTGGACGGACTGGGGGAGTATCTGGGCACCGAGCATCCAGTTGAATGCTACCGAATA  5700
Sbjct  5692  ..............A.............................................  5751

Query  5701  ACGTACCCGGTGCCTATATACTCAACTAGTGACCTCAGAGGTCTGTCTAGTGCCAAAACA  5760
Sbjct  5752  .....T....C...................AT..G.........................  5811

Query  5761  GCTGTTAGAGCTTGCAATGCATTTTTGGAAGCTAATTTTCCATCAGTCACTTCATATAAA  5820
Sbjct  5812  ............................................................  5871

Query  5821  ATTACTGATGAATACGACGCATACCTAGATATGGTAGATGGATCAGAGAGCTGTCTGGAC  5880
Sbjct  5872  ............................................................  5931

Query  5881  AGATCCTCCTTTTCGCCGTCTAGATTGCGTAGCTTTCCAAAAACACACTCATACTTGGAC  5940
Sbjct  5932  .................A...........C..............................  5991

Query  5941  CCACAGATCAACAGTGCGGTACCGTCACCATTCCAAAACACCTTACAAAATGTATTGGCA  6000
Sbjct  5992  ..G.........................................................  6051

Query  6001  GCGGCCACCAAAAGAAACTGTAATGTCACACAGATGAGAGAACTACCAACATATGATTCT  6060
Sbjct  6052  ...............................................G............  6111

Query  6061  GCAGTGCTAAATGTAGAGGCCTTCAGGAAATATGCGTGCAAGCCAGACGTATGGGATGAG  6120
Sbjct  6112  ............................................................  6171

Query  6121  TACAGGGATAATCCGATTTGCATAACCACCGAAAATGTCACCACTTACGTCGCCAAGTTG  6180
Sbjct  6172  ..............A...................................T..T......  6231

Query  6181  AAAGGACCGAAAGCTGCGGCCTTGTTTGCAAAAACACATAACCTGATACCACTACACCAA  6240
Sbjct  6232  ............................................................  6291

Query  6241  GTTCCTATGGACAAATTCACGGTAGATATGAAGAGAGATGTCAAAGTCACGCCCGGAACC  6300
Sbjct  6292  ............................................................  6351

Query  6301  AAGCACACCGAAGAGAGACCAAAGGTACAGGTGATTCAAGCGGCAGAGCCACTAGCCACT  6360
Sbjct  6352  ............................................................  6411

Query  6361  GCCTACCTCTGCGGAATTCACCGTGAATTGGTGCGCCGTCTCAACAACGCGCTTTTCCCA  6420
Sbjct  6412  .....................................................C.....T  6471

Query  6421  AACATCCACACTTTGTTTGATATGTCCGCAGAGGATTTCGATGCAATCATAGCGGAACAT  6480
Sbjct  6472  ..T...................................T.....................  6531
```

```
Query  6481  TTTAAGCACGGTGACCATGTGTTGGAAACGGATATAGCCTCTTTTGACAAAAGTCAAGAT  6540
Sbjct  6532  .................C..........................................  6591

Query  6541  GATTCCATGGCACTCACTGCGTTAATGATCCTTGAGGACCTGGGAGTAGACCAAAACCTA  6600
Sbjct  6592  ............................................................  6651

Query  6601  ATGAATTTGATAGAGGCTGCATTCGGGGAAATCGTGAGTACACACTTGCCCACAGGTACT  6660
Sbjct  6652  ............................................................  6711

Query  6661  AGATTCAAATTTGGAGCTATGATGAAGTCTGGAATGTTTTTGACGCTGTTCGTCAATACA  6720
Sbjct  6712  .......G.....................C...............T..............  6771

Query  6721  ATTCTTAATGTGGTTATTGCGTGCCGAGTGTTGGAGGATCAATTGGCGCAGTCGCCGTGG  6780
Sbjct  6772  ........C..................................................C  6831

Query  6781  CCTGCTTTCATAGGAGATGACAACATAATCCATGGTATAATATCAGACAAATTGATGGCA  6840
Sbjct  6832  G..........................................................G  6891

Query  6841  GATAGATGTGCCACCTGGATGAACATGGAGGTCAAGATACTGGACTCTATAGTTGGAATA  6900
Sbjct  6892  ............................................T..C............  6951

Query  6901  CGGCCACCTTACTTCTGTGGAGGATTTATTGTATGTGACGATGTAACAGGTACAGCCTGC  6960
Sbjct  6952  ...........T................................................  7011

Query  6961  CGCGTCGCAGACCCACTGAAGAGATTGTTCAAGCTAGGTAAGCCATTGCCACTTGACGAT  7020
Sbjct  7012  ........................C...................................  7071

Query  7021  GGCCAAGATGAAGACAGAAGACGTGCATTACATGATGAAGTGAAAACCTGGTCGCGCGTA  7080
Sbjct  7072  .............................G..............................  7131

Query  7081  GGGCTGCGACACAGAGTGTGTGAAGCCATCGAAGACCGTTATGCCGTCCACTCATCAGAA  7140
Sbjct  7132  ..................................................T.........  7191

Query  7141  CTAGTTTTATTGGCACTGACTACTCTGTCTAAGAACTTGAAGTCCTTCAGAAACATAAGA  7200
Sbjct  7192  ............................................................  7251

Query  7201  GGGAAACCAATACATCTCTACGGTGGTCCTAAATAG  7236
Sbjct  7252  ....................................  7287
```

>Barmah Forest virus isolate SW97836, complete genome
Sequence ID: MN689045.1 Length: 11298
Range 1: 56 to 7291

Score:12645 bits(6847), Expect:0.0,
Identities:7106/7236(98%), Gaps:0/7236(0%), Strand: Plus/Plus

```
Query  1     ATGGCGAAACCAGTTGTGAAGATCGACGTGGAACCTGAAAGCCATTTCGCTAAGCAGGTC  60
Sbjct  56    ...............................................T............  115

Query  61    CAGAGTTGCTTCCCGCAGTTTGAGATCGAAGCAGTGCAGACCACACCAAACGATCATGCA  120
Sbjct  116   .........................................G..................  175
```

```
Query  121   CACGCGAGGGCGTTTTCGCACCTTGCTACGAAGCTCATAGAAATGGAGACAGCAAAAGAT  180
Sbjct  176   ............................................................  235

Query  181   CAGATCATCCTCGATATCGGAAGTGCACCCGCGAGGAGACTGTATTCAGAACACAAGTAC  240
Sbjct  236   ............................................................  295

Query  241   CACTGTGTTTGCCCAATGAAGTGCACGGAAGATCCAGAGAGAATGCTAGGATATGCACGT  300
Sbjct  296   ............................................................  355

Query  301   AAGTTGATCGCAGGCTCTGCGAAAGGGAAGGCAGAAAAGTTACGCGATCTCAGGGATGTC  360
Sbjct  356   ............................................................  415

Query  361   TTGGCTACGCCAGACATCGAGACGCAGTCGCTATGTCTCCACACAGACGCATCCTGCAGA  420
Sbjct  416   ............................................................  475

Query  421   TACCGCGGTGATGTTGCCGTGTATCAAGACGTGTATGCCATTGACGCACCTACCACGCTG  480
Sbjct  476   ..................................................C.........  535

Query  481   TACCACCAAGCGTTAAAGGGCGTCAGGACCGCATATTGGATAGGCTTTGATACAACGCCG  540
Sbjct  536   ...........................................................A  595

Query  541   TTCATGTACGATGCACTAGCAGGAGCTTACCCGCTCTACTCCACAAACTGGGCTGATGAG  600
Sbjct  596   .........A...........................................C......  655

Query  601   CAAGTGCTCGAGTCCAGAAACATTGGGCTATGTTCAGACAAAGTTTCTGAAGGGGGAAAG  660
Sbjct  656   ............................................................  715

Query  661   AAAGGGAGATCAATCCTCAGGAAGAAGTTCTTGAAGCAGTCAGACAGAGTCATGTTCTCT  720
Sbjct  716   ............................................................  775

Query  721   GTCGGCTCGACGTTGTATACGGAAAGCCGTAAATTACTGCAAAGTTGGCACCTGCCATCC  780
Sbjct  776   ............................................C...............  835

Query  781   ACATTCCATCTCAAAGGCAAATCTTCGTTCACGTGCCGCTGCGACACTATCGTCAGCTGC  840
Sbjct  836   ................................A..............C............  895

Query  841   GAAGGGTATGTTCTGAAGAAAATTACAATGTGTCCTGGAGTGACAGGCAAACCGATAGGA  900
Sbjct  896   ................................C..C........................  955

Query  901   TATGCCGTCACCCATCACAAAGAAGGATTCGTAGTCGGAAAAGTCACAGATACCATTCGC  960
Sbjct  956   ....................G.............................C.........  1015

Query  961   GGCGAGAGAGTCTCCTTCGCCGTGTGTACTTATGTACCAACAACACTCTGCGACCAGATG  1020
Sbjct  1016  ............................................................  1075

Query  1021  ACCGGGATCCTAGCAACAGAAGTAACAGCCGATGATGCCCAGAAACTGCTGGTGGGTTTG  1080
Sbjct  1076  ........T...................................................  1135

Query  1081  AACCAGAGAATAGTAGTTAATGGTAGGACCCAGAGAAATACCAATACTATGAAGAACTAC  1140
Sbjct  1136  ............................................C...............  1195

Query  1141  CTGCTACCACTGGTTGCACAAGCGCTAGCAAAATGGGCGAAGGAAGCAAAACAGGATATG  1200
Sbjct  1196  ......................................A.....................  1255
```

```
Query  1201  GAAGATGAAAGACCCCTGAACGAACGCCAACGAACGCTAACGTGCCTCTGCTGCTGGGCA  1260
Sbjct  1256  ............................................................  1315

Query  1261  TTTAAGCGAAACAAACGCCACGCCATTTACAAGAGACCAGACACACAGAGTATAGTCAAG  1320
Sbjct  1316  ............................................................  1375

Query  1321  GTCCCTTGCGAATTCACAAGCTTTCCTTTGGTCAGCCTGTGGTCCGCTGGGATGTCTATA  1380
Sbjct  1376  ............................................................  1435

Query  1381  TCTCTTAGGCAGAAGTTGAAGATGATGCTGCAGGCGAGGCAGCCCACACAAATAGCAGCA  1440
Sbjct  1436  ............................................................  1495

Query  1441  GTGACTGAGGAACTCATACAAGAAGCAGCTGCAGTAGAGCAAGAGGCCGTGGATACGGCC  1500
Sbjct  1496  ..................................C.........................  1555

Query  1501  AATGCCGAGCTGGACCACGCCGCATGGCCCTCCATTGTGGATACGACAGAGCGCCATGTT  1560
Sbjct  1556  ...............................................G............  1615

Query  1561  GAGGTCGAAGTGGAAGAACTCGACCAGCGTGCAGGGGAAGGGGTAGTGGAAACACCTCGA  1620
Sbjct  1616  ............................................................  1675

Query  1621  AACTCTATCAAAGTTTCAACACAGATCGGGGACGCGTTAATCGGCAGTTACCTGATCCTA  1680
Sbjct  1676  ........T........T..........................................  1735

Query  1681  TCACCCCAAGCAGTCCTACGCAGCGAAAAATTAGCCTGCATACATGATCTTGCAGAGCAG  1740
Sbjct  1736  ............................................................  1795

Query  1741  GTTAAGTTGGTCACACACTCTGGCCGTAGTGGTAGGTACGCCGTCGACAAATACNACGGA  1800
Sbjct  1796  ..C.................C..T..............................G.....  1855

Query  1801  AGAGTACTAGTCCCTACAGGAGTGGCTATAGACATTCAATCGTTCCAGGCTCTCAGTGAG  1860
Sbjct  1856  ...................................C........................  1915

Query  1861  AGCGCGACCCTTGTGTACAACGAACGCGAGTTCGTTAACAGGAAGCTGTGGCACATAGCA  1920
Sbjct  1916  ..T..........................................T..............  1975

Query  1921  GTATACGGGGCAGCACTCAATACTGATGAAGAAGGATACGAGAAGGTCCCGGTAGAGAGA  1980
Sbjct  1976  ............................................................  2035

Query  1981  GCAGAATCAGATTATGTGTTTGATGTAGACCAAAAAATGTGCCTaaaaaaaGAGCAGGCA  2040
Sbjct  2036  .................A............A.............C...............  2095

Query  2041  TCAGGTTGGGTACTCTGTGGCGAACTAGTCAACCCCCCATTCCACGAATTCGCATATGAA  2100
Sbjct  2096  .....A......................................................  2155

Query  2101  GGGCTCCGCACGAGACCGTCAGCACCCTACAAGGTTCATACAGTAGGTGTGTACGGAGTG  2160
Sbjct  2156  ........T..T................................................  2215

Query  2161  CCAGGATCAGGCAAATCCGCAATAATCAAGAACACGGTCACCATGTCTGACCTAGTATTG  2220
Sbjct  2216  ............................................................  2275

Query  2221  AGTGGTAAGAAAGAGAACTGCTTAGAAATTATGAACGATGTACTTAAACACAGAGCTCTA  2280
Sbjct  2276  ............................................................  2335
```

```
Query  2281  CGTATCACAGCGAAGACCGTAGACTCAGTGTTATTAAACGGCGTGAAACACACGCCTAAC  2340
Sbjct  2336  .....T.............................G........................  2395

Query  2341  ATACTATACATCGACGAAGCGTTCTCATGCCATGCAGGGACTCTGTTGGCCACTATAGCC  2400
Sbjct  2396  ............................................................  2455

Query  2401  ATAGTCAGGCCCAAACAGAAAGTGGTACTGTGCGGAGACCCGAAACAATGCGGATTCTTC  2460
Sbjct  2456  T...........................................................  2515

Query  2461  AATATGATGCAACTGAAAGTTAATTACAATCATGACATCTGCTCAGAAGTCTTCCACAAA  2520
Sbjct  2516  .......................C....................................  2575

Query  2521  AGTATCTCTAGACGGTGCACCCAGGATATCACGGCCATCGTTTCCAAATTACATTACCAG  2580
Sbjct  2576  ............................................T...............  2635

Query  2581  GACCGAATGAGGACCACAAACCCCCGAAAAGGAGACATCATTATAGACACTACCGGCACT  2640
Sbjct  2636  ..................................................C.........  2695

Query  2641  ACCAAACCAGCCAAAACAGATCTGATTCTGACGTGCTTCAGGGGATGGGTGAAACAGTTG  2700
Sbjct  2696  ....................C.......................................  2755

Query  2701  CAGCAAGACTACAGAGGTAACGAAGTAATGACGGCTGCAGCGTCCCAAGGACTGACGAGG  2760
Sbjct  2756  ............................................................  2815

Query  2761  GCCTCCGTATATGCGGTTCGAACTAAAGTCAATGAGAACCCGCTATATGCACAGACCTCC  2820
Sbjct  2816  ...........C................................................  2875

Query  2821  GAGCACGTGAACGTGTTGTTAACACGCACAGAAAACAAGCTAGTATGGAAGACCTTGTCA  2880
Sbjct  2876  .......................................T....................  2935

Query  2881  ACAGATCCCTGGATTAAAACACTGACTAACCCACCTAGAGGGCACTATACCGCCACCATA  2940
Sbjct  2936  ............................................................  2995

Query  2941  GCAGAATGGGAAGCGGAACACCAGGGTATAATGAAGGCCATACAAGGGTATGCACCGCCC  3000
Sbjct  2996  ..............A.............................................  3055

Query  3001  GTGAACACCTTCATGAACAAAGTAAATGTGTGCTGGGCAAAGACACTTACGCCTGTGCTG  3060
Sbjct  3056  ...........T................................G..C..A.........  3115

Query  3061  GAAACTGCGGGTATCTCCCTGTCAGCAGAAGACTGGTCTGAACTGCTGCCCCCGTTTGCC  3120
Sbjct  3116  ............................................................  3175

Query  3121  CAGGACGTGGCGTACTCACCCGAGGTGGCATTAAACATCATATGCACGAAAATGTATGGG  3180
Sbjct  3176  ...............................................A............  3235

Query  3181  TTTGACTTAGACACTGGTCTTTTTTCCAGGCCATCAGTGCCAATGACATACACCAAAGAC  3240
Sbjct  3236  ........G.....C.............A.........A.....................  3295

Query  3241  CATTGGGATAACAGAGTTGGAGGGAAAATGTATGGATTCAGCCAACAAGCATACGATCAG  3300
Sbjct  3296  ............................................................  3355

Query  3301  CTGGCAAGACGACATCCGTACCTTCGAGGTAGAGAGAAATCAGGAATGCAGATCGTAGTC  3360
Sbjct  3356  ...................................A........................  3415
```

```
Query  3361  ACTGAAATGCGTATCCAGCGCCCAAGATCGGATGCCAACATCATCCCGATCAACCGCAGG  3420
Sbjct  3416  .....G......................................................  3475

Query  3421  CTCCCTCACTCACTCGTAGCCACACACGAGTATAGGCGAGCTGCACGGGCCGAGGAATTC  3480
Sbjct  3476  ............................................G...............  3535

Query  3481  TTCACCACGACACGAGGGTACACTATGCTGCTGGTCTCTGAGTATAACATGAACTTACCA  3540
Sbjct  3536  ..........T.................................................  3595

Query  3541  AACAAGAAGATCACCTGGCTGGCTCCGATAGGGACGCAGGGGGCCCATCACACCGCCAAC  3600
Sbjct  3596  ............................................................  3655

Query  3601  CTAAACTTGGGGATACCACCTCTGCTGGGCAGTTTTGATGCGGTGGTTGTGAACATGCCG  3660
Sbjct  3656  ........................................T...................  3715

Query  3661  ACTCCATTCCGGAACCATCACTACCAGCAATGTGAAGACCACGCGATGAAACTCCAGATG  3720
Sbjct  3716  ................................C...........................  3775

Query  3721  CTGGCAGGCGACGCACTGAGGCACATTAAACCTGGCGGATCATTGTGGGTCAAGGCATAC  3780
Sbjct  3776  .............................G..............................  3835

Query  3781  GGCTACGCAGACCGGCACAGCGAGCACGTGGTCTTGGCATTGGCTAGAAAGTTTAAAAGC  3840
Sbjct  3836  .......................................C....................  3895

Query  3841  TTCAGAGTCACACAACCCTCATGCGTGACTTCCAACACCGAGGTGTTTCTCCACTTCTCA  3900
Sbjct  3896  ...........G........G.....A.................................  3955

Query  3901  ATTTTTGACAATGGCAAACGCGCGATAGCCCTGCATTCAGCTAATAGGAAGGCTAACAGT  3960
Sbjct  3956  ............................................................  4015

Query  3961  ATCTTCCAAAACACCTTCTTACCGGCGGGCAGTGCACCGGCGTACAGAGTCAAACGTGGA  4020
Sbjct  4016  ........G.................A....A............................  4075

Query  4021  GACATTTCGAACGCCCCAGAGGATGCAGTGGTCAATGCAGCAAACCAACAGGGAGTGAAG  4080
Sbjct  4076  ............................................................  4135

Query  4081  GGTGCTGGAGTTTGCGGTGCAATTTACCGTAAGTGGCCGGACGCTTTCGGTGATGTCGCT  4140
Sbjct  4136  ............................................................  4195

Query  4141  ACTCCAACCGGAACAGCAGTTTCGAAATCCGTCCAAGATAAATTGGTGATCCACGCTGTC  4200
Sbjct  4196  ............................................................  4255

Query  4201  GGCCCGAATTTCTCAAAATGTTCAGAAGAGGAAGGGGACAGAGACCTAGCATCTGCTTAC  4260
Sbjct  4256  ...........T..........................T.....................  4315

Query  4261  AGAGCTGCAGCAGAAATAGTGATGGATaaaaaaaTTACAACAGTGGCCGTCCCCTTACTC  4320
Sbjct  4316  ..........................A...........................C.....  4375

Query  4321  TCCACCGGCATTTATGCCGGAGGAAAAAACAGAGTAGAACAGTCACTCAACCATCTCTTC  4380
Sbjct  4376  ..............C........G....................................  4435

Query  4381  ACGGCATTCGACAATACTGATGCAGATGTGACCATATATTGCATGGACAAAACATGGGAA  4440
Sbjct  4436  ........T...................................................  4495
```

```
Query  4441  AAGAAGATTAAGGAGGCAATCGATCACCGGACTTCGGTTGAGATGGTGCAGGATGACGTG  4500
Sbjct  4496  ............................................................  4555

Query  4501  CAGTTGGAGGAGGAACTGGTACGAGTACACCCTTTGAGTAGTTTAGCAGGTAGGAAGGGT  4560
Sbjct  4556  ......................................C.....................  4615

Query  4561  TACAGTACGGACAGCGGCCGAGTGTTTTCCTACCTGGAAGGTACCAAATTCCATCAGACT  4620
Sbjct  4616  ............................................................  4675

Query  4621  GCGGTGGACATAGCCGAAATGCAAGTGCTGTGGCCCGCCCTCAAAGAGTCTAATGAGCAA  4680
Sbjct  4676  ........T.............................T.....................  4735

Query  4681  ATAGTGGCATACACCTTAGGAGAATCAATGGACCAGATACGTGGCAAGTGCCCGACAGAA  4740
Sbjct  4736  ............................................................  4795

Query  4741  GATACTGACGCCTCCACACCTCCACGGACTGTGCCGTGCCTCTGTCGATACGCCATGACA  4800
Sbjct  4796  ............................................................  4855

Query  4801  CCAGAGAGAGTGTACCGACTTAAATGCACGAACACTACCCAATTTACGGTTTGCTCATCT  4860
Sbjct  4856  ............................................C.....C.........  4915

Query  4861  TTTGAGTTGCCAAAGTATCACATTCAGGGAGTGCAGAGAGTAAAATGTGAAAGAATCATC  4920
Sbjct  4916  ............................................................  4975

Query  4921  ATCTTAGATCCCACTGTTCCACCAACTTACAAACGGCCATGCATCAGACGGTACCCCTCC  4980
Sbjct  4976  ............................................................  5035

Query  4981  ACAATCTCTTGTAACTCCTCTGAGGACTCCAGGAGCTTGTCTACTTTTTCTGTCAGCTCC  5040
Sbjct  5036  ........C......................................C............  5095

Query  5041  GACTCCTCGATTGGTTCTCTGCCGGTCGGAGACACGAGACCCATTCCAGCCCCGAGGACC  5100
Sbjct  5096  .................CG..A...................T...........A......  5155

Query  5101  ATTTTCAGACCCGTCCCTGCCCCGAGAGCACCCGTGCTCAGAACCACACCGCCTCCTAAA  5160
Sbjct  5156  G...............................T..........T................  5215

Query  5161  CCACCGCGCACATTCACCGTGCGTGCAGAAGTGCACCAAGCACCCCCTACACCTGTACCT  5220
Sbjct  5216  ...................................T........................  5275

Query  5221  CCACCCAGACCGAAGAGGGCTGCAAAGTTGGCTCGTGAGATGCACCCCGGGTTCACCTTC  5280
Sbjct  5276  .....T......................................................  5335

Query  5281  GGGGACTTCGGAGAGCACGAGGTTGAGGAGCTTACGGCCTCTCCCTTAACCTTCGGAGAT  5340
Sbjct  5336  ..A..................A.................G....................  5395

Query  5341  TTTGCTGAAGGAGAGATCCAGGGGATGGGAGTGGAGTTTGAATGACTAGGCAGAGCCGGC  5400
Sbjct  5396  ....................A....C..................................  5455

Query  5401  GGGTACATTTTTTCGTCAGACACGGGTCCAGGCCACCTACAGCAGAGATCCGTTTTACAA  5460
Sbjct  5456  .........................................A.....G............  5515

Query  5461  AATTGCACGGCAGAATGTATCTACGAACCGGCAAAACTAGAAAAAATTCATGCACCAAAG  5520
Sbjct  5516  ............................................................  5575
```

```
Query  5521  TTGGATAAAACCAAGGAAGATATCTTAAGGAGCAAGTACCAAATGAAACCGTCTGAAGCA  5580
Sbjct  5576  ............................................................  5635

Query  5581  AACAAAAGCAGGTACCAATCTAGAAAAGTAGAAAATATGAAAGCAGAGATCGTAGGTAGA  5640
Sbjct  5636  ........T.........................................T.........  5695

Query  5641  CTCTTGGACGGACTGGGGGAGTATCTGGGCACCGAGCATCCAGTTGAATGCTACCGAATA  5700
Sbjct  5696  ..............A.............................................  5755

Query  5701  ACGTACCCGGTGCCTATATACTCAACTAGTGACCTCAGAGGTCTGTCTAGTGCCAAAACA  5760
Sbjct  5756  .....T....C...................AT..G.........................  5815

Query  5761  GCTGTTAGAGCTTGCAATGCATTTTTGGAAGCTAATTTTCCATCAGTCACTTCATATAAA  5820
Sbjct  5816  ............................................................  5875

Query  5821  ATTACTGATGAATACGACGCATACCTAGATATGGTAGATGGATCAGAGAGCTGTCTGGAC  5880
Sbjct  5876  ............................................................  5935

Query  5881  AGATCCTCCTTTTCGCCGTCTAGATTGCGTAGCTTTCCAAAAACACACTCATACTTGGAC  5940
Sbjct  5936  .................A...........C..............................  5995

Query  5941  CCACAGATCAACAGTGCGGTACCGTCACCATTCCAAAACACCTTACAAAATGTATTGGCA  6000
Sbjct  5996  ..G.........................................................  6055

Query  6001  GCGGCCACCAAAAGAAACTGTAATGTCACACAGATGAGAGAACTACCAACATATGATTCT  6060
Sbjct  6056  ...............................................G............  6115

Query  6061  GCAGTGCTAAATGTAGAGGCCTTCAGGAAATATGCGTGCAAGCCAGACGTATGGGATGAG  6120
Sbjct  6116  ............................................................  6175

Query  6121  TACAGGGATAATCCGATTTGCATAACCACCGAAAATGTCACCACTTACGTCGCCAAGTTG  6180
Sbjct  6176  ..............A...................................T..T......  6235

Query  6181  AAAGGACCGAAAGCTGCGGCCTTGTTTGCAAAAACACATAACCTGATACCACTACACCAA  6240
Sbjct  6236  ............................................................  6295

Query  6241  GTTCCTATGGACAAATTCACGGTAGATATGAAGAGAGATGTCAAAGTCACGCCCGGAACC  6300
Sbjct  6296  ............................................................  6355

Query  6301  AAGCACACCGAAGAGAGACCAAAGGTACAGGTGATTCAAGCGGCAGAGCCACTAGCCACT  6360
Sbjct  6356  ............................................................  6415

Query  6361  GCCTACCTCTGCGGAATTCACCGTGAATTGGTGCGCCGTCTCAACAACGCGCTTTTCCCA  6420
Sbjct  6416  .....................................................C.....T  6475

Query  6421  AACATCCACACTTTGTTTGATATGTCCGCAGAGGATTTCGATGCAATCATAGCGGAACAT  6480
Sbjct  6476  ..T...................................T.....................  6535

Query  6481  TTTAAGCACGGTGACCATGTGTTGGAAACGGATATAGCCTCTTTTGACAAAAGTCAAGAT  6540
Sbjct  6536  .................C..........................................  6595

Query  6541  GATTCCATGGCACTCACTGCGTTAATGATCCTTGAGGACCTGGGAGTAGACCAAAACCTA  6600
Sbjct  6596  ............................................................  6655
```

```
Query  6601  ATGAATTTGATAGAGGCTGCATTCGGGGAAATCGTGAGTACACACTTGCCCACAGGTACT  6660
Sbjct  6656  ............................................................  6715

Query  6661  AGATTCAAATTTGGAGCTATGATGAAGTCTGGAATGTTTTTGACGCTGTTCGTCAATACA  6720
Sbjct  6716  .......G.....................C...............T..............  6775

Query  6721  ATTCTTAATGTGGTTATTGCGTGCCGAGTGTTGGAGGATCAATTGGCGCAGTCGCCGTGG  6780
Sbjct  6776  ........C..................................................C  6835

Query  6781  CCTGCTTTCATAGGAGATGACAACATAATCCATGGTATAATATCAGACAAATTGATGGCA  6840
Sbjct  6836  G..........................................................G  6895

Query  6841  GATAGATGTGCCACCTGGATGAACATGGAGGTCAAGATACTGGACTCTATAGTTGGAATA  6900
Sbjct  6896  ............................................T..C............  6955

Query  6901  CGGCCACCTTACTTCTGTGGAGGATTTATTGTATGTGACGATGTAACAGGTACAGCCTGC  6960
Sbjct  6956  ...........T................................................  7015

Query  6961  CGCGTCGCAGACCCACTGAAGAGATTGTTCAAGCTAGGTAAGCCATTGCCACTTGACGAT  7020
Sbjct  7016  ........................C...................................  7075

Query  7021  GGCCAAGATGAAGACAGAAGACGTGCATTACATGATGAAGTGAAAACCTGGTCGCGCGTA  7080
Sbjct  7076  .............................G..............................  7135

Query  7081  GGGCTGCGACACAGAGTGTGTGAAGCCATCGAAGACCGTTATGCCGTCCACTCATCAGAA  7140
Sbjct  7136  ..................................................T.........  7195

Query  7141  CTAGTTTTATTGGCACTGACTACTCTGTCTAAGAACTTGAAGTCCTTCAGAAACATAAGA  7200
Sbjct  7196  ............................................................  7255

Query  7201  GGGAAACCAATACATCTCTACGGTGGTCCTAAATAG  7236
Sbjct  7256  ....................................  7291
```

>Barmah Forest virus isolate SW77318, complete genome
Sequence ID: MN689040.1 Length: 11492
Range 1: 54 to 7289

Score:12639 bits(6844), Expect:0.0,
Identities:7105/7236(98%),  Gaps:0/7236(0%), Strand: Plus/Plus

```
Query  1     ATGGCGAAACCAGTTGTGAAGATCGACGTGGAACCTGAAAGCCATTTCGCTAAGCAGGTC  60
Sbjct  54    ...............................................T............  113

Query  61    CAGAGTTGCTTCCCGCAGTTTGAGATCGAAGCAGTGCAGACCACACCAAACGATCATGCA  120
Sbjct  114   .........................................G..................  173

Query  121   CACGCGAGGGCGTTTTCGCACCTTGCTACGAAGCTCATAGAAATGGAGACAGCAAAAGAT  180
Sbjct  174   ............................................................  233

Query  181   CAGATCATCCTCGATATCGGAAGTGCACCCGCGAGGAGACTGTATTCAGAACACAAGTAC  240
```

```
Sbjct  234    ............................................................  293

Query  241    CACTGTGTTTGCCCAATGAAGTGCACGGAAGATCCAGAGAGAATGCTAGGATATGCACGT  300
Sbjct  294    ............................................................  353

Query  301    AAGTTGATCGCAGGCTCTGCGAAAGGGAAGGCAGAAAAGTTACGCGATCTCAGGGATGTC  360
Sbjct  354    ............................................................  413

Query  361    TTGGCTACGCCAGACATCGAGACGCAGTCGCTATGTCTCCACACAGACGCATCCTGCAGA  420
Sbjct  414    ............................................................  473

Query  421    TACCGCGGTGATGTTGCCGTGTATCAAGACGTGTATGCCATTGACGCACCTACCACGCTG  480
Sbjct  474    ..................................................C.........  533

Query  481    TACCACCAAGCGTTAAAGGGCGTCAGGACCGCATATTGGATAGGCTTTGATACAACGCCG  540
Sbjct  534    ...........................................................A  593

Query  541    TTCATGTACGATGCACTAGCAGGAGCTTACCCGCTCTACTCCACAAACTGGGCTGATGAG  600
Sbjct  594    .........A...........................................C......  653

Query  601    CAAGTGCTCGAGTCCAGAAACATTGGGCTATGTTCAGACAAAGTTTCTGAAGGGGGAAAG  660
Sbjct  654    ............................................................  713

Query  661    AAAGGGAGATCAATCCTCAGGAAGAAGTTCTTGAAGCAGTCAGACAGAGTCATGTTCTCT  720
Sbjct  714    ............................................................  773

Query  721    GTCGGCTCGACGTTGTATACGGAAAGCCGTAAATTACTGCAAAGTTGGCACCTGCCATCC  780
Sbjct  774    ............................................C...............  833

Query  781    ACATTCCATCTCAAAGGCAAATCTTCGTTCACGTGCCGCTGCGACACTATCGTCAGCTGC  840
Sbjct  834    ................................A..............C............  893

Query  841    GAAGGGTATGTTCTGAAGAAAATTACAATGTGTCCTGGAGTGACAGGCAAACCGATAGGA  900
Sbjct  894    ................................C..C........................  953

Query  901    TATGCCGTCACCCATCACAAAGAAGGATTCGTAGTCGGAAAAGTCACAGATACCATTCGC  960
Sbjct  954    ....................G.............................C.........  1013

Query  961    GGCGAGAGAGTCTCCTTCGCCGTGTGTACTTATGTACCAACAACACTCTGCGACCAGATG  1020
Sbjct  1014   ............................................................  1073

Query  1021   ACCGGGATCCTAGCAACAGAAGTAACAGCCGATGATGCCCAGAAACTGCTGGTGGGTTTG  1080
Sbjct  1074   ........T...................................................  1133

Query  1081   AACCAGAGAATAGTAGTTAATGGTAGGACCCAGAGAAATACCAATACTATGAAGAACTAC  1140
Sbjct  1134   ............................................C...............  1193

Query  1141   CTGCTACCACTGGTTGCACAAGCGCTAGCAAAATGGGCGAAGGAAGCAAAACAGGATATG  1200
Sbjct  1194   ......................................A.....................  1253

Query  1201   GAAGATGAAAGACCCCTGAACGAACGCCAACGAACGCTAACGTGCCTCTGCTGCTGGGCA  1260
Sbjct  1254   ............................................................  1313

Query  1261   TTTAAGCGAAACAAACGCCACGCCATTTACAAGAGACCAGACACACAGAGTATAGTCAAG  1320
```

```
Sbjct  1314  ............................................................  1373

Query  1321  GTCCCTTGCGAATTCACAAGCTTTCCTTTGGTCAGCCTGTGGTCCGCTGGGATGTCTATA  1380
Sbjct  1374  ............................................................  1433

Query  1381  TCTCTTAGGCAGAAGTTGAAGATGATGCTGCAGGCGAGGCAGCCCACACAAATAGCAGCA  1440
Sbjct  1434  ............................................................  1493

Query  1441  GTGACTGAGGAACTCATACAAGAAGCAGCTGCAGTAGAGCAAGAGGCCGTGGATACGGCC  1500
Sbjct  1494  ............T.....................C.........................  1553

Query  1501  AATGCCGAGCTGGACCACGCCGCATGGCCCTCCATTGTGGATACGACAGAGCGCCATGTT  1560
Sbjct  1554  ...............................................G............  1613

Query  1561  GAGGTCGAAGTGGAAGAACTCGACCAGCGTGCAGGGGAAGGGGTAGTGGAAACACCTCGA  1620
Sbjct  1614  ............................................................  1673

Query  1621  AACTCTATCAAAGTTTCAACACAGATCGGGGACGCGTTAATCGGCAGTTACCTGATCCTA  1680
Sbjct  1674  ........T...................................................  1733

Query  1681  TCACCCCAAGCAGTCCTACGCAGCGAAAAATTAGCCTGCATACATGATCTTGCAGAGCAG  1740
Sbjct  1734  ............................................................  1793

Query  1741  GTTAAGTTGGTCACACACTCTGGCCGTAGTGGTAGGTACGCCGTCGACAAATACNACGGA  1800
Sbjct  1794  ..C.................C..T..............................G.....  1853

Query  1801  AGAGTACTAGTCCCTACAGGAGTGGCTATAGACATTCAATCGTTCCAGGCTCTCAGTGAG  1860
Sbjct  1854  ...................................C........................  1913

Query  1861  AGCGCGACCCTTGTGTACAACGAACGCGAGTTCGTTAACAGGAAGCTGTGGCACATAGCA  1920
Sbjct  1914  ..T..........................................T..............  1973

Query  1921  GTATACGGGGCAGCACTCAATACTGATGAAGAAGGATACGAGAAGGTCCCGGTAGAGAGA  1980
Sbjct  1974  ............................................................  2033

Query  1981  GCAGAATCAGATTATGTGTTTGATGTAGACCAAAAAATGTGCCTaaaaaaaGAGCAGGCA  2040
Sbjct  2034  .................A............A.............C...............  2093

Query  2041  TCAGGTTGGGTACTCTGTGGCGAACTAGTCAACCCCCCATTCCACGAATTCGCATATGAA  2100
Sbjct  2094  .....A......................................................  2153

Query  2101  GGGCTCCGCACGAGACCGTCAGCACCCTACAAGGTTCATACAGTAGGTGTGTACGGAGTG  2160
Sbjct  2154  ........T..T................................................  2213

Query  2161  CCAGGATCAGGCAAATCCGCAATAATCAAGAACACGGTCACCATGTCTGACCTAGTATTG  2220
Sbjct  2214  ............................................................  2273

Query  2221  AGTGGTAAGAAAGAGAACTGCTTAGAAATTATGAACGATGTACTTAAACACAGAGCTCTA  2280
Sbjct  2274  ............................................................  2333

Query  2281  CGTATCACAGCGAAGACCGTAGACTCAGTGTTATTAAACGGCGTGAAACACACGCCTAAC  2340
Sbjct  2334  .....T.............................G........................  2393

Query  2341  ATACTATACATCGACGAAGCGTTCTCATGCCATGCAGGGACTCTGTTGGCCACTATAGCC  2400
```

```
Sbjct  2394  ............................................................  2453

Query  2401  ATAGTCAGGCCCAAACAGAAAGTGGTACTGTGCGGAGACCCGAAACAATGCGGATTCTTC  2460
Sbjct  2454  T..........................................................T  2513

Query  2461  AATATGATGCAACTGAAAGTTAATTACAATCATGACATCTGCTCAGAAGTCTTCCACAAA  2520
Sbjct  2514  .......................C....................................  2573

Query  2521  AGTATCTCTAGACGGTGCACCCAGGATATCACGGCCATCGTTTCCAAATTACATTACCAG  2580
Sbjct  2574  ............................................T...............  2633

Query  2581  GACCGAATGAGGACCACAAACCCCGAAAAGGAGACATCATTATAGACACTACCGGCACT  2640
Sbjct  2634  ..................................................C.........  2693

Query  2641  ACCAAACCAGCCAAAACAGATCTGATTCTGACGTGCTTCAGGGGATGGGTGAAACAGTTG  2700
Sbjct  2694  ....................C.......................................  2753

Query  2701  CAGCAAGACTACAGAGGTAACGAAGTAATGACGGCTGCAGCGTCCCAAGGACTGACGAGG  2760
Sbjct  2754  ............................................................  2813

Query  2761  GCCTCCGTATATGCGGTTCGAACTAAAGTCAATGAGAACCCGCTATATGCACAGACCTCC  2820
Sbjct  2814  ............................................................  2873

Query  2821  GAGCACGTGAACGTGTTGTTAACACGCACAGAAAACAAGCTAGTATGGAAGACCTTGTCA  2880
Sbjct  2874  ..................C....................T....................  2933

Query  2881  ACAGATCCCTGGATTAAAACACTGACTAACCCACCTAGAGGGCACTATACCGCCACCATA  2940
Sbjct  2934  ............................................................  2993

Query  2941  GCAGAATGGGAAGCGGAACACCAGGGTATAATGAAGGCCATACAAGGGTATGCACCGCCC  3000
Sbjct  2994  ..............A.......................T.....................  3053

Query  3001  GTGAACACCTTCATGAACAAAGTAAATGTGTGCTGGGCAAAGACACTTACGCCTGTGCTG  3060
Sbjct  3054  ...............................................C..A.........  3113

Query  3061  GAAACTGCGGGTATCTCCCTGTCAGCAGAAGACTGGTCTGAACTGCTGCCCCCGTTTGCC  3120
Sbjct  3114  ............................................................  3173

Query  3121  CAGGACGTGGCGTACTCACCCGAGGTGGCATTAAACATCATATGCACGAAAATGTATGGG  3180
Sbjct  3174  ...............................................A............  3233

Query  3181  TTTGACTTAGACACTGGTCTTTTTTCCAGGCCATCAGTGCCAATGACATACACCAAAGAC  3240
Sbjct  3234  ........G.....C.............A.........A.....................  3293

Query  3241  CATTGGGATAACAGAGTTGGAGGGAAAATGTATGGATTCAGCCAACAAGCATACGATCAG  3300
Sbjct  3294  ............................................................  3353

Query  3301  CTGGCAAGACGACATCCGTACCTTCGAGGTAGAGAGAAATCAGGAATGCAGATCGTAGTC  3360
Sbjct  3354  ...................................A........................  3413

Query  3361  ACTGAAATGCGTATCCAGCGCCCAAGATCGGATGCCAACATCATCCCGATCAACCGCAGG  3420
Sbjct  3414  .....G......................................................  3473

Query  3421  CTCCCTCACTCACTCGTAGCCACACACGAGTATAGGCGAGCTGCACGGGCCGAGGAATTC  3480
```

```
Sbjct  3474  ............................................G...............  3533

Query  3481  TTCACCACGACACGAGGGTACACTATGCTGCTGGTCTCTGAGTATAACATGAACTTACCA  3540
Sbjct  3534  ..........T.................................................  3593

Query  3541  AACAAGAAGATCACCTGGCTGGCTCCGATAGGGACGCAGGGGGCCCATCACACCGCCAAC  3600
Sbjct  3594  ............................................................  3653

Query  3601  CTAAACTTGGGGATACCACCTCTGCTGGGCAGTTTTGATGCGGTGGTTGTGAACATGCCG  3660
Sbjct  3654  ............................................................  3713

Query  3661  ACTCCATTCCGGAACCATCACTACCAGCAATGTGAAGACCACGCGATGAAACTCCAGATG  3720
Sbjct  3714  ................................C...........................  3773

Query  3721  CTGGCAGGCGACGCACTGAGGCACATTAAACCTGGCGGATCATTGTGGGTCAAGGCATAC  3780
Sbjct  3774  ............................................................  3833

Query  3781  GGCTACGCAGACCGGCACAGCGAGCACGTGGTCTTGGCATTGGCTAGAAAGTTTAAAAGC  3840
Sbjct  3834  .......................................C....................  3893

Query  3841  TTCAGAGTCACACAACCCTCATGCGTGACTTCCAACACCGAGGTGTTTCTCCACTTCTCA  3900
Sbjct  3894  ...........G.....A..G.....A.................................  3953

Query  3901  ATTTTTGACAATGGCAAACGCGCGATAGCCCTGCATTCAGCTAATAGGAAGGCTAACAGT  3960
Sbjct  3954  ............................................................  4013

Query  3961  ATCTTCCAAAACACCTTCTTACCGGCGGGCAGTGCACCGGCGTACAGAGTCAAACGTGGA  4020
Sbjct  4014  ..........................A....A............................  4073

Query  4021  GACATTTCGAACGCCCCAGAGGATGCAGTGGTCAATGCAGCAAACCAACAGGGAGTGAAG  4080
Sbjct  4074  ............................................................  4133

Query  4081  GGTGCTGGAGTTTGCGGTGCAATTTACCGTAAGTGGCCGGACGCTTTCGGTGATGTCGCT  4140
Sbjct  4134  ............................................................  4193

Query  4141  ACTCCAACCGGAACAGCAGTTTCGAAATCCGTCCAAGATAAATTGGTGATCCACGCTGTC  4200
Sbjct  4194  ............................T...............................  4253

Query  4201  GGCCCGAATTTCTCAAAATGTTCAGAAGAGGAAGGGGACAGAGACCTAGCATCTGCTTAC  4260
Sbjct  4254  ............................................................  4313

Query  4261  AGAGCTGCAGCAGAAATAGTGATGGATaaaaaaaTTACAACAGTGGCCGTCCCCTTACTC  4320
Sbjct  4314  ..........................A.................................  4373

Query  4321  TCCACCGGCATTTATGCCGGAGGAAAAAACAGAGTAGAACAGTCACTCAACCATCTCTTC  4380
Sbjct  4374  ..............C........G....................................  4433

Query  4381  ACGGCATTCGACAATACTGATGCAGATGTGACCATATATTGCATGGACAAAACATGGGAA  4440
Sbjct  4434  ........T...................................................  4493

Query  4441  AAGAAGATTAAGGAGGCAATCGATCACCGGACTTCGGTTGAGATGGTGCAGGATGACGTG  4500
Sbjct  4494  ............................................................  4553

Query  4501  CAGTTGGAGGAGGAACTGGTACGAGTACACCCTTTGAGTAGTTTAGCAGGTAGGAAGGGT  4560
```

```
Sbjct  4554  ............................................................  4613

Query  4561  TACAGTACGGACAGCGGCCGAGTGTTTTCCTACCTGGAAGGTACCAAATTCCATCAGACT  4620
Sbjct  4614  ............................................................  4673

Query  4621  GCGGTGGACATAGCCGAAATGCAAGTGCTGTGGCCCGCCCTCAAAGAGTCTAATGAGCAA  4680
Sbjct  4674  ........T.............................T.....................  4733

Query  4681  ATAGTGGCATACACCTTAGGAGAATCAATGGACCAGATACGTGGCAAGTGCCCGACAGAA  4740
Sbjct  4734  ............................................................  4793

Query  4741  GATACTGACGCCTCCACACCTCCACGGACTGTGCCGTGCCTCTGTCGATACGCCATGACA  4800
Sbjct  4794  ..C.........................................................  4853

Query  4801  CCAGAGAGAGTGTACCGACTTAAATGCACGAACACTACCCAATTTACGGTTTGCTCATCT  4860
Sbjct  4854  ............................................C.....C.........  4913

Query  4861  TTTGAGTTGCCAAAGTATCACATTCAGGGAGTGCAGAGAGTAAAATGTGAAAGAATCATC  4920
Sbjct  4914  ............................................................  4973

Query  4921  ATCTTAGATCCCACTGTTCCACCAACTTACAAACGGCCATGCATCAGACGGTACCCCTCC  4980
Sbjct  4974  ............................................................  5033

Query  4981  ACAATCTCTTGTAACTCCTCTGAGGACTCCAGGAGCTTGTCTACTTTTTCTGTCAGCTCC  5040
Sbjct  5034  ........C......................................C............  5093

Query  5041  GACTCCTCGATTGGTTCTCTGCCGGTCGGAGACACGAGACCCATTCCAGCCCCGAGGACC  5100
Sbjct  5094  .................CG..A...................T...........A.....T  5153

Query  5101  ATTTTCAGACCCGTCCCTGCCCCGAGAGCACCCGTGCTCAGAACCACACCGCCTCCTAAA  5160
Sbjct  5154  G..........................................T................  5213

Query  5161  CCACCGCGCACATTCACCGTGCGTGCAGAAGTGCACCAAGCACCCCCTACACCTGTACCT  5220
Sbjct  5214  ...................................T........................  5273

Query  5221  CCACCCAGACCGAAGAGGGCTGCAAAGTTGGCTCGTGAGATGCACCCCGGGTTCACCTTC  5280
Sbjct  5274  ............................................................  5333

Query  5281  GGGGACTTCGGAGAGCACGAGGTTGAGGAGCTTACGGCCTCTCCCTTAACCTTCGGAGAT  5340
Sbjct  5334  ..A..................A.................G....................  5393

Query  5341  TTTGCTGAAGGAGAGATCCAGGGGATGGGAGTGGAGTTTGAATGACTAGGCAGAGCCGGC  5400
Sbjct  5394  ......................................C.....................  5453

Query  5401  GGGTACATTTTTTCGTCAGACACGGGTCCAGGCCACCTACAGCAGAGATCCGTTTTACAA  5460
Sbjct  5454  ...................................TT....A..................  5513

Query  5461  AATTGCACGGCAGAATGTATCTACGAACCGGCAAAACTAGAAAAAATTCATGCACCAAAG  5520
Sbjct  5514  ............................................................  5573

Query  5521  TTGGATAAAACCAAGGAAGATATCTTAAGGAGCAAGTACCAAATGAAACCGTCTGAAGCA  5580
Sbjct  5574  ........G...................................................  5633

Query  5581  AACAAAAGCAGGTACCAATCTAGAAAAGTAGAAAATATGAAAGCAGAGATCGTAGGTAGA  5640
```

```
Sbjct  5634  ........T.......................................T.........  5693

Query  5641  CTCTTGGACGGACTGGGGGAGTATCTGGGCACCGAGCATCCAGTTGAATGCTACCGAATA  5700
Sbjct  5694  ..............A.....................C.....................  5753

Query  5701  ACGTACCCGGTGCCTATATACTCAACTAGTGACCTCAGAGGTCTGTCTAGTGCCAAAACA  5760
Sbjct  5754  .....T.......................AT..G.........................  5813

Query  5761  GCTGTTAGAGCTTGCAATGCATTTTTGGAAGCTAATTTTCCATCAGTCACTTCATATAAA  5820
Sbjct  5814  ............................................................  5873

Query  5821  ATTACTGATGAATACGACGCATACCTAGATATGGTAGATGGATCAGAGAGCTGTCTGGAC  5880
Sbjct  5874  ............................................................  5933

Query  5881  AGATCCTCCTTTTCGCCGTCTAGATTGCGTAGCTTTCCAAAAACACACTCATACTTGGAC  5940
Sbjct  5934  .................A...........C..............................  5993

Query  5941  CCACAGATCAACAGTGCGGTACCGTCACCATTCCAAAACACCTTACAAAATGTATTGGCA  6000
Sbjct  5994  ..G.......................G.................................  6053

Query  6001  GCGGCCACCAAAAGAAACTGTAATGTCACACAGATGAGAGAACTACCAACATATGATTCT  6060
Sbjct  6054  ................................................G...........  6113

Query  6061  GCAGTGCTAAATGTAGAGGCCTTCAGGAAATATGCGTGCAAGCCAGACGTATGGGATGAG  6120
Sbjct  6114  ............................................................  6173

Query  6121  TACAGGGATAATCCGATTTGCATAACCACCGAAAATGTCACCACTTACGTCGCCAAGTTG  6180
Sbjct  6174  ..............A..............A.......................T......  6233

Query  6181  AAAGGACCGAAAGCTGCGGCCTTGTTTGCAAAAACACATAACCTGATACCACTACACCAA  6240
Sbjct  6234  ............................................................  6293

Query  6241  GTTCCTATGGACAAATTCACGGTAGATATGAAGAGAGATGTCAAAGTCACGCCCGGAACC  6300
Sbjct  6294  ............................................................  6353

Query  6301  AAGCACACCGAAGAGAGACCAAAGGTACAGGTGATTCAAGCGGCAGAGCCACTAGCCACT  6360
Sbjct  6354  ..................................................T........  6413

Query  6361  GCCTACCTCTGCGGAATTCACCGTGAATTGGTGCGCCGTCTCAACAACGCGCTTTTCCCA  6420
Sbjct  6414  .....................................................C......  6473

Query  6421  AACATCCACACTTTGTTTGATATGTCCGCAGAGGATTTCGATGCAATCATAGCGGAACAT  6480
Sbjct  6474  ..T...............................C..T......................  6533

Query  6481  TTTAAGCACGGTGACCATGTGTTGGAAACGGATATAGCCTCTTTTGACAAAAGTCAAGAT  6540
Sbjct  6534  .................C..........................................  6593

Query  6541  GATTCCATGGCACTCACTGCGTTAATGATCCTTGAGGACCTGGGAGTAGACCAAAACCTA  6600
Sbjct  6594  ............................................................  6653

Query  6601  ATGAATTTGATAGAGGCTGCATTCGGGGAAATCGTGAGTACACACTTGCCCACAGGTACT  6660
Sbjct  6654  ............................................................  6713

Query  6661  AGATTCAAATTTGGAGCTATGATGAAGTCTGGAATGTTTTTGACGCTGTTCGTCAATACA  6720
```

```
Sbjct  6714  .......G.....................C...............T..............  6773

Query  6721  ATTCTTAATGTGGTTATTGCGTGCCGAGTGTTGGAGGATCAATTGGCGCAGTCGCCGTGG  6780
Sbjct  6774  ........C......................................A...........C  6833

Query  6781  CCTGCTTTCATAGGAGATGACAACATAATCCATGGTATAATATCAGACAAATTGATGGCA  6840
Sbjct  6834  G..........................................................G  6893

Query  6841  GATAGATGTGCCACCTGGATGAACATGGAGGTCAAGATACTGGACTCTATAGTTGGAATA  6900
Sbjct  6894  ..............T.............................T..C............  6953

Query  6901  CGGCCACCTTACTTCTGTGGAGGATTTATTGTATGTGACGATGTAACAGGTACAGCCTGC  6960
Sbjct  6954  ...........T................................................  7013

Query  6961  CGCGTCGCAGACCCACTGAAGAGATTGTTCAAGCTAGGTAAGCCATTGCCACTTGACGAT  7020
Sbjct  7014  ..............G.........C...................................  7073

Query  7021  GGCCAAGATGAAGACAGAAGACGTGCATTACATGATGAAGTGAAAACCTGGTCGCGCGTA  7080
Sbjct  7074  ........C...................................................  7133

Query  7081  GGGCTGCGACACAGAGTGTGTGAAGCCATCGAAGACCGTTATGCCGTCCACTCATCAGAA  7140
Sbjct  7134  ..................................................T.........  7193

Query  7141  CTAGTTTTATTGGCACTGACTACTCTGTCTAAGAACTTGAAGTCCTTCAGAAACATAAGA  7200
Sbjct  7194  ............................................................  7253

Query  7201  GGGAAACCAATACATCTCTACGGTGGTCCTAAATAG  7236
Sbjct  7254  ....................................  7289
```

>Barmah Forest virus isolate SW75325, complete genome
Sequence ID: MN689038.1 Length: 11461
Range 1: 53 to 7288

Score:12639 bits(6844), Expect:0.0,
Identities:7105/7236(98%),  Gaps:0/7236(0%), Strand: Plus/Plus

```
Query  1     ATGGCGAAACCAGTTGTGAAGATCGACGTGGAACCTGAAAGCCATTTCGCTAAGCAGGTC  60
Sbjct  53    ...............................................T............  112

Query  61    CAGAGTTGCTTCCCGCAGTTTGAGATCGAAGCAGTGCAGACCACACCAAACGATCATGCA  120
Sbjct  113   .........................................G..................  172

Query  121   CACGCGAGGGCGTTTTCGCACCTTGCTACGAAGCTCATAGAAATGGAGACAGCAAAAGAT  180
Sbjct  173   ............................................................  232

Query  181   CAGATCATCCTCGATATCGGAAGTGCACCCGCGAGGAGACTGTATTCAGAACACAAGTAC  240
Sbjct  233   ............................................................  292

Query  241   CACTGTGTTTGCCCAATGAAGTGCACGGAAGATCCAGAGAGAATGCTAGGATATGCACGT  300
Sbjct  293   ............................................................  352
```

```
Query  301   AAGTTGATCGCAGGCTCTGCGAAAGGGAAGGCAGAAAAGTTACGCGATCTCAGGGATGTC  360
Sbjct  353   ............................................................  412

Query  361   TTGGCTACGCCAGACATCGAGACGCAGTCGCTATGTCTCCACACAGACGCATCCTGCAGA  420
Sbjct  413   ............................................................  472

Query  421   TACCGCGGTGATGTTGCCGTGTATCAAGACGTGTATGCCATTGACGCACCTACCACGCTG  480
Sbjct  473   ..................................................C.........  532

Query  481   TACCACCAAGCGTTAAAGGGCGTCAGGACCGCATATTGGATAGGCTTTGATACAACGCCG  540
Sbjct  533   ...........................................................A  592

Query  541   TTCATGTACGATGCACTAGCAGGAGCTTACCCGCTCTACTCCACAAACTGGGCTGATGAG  600
Sbjct  593   .........A...........................................C......  652

Query  601   CAAGTGCTCGAGTCCAGAAACATTGGGCTATGTTCAGACAAAGTTTCTGAAGGGGGAAAG  660
Sbjct  653   ............................................................  712

Query  661   AAAGGGAGATCAATCCTCAGGAAGAAGTTCTTGAAGCAGTCAGACAGAGTCATGTTCTCT  720
Sbjct  713   ............................................................  772

Query  721   GTCGGCTCGACGTTGTATACGGAAAGCCGTAAATTACTGCAAAGTTGGCACCTGCCATCC  780
Sbjct  773   ............................................C...............  832

Query  781   ACATTCCATCTCAAAGGCAAATCTTCGTTCACGTGCCGCTGCGACACTATCGTCAGCTGC  840
Sbjct  833   ................................A..............C............  892

Query  841   GAAGGGTATGTTCTGAAGAAAATTACAATGTGTCCTGGAGTGACAGGCAAACCGATAGGA  900
Sbjct  893   ................................C..C........................  952

Query  901   TATGCCGTCACCCATCACAAAGAAGGATTCGTAGTCGGAAAAGTCACAGATACCATTCGC  960
Sbjct  953   ....................G.............................C.........  1012

Query  961   GGCGAGAGAGTCTCCTTCGCCGTGTGTACTTATGTACCAACAACACTCTGCGACCAGATG  1020
Sbjct  1013  ............................................................  1072

Query  1021  ACCGGGATCCTAGCAACAGAAGTAACAGCCGATGATGCCCAGAAACTGCTGGTGGGTTTG  1080
Sbjct  1073  ........T...................................................  1132

Query  1081  AACCAGAGAATAGTAGTTAATGGTAGGACCCAGAGAAATACCAATACTATGAAGAACTAC  1140
Sbjct  1133  ............................................C...............  1192

Query  1141  CTGCTACCACTGGTTGCACAAGCGCTAGCAAAATGGGCGAAGGAAGCAAAACAGGATATG  1200
Sbjct  1193  ......................................A.....................  1252

Query  1201  GAAGATGAAAGACCCCTGAACGAACGCCAACGAACGCTAACGTGCCTCTGCTGCTGGGCA  1260
Sbjct  1253  ............................................................  1312

Query  1261  TTTAAGCGAAACAAACGCCACGCCATTTACAAGAGACCAGACACACAGAGTATAGTCAAG  1320
Sbjct  1313  ............................................................  1372

Query  1321  GTCCCTTGCGAATTCACAAGCTTTCCTTTGGTCAGCCTGTGGTCCGCTGGGATGTCTATA  1380
Sbjct  1373  ............................................................  1432
```

```
Query  1381  TCTCTTAGGCAGAAGTTGAAGATGATGCTGCAGGCGAGGCAGCCCACACAAATAGCAGCA  1440
Sbjct  1433  ............................................................  1492

Query  1441  GTGACTGAGGAACTCATACAAGAAGCAGCTGCAGTAGAGCAAGAGGCCGTGGATACGGCC  1500
Sbjct  1493  ............T.....................C.........................  1552

Query  1501  AATGCCGAGCTGGACCACGCCGCATGGCCCTCCATTGTGGATACGACAGAGCGCCATGTT  1560
Sbjct  1553  ...............................................G............  1612

Query  1561  GAGGTCGAAGTGGAAGAACTCGACCAGCGTGCAGGGGAAGGGGTAGTGGAAACACCTCGA  1620
Sbjct  1613  ............................................................  1672

Query  1621  AACTCTATCAAAGTTTCAACACAGATCGGGGACGCGTTAATCGGCAGTTACCTGATCCTA  1680
Sbjct  1673  ........T...................................................  1732

Query  1681  TCACCCCAAGCAGTCCTACGCAGCGAAAAATTAGCCTGCATACATGATCTTGCAGAGCAG  1740
Sbjct  1733  ............................................................  1792

Query  1741  GTTAAGTTGGTCACACACTCTGGCCGTAGTGGTAGGTACGCCGTCGACAAATACNACGGA  1800
Sbjct  1793  ..C.................C..T..............................G.....  1852

Query  1801  AGAGTACTAGTCCCTACAGGAGTGGCTATAGACATTCAATCGTTCCAGGCTCTCAGTGAG  1860
Sbjct  1853  ...................................C........................  1912

Query  1861  AGCGCGACCCTTGTGTACAACGAACGCGAGTTCGTTAACAGGAAGCTGTGGCACATAGCA  1920
Sbjct  1913  ..T..........................................T..............  1972

Query  1921  GTATACGGGGCAGCACTCAATACTGATGAAGAAGGATACGAGAAGGTCCCGGTAGAGAGA  1980
Sbjct  1973  ............................................................  2032

Query  1981  GCAGAATCAGATTATGTGTTTGATGTAGACCAAAAAATGTGCCTaaaaaaaGAGCAGGCA  2040
Sbjct  2033  .................A............A.............C...............  2092

Query  2041  TCAGGTTGGGTACTCTGTGGCGAACTAGTCAACCCCCCATTCCACGAATTCGCATATGAA  2100
Sbjct  2093  .....A......................................................  2152

Query  2101  GGGCTCCGCACGAGACCGTCAGCACCCTACAAGGTTCATACAGTAGGTGTGTACGGAGTG  2160
Sbjct  2153  ........T..T................................................  2212

Query  2161  CCAGGATCAGGCAAATCCGCAATAATCAAGAACACGGTCACCATGTCTGACCTAGTATTG  2220
Sbjct  2213  ............................................................  2272

Query  2221  AGTGGTAAGAAAGAGAACTGCTTAGAAATTATGAACGATGTACTTAAACACAGAGCTCTA  2280
Sbjct  2273  ............................................................  2332

Query  2281  CGTATCACAGCGAAGACCGTAGACTCAGTGTTATTAAACGGCGTGAAACACACGCCTAAC  2340
Sbjct  2333  .....T.............................G........................  2392

Query  2341  ATACTATACATCGACGAAGCGTTCTCATGCCATGCAGGGACTCTGTTGGCCACTATAGCC  2400
Sbjct  2393  ............................................................  2452

Query  2401  ATAGTCAGGCCCAAACAGAAAGTGGTACTGTGCGGAGACCCGAAACAATGCGGATTCTTC  2460
Sbjct  2453  T..........................................................T  2512
```

```
Query  2461  AATATGATGCAACTGAAAGTTAATTACAATCATGACATCTGCTCAGAAGTCTTCCACAAA  2520
Sbjct  2513  .......................C....................................  2572

Query  2521  AGTATCTCTAGACGGTGCACCCAGGATATCACGGCCATCGTTTCCAAATTACATTACCAG  2580
Sbjct  2573  ............................................T...............  2632

Query  2581  GACCGAATGAGGACCACAAACCCCCGAAAAGGAGACATCATTATAGACACTACCGGCACT  2640
Sbjct  2633  ..................................................C.........  2692

Query  2641  ACCAAACCAGCCAAAACAGATCTGATTCTGACGTGCTTCAGGGGATGGGTGAAACAGTTG  2700
Sbjct  2693  ....................C.......................................  2752

Query  2701  CAGCAAGACTACAGAGGTAACGAAGTAATGACGGCTGCAGCGTCCCAAGGACTGACGAGG  2760
Sbjct  2753  ............................................................  2812

Query  2761  GCCTCCGTATATGCGGTTCGAACTAAAGTCAATGAGAACCCGCTATATGCACAGACCTCC  2820
Sbjct  2813  ............................................................  2872

Query  2821  GAGCACGTGAACGTGTTGTTAACACGCACAGAAAACAAGCTAGTATGGAAGACCTTGTCA  2880
Sbjct  2873  ..................C....................T....................  2932

Query  2881  ACAGATCCCTGGATTAAAACACTGACTAACCCACCTAGAGGGCACTATACCGCCACCATA  2940
Sbjct  2933  ............................................................  2992

Query  2941  GCAGAATGGGAAGCGGAACACCAGGGTATAATGAAGGCCATACAAGGGTATGCACCGCCC  3000
Sbjct  2993  ..............A.......................T.....................  3052

Query  3001  GTGAACACCTTCATGAACAAAGTAAATGTGTGCTGGGCAAAGACACTTACGCCTGTGCTG  3060
Sbjct  3053  ...............................................C..A.........  3112

Query  3061  GAAACTGCGGGTATCTCCCTGTCAGCAGAAGACTGGTCTGAACTGCTGCCCCCGTTTGCC  3120
Sbjct  3113  ............................................................  3172

Query  3121  CAGGACGTGGCGTACTCACCCGAGGTGGCATTAAACATCATATGCACGAAAATGTATGGG  3180
Sbjct  3173  ...............................................A............  3232

Query  3181  TTTGACTTAGACACTGGTCTTTTTTCCAGGCCATCAGTGCCAATGACATACACCAAAGAC  3240
Sbjct  3233  ........G.....C.............A.........A.....................  3292

Query  3241  CATTGGGATAACAGAGTTGGAGGGAAAATGTATGGATTCAGCCAACAAGCATACGATCAG  3300
Sbjct  3293  ............................................................  3352

Query  3301  CTGGCAAGACGACATCCGTACCTTCGAGGTAGAGAGAAATCAGGAATGCAGATCGTAGTC  3360
Sbjct  3353  ...................................A........................  3412

Query  3361  ACTGAAATGCGTATCCAGCGCCCAAGATCGGATGCCAACATCATCCCGATCAACCGCAGG  3420
Sbjct  3413  .....G......................................................  3472

Query  3421  CTCCCTCACTCACTCGTAGCCACACACGAGTATAGGCGAGCTGCACGGGCCGAGGAATTC  3480
Sbjct  3473  ............................................G...............  3532

Query  3481  TTCACCACGACACGAGGGTACACTATGCTGCTGGTCTCTGAGTATAACATGAACTTACCA  3540
Sbjct  3533  ..........T.................................................  3592
```

```
Query  3541  AACAAGAAGATCACCTGGCTGGCTCCGATAGGGACGCAGGGGGCCCATCACACCGCCAAC  3600
Sbjct  3593  ............................................................  3652

Query  3601  CTAAACTTGGGGATACCACCTCTGCTGGGCAGTTTTGATGCGGTGGTTGTGAACATGCCG  3660
Sbjct  3653  ............................................................  3712

Query  3661  ACTCCATTCCGGAACCATCACTACCAGCAATGTGAAGACCACGCGATGAAACTCCAGATG  3720
Sbjct  3713  ................................C...........................  3772

Query  3721  CTGGCAGGCGACGCACTGAGGCACATTAAACCTGGCGGATCATTGTGGGTCAAGGCATAC  3780
Sbjct  3773  ............................................................  3832

Query  3781  GGCTACGCAGACCGGCACAGCGAGCACGTGGTCTTGGCATTGGCTAGAAAGTTTAAAAGC  3840
Sbjct  3833  .......................................C....................  3892

Query  3841  TTCAGAGTCACACAACCCTCATGCGTGACTTCCAACACCGAGGTGTTTCTCCACTTCTCA  3900
Sbjct  3893  ...........G.....A..G.....A.................................  3952

Query  3901  ATTTTTGACAATGGCAAACGCGCGATAGCCCTGCATTCAGCTAATAGGAAGGCTAACAGT  3960
Sbjct  3953  ............................................................  4012

Query  3961  ATCTTCCAAAACACCTTCTTACCGGCGGGCAGTGCACCGGCGTACAGAGTCAAACGTGGA  4020
Sbjct  4013  ..........................A....A............................  4072

Query  4021  GACATTTCGAACGCCCCAGAGGATGCAGTGGTCAATGCAGCAAACCAACAGGGAGTGAAG  4080
Sbjct  4073  ............................................................  4132

Query  4081  GGTGCTGGAGTTTGCGGTGCAATTTACCGTAAGTGGCCGGACGCTTTCGGTGATGTCGCT  4140
Sbjct  4133  ............................................................  4192

Query  4141  ACTCCAACCGGAACAGCAGTTTCGAAATCCGTCCAAGATAAATTGGTGATCCACGCTGTC  4200
Sbjct  4193  ............................T...............................  4252

Query  4201  GGCCCGAATTTCTCAAAATGTTCAGAAGAGGAAGGGGACAGAGACCTAGCATCTGCTTAC  4260
Sbjct  4253  ............................................................  4312

Query  4261  AGAGCTGCAGCAGAAATAGTGATGGATaaaaaaaTTACAACAGTGGCCGTCCCCTTACTC  4320
Sbjct  4313  ..........................A.................................  4372

Query  4321  TCCACCGGCATTTATGCCGGAGGAAAAAACAGAGTAGAACAGTCACTCAACCATCTCTTC  4380
Sbjct  4373  ..............C........G....................................  4432

Query  4381  ACGGCATTCGACAATACTGATGCAGATGTGACCATATATTGCATGGACAAAACATGGGAA  4440
Sbjct  4433  ........T...................................................  4492

Query  4441  AAGAAGATTAAGGAGGCAATCGATCACCGGACTTCGGTTGAGATGGTGCAGGATGACGTG  4500
Sbjct  4493  ............................................................  4552

Query  4501  CAGTTGGAGGAGGAACTGGTACGAGTACACCCTTTGAGTAGTTTAGCAGGTAGGAAGGGT  4560
Sbjct  4553  ............................................................  4612

Query  4561  TACAGTACGGACAGCGGCCGAGTGTTTTCCTACCTGGAAGGTACCAAATTCCATCAGACT  4620
Sbjct  4613  ............................................................  4672
```

```
Query  4621  GCGGTGGACATAGCCGAAATGCAAGTGCTGTGGCCCGCCCTCAAAGAGTCTAATGAGCAA  4680
Sbjct  4673  ........T.............................T.....................  4732

Query  4681  ATAGTGGCATACACCTTAGGAGAATCAATGGACCAGATACGTGGCAAGTGCCCGACAGAA  4740
Sbjct  4733  ............................................................  4792

Query  4741  GATACTGACGCCTCCACACCTCCACGGACTGTGCCGTGCCTCTGTCGATACGCCATGACA  4800
Sbjct  4793  ..C.........................................................  4852

Query  4801  CCAGAGAGAGTGTACCGACTTAAATGCACGAACACTACCCAATTTACGGTTTGCTCATCT  4860
Sbjct  4853  ............................................C.....C.........  4912

Query  4861  TTTGAGTTGCCAAAGTATCACATTCAGGGAGTGCAGAGAGTAAAATGTGAAAGAATCATC  4920
Sbjct  4913  ............................................................  4972

Query  4921  ATCTTAGATCCCACTGTTCCACCAACTTACAAACGGCCATGCATCAGACGGTACCCCTCC  4980
Sbjct  4973  ............................................................  5032

Query  4981  ACAATCTCTTGTAACTCCTCTGAGGACTCCAGGAGCTTGTCTACTTTTTCTGTCAGCTCC  5040
Sbjct  5033  ........C......................................C............  5092

Query  5041  GACTCCTCGATTGGTTCTCTGCCGGTCGGAGACACGAGACCCATTCCAGCCCCGAGGACC  5100
Sbjct  5093  .................CG..A...................T...........A.....T  5152

Query  5101  ATTTTCAGACCCGTCCCTGCCCCGAGAGCACCCGTGCTCAGAACCACACCGCCTCCTAAA  5160
Sbjct  5153  G..........................................T................  5212

Query  5161  CCACCGCGCACATTCACCGTGCGTGCAGAAGTGCACCAAGCACCCCCTACACCTGTACCT  5220
Sbjct  5213  ...................................T........................  5272

Query  5221  CCACCCAGACCGAAGAGGGCTGCAAAGTTGGCTCGTGAGATGCACCCCGGGTTCACCTTC  5280
Sbjct  5273  ............................................................  5332

Query  5281  GGGGACTTCGGAGAGCACGAGGTTGAGGAGCTTACGGCCTCTCCCTTAACCTTCGGAGAT  5340
Sbjct  5333  ..A..................A.................G....................  5392

Query  5341  TTTGCTGAAGGAGAGATCCAGGGGATGGGAGTGGAGTTTGAATGACTAGGCAGAGCCGGC  5400
Sbjct  5393  ............................................................  5452

Query  5401  GGGTACATTTTTTCGTCAGACACGGGTCCAGGCCACCTACAGCAGAGATCCGTTTTACAA  5460
Sbjct  5453  ...................................TT....A..................  5512

Query  5461  AATTGCACGGCAGAATGTATCTACGAACCGGCAAAACTAGAAAAAATTCATGCACCAAAG  5520
Sbjct  5513  ............................................................  5572

Query  5521  TTGGATAAAACCAAGGAAGATATCTTAAGGAGCAAGTACCAAATGAAACCGTCTGAAGCA  5580
Sbjct  5573  ........G...................................................  5632

Query  5581  AACAAAAGCAGGTACCAATCTAGAAAAGTAGAAAATATGAAAGCAGAGATCGTAGGTAGA  5640
Sbjct  5633  ........T.........................................T.........  5692

Query  5641  CTCTTGGACGGACTGGGGGAGTATCTGGGCACCGAGCATCCAGTTGAATGCTACCGAATA  5700
Sbjct  5693  ..............A.......................C.....................  5752
```

```
Query  5701  ACGTACCCGGTGCCTATATACTCAACTAGTGACCTCAGAGGTCTGTCTAGTGCCAAAACA  5760
Sbjct  5753  .....T........................AT..G.........................  5812

Query  5761  GCTGTTAGAGCTTGCAATGCATTTTTGGAAGCTAATTTTCCATCAGTCACTTCATATAAA  5820
Sbjct  5813  ............................................................  5872

Query  5821  ATTACTGATGAATACGACGCATACCTAGATATGGTAGATGGATCAGAGAGCTGTCTGGAC  5880
Sbjct  5873  ............................................................  5932

Query  5881  AGATCCTCCTTTTCGCCGTCTAGATTGCGTAGCTTTCCAAAAACACACTCATACTTGGAC  5940
Sbjct  5933  .................A...........C..............................  5992

Query  5941  CCACAGATCAACAGTGCGGTACCGTCACCATTCCAAAACACCTTACAAAATGTATTGGCA  6000
Sbjct  5993  ..G.......................G.................................  6052

Query  6001  GCGGCCACCAAAAGAAACTGTAATGTCACACAGATGAGAGAACTACCAACATATGATTCT  6060
Sbjct  6053  ...............................................G............  6112

Query  6061  GCAGTGCTAAATGTAGAGGCCTTCAGGAAATATGCGTGCAAGCCAGACGTATGGGATGAG  6120
Sbjct  6113  ............................................................  6172

Query  6121  TACAGGGATAATCCGATTTGCATAACCACCGAAAATGTCACCACTTACGTCGCCAAGTTG  6180
Sbjct  6173  ..............A..............A.......................T......  6232

Query  6181  AAAGGACCGAAAGCTGCGGCCTTGTTTGCAAAAACACATAACCTGATACCACTACACCAA  6240
Sbjct  6233  ..........................................................G.  6292

Query  6241  GTTCCTATGGACAAATTCACGGTAGATATGAAGAGAGATGTCAAAGTCACGCCCGGAACC  6300
Sbjct  6293  ............................................................  6352

Query  6301  AAGCACACCGAAGAGAGACCAAAGGTACAGGTGATTCAAGCGGCAGAGCCACTAGCCACT  6360
Sbjct  6353  ...................................................T........  6412

Query  6361  GCCTACCTCTGCGGAATTCACCGTGAATTGGTGCGCCGTCTCAACAACGCGCTTTTCCCA  6420
Sbjct  6413  .....................................................C......  6472

Query  6421  AACATCCACACTTTGTTTGATATGTCCGCAGAGGATTTCGATGCAATCATAGCGGAACAT  6480
Sbjct  6473  ..T................................C..T.....................  6532

Query  6481  TTTAAGCACGGTGACCATGTGTTGGAAACGGATATAGCCTCTTTTGACAAAAGTCAAGAT  6540
Sbjct  6533  .................C..........................................  6592

Query  6541  GATTCCATGGCACTCACTGCGTTAATGATCCTTGAGGACCTGGGAGTAGACCAAAACCTA  6600
Sbjct  6593  ............................................................  6652

Query  6601  ATGAATTTGATAGAGGCTGCATTCGGGGAAATCGTGAGTACACACTTGCCCACAGGTACT  6660
Sbjct  6653  ............................................................  6712

Query  6661  AGATTCAAATTTGGAGCTATGATGAAGTCTGGAATGTTTTTGACGCTGTTCGTCAATACA  6720
Sbjct  6713  .......G.....................C...............T..............  6772

Query  6721  ATTCTTAATGTGGTTATTGCGTGCCGAGTGTTGGAGGATCAATTGGCGCAGTCGCCGTGG  6780
Sbjct  6773  ........C......................................A...........C  6832
```

```
Query  6781  CCTGCTTTCATAGGAGATGACAACATAATCCATGGTATAATATCAGACAAATTGATGGCA  6840
Sbjct  6833  G..........................................................G  6892

Query  6841  GATAGATGTGCCACCTGGATGAACATGGAGGTCAAGATACTGGACTCTATAGTTGGAATA  6900
Sbjct  6893  ..............T.............................T..C............  6952

Query  6901  CGGCCACCTTACTTCTGTGGAGGATTTATTGTATGTGACGATGTAACAGGTACAGCCTGC  6960
Sbjct  6953  ...........T................................................  7012

Query  6961  CGCGTCGCAGACCCACTGAAGAGATTGTTCAAGCTAGGTAAGCCATTGCCACTTGACGAT  7020
Sbjct  7013  ..............G.........C...................................  7072

Query  7021  GGCCAAGATGAAGACAGAAGACGTGCATTACATGATGAAGTGAAAACCTGGTCGCGCGTA  7080
Sbjct  7073  ........C...................................................  7132

Query  7081  GGGCTGCGACACAGAGTGTGTGAAGCCATCGAAGACCGTTATGCCGTCCACTCATCAGAA  7140
Sbjct  7133  ..................................................T.........  7192

Query  7141  CTAGTTTTATTGGCACTGACTACTCTGTCTAAGAACTTGAAGTCCTTCAGAAACATAAGA  7200
Sbjct  7193  ............................................................  7252

Query  7201  GGGAAACCAATACATCTCTACGGTGGTCCTAAATAG  7236
Sbjct  7253  ....................................  7288
```

>Barmah Forest virus isolate SW68009, complete genome
Sequence ID: MN689037.1 Length: 11525
Range 1: 60 to 7295

Score:12639 bits(6844), Expect:0.0,
Identities:7105/7236(98%), Gaps:0/7236(0%), Strand: Plus/Plus

```
Query  1     ATGGCGAAACCAGTTGTGAAGATCGACGTGGAACCTGAAAGCCATTTCGCTAAGCAGGTC  60
Sbjct  60    ...............................................T............  119

Query  61    CAGAGTTGCTTCCCGCAGTTTGAGATCGAAGCAGTGCAGACCACACCAAACGATCATGCA  120
Sbjct  120   .........................................G..................  179

Query  121   CACGCGAGGGCGTTTTCGCACCTTGCTACGAAGCTCATAGAAATGGAGACAGCAAAAGAT  180
Sbjct  180   ............................................................  239

Query  181   CAGATCATCCTCGATATCGGAAGTGCACCCGCGAGGAGACTGTATTCAGAACACAAGTAC  240
Sbjct  240   ..................................................G.........  299

Query  241   CACTGTGTTTGCCCAATGAAGTGCACGGAAGATCCAGAGAGAATGCTAGGATATGCACGT  300
Sbjct  300   ............................................................  359

Query  301   AAGTTGATCGCAGGCTCTGCGAAAGGGAAGGCAGAAAAGTTACGCGATCTCAGGGATGTC  360
Sbjct  360   ............................................................  419

Query  361   TTGGCTACGCCAGACATCGAGACGCAGTCGCTATGTCTCCACACAGACGCATCCTGCAGA  420
Sbjct  420   ............................................................  479
```

```
Query  421   TACCGCGGTGATGTTGCCGTGTATCAAGACGTGTATGCCATTGACGCACCTACCACGCTG  480
Sbjct  480   ..................................................C.........  539

Query  481   TACCACCAAGCGTTAAAGGGCGTCAGGACCGCATATTGGATAGGCTTTGATACAACGCCG  540
Sbjct  540   ...........................................................A  599

Query  541   TTCATGTACGATGCACTAGCAGGAGCTTACCCGCTCTACTCCACAAACTGGGCTGATGAG  600
Sbjct  600   .........A...........................................C......  659

Query  601   CAAGTGCTCGAGTCCAGAAACATTGGGCTATGTTCAGACAAAGTTTCTGAAGGGGGAAAG  660
Sbjct  660   ............................................................  719

Query  661   AAAGGGAGATCAATCCTCAGGAAGAAGTTCTTGAAGCAGTCAGACAGAGTCATGTTCTCT  720
Sbjct  720   ............................................................  779

Query  721   GTCGGCTCGACGTTGTATACGGAAAGCCGTAAATTACTGCAAAGTTGGCACCTGCCATCC  780
Sbjct  780   ............................................C...............  839

Query  781   ACATTCCATCTCAAAGGCAAATCTTCGTTCACGTGCCGCTGCGACACTATCGTCAGCTGC  840
Sbjct  840   ................................A..............C............  899

Query  841   GAAGGGTATGTTCTGAAGAAAATTACAATGTGTCCTGGAGTGACAGGCAAACCGATAGGA  900
Sbjct  900   ................................C..C........................  959

Query  901   TATGCCGTCACCCATCACAAAGAAGGATTCGTAGTCGGAAAAGTCACAGATACCATTCGC  960
Sbjct  960   ....................G.............................C.........  1019

Query  961   GGCGAGAGAGTCTCCTTCGCCGTGTGTACTTATGTACCAACAACACTCTGCGACCAGATG  1020
Sbjct  1020  ..............T.............................................  1079

Query  1021  ACCGGGATCCTAGCAACAGAAGTAACAGCCGATGATGCCCAGAAACTGCTGGTGGGTTTG  1080
Sbjct  1080  ........T...................................................  1139

Query  1081  AACCAGAGAATAGTAGTTAATGGTAGGACCCAGAGAAATACCAATACTATGAAGAACTAC  1140
Sbjct  1140  ............................................C...............  1199

Query  1141  CTGCTACCACTGGTTGCACAAGCGCTAGCAAAATGGGCGAAGGAAGCAAAACAGGATATG  1200
Sbjct  1200  ......................................A.....................  1259

Query  1201  GAAGATGAAAGACCCCTGAACGAACGCCAACGAACGCTAACGTGCCTCTGCTGCTGGGCA  1260
Sbjct  1260  ............................................................  1319

Query  1261  TTTAAGCGAAACAAACGCCACGCCATTTACAAGAGACCAGACACACAGAGTATAGTCAAG  1320
Sbjct  1320  ............................................................  1379

Query  1321  GTCCCTTGCGAATTCACAAGCTTTCCTTTGGTCAGCCTGTGGTCCGCTGGGATGTCTATA  1380
Sbjct  1380  ............................................................  1439

Query  1381  TCTCTTAGGCAGAAGTTGAAGATGATGCTGCAGGCGAGGCAGCCCACACAAATAGCAGCA  1440
Sbjct  1440  ............................................................  1499

Query  1441  GTGACTGAGGAACTCATACAAGAAGCAGCTGCAGTAGAGCAAGAGGCCGTGGATACGGCC  1500
Sbjct  1500  ............T.....................C.........................  1559
```

```
Query  1501  AATGCCGAGCTGGACCACGCCGCATGGCCCTCCATTGTGGATACGACAGAGCGCCATGTT  1560
Sbjct  1560  ...............................................G............  1619

Query  1561  GAGGTCGAAGTGGAAGAACTCGACCAGCGTGCAGGGGAAGGGGTAGTGGAAACACCTCGA  1620
Sbjct  1620  ............................................................  1679

Query  1621  AACTCTATCAAAGTTTCAACACAGATCGGGGACGCGTTAATCGGCAGTTACCTGATCCTA  1680
Sbjct  1680  ........T...................................................  1739

Query  1681  TCACCCCAAGCAGTCCTACGCAGCGAAAAATTAGCCTGCATACATGATCTTGCAGAGCAG  1740
Sbjct  1740  ............................................................  1799

Query  1741  GTTAAGTTGGTCACACACTCTGGCCGTAGTGGTAGGTACGCCGTCGACAAATACNACGGA  1800
Sbjct  1800  ..C.................C..T..............................G.....  1859

Query  1801  AGAGTACTAGTCCCTACAGGAGTGGCTATAGACATTCAATCGTTCCAGGCTCTCAGTGAG  1860
Sbjct  1860  ...................................C........................  1919

Query  1861  AGCGCGACCCTTGTGTACAACGAACGCGAGTTCGTTAACAGGAAGCTGTGGCACATAGCA  1920
Sbjct  1920  ..T..........................................T..............  1979

Query  1921  GTATACGGGGCAGCACTCAATACTGATGAAGAAGGATACGAGAAGGTCCCGGTAGAGAGA  1980
Sbjct  1980  ............................................................  2039

Query  1981  GCAGAATCAGATTATGTGTTTGATGTAGACCAAAAAATGTGCCTaaaaaaaGAGCAGGCA  2040
Sbjct  2040  .................A............A.............C...............  2099

Query  2041  TCAGGTTGGGTACTCTGTGGCGAACTAGTCAACCCCCCATTCCACGAATTCGCATATGAA  2100
Sbjct  2100  .....A......................................................  2159

Query  2101  GGGCTCCGCACGAGACCGTCAGCACCCTACAAGGTTCATACAGTAGGTGTGTACGGAGTG  2160
Sbjct  2160  ........T..T................................................  2219

Query  2161  CCAGGATCAGGCAAATCCGCAATAATCAAGAACACGGTCACCATGTCTGACCTAGTATTG  2220
Sbjct  2220  ............................................................  2279

Query  2221  AGTGGTAAGAAAGAGAACTGCTTAGAAATTATGAACGATGTACTTAAACACAGAGCTCTA  2280
Sbjct  2280  ............................................................  2339

Query  2281  CGTATCACAGCGAAGACCGTAGACTCAGTGTTATTAAACGGCGTGAAACACACGCCTAAC  2340
Sbjct  2340  .....T.............................G........................  2399

Query  2341  ATACTATACATCGACGAAGCGTTCTCATGCCATGCAGGGACTCTGTTGGCCACTATAGCC  2400
Sbjct  2400  ............................................................  2459

Query  2401  ATAGTCAGGCCCAAACAGAAAGTGGTACTGTGCGGAGACCCGAAACAATGCGGATTCTTC  2460
Sbjct  2460  T................T.........................................T  2519

Query  2461  AATATGATGCAACTGAAAGTTAATTACAATCATGACATCTGCTCAGAAGTCTTCCACAAA  2520
Sbjct  2520  .......................C....................................  2579

Query  2521  AGTATCTCTAGACGGTGCACCCAGGATATCACGGCCATCGTTTCCAAATTACATTACCAG  2580
Sbjct  2580  ............................................T...............  2639
```

```
Query  2581  GACCGAATGAGGACCACAAACCCCCGAAAAGGAGACATCATTATAGACACTACCGGCACT  2640
Sbjct  2640  ..................................................C.........  2699

Query  2641  ACCAAACCAGCCAAAACAGATCTGATTCTGACGTGCTTCAGGGGATGGGTGAAACAGTTG  2700
Sbjct  2700  ....................C.......................................  2759

Query  2701  CAGCAAGACTACAGAGGTAACGAAGTAATGACGGCTGCAGCGTCCCAAGGACTGACGAGG  2760
Sbjct  2760  ............................................................  2819

Query  2761  GCCTCCGTATATGCGGTTCGAACTAAAGTCAATGAGAACCCGCTATATGCACAGACCTCC  2820
Sbjct  2820  ............................................................  2879

Query  2821  GAGCACGTGAACGTGTTGTTAACACGCACAGAAAACAAGCTAGTATGGAAGACCTTGTCA  2880
Sbjct  2880  ..................C....................T....................  2939

Query  2881  ACAGATCCCTGGATTAAAACACTGACTAACCCACCTAGAGGGCACTATACCGCCACCATA  2940
Sbjct  2940  ............................................................  2999

Query  2941  GCAGAATGGGAAGCGGAACACCAGGGTATAATGAAGGCCATACAAGGGTATGCACCGCCC  3000
Sbjct  3000  ..............A.......................T.....................  3059

Query  3001  GTGAACACCTTCATGAACAAAGTAAATGTGTGCTGGGCAAAGACACTTACGCCTGTGCTG  3060
Sbjct  3060  ...............................................C..A.........  3119

Query  3061  GAAACTGCGGGTATCTCCCTGTCAGCAGAAGACTGGTCTGAACTGCTGCCCCCGTTTGCC  3120
Sbjct  3120  ............................................................  3179

Query  3121  CAGGACGTGGCGTACTCACCCGAGGTGGCATTAAACATCATATGCACGAAAATGTATGGG  3180
Sbjct  3180  ...............................................A............  3239

Query  3181  TTTGACTTAGACACTGGTCTTTTTTCCAGGCCATCAGTGCCAATGACATACACCAAAGAC  3240
Sbjct  3240  ........G.....C.............A.........A.....................  3299

Query  3241  CATTGGGATAACAGAGTTGGAGGGAAAATGTATGGATTCAGCCAACAAGCATACGATCAG  3300
Sbjct  3300  ............................................................  3359

Query  3301  CTGGCAAGACGACATCCGTACCTTCGAGGTAGAGAGAAATCAGGAATGCAGATCGTAGTC  3360
Sbjct  3360  ...................................A........................  3419

Query  3361  ACTGAAATGCGTATCCAGCGCCCAAGATCGGATGCCAACATCATCCCGATCAACCGCAGG  3420
Sbjct  3420  .....G......................................................  3479

Query  3421  CTCCCTCACTCACTCGTAGCCACACACGAGTATAGGCGAGCTGCACGGGCCGAGGAATTC  3480
Sbjct  3480  ............................................G...............  3539

Query  3481  TTCACCACGACACGAGGGTACACTATGCTGCTGGTCTCTGAGTATAACATGAACTTACCA  3540
Sbjct  3540  ..........T.................................................  3599

Query  3541  AACAAGAAGATCACCTGGCTGGCTCCGATAGGGACGCAGGGGGCCCATCACACCGCCAAC  3600
Sbjct  3600  ............................................................  3659

Query  3601  CTAAACTTGGGGATACCACCTCTGCTGGGCAGTTTTGATGCGGTGGTTGTGAACATGCCG  3660
Sbjct  3660  .....................T......................................  3719
```

```
Query  3661  ACTCCATTCCGGAACCATCACTACCAGCAATGTGAAGACCACGCGATGAAACTCCAGATG  3720
Sbjct  3720  ................................C...........................  3779

Query  3721  CTGGCAGGCGACGCACTGAGGCACATTAAACCTGGCGGATCATTGTGGGTCAAGGCATAC  3780
Sbjct  3780  ............................................................  3839

Query  3781  GGCTACGCAGACCGGCACAGCGAGCACGTGGTCTTGGCATTGGCTAGAAAGTTTAAAAGC  3840
Sbjct  3840  .......................................C....................  3899

Query  3841  TTCAGAGTCACACAACCCTCATGCGTGACTTCCAACACCGAGGTGTTTCTCCACTTCTCA  3900
Sbjct  3900  ...........G........G.....A.................................  3959

Query  3901  ATTTTTGACAATGGCAAACGCGCGATAGCCCTGCATTCAGCTAATAGGAAGGCTAACAGT  3960
Sbjct  3960  ............................................................  4019

Query  3961  ATCTTCCAAAACACCTTCTTACCGGCGGGCAGTGCACCGGCGTACAGAGTCAAACGTGGA  4020
Sbjct  4020  ..........................A....A............................  4079

Query  4021  GACATTTCGAACGCCCCAGAGGATGCAGTGGTCAATGCAGCAAACCAACAGGGAGTGAAG  4080
Sbjct  4080  ............................................................  4139

Query  4081  GGTGCTGGAGTTTGCGGTGCAATTTACCGTAAGTGGCCGGACGCTTTCGGTGATGTCGCT  4140
Sbjct  4140  ............................................................  4199

Query  4141  ACTCCAACCGGAACAGCAGTTTCGAAATCCGTCCAAGATAAATTGGTGATCCACGCTGTC  4200
Sbjct  4200  ............................................................  4259

Query  4201  GGCCCGAATTTCTCAAAATGTTCAGAAGAGGAAGGGGACAGAGACCTAGCATCTGCTTAC  4260
Sbjct  4260  ............................................................  4319

Query  4261  AGAGCTGCAGCAGAAATAGTGATGGATaaaaaaaTTACAACAGTGGCCGTCCCCTTACTC  4320
Sbjct  4320  ..........................A.................................  4379

Query  4321  TCCACCGGCATTTATGCCGGAGGAAAAAACAGAGTAGAACAGTCACTCAACCATCTCTTC  4380
Sbjct  4380  ..............C........G....................................  4439

Query  4381  ACGGCATTCGACAATACTGATGCAGATGTGACCATATATTGCATGGACAAAACATGGGAA  4440
Sbjct  4440  ........T...................................................  4499

Query  4441  AAGAAGATTAAGGAGGCAATCGATCACCGGACTTCGGTTGAGATGGTGCAGGATGACGTG  4500
Sbjct  4500  ............................................................  4559

Query  4501  CAGTTGGAGGAGGAACTGGTACGAGTACACCCTTTGAGTAGTTTAGCAGGTAGGAAGGGT  4560
Sbjct  4560  ............................................................  4619

Query  4561  TACAGTACGGACAGCGGCCGAGTGTTTTCCTACCTGGAAGGTACCAAATTCCATCAGACT  4620
Sbjct  4620  ............................................................  4679

Query  4621  GCGGTGGACATAGCCGAAATGCAAGTGCTGTGGCCCGCCCTCAAAGAGTCTAATGAGCAA  4680
Sbjct  4680  ........T...........A.................T.....................  4739

Query  4681  ATAGTGGCATACACCTTAGGAGAATCAATGGACCAGATACGTGGCAAGTGCCCGACAGAA  4740
Sbjct  4740  ............................................................  4799
```

```
Query  4741  GATACTGACGCCTCCACACCTCCACGGACTGTGCCGTGCCTCTGTCGATACGCCATGACA  4800
Sbjct  4800  ..C.........................................................  4859

Query  4801  CCAGAGAGAGTGTACCGACTTAAATGCACGAACACTACCCAATTTACGGTTTGCTCATCT  4860
Sbjct  4860  ..T.........................................C.....C.........  4919

Query  4861  TTTGAGTTGCCAAAGTATCACATTCAGGGAGTGCAGAGAGTAAAATGTGAAAGAATCATC  4920
Sbjct  4920  ............................................................  4979

Query  4921  ATCTTAGATCCCACTGTTCCACCAACTTACAAACGGCCATGCATCAGACGGTACCCCTCC  4980
Sbjct  4980  ..............C.............................................  5039

Query  4981  ACAATCTCTTGTAACTCCTCTGAGGACTCCAGGAGCTTGTCTACTTTTTCTGTCAGCTCC  5040
Sbjct  5040  ........C......................................C............  5099

Query  5041  GACTCCTCGATTGGTTCTCTGCCGGTCGGAGACACGAGACCCATTCCAGCCCCGAGGACC  5100
Sbjct  5100  .................CG..A...................T...........A.....T  5159

Query  5101  ATTTTCAGACCCGTCCCTGCCCCGAGAGCACCCGTGCTCAGAACCACACCGCCTCCTAAA  5160
Sbjct  5160  G..........................................T................  5219

Query  5161  CCACCGCGCACATTCACCGTGCGTGCAGAAGTGCACCAAGCACCCCCTACACCTGTACCT  5220
Sbjct  5220  ...................................T........................  5279

Query  5221  CCACCCAGACCGAAGAGGGCTGCAAAGTTGGCTCGTGAGATGCACCCCGGGTTCACCTTC  5280
Sbjct  5280  ............................................................  5339

Query  5281  GGGGACTTCGGAGAGCACGAGGTTGAGGAGCTTACGGCCTCTCCCTTAACCTTCGGAGAT  5340
Sbjct  5340  ..A..................A.................G....................  5399

Query  5341  TTTGCTGAAGGAGAGATCCAGGGGATGGGAGTGGAGTTTGAATGACTAGGCAGAGCCGGC  5400
Sbjct  5400  ............................................................  5459

Query  5401  GGGTACATTTTTTCGTCAGACACGGGTCCAGGCCACCTACAGCAGAGATCCGTTTTACAA  5460
Sbjct  5460  .........................................A..................  5519

Query  5461  AATTGCACGGCAGAATGTATCTACGAACCGGCAAAACTAGAAAAAATTCATGCACCAAAG  5520
Sbjct  5520  ............................................................  5579

Query  5521  TTGGATAAAACCAAGGAAGATATCTTAAGGAGCAAGTACCAAATGAAACCGTCTGAAGCA  5580
Sbjct  5580  ........G...................................................  5639

Query  5581  AACAAAAGCAGGTACCAATCTAGAAAAGTAGAAAATATGAAAGCAGAGATCGTAGGTAGA  5640
Sbjct  5640  ........T.........................................T.........  5699

Query  5641  CTCTTGGACGGACTGGGGGAGTATCTGGGCACCGAGCATCCAGTTGAATGCTACCGAATA  5700
Sbjct  5700  ..............A.......................C.....................  5759

Query  5701  ACGTACCCGGTGCCTATATACTCAACTAGTGACCTCAGAGGTCTGTCTAGTGCCAAAACA  5760
Sbjct  5760  .....T........................AT..G.........................  5819

Query  5761  GCTGTTAGAGCTTGCAATGCATTTTTGGAAGCTAATTTTCCATCAGTCACTTCATATAAA  5820
Sbjct  5820  ............................................................  5879
```

```
Query  5821  ATTACTGATGAATACGACGCATACCTAGATATGGTAGATGGATCAGAGAGCTGTCTGGAC  5880
Sbjct  5880  ............................................................  5939

Query  5881  AGATCCTCCTTTTCGCCGTCTAGATTGCGTAGCTTTCCAAAAACACACTCATACTTGGAC  5940
Sbjct  5940  .................A...........C..............................  5999

Query  5941  CCACAGATCAACAGTGCGGTACCGTCACCATTCCAAAACACCTTACAAAATGTATTGGCA  6000
Sbjct  6000  ..G.......................G.................................  6059

Query  6001  GCGGCCACCAAAAGAAACTGTAATGTCACACAGATGAGAGAACTACCAACATATGATTCT  6060
Sbjct  6060  ...............................................G............  6119

Query  6061  GCAGTGCTAAATGTAGAGGCCTTCAGGAAATATGCGTGCAAGCCAGACGTATGGGATGAG  6120
Sbjct  6120  ............................................................  6179

Query  6121  TACAGGGATAATCCGATTTGCATAACCACCGAAAATGTCACCACTTACGTCGCCAAGTTG  6180
Sbjct  6180  ..............A......................................T......  6239

Query  6181  AAAGGACCGAAAGCTGCGGCCTTGTTTGCAAAAACACATAACCTGATACCACTACACCAA  6240
Sbjct  6240  ............................................................  6299

Query  6241  GTTCCTATGGACAAATTCACGGTAGATATGAAGAGAGATGTCAAAGTCACGCCCGGAACC  6300
Sbjct  6300  ............................................................  6359

Query  6301  AAGCACACCGAAGAGAGACCAAAGGTACAGGTGATTCAAGCGGCAGAGCCACTAGCCACT  6360
Sbjct  6360  ...................................................T........  6419

Query  6361  GCCTACCTCTGCGGAATTCACCGTGAATTGGTGCGCCGTCTCAACAACGCGCTTTTCCCA  6420
Sbjct  6420  .....................................................C......  6479

Query  6421  AACATCCACACTTTGTTTGATATGTCCGCAGAGGATTTCGATGCAATCATAGCGGAACAT  6480
Sbjct  6480  ..T................................C..T.....................  6539

Query  6481  TTTAAGCACGGTGACCATGTGTTGGAAACGGATATAGCCTCTTTTGACAAAAGTCAAGAT  6540
Sbjct  6540  .................C..........................................  6599

Query  6541  GATTCCATGGCACTCACTGCGTTAATGATCCTTGAGGACCTGGGAGTAGACCAAAACCTA  6600
Sbjct  6600  ............................................................  6659

Query  6601  ATGAATTTGATAGAGGCTGCATTCGGGGAAATCGTGAGTACACACTTGCCCACAGGTACT  6660
Sbjct  6660  ............................................................  6719

Query  6661  AGATTCAAATTTGGAGCTATGATGAAGTCTGGAATGTTTTTGACGCTGTTCGTCAATACA  6720
Sbjct  6720  .......G.....................C...............T..............  6779

Query  6721  ATTCTTAATGTGGTTATTGCGTGCCGAGTGTTGGAGGATCAATTGGCGCAGTCGCCGTGG  6780
Sbjct  6780  ........C......................................A...........C  6839

Query  6781  CCTGCTTTCATAGGAGATGACAACATAATCCATGGTATAATATCAGACAAATTGATGGCA  6840
Sbjct  6840  G..........................................................G  6899

Query  6841  GATAGATGTGCCACCTGGATGAACATGGAGGTCAAGATACTGGACTCTATAGTTGGAATA  6900
Sbjct  6900  ..............T.............................T..C............  6959
```

```
Query  6901  CGGCCACCTTACTTCTGTGGAGGATTTATTGTATGTGACGATGTAACAGGTACAGCCTGC  6960
Sbjct  6960  ...........T................................................  7019

Query  6961  CGCGTCGCAGACCCACTGAAGAGATTGTTCAAGCTAGGTAAGCCATTGCCACTTGACGAT  7020
Sbjct  7020  ..............G.........C...................................  7079

Query  7021  GGCCAAGATGAAGACAGAAGACGTGCATTACATGATGAAGTGAAAACCTGGTCGCGCGTA  7080
Sbjct  7080  ............................................................  7139

Query  7081  GGGCTGCGACACAGAGTGTGTGAAGCCATCGAAGACCGTTATGCCGTCCACTCATCAGAA  7140
Sbjct  7140  ..................................................T.........  7199

Query  7141  CTAGTTTTATTGGCACTGACTACTCTGTCTAAGAACTTGAAGTCCTTCAGAAACATAAGA  7200
Sbjct  7200  ............................................................  7259

Query  7201  GGGAAACCAATACATCTCTACGGTGGTCCTAAATAG  7236
Sbjct  7260  ....................................  7295
```

>Barmah Forest virus isolate SW76326, complete genome
Sequence ID: MN689039.1 Length: 11493
Range 1: 55 to 7290

Score:12628 bits(6838), Expect:0.0,
Identities:7103/7236(98%), Gaps:0/7236(0%), Strand: Plus/Plus

```
Query  1     ATGGCGAAACCAGTTGTGAAGATCGACGTGGAACCTGAAAGCCATTTCGCTAAGCAGGTC  60
Sbjct  55    ...............................................T............  114

Query  61    CAGAGTTGCTTCCCGCAGTTTGAGATCGAAGCAGTGCAGACCACACCAAACGATCATGCA  120
Sbjct  115   .........................................G..................  174

Query  121   CACGCGAGGGCGTTTTCGCACCTTGCTACGAAGCTCATAGAAATGGAGACAGCAAAAGAT  180
Sbjct  175   ............................................................  234

Query  181   CAGATCATCCTCGATATCGGAAGTGCACCCGCGAGGAGACTGTATTCAGAACACAAGTAC  240
Sbjct  235   ............................................................  294

Query  241   CACTGTGTTTGCCCAATGAAGTGCACGGAAGATCCAGAGAGAATGCTAGGATATGCACGT  300
Sbjct  295   ............................................................  354

Query  301   AAGTTGATCGCAGGCTCTGCGAAAGGGAAGGCAGAAAAGTTACGCGATCTCAGGGATGTC  360
Sbjct  355   ............................................................  414

Query  361   TTGGCTACGCCAGACATCGAGACGCAGTCGCTATGTCTCCACACAGACGCATCCTGCAGA  420
Sbjct  415   ............................................................  474

Query  421   TACCGCGGTGATGTTGCCGTGTATCAAGACGTGTATGCCATTGACGCACCTACCACGCTG  480
Sbjct  475   ..................................................C.........  534

Query  481   TACCACCAAGCGTTAAAGGGCGTCAGGACCGCATATTGGATAGGCTTTGATACAACGCCG  540
```

```
Sbjct  535   ...........................................................A  594

Query  541   TTCATGTACGATGCACTAGCAGGAGCTTACCCGCTCTACTCCACAAACTGGGCTGATGAG  600
Sbjct  595   .........A...........................................C......  654

Query  601   CAAGTGCTCGAGTCCAGAAACATTGGGCTATGTTCAGACAAAGTTTCTGAAGGGGGAAAG  660
Sbjct  655   ............................................................  714

Query  661   AAAGGGAGATCAATCCTCAGGAAGAAGTTCTTGAAGCAGTCAGACAGAGTCATGTTCTCT  720
Sbjct  715   ............................................................  774

Query  721   GTCGGCTCGACGTTGTATACGGAAAGCCGTAAATTACTGCAAAGTTGGCACCTGCCATCC  780
Sbjct  775   ............................................C...............  834

Query  781   ACATTCCATCTCAAAGGCAAATCTTCGTTCACGTGCCGCTGCGACACTATCGTCAGCTGC  840
Sbjct  835   ................................A..............C............  894

Query  841   GAAGGGTATGTTCTGAAGAAAATTACAATGTGTCCTGGAGTGACAGGCAAACCGATAGGA  900
Sbjct  895   ................................C..C........................  954

Query  901   TATGCCGTCACCCATCACAAAGAAGGATTCGTAGTCGGAAAAGTCACAGATACCATTCGC  960
Sbjct  955   ....................G.............................C.........  1014

Query  961   GGCGAGAGAGTCTCCTTCGCCGTGTGTACTTATGTACCAACAACACTCTGCGACCAGATG  1020
Sbjct  1015  ............................................................  1074

Query  1021  ACCGGGATCCTAGCAACAGAAGTAACAGCCGATGATGCCCAGAAACTGCTGGTGGGTTTG  1080
Sbjct  1075  ........T...................................................  1134

Query  1081  AACCAGAGAATAGTAGTTAATGGTAGGACCCAGAGAAATACCAATACTATGAAGAACTAC  1140
Sbjct  1135  ............................................C...............  1194

Query  1141  CTGCTACCACTGGTTGCACAAGCGCTAGCAAAATGGGCGAAGGAAGCAAAACAGGATATG  1200
Sbjct  1195  ......................................A.....................  1254

Query  1201  GAAGATGAAAGACCCCTGAACGAACGCCAACGAACGCTAACGTGCCTCTGCTGCTGGGCA  1260
Sbjct  1255  ............................................................  1314

Query  1261  TTTAAGCGAAACAAACGCCACGCCATTTACAAGAGACCAGACACACAGAGTATAGTCAAG  1320
Sbjct  1315  ............................................................  1374

Query  1321  GTCCCTTGCGAATTCACAAGCTTTCCTTTGGTCAGCCTGTGGTCCGCTGGGATGTCTATA  1380
Sbjct  1375  ..................G.........................................  1434

Query  1381  TCTCTTAGGCAGAAGTTGAAGATGATGCTGCAGGCGAGGCAGCCCACACAAATAGCAGCA  1440
Sbjct  1435  ............................................................  1494

Query  1441  GTGACTGAGGAACTCATACAAGAAGCAGCTGCAGTAGAGCAAGAGGCCGTGGATACGGCC  1500
Sbjct  1495  ............T.....................C.........................  1554

Query  1501  AATGCCGAGCTGGACCACGCCGCATGGCCCTCCATTGTGGATACGACAGAGCGCCATGTT  1560
Sbjct  1555  ...............................................G............  1614

Query  1561  GAGGTCGAAGTGGAAGAACTCGACCAGCGTGCAGGGGAAGGGGTAGTGGAAACACCTCGA  1620
```

```
Sbjct  1615  ............................................................  1674

Query  1621  AACTCTATCAAAGTTTCAACACAGATCGGGGACGCGTTAATCGGCAGTTACCTGATCCTA  1680
Sbjct  1675  ........T...................................................  1734

Query  1681  TCACCCCAAGCAGTCCTACGCAGCGAAAAATTAGCCTGCATACATGATCTTGCAGAGCAG  1740
Sbjct  1735  ............................................................  1794

Query  1741  GTTAAGTTGGTCACACACTCTGGCCGTAGTGGTAGGTACGCCGTCGACAAATACNACGGA  1800
Sbjct  1795  ..C.................C..T..............................G.....  1854

Query  1801  AGAGTACTAGTCCCTACAGGAGTGGCTATAGACATTCAATCGTTCCAGGCTCTCAGTGAG  1860
Sbjct  1855  ...................................C........................  1914

Query  1861  AGCGCGACCCTTGTGTACAACGAACGCGAGTTCGTTAACAGGAAGCTGTGGCACATAGCA  1920
Sbjct  1915  ..T..........................................T..............  1974

Query  1921  GTATACGGGGCAGCACTCAATACTGATGAAGAAGGATACGAGAAGGTCCCGGTAGAGAGA  1980
Sbjct  1975  ............................................................  2034

Query  1981  GCAGAATCAGATTATGTGTTTGATGTAGACCAAAAAATGTGCCTaaaaaaaGAGCAGGCA  2040
Sbjct  2035  .................A............A.............C...............  2094

Query  2041  TCAGGTTGGGTACTCTGTGGCGAACTAGTCAACCCCCCATTCCACGAATTCGCATATGAA  2100
Sbjct  2095  .....A......................................................  2154

Query  2101  GGGCTCCGCACGAGACCGTCAGCACCCTACAAGGTTCATACAGTAGGTGTGTACGGAGTG  2160
Sbjct  2155  ........T..T................................................  2214

Query  2161  CCAGGATCAGGCAAATCCGCAATAATCAAGAACACGGTCACCATGTCTGACCTAGTATTG  2220
Sbjct  2215  ............................................................  2274

Query  2221  AGTGGTAAGAAAGAGAACTGCTTAGAAATTATGAACGATGTACTTAAACACAGAGCTCTA  2280
Sbjct  2275  ............................................................  2334

Query  2281  CGTATCACAGCGAAGACCGTAGACTCAGTGTTATTAAACGGCGTGAAACACACGCCTAAC  2340
Sbjct  2335  .....T.............................G........................  2394

Query  2341  ATACTATACATCGACGAAGCGTTCTCATGCCATGCAGGGACTCTGTTGGCCACTATAGCC  2400
Sbjct  2395  ............................................................  2454

Query  2401  ATAGTCAGGCCCAAACAGAAAGTGGTACTGTGCGGAGACCCGAAACAATGCGGATTCTTC  2460
Sbjct  2455  T..........................................................T  2514

Query  2461  AATATGATGCAACTGAAAGTTAATTACAATCATGACATCTGCTCAGAAGTCTTCCACAAA  2520
Sbjct  2515  .......................C....................................  2574

Query  2521  AGTATCTCTAGACGGTGCACCCAGGATATCACGGCCATCGTTTCCAAATTACATTACCAG  2580
Sbjct  2575  ............................................T...............  2634

Query  2581  GACCGAATGAGGACCACAAACCCCCGAAAAGGAGACATCATTATAGACACTACCGGCACT  2640
Sbjct  2635  ..................................................C.........  2694

Query  2641  ACCAAACCAGCCAAAACAGATCTGATTCTGACGTGCTTCAGGGGATGGGTGAAACAGTTG  2700
```

```
Sbjct  2695  ....................C.......................................  2754

Query  2701  CAGCAAGACTACAGAGGTAACGAAGTAATGACGGCTGCAGCGTCCCAAGGACTGACGAGG  2760
Sbjct  2755  ............................................................  2814

Query  2761  GCCTCCGTATATGCGGTTCGAACTAAAGTCAATGAGAACCCGCTATATGCACAGACCTCC  2820
Sbjct  2815  ...............................................C............  2874

Query  2821  GAGCACGTGAACGTGTTGTTAACACGCACAGAAAACAAGCTAGTATGGAAGACCTTGTCA  2880
Sbjct  2875  ..................C....................T....................  2934

Query  2881  ACAGATCCCTGGATTAAAACACTGACTAACCCACCTAGAGGGCACTATACCGCCACCATA  2940
Sbjct  2935  ............................................................  2994

Query  2941  GCAGAATGGGAAGCGGAACACCAGGGTATAATGAAGGCCATACAAGGGTATGCACCGCCC  3000
Sbjct  2995  ..............A.......................T.....................  3054

Query  3001  GTGAACACCTTCATGAACAAAGTAAATGTGTGCTGGGCAAAGACACTTACGCCTGTGCTG  3060
Sbjct  3055  ...............................................C..A.........  3114

Query  3061  GAAACTGCGGGTATCTCCCTGTCAGCAGAAGACTGGTCTGAACTGCTGCCCCCGTTTGCC  3120
Sbjct  3115  ............................................................  3174

Query  3121  CAGGACGTGGCGTACTCACCCGAGGTGGCATTAAACATCATATGCACGAAAATGTATGGG  3180
Sbjct  3175  ...............................................A............  3234

Query  3181  TTTGACTTAGACACTGGTCTTTTTTCCAGGCCATCAGTGCCAATGACATACACCAAAGAC  3240
Sbjct  3235  ........G.....C.............A.........A.....................  3294

Query  3241  CATTGGGATAACAGAGTTGGAGGGAAAATGTATGGATTCAGCCAACAAGCATACGATCAG  3300
Sbjct  3295  ............................................................  3354

Query  3301  CTGGCAAGACGACATCCGTACCTTCGAGGTAGAGAGAAATCAGGAATGCAGATCGTAGTC  3360
Sbjct  3355  ...................................A........................  3414

Query  3361  ACTGAAATGCGTATCCAGCGCCCAAGATCGGATGCCAACATCATCCCGATCAACCGCAGG  3420
Sbjct  3415  .....G......................................................  3474

Query  3421  CTCCCTCACTCACTCGTAGCCACACACGAGTATAGGCGAGCTGCACGGGCCGAGGAATTC  3480
Sbjct  3475  ............................................G...............  3534

Query  3481  TTCACCACGACACGAGGGTACACTATGCTGCTGGTCTCTGAGTATAACATGAACTTACCA  3540
Sbjct  3535  ..........T.................................................  3594

Query  3541  AACAAGAAGATCACCTGGCTGGCTCCGATAGGGACGCAGGGGGCCCATCACACCGCCAAC  3600
Sbjct  3595  ............................................................  3654

Query  3601  CTAAACTTGGGGATACCACCTCTGCTGGGCAGTTTTGATGCGGTGGTTGTGAACATGCCG  3660
Sbjct  3655  ............................................................  3714

Query  3661  ACTCCATTCCGGAACCATCACTACCAGCAATGTGAAGACCACGCGATGAAACTCCAGATG  3720
Sbjct  3715  ................................C...........................  3774

Query  3721  CTGGCAGGCGACGCACTGAGGCACATTAAACCTGGCGGATCATTGTGGGTCAAGGCATAC  3780
```

```
Sbjct  3775  ............................................................  3834

Query  3781  GGCTACGCAGACCGGCACAGCGAGCACGTGGTCTTGGCATTGGCTAGAAAGTTTAAAAGC  3840
Sbjct  3835  .......................................C....................  3894

Query  3841  TTCAGAGTCACACAACCCTCATGCGTGACTTCCAACACCGAGGTGTTTCTCCACTTCTCA  3900
Sbjct  3895  ...........G.....A..G.....A.................................  3954

Query  3901  ATTTTTGACAATGGCAAACGCGCGATAGCCCTGCATTCAGCTAATAGGAAGGCTAACAGT  3960
Sbjct  3955  ............................................................  4014

Query  3961  ATCTTCCAAAACACCTTCTTACCGGCGGGCAGTGCACCGGCGTACAGAGTCAAACGTGGA  4020
Sbjct  4015  ..........................A....A..................T.........  4074

Query  4021  GACATTTCGAACGCCCCAGAGGATGCAGTGGTCAATGCAGCAAACCAACAGGGAGTGAAG  4080
Sbjct  4075  ............................................................  4134

Query  4081  GGTGCTGGAGTTTGCGGTGCAATTTACCGTAAGTGGCCGGACGCTTTCGGTGATGTCGCT  4140
Sbjct  4135  ............................................................  4194

Query  4141  ACTCCAACCGGAACAGCAGTTTCGAAATCCGTCCAAGATAAATTGGTGATCCACGCTGTC  4200
Sbjct  4195  ............................................................  4254

Query  4201  GGCCCGAATTTCTCAAAATGTTCAGAAGAGGAAGGGGACAGAGACCTAGCATCTGCTTAC  4260
Sbjct  4255  ............................................................  4314

Query  4261  AGAGCTGCAGCAGAAATAGTGATGGATaaaaaaaTTACAACAGTGGCCGTCCCCTTACTC  4320
Sbjct  4315  ..........................A.................................  4374

Query  4321  TCCACCGGCATTTATGCCGGAGGAAAAAACAGAGTAGAACAGTCACTCAACCATCTCTTC  4380
Sbjct  4375  ..............C........G....................................  4434

Query  4381  ACGGCATTCGACAATACTGATGCAGATGTGACCATATATTGCATGGACAAAACATGGGAA  4440
Sbjct  4435  ........T........C..........................................  4494

Query  4441  AAGAAGATTAAGGAGGCAATCGATCACCGGACTTCGGTTGAGATGGTGCAGGATGACGTG  4500
Sbjct  4495  ............................................................  4554

Query  4501  CAGTTGGAGGAGGAACTGGTACGAGTACACCCTTTGAGTAGTTTAGCAGGTAGGAAGGGT  4560
Sbjct  4555  ............................................................  4614

Query  4561  TACAGTACGGACAGCGGCCGAGTGTTTTCCTACCTGGAAGGTACCAAATTCCATCAGACT  4620
Sbjct  4615  ............................................................  4674

Query  4621  GCGGTGGACATAGCCGAAATGCAAGTGCTGTGGCCCGCCCTCAAAGAGTCTAATGAGCAA  4680
Sbjct  4675  ........T.............................T.....................  4734

Query  4681  ATAGTGGCATACACCTTAGGAGAATCAATGGACCAGATACGTGGCAAGTGCCCGACAGAA  4740
Sbjct  4735  ............................................................  4794

Query  4741  GATACTGACGCCTCCACACCTCCACGGACTGTGCCGTGCCTCTGTCGATACGCCATGACA  4800
Sbjct  4795  ..C.........................................................  4854

Query  4801  CCAGAGAGAGTGTACCGACTTAAATGCACGAACACTACCCAATTTACGGTTTGCTCATCT  4860
```

```
Sbjct  4855  ............................................C.....C.........  4914

Query  4861  TTTGAGTTGCCAAAGTATCACATTCAGGGAGTGCAGAGAGTAAAATGTGAAAGAATCATC  4920
Sbjct  4915  ............................................................  4974

Query  4921  ATCTTAGATCCCACTGTTCCACCAACTTACAAACGGCCATGCATCAGACGGTACCCCTCC  4980
Sbjct  4975  ............................................................  5034

Query  4981  ACAATCTCTTGTAACTCCTCTGAGGACTCCAGGAGCTTGTCTACTTTTTCTGTCAGCTCC  5040
Sbjct  5035  ........C......................................C............  5094

Query  5041  GACTCCTCGATTGGTTCTCTGCCGGTCGGAGACACGAGACCCATTCCAGCCCCGAGGACC  5100
Sbjct  5095  .................CG..A...................T...........A.....T  5154

Query  5101  ATTTTCAGACCCGTCCCTGCCCCGAGAGCACCCGTGCTCAGAACCACACCGCCTCCTAAA  5160
Sbjct  5155  G..........................................T................  5214

Query  5161  CCACCGCGCACATTCACCGTGCGTGCAGAAGTGCACCAAGCACCCCCTACACCTGTACCT  5220
Sbjct  5215  ...................................T........................  5274

Query  5221  CCACCCAGACCGAAGAGGGCTGCAAAGTTGGCTCGTGAGATGCACCCCGGGTTCACCTTC  5280
Sbjct  5275  ............................................................  5334

Query  5281  GGGGACTTCGGAGAGCACGAGGTTGAGGAGCTTACGGCCTCTCCCTTAACCTTCGGAGAT  5340
Sbjct  5335  ..A..................A.................G....................  5394

Query  5341  TTTGCTGAAGGAGAGATCCAGGGGATGGGAGTGGAGTTTGAATGACTAGGCAGAGCCGGC  5400
Sbjct  5395  ............................................................  5454

Query  5401  GGGTACATTTTTTCGTCAGACACGGGTCCAGGCCACCTACAGCAGAGATCCGTTTTACAA  5460
Sbjct  5455  ...................................TT....A..................  5514

Query  5461  AATTGCACGGCAGAATGTATCTACGAACCGGCAAAACTAGAAAAAATTCATGCACCAAAG  5520
Sbjct  5515  ............................................................  5574

Query  5521  TTGGATAAAACCAAGGAAGATATCTTAAGGAGCAAGTACCAAATGAAACCGTCTGAAGCA  5580
Sbjct  5575  ........G...................................................  5634

Query  5581  AACAAAAGCAGGTACCAATCTAGAAAAGTAGAAAATATGAAAGCAGAGATCGTAGGTAGA  5640
Sbjct  5635  ........T.........................................T.........  5694

Query  5641  CTCTTGGACGGACTGGGGGAGTATCTGGGCACCGAGCATCCAGTTGAATGCTACCGAATA  5700
Sbjct  5695  ..............A.......................C.....................  5754

Query  5701  ACGTACCCGGTGCCTATATACTCAACTAGTGACCTCAGAGGTCTGTCTAGTGCCAAAACA  5760
Sbjct  5755  .....T........................AT..G.........................  5814

Query  5761  GCTGTTAGAGCTTGCAATGCATTTTTGGAAGCTAATTTTCCATCAGTCACTTCATATAAA  5820
Sbjct  5815  ............................................................  5874

Query  5821  ATTACTGATGAATACGACGCATACCTAGATATGGTAGATGGATCAGAGAGCTGTCTGGAC  5880
Sbjct  5875  ............................................................  5934

Query  5881  AGATCCTCCTTTTCGCCGTCTAGATTGCGTAGCTTTCCAAAAACACACTCATACTTGGAC  5940
```

```
Sbjct  5935  .................A..........C.............................  5994

Query  5941  CCACAGATCAACAGTGCGGTACCGTCACCATTCCAAAACACCTTACAAAATGTATTGGCA  6000
Sbjct  5995  ..G.....................G...................................  6054

Query  6001  GCGGCCACCAAAAGAAACTGTAATGTCACACAGATGAGAGAACTACCAACATATGATTCT  6060
Sbjct  6055  ................................................G...........  6114

Query  6061  GCAGTGCTAAATGTAGAGGCCTTCAGGAAATATGCGTGCAAGCCAGACGTATGGGATGAG  6120
Sbjct  6115  ............................................................  6174

Query  6121  TACAGGGATAATCCGATTTGCATAACCACCGAAAATGTCACCACTTACGTCGCCAAGTTG  6180
Sbjct  6175  ..............A..............A......................T......  6234

Query  6181  AAAGGACCGAAAGCTGCGGCCTTGTTTGCAAAAACACATAACCTGATACCACTACACCAA  6240
Sbjct  6235  ............................................................  6294

Query  6241  GTTCCTATGGACAAATTCACGGTAGATATGAAGAGAGATGTCAAAGTCACGCCCGGAACC  6300
Sbjct  6295  ............................................................  6354

Query  6301  AAGCACACCGAAGAGAGACCAAAGGTACAGGTGATTCAAGCGGCAGAGCCACTAGCCACT  6360
Sbjct  6355  ...................................................T........  6414

Query  6361  GCCTACCTCTGCGGAATTCACCGTGAATTGGTGCGCCGTCTCAACAACGCGCTTTTCCCA  6420
Sbjct  6415  .....................................................C......  6474

Query  6421  AACATCCACACTTTGTTTGATATGTCCGCAGAGGATTTCGATGCAATCATAGCGGAACAT  6480
Sbjct  6475  ..T..............................C..T.......................  6534

Query  6481  TTTAAGCACGGTGACCATGTGTTGGAAACGGATATAGCCTCTTTTGACAAAAGTCAAGAT  6540
Sbjct  6535  .................C..........................................  6594

Query  6541  GATTCCATGGCACTCACTGCGTTAATGATCCTTGAGGACCTGGGAGTAGACCAAAACCTA  6600
Sbjct  6595  ............................................................  6654

Query  6601  ATGAATTTGATAGAGGCTGCATTCGGGGAAATCGTGAGTACACACTTGCCCACAGGTACT  6660
Sbjct  6655  ............................................................  6714

Query  6661  AGATTCAAATTTGGAGCTATGATGAAGTCTGGAATGTTTTTGACGCTGTTCGTCAATACA  6720
Sbjct  6715  .......G....................C...............T..............  6774

Query  6721  ATTCTTAATGTGGTTATTGCGTGCCGAGTGTTGGAGGATCAATTGGCGCAGTCGCCGTGG  6780
Sbjct  6775  ........C.....................................A...........C  6834

Query  6781  CCTGCTTTCATAGGAGATGACAACATAATCCATGGTATAATATCAGACAAATTGATGGCA  6840
Sbjct  6835  G..........................................................G  6894

Query  6841  GATAGATGTGCCACCTGGATGAACATGGAGGTCAAGATACTGGACTCTATAGTTGGAATA  6900
Sbjct  6895  ..............T..........................T..C...........  6954

Query  6901  CGGCCACCTTACTTCTGTGGAGGATTTATTGTATGTGACGATGTAACAGGTACAGCCTGC  6960
Sbjct  6955  ...........T................................................  7014

Query  6961  CGCGTCGCAGACCCACTGAAGAGATTGTTCAAGCTAGGTAAGCCATTGCCACTTGACGAT  7020
```

```
Sbjct  7015  ..............G.........C...................................  7074

Query  7021  GGCCAAGATGAAGACAGAAGACGTGCATTACATGATGAAGTGAAAACCTGGTCGCGCGTA  7080
Sbjct  7075  ........C...................................................  7134

Query  7081  GGGCTGCGACACAGAGTGTGTGAAGCCATCGAAGACCGTTATGCCGTCCACTCATCAGAA  7140
Sbjct  7135  ..................................................T.........  7194

Query  7141  CTAGTTTTATTGGCACTGACTACTCTGTCTAAGAACTTGAAGTCCTTCAGAAACATAAGA  7200
Sbjct  7195  ............................................................  7254

Query  7201  GGGAAACCAATACATCTCTACGGTGGTCCTAAATAG  7236
Sbjct  7255  ....................................  7290
```

>Barmah Forest virus isolate DC45960, complete genome
Sequence ID: MN689022.1 Length: 11487
Range 1: 54 to 7289

Score:12628 bits(6838), Expect:0.0,
Identities:7103/7236(98%),  Gaps:0/7236(0%), Strand: Plus/Plus

```
Query  1     ATGGCGAAACCAGTTGTGAAGATCGACGTGGAACCTGAAAGCCATTTCGCTAAGCAGGTC  60
Sbjct  54    ...............................................T............  113

Query  61    CAGAGTTGCTTCCCGCAGTTTGAGATCGAAGCAGTGCAGACCACACCAAACGATCATGCA  120
Sbjct  114   .........................................G..................  173

Query  121   CACGCGAGGGCGTTTTCGCACCTTGCTACGAAGCTCATAGAAATGGAGACAGCAAAAGAT  180
Sbjct  174   ............................................................  233

Query  181   CAGATCATCCTCGATATCGGAAGTGCACCCGCGAGGAGACTGTATTCAGAACACAAGTAC  240
Sbjct  234   ............................................................  293

Query  241   CACTGTGTTTGCCCAATGAAGTGCACGGAAGATCCAGAGAGAATGCTAGGATATGCACGT  300
Sbjct  294   ............................................................  353

Query  301   AAGTTGATCGCAGGCTCTGCGAAAGGGAAGGCAGAAAAGTTACGCGATCTCAGGGATGTC  360
Sbjct  354   ............................................................  413

Query  361   TTGGCTACGCCAGACATCGAGACGCAGTCGCTATGTCTCCACACAGACGCATCCTGCAGA  420
Sbjct  414   ............................................................  473

Query  421   TACCGCGGTGATGTTGCCGTGTATCAAGACGTGTATGCCATTGACGCACCTACCACGCTG  480
Sbjct  474   ..................................................C.........  533

Query  481   TACCACCAAGCGTTAAAGGGCGTCAGGACCGCATATTGGATAGGCTTTGATACAACGCCG  540
Sbjct  534   ...........................................................A  593

Query  541   TTCATGTACGATGCACTAGCAGGAGCTTACCCGCTCTACTCCACAAACTGGGCTGATGAG  600
Sbjct  594   .........A...........................................C......  653
```

```
Query  601   CAAGTGCTCGAGTCCAGAAACATTGGGCTATGTTCAGACAAAGTTTCTGAAGGGGGAAAG  660
Sbjct  654   ............................................................  713

Query  661   AAAGGGAGATCAATCCTCAGGAAGAAGTTCTTGAAGCAGTCAGACAGAGTCATGTTCTCT  720
Sbjct  714   ............................................................  773

Query  721   GTCGGCTCGACGTTGTATACGGAAAGCCGTAAATTACTGCAAAGTTGGCACCTGCCATCC  780
Sbjct  774   ............................................C...............  833

Query  781   ACATTCCATCTCAAAGGCAAATCTTCGTTCACGTGCCGCTGCGACACTATCGTCAGCTGC  840
Sbjct  834   ................................A..............C............  893

Query  841   GAAGGGTATGTTCTGAAGAAAATTACAATGTGTCCTGGAGTGACAGGCAAACCGATAGGA  900
Sbjct  894   ................................C..C........................  953

Query  901   TATGCCGTCACCCATCACAAAGAAGGATTCGTAGTCGGAAAAGTCACAGATACCATTCGC  960
Sbjct  954   ....................G.............................C.........  1013

Query  961   GGCGAGAGAGTCTCCTTCGCCGTGTGTACTTATGTACCAACAACACTCTGCGACCAGATG  1020
Sbjct  1014  ............................................................  1073

Query  1021  ACCGGGATCCTAGCAACAGAAGTAACAGCCGATGATGCCCAGAAACTGCTGGTGGGTTTG  1080
Sbjct  1074  ........T...................................................  1133

Query  1081  AACCAGAGAATAGTAGTTAATGGTAGGACCCAGAGAAATACCAATACTATGAAGAACTAC  1140
Sbjct  1134  ............................................C...............  1193

Query  1141  CTGCTACCACTGGTTGCACAAGCGCTAGCAAAATGGGCGAAGGAAGCAAAACAGGATATG  1200
Sbjct  1194  ......................................A.....................  1253

Query  1201  GAAGATGAAAGACCCCTGAACGAACGCCAACGAACGCTAACGTGCCTCTGCTGCTGGGCA  1260
Sbjct  1254  ............................................................  1313

Query  1261  TTTAAGCGAAACAAACGCCACGCCATTTACAAGAGACCAGACACACAGAGTATAGTCAAG  1320
Sbjct  1314  ............................................................  1373

Query  1321  GTCCCTTGCGAATTCACAAGCTTTCCTTTGGTCAGCCTGTGGTCCGCTGGGATGTCTATA  1380
Sbjct  1374  ............................................................  1433

Query  1381  TCTCTTAGGCAGAAGTTGAAGATGATGCTGCAGGCGAGGCAGCCCACACAAATAGCAGCA  1440
Sbjct  1434  ............................................................  1493

Query  1441  GTGACTGAGGAACTCATACAAGAAGCAGCTGCAGTAGAGCAAGAGGCCGTGGATACGGCC  1500
Sbjct  1494  ............T.....................C.........................  1553

Query  1501  AATGCCGAGCTGGACCACGCCGCATGGCCCTCCATTGTGGATACGACAGAGCGCCATGTT  1560
Sbjct  1554  ...............................................G............  1613

Query  1561  GAGGTCGAAGTGGAAGAACTCGACCAGCGTGCAGGGGAAGGGGTAGTGGAAACACCTCGA  1620
Sbjct  1614  ............................................................  1673

Query  1621  AACTCTATCAAAGTTTCAACACAGATCGGGGACGCGTTAATCGGCAGTTACCTGATCCTA  1680
Sbjct  1674  ........T...................................................  1733
```

```
Query  1681  TCACCCCAAGCAGTCCTACGCAGCGAAAAATTAGCCTGCATACATGATCTTGCAGAGCAG  1740
Sbjct  1734  ............................................................  1793

Query  1741  GTTAAGTTGGTCACACACTCTGGCCGTAGTGGTAGGTACGCCGTCGACAAATACNACGGA  1800
Sbjct  1794  ..C................C..T............................G.....  1853

Query  1801  AGAGTACTAGTCCCTACAGGAGTGGCTATAGACATTCAATCGTTCCAGGCTCTCAGTGAG  1860
Sbjct  1854  ................................C.......................  1913

Query  1861  AGCGCGACCCTTGTGTACAACGAACGCGAGTTCGTTAACAGGAAGCTGTGGCACATAGCA  1920
Sbjct  1914  ..T....................................T..............  1973

Query  1921  GTATACGGGGCAGCACTCAATACTGATGAAGAAGGATACGAGAAGGTCCCGGTAGAGAGA  1980
Sbjct  1974  ............................................................  2033

Query  1981  GCAGAATCAGATTATGTGTTTGATGTAGACCAAAAAATGTGCCTaaaaaaaGAGCAGGCA  2040
Sbjct  2034  ................A...........A......C......C..............  2093

Query  2041  TCAGGTTGGGTACTCTGTGGCGAACTAGTCAACCCCCCATTCCACGAATTCGCATATGAA  2100
Sbjct  2094  .....A......................................................  2153

Query  2101  GGGCTCCGCACGAGACCGTCAGCACCCTACAAGGTTCATACAGTAGGTGTGTACGGAGTG  2160
Sbjct  2154  ........T..T................................................  2213

Query  2161  CCAGGATCAGGCAAATCCGCAATAATCAAGAACACGGTCACCATGTCTGACCTAGTATTG  2220
Sbjct  2214  ............................................................  2273

Query  2221  AGTGGTAAGAAAGAGAACTGCTTAGAAATTATGAACGATGTACTTAAACACAGAGCTCTA  2280
Sbjct  2274  ............................................................  2333

Query  2281  CGTATCACAGCGAAGACCGTAGACTCAGTGTTATTAAACGGCGTGAAACACACGCCTAAC  2340
Sbjct  2334  .....T...........................G.......................  2393

Query  2341  ATACTATACATCGACGAAGCGTTCTCATGCCATGCAGGGACTCTGTTGGCCACTATAGCC  2400
Sbjct  2394  ............................................................  2453

Query  2401  ATAGTCAGGCCCAAACAGAAAGTGGTACTGTGCGGAGACCCGAAACAATGCGGATTCTTC  2460
Sbjct  2454  T..........................................................T  2513

Query  2461  AATATGATGCAACTGAAAGTTAATTACAATCATGACATCTGCTCAGAAGTCTTCCACAAA  2520
Sbjct  2514  .....................C....................................  2573

Query  2521  AGTATCTCTAGACGGTGCACCCAGGATATCACGGCCATCGTTTCCAAATTACATTACCAG  2580
Sbjct  2574  ............................................T..............  2633

Query  2581  GACCGAATGAGGACCACAAACCCCCGAAAAGGAGACATCATTATAGACACTACCGGCACT  2640
Sbjct  2634  ...................................................C........  2693

Query  2641  ACCAAACCAGCCAAAACAGATCTGATTCTGACGTGCTTCAGGGGATGGGTGAAACAGTTG  2700
Sbjct  2694  ..................C.........................................  2753

Query  2701  CAGCAAGACTACAGAGGTAACGAAGTAATGACGGCTGCAGCGTCCCAAGGACTGACGAGG  2760
Sbjct  2754  ............................................................  2813
```

```
Query  2761  GCCTCCGTATATGCGGTTCGAACTAAAGTCAATGAGAACCCGCTATATGCACAGACCTCC  2820
Sbjct  2814  ............................................................  2873

Query  2821  GAGCACGTGAACGTGTTGTTAACACGCACAGAAAACAAGCTAGTATGGAAGACCTTGTCA  2880
Sbjct  2874  ..................C....................T....................  2933

Query  2881  ACAGATCCCTGGATTAAAACACTGACTAACCCACCTAGAGGGCACTATACCGCCACCATA  2940
Sbjct  2934  ............................................................  2993

Query  2941  GCAGAATGGGAAGCGGAACACCAGGGTATAATGAAGGCCATACAAGGGTATGCACCGCCC  3000
Sbjct  2994  ..............A.......................T.....................  3053

Query  3001  GTGAACACCTTCATGAACAAAGTAAATGTGTGCTGGGCAAAGACACTTACGCCTGTGCTG  3060
Sbjct  3054  ...............................................C..A.........  3113

Query  3061  GAAACTGCGGGTATCTCCCTGTCAGCAGAAGACTGGTCTGAACTGCTGCCCCCGTTTGCC  3120
Sbjct  3114  ............................................................  3173

Query  3121  CAGGACGTGGCGTACTCACCCGAGGTGGCATTAAACATCATATGCACGAAAATGTATGGG  3180
Sbjct  3174  ...............................................A............  3233

Query  3181  TTTGACTTAGACACTGGTCTTTTTTCCAGGCCATCAGTGCCAATGACATACACCAAAGAC  3240
Sbjct  3234  ........G.....C.............A.........A.....................  3293

Query  3241  CATTGGGATAACAGAGTTGGAGGGAAAATGTATGGATTCAGCCAACAAGCATACGATCAG  3300
Sbjct  3294  ............................................................  3353

Query  3301  CTGGCAAGACGACATCCGTACCTTCGAGGTAGAGAGAAATCAGGAATGCAGATCGTAGTC  3360
Sbjct  3354  ...................................A........................  3413

Query  3361  ACTGAAATGCGTATCCAGCGCCCAAGATCGGATGCCAACATCATCCCGATCAACCGCAGG  3420
Sbjct  3414  .....G......................................................  3473

Query  3421  CTCCCTCACTCACTCGTAGCCACACACGAGTATAGGCGAGCTGCACGGGCCGAGGAATTC  3480
Sbjct  3474  ............................................G...............  3533

Query  3481  TTCACCACGACACGAGGGTACACTATGCTGCTGGTCTCTGAGTATAACATGAACTTACCA  3540
Sbjct  3534  ..........T.................................................  3593

Query  3541  AACAAGAAGATCACCTGGCTGGCTCCGATAGGGACGCAGGGGGCCCATCACACCGCCAAC  3600
Sbjct  3594  ............................................................  3653

Query  3601  CTAAACTTGGGGATACCACCTCTGCTGGGCAGTTTTGATGCGGTGGTTGTGAACATGCCG  3660
Sbjct  3654  ............................................................  3713

Query  3661  ACTCCATTCCGGAACCATCACTACCAGCAATGTGAAGACCACGCGATGAAACTCCAGATG  3720
Sbjct  3714  ................................C...........................  3773

Query  3721  CTGGCAGGCGACGCACTGAGGCACATTAAACCTGGCGGATCATTGTGGGTCAAGGCATAC  3780
Sbjct  3774  ............................................................  3833

Query  3781  GGCTACGCAGACCGGCACAGCGAGCACGTGGTCTTGGCATTGGCTAGAAAGTTTAAAAGC  3840
Sbjct  3834  .......................................C....................  3893
```

```
Query  3841  TTCAGAGTCACACAACCCTCATGCGTGACTTCCAACACCGAGGTGTTTCTCCACTTCTCA  3900
Sbjct  3894  ...........G.....A..G.....A.................................  3953

Query  3901  ATTTTTGACAATGGCAAACGCGCGATAGCCCTGCATTCAGCTAATAGGAAGGCTAACAGT  3960
Sbjct  3954  ............................................................  4013

Query  3961  ATCTTCCAAAACACCTTCTTACCGGCGGGCAGTGCACCGGCGTACAGAGTCAAACGTGGA  4020
Sbjct  4014  ..........................A....A............................  4073

Query  4021  GACATTTCGAACGCCCCAGAGGATGCAGTGGTCAATGCAGCAAACCAACAGGGAGTGAAG  4080
Sbjct  4074  ............................................................  4133

Query  4081  GGTGCTGGAGTTTGCGGTGCAATTTACCGTAAGTGGCCGGACGCTTTCGGTGATGTCGCT  4140
Sbjct  4134  ............................................................  4193

Query  4141  ACTCCAACCGGAACAGCAGTTTCGAAATCCGTCCAAGATAAATTGGTGATCCACGCTGTC  4200
Sbjct  4194  ............................T...............................  4253

Query  4201  GGCCCGAATTTCTCAAAATGTTCAGAAGAGGAAGGGGACAGAGACCTAGCATCTGCTTAC  4260
Sbjct  4254  ............................................................  4313

Query  4261  AGAGCTGCAGCAGAAATAGTGATGGATaaaaaaaTTACAACAGTGGCCGTCCCCTTACTC  4320
Sbjct  4314  ..........................A.................................  4373

Query  4321  TCCACCGGCATTTATGCCGGAGGAAAAAACAGAGTAGAACAGTCACTCAACCATCTCTTC  4380
Sbjct  4374  ..T...........C........G....................................  4433

Query  4381  ACGGCATTCGACAATACTGATGCAGATGTGACCATATATTGCATGGACAAAACATGGGAA  4440
Sbjct  4434  ........T...................................................  4493

Query  4441  AAGAAGATTAAGGAGGCAATCGATCACCGGACTTCGGTTGAGATGGTGCAGGATGACGTG  4500
Sbjct  4494  ............................................................  4553

Query  4501  CAGTTGGAGGAGGAACTGGTACGAGTACACCCTTTGAGTAGTTTAGCAGGTAGGAAGGGT  4560
Sbjct  4554  ............................................................  4613

Query  4561  TACAGTACGGACAGCGGCCGAGTGTTTTCCTACCTGGAAGGTACCAAATTCCATCAGACT  4620
Sbjct  4614  ............................................................  4673

Query  4621  GCGGTGGACATAGCCGAAATGCAAGTGCTGTGGCCCGCCCTCAAAGAGTCTAATGAGCAA  4680
Sbjct  4674  ........T.............................T.....................  4733

Query  4681  ATAGTGGCATACACCTTAGGAGAATCAATGGACCAGATACGTGGCAAGTGCCCGACAGAA  4740
Sbjct  4734  ............................................................  4793

Query  4741  GATACTGACGCCTCCACACCTCCACGGACTGTGCCGTGCCTCTGTCGATACGCCATGACA  4800
Sbjct  4794  ..C.........................................................  4853

Query  4801  CCAGAGAGAGTGTACCGACTTAAATGCACGAACACTACCCAATTTACGGTTTGCTCATCT  4860
Sbjct  4854  ............................................C.....C.........  4913

Query  4861  TTTGAGTTGCCAAAGTATCACATTCAGGGAGTGCAGAGAGTAAAATGTGAAAGAATCATC  4920
Sbjct  4914  ............................................................  4973
```

```
Query  4921  ATCTTAGATCCCACTGTTCCACCAACTTACAAACGGCCATGCATCAGACGGTACCCCTCC  4980
Sbjct  4974  ............................................................  5033

Query  4981  ACAATCTCTTGTAACTCCTCTGAGGACTCCAGGAGCTTGTCTACTTTTTCTGTCAGCTCC  5040
Sbjct  5034  ........C......................................C............  5093

Query  5041  GACTCCTCGATTGGTTCTCTGCCGGTCGGAGACACGAGACCCATTCCAGCCCCGAGGACC  5100
Sbjct  5094  .................CG..A...................T...........A.....T  5153

Query  5101  ATTTTCAGACCCGTCCCTGCCCCGAGAGCACCCGTGCTCAGAACCACACCGCCTCCTAAA  5160
Sbjct  5154  G..........................................T................  5213

Query  5161  CCACCGCGCACATTCACCGTGCGTGCAGAAGTGCACCAAGCACCCCCTACACCTGTACCT  5220
Sbjct  5214  ...................................T........................  5273

Query  5221  CCACCCAGACCGAAGAGGGCTGCAAAGTTGGCTCGTGAGATGCACCCCGGGTTCACCTTC  5280
Sbjct  5274  ............................................................  5333

Query  5281  GGGGACTTCGGAGAGCACGAGGTTGAGGAGCTTACGGCCTCTCCCTTAACCTTCGGAGAT  5340
Sbjct  5334  ..A..................A.................G....................  5393

Query  5341  TTTGCTGAAGGAGAGATCCAGGGGATGGGAGTGGAGTTTGAATGACTAGGCAGAGCCGGC  5400
Sbjct  5394  ............................................................  5453

Query  5401  GGGTACATTTTTTCGTCAGACACGGGTCCAGGCCACCTACAGCAGAGATCCGTTTTACAA  5460
Sbjct  5454  ...................................TT....A..................  5513

Query  5461  AATTGCACGGCAGAATGTATCTACGAACCGGCAAAACTAGAAAAAATTCATGCACCAAAG  5520
Sbjct  5514  ............................................................  5573

Query  5521  TTGGATAAAACCAAGGAAGATATCTTAAGGAGCAAGTACCAAATGAAACCGTCTGAAGCA  5580
Sbjct  5574  ........G...................................................  5633

Query  5581  AACAAAAGCAGGTACCAATCTAGAAAAGTAGAAAATATGAAAGCAGAGATCGTAGGTAGA  5640
Sbjct  5634  ........T.........................................T.........  5693

Query  5641  CTCTTGGACGGACTGGGGGAGTATCTGGGCACCGAGCATCCAGTTGAATGCTACCGAATA  5700
Sbjct  5694  ..............A.......................C.....................  5753

Query  5701  ACGTACCCGGTGCCTATATACTCAACTAGTGACCTCAGAGGTCTGTCTAGTGCCAAAACA  5760
Sbjct  5754  .....T........................AT..G.........................  5813

Query  5761  GCTGTTAGAGCTTGCAATGCATTTTTGGAAGCTAATTTTCCATCAGTCACTTCATATAAA  5820
Sbjct  5814  ............................................................  5873

Query  5821  ATTACTGATGAATACGACGCATACCTAGATATGGTAGATGGATCAGAGAGCTGTCTGGAC  5880
Sbjct  5874  ............................................................  5933

Query  5881  AGATCCTCCTTTTCGCCGTCTAGATTGCGTAGCTTTCCAAAAACACACTCATACTTGGAC  5940
Sbjct  5934  .................A...........C..............................  5993

Query  5941  CCACAGATCAACAGTGCGGTACCGTCACCATTCCAAAACACCTTACAAAATGTATTGGCA  6000
Sbjct  5994  ..G.......................G.................................  6053
```

```
Query  6001  GCGGCCACCAAAAGAAACTGTAATGTCACACAGATGAGAGAACTACCAACATATGATTCT  6060
Sbjct  6054  .......................C.......................G............  6113

Query  6061  GCAGTGCTAAATGTAGAGGCCTTCAGGAAATATGCGTGCAAGCCAGACGTATGGGATGAG  6120
Sbjct  6114  ............................................................  6173

Query  6121  TACAGGGATAATCCGATTTGCATAACCACCGAAAATGTCACCACTTACGTCGCCAAGTTG  6180
Sbjct  6174  ..............A..............A.......................T......  6233

Query  6181  AAAGGACCGAAAGCTGCGGCCTTGTTTGCAAAAACACATAACCTGATACCACTACACCAA  6240
Sbjct  6234  ............................................................  6293

Query  6241  GTTCCTATGGACAAATTCACGGTAGATATGAAGAGAGATGTCAAAGTCACGCCCGGAACC  6300
Sbjct  6294  ............................................................  6353

Query  6301  AAGCACACCGAAGAGAGACCAAAGGTACAGGTGATTCAAGCGGCAGAGCCACTAGCCACT  6360
Sbjct  6354  ...................................................T........  6413

Query  6361  GCCTACCTCTGCGGAATTCACCGTGAATTGGTGCGCCGTCTCAACAACGCGCTTTTCCCA  6420
Sbjct  6414  .....................................................C......  6473

Query  6421  AACATCCACACTTTGTTTGATATGTCCGCAGAGGATTTCGATGCAATCATAGCGGAACAT  6480
Sbjct  6474  ..T................................C..T.....................  6533

Query  6481  TTTAAGCACGGTGACCATGTGTTGGAAACGGATATAGCCTCTTTTGACAAAAGTCAAGAT  6540
Sbjct  6534  .................C..........................................  6593

Query  6541  GATTCCATGGCACTCACTGCGTTAATGATCCTTGAGGACCTGGGAGTAGACCAAAACCTA  6600
Sbjct  6594  ............................................................  6653

Query  6601  ATGAATTTGATAGAGGCTGCATTCGGGGAAATCGTGAGTACACACTTGCCCACAGGTACT  6660
Sbjct  6654  ............................................................  6713

Query  6661  AGATTCAAATTTGGAGCTATGATGAAGTCTGGAATGTTTTTGACGCTGTTCGTCAATACA  6720
Sbjct  6714  .......G.....................C...............T..............  6773

Query  6721  ATTCTTAATGTGGTTATTGCGTGCCGAGTGTTGGAGGATCAATTGGCGCAGTCGCCGTGG  6780
Sbjct  6774  ........C......................................A...........C  6833

Query  6781  CCTGCTTTCATAGGAGATGACAACATAATCCATGGTATAATATCAGACAAATTGATGGCA  6840
Sbjct  6834  G..........................................................G  6893

Query  6841  GATAGATGTGCCACCTGGATGAACATGGAGGTCAAGATACTGGACTCTATAGTTGGAATA  6900
Sbjct  6894  ..............T.............................T..C............  6953

Query  6901  CGGCCACCTTACTTCTGTGGAGGATTTATTGTATGTGACGATGTAACAGGTACAGCCTGC  6960
Sbjct  6954  ...........T................................................  7013

Query  6961  CGCGTCGCAGACCCACTGAAGAGATTGTTCAAGCTAGGTAAGCCATTGCCACTTGACGAT  7020
Sbjct  7014  ..............G.........C...................................  7073

Query  7021  GGCCAAGATGAAGACAGAAGACGTGCATTACATGATGAAGTGAAAACCTGGTCGCGCGTA  7080
Sbjct  7074  ........C...................................................  7133
```

```
Query  7081  GGGCTGCGACACAGAGTGTGTGAAGCCATCGAAGACCGTTATGCCGTCCACTCATCAGAA  7140
Sbjct  7134  ..................................................T.........  7193

Query  7141  CTAGTTTTATTGGCACTGACTACTCTGTCTAAGAACTTGAAGTCCTTCAGAAACATAAGA  7200
Sbjct  7194  ............................................................  7253

Query  7201  GGGAAACCAATACATCTCTACGGTGGTCCTAAATAG  7236
Sbjct  7254  ....................................  7289
```

```
>Barmah Forest virus isolate EGR27629, complete genome
Sequence ID: MN689025.1 Length: 11499
Range 1: 57 to 7292

Score:12622 bits(6835), Expect:0.0,
Identities:7102/7236(98%),  Gaps:0/7236(0%), Strand: Plus/Plus

Query  1     ATGGCGAAACCAGTTGTGAAGATCGACGTGGAACCTGAAAGCCATTTCGCTAAGCAGGTC  60
Sbjct  57    ...........G...................................T............  116

Query  61    CAGAGTTGCTTCCCGCAGTTTGAGATCGAAGCAGTGCAGACCACACCAAACGATCATGCA  120
Sbjct  117   .........................................G..................  176

Query  121   CACGCGAGGGCGTTTTCGCACCTTGCTACGAAGCTCATAGAAATGGAGACAGCAAAAGAT  180
Sbjct  177   ............................................................  236

Query  181   CAGATCATCCTCGATATCGGAAGTGCACCCGCGAGGAGACTGTATTCAGAACACAAGTAC  240
Sbjct  237   ............................................................  296

Query  241   CACTGTGTTTGCCCAATGAAGTGCACGGAAGATCCAGAGAGAATGCTAGGATATGCACGT  300
Sbjct  297   ............................................................  356

Query  301   AAGTTGATCGCAGGCTCTGCGAAAGGGAAGGCAGAAAAGTTACGCGATCTCAGGGATGTC  360
Sbjct  357   ............................................................  416

Query  361   TTGGCTACGCCAGACATCGAGACGCAGTCGCTATGTCTCCACACAGACGCATCCTGCAGA  420
Sbjct  417   ............................................................  476

Query  421   TACCGCGGTGATGTTGCCGTGTATCAAGACGTGTATGCCATTGACGCACCTACCACGCTG  480
Sbjct  477   ..................................................C.........  536

Query  481   TACCACCAAGCGTTAAAGGGCGTCAGGACCGCATATTGGATAGGCTTTGATACAACGCCG  540
Sbjct  537   ...........................................................A  596

Query  541   TTCATGTACGATGCACTAGCAGGAGCTTACCCGCTCTACTCCACAAACTGGGCTGATGAG  600
Sbjct  597   .........A...........................................C......  656

Query  601   CAAGTGCTCGAGTCCAGAAACATTGGGCTATGTTCAGACAAAGTTTCTGAAGGGGGAAAG  660
Sbjct  657   ............................................................  716

Query  661   AAAGGGAGATCAATCCTCAGGAAGAAGTTCTTGAAGCAGTCAGACAGAGTCATGTTCTCT  720
Sbjct  717   ............................................................  776
```

```
Query  721   GTCGGCTCGACGTTGTATACGGAAAGCCGTAAATTACTGCAAAGTTGGCACCTGCCATCC  780
Sbjct  777   ............................................C...............  836

Query  781   ACATTCCATCTCAAAGGCAAATCTTCGTTCACGTGCCGCTGCGACACTATCGTCAGCTGC  840
Sbjct  837   ................................A..............C............  896

Query  841   GAAGGGTATGTTCTGAAGAAAATTACAATGTGTCCTGGAGTGACAGGCAAACCGATAGGA  900
Sbjct  897   ................................C..C........................  956

Query  901   TATGCCGTCACCCATCACAAAGAAGGATTCGTAGTCGGAAAAGTCACAGATACCATTCGC  960
Sbjct  957   ....................G.............................C.........  1016

Query  961   GGCGAGAGAGTCTCCTTCGCCGTGTGTACTTATGTACCAACAACACTCTGCGACCAGATG  1020
Sbjct  1017  ............................................................  1076

Query  1021  ACCGGGATCCTAGCAACAGAAGTAACAGCCGATGATGCCCAGAAACTGCTGGTGGGTTTG  1080
Sbjct  1077  ........T...................................................  1136

Query  1081  AACCAGAGAATAGTAGTTAATGGTAGGACCCAGAGAAATACCAATACTATGAAGAACTAC  1140
Sbjct  1137  ............................................C...............  1196

Query  1141  CTGCTACCACTGGTTGCACAAGCGCTAGCAAAATGGGCGAAGGAAGCAAAACAGGATATG  1200
Sbjct  1197  ......................................A.....................  1256

Query  1201  GAAGATGAAAGACCCCTGAACGAACGCCAACGAACGCTAACGTGCCTCTGCTGCTGGGCA  1260
Sbjct  1257  ............................................................  1316

Query  1261  TTTAAGCGAAACAAACGCCACGCCATTTACAAGAGACCAGACACACAGAGTATAGTCAAG  1320
Sbjct  1317  ............................................................  1376

Query  1321  GTCCCTTGCGAATTCACAAGCTTTCCTTTGGTCAGCCTGTGGTCCGCTGGGATGTCTATA  1380
Sbjct  1377  ............................................................  1436

Query  1381  TCTCTTAGGCAGAAGTTGAAGATGATGCTGCAGGCGAGGCAGCCCACACAAATAGCAGCA  1440
Sbjct  1437  ............................................................  1496

Query  1441  GTGACTGAGGAACTCATACAAGAAGCAGCTGCAGTAGAGCAAGAGGCCGTGGATACGGCC  1500
Sbjct  1497  ..................................C.........................  1556

Query  1501  AATGCCGAGCTGGACCACGCCGCATGGCCCTCCATTGTGGATACGACAGAGCGCCATGTT  1560
Sbjct  1557  ...............................................G............  1616

Query  1561  GAGGTCGAAGTGGAAGAACTCGACCAGCGTGCAGGGGAAGGGGTAGTGGAAACACCTCGA  1620
Sbjct  1617  ............................................................  1676

Query  1621  AACTCTATCAAAGTTTCAACACAGATCGGGGACGCGTTAATCGGCAGTTACCTGATCCTA  1680
Sbjct  1677  ........T........T..........................................  1736

Query  1681  TCACCCCAAGCAGTCCTACGCAGCGAAAAATTAGCCTGCATACATGATCTTGCAGAGCAG  1740
Sbjct  1737  ............................................................  1796

Query  1741  GTTAAGTTGGTCACACACTCTGGCCGTAGTGGTAGGTACGCCGTCGACAAATACNACGGA  1800
Sbjct  1797  ..C.................C..T..............................G.....  1856
```

```
Query  1801  AGAGTACTAGTCCCTACAGGAGTGGCTATAGACATTCAATCGTTCCAGGCTCTCAGTGAG  1860
Sbjct  1857  ...................................C........................  1916

Query  1861  AGCGCGACCCTTGTGTACAACGAACGCGAGTTCGTTAACAGGAAGCTGTGGCACATAGCA  1920
Sbjct  1917  ..T..........................................T..............  1976

Query  1921  GTATACGGGGCAGCACTCAATACTGATGAAGAAGGATACGAGAAGGTCCCGGTAGAGAGA  1980
Sbjct  1977  ............................................................  2036

Query  1981  GCAGAATCAGATTATGTGTTTGATGTAGACCAAAAAATGTGCCTaaaaaaaGAGCAGGCA  2040
Sbjct  2037  .................A............A.............C...............  2096

Query  2041  TCAGGTTGGGTACTCTGTGGCGAACTAGTCAACCCCCCATTCCACGAATTCGCATATGAA  2100
Sbjct  2097  .....A..................T...................................  2156

Query  2101  GGGCTCCGCACGAGACCGTCAGCACCCTACAAGGTTCATACAGTAGGTGTGTACGGAGTG  2160
Sbjct  2157  ........T..T................................................  2216

Query  2161  CCAGGATCAGGCAAATCCGCAATAATCAAGAACACGGTCACCATGTCTGACCTAGTATTG  2220
Sbjct  2217  ............................................................  2276

Query  2221  AGTGGTAAGAAAGAGAACTGCTTAGAAATTATGAACGATGTACTTAAACACAGAGCTCTA  2280
Sbjct  2277  ............................................................  2336

Query  2281  CGTATCACAGCGAAGACCGTAGACTCAGTGTTATTAAACGGCGTGAAACACACGCCTAAC  2340
Sbjct  2337  .....T.............................G........................  2396

Query  2341  ATACTATACATCGACGAAGCGTTCTCATGCCATGCAGGGACTCTGTTGGCCACTATAGCC  2400
Sbjct  2397  ............................................................  2456

Query  2401  ATAGTCAGGCCCAAACAGAAAGTGGTACTGTGCGGAGACCCGAAACAATGCGGATTCTTC  2460
Sbjct  2457  T...........................................................  2516

Query  2461  AATATGATGCAACTGAAAGTTAATTACAATCATGACATCTGCTCAGAAGTCTTCCACAAA  2520
Sbjct  2517  .......................C....................................  2576

Query  2521  AGTATCTCTAGACGGTGCACCCAGGATATCACGGCCATCGTTTCCAAATTACATTACCAG  2580
Sbjct  2577  ............................................T...............  2636

Query  2581  GACCGAATGAGGACCACAAACCCCCGAAAAGGAGACATCATTATAGACACTACCGGCACT  2640
Sbjct  2637  ..................................................C.........  2696

Query  2641  ACCAAACCAGCCAAAACAGATCTGATTCTGACGTGCTTCAGGGGATGGGTGAAACAGTTG  2700
Sbjct  2697  ....................C.......................................  2756

Query  2701  CAGCAAGACTACAGAGGTAACGAAGTAATGACGGCTGCAGCGTCCCAAGGACTGACGAGG  2760
Sbjct  2757  ............................................................  2816

Query  2761  GCCTCCGTATATGCGGTTCGAACTAAAGTCAATGAGAACCCGCTATATGCACAGACCTCC  2820
Sbjct  2817  ............................................................  2876

Query  2821  GAGCACGTGAACGTGTTGTTAACACGCACAGAAAACAAGCTAGTATGGAAGACCTTGTCA  2880
Sbjct  2877  .......................................T....................  2936
```

```
Query  2881  ACAGATCCCTGGATTAAAACACTGACTAACCCACCTAGAGGGCACTATACCGCCACCATA  2940
Sbjct  2937  ............................................................  2996

Query  2941  GCAGAATGGGAAGCGGAACACCAGGGTATAATGAAGGCCATACAAGGGTATGCACCGCCC  3000
Sbjct  2997  ..............A.............................................  3056

Query  3001  GTGAACACCTTCATGAACAAAGTAAATGTGTGCTGGGCAAAGACACTTACGCCTGTGCTG  3060
Sbjct  3057  ...........T................................G..C..A.........  3116

Query  3061  GAAACTGCGGGTATCTCCCTGTCAGCAGAAGACTGGTCTGAACTGCTGCCCCCGTTTGCC  3120
Sbjct  3117  ..................T.........................................  3176

Query  3121  CAGGACGTGGCGTACTCACCCGAGGTGGCATTAAACATCATATGCACGAAAATGTATGGG  3180
Sbjct  3177  ...............................................A............  3236

Query  3181  TTTGACTTAGACACTGGTCTTTTTCCAGGCCATCAGTGCCAATGACATACACCAAAGAC   3240
Sbjct  3237  ........G.....C.............A.........A.....................  3296

Query  3241  CATTGGGATAACAGAGTTGGAGGGAAAATGTATGGATTCAGCCAACAAGCATACGATCAG  3300
Sbjct  3297  ............................................................  3356

Query  3301  CTGGCAAGACGACATCCGTACCTTCGAGGTAGAGAGAAATCAGGAATGCAGATCGTAGTC  3360
Sbjct  3357  ...................................A........................  3416

Query  3361  ACTGAAATGCGTATCCAGCGCCCAAGATCGGATGCCAACATCATCCCGATCAACCGCAGG  3420
Sbjct  3417  .....G......................................................  3476

Query  3421  CTCCCTCACTCACTCGTAGCCACACACGAGTATAGGCGAGCTGCACGGGCCGAGGAATTC  3480
Sbjct  3477  ............................................G...............  3536

Query  3481  TTCACCACGACACGAGGGTACACTATGCTGCTGGTCTCTGAGTATAACATGAACTTACCA  3540
Sbjct  3537  ..........T.................................................  3596

Query  3541  AACAAGAAGATCACCTGGCTGGCTCCGATAGGGACGCAGGGGGCCCATCACACCGCCAAC  3600
Sbjct  3597  ............................................................  3656

Query  3601  CTAAACTTGGGGATACCACCTCTGCTGGGCAGTTTTGATGCGGTGGTTGTGAACATGCCG  3660
Sbjct  3657  ........................................T...................  3716

Query  3661  ACTCCATTCCGGAACCATCACTACCAGCAATGTGAAGACCACGCGATGAAACTCCAGATG  3720
Sbjct  3717  ................................C...........................  3776

Query  3721  CTGGCAGGCGACGCACTGAGGCACATTAAACCTGGCGGATCATTGTGGGTCAAGGCATAC  3780
Sbjct  3777  .............................G..............................  3836

Query  3781  GGCTACGCAGACCGGCACAGCGAGCACGTGGTCTTGGCATTGGCTAGAAAGTTTAAAAGC  3840
Sbjct  3837  .......................................C....................  3896

Query  3841  TTCAGAGTCACACAACCCTCATGCGTGACTTCCAACACCGAGGTGTTTCTCCACTTCTCA  3900
Sbjct  3897  ...........G........G.....A................................G  3956

Query  3901  ATTTTTGACAATGGCAAACGCGCGATAGCCCTGCATTCAGCTAATAGGAAGGCTAACAGT  3960
Sbjct  3957  ............................................................  4016
```

```
Query  3961  ATCTTCCAAAACACCTTCTTACCGGCGGGCAGTGCACCGGCGTACAGAGTCAAACGTGGA  4020
Sbjct  4017  ..........................A....A............................  4076

Query  4021  GACATTTCGAACGCCCCAGAGGATGCAGTGGTCAATGCAGCAAACCAACAGGGAGTGAAG  4080
Sbjct  4077  ............................................................  4136

Query  4081  GGTGCTGGAGTTTGCGGTGCAATTTACCGTAAGTGGCCGGACGCTTTCGGTGATGTCGCT  4140
Sbjct  4137  ............................................................  4196

Query  4141  ACTCCAACCGGAACAGCAGTTTCGAAATCCGTCCAAGATAAATTGGTGATCCACGCTGTC  4200
Sbjct  4197  ............................................................  4256

Query  4201  GGCCCGAATTTCTCAAAATGTTCAGAAGAGGAAGGGGACAGAGACCTAGCATCTGCTTAC  4260
Sbjct  4257  ...........T................................................  4316

Query  4261  AGAGCTGCAGCAGAAATAGTGATGGATaaaaaaaTTACAACAGTGGCCGTCCCCTTACTC  4320
Sbjct  4317  ..........................A...........................C.....  4376

Query  4321  TCCACCGGCATTTATGCCGGAGGAAAAAACAGAGTAGAACAGTCACTCAACCATCTCTTC  4380
Sbjct  4377  ..............C......A.G....................................  4436

Query  4381  ACGGCATTCGACAATACTGATGCAGATGTGACCATATATTGCATGGACAAAACATGGGAA  4440
Sbjct  4437  ........T...................................................  4496

Query  4441  AAGAAGATTAAGGAGGCAATCGATCACCGGACTTCGGTTGAGATGGTGCAGGATGACGTG  4500
Sbjct  4497  ............................................................  4556

Query  4501  CAGTTGGAGGAGGAACTGGTACGAGTACACCCTTTGAGTAGTTTAGCAGGTAGGAAGGGT  4560
Sbjct  4557  ......................................C.................A...  4616

Query  4561  TACAGTACGGACAGCGGCCGAGTGTTTTCCTACCTGGAAGGTACCAAATTCCATCAGACT  4620
Sbjct  4617  ............................................................  4676

Query  4621  GCGGTGGACATAGCCGAAATGCAAGTGCTGTGGCCCGCCCTCAAAGAGTCTAATGAGCAA  4680
Sbjct  4677  ........T.............................T.....................  4736

Query  4681  ATAGTGGCATACACCTTAGGAGAATCAATGGACCAGATACGTGGCAAGTGCCCGACAGAA  4740
Sbjct  4737  ............................................................  4796

Query  4741  GATACTGACGCCTCCACACCTCCACGGACTGTGCCGTGCCTCTGTCGATACGCCATGACA  4800
Sbjct  4797  ............................................................  4856

Query  4801  CCAGAGAGAGTGTACCGACTTAAATGCACGAACACTACCCAATTTACGGTTTGCTCATCT  4860
Sbjct  4857  ............................................C.....C.........  4916

Query  4861  TTTGAGTTGCCAAAGTATCACATTCAGGGAGTGCAGAGAGTAAAATGTGAAAGAATCATC  4920
Sbjct  4917  ............................................................  4976

Query  4921  ATCTTAGATCCCACTGTTCCACCAACTTACAAACGGCCATGCATCAGACGGTACCCCTCC  4980
Sbjct  4977  ............................................................  5036

Query  4981  ACAATCTCTTGTAACTCCTCTGAGGACTCCAGGAGCTTGTCTACTTTTTCTGTCAGCTCC  5040
Sbjct  5037  ........C......................................C............  5096
```

```
Query  5041  GACTCCTCGATTGGTTCTCTGCCGGTCGGAGACACGAGACCCATTCCAGCCCCGAGGACC  5100
Sbjct  5097  .................CG..A...................T...........A......  5156

Query  5101  ATTTTCAGACCCGTCCCTGCCCCGAGAGCACCCGTGCTCAGAACCACACCGCCTCCTAAA  5160
Sbjct  5157  G...............................T..........T................  5216

Query  5161  CCACCGCGCACATTCACCGTGCGTGCAGAAGTGCACCAAGCACCCCCTACACCTGTACCT  5220
Sbjct  5217  ...................................T........................  5276

Query  5221  CCACCCAGACCGAAGAGGGCTGCAAAGTTGGCTCGTGAGATGCACCCCGGGTTCACCTTC  5280
Sbjct  5277  .....T......................................................  5336

Query  5281  GGGGACTTCGGAGAGCACGAGGTTGAGGAGCTTACGGCCTCTCCCTTAACCTTCGGAGAT  5340
Sbjct  5337  ..A..................A.................G....................  5396

Query  5341  TTTGCTGAAGGAGAGATCCAGGGGATGGGAGTGGAGTTTGAATGACTAGGCAGAGCCGGC  5400
Sbjct  5397  ....................A....C..................................  5456

Query  5401  GGGTACATTTTTTCGTCAGACACGGGTCCAGGCCACCTACAGCAGAGATCCGTTTTACAA  5460
Sbjct  5457  .........................................A.....G............  5516

Query  5461  AATTGCACGGCAGAATGTATCTACGAACCGGCAAAACTAGAAAAAATTCATGCACCAAAG  5520
Sbjct  5517  ............................................................  5576

Query  5521  TTGGATAAAACCAAGGAAGATATCTTAAGGAGCAAGTACCAAATGAAACCGTCTGAAGCA  5580
Sbjct  5577  ............................................................  5636

Query  5581  AACAAAAGCAGGTACCAATCTAGAAAAGTAGAAAATATGAAAGCAGAGATCGTAGGTAGA  5640
Sbjct  5637  ........T.........................................T.........  5696

Query  5641  CTCTTGGACGGACTGGGGGAGTATCTGGGCACCGAGCATCCAGTTGAATGCTACCGAATA  5700
Sbjct  5697  ..............A.............................................  5756

Query  5701  ACGTACCCGGTGCCTATATACTCAACTAGTGACCTCAGAGGTCTGTCTAGTGCCAAAACA  5760
Sbjct  5757  .....T....C...................AT..G.........................  5816

Query  5761  GCTGTTAGAGCTTGCAATGCATTTTTGGAAGCTAATTTTCCATCAGTCACTTCATATAAA  5820
Sbjct  5817  ............................................................  5876

Query  5821  ATTACTGATGAATACGACGCATACCTAGATATGGTAGATGGATCAGAGAGCTGTCTGGAC  5880
Sbjct  5877  ............................................................  5936

Query  5881  AGATCCTCCTTTTCGCCGTCTAGATTGCGTAGCTTTCCAAAAACACACTCATACTTGGAC  5940
Sbjct  5937  .................A...........C..............................  5996

Query  5941  CCACAGATCAACAGTGCGGTACCGTCACCATTCCAAAACACCTTACAAAATGTATTGGCA  6000
Sbjct  5997  ..G.........................................................  6056

Query  6001  GCGGCCACCAAAAGAAACTGTAATGTCACACAGATGAGAGAACTACCAACATATGATTCT  6060
Sbjct  6057  ...............................................G............  6116

Query  6061  GCAGTGCTAAATGTAGAGGCCTTCAGGAAATATGCGTGCAAGCCAGACGTATGGGATGAG  6120
Sbjct  6117  ............................................................  6176
```

```
Query  6121  TACAGGGATAATCCGATTTGCATAACCACCGAAAATGTCACCACTTACGTCGCCAAGTTG  6180
Sbjct  6177  ..............A...................................T..T......  6236

Query  6181  AAAGGACCGAAAGCTGCGGCCTTGTTTGCAAAAACACATAACCTGATACCACTACACCAA  6240
Sbjct  6237  ............................................................  6296

Query  6241  GTTCCTATGGACAAATTCACGGTAGATATGAAGAGAGATGTCAAAGTCACGCCCGGAACC  6300
Sbjct  6297  ............................................................  6356

Query  6301  AAGCACACCGAAGAGAGACCAAAGGTACAGGTGATTCAAGCGGCAGAGCCACTAGCCACT  6360
Sbjct  6357  ............................................................  6416

Query  6361  GCCTACCTCTGCGGAATTCACCGTGAATTGGTGCGCCGTCTCAACAACGCGCTTTTCCCA  6420
Sbjct  6417  .....................................................C.....T  6476

Query  6421  AACATCCACACTTTGTTTGATATGTCCGCAGAGGATTTCGATGCAATCATAGCGGAACAT  6480
Sbjct  6477  ..T...................................T.....................  6536

Query  6481  TTTAAGCACGGTGACCATGTGTTGGAAACGGATATAGCCTCTTTTGACAAAAGTCAAGAT  6540
Sbjct  6537  .................C..........................................  6596

Query  6541  GATTCCATGGCACTCACTGCGTTAATGATCCTTGAGGACCTGGGAGTAGACCAAAACCTA  6600
Sbjct  6597  ............................................................  6656

Query  6601  ATGAATTTGATAGAGGCTGCATTCGGGGAAATCGTGAGTACACACTTGCCCACAGGTACT  6660
Sbjct  6657  ............................................................  6716

Query  6661  AGATTCAAATTTGGAGCTATGATGAAGTCTGGAATGTTTTTGACGCTGTTCGTCAATACA  6720
Sbjct  6717  .......G.....................C...............T..............  6776

Query  6721  ATTCTTAATGTGGTTATTGCGTGCCGAGTGTTGGAGGATCAATTGGCGCAGTCGCCGTGG  6780
Sbjct  6777  ........C..................................................C  6836

Query  6781  CCTGCTTTCATAGGAGATGACAACATAATCCATGGTATAATATCAGACAAATTGATGGCA  6840
Sbjct  6837  G..........................................................G  6896

Query  6841  GATAGATGTGCCACCTGGATGAACATGGAGGTCAAGATACTGGACTCTATAGTTGGAATA  6900
Sbjct  6897  ............................................T..C............  6956

Query  6901  CGGCCACCTTACTTCTGTGGAGGATTTATTGTATGTGACGATGTAACAGGTACAGCCTGC  6960
Sbjct  6957  ...........T................................................  7016

Query  6961  CGCGTCGCAGACCCACTGAAGAGATTGTTCAAGCTAGGTAAGCCATTGCCACTTGACGAT  7020
Sbjct  7017  ........................C...................................  7076

Query  7021  GGCCAAGATGAAGACAGAAGACGTGCATTACATGATGAAGTGAAAACCTGGTCGCGCGTA  7080
Sbjct  7077  .............................G..............................  7136

Query  7081  GGGCTGCGACACAGAGTGTGTGAAGCCATCGAAGACCGTTATGCCGTCCACTCATCAGAA  7140
Sbjct  7137  ..................................................T.........  7196

Query  7141  CTAGTTTTATTGGCACTGACTACTCTGTCTAAGAACTTGAAGTCCTTCAGAAACATAAGA  7200
Sbjct  7197  ..............G.............................................  7256
```

```
Query  7201   GGGAAACCAATACATCTCTACGGTGGTCCTAAATAG  7236
Sbjct  7257   ....................................  7292
```

```
>Barmah Forest virus isolate MIDITullyA.2017, complete genome
Sequence ID: MN064696.1 Length: 11574
Range 1: 63 to 7298

Score:12617 bits(6832), Expect:0.0,
Identities:7101/7236(98%),  Gaps:0/7236(0%), Strand: Plus/Plus

Query  1      ATGGCGAAACCAGTTGTGAAGATCGACGTGGAACCTGAAAGCCATTTCGCTAAGCAGGTC  60
Sbjct  63     ...........G...................................T............  122

Query  61     CAGAGTTGCTTCCCGCAGTTTGAGATCGAAGCAGTGCAGACCACACCAAACGATCATGCA  120
Sbjct  123    .......................................G..................  182

Query  121    CACGCGAGGGCGTTTTCGCACCTTGCTACGAAGCTCATAGAAATGGAGACAGCAAAAGAT  180
Sbjct  183    ............................................................  242

Query  181    CAGATCATCCTCGATATCGGAAGTGCACCCGCGAGGAGACTGTATTCAGAACACAAGTAC  240
Sbjct  243    ............................................................  302

Query  241    CACTGTGTTTGCCCAATGAAGTGCACGGAAGATCCAGAGAGAATGCTAGGATATGCACGT  300
Sbjct  303    ............................................................  362

Query  301    AAGTTGATCGCAGGCTCTGCGAAAGGGAAGGCAGAAAAGTTACGCGATCTCAGGGATGTC  360
Sbjct  363    ............................................................  422

Query  361    TTGGCTACGCCAGACATCGAGACGCAGTCGCTATGTCTCCACACAGACGCATCCTGCAGA  420
Sbjct  423    ............................................................  482

Query  421    TACCGCGGTGATGTTGCCGTGTATCAAGACGTGTATGCCATTGACGCACCTACCACGCTG  480
Sbjct  483    ..................................................C.........  542

Query  481    TACCACCAAGCGTTAAAGGGCGTCAGGACCGCATATTGGATAGGCTTTGATACAACGCCG  540
Sbjct  543    ...........................................................A  602

Query  541    TTCATGTACGATGCACTAGCAGGAGCTTACCCGCTCTACTCCACAAACTGGGCTGATGAG  600
Sbjct  603    .........A.........................................C......  662

Query  601    CAAGTGCTCGAGTCCAGAAACATTGGGCTATGTTCAGACAAAGTTTCTGAAGGGGGAAAG  660
Sbjct  663    ............................................................  722

Query  661    AAAGGGAGATCAATCCTCAGGAAGAAGTTCTTGAAGCAGTCAGACAGAGTCATGTTCTCT  720
Sbjct  723    ............................................................  782

Query  721    GTCGGCTCGACGTTGTATACGGAAAGCCGTAAATTACTGCAAAGTTGGCACCTGCCATCC  780
Sbjct  783    ............................................C...............  842

Query  781    ACATTCCATCTCAAAGGCAAATCTTCGTTCACGTGCCGCTGCGACACTATCGTCAGCTGC  840
```

```
Sbjct  843   ................................A..............C............  902

Query  841   GAAGGGTATGTTCTGAAGAAAATTACAATGTGTCCTGGAGTGACAGGCAAACCGATAGGA  900
Sbjct  903   ................................C..C.............T..........  962

Query  901   TATGCCGTCACCCATCACAAAGAAGGATTCGTAGTCGGAAAAGTCACAGATACCATTCGC  960
Sbjct  963   ....................G.............................C.........  1022

Query  961   GGCGAGAGAGTCTCCTTCGCCGTGTGTACTTATGTACCAACAACACTCTGCGACCAGATG  1020
Sbjct  1023  ............................................................  1082

Query  1021  ACCGGGATCCTAGCAACAGAAGTAACAGCCGATGATGCCCAGAAACTGCTGGTGGGTTTG  1080
Sbjct  1083  ........T...................................................  1142

Query  1081  AACCAGAGAATAGTAGTTAATGGTAGGACCCAGAGAAATACCAATACTATGAAGAACTAC  1140
Sbjct  1143  ............................................C...............  1202

Query  1141  CTGCTACCACTGGTTGCACAAGCGCTAGCAAAATGGGCGAAGGAAGCAAAACAGGATATG  1200
Sbjct  1203  ......................................A.....................  1262

Query  1201  GAAGATGAAAGACCCCTGAACGAACGCCAACGAACGCTAACGTGCCTCTGCTGCTGGGCA  1260
Sbjct  1263  ............................................................  1322

Query  1261  TTTAAGCGAAACAAACGCCACGCCATTTACAAGAGACCAGACACACAGAGTATAGTCAAG  1320
Sbjct  1323  ............................................................  1382

Query  1321  GTCCCTTGCGAATTCACAAGCTTTCCTTTGGTCAGCCTGTGGTCCGCTGGGATGTCTATA  1380
Sbjct  1383  ............................................................  1442

Query  1381  TCTCTTAGGCAGAAGTTGAAGATGATGCTGCAGGCGAGGCAGCCCACACAAATAGCAGCA  1440
Sbjct  1443  .....C......................................................  1502

Query  1441  GTGACTGAGGAACTCATACAAGAAGCAGCTGCAGTAGAGCAAGAGGCCGTGGATACGGCC  1500
Sbjct  1503  ..................................C.........................  1562

Query  1501  AATGCCGAGCTGGACCACGCCGCATGGCCCTCCATTGTGGATACGACAGAGCGCCATGTT  1560
Sbjct  1563  ...............................................G............  1622

Query  1561  GAGGTCGAAGTGGAAGAACTCGACCAGCGTGCAGGGGAAGGGGTAGTGGAAACACCTCGA  1620
Sbjct  1623  ............................................................  1682

Query  1621  AACTCTATCAAAGTTTCAACACAGATCGGGGACGCGTTAATCGGCAGTTACCTGATCCTA  1680
Sbjct  1683  ........T........T..........................................  1742

Query  1681  TCACCCCAAGCAGTCCTACGCAGCGAAAAATTAGCCTGCATACATGATCTTGCAGAGCAG  1740
Sbjct  1743  ............................................................  1802

Query  1741  GTTAAGTTGGTCACACACTCTGGCCGTAGTGGTAGGTACGCCGTCGACAAATACNACGGA  1800
Sbjct  1803  ..C.................C..T..G...........................G.....  1862

Query  1801  AGAGTACTAGTCCCTACAGGAGTGGCTATAGACATTCAATCGTTCCAGGCTCTCAGTGAG  1860
Sbjct  1863  ...................................C........................  1922

Query  1861  AGCGCGACCCTTGTGTACAACGAACGCGAGTTCGTTAACAGGAAGCTGTGGCACATAGCA  1920
```

```
Sbjct  1923  ..T.......................................T..............  1982

Query  1921  GTATACGGGGCAGCACTCAATACTGATGAAGAAGGATACGAGAAGGTCCCGGTAGAGAGA  1980
Sbjct  1983  ............................................................  2042

Query  1981  GCAGAATCAGATTATGTGTTTGATGTAGACCAAAAAATGTGCCTaaaaaaaGAGCAGGCA  2040
Sbjct  2043  .................A...........A............C...............  2102

Query  2041  TCAGGTTGGGTACTCTGTGGCGAACTAGTCAACCCCCCATTCCACGAATTCGCATATGAA  2100
Sbjct  2103  .....A......................................................  2162

Query  2101  GGGCTCCGCACGAGACCGTCAGCACCCTACAAGGTTCATACAGTAGGTGTGTACGGAGTG  2160
Sbjct  2163  ........T..T................................................  2222

Query  2161  CCAGGATCAGGCAAATCCGCAATAATCAAGAACACGGTCACCATGTCTGACCTAGTATTG  2220
Sbjct  2223  ............................................................  2282

Query  2221  AGTGGTAAGAAAGAGAACTGCTTAGAAATTATGAACGATGTACTTAAACACAGAGCTCTA  2280
Sbjct  2283  ............................................................  2342

Query  2281  CGTATCACAGCGAAGACCGTAGACTCAGTGTTATTAAACGGCGTGAAACACACGCCTAAC  2340
Sbjct  2343  .....T.........................G.........................  2402

Query  2341  ATACTATACATCGACGAAGCGTTCTCATGCCATGCAGGGACTCTGTTGGCCACTATAGCC  2400
Sbjct  2403  ............................................................  2462

Query  2401  ATAGTCAGGCCCAAACAGAAAGTGGTACTGTGCGGAGACCCGAAACAATGCGGATTCTTC  2460
Sbjct  2463  T...........................................................  2522

Query  2461  AATATGATGCAACTGAAAGTTAATTACAATCATGACATCTGCTCAGAAGTCTTCCACAAA  2520
Sbjct  2523  .......................C....................................  2582

Query  2521  AGTATCTCTAGACGGTGCACCCAGGATATCACGGCCATCGTTTCCAAATTACATTACCAG  2580
Sbjct  2583  ..........................................T...............  2642

Query  2581  GACCGAATGAGGACCACAAACCCCCGAAAAGGAGACATCATTATAGACACTACCGGCACT  2640
Sbjct  2643  ..................................................C.........  2702

Query  2641  ACCAAACCAGCCAAAACAGATCTGATTCTGACGTGCTTCAGGGGATGGGTGAAACAGTTG  2700
Sbjct  2703  ....................C.......................................  2762

Query  2701  CAGCAAGACTACAGAGGTAACGAAGTAATGACGGCTGCAGCGTCCCAAGGACTGACGAGG  2760
Sbjct  2763  ............................................................  2822

Query  2761  GCCTCCGTATATGCGGTTCGAACTAAAGTCAATGAGAACCCGCTATATGCACAGACCTCC  2820
Sbjct  2823  ......................................T.....................  2882

Query  2821  GAGCACGTGAACGTGTTGTTAACACGCACAGAAAACAAGCTAGTATGGAAGACCTTGTCA  2880
Sbjct  2883  .......................................T....................  2942

Query  2881  ACAGATCCCTGGATTAAAACACTGACTAACCCACCTAGAGGGCACTATACCGCCACCATA  2940
Sbjct  2943  ............................................................  3002

Query  2941  GCAGAATGGGAAGCGGAACACCAGGGTATAATGAAGGCCATACAAGGGTATGCACCGCCC  3000
```

```
Sbjct  3003  ..............A.............................................  3062

Query  3001  GTGAACACCTTCATGAACAAAGTAAATGTGTGCTGGGCAAAGACACTTACGCCTGTGCTG  3060
Sbjct  3063  ...........T................................G..C..A.........  3122

Query  3061  GAAACTGCGGGTATCTCCCTGTCAGCAGAAGACTGGTCTGAACTGCTGCCCCCGTTTGCC  3120
Sbjct  3123  ..................T.........................................  3182

Query  3121  CAGGACGTGGCGTACTCACCCGAGGTGGCATTAAACATCATATGCACGAAAATGTATGGG  3180
Sbjct  3183  ...............................................A............  3242

Query  3181  TTTGACTTAGACACTGGTCTTTTTTCCAGGCCATCAGTGCCAATGACATACACCAAAGAC  3240
Sbjct  3243  ........G.....C.............A.........A.....................  3302

Query  3241  CATTGGGATAACAGAGTTGGAGGGAAAATGTATGGATTCAGCCAACAAGCATACGATCAG  3300
Sbjct  3303  ............................................................  3362

Query  3301  CTGGCAAGACGACATCCGTACCTTCGAGGTAGAGAGAAATCAGGAATGCAGATCGTAGTC  3360
Sbjct  3363  ...................................A........................  3422

Query  3361  ACTGAAATGCGTATCCAGCGCCCAAGATCGGATGCCAACATCATCCCGATCAACCGCAGG  3420
Sbjct  3423  .....G......................................................  3482

Query  3421  CTCCCTCACTCACTCGTAGCCACACACGAGTATAGGCGAGCTGCACGGGCCGAGGAATTC  3480
Sbjct  3483  ............................................G...............  3542

Query  3481  TTCACCACGACACGAGGGTACACTATGCTGCTGGTCTCTGAGTATAACATGAACTTACCA  3540
Sbjct  3543  ............................................................  3602

Query  3541  AACAAGAAGATCACCTGGCTGGCTCCGATAGGGACGCAGGGGGCCCATCACACCGCCAAC  3600
Sbjct  3603  ............................................................  3662

Query  3601  CTAAACTTGGGGATACCACCTCTGCTGGGCAGTTTTGATGCGGTGGTTGTGAACATGCCG  3660
Sbjct  3663  ........................................T...................  3722

Query  3661  ACTCCATTCCGGAACCATCACTACCAGCAATGTGAAGACCACGCGATGAAACTCCAGATG  3720
Sbjct  3723  ................................C...........................  3782

Query  3721  CTGGCAGGCGACGCACTGAGGCACATTAAACCTGGCGGATCATTGTGGGTCAAGGCATAC  3780
Sbjct  3783  .............................G..............................  3842

Query  3781  GGCTACGCAGACCGGCACAGCGAGCACGTGGTCTTGGCATTGGCTAGAAAGTTTAAAAGC  3840
Sbjct  3843  .......................................C....................  3902

Query  3841  TTCAGAGTCACACAACCCTCATGCGTGACTTCCAACACCGAGGTGTTTCTCCACTTCTCA  3900
Sbjct  3903  ...........G........G.....A................................G  3962

Query  3901  ATTTTTGACAATGGCAAACGCGCGATAGCCCTGCATTCAGCTAATAGGAAGGCTAACAGT  3960
Sbjct  3963  ............................................................  4022

Query  3961  ATCTTCCAAAACACCTTCTTACCGGCGGGCAGTGCACCGGCGTACAGAGTCAAACGTGGA  4020
Sbjct  4023  ..........................A....A............................  4082

Query  4021  GACATTTCGAACGCCCCAGAGGATGCAGTGGTCAATGCAGCAAACCAACAGGGAGTGAAG  4080
```

```
Sbjct  4083  ............................................................  4142

Query  4081  GGTGCTGGAGTTTGCGGTGCAATTTACCGTAAGTGGCCGGACGCTTTCGGTGATGTCGCT  4140
Sbjct  4143  ............................................................  4202

Query  4141  ACTCCAACCGGAACAGCAGTTTCGAAATCCGTCCAAGATAAATTGGTGATCCACGCTGTC  4200
Sbjct  4203  ............................................................  4262

Query  4201  GGCCCGAATTTCTCAAAATGTTCAGAAGAGGAAGGGGACAGAGACCTAGCATCTGCTTAC  4260
Sbjct  4263  ...........T................................................  4322

Query  4261  AGAGCTGCAGCAGAAATAGTGATGGATaaaaaaaTTACAACAGTGGCCGTCCCCTTACTC  4320
Sbjct  4323  ..........................G...........T...............C.....  4382

Query  4321  TCCACCGGCATTTATGCCGGAGGAAAAAACAGAGTAGAACAGTCACTCAACCATCTCTTC  4380
Sbjct  4383  ..............C........G....................................  4442

Query  4381  ACGGCATTCGACAATACTGATGCAGATGTGACCATATATTGCATGGACAAAACATGGGAA  4440
Sbjct  4443  ........T...................................................  4502

Query  4441  AAGAAGATTAAGGAGGCAATCGATCACCGGACTTCGGTTGAGATGGTGCAGGATGACGTG  4500
Sbjct  4503  ............................................................  4562

Query  4501  CAGTTGGAGGAGGAACTGGTACGAGTACACCCTTTGAGTAGTTTAGCAGGTAGGAAGGGT  4560
Sbjct  4563  ......................................C.................A...  4622

Query  4561  TACAGTACGGACAGCGGCCGAGTGTTTTCCTACCTGGAAGGTACCAAATTCCATCAGACT  4620
Sbjct  4623  ............................................................  4682

Query  4621  GCGGTGGACATAGCCGAAATGCAAGTGCTGTGGCCCGCCCTCAAAGAGTCTAATGAGCAA  4680
Sbjct  4683  ........T.............................T.....................  4742

Query  4681  ATAGTGGCATACACCTTAGGAGAATCAATGGACCAGATACGTGGCAAGTGCCCGACAGAA  4740
Sbjct  4743  ............................................................  4802

Query  4741  GATACTGACGCCTCCACACCTCCACGGACTGTGCCGTGCCTCTGTCGATACGCCATGACA  4800
Sbjct  4803  ............................................................  4862

Query  4801  CCAGAGAGAGTGTACCGACTTAAATGCACGAACACTACCCAATTTACGGTTTGCTCATCT  4860
Sbjct  4863  ............................................C.....C.........  4922

Query  4861  TTTGAGTTGCCAAAGTATCACATTCAGGGAGTGCAGAGAGTAAAATGTGAAAGAATCATC  4920
Sbjct  4923  ............................................................  4982

Query  4921  ATCTTAGATCCCACTGTTCCACCAACTTACAAACGGCCATGCATCAGACGGTACCCCTCC  4980
Sbjct  4983  ............................................................  5042

Query  4981  ACAATCTCTTGTAACTCCTCTGAGGACTCCAGGAGCTTGTCTACTTTTTCTGTCAGCTCC  5040
Sbjct  5043  ........C......................................C............  5102

Query  5041  GACTCCTCGATTGGTTCTCTGCCGGTCGGAGACACGAGACCCATTCCAGCCCCGAGGACC  5100
Sbjct  5103  .................CG..A...................T...........A......  5162

Query  5101  ATTTTCAGACCCGTCCCTGCCCCGAGAGCACCCGTGCTCAGAACCACACCGCCTCCTAAA  5160
```

```
Sbjct  5163  G.............................T.........T................  5222

Query  5161  CCACCGCGCACATTCACCGTGCGTGCAGAAGTGCACCAAGCACCCCCTACACCTGTACCT  5220
Sbjct  5223  ...................................T........................  5282

Query  5221  CCACCCAGACCGAAGAGGGCTGCAAAGTTGGCTCGTGAGATGCACCCCGGGTTCACCTTC  5280
Sbjct  5283  .....T......................................................  5342

Query  5281  GGGGACTTCGGAGAGCACGAGGTTGAGGAGCTTACGGCCTCTCCCTTAACCTTCGGAGAT  5340
Sbjct  5343  ..A..................A.................G....................  5402

Query  5341  TTTGCTGAAGGAGAGATCCAGGGGATGGGAGTGGAGTTTGAATGACTAGGCAGAGCCGGC  5400
Sbjct  5403  ....................A....C..................................  5462

Query  5401  GGGTACATTTTTTCGTCAGACACGGGTCCAGGCCACCTACAGCAGAGATCCGTTTTACAA  5460
Sbjct  5463  .........................................A.....G............  5522

Query  5461  AATTGCACGGCAGAATGTATCTACGAACCGGCAAAACTAGAAAAAATTCATGCACCAAAG  5520
Sbjct  5523  ............................................................  5582

Query  5521  TTGGATAAAACCAAGGAAGATATCTTAAGGAGCAAGTACCAAATGAAACCGTCTGAAGCA  5580
Sbjct  5583  ............................................................  5642

Query  5581  AACAAAAGCAGGTACCAATCTAGAAAAGTAGAAAATATGAAAGCAGAGATCGTAGGTAGA  5640
Sbjct  5643  ........T.........................................T.........  5702

Query  5641  CTCTTGGACGGACTGGGGGAGTATCTGGGCACCGAGCATCCAGTTGAATGCTACCGAATA  5700
Sbjct  5703  ..............A.............................................  5762

Query  5701  ACGTACCCGGTGCCTATATACTCAACTAGTGACCTCAGAGGTCTGTCTAGTGCCAAAACA  5760
Sbjct  5763  .....T....C...................AT..G.........................  5822

Query  5761  GCTGTTAGAGCTTGCAATGCATTTTTGGAAGCTAATTTTCCATCAGTCACTTCATATAAA  5820
Sbjct  5823  ............................................................  5882

Query  5821  ATTACTGATGAATACGACGCATACCTAGATATGGTAGATGGATCAGAGAGCTGTCTGGAC  5880
Sbjct  5883  ............................................................  5942

Query  5881  AGATCCTCCTTTTCGCCGTCTAGATTGCGTAGCTTTCCAAAAACACACTCATACTTGGAC  5940
Sbjct  5943  .................A...........C..............................  6002

Query  5941  CCACAGATCAACAGTGCGGTACCGTCACCATTCCAAAACACCTTACAAAATGTATTGGCA  6000
Sbjct  6003  ..G.........................................................  6062

Query  6001  GCGGCCACCAAAAGAAACTGTAATGTCACACAGATGAGAGAACTACCAACATATGATTCT  6060
Sbjct  6063  ...............................................G............  6122

Query  6061  GCAGTGCTAAATGTAGAGGCCTTCAGGAAATATGCGTGCAAGCCAGACGTATGGGATGAG  6120
Sbjct  6123  ............................................................  6182

Query  6121  TACAGGGATAATCCGATTTGCATAACCACCGAAAATGTCACCACTTACGTCGCCAAGTTG  6180
Sbjct  6183  ..............A....................................T..T......  6242

Query  6181  AAAGGACCGAAAGCTGCGGCCTTGTTTGCAAAAACACATAACCTGATACCACTACACCAA  6240
```

```
Sbjct  6243  ............................................................  6302

Query  6241  GTTCCTATGGACAAATTCACGGTAGATATGAAGAGAGATGTCAAAGTCACGCCCGGAACC  6300
Sbjct  6303  ............................................................  6362

Query  6301  AAGCACACCGAAGAGAGACCAAAGGTACAGGTGATTCAAGCGGCAGAGCCACTAGCCACT  6360
Sbjct  6363  ............................................................  6422

Query  6361  GCCTACCTCTGCGGAATTCACCGTGAATTGGTGCGCCGTCTCAACAACGCGCTTTTCCCA  6420
Sbjct  6423  .....................................................C.....T  6482

Query  6421  AACATCCACACTTTGTTTGATATGTCCGCAGAGGATTTCGATGCAATCATAGCGGAACAT  6480
Sbjct  6483  ..T...................................T.....................  6542

Query  6481  TTTAAGCACGGTGACCATGTGTTGGAAACGGATATAGCCTCTTTTGACAAAAGTCAAGAT  6540
Sbjct  6543  .................C..........................................  6602

Query  6541  GATTCCATGGCACTCACTGCGTTAATGATCCTTGAGGACCTGGGAGTAGACCAAAACCTA  6600
Sbjct  6603  ............................................................  6662

Query  6601  ATGAATTTGATAGAGGCTGCATTCGGGGAAATCGTGAGTACACACTTGCCCACAGGTACT  6660
Sbjct  6663  ............................................................  6722

Query  6661  AGATTCAAATTTGGAGCTATGATGAAGTCTGGAATGTTTTTGACGCTGTTCGTCAATACA  6720
Sbjct  6723  .......G.....................C...............T..............  6782

Query  6721  ATTCTTAATGTGGTTATTGCGTGCCGAGTGTTGGAGGATCAATTGGCGCAGTCGCCGTGG  6780
Sbjct  6783  ........C..................................................C  6842

Query  6781  CCTGCTTTCATAGGAGATGACAACATAATCCATGGTATAATATCAGACAAATTGATGGCA  6840
Sbjct  6843  G..........................................................G  6902

Query  6841  GATAGATGTGCCACCTGGATGAACATGGAGGTCAAGATACTGGACTCTATAGTTGGAATA  6900
Sbjct  6903  ............................................T..C............  6962

Query  6901  CGGCCACCTTACTTCTGTGGAGGATTTATTGTATGTGACGATGTAACAGGTACAGCCTGC  6960
Sbjct  6963  ...........T................................................  7022

Query  6961  CGCGTCGCAGACCCACTGAAGAGATTGTTCAAGCTAGGTAAGCCATTGCCACTTGACGAT  7020
Sbjct  7023  ........................C...................................  7082

Query  7021  GGCCAAGATGAAGACAGAAGACGTGCATTACATGATGAAGTGAAAACCTGGTCGCGCGTA  7080
Sbjct  7083  .............................G..............................  7142

Query  7081  GGGCTGCGACACAGAGTGTGTGAAGCCATCGAAGACCGTTATGCCGTCCACTCATCAGAA  7140
Sbjct  7143  ..................................................T.........  7202

Query  7141  CTAGTTTTATTGGCACTGACTACTCTGTCTAAGAACTTGAAGTCCTTCAGAAACATAAGA  7200
Sbjct  7203  ............................................................  7262

Query  7201  GGGAAACCAATACATCTCTACGGTGGTCCTAAATAG  7236
Sbjct  7263  ....................................  7298
```

```
>Barmah Forest virus isolate MIDITully.2017, complete genome
Sequence ID: MK697273.1 Length: 11489
Range 1: 63 to 7298

Score:12617 bits(6832), Expect:0.0,
Identities:7101/7236(98%),  Gaps:0/7236(0%), Strand: Plus/Plus

Query  1    ATGGCGAAACCAGTTGTGAAGATCGACGTGGAACCTGAAAGCCATTTCGCTAAGCAGGTC  60
Sbjct  63   ...........G...................................T............  122

Query  61   CAGAGTTGCTTCCCGCAGTTTGAGATCGAAGCAGTGCAGACCACACCAAACGATCATGCA  120
Sbjct  123  .........................................G..................  182

Query  121  CACGCGAGGGCGTTTTCGCACCTTGCTACGAAGCTCATAGAAATGGAGACAGCAAAAGAT  180
Sbjct  183  ............................................................  242

Query  181  CAGATCATCCTCGATATCGGAAGTGCACCCGCGAGGAGACTGTATTCAGAACACAAGTAC  240
Sbjct  243  ............................................................  302

Query  241  CACTGTGTTTGCCCAATGAAGTGCACGGAAGATCCAGAGAGAATGCTAGGATATGCACGT  300
Sbjct  303  ............................................................  362

Query  301  AAGTTGATCGCAGGCTCTGCGAAAGGGAAGGCAGAAAAGTTACGCGATCTCAGGGATGTC  360
Sbjct  363  ............................................................  422

Query  361  TTGGCTACGCCAGACATCGAGACGCAGTCGCTATGTCTCCACACAGACGCATCCTGCAGA  420
Sbjct  423  ............................................................  482

Query  421  TACCGCGGTGATGTTGCCGTGTATCAAGACGTGTATGCCATTGACGCACCTACCACGCTG  480
Sbjct  483  ..................................................C.........  542

Query  481  TACCACCAAGCGTTAAAGGGCGTCAGGACCGCATATTGGATAGGCTTTGATACAACGCCG  540
Sbjct  543  ...........................................................A  602

Query  541  TTCATGTACGATGCACTAGCAGGAGCTTACCCGCTCTACTCCACAAACTGGGCTGATGAG  600
Sbjct  603  .........A...........................................C......  662

Query  601  CAAGTGCTCGAGTCCAGAAACATTGGGCTATGTTCAGACAAAGTTTCTGAAGGGGGAAAG  660
Sbjct  663  ............................................................  722

Query  661  AAAGGGAGATCAATCCTCAGGAAGAAGTTCTTGAAGCAGTCAGACAGAGTCATGTTCTCT  720
Sbjct  723  ............................................................  782

Query  721  GTCGGCTCGACGTTGTATACGGAAAGCCGTAAATTACTGCAAAGTTGGCACCTGCCATCC  780
Sbjct  783  ............................................C...............  842

Query  781  ACATTCCATCTCAAAGGCAAATCTTCGTTCACGTGCCGCTGCGACACTATCGTCAGCTGC  840
Sbjct  843  ................................A..............C............  902

Query  841  GAAGGGTATGTTCTGAAGAAAATTACAATGTGTCCTGGAGTGACAGGCAAACCGATAGGA  900
Sbjct  903  ................................C..C.............T..........  962
```

```
Query  901   TATGCCGTCACCCATCACAAAGAAGGATTCGTAGTCGGAAAAGTCACAGATACCATTCGC  960
Sbjct  963   ....................G.............................C.........  1022

Query  961   GGCGAGAGAGTCTCCTTCGCCGTGTGTACTTATGTACCAACAACACTCTGCGACCAGATG  1020
Sbjct  1023  ............................................................  1082

Query  1021  ACCGGGATCCTAGCAACAGAAGTAACAGCCGATGATGCCCAGAAACTGCTGGTGGGTTTG  1080
Sbjct  1083  ........T...................................................  1142

Query  1081  AACCAGAGAATAGTAGTTAATGGTAGGACCCAGAGAAATACCAATACTATGAAGAACTAC  1140
Sbjct  1143  ............................................C...............  1202

Query  1141  CTGCTACCACTGGTTGCACAAGCGCTAGCAAAATGGGCGAAGGAAGCAAAACAGGATATG  1200
Sbjct  1203  ......................................A.....................  1262

Query  1201  GAAGATGAAAGACCCCTGAACGAACGCCAACGAACGCTAACGTGCCTCTGCTGCTGGGCA  1260
Sbjct  1263  ............................................................  1322

Query  1261  TTTAAGCGAAACAAACGCCACGCCATTTACAAGAGACCAGACACACAGAGTATAGTCAAG  1320
Sbjct  1323  ............................................................  1382

Query  1321  GTCCCTTGCGAATTCACAAGCTTTCCTTTGGTCAGCCTGTGGTCCGCTGGGATGTCTATA  1380
Sbjct  1383  ............................................................  1442

Query  1381  TCTCTTAGGCAGAAGTTGAAGATGATGCTGCAGGCGAGGCAGCCCACACAAATAGCAGCA  1440
Sbjct  1443  .....C......................................................  1502

Query  1441  GTGACTGAGGAACTCATACAAGAAGCAGCTGCAGTAGAGCAAGAGGCCGTGGATACGGCC  1500
Sbjct  1503  ..................................C.........................  1562

Query  1501  AATGCCGAGCTGGACCACGCCGCATGGCCCTCCATTGTGGATACGACAGAGCGCCATGTT  1560
Sbjct  1563  ...............................................G............  1622

Query  1561  GAGGTCGAAGTGGAAGAACTCGACCAGCGTGCAGGGGAAGGGGTAGTGGAAACACCTCGA  1620
Sbjct  1623  ............................................................  1682

Query  1621  AACTCTATCAAAGTTTCAACACAGATCGGGGACGCGTTAATCGGCAGTTACCTGATCCTA  1680
Sbjct  1683  ........T........T..........................................  1742

Query  1681  TCACCCCAAGCAGTCCTACGCAGCGAAAAATTAGCCTGCATACATGATCTTGCAGAGCAG  1740
Sbjct  1743  ............................................................  1802

Query  1741  GTTAAGTTGGTCACACACTCTGGCCGTAGTGGTAGGTACGCCGTCGACAAATACNACGGA  1800
Sbjct  1803  ..C.................C..T..G...........................G.....  1862

Query  1801  AGAGTACTAGTCCCTACAGGAGTGGCTATAGACATTCAATCGTTCCAGGCTCTCAGTGAG  1860
Sbjct  1863  ...................................C........................  1922

Query  1861  AGCGCGACCCTTGTGTACAACGAACGCGAGTTCGTTAACAGGAAGCTGTGGCACATAGCA  1920
Sbjct  1923  ..T..........................................T..............  1982

Query  1921  GTATACGGGGCAGCACTCAATACTGATGAAGAAGGATACGAGAAGGTCCCGGTAGAGAGA  1980
Sbjct  1983  ............................................................  2042
```

```
Query  1981  GCAGAATCAGATTATGTGTTTGATGTAGACCAAAAAATGTGCCTaaaaaaaGAGCAGGCA  2040
Sbjct  2043  .................A............A.............C...............  2102

Query  2041  TCAGGTTGGGTACTCTGTGGCGAACTAGTCAACCCCCCATTCCACGAATTCGCATATGAA  2100
Sbjct  2103  .....A......................................................  2162

Query  2101  GGGCTCCGCACGAGACCGTCAGCACCCTACAAGGTTCATACAGTAGGTGTGTACGGAGTG  2160
Sbjct  2163  ........T..T................................................  2222

Query  2161  CCAGGATCAGGCAAATCCGCAATAATCAAGAACACGGTCACCATGTCTGACCTAGTATTG  2220
Sbjct  2223  ............................................................  2282

Query  2221  AGTGGTAAGAAAGAGAACTGCTTAGAAATTATGAACGATGTACTTAAACACAGAGCTCTA  2280
Sbjct  2283  ............................................................  2342

Query  2281  CGTATCACAGCGAAGACCGTAGACTCAGTGTTATTAAACGGCGTGAAACACACGCCTAAC  2340
Sbjct  2343  .....T.............................G........................  2402

Query  2341  ATACTATACATCGACGAAGCGTTCTCATGCCATGCAGGGACTCTGTTGGCCACTATAGCC  2400
Sbjct  2403  ............................................................  2462

Query  2401  ATAGTCAGGCCCAAACAGAAAGTGGTACTGTGCGGAGACCCGAAACAATGCGGATTCTTC  2460
Sbjct  2463  T...........................................................  2522

Query  2461  AATATGATGCAACTGAAAGTTAATTACAATCATGACATCTGCTCAGAAGTCTTCCACAAA  2520
Sbjct  2523  .......................C....................................  2582

Query  2521  AGTATCTCTAGACGGTGCACCCAGGATATCACGGCCATCGTTTCCAAATTACATTACCAG  2580
Sbjct  2583  ............................................T...............  2642

Query  2581  GACCGAATGAGGACCACAAACCCCCGAAAAGGAGACATCATTATAGACACTACCGGCACT  2640
Sbjct  2643  ..................................................C.........  2702

Query  2641  ACCAAACCAGCCAAAACAGATCTGATTCTGACGTGCTTCAGGGGATGGGTGAAACAGTTG  2700
Sbjct  2703  ....................C.......................................  2762

Query  2701  CAGCAAGACTACAGAGGTAACGAAGTAATGACGGCTGCAGCGTCCCAAGGACTGACGAGG  2760
Sbjct  2763  ............................................................  2822

Query  2761  GCCTCCGTATATGCGGTTCGAACTAAAGTCAATGAGAACCCGCTATATGCACAGACCTCC  2820
Sbjct  2823  ......................................T.....................  2882

Query  2821  GAGCACGTGAACGTGTTGTTAACACGCACAGAAAACAAGCTAGTATGGAAGACCTTGTCA  2880
Sbjct  2883  .......................................T....................  2942

Query  2881  ACAGATCCCTGGATTAAAACACTGACTAACCCACCTAGAGGGCACTATACCGCCACCATA  2940
Sbjct  2943  ............................................................  3002

Query  2941  GCAGAATGGGAAGCGGAACACCAGGGTATAATGAAGGCCATACAAGGGTATGCACCGCCC  3000
Sbjct  3003  ..............A.............................................  3062

Query  3001  GTGAACACCTTCATGAACAAAGTAAATGTGTGCTGGGCAAAGACACTTACGCCTGTGCTG  3060
Sbjct  3063  ...........T................................G..C..A.........  3122
```

```
Query  3061  GAAACTGCGGGTATCTCCCTGTCAGCAGAAGACTGGTCTGAACTGCTGCCCCCGTTTGCC  3120
Sbjct  3123  ..................T.........................................  3182

Query  3121  CAGGACGTGGCGTACTCACCCGAGGTGGCATTAAACATCATATGCACGAAAATGTATGGG  3180
Sbjct  3183  ...............................................A............  3242

Query  3181  TTTGACTTAGACACTGGTCTTTTTCCAGGCCATCAGTGCCAATGACATACACCAAAGAC  3240
Sbjct  3243  ........G.....C.............A.........A.....................  3302

Query  3241  CATTGGGATAACAGAGTTGGAGGGAAAATGTATGGATTCAGCCAACAAGCATACGATCAG  3300
Sbjct  3303  ............................................................  3362

Query  3301  CTGGCAAGACGACATCCGTACCTTCGAGGTAGAGAGAAATCAGGAATGCAGATCGTAGTC  3360
Sbjct  3363  ...................................A........................  3422

Query  3361  ACTGAAATGCGTATCCAGCGCCCAAGATCGGATGCCAACATCATCCCGATCAACCGCAGG  3420
Sbjct  3423  .....G......................................................  3482

Query  3421  CTCCCTCACTCACTCGTAGCCACACACGAGTATAGGCGAGCTGCACGGGCCGAGGAATTC  3480
Sbjct  3483  ............................................G...............  3542

Query  3481  TTCACCACGACACGAGGGTACACTATGCTGCTGGTCTCTGAGTATAACATGAACTTACCA  3540
Sbjct  3543  ............................................................  3602

Query  3541  AACAAGAAGATCACCTGGCTGGCTCCGATAGGGACGCAGGGGGCCCATCACACCGCCAAC  3600
Sbjct  3603  ............................................................  3662

Query  3601  CTAAACTTGGGGATACCACCTCTGCTGGGCAGTTTTGATGCGGTGGTTGTGAACATGCCG  3660
Sbjct  3663  ........................................T...................  3722

Query  3661  ACTCCATTCCGGAACCATCACTACCAGCAATGTGAAGACCACGCGATGAAACTCCAGATG  3720
Sbjct  3723  ................................C...........................  3782

Query  3721  CTGGCAGGCGACGCACTGAGGCACATTAAACCTGGCGGATCATTGTGGGTCAAGGCATAC  3780
Sbjct  3783  .............................G..............................  3842

Query  3781  GGCTACGCAGACCGGCACAGCGAGCACGTGGTCTTGGCATTGGCTAGAAAGTTTAAAAGC  3840
Sbjct  3843  .......................................C....................  3902

Query  3841  TTCAGAGTCACACAACCCTCATGCGTGACTTCCAACACCGAGGTGTTTCTCCACTTCTCA  3900
Sbjct  3903  ...........G........G.....A................................G  3962

Query  3901  ATTTTTGACAATGGCAAACGCGCGATAGCCCTGCATTCAGCTAATAGGAAGGCTAACAGT  3960
Sbjct  3963  ............................................................  4022

Query  3961  ATCTTCCAAAACACCTTCTTACCGGCGGGCAGTGCACCGGCGTACAGAGTCAAACGTGGA  4020
Sbjct  4023  ..........................A....A............................  4082

Query  4021  GACATTTCGAACGCCCCAGAGGATGCAGTGGTCAATGCAGCAAACCAACAGGGAGTGAAG  4080
Sbjct  4083  ............................................................  4142

Query  4081  GGTGCTGGAGTTTGCGGTGCAATTTACCGTAAGTGGCCGGACGCTTTCGGTGATGTCGCT  4140
Sbjct  4143  ............................................................  4202
```

```
Query  4141  ACTCCAACCGGAACAGCAGTTTCGAAATCCGTCCAAGATAAATTGGTGATCCACGCTGTC  4200
Sbjct  4203  ............................................................  4262

Query  4201  GGCCCGAATTTCTCAAAATGTTCAGAAGAGGAAGGGGACAGAGACCTAGCATCTGCTTAC  4260
Sbjct  4263  ...........T................................................  4322

Query  4261  AGAGCTGCAGCAGAAATAGTGATGGATaaaaaaaTTACAACAGTGGCCGTCCCCTTACTC  4320
Sbjct  4323  ..........................G...........T...............C.....  4382

Query  4321  TCCACCGGCATTTATGCCGGAGGAAAAAACAGAGTAGAACAGTCACTCAACCATCTCTTC  4380
Sbjct  4383  ..............C........G....................................  4442

Query  4381  ACGGCATTCGACAATACTGATGCAGATGTGACCATATATTGCATGGACAAAACATGGGAA  4440
Sbjct  4443  ........T...................................................  4502

Query  4441  AAGAAGATTAAGGAGGCAATCGATCACCGGACTTCGGTTGAGATGGTGCAGGATGACGTG  4500
Sbjct  4503  ............................................................  4562

Query  4501  CAGTTGGAGGAGGAACTGGTACGAGTACACCCTTTGAGTAGTTTAGCAGGTAGGAAGGGT  4560
Sbjct  4563  ......................................C.................A...  4622

Query  4561  TACAGTACGGACAGCGGCCGAGTGTTTTCCTACCTGGAAGGTACCAAATTCCATCAGACT  4620
Sbjct  4623  ............................................................  4682

Query  4621  GCGGTGGACATAGCCGAAATGCAAGTGCTGTGGCCCGCCCTCAAAGAGTCTAATGAGCAA  4680
Sbjct  4683  ........T.............................T.....................  4742

Query  4681  ATAGTGGCATACACCTTAGGAGAATCAATGGACCAGATACGTGGCAAGTGCCCGACAGAA  4740
Sbjct  4743  ............................................................  4802

Query  4741  GATACTGACGCCTCCACACCTCCACGGACTGTGCCGTGCCTCTGTCGATACGCCATGACA  4800
Sbjct  4803  ............................................................  4862

Query  4801  CCAGAGAGAGTGTACCGACTTAAATGCACGAACACTACCCAATTTACGGTTTGCTCATCT  4860
Sbjct  4863  ............................................C.....C.........  4922

Query  4861  TTTGAGTTGCCAAAGTATCACATTCAGGGAGTGCAGAGAGTAAAATGTGAAAGAATCATC  4920
Sbjct  4923  ............................................................  4982

Query  4921  ATCTTAGATCCCACTGTTCCACCAACTTACAAACGGCCATGCATCAGACGGTACCCCTCC  4980
Sbjct  4983  ............................................................  5042

Query  4981  ACAATCTCTTGTAACTCCTCTGAGGACTCCAGGAGCTTGTCTACTTTTTCTGTCAGCTCC  5040
Sbjct  5043  ........C......................................C............  5102

Query  5041  GACTCCTCGATTGGTTCTCTGCCGGTCGGAGACACGAGACCCATTCCAGCCCCGAGGACC  5100
Sbjct  5103  .................CG..A...................T...........A......  5162

Query  5101  ATTTTCAGACCCGTCCCTGCCCCGAGAGCACCCGTGCTCAGAACCACACCGCCTCCTAAA  5160
Sbjct  5163  G...............................T..........T................  5222

Query  5161  CCACCGCGCACATTCACCGTGCGTGCAGAAGTGCACCAAGCACCCCCTACACCTGTACCT  5220
Sbjct  5223  ...................................T........................  5282
```

```
Query  5221  CCACCCAGACCGAAGAGGGCTGCAAAGTTGGCTCGTGAGATGCACCCCGGGTTCACCTTC  5280
Sbjct  5283  .....T......................................................  5342

Query  5281  GGGGACTTCGGAGAGCACGAGGTTGAGGAGCTTACGGCCTCTCCCTTAACCTTCGGAGAT  5340
Sbjct  5343  ..A..................A.................G....................  5402

Query  5341  TTTGCTGAAGGAGAGATCCAGGGGATGGGAGTGGAGTTTGAATGACTAGGCAGAGCCGGC  5400
Sbjct  5403  ....................A....C..................................  5462

Query  5401  GGGTACATTTTTTCGTCAGACACGGGTCCAGGCCACCTACAGCAGAGATCCGTTTTACAA  5460
Sbjct  5463  .........................................A.....G............  5522

Query  5461  AATTGCACGGCAGAATGTATCTACGAACCGGCAAAACTAGAAAAAATTCATGCACCAAAG  5520
Sbjct  5523  ............................................................  5582

Query  5521  TTGGATAAAACCAAGGAAGATATCTTAAGGAGCAAGTACCAAATGAAACCGTCTGAAGCA  5580
Sbjct  5583  ............................................................  5642

Query  5581  AACAAAAGCAGGTACCAATCTAGAAAAGTAGAAAATATGAAAGCAGAGATCGTAGGTAGA  5640
Sbjct  5643  ........T.........................................T.........  5702

Query  5641  CTCTTGGACGGACTGGGGGAGTATCTGGGCACCGAGCATCCAGTTGAATGCTACCGAATA  5700
Sbjct  5703  ..............A.............................................  5762

Query  5701  ACGTACCCGGTGCCTATATACTCAACTAGTGACCTCAGAGGTCTGTCTAGTGCCAAAACA  5760
Sbjct  5763  .....T....C...................AT..G.........................  5822

Query  5761  GCTGTTAGAGCTTGCAATGCATTTTTGGAAGCTAATTTTCCATCAGTCACTTCATATAAA  5820
Sbjct  5823  ............................................................  5882

Query  5821  ATTACTGATGAATACGACGCATACCTAGATATGGTAGATGGATCAGAGAGCTGTCTGGAC  5880
Sbjct  5883  ............................................................  5942

Query  5881  AGATCCTCCTTTTCGCCGTCTAGATTGCGTAGCTTTCCAAAAACACACTCATACTTGGAC  5940
Sbjct  5943  .................A...........C..............................  6002

Query  5941  CCACAGATCAACAGTGCGGTACCGTCACCATTCCAAAACACCTTACAAAATGTATTGGCA  6000
Sbjct  6003  ..G.........................................................  6062

Query  6001  GCGGCCACCAAAAGAAACTGTAATGTCACACAGATGAGAGAACTACCAACATATGATTCT  6060
Sbjct  6063  ...............................................G............  6122

Query  6061  GCAGTGCTAAATGTAGAGGCCTTCAGGAAATATGCGTGCAAGCCAGACGTATGGGATGAG  6120
Sbjct  6123  ............................................................  6182

Query  6121  TACAGGGATAATCCGATTTGCATAACCACCGAAAATGTCACCACTTACGTCGCCAAGTTG  6180
Sbjct  6183  ..............A...................................T..T......  6242

Query  6181  AAAGGACCGAAAGCTGCGGCCTTGTTTGCAAAAACACATAACCTGATACCACTACACCAA  6240
Sbjct  6243  ............................................................  6302

Query  6241  GTTCCTATGGACAAATTCACGGTAGATATGAAGAGAGATGTCAAAGTCACGCCCGGAACC  6300
Sbjct  6303  ............................................................  6362
```

```
Query  6301  AAGCACACCGAAGAGAGACCAAAGGTACAGGTGATTCAAGCGGCAGAGCCACTAGCCACT  6360
Sbjct  6363  ............................................................  6422

Query  6361  GCCTACCTCTGCGGAATTCACCGTGAATTGGTGCGCCGTCTCAACAACGCGCTTTTCCCA  6420
Sbjct  6423  .....................................................C.....T  6482

Query  6421  AACATCCACACTTTGTTTGATATGTCCGCAGAGGATTTCGATGCAATCATAGCGGAACAT  6480
Sbjct  6483  ..T...................................T.....................  6542

Query  6481  TTTAAGCACGGTGACCATGTGTTGGAAACGGATATAGCCTCTTTTGACAAAAGTCAAGAT  6540
Sbjct  6543  .................C..........................................  6602

Query  6541  GATTCCATGGCACTCACTGCGTTAATGATCCTTGAGGACCTGGGAGTAGACCAAAACCTA  6600
Sbjct  6603  ............................................................  6662

Query  6601  ATGAATTTGATAGAGGCTGCATTCGGGGAAATCGTGAGTACACACTTGCCCACAGGTACT  6660
Sbjct  6663  ............................................................  6722

Query  6661  AGATTCAAATTTGGAGCTATGATGAAGTCTGGAATGTTTTTGACGCTGTTCGTCAATACA  6720
Sbjct  6723  .......G.....................C...............T..............  6782

Query  6721  ATTCTTAATGTGGTTATTGCGTGCCGAGTGTTGGAGGATCAATTGGCGCAGTCGCCGTGG  6780
Sbjct  6783  ........C..................................................C  6842

Query  6781  CCTGCTTTCATAGGAGATGACAACATAATCCATGGTATAATATCAGACAAATTGATGGCA  6840
Sbjct  6843  G..........................................................G  6902

Query  6841  GATAGATGTGCCACCTGGATGAACATGGAGGTCAAGATACTGGACTCTATAGTTGGAATA  6900
Sbjct  6903  ............................................T..C............  6962

Query  6901  CGGCCACCTTACTTCTGTGGAGGATTTATTGTATGTGACGATGTAACAGGTACAGCCTGC  6960
Sbjct  6963  ...........T................................................  7022

Query  6961  CGCGTCGCAGACCCACTGAAGAGATTGTTCAAGCTAGGTAAGCCATTGCCACTTGACGAT  7020
Sbjct  7023  ........................C...................................  7082

Query  7021  GGCCAAGATGAAGACAGAAGACGTGCATTACATGATGAAGTGAAAACCTGGTCGCGCGTA  7080
Sbjct  7083  .............................G..............................  7142

Query  7081  GGGCTGCGACACAGAGTGTGTGAAGCCATCGAAGACCGTTATGCCGTCCACTCATCAGAA  7140
Sbjct  7143  ..................................................T.........  7202

Query  7141  CTAGTTTTATTGGCACTGACTACTCTGTCTAAGAACTTGAAGTCCTTCAGAAACATAAGA  7200
Sbjct  7203  ............................................................  7262

Query  7201  GGGAAACCAATACATCTCTACGGTGGTCCTAAATAG  7236
Sbjct  7263  ....................................  7298
```

>Barmah Forest virus isolate K80639, complete genome

Sequence ID: MN689030.1 Length: 11490

Range 1: 52 to 7287

```
Score:12595 bits(6820), Expect:0.0,
Identities:7097/7236(98%),  Gaps:0/7236(0%), Strand: Plus/Plus

Query  1     ATGGCGAAACCAGTTGTGAAGATCGACGTGGAACCTGAAAGCCATTTCGCTAAGCAGGTC  60
Sbjct  52    ...........G..............................T....T............  111

Query  61    CAGAGTTGCTTCCCGCAGTTTGAGATCGAAGCAGTGCAGACCACACCAAACGATCATGCA  120
Sbjct  112   .........................................G..................  171

Query  121   CACGCGAGGGCGTTTTCGCACCTTGCTACGAAGCTCATAGAAATGGAGACAGCAAAAGAT  180
Sbjct  172   ............................................................  231

Query  181   CAGATCATCCTCGATATCGGAAGTGCACCCGCGAGGAGACTGTATTCAGAACACAAGTAC  240
Sbjct  232   ............................................................  291

Query  241   CACTGTGTTTGCCCAATGAAGTGCACGGAAGATCCAGAGAGAATGCTAGGATATGCACGT  300
Sbjct  292   ............................................................  351

Query  301   AAGTTGATCGCAGGCTCTGCGAAAGGGAAGGCAGAAAAGTTACGCGATCTCAGGGATGTC  360
Sbjct  352   ............................................................  411

Query  361   TTGGCTACGCCAGACATCGAGACGCAGTCGCTATGTCTCCACACAGACGCATCCTGCAGA  420
Sbjct  412   ............................................................  471

Query  421   TACCGCGGTGATGTTGCCGTGTATCAAGACGTGTATGCCATTGACGCACCTACCACGCTG  480
Sbjct  472   ..................................................C.........  531

Query  481   TACCACCAAGCGTTAAAGGGCGTCAGGACCGCATATTGGATAGGCTTTGATACAACGCCG  540
Sbjct  532   ...........................................................A  591

Query  541   TTCATGTACGATGCACTAGCAGGAGCTTACCCGCTCTACTCCACAAACTGGGCTGATGAG  600
Sbjct  592   .........A...........................................C......  651

Query  601   CAAGTGCTCGAGTCCAGAAACATTGGGCTATGTTCAGACAAAGTTTCTGAAGGGGGAAAG  660
Sbjct  652   ............................................................  711

Query  661   AAAGGGAGATCAATCCTCAGGAAGAAGTTCTTGAAGCAGTCAGACAGAGTCATGTTCTCT  720
Sbjct  712   ............................................................  771

Query  721   GTCGGCTCGACGTTGTATACGGAAAGCCGTAAATTACTGCAAAGTTGGCACCTGCCATCC  780
Sbjct  772   ............................................C...............  831

Query  781   ACATTCCATCTCAAAGGCAAATCTTCGTTCACGTGCCGCTGCGACACTATCGTCAGCTGC  840
Sbjct  832   ................................A..............C............  891

Query  841   GAAGGGTATGTTCTGAAGAAAATTACAATGTGTCCTGGAGTGACAGGCAAACCGATAGGA  900
Sbjct  892   .................A..............C..C........................  951

Query  901   TATGCCGTCACCCATCACAAAGAAGGATTCGTAGTCGGAAAAGTCACAGATACCATTCGC  960
Sbjct  952   ....................G.............................C.........  1011

Query  961   GGCGAGAGAGTCTCCTTCGCCGTGTGTACTTATGTACCAACAACACTCTGCGACCAGATG  1020
Sbjct  1012  ............................................................  1071
```

```
Query  1021  ACCGGGATCCTAGCAACAGAAGTAACAGCCGATGATGCCCAGAAACTGCTGGTGGGTTTG  1080
Sbjct  1072  ........T...................................................  1131

Query  1081  AACCAGAGAATAGTAGTTAATGGTAGGACCCAGAGAAATACCAATACTATGAAGAACTAC  1140
Sbjct  1132  ............................................C...............  1191

Query  1141  CTGCTACCACTGGTTGCACAAGCGCTAGCAAAATGGGCGAAGGAAGCAAAACAGGATATG  1200
Sbjct  1192  ......................................A.....................  1251

Query  1201  GAAGATGAAAGACCCCTGAACGAACGCCAACGAACGCTAACGTGCCTCTGCTGCTGGGCA  1260
Sbjct  1252  ............................................................  1311

Query  1261  TTTAAGCGAAACAAACGCCACGCCATTTACAAGAGACCAGACACACAGAGTATAGTCAAG  1320
Sbjct  1312  ............................................................  1371

Query  1321  GTCCCTTGCGAATTCACAAGCTTTCCTTTGGTCAGCCTGTGGTCCGCTGGGATGTCTATA  1380
Sbjct  1372  ............................................................  1431

Query  1381  TCTCTTAGGCAGAAGTTGAAGATGATGCTGCAGGCGAGGCAGCCCACACAAATAGCAGCA  1440
Sbjct  1432  .............C..............................................  1491

Query  1441  GTGACTGAGGAACTCATACAAGAAGCAGCTGCAGTAGAGCAAGAGGCCGTGGATACGGCC  1500
Sbjct  1492  ..................................C.........................  1551

Query  1501  AATGCCGAGCTGGACCACGCCGCATGGCCCTCCATTGTGGATACGACAGAGCGCCATGTT  1560
Sbjct  1552  ...............................................G............  1611

Query  1561  GAGGTCGAAGTGGAAGAACTCGACCAGCGTGCAGGGGAAGGGGTAGTGGAAACACCTCGA  1620
Sbjct  1612  ............................................................  1671

Query  1621  AACTCTATCAAAGTTTCAACACAGATCGGGGACGCGTTAATCGGCAGTTACCTGATCCTA  1680
Sbjct  1672  ........T........T..........................................  1731

Query  1681  TCACCCCAAGCAGTCCTACGCAGCGAAAAATTAGCCTGCATACATGATCTTGCAGAGCAG  1740
Sbjct  1732  ............................................................  1791

Query  1741  GTTAAGTTGGTCACACACTCTGGCCGTAGTGGTAGGTACGCCGTCGACAAATACNACGGA  1800
Sbjct  1792  ..C.................C..T..G...........................G.....  1851

Query  1801  AGAGTACTAGTCCCTACAGGAGTGGCTATAGACATTCAATCGTTCCAGGCTCTCAGTGAG  1860
Sbjct  1852  ...................................C........................  1911

Query  1861  AGCGCGACCCTTGTGTACAACGAACGCGAGTTCGTTAACAGGAAGCTGTGGCACATAGCA  1920
Sbjct  1912  ..T..........................................T..............  1971

Query  1921  GTATACGGGGCAGCACTCAATACTGATGAAGAAGGATACGAGAAGGTCCCGGTAGAGAGA  1980
Sbjct  1972  ............................................................  2031

Query  1981  GCAGAATCAGATTATGTGTTTGATGTAGACCAAAAAATGTGCCTaaaaaaaGAGCAGGCA  2040
Sbjct  2032  .................A............A.............C...............  2091

Query  2041  TCAGGTTGGGTACTCTGTGGCGAACTAGTCAACCCCCCATTCCACGAATTCGCATATGAA  2100
Sbjct  2092  .....A......................................................  2151
```

```
Query  2101  GGGCTCCGCACGAGACCGTCAGCACCCTACAAGGTTCATACAGTAGGTGTGTACGGAGTG  2160
Sbjct  2152  ........T..T................................................  2211

Query  2161  CCAGGATCAGGCAAATCCGCAATAATCAAGAACACGGTCACCATGTCTGACCTAGTATTG  2220
Sbjct  2212  ............................................................  2271

Query  2221  AGTGGTAAGAAAGAGAACTGCTTAGAAATTATGAACGATGTACTTAAACACAGAGCTCTA  2280
Sbjct  2272  ............................................................  2331

Query  2281  CGTATCACAGCGAAGACCGTAGACTCAGTGTTATTAAACGGCGTGAAACACACGCCTAAC  2340
Sbjct  2332  .....T.............................G........................  2391

Query  2341  ATACTATACATCGACGAAGCGTTCTCATGCCATGCAGGGACTCTGTTGGCCACTATAGCC  2400
Sbjct  2392  ............................................................  2451

Query  2401  ATAGTCAGGCCCAAACAGAAAGTGGTACTGTGCGGAGACCCGAAACAATGCGGATTCTTC  2460
Sbjct  2452  T...........................................................  2511

Query  2461  AATATGATGCAACTGAAAGTTAATTACAATCATGACATCTGCTCAGAAGTCTTCCACAAA  2520
Sbjct  2512  .......................C....................................  2571

Query  2521  AGTATCTCTAGACGGTGCACCCAGGATATCACGGCCATCGTTTCCAAATTACATTACCAG  2580
Sbjct  2572  ............................................T...............  2631

Query  2581  GACCGAATGAGGACCACAAACCCCCGAAAAGGAGACATCATTATAGACACTACCGGCACT  2640
Sbjct  2632  ..................................................C.........  2691

Query  2641  ACCAAACCAGCCAAAACAGATCTGATTCTGACGTGCTTCAGGGGATGGGTGAAACAGTTG  2700
Sbjct  2692  ....................C.................T.....................  2751

Query  2701  CAGCAAGACTACAGAGGTAACGAAGTAATGACGGCTGCAGCGTCCCAAGGACTGACGAGG  2760
Sbjct  2752  ............................................................  2811

Query  2761  GCCTCCGTATATGCGGTTCGAACTAAAGTCAATGAGAACCCGCTATATGCACAGACCTCC  2820
Sbjct  2812  ............................................................  2871

Query  2821  GAGCACGTGAACGTGTTGTTAACACGCACAGAAAACAAGCTAGTATGGAAGACCTTGTCA  2880
Sbjct  2872  .......................................T....................  2931

Query  2881  ACAGATCCCTGGATTAAAACACTGACTAACCCACCTAGAGGGCACTATACCGCCACCATA  2940
Sbjct  2932  ............................................................  2991

Query  2941  GCAGAATGGGAAGCGGAACACCAGGGTATAATGAAGGCCATACAAGGGTATGCACCGCCC  3000
Sbjct  2992  ..............A.............................................  3051

Query  3001  GTGAACACCTTCATGAACAAAGTAAATGTGTGCTGGGCAAAGACACTTACGCCTGTGCTG  3060
Sbjct  3052  ...........T................................G..C..A.........  3111

Query  3061  GAAACTGCGGGTATCTCCCTGTCAGCAGAAGACTGGTCTGAACTGCTGCCCCCGTTTGCC  3120
Sbjct  3112  ..................T.........................................  3171

Query  3121  CAGGACGTGGCGTACTCACCCGAGGTGGCATTAAACATCATATGCACGAAAATGTATGGG  3180
Sbjct  3172  ...............................................A............  3231
```

```
Query  3181  TTTGACTTAGACACTGGTCTTTTTTCCAGGCCATCAGTGCCAATGACATACACCAAAGAC  3240
Sbjct  3232  ........G.....C.............A.........A.....................  3291

Query  3241  CATTGGGATAACAGAGTTGGAGGGAAAATGTATGGATTCAGCCAACAAGCATACGATCAG  3300
Sbjct  3292  ............................................................  3351

Query  3301  CTGGCAAGACGACATCCGTACCTTCGAGGTAGAGAGAAATCAGGAATGCAGATCGTAGTC  3360
Sbjct  3352  ...................................A........................  3411

Query  3361  ACTGAAATGCGTATCCAGCGCCCAAGATCGGATGCCAACATCATCCCGATCAACCGCAGG  3420
Sbjct  3412  .....G......................................................  3471

Query  3421  CTCCCTCACTCACTCGTAGCCACACACGAGTATAGGCGAGCTGCACGGGCCGAGGAATTC  3480
Sbjct  3472  ............................................G...............  3531

Query  3481  TTCACCACGACACGAGGGTACACTATGCTGCTGGTCTCTGAGTATAACATGAACTTACCA  3540
Sbjct  3532  ..........T.................................................  3591

Query  3541  AACAAGAAGATCACCTGGCTGGCTCCGATAGGGACGCAGGGGGCCCATCACACCGCCAAC  3600
Sbjct  3592  ............................................................  3651

Query  3601  CTAAACTTGGGGATACCACCTCTGCTGGGCAGTTTTGATGCGGTGGTTGTGAACATGCCG  3660
Sbjct  3652  ........................................T...................  3711

Query  3661  ACTCCATTCCGGAACCATCACTACCAGCAATGTGAAGACCACGCGATGAAACTCCAGATG  3720
Sbjct  3712  ................................C...........................  3771

Query  3721  CTGGCAGGCGACGCACTGAGGCACATTAAACCTGGCGGATCATTGTGGGTCAAGGCATAC  3780
Sbjct  3772  .............................G..............................  3831

Query  3781  GGCTACGCAGACCGGCACAGCGAGCACGTGGTCTTGGCATTGGCTAGAAAGTTTAAAAGC  3840
Sbjct  3832  .......................................C....................  3891

Query  3841  TTCAGAGTCACACAACCCTCATGCGTGACTTCCAACACCGAGGTGTTTCTCCACTTCTCA  3900
Sbjct  3892  ...........G........G.....A................................G  3951

Query  3901  ATTTTTGACAATGGCAAACGCGCGATAGCCCTGCATTCAGCTAATAGGAAGGCTAACAGT  3960
Sbjct  3952  ............................................................  4011

Query  3961  ATCTTCCAAAACACCTTCTTACCGGCGGGCAGTGCACCGGCGTACAGAGTCAAACGTGGA  4020
Sbjct  4012  ..........................A....A............................  4071

Query  4021  GACATTTCGAACGCCCCAGAGGATGCAGTGGTCAATGCAGCAAACCAACAGGGAGTGAAG  4080
Sbjct  4072  ............................................................  4131

Query  4081  GGTGCTGGAGTTTGCGGTGCAATTTACCGTAAGTGGCCGGACGCTTTCGGTGATGTCGCT  4140
Sbjct  4132  ............................................................  4191

Query  4141  ACTCCAACCGGAACAGCAGTTTCGAAATCCGTCCAAGATAAATTGGTGATCCACGCTGTC  4200
Sbjct  4192  ............................................................  4251

Query  4201  GGCCCGAATTTCTCAAAATGTTCAGAAGAGGAAGGGGACAGAGACCTAGCATCTGCTTAC  4260
Sbjct  4252  ...........T................................................  4311
```

```
Query  4261  AGAGCTGCAGCAGAAATAGTGATGGATaaaaaaaTTACAACAGTGGCCGTCCCCTTACTC  4320
Sbjct  4312  ..........................A...........................C.....  4371

Query  4321  TCCACCGGCATTTATGCCGGAGGAAAAAACAGAGTAGAACAGTCACTCAACCATCTCTTC  4380
Sbjct  4372  ..............C........G....................................  4431

Query  4381  ACGGCATTCGACAATACTGATGCAGATGTGACCATATATTGCATGGACAAAACATGGGAA  4440
Sbjct  4432  ........T..............G....................................  4491

Query  4441  AAGAAGATTAAGGAGGCAATCGATCACCGGACTTCGGTTGAGATGGTGCAGGATGACGTG  4500
Sbjct  4492  ............................................................  4551

Query  4501  CAGTTGGAGGAGGAACTGGTACGAGTACACCCTTTGAGTAGTTTAGCAGGTAGGAAGGGT  4560
Sbjct  4552  ......................................C.................A...  4611

Query  4561  TACAGTACGGACAGCGGCCGAGTGTTTTCCTACCTGGAAGGTACCAAATTCCATCAGACT  4620
Sbjct  4612  ............................................................  4671

Query  4621  GCGGTGGACATAGCCGAAATGCAAGTGCTGTGGCCCGCCCTCAAAGAGTCTAATGAGCAA  4680
Sbjct  4672  ........T..........................A..T.....................  4731

Query  4681  ATAGTGGCATACACCTTAGGAGAATCAATGGACCAGATACGTGGCAAGTGCCCGACAGAA  4740
Sbjct  4732  ............................................................  4791

Query  4741  GATACTGACGCCTCCACACCTCCACGGACTGTGCCGTGCCTCTGTCGATACGCCATGACA  4800
Sbjct  4792  ............................................................  4851

Query  4801  CCAGAGAGAGTGTACCGACTTAAATGCACGAACACTACCCAATTTACGGTTTGCTCATCT  4860
Sbjct  4852  ............................................C.....C.........  4911

Query  4861  TTTGAGTTGCCAAAGTATCACATTCAGGGAGTGCAGAGAGTAAAATGTGAAAGAATCATC  4920
Sbjct  4912  ............................................................  4971

Query  4921  ATCTTAGATCCCACTGTTCCACCAACTTACAAACGGCCATGCATCAGACGGTACCCCTCC  4980
Sbjct  4972  ............................................................  5031

Query  4981  ACAATCTCTTGTAACTCCTCTGAGGACTCCAGGAGCTTGTCTACTTTTTCTGTCAGCTCC  5040
Sbjct  5032  ........C......................................C............  5091

Query  5041  GACTCCTCGATTGGTTCTCTGCCGGTCGGAGACACGAGACCCATTCCAGCCCCGAGGACC  5100
Sbjct  5092  .................CG..A...................T.C.........A......  5151

Query  5101  ATTTTCAGACCCGTCCCTGCCCCGAGAGCACCCGTGCTCAGAACCACACCGCCTCCTAAA  5160
Sbjct  5152  G...............................T..........T................  5211

Query  5161  CCACCGCGCACATTCACCGTGCGTGCAGAAGTGCACCAAGCACCCCCTACACCTGTACCT  5220
Sbjct  5212  ...................................T........................  5271

Query  5221  CCACCCAGACCGAAGAGGGCTGCAAAGTTGGCTCGTGAGATGCACCCCGGGTTCACCTTC  5280
Sbjct  5272  .....T......................................................  5331

Query  5281  GGGGACTTCGGAGAGCACGAGGTTGAGGAGCTTACGGCCTCTCCCTTAACCTTCGGAGAT  5340
Sbjct  5332  ..A..................A.................G....................  5391
```

```
Query  5341  TTTGCTGAAGGAGAGATCCAGGGGATGGGAGTGGAGTTTGAATGACTAGGCAGAGCCGGC  5400
Sbjct  5392  ....................A....C..................................  5451

Query  5401  GGGTACATTTTTTCGTCAGACACGGGTCCAGGCCACCTACAGCAGAGATCCGTTTTACAA  5460
Sbjct  5452  .........................................A.....G............  5511

Query  5461  AATTGCACGGCAGAATGTATCTACGAACCGGCAAAACTAGAAAAAATTCATGCACCAAAG  5520
Sbjct  5512  ............................................................  5571

Query  5521  TTGGATAAAACCAAGGAAGATATCTTAAGGAGCAAGTACCAAATGAAACCGTCTGAAGCA  5580
Sbjct  5572  ............................................................  5631

Query  5581  AACAAAAGCAGGTACCAATCTAGAAAAGTAGAAAATATGAAAGCAGAGATCGTAGGTAGA  5640
Sbjct  5632  ........T.........................................T.........  5691

Query  5641  CTCTTGGACGGACTGGGGGAGTATCTGGGCACCGAGCATCCAGTTGAATGCTACCGAATA  5700
Sbjct  5692  ..............A.............................................  5751

Query  5701  ACGTACCCGGTGCCTATATACTCAACTAGTGACCTCAGAGGTCTGTCTAGTGCCAAAACA  5760
Sbjct  5752  .....T....C...................AT..G.........................  5811

Query  5761  GCTGTTAGAGCTTGCAATGCATTTTTGGAAGCTAATTTTCCATCAGTCACTTCATATAAA  5820
Sbjct  5812  ............................................................  5871

Query  5821  ATTACTGATGAATACGACGCATACCTAGATATGGTAGATGGATCAGAGAGCTGTCTGGAC  5880
Sbjct  5872  ............................................................  5931

Query  5881  AGATCCTCCTTTTCGCCGTCTAGATTGCGTAGCTTTCCAAAAACACACTCATACTTGGAC  5940
Sbjct  5932  .................A...........C..............................  5991

Query  5941  CCACAGATCAACAGTGCGGTACCGTCACCATTCCAAAACACCTTACAAAATGTATTGGCA  6000
Sbjct  5992  ..G.........................................................  6051

Query  6001  GCGGCCACCAAAAGAAACTGTAATGTCACACAGATGAGAGAACTACCAACATATGATTCT  6060
Sbjct  6052  ...............................................G............  6111

Query  6061  GCAGTGCTAAATGTAGAGGCCTTCAGGAAATATGCGTGCAAGCCAGACGTATGGGATGAG  6120
Sbjct  6112  ............................................................  6171

Query  6121  TACAGGGATAATCCGATTTGCATAACCACCGAAAATGTCACCACTTACGTCGCCAAGTTG  6180
Sbjct  6172  ..............A...................................T..T......  6231

Query  6181  AAAGGACCGAAAGCTGCGGCCTTGTTTGCAAAAACACATAACCTGATACCACTACACCAA  6240
Sbjct  6232  ............................................................  6291

Query  6241  GTTCCTATGGACAAATTCACGGTAGATATGAAGAGAGATGTCAAAGTCACGCCCGGAACC  6300
Sbjct  6292  ............................................................  6351

Query  6301  AAGCACACCGAAGAGAGACCAAAGGTACAGGTGATTCAAGCGGCAGAGCCACTAGCCACT  6360
Sbjct  6352  ............................................................  6411

Query  6361  GCCTACCTCTGCGGAATTCACCGTGAATTGGTGCGCCGTCTCAACAACGCGCTTTTCCCA  6420
Sbjct  6412  .....................................................C.....T  6471
```

```
Query  6421  AACATCCACACTTTGTTTGATATGTCCGCAGAGGATTTCGATGCAATCATAGCGGAACAT  6480
Sbjct  6472  ..T...................................T.....................  6531

Query  6481  TTTAAGCACGGTGACCATGTGTTGGAAACGGATATAGCCTCTTTTGACAAAAGTCAAGAT  6540
Sbjct  6532  .................C..........................................  6591

Query  6541  GATTCCATGGCACTCACTGCGTTAATGATCCTTGAGGACCTGGGAGTAGACCAAAACCTA  6600
Sbjct  6592  ............................................................  6651

Query  6601  ATGAATTTGATAGAGGCTGCATTCGGGGAAATCGTGAGTACACACTTGCCCACAGGTACT  6660
Sbjct  6652  ............................................................  6711

Query  6661  AGATTCAAATTTGGAGCTATGATGAAGTCTGGAATGTTTTTGACGCTGTTCGTCAATACA  6720
Sbjct  6712  .......G.....................C...............T..............  6771

Query  6721  ATTCTTAATGTGGTTATTGCGTGCCGAGTGTTGGAGGATCAATTGGCGCAGTCGCCGTGG  6780
Sbjct  6772  ........C..................................................C  6831

Query  6781  CCTGCTTTCATAGGAGATGACAACATAATCCATGGTATAATATCAGACAAATTGATGGCA  6840
Sbjct  6832  G..........................................................G  6891

Query  6841  GATAGATGTGCCACCTGGATGAACATGGAGGTCAAGATACTGGACTCTATAGTTGGAATA  6900
Sbjct  6892  ............................................T..C............  6951

Query  6901  CGGCCACCTTACTTCTGTGGAGGATTTATTGTATGTGACGATGTAACAGGTACAGCCTGC  6960
Sbjct  6952  ...........T................................................  7011

Query  6961  CGCGTCGCAGACCCACTGAAGAGATTGTTCAAGCTAGGTAAGCCATTGCCACTTGACGAT  7020
Sbjct  7012  ........................C...................................  7071

Query  7021  GGCCAAGATGAAGACAGAAGACGTGCATTACATGATGAAGTGAAAACCTGGTCGCGCGTA  7080
Sbjct  7072  .............................G..............................  7131

Query  7081  GGGCTGCGACACAGAGTGTGTGAAGCCATCGAAGACCGTTATGCCGTCCACTCATCAGAA  7140
Sbjct  7132  ..................................................T.........  7191

Query  7141  CTAGTTTTATTGGCACTGACTACTCTGTCTAAGAACTTGAAGTCCTTCAGAAACATAAGA  7200
Sbjct  7192  ............................................................  7251

Query  7201  GGGAAACCAATACATCTCTACGGTGGTCCTAAATAG  7236
Sbjct  7252  ....................................  7287
```

>Barmah Forest virus isolate ARBO304, complete genome
Sequence ID: MW556196.1 Length: 11526
Range 1: 42 to 7277

Score:12589 bits(6817), Expect:0.0,
Identities:7096/7236(98%), Gaps:0/7236(0%), Strand: Plus/Plus

```
Query  1     ATGGCGAAACCAGTTGTGAAGATCGACGTGGAACCTGAAAGCCATTTCGCTAAGCAGGTC  60
```

```
Sbjct  42     ...........G...................................T............  101

Query  61     CAGAGTTGCTTCCCGCAGTTTGAGATCGAAGCAGTGCAGACCACACCAAACGATCATGCA  120
Sbjct  102    .........................................G..................  161

Query  121    CACGCGAGGGCGTTTTCGCACCTTGCTACGAAGCTCATAGAAATGGAGACAGCAAAAGAT  180
Sbjct  162    ............................................................  221

Query  181    CAGATCATCCTCGATATCGGAAGTGCACCCGCGAGGAGACTGTATTCAGAACACAAGTAC  240
Sbjct  222    ............................................................  281

Query  241    CACTGTGTTTGCCCAATGAAGTGCACGGAAGATCCAGAGAGAATGCTAGGATATGCACGT  300
Sbjct  282    ............................................................  341

Query  301    AAGTTGATCGCAGGCTCTGCGAAAGGGAAGGCAGAAAAGTTACGCGATCTCAGGGATGTC  360
Sbjct  342    ............................................................  401

Query  361    TTGGCTACGCCAGACATCGAGACGCAGTCGCTATGTCTCCACACAGACGCATCCTGCAGA  420
Sbjct  402    ............................................................  461

Query  421    TACCGCGGTGATGTTGCCGTGTATCAAGACGTGTATGCCATTGACGCACCTACCACGCTG  480
Sbjct  462    ..................................................C.........  521

Query  481    TACCACCAAGCGTTAAAGGGCGTCAGGACCGCATATTGGATAGGCTTTGATACAACGCCG  540
Sbjct  522    ...........................................................A  581

Query  541    TTCATGTACGATGCACTAGCAGGAGCTTACCCGCTCTACTCCACAAACTGGGCTGATGAG  600
Sbjct  582    .........A...........................................C......  641

Query  601    CAAGTGCTCGAGTCCAGAAACATTGGGCTATGTTCAGACAAAGTTTCTGAAGGGGGAAAG  660
Sbjct  642    ............................................................  701

Query  661    AAAGGGAGATCAATCCTCAGGAAGAAGTTCTTGAAGCAGTCAGACAGAGTCATGTTCTCT  720
Sbjct  702    ............................................................  761

Query  721    GTCGGCTCGACGTTGTATACGGAAAGCCGTAAATTACTGCAAAGTTGGCACCTGCCATCC  780
Sbjct  762    ............................................C...............  821

Query  781    ACATTCCATCTCAAAGGCAAATCTTCGTTCACGTGCCGCTGCGACACTATCGTCAGCTGC  840
Sbjct  822    ................................A..............C............  881

Query  841    GAAGGGTATGTTCTGAAGAAAATTACAATGTGTCCTGGAGTGACAGGCAAACCGATAGGA  900
Sbjct  882    ................................C..C........................  941

Query  901    TATGCCGTCACCCATCACAAAGAAGGATTCGTAGTCGGAAAAGTCACAGATACCATTCGC  960
Sbjct  942    ..............C.....G.............................C.........  1001

Query  961    GGCGAGAGAGTCTCCTTCGCCGTGTGTACTTATGTACCAACAACACTCTGCGACCAGATG  1020
Sbjct  1002   ............................................................  1061

Query  1021   ACCGGGATCCTAGCAACAGAAGTAACAGCCGATGATGCCCAGAAACTGCTGGTGGGTTTG  1080
Sbjct  1062   ........T...................................................  1121

Query  1081   AACCAGAGAATAGTAGTTAATGGTAGGACCCAGAGAAATACCAATACTATGAAGAACTAC  1140
```

```
Sbjct  1122  ............................................C...............  1181

Query  1141  CTGCTACCACTGGTTGCACAAGCGCTAGCAAAATGGGCGAAGGAAGCAAAACAGGATATG  1200
Sbjct  1182  ......................................A.....................  1241

Query  1201  GAAGATGAAAGACCCCTGAACGAACGCCAACGAACGCTAACGTGCCTCTGCTGCTGGGCA  1260
Sbjct  1242  ............................................................  1301

Query  1261  TTTAAGCGAAACAAACGCCACGCCATTTACAAGAGACCAGACACACAGAGTATAGTCAAG  1320
Sbjct  1302  ............................................................  1361

Query  1321  GTCCCTTGCGAATTCACAAGCTTTCCTTTGGTCAGCCTGTGGTCCGCTGGGATGTCTATA  1380
Sbjct  1362  ............................................................  1421

Query  1381  TCTCTTAGGCAGAAGTTGAAGATGATGCTGCAGGCGAGGCAGCCCACACAAATAGCAGCA  1440
Sbjct  1422  ............................................................  1481

Query  1441  GTGACTGAGGAACTCATACAAGAAGCAGCTGCAGTAGAGCAAGAGGCCGTGGATACGGCC  1500
Sbjct  1482  ..................................C.........................  1541

Query  1501  AATGCCGAGCTGGACCACGCCGCATGGCCCTCCATTGTGGATACGACAGAGCGCCATGTT  1560
Sbjct  1542  ...............................................G............  1601

Query  1561  GAGGTCGAAGTGGAAGAACTCGACCAGCGTGCAGGGGAAGGGGTAGTGGAAACACCTCGA  1620
Sbjct  1602  ............................................................  1661

Query  1621  AACTCTATCAAAGTTTCAACACAGATCGGGGACGCGTTAATCGGCAGTTACCTGATCCTA  1680
Sbjct  1662  ........T........T..........................................  1721

Query  1681  TCACCCCAAGCAGTCCTACGCAGCGAAAAATTAGCCTGCATACATGATCTTGCAGAGCAG  1740
Sbjct  1722  ............................................................  1781

Query  1741  GTTAAGTTGGTCACACACTCTGGCCGTAGTGGTAGGTACGCCGTCGACAAATACNACGGA  1800
Sbjct  1782  ..C.................C..T..............................G.....  1841

Query  1801  AGAGTACTAGTCCCTACAGGAGTGGCTATAGACATTCAATCGTTCCAGGCTCTCAGTGAG  1860
Sbjct  1842  ...................................C........................  1901

Query  1861  AGCGCGACCCTTGTGTACAACGAACGCGAGTTCGTTAACAGGAAGCTGTGGCACATAGCA  1920
Sbjct  1902  ..T..........................................T..............  1961

Query  1921  GTATACGGGGCAGCACTCAATACTGATGAAGAAGGATACGAGAAGGTCCCGGTAGAGAGA  1980
Sbjct  1962  ............................................................  2021

Query  1981  GCAGAATCAGATTATGTGTTTGATGTAGACCAAAAAATGTGCCTaaaaaaaGAGCAGGCA  2040
Sbjct  2022  .................A............A.............C...............  2081

Query  2041  TCAGGTTGGGTACTCTGTGGCGAACTAGTCAACCCCCCATTCCACGAATTCGCATATGAA  2100
Sbjct  2082  .....A..................T...................................  2141

Query  2101  GGGCTCCGCACGAGACCGTCAGCACCCTACAAGGTTCATACAGTAGGTGTGTACGGAGTG  2160
Sbjct  2142  ........T..T......G.........................................  2201

Query  2161  CCAGGATCAGGCAAATCCGCAATAATCAAGAACACGGTCACCATGTCTGACCTAGTATTG  2220
```

```
Sbjct  2202  ............................................................  2261

Query  2221  AGTGGTAAGAAAGAGAACTGCTTAGAAATTATGAACGATGTACTTAAACACAGAGCTCTA  2280
Sbjct  2262  ............................................................  2321

Query  2281  CGTATCACAGCGAAGACCGTAGACTCAGTGTTATTAAACGGCGTGAAACACACGCCTAAC  2340
Sbjct  2322  .....T.............................G........................  2381

Query  2341  ATACTATACATCGACGAAGCGTTCTCATGCCATGCAGGGACTCTGTTGGCCACTATAGCC  2400
Sbjct  2382  ............................................................  2441

Query  2401  ATAGTCAGGCCCAAACAGAAAGTGGTACTGTGCGGAGACCCGAAACAATGCGGATTCTTC  2460
Sbjct  2442  T...........................................................  2501

Query  2461  AATATGATGCAACTGAAAGTTAATTACAATCATGACATCTGCTCAGAAGTCTTCCACAAA  2520
Sbjct  2502  .......................C....................................  2561

Query  2521  AGTATCTCTAGACGGTGCACCCAGGATATCACGGCCATCGTTTCCAAATTACATTACCAG  2580
Sbjct  2562  ............................................T...............  2621

Query  2581  GACCGAATGAGGACCACAAACCCCCGAAAAGGAGACATCATTATAGACACTACCGGCACT  2640
Sbjct  2622  ..................................................C.........  2681

Query  2641  ACCAAACCAGCCAAAACAGATCTGATTCTGACGTGCTTCAGGGGATGGGTGAAACAGTTG  2700
Sbjct  2682  ....................C.......................................  2741

Query  2701  CAGCAAGACTACAGAGGTAACGAAGTAATGACGGCTGCAGCGTCCCAAGGACTGACGAGG  2760
Sbjct  2742  ............................................................  2801

Query  2761  GCCTCCGTATATGCGGTTCGAACTAAAGTCAATGAGAACCCGCTATATGCACAGACCTCC  2820
Sbjct  2802  ............................................G...............  2861

Query  2821  GAGCACGTGAACGTGTTGTTAACACGCACAGAAAACAAGCTAGTATGGAAGACCTTGTCA  2880
Sbjct  2862  .......................................T....................  2921

Query  2881  ACAGATCCCTGGATTAAAACACTGACTAACCCACCTAGAGGGCACTATACCGCCACCATA  2940
Sbjct  2922  ............................................................  2981

Query  2941  GCAGAATGGGAAGCGGAACACCAGGGTATAATGAAGGCCATACAAGGGTATGCACCGCCC  3000
Sbjct  2982  ..............A...........C.................................  3041

Query  3001  GTGAACACCTTCATGAACAAAGTAAATGTGTGCTGGGCAAAGACACTTACGCCTGTGCTG  3060
Sbjct  3042  ...........T................................G..C..A.........  3101

Query  3061  GAAACTGCGGGTATCTCCCTGTCAGCAGAAGACTGGTCTGAACTGCTGCCCCCGTTTGCC  3120
Sbjct  3102  ..................T.........................................  3161

Query  3121  CAGGACGTGGCGTACTCACCCGAGGTGGCATTAAACATCATATGCACGAAAATGTATGGG  3180
Sbjct  3162  ...............................................A............  3221

Query  3181  TTTGACTTAGACACTGGTCTTTTTTCCAGGCCATCAGTGCCAATGACATACACCAAAGAC  3240
Sbjct  3222  ........G.....C.............A.........A.....................  3281

Query  3241  CATTGGGATAACAGAGTTGGAGGGAAAATGTATGGATTCAGCCAACAAGCATACGATCAG  3300
```

```
Sbjct  3282  ............................................................  3341

Query  3301  CTGGCAAGACGACATCCGTACCTTCGAGGTAGAGAGAAATCAGGAATGCAGATCGTAGTC  3360
Sbjct  3342  ...................................A........................  3401

Query  3361  ACTGAAATGCGTATCCAGCGCCCAAGATCGGATGCCAACATCATCCCGATCAACCGCAGG  3420
Sbjct  3402  .....G......................................................  3461

Query  3421  CTCCCTCACTCACTCGTAGCCACACACGAGTATAGGCGAGCTGCACGGGCCGAGGAATTC  3480
Sbjct  3462  ............................................G...............  3521

Query  3481  TTCACCACGACACGAGGGTACACTATGCTGCTGGTCTCTGAGTATAACATGAACTTACCA  3540
Sbjct  3522  ..........T.................................................  3581

Query  3541  AACAAGAAGATCACCTGGCTGGCTCCGATAGGGACGCAGGGGGCCCATCACACCGCCAAC  3600
Sbjct  3582  ............................................................  3641

Query  3601  CTAAACTTGGGGATACCACCTCTGCTGGGCAGTTTTGATGCGGTGGTTGTGAACATGCCG  3660
Sbjct  3642  ........................................T...................  3701

Query  3661  ACTCCATTCCGGAACCATCACTACCAGCAATGTGAAGACCACGCGATGAAACTCCAGATG  3720
Sbjct  3702  ................................C...........................  3761

Query  3721  CTGGCAGGCGACGCACTGAGGCACATTAAACCTGGCGGATCATTGTGGGTCAAGGCATAC  3780
Sbjct  3762  .............................G..............................  3821

Query  3781  GGCTACGCAGACCGGCACAGCGAGCACGTGGTCTTGGCATTGGCTAGAAAGTTTAAAAGC  3840
Sbjct  3822  .......................................C....................  3881

Query  3841  TTCAGAGTCACACAACCCTCATGCGTGACTTCCAACACCGAGGTGTTTCTCCACTTCTCA  3900
Sbjct  3882  ..T........G........G.....A................................G  3941

Query  3901  ATTTTTGACAATGGCAAACGCGCGATAGCCCTGCATTCAGCTAATAGGAAGGCTAACAGT  3960
Sbjct  3942  ............................................................  4001

Query  3961  ATCTTCCAAAACACCTTCTTACCGGCGGGCAGTGCACCGGCGTACAGAGTCAAACGTGGA  4020
Sbjct  4002  ................G.........A....A............................  4061

Query  4021  GACATTTCGAACGCCCCAGAGGATGCAGTGGTCAATGCAGCAAACCAACAGGGAGTGAAG  4080
Sbjct  4062  ............................................................  4121

Query  4081  GGTGCTGGAGTTTGCGGTGCAATTTACCGTAAGTGGCCGGACGCTTTCGGTGATGTCGCT  4140
Sbjct  4122  ............................................................  4181

Query  4141  ACTCCAACCGGAACAGCAGTTTCGAAATCCGTCCAAGATAAATTGGTGATCCACGCTGTC  4200
Sbjct  4182  ............................................................  4241

Query  4201  GGCCCGAATTTCTCAAAATGTTCAGAAGAGGAAGGGGACAGAGACCTAGCATCTGCTTAC  4260
Sbjct  4242  ...........T................................................  4301

Query  4261  AGAGCTGCAGCAGAAATAGTGATGGATaaaaaaaTTACAACAGTGGCCGTCCCCTTACTC  4320
Sbjct  4302  ..........................A...........................C.....  4361

Query  4321  TCCACCGGCATTTATGCCGGAGGAAAAAACAGAGTAGAACAGTCACTCAACCATCTCTTC  4380
```

```
Sbjct  4362  ..............C........G....................................  4421

Query  4381  ACGGCATTCGACAATACTGATGCAGATGTGACCATATATTGCATGGACAAAACATGGGAA  4440
Sbjct  4422  ........T...................................................  4481

Query  4441  AAGAAGATTAAGGAGGCAATCGATCACCGGACTTCGGTTGAGATGGTGCAGGATGACGTG  4500
Sbjct  4482  ............................................................  4541

Query  4501  CAGTTGGAGGAGGAACTGGTACGAGTACACCCTTTGAGTAGTTTAGCAGGTAGGAAGGGT  4560
Sbjct  4542  ......................................C.................A...  4601

Query  4561  TACAGTACGGACAGCGGCCGAGTGTTTTCCTACCTGGAAGGTACCAAATTCCATCAGACT  4620
Sbjct  4602  ............................................................  4661

Query  4621  GCGGTGGACATAGCCGAAATGCAAGTGCTGTGGCCCGCCCTCAAAGAGTCTAATGAGCAA  4680
Sbjct  4662  ........T.............................T.....................  4721

Query  4681  ATAGTGGCATACACCTTAGGAGAATCAATGGACCAGATACGTGGCAAGTGCCCGACAGAA  4740
Sbjct  4722  ............................................................  4781

Query  4741  GATACTGACGCCTCCACACCTCCACGGACTGTGCCGTGCCTCTGTCGATACGCCATGACA  4800
Sbjct  4782  ............................................................  4841

Query  4801  CCAGAGAGAGTGTACCGACTTAAATGCACGAACACTACCCAATTTACGGTTTGCTCATCT  4860
Sbjct  4842  ............................................C.....C.........  4901

Query  4861  TTTGAGTTGCCAAAGTATCACATTCAGGGAGTGCAGAGAGTAAAATGTGAAAGAATCATC  4920
Sbjct  4902  ............................................................  4961

Query  4921  ATCTTAGATCCCACTGTTCCACCAACTTACAAACGGCCATGCATCAGACGGTACCCCTCC  4980
Sbjct  4962  ............................................................  5021

Query  4981  ACAATCTCTTGTAACTCCTCTGAGGACTCCAGGAGCTTGTCTACTTTTTCTGTCAGCTCC  5040
Sbjct  5022  ........C......................................C............  5081

Query  5041  GACTCCTCGATTGGTTCTCTGCCGGTCGGAGACACGAGACCCATTCCAGCCCCGAGGACC  5100
Sbjct  5082  .................CG..A...................T...........A......  5141

Query  5101  ATTTTCAGACCCGTCCCTGCCCCGAGAGCACCCGTGCTCAGAACCACACCGCCTCCTAAA  5160
Sbjct  5142  G...............................T..........T................  5201

Query  5161  CCACCGCGCACATTCACCGTGCGTGCAGAAGTGCACCAAGCACCCCCTACACCTGTACCT  5220
Sbjct  5202  ...................................T........................  5261

Query  5221  CCACCCAGACCGAAGAGGGCTGCAAAGTTGGCTCGTGAGATGCACCCCGGGTTCACCTTC  5280
Sbjct  5262  .....T......................................................  5321

Query  5281  GGGGACTTCGGAGAGCACGAGGTTGAGGAGCTTACGGCCTCTCCCTTAACCTTCGGAGAT  5340
Sbjct  5322  ..A..................A.................G....................  5381

Query  5341  TTTGCTGAAGGAGAGATCCAGGGGATGGGAGTGGAGTTTGAATGACTAGGCAGAGCCGGC  5400
Sbjct  5382  ....................A....C..................................  5441

Query  5401  GGGTACATTTTTTCGTCAGACACGGGTCCAGGCCACCTACAGCAGAGATCCGTTTTACAA  5460
```

```
Sbjct  5442  ........................................A.....G............  5501

Query  5461  AATTGCACGGCAGAATGTATCTACGAACCGGCAAAACTAGAAAAAATTCATGCACCAAAG  5520
Sbjct  5502  ............................................................  5561

Query  5521  TTGGATAAAACCAAGGAAGATATCTTAAGGAGCAAGTACCAAATGAAACCGTCTGAAGCA  5580
Sbjct  5562  ............................................................  5621

Query  5581  AACAAAAGCAGGTACCAATCTAGAAAAGTAGAAAATATGAAAGCAGAGATCGTAGGTAGA  5640
Sbjct  5622  ........T.........................................T.........  5681

Query  5641  CTCTTGGACGGACTGGGGGAGTATCTGGGCACCGAGCATCCAGTTGAATGCTACCGAATA  5700
Sbjct  5682  ..............A.............................................  5741

Query  5701  ACGTACCCGGTGCCTATATACTCAACTAGTGACCTCAGAGGTCTGTCTAGTGCCAAAACA  5760
Sbjct  5742  .....T....C...................AT..G.........................  5801

Query  5761  GCTGTTAGAGCTTGCAATGCATTTTTGGAAGCTAATTTTCCATCAGTCACTTCATATAAA  5820
Sbjct  5802  ............................................................  5861

Query  5821  ATTACTGATGAATACGACGCATACCTAGATATGGTAGATGGATCAGAGAGCTGTCTGGAC  5880
Sbjct  5862  ............................................................  5921

Query  5881  AGATCCTCCTTTTCGCCGTCTAGATTGCGTAGCTTTCCAAAAACACACTCATACTTGGAC  5940
Sbjct  5922  .................A...........C..............................  5981

Query  5941  CCACAGATCAACAGTGCGGTACCGTCACCATTCCAAAACACCTTACAAAATGTATTGGCA  6000
Sbjct  5982  ..G.........................................................  6041

Query  6001  GCGGCCACCAAAAGAAACTGTAATGTCACACAGATGAGAGAACTACCAACATATGATTCT  6060
Sbjct  6042  ................................................G...........  6101

Query  6061  GCAGTGCTAAATGTAGAGGCCTTCAGGAAATATGCGTGCAAGCCAGACGTATGGGATGAG  6120
Sbjct  6102  ............................................................  6161

Query  6121  TACAGGGATAATCCGATTTGCATAACCACCGAAAATGTCACCACTTACGTCGCCAAGTTG  6180
Sbjct  6162  ..............A...................................T..T......  6221

Query  6181  AAAGGACCGAAAGCTGCGGCCTTGTTTGCAAAAACACATAACCTGATACCACTACACCAA  6240
Sbjct  6222  ............................................................  6281

Query  6241  GTTCCTATGGACAAATTCACGGTAGATATGAAGAGAGATGTCAAAGTCACGCCCGGAACC  6300
Sbjct  6282  ............................................................  6341

Query  6301  AAGCACACCGAAGAGAGACCAAAGGTACAGGTGATTCAAGCGGCAGAGCCACTAGCCACT  6360
Sbjct  6342  ............................................................  6401

Query  6361  GCCTACCTCTGCGGAATTCACCGTGAATTGGTGCGCCGTCTCAACAACGCGCTTTTCCCA  6420
Sbjct  6402  ...................................................C.....T  6461

Query  6421  AACATCCACACTTTGTTTGATATGTCCGCAGAGGATTTCGATGCAATCATAGCGGAACAT  6480
Sbjct  6462  ..T.................................T.....................  6521

Query  6481  TTTAAGCACGGTGACCATGTGTTGGAAACGGATATAGCCTCTTTTGACAAAAGTCAAGAT  6540
```

```
Sbjct  6522  .................C..........................................  6581

Query  6541  GATTCCATGGCACTCACTGCGTTAATGATCCTTGAGGACCTGGGAGTAGACCAAAACCTA  6600
Sbjct  6582  ............................................................  6641

Query  6601  ATGAATTTGATAGAGGCTGCATTCGGGGAAATCGTGAGTACACACTTGCCCACAGGTACT  6660
Sbjct  6642  ............................................................  6701

Query  6661  AGATTCAAATTTGGAGCTATGATGAAGTCTGGAATGTTTTTGACGCTGTTCGTCAATACA  6720
Sbjct  6702  .......G.....................C...............T..............  6761

Query  6721  ATTCTTAATGTGGTTATTGCGTGCCGAGTGTTGGAGGATCAATTGGCGCAGTCGCCGTGG  6780
Sbjct  6762  ........C..................................................C  6821

Query  6781  CCTGCTTTCATAGGAGATGACAACATAATCCATGGTATAATATCAGACAAATTGATGGCA  6840
Sbjct  6822  G...............................C..........................G  6881

Query  6841  GATAGATGTGCCACCTGGATGAACATGGAGGTCAAGATACTGGACTCTATAGTTGGAATA  6900
Sbjct  6882  ............................................T..C............  6941

Query  6901  CGGCCACCTTACTTCTGTGGAGGATTTATTGTATGTGACGATGTAACAGGTACAGCCTGC  6960
Sbjct  6942  ...........T................................................  7001

Query  6961  CGCGTCGCAGACCCACTGAAGAGATTGTTCAAGCTAGGTAAGCCATTGCCACTTGACGAT  7020
Sbjct  7002  ........................C...................................  7061

Query  7021  GGCCAAGATGAAGACAGAAGACGTGCATTACATGATGAAGTGAAAACCTGGTCGCGCGTA  7080
Sbjct  7062  .............................G..............................  7121

Query  7081  GGGCTGCGACACAGAGTGTGTGAAGCCATCGAAGACCGTTATGCCGTCCACTCATCAGAA  7140
Sbjct  7122  ..................................................T.........  7181

Query  7141  CTAGTTTTATTGGCACTGACTACTCTGTCTAAGAACTTGAAGTCCTTCAGAAACATAAGA  7200
Sbjct  7182  ..............G.............................................  7241

Query  7201  GGGAAACCAATACATCTCTACGGTGGTCCTAAATAG  7236
Sbjct  7242  ....................................  7277
```

>Barmah Forest virus isolate SW105045, complete genome
Sequence ID: MN689046.1 Length: 11486
Range 1: 54 to 7289

Score:12584 bits(6814), Expect:0.0,
Identities:7095/7236(98%),  Gaps:0/7236(0%), Strand: Plus/Plus

```
Query  1     ATGGCGAAACCAGTTGTGAAGATCGACGTGGAACCTGAAAGCCATTTCGCTAAGCAGGTC  60
Sbjct  54    ...........G...................................T............  113

Query  61    CAGAGTTGCTTCCCGCAGTTTGAGATCGAAGCAGTGCAGACCACACCAAACGATCATGCA  120
Sbjct  114   .........................................G..................  173
```

```
Query  121   CACGCGAGGGCGTTTTCGCACCTTGCTACGAAGCTCATAGAAATGGAGACAGCAAAAGAT  180
Sbjct  174   ............................................................  233

Query  181   CAGATCATCCTCGATATCGGAAGTGCACCCGCGAGGAGACTGTATTCAGAACACAAGTAC  240
Sbjct  234   ............................................................  293

Query  241   CACTGTGTTTGCCCAATGAAGTGCACGGAAGATCCAGAGAGAATGCTAGGATATGCACGT  300
Sbjct  294   ............................................................  353

Query  301   AAGTTGATCGCAGGCTCTGCGAAAGGGAAGGCAGAAAAGTTACGCGATCTCAGGGATGTC  360
Sbjct  354   ............................................................  413

Query  361   TTGGCTACGCCAGACATCGAGACGCAGTCGCTATGTCTCCACACAGACGCATCCTGCAGA  420
Sbjct  414   ............................................................  473

Query  421   TACCGCGGTGATGTTGCCGTGTATCAAGACGTGTATGCCATTGACGCACCTACCACGCTG  480
Sbjct  474   ..................................................C.........  533

Query  481   TACCACCAAGCGTTAAAGGGCGTCAGGACCGCATATTGGATAGGCTTTGATACAACGCCG  540
Sbjct  534   ...........................................................A  593

Query  541   TTCATGTACGATGCACTAGCAGGAGCTTACCCGCTCTACTCCACAAACTGGGCTGATGAG  600
Sbjct  594   .........A...........................................C......  653

Query  601   CAAGTGCTCGAGTCCAGAAACATTGGGCTATGTTCAGACAAAGTTTCTGAAGGGGGAAAG  660
Sbjct  654   ............................................................  713

Query  661   AAAGGGAGATCAATCCTCAGGAAGAAGTTCTTGAAGCAGTCAGACAGAGTCATGTTCTCT  720
Sbjct  714   ............................................................  773

Query  721   GTCGGCTCGACGTTGTATACGGAAAGCCGTAAATTACTGCAAAGTTGGCACCTGCCATCC  780
Sbjct  774   ............................................C...............  833

Query  781   ACATTCCATCTCAAAGGCAAATCTTCGTTCACGTGCCGCTGCGACACTATCGTCAGCTGC  840
Sbjct  834   ................................A..............C............  893

Query  841   GAAGGGTATGTTCTGAAGAAAATTACAATGTGTCCTGGAGTGACAGGCAAACCGATAGGA  900
Sbjct  894   ................................C..C........................  953

Query  901   TATGCCGTCACCCATCACAAAGAAGGATTCGTAGTCGGAAAAGTCACAGATACCATTCGC  960
Sbjct  954   ..............C.....G.............................C.........  1013

Query  961   GGCGAGAGAGTCTCCTTCGCCGTGTGTACTTATGTACCAACAACACTCTGCGACCAGATG  1020
Sbjct  1014  ............................................................  1073

Query  1021  ACCGGGATCCTAGCAACAGAAGTAACAGCCGATGATGCCCAGAAACTGCTGGTGGGTTTG  1080
Sbjct  1074  ........T...................................................  1133

Query  1081  AACCAGAGAATAGTAGTTAATGGTAGGACCCAGAGAAATACCAATACTATGAAGAACTAC  1140
Sbjct  1134  ............................................C...............  1193

Query  1141  CTGCTACCACTGGTTGCACAAGCGCTAGCAAAATGGGCGAAGGAAGCAAAACAGGATATG  1200
Sbjct  1194  ......................................A.....................  1253
```

```
Query  1201  GAAGATGAAAGACCCCTGAACGAACGCCAACGAACGCTAACGTGCCTCTGCTGCTGGGCA  1260
Sbjct  1254  ............................................................  1313

Query  1261  TTTAAGCGAAACAAACGCCACGCCATTTACAAGAGACCAGACACACAGAGTATAGTCAAG  1320
Sbjct  1314  ............................................................  1373

Query  1321  GTCCCTTGCGAATTCACAAGCTTTCCTTTGGTCAGCCTGTGGTCCGCTGGGATGTCTATA  1380
Sbjct  1374  ............................................................  1433

Query  1381  TCTCTTAGGCAGAAGTTGAAGATGATGCTGCAGGCGAGGCAGCCCACACAAATAGCAGCA  1440
Sbjct  1434  ............................................................  1493

Query  1441  GTGACTGAGGAACTCATACAAGAAGCAGCTGCAGTAGAGCAAGAGGCCGTGGATACGGCC  1500
Sbjct  1494  ..................................C.........................  1553

Query  1501  AATGCCGAGCTGGACCACGCCGCATGGCCCTCCATTGTGGATACGACAGAGCGCCATGTT  1560
Sbjct  1554  ...............................................G............  1613

Query  1561  GAGGTCGAAGTGGAAGAACTCGACCAGCGTGCAGGGGAAGGGGTAGTGGAAACACCTCGA  1620
Sbjct  1614  ............................................................  1673

Query  1621  AACTCTATCAAAGTTTCAACACAGATCGGGGACGCGTTAATCGGCAGTTACCTGATCCTA  1680
Sbjct  1674  ........T........T..........................................  1733

Query  1681  TCACCCCAAGCAGTCCTACGCAGCGAAAAATTAGCCTGCATACATGATCTTGCAGAGCAG  1740
Sbjct  1734  ............................................................  1793

Query  1741  GTTAAGTTGGTCACACACTCTGGCCGTAGTGGTAGGTACGCCGTCGACAAATACNACGGA  1800
Sbjct  1794  ..C.................C..T..............................G.....  1853

Query  1801  AGAGTACTAGTCCCTACAGGAGTGGCTATAGACATTCAATCGTTCCAGGCTCTCAGTGAG  1860
Sbjct  1854  ...................................C........................  1913

Query  1861  AGCGCGACCCTTGTGTACAACGAACGCGAGTTCGTTAACAGGAAGCTGTGGCACATAGCA  1920
Sbjct  1914  ..T..........................................T..............  1973

Query  1921  GTATACGGGGCAGCACTCAATACTGATGAAGAAGGATACGAGAAGGTCCCGGTAGAGAGA  1980
Sbjct  1974  ............................................................  2033

Query  1981  GCAGAATCAGATTATGTGTTTGATGTAGACCAAAAAATGTGCCTaaaaaaaGAGCAGGCA  2040
Sbjct  2034  .................A............A.............C...............  2093

Query  2041  TCAGGTTGGGTACTCTGTGGCGAACTAGTCAACCCCCCATTCCACGAATTCGCATATGAA  2100
Sbjct  2094  .....A..................T...................................  2153

Query  2101  GGGCTCCGCACGAGACCGTCAGCACCCTACAAGGTTCATACAGTAGGTGTGTACGGAGTG  2160
Sbjct  2154  ........T..T......G.........................................  2213

Query  2161  CCAGGATCAGGCAAATCCGCAATAATCAAGAACACGGTCACCATGTCTGACCTAGTATTG  2220
Sbjct  2214  ............................................................  2273

Query  2221  AGTGGTAAGAAAGAGAACTGCTTAGAAATTATGAACGATGTACTTAAACACAGAGCTCTA  2280
Sbjct  2274  ............................................................  2333
```

```
Query  2281  CGTATCACAGCGAAGACCGTAGACTCAGTGTTATTAAACGGCGTGAAACACACGCCTAAC  2340
Sbjct  2334  .....T.............................G........................  2393

Query  2341  ATACTATACATCGACGAAGCGTTCTCATGCCATGCAGGGACTCTGTTGGCCACTATAGCC  2400
Sbjct  2394  ............................................................  2453

Query  2401  ATAGTCAGGCCCAAACAGAAAGTGGTACTGTGCGGAGACCCGAAACAATGCGGATTCTTC  2460
Sbjct  2454  T...........................................................  2513

Query  2461  AATATGATGCAACTGAAAGTTAATTACAATCATGACATCTGCTCAGAAGTCTTCCACAAA  2520
Sbjct  2514  .......................C....................................  2573

Query  2521  AGTATCTCTAGACGGTGCACCCAGGATATCACGGCCATCGTTTCCAAATTACATTACCAG  2580
Sbjct  2574  ............................................T...............  2633

Query  2581  GACCGAATGAGGACCACAAACCCCCGAAAAGGAGACATCATTATAGACACTACCGGCACT  2640
Sbjct  2634  ..................................................C.........  2693

Query  2641  ACCAAACCAGCCAAAACAGATCTGATTCTGACGTGCTTCAGGGGATGGGTGAAACAGTTG  2700
Sbjct  2694  ....................C.......................................  2753

Query  2701  CAGCAAGACTACAGAGGTAACGAAGTAATGACGGCTGCAGCGTCCCAAGGACTGACGAGG  2760
Sbjct  2754  ............................................................  2813

Query  2761  GCCTCCGTATATGCGGTTCGAACTAAAGTCAATGAGAACCCGCTATATGCACAGACCTCC  2820
Sbjct  2814  ............................................................  2873

Query  2821  GAGCACGTGAACGTGTTGTTAACACGCACAGAAAACAAGCTAGTATGGAAGACCTTGTCA  2880
Sbjct  2874  .......................................T....................  2933

Query  2881  ACAGATCCCTGGATTAAAACACTGACTAACCCACCTAGAGGGCACTATACCGCCACCATA  2940
Sbjct  2934  ............................................................  2993

Query  2941  GCAGAATGGGAAGCGGAACACCAGGGTATAATGAAGGCCATACAAGGGTATGCACCGCCC  3000
Sbjct  2994  ..............A.............................................  3053

Query  3001  GTGAACACCTTCATGAACAAAGTAAATGTGTGCTGGGCAAAGACACTTACGCCTGTGCTG  3060
Sbjct  3054  ...........T................................G..C..A.........  3113

Query  3061  GAAACTGCGGGTATCTCCCTGTCAGCAGAAGACTGGTCTGAACTGCTGCCCCCGTTTGCC  3120
Sbjct  3114  ..................T.........................................  3173

Query  3121  CAGGACGTGGCGTACTCACCCGAGGTGGCATTAAACATCATATGCACGAAAATGTATGGG  3180
Sbjct  3174  ...............................................A............  3233

Query  3181  TTTGACTTAGACACTGGTCTTTTTCCAGGCCATCAGTGCCAATGACATACACCAAAGAC  3240
Sbjct  3234  ........G.....C.............A.........A.....................  3293

Query  3241  CATTGGGATAACAGAGTTGGAGGGAAAATGTATGGATTCAGCCAACAAGCATACGATCAG  3300
Sbjct  3294  ............................................................  3353

Query  3301  CTGGCAAGACGACATCCGTACCTTCGAGGTAGAGAGAAATCAGGAATGCAGATCGTAGTC  3360
Sbjct  3354  ...................................A........................  3413
```

```
Query  3361  ACTGAAATGCGTATCCAGCGCCCAAGATCGGATGCCAACATCATCCCGATCAACCGCAGG  3420
Sbjct  3414  .....G......................................................  3473

Query  3421  CTCCCTCACTCACTCGTAGCCACACACGAGTATAGGCGAGCTGCACGGGCCGAGGAATTC  3480
Sbjct  3474  ............................................G...............  3533

Query  3481  TTCACCACGACACGAGGGTACACTATGCTGCTGGTCTCTGAGTATAACATGAACTTACCA  3540
Sbjct  3534  ..........T.................................................  3593

Query  3541  AACAAGAAGATCACCTGGCTGGCTCCGATAGGGACGCAGGGGGCCCATCACACCGCCAAC  3600
Sbjct  3594  ............................................................  3653

Query  3601  CTAAACTTGGGGATACCACCTCTGCTGGGCAGTTTTGATGCGGTGGTTGTGAACATGCCG  3660
Sbjct  3654  ........................................T...................  3713

Query  3661  ACTCCATTCCGGAACCATCACTACCAGCAATGTGAAGACCACGCGATGAAACTCCAGATG  3720
Sbjct  3714  ................................C...........................  3773

Query  3721  CTGGCAGGCGACGCACTGAGGCACATTAAACCTGGCGGATCATTGTGGGTCAAGGCATAC  3780
Sbjct  3774  .............................G..............................  3833

Query  3781  GGCTACGCAGACCGGCACAGCGAGCACGTGGTCTTGGCATTGGCTAGAAAGTTTAAAAGC  3840
Sbjct  3834  .......................................C....................  3893

Query  3841  TTCAGAGTCACACAACCCTCATGCGTGACTTCCAACACCGAGGTGTTTCTCCACTTCTCA  3900
Sbjct  3894  ..T........G........G.....A................................G  3953

Query  3901  ATTTTTGACAATGGCAAACGCGCGATAGCCCTGCATTCAGCTAATAGGAAGGCTAACAGT  3960
Sbjct  3954  ............................................................  4013

Query  3961  ATCTTCCAAAACACCTTCTTACCGGCGGGCAGTGCACCGGCGTACAGAGTCAAACGTGGA  4020
Sbjct  4014  ..........................A....A............................  4073

Query  4021  GACATTTCGAACGCCCCAGAGGATGCAGTGGTCAATGCAGCAAACCAACAGGGAGTGAAG  4080
Sbjct  4074  ............................................................  4133

Query  4081  GGTGCTGGAGTTTGCGGTGCAATTTACCGTAAGTGGCCGGACGCTTTCGGTGATGTCGCT  4140
Sbjct  4134  ............................................................  4193

Query  4141  ACTCCAACCGGAACAGCAGTTTCGAAATCCGTCCAAGATAAATTGGTGATCCACGCTGTC  4200
Sbjct  4194  ............................................................  4253

Query  4201  GGCCCGAATTTCTCAAAATGTTCAGAAGAGGAAGGGGACAGAGACCTAGCATCTGCTTAC  4260
Sbjct  4254  ...........T................................................  4313

Query  4261  AGAGCTGCAGCAGAAATAGTGATGGATaaaaaaaTTACAACAGTGGCCGTCCCCTTACTC  4320
Sbjct  4314  ..........................A...........................C.....  4373

Query  4321  TCCACCGGCATTTATGCCGGAGGAAAAAACAGAGTAGAACAGTCACTCAACCATCTCTTC  4380
Sbjct  4374  ..........C...C........G....................................  4433

Query  4381  ACGGCATTCGACAATACTGATGCAGATGTGACCATATATTGCATGGACAAAACATGGGAA  4440
Sbjct  4434  ........T...................................................  4493
```

```
Query  4441  AAGAAGATTAAGGAGGCAATCGATCACCGGACTTCGGTTGAGATGGTGCAGGATGACGTG  4500
Sbjct  4494  ............................................................  4553

Query  4501  CAGTTGGAGGAGGAACTGGTACGAGTACACCCTTTGAGTAGTTTAGCAGGTAGGAAGGGT  4560
Sbjct  4554  ......................................C.................A...  4613

Query  4561  TACAGTACGGACAGCGGCCGAGTGTTTTCCTACCTGGAAGGTACCAAATTCCATCAGACT  4620
Sbjct  4614  ............................................................  4673

Query  4621  GCGGTGGACATAGCCGAAATGCAAGTGCTGTGGCCCGCCCTCAAAGAGTCTAATGAGCAA  4680
Sbjct  4674  ........T.............................T.....................  4733

Query  4681  ATAGTGGCATACACCTTAGGAGAATCAATGGACCAGATACGTGGCAAGTGCCCGACAGAA  4740
Sbjct  4734  ............................................................  4793

Query  4741  GATACTGACGCCTCCACACCTCCACGGACTGTGCCGTGCCTCTGTCGATACGCCATGACA  4800
Sbjct  4794  ............................................................  4853

Query  4801  CCAGAGAGAGTGTACCGACTTAAATGCACGAACACTACCCAATTTACGGTTTGCTCATCT  4860
Sbjct  4854  ............................................C.....C.........  4913

Query  4861  TTTGAGTTGCCAAAGTATCACATTCAGGGAGTGCAGAGAGTAAAATGTGAAAGAATCATC  4920
Sbjct  4914  ............................................................  4973

Query  4921  ATCTTAGATCCCACTGTTCCACCAACTTACAAACGGCCATGCATCAGACGGTACCCCTCC  4980
Sbjct  4974  ............................................................  5033

Query  4981  ACAATCTCTTGTAACTCCTCTGAGGACTCCAGGAGCTTGTCTACTTTTTCTGTCAGCTCC  5040
Sbjct  5034  ........C......................................C............  5093

Query  5041  GACTCCTCGATTGGTTCTCTGCCGGTCGGAGACACGAGACCCATTCCAGCCCCGAGGACC  5100
Sbjct  5094  .........G.......CG..A...................T...........A......  5153

Query  5101  ATTTTCAGACCCGTCCCTGCCCCGAGAGCACCCGTGCTCAGAACCACACCGCCTCCTAAA  5160
Sbjct  5154  G...............................T..........T............C...  5213

Query  5161  CCACCGCGCACATTCACCGTGCGTGCAGAAGTGCACCAAGCACCCCCTACACCTGTACCT  5220
Sbjct  5214  ...................................T........................  5273

Query  5221  CCACCCAGACCGAAGAGGGCTGCAAAGTTGGCTCGTGAGATGCACCCCGGGTTCACCTTC  5280
Sbjct  5274  .....T.............................C........................  5333

Query  5281  GGGGACTTCGGAGAGCACGAGGTTGAGGAGCTTACGGCCTCTCCCTTAACCTTCGGAGAT  5340
Sbjct  5334  ..A..................A.................G....................  5393

Query  5341  TTTGCTGAAGGAGAGATCCAGGGGATGGGAGTGGAGTTTGAATGACTAGGCAGAGCCGGC  5400
Sbjct  5394  ....................A....C..................................  5453

Query  5401  GGGTACATTTTTTCGTCAGACACGGGTCCAGGCCACCTACAGCAGAGATCCGTTTTACAA  5460
Sbjct  5454  .........................................A.....G............  5513

Query  5461  AATTGCACGGCAGAATGTATCTACGAACCGGCAAAACTAGAAAAAATTCATGCACCAAAG  5520
Sbjct  5514  ............................................................  5573
```

```
Query  5521  TTGGATAAAACCAAGGAAGATATCTTAAGGAGCAAGTACCAAATGAAACCGTCTGAAGCA  5580
Sbjct  5574  ............................................................  5633

Query  5581  AACAAAAGCAGGTACCAATCTAGAAAAGTAGAAAATATGAAAGCAGAGATCGTAGGTAGA  5640
Sbjct  5634  ........T.........................................T.........  5693

Query  5641  CTCTTGGACGGACTGGGGGAGTATCTGGGCACCGAGCATCCAGTTGAATGCTACCGAATA  5700
Sbjct  5694  ..............A.............................................  5753

Query  5701  ACGTACCCGGTGCCTATATACTCAACTAGTGACCTCAGAGGTCTGTCTAGTGCCAAAACA  5760
Sbjct  5754  .....T....C...................AT..G.........................  5813

Query  5761  GCTGTTAGAGCTTGCAATGCATTTTTGGAAGCTAATTTTCCATCAGTCACTTCATATAAA  5820
Sbjct  5814  ............................................................  5873

Query  5821  ATTACTGATGAATACGACGCATACCTAGATATGGTAGATGGATCAGAGAGCTGTCTGGAC  5880
Sbjct  5874  ............................................................  5933

Query  5881  AGATCCTCCTTTTCGCCGTCTAGATTGCGTAGCTTTCCAAAAACACACTCATACTTGGAC  5940
Sbjct  5934  .................A...........C..............................  5993

Query  5941  CCACAGATCAACAGTGCGGTACCGTCACCATTCCAAAACACCTTACAAAATGTATTGGCA  6000
Sbjct  5994  ..G.........................................................  6053

Query  6001  GCGGCCACCAAAAGAAACTGTAATGTCACACAGATGAGAGAACTACCAACATATGATTCT  6060
Sbjct  6054  ...............................................G............  6113

Query  6061  GCAGTGCTAAATGTAGAGGCCTTCAGGAAATATGCGTGCAAGCCAGACGTATGGGATGAG  6120
Sbjct  6114  ............................................................  6173

Query  6121  TACAGGGATAATCCGATTTGCATAACCACCGAAAATGTCACCACTTACGTCGCCAAGTTG  6180
Sbjct  6174  ..............A...................................T..T......  6233

Query  6181  AAAGGACCGAAAGCTGCGGCCTTGTTTGCAAAAACACATAACCTGATACCACTACACCAA  6240
Sbjct  6234  ............................................................  6293

Query  6241  GTTCCTATGGACAAATTCACGGTAGATATGAAGAGAGATGTCAAAGTCACGCCCGGAACC  6300
Sbjct  6294  ............................................................  6353

Query  6301  AAGCACACCGAAGAGAGACCAAAGGTACAGGTGATTCAAGCGGCAGAGCCACTAGCCACT  6360
Sbjct  6354  ............................................................  6413

Query  6361  GCCTACCTCTGCGGAATTCACCGTGAATTGGTGCGCCGTCTCAACAACGCGCTTTTCCCA  6420
Sbjct  6414  .....................................................C.....T  6473

Query  6421  AACATCCACACTTTGTTTGATATGTCCGCAGAGGATTTCGATGCAATCATAGCGGAACAT  6480
Sbjct  6474  ..T...................................T.....................  6533

Query  6481  TTTAAGCACGGTGACCATGTGTTGGAAACGGATATAGCCTCTTTTGACAAAAGTCAAGAT  6540
Sbjct  6534  .................C..........................................  6593

Query  6541  GATTCCATGGCACTCACTGCGTTAATGATCCTTGAGGACCTGGGAGTAGACCAAAACCTA  6600
Sbjct  6594  ............................................................  6653
```

```
Query  6601  ATGAATTTGATAGAGGCTGCATTCGGGGAAATCGTGAGTACACACTTGCCCACAGGTACT  6660
Sbjct  6654  ............................................................  6713

Query  6661  AGATTCAAATTTGGAGCTATGATGAAGTCTGGAATGTTTTTGACGCTGTTCGTCAATACA  6720
Sbjct  6714  .......G.....................C...............T..............  6773

Query  6721  ATTCTTAATGTGGTTATTGCGTGCCGAGTGTTGGAGGATCAATTGGCGCAGTCGCCGTGG  6780
Sbjct  6774  ........C..................................................C  6833

Query  6781  CCTGCTTTCATAGGAGATGACAACATAATCCATGGTATAATATCAGACAAATTGATGGCA  6840
Sbjct  6834  G...............................C..........................G  6893

Query  6841  GATAGATGTGCCACCTGGATGAACATGGAGGTCAAGATACTGGACTCTATAGTTGGAATA  6900
Sbjct  6894  ............................................T..C............  6953

Query  6901  CGGCCACCTTACTTCTGTGGAGGATTTATTGTATGTGACGATGTAACAGGTACAGCCTGC  6960
Sbjct  6954  ...........T................................................  7013

Query  6961  CGCGTCGCAGACCCACTGAAGAGATTGTTCAAGCTAGGTAAGCCATTGCCACTTGACGAT  7020
Sbjct  7014  ........................C...................................  7073

Query  7021  GGCCAAGATGAAGACAGAAGACGTGCATTACATGATGAAGTGAAAACCTGGTCGCGCGTA  7080
Sbjct  7074  .............................G..............................  7133

Query  7081  GGGCTGCGACACAGAGTGTGTGAAGCCATCGAAGACCGTTATGCCGTCCACTCATCAGAA  7140
Sbjct  7134  ..................................................T.........  7193

Query  7141  CTAGTTTTATTGGCACTGACTACTCTGTCTAAGAACTTGAAGTCCTTCAGAAACATAAGA  7200
Sbjct  7194  ..............G.............................................  7253

Query  7201  GGGAAACCAATACATCTCTACGGTGGTCCTAAATAG  7236
Sbjct  7254  ....................................  7289
```

>Barmah Forest virus isolate MIDIWBTA.2018, complete genome
Sequence ID: MN064697.1 Length: 11563
Range 1: 63 to 7298

Score:12578 bits(6811), Expect:0.0,
Identities:7094/7236(98%), Gaps:0/7236(0%), Strand: Plus/Plus

```
Query  1     ATGGCGAAACCAGTTGTGAAGATCGACGTGGAACCTGAAAGCCATTTCGCTAAGCAGGTC  60
Sbjct  63    ...........G...................................T............  122

Query  61    CAGAGTTGCTTCCCGCAGTTTGAGATCGAAGCAGTGCAGACCACACCAAACGATCATGCA  120
Sbjct  123   .........................................G..................  182

Query  121   CACGCGAGGGCGTTTTCGCACCTTGCTACGAAGCTCATAGAAATGGAGACAGCAAAAGAT  180
Sbjct  183   ............................................................  242

Query  181   CAGATCATCCTCGATATCGGAAGTGCACCCGCGAGGAGACTGTATTCAGAACACAAGTAC  240
Sbjct  243   ............................................................  302
```

```
Query  241   CACTGTGTTTGCCCAATGAAGTGCACGGAAGATCCAGAGAGAATGCTAGGATATGCACGT  300
Sbjct  303   ............................................................  362

Query  301   AAGTTGATCGCAGGCTCTGCGAAAGGGAAGGCAGAAAAGTTACGCGATCTCAGGGATGTC  360
Sbjct  363   ............................................................  422

Query  361   TTGGCTACGCCAGACATCGAGACGCAGTCGCTATGTCTCCACACAGACGCATCCTGCAGA  420
Sbjct  423   ................................G...........................  482

Query  421   TACCGCGGTGATGTTGCCGTGTATCAAGACGTGTATGCCATTGACGCACCTACCACGCTG  480
Sbjct  483   ..................................................C.........  542

Query  481   TACCACCAAGCGTTAAAGGGCGTCAGGACCGCATATTGGATAGGCTTTGATACAACGCCG  540
Sbjct  543   ...........................................................A  602

Query  541   TTCATGTACGATGCACTAGCAGGAGCTTACCCGCTCTACTCCACAAACTGGGCTGATGAG  600
Sbjct  603   .....................................................C......  662

Query  601   CAAGTGCTCGAGTCCAGAAACATTGGGCTATGTTCAGACAAAGTTTCTGAAGGGGGAAAG  660
Sbjct  663   ............................................................  722

Query  661   AAAGGGAGATCAATCCTCAGGAAGAAGTTCTTGAAGCAGTCAGACAGAGTCATGTTCTCT  720
Sbjct  723   ............................................................  782

Query  721   GTCGGCTCGACGTTGTATACGGAAAGCCGTAAATTACTGCAAAGTTGGCACCTGCCATCC  780
Sbjct  783   ............................................C...............  842

Query  781   ACATTCCATCTCAAAGGCAAATCTTCGTTCACGTGCCGCTGCGACACTATCGTCAGCTGC  840
Sbjct  843   ................................A..............C............  902

Query  841   GAAGGGTATGTTCTGAAGAAAATTACAATGTGTCCTGGAGTGACAGGCAAACCGATAGGA  900
Sbjct  903   ................................C..C........................  962

Query  901   TATGCCGTCACCCATCACAAAGAAGGATTCGTAGTCGGAAAAGTCACAGATACCATTCGC  960
Sbjct  963   ..............C.....G.............................C.........  1022

Query  961   GGCGAGAGAGTCTCCTTCGCCGTGTGTACTTATGTACCAACAACACTCTGCGACCAGATG  1020
Sbjct  1023  ............................................................  1082

Query  1021  ACCGGGATCCTAGCAACAGAAGTAACAGCCGATGATGCCCAGAAACTGCTGGTGGGTTTG  1080
Sbjct  1083  ........T...................................................  1142

Query  1081  AACCAGAGAATAGTAGTTAATGGTAGGACCCAGAGAAATACCAATACTATGAAGAACTAC  1140
Sbjct  1143  ............................................C...............  1202

Query  1141  CTGCTACCACTGGTTGCACAAGCGCTAGCAAAATGGGCGAAGGAAGCAAAACAGGATATG  1200
Sbjct  1203  ......................................A.....................  1262

Query  1201  GAAGATGAAAGACCCCTGAACGAACGCCAACGAACGCTAACGTGCCTCTGCTGCTGGGCA  1260
Sbjct  1263  ............................................................  1322

Query  1261  TTTAAGCGAAACAAACGCCACGCCATTTACAAGAGACCAGACACACAGAGTATAGTCAAG  1320
Sbjct  1323  ............................................................  1382
```

```
Query  1321  GTCCCTTGCGAATTCACAAGCTTTCCTTTGGTCAGCCTGTGGTCCGCTGGGATGTCTATA  1380
Sbjct  1383  ............................................................  1442

Query  1381  TCTCTTAGGCAGAAGTTGAAGATGATGCTGCAGGCGAGGCAGCCCACACAAATAGCAGCA  1440
Sbjct  1443  ............................................................  1502

Query  1441  GTGACTGAGGAACTCATACAAGAAGCAGCTGCAGTAGAGCAAGAGGCCGTGGATACGGCC  1500
Sbjct  1503  ..................................C.........................  1562

Query  1501  AATGCCGAGCTGGACCACGCCGCATGGCCCTCCATTGTGGATACGACAGAGCGCCATGTT  1560
Sbjct  1563  ...............................................G............  1622

Query  1561  GAGGTCGAAGTGGAAGAACTCGACCAGCGTGCAGGGGAAGGGGTAGTGGAAACACCTCGA  1620
Sbjct  1623  ............................................................  1682

Query  1621  AACTCTATCAAAGTTTCAACACAGATCGGGGACGCGTTAATCGGCAGTTACCTGATCCTA  1680
Sbjct  1683  ........T........T..........................................  1742

Query  1681  TCACCCCAAGCAGTCCTACGCAGCGAAAAATTAGCCTGCATACATGATCTTGCAGAGCAG  1740
Sbjct  1743  ............................................................  1802

Query  1741  GTTAAGTTGGTCACACACTCTGGCCGTAGTGGTAGGTACGCCGTCGACAAATACNACGGA  1800
Sbjct  1803  ..C.................C..T..............................G.....  1862

Query  1801  AGAGTACTAGTCCCTACAGGAGTGGCTATAGACATTCAATCGTTCCAGGCTCTCAGTGAG  1860
Sbjct  1863  ...................................C........................  1922

Query  1861  AGCGCGACCCTTGTGTACAACGAACGCGAGTTCGTTAACAGGAAGCTGTGGCACATAGCA  1920
Sbjct  1923  ..T..........................................T..............  1982

Query  1921  GTATACGGGGCAGCACTCAATACTGATGAAGAAGGATACGAGAAGGTCCCGGTAGAGAGA  1980
Sbjct  1983  ............................................................  2042

Query  1981  GCAGAATCAGATTATGTGTTTGATGTAGACCAAAAAATGTGCCTaaaaaaaGAGCAGGCA  2040
Sbjct  2043  .................A............A.............C...............  2102

Query  2041  TCAGGTTGGGTACTCTGTGGCGAACTAGTCAACCCCCCATTCCACGAATTCGCATATGAA  2100
Sbjct  2103  .....A..................T...................................  2162

Query  2101  GGGCTCCGCACGAGACCGTCAGCACCCTACAAGGTTCATACAGTAGGTGTGTACGGAGTG  2160
Sbjct  2163  ........T..T......G.........................................  2222

Query  2161  CCAGGATCAGGCAAATCCGCAATAATCAAGAACACGGTCACCATGTCTGACCTAGTATTG  2220
Sbjct  2223  ............................................................  2282

Query  2221  AGTGGTAAGAAAGAGAACTGCTTAGAAATTATGAACGATGTACTTAAACACAGAGCTCTA  2280
Sbjct  2283  ............................................................  2342

Query  2281  CGTATCACAGCGAAGACCGTAGACTCAGTGTTATTAAACGGCGTGAAACACACGCCTAAC  2340
Sbjct  2343  .....T....................G........G........................  2402

Query  2341  ATACTATACATCGACGAAGCGTTCTCATGCCATGCAGGGACTCTGTTGGCCACTATAGCC  2400
Sbjct  2403  ............................................................  2462
```

```
Query  2401  ATAGTCAGGCCCAAACAGAAAGTGGTACTGTGCGGAGACCCGAAACAATGCGGATTCTTC  2460
Sbjct  2463  T...........................................................  2522

Query  2461  AATATGATGCAACTGAAAGTTAATTACAATCATGACATCTGCTCAGAAGTCTTCCACAAA  2520
Sbjct  2523  .......................C....................................  2582

Query  2521  AGTATCTCTAGACGGTGCACCCAGGATATCACGGCCATCGTTTCCAAATTACATTACCAG  2580
Sbjct  2583  ............................................T...............  2642

Query  2581  GACCGAATGAGGACCACAAACCCCCGAAAGGAGACATCATTATAGACACTACCGGCACT  2640
Sbjct  2643  ..................................................C.........  2702

Query  2641  ACCAAACCAGCCAAAACAGATCTGATTCTGACGTGCTTCAGGGGATGGGTGAAACAGTTG  2700
Sbjct  2703  ....................C.......................................  2762

Query  2701  CAGCAAGACTACAGAGGTAACGAAGTAATGACGGCTGCAGCGTCCCAAGGACTGACGAGG  2760
Sbjct  2763  ............................................................  2822

Query  2761  GCCTCCGTATATGCGGTTCGAACTAAAGTCAATGAGAACCCGCTATATGCACAGACCTCC  2820
Sbjct  2823  ............................................................  2882

Query  2821  GAGCACGTGAACGTGTTGTTAACACGCACAGAAAACAAGCTAGTATGGAAGACCTTGTCA  2880
Sbjct  2883  .......................................T....................  2942

Query  2881  ACAGATCCCTGGATTAAAACACTGACTAACCCACCTAGAGGGCACTATACCGCCACCATA  2940
Sbjct  2943  ............................................................  3002

Query  2941  GCAGAATGGGAAGCGGAACACCAGGGTATAATGAAGGCCATACAAGGGTATGCACCGCCC  3000
Sbjct  3003  ..............A...........C.................................  3062

Query  3001  GTGAACACCTTCATGAACAAAGTAAATGTGTGCTGGGCAAAGACACTTACGCCTGTGCTG  3060
Sbjct  3063  ...........T................................G..C..A.........  3122

Query  3061  GAAACTGCGGGTATCTCCCTGTCAGCAGAAGACTGGTCTGAACTGCTGCCCCCGTTTGCC  3120
Sbjct  3123  ..................T.........................................  3182

Query  3121  CAGGACGTGGCGTACTCACCCGAGGTGGCATTAAACATCATATGCACGAAAATGTATGGG  3180
Sbjct  3183  ...............................................A............  3242

Query  3181  TTTGACTTAGACACTGGTCTTTTTTCCAGGCCATCAGTGCCAATGACATACACCAAAGAC  3240
Sbjct  3243  ........G.....C.............A.........A.....................  3302

Query  3241  CATTGGGATAACAGAGTTGGAGGGAAAATGTATGGATTCAGCCAACAAGCATACGATCAG  3300
Sbjct  3303  ............................................................  3362

Query  3301  CTGGCAAGACGACATCCGTACCTTCGAGGTAGAGAGAAATCAGGAATGCAGATCGTAGTC  3360
Sbjct  3363  ...................................A........................  3422

Query  3361  ACTGAAATGCGTATCCAGCGCCCAAGATCGGATGCCAACATCATCCCGATCAACCGCAGG  3420
Sbjct  3423  .....G......................................................  3482

Query  3421  CTCCCTCACTCACTCGTAGCCACACACGAGTATAGGCGAGCTGCACGGGCCGAGGAATTC  3480
Sbjct  3483  ............................................G...............  3542
```

```
Query  3481  TTCACCACGACACGAGGGTACACTATGCTGCTGGTCTCTGAGTATAACATGAACTTACCA  3540
Sbjct  3543  ..........T.................................................  3602

Query  3541  AACAAGAAGATCACCTGGCTGGCTCCGATAGGGACGCAGGGGGCCCATCACACCGCCAAC  3600
Sbjct  3603  ............................................................  3662

Query  3601  CTAAACTTGGGGATACCACCTCTGCTGGGCAGTTTTGATGCGGTGGTTGTGAACATGCCG  3660
Sbjct  3663  ........................................T...................  3722

Query  3661  ACTCCATTCCGGAACCATCACTACCAGCAATGTGAAGACCACGCGATGAAACTCCAGATG  3720
Sbjct  3723  ................................C...........................  3782

Query  3721  CTGGCAGGCGACGCACTGAGGCACATTAAACCTGGCGGATCATTGTGGGTCAAGGCATAC  3780
Sbjct  3783  .............................G..............................  3842

Query  3781  GGCTACGCAGACCGGCACAGCGAGCACGTGGTCTTGGCATTGGCTAGAAAGTTTAAAAGC  3840
Sbjct  3843  .......................................C....................  3902

Query  3841  TTCAGAGTCACACAACCCTCATGCGTGACTTCCAACACCGAGGTGTTTCTCCACTTCTCA  3900
Sbjct  3903  ..T........G........G.....A................................G  3962

Query  3901  ATTTTTGACAATGGCAAACGCGCGATAGCCCTGCATTCAGCTAATAGGAAGGCTAACAGT  3960
Sbjct  3963  ............................................................  4022

Query  3961  ATCTTCCAAAACACCTTCTTACCGGCGGGCAGTGCACCGGCGTACAGAGTCAAACGTGGA  4020
Sbjct  4023  ..........................A....A............................  4082

Query  4021  GACATTTCGAACGCCCCAGAGGATGCAGTGGTCAATGCAGCAAACCAACAGGGAGTGAAG  4080
Sbjct  4083  ............................................................  4142

Query  4081  GGTGCTGGAGTTTGCGGTGCAATTTACCGTAAGTGGCCGGACGCTTTCGGTGATGTCGCT  4140
Sbjct  4143  ............................................................  4202

Query  4141  ACTCCAACCGGAACAGCAGTTTCGAAATCCGTCCAAGATAAATTGGTGATCCACGCTGTC  4200
Sbjct  4203  ............................................................  4262

Query  4201  GGCCCGAATTTCTCAAAATGTTCAGAAGAGGAAGGGGACAGAGACCTAGCATCTGCTTAC  4260
Sbjct  4263  ...........T................................................  4322

Query  4261  AGAGCTGCAGCAGAAATAGTGATGGATaaaaaaaTTACAACAGTGGCCGTCCCCTTACTC  4320
Sbjct  4323  .............C............A...........................C.....  4382

Query  4321  TCCACCGGCATTTATGCCGGAGGAAAAAACAGAGTAGAACAGTCACTCAACCATCTCTTC  4380
Sbjct  4383  ..............C........G....................................  4442

Query  4381  ACGGCATTCGACAATACTGATGCAGATGTGACCATATATTGCATGGACAAAACATGGGAA  4440
Sbjct  4443  ........T...................................................  4502

Query  4441  AAGAAGATTAAGGAGGCAATCGATCACCGGACTTCGGTTGAGATGGTGCAGGATGACGTG  4500
Sbjct  4503  ............................................................  4562

Query  4501  CAGTTGGAGGAGGAACTGGTACGAGTACACCCTTTGAGTAGTTTAGCAGGTAGGAAGGGT  4560
Sbjct  4563  ......................................C.................A...  4622
```

```
Query  4561  TACAGTACGGACAGCGGCCGAGTGTTTTCCTACCTGGAAGGTACCAAATTCCATCAGACT  4620
Sbjct  4623  ............................................................  4682

Query  4621  GCGGTGGACATAGCCGAAATGCAAGTGCTGTGGCCCGCCCTCAAAGAGTCTAATGAGCAA  4680
Sbjct  4683  ........T.............................T.....................  4742

Query  4681  ATAGTGGCATACACCTTAGGAGAATCAATGGACCAGATACGTGGCAAGTGCCCGACAGAA  4740
Sbjct  4743  ............................................................  4802

Query  4741  GATACTGACGCCTCCACACCTCCACGGACTGTGCCGTGCCTCTGTCGATACGCCATGACA  4800
Sbjct  4803  ............................................................  4862

Query  4801  CCAGAGAGAGTGTACCGACTTAAATGCACGAACACTACCCAATTTACGGTTTGCTCATCT  4860
Sbjct  4863  ............................................C.....C.........  4922

Query  4861  TTTGAGTTGCCAAAGTATCACATTCAGGGAGTGCAGAGAGTAAAATGTGAAAGAATCATC  4920
Sbjct  4923  ............................................................  4982

Query  4921  ATCTTAGATCCCACTGTTCCACCAACTTACAAACGGCCATGCATCAGACGGTACCCCTCC  4980
Sbjct  4983  ............................................................  5042

Query  4981  ACAATCTCTTGTAACTCCTCTGAGGACTCCAGGAGCTTGTCTACTTTTTCTGTCAGCTCC  5040
Sbjct  5043  ........C......................................C............  5102

Query  5041  GACTCCTCGATTGGTTCTCTGCCGGTCGGAGACACGAGACCCATTCCAGCCCCGAGGACC  5100
Sbjct  5103  .................CG..A...................T...........A......  5162

Query  5101  ATTTTCAGACCCGTCCCTGCCCCGAGAGCACCCGTGCTCAGAACCACACCGCCTCCTAAA  5160
Sbjct  5163  G...............................T..........T..T.............  5222

Query  5161  CCACCGCGCACATTCACCGTGCGTGCAGAAGTGCACCAAGCACCCCCTACACCTGTACCT  5220
Sbjct  5223  ...................................T........................  5282

Query  5221  CCACCCAGACCGAAGAGGGCTGCAAAGTTGGCTCGTGAGATGCACCCCGGGTTCACCTTC  5280
Sbjct  5283  .....T......................................................  5342

Query  5281  GGGGACTTCGGAGAGCACGAGGTTGAGGAGCTTACGGCCTCTCCCTTAACCTTCGGAGAT  5340
Sbjct  5343  ..A..................A.................G....................  5402

Query  5341  TTTGCTGAAGGAGAGATCCAGGGGATGGGAGTGGAGTTTGAATGACTAGGCAGAGCCGGC  5400
Sbjct  5403  ....................A....C..................................  5462

Query  5401  GGGTACATTTTTTCGTCAGACACGGGTCCAGGCCACCTACAGCAGAGATCCGTTTTACAA  5460
Sbjct  5463  .........................................A.....G............  5522

Query  5461  AATTGCACGGCAGAATGTATCTACGAACCGGCAAAACTAGAAAAAATTCATGCACCAAAG  5520
Sbjct  5523  ............................................................  5582

Query  5521  TTGGATAAAACCAAGGAAGATATCTTAAGGAGCAAGTACCAAATGAAACCGTCTGAAGCA  5580
Sbjct  5583  ............................................................  5642

Query  5581  AACAAAAGCAGGTACCAATCTAGAAAAGTAGAAAATATGAAAGCAGAGATCGTAGGTAGA  5640
Sbjct  5643  ........T.........................................T.........  5702
```

```
Query  5641  CTCTTGGACGGACTGGGGGAGTATCTGGGCACCGAGCATCCAGTTGAATGCTACCGAATA  5700
Sbjct  5703  ..............A.............................................  5762

Query  5701  ACGTACCCGGTGCCTATATACTCAACTAGTGACCTCAGAGGTCTGTCTAGTGCCAAAACA  5760
Sbjct  5763  .....T....C...................AT..G.........................  5822

Query  5761  GCTGTTAGAGCTTGCAATGCATTTTTGGAAGCTAATTTTCCATCAGTCACTTCATATAAA  5820
Sbjct  5823  .....C......................................................  5882

Query  5821  ATTACTGATGAATACGACGCATACCTAGATATGGTAGATGGATCAGAGAGCTGTCTGGAC  5880
Sbjct  5883  ............................................................  5942

Query  5881  AGATCCTCCTTTTCGCCGTCTAGATTGCGTAGCTTTCCAAAAACACACTCATACTTGGAC  5940
Sbjct  5943  .................A...........C..............................  6002

Query  5941  CCACAGATCAACAGTGCGGTACCGTCACCATTCCAAAACACCTTACAAAATGTATTGGCA  6000
Sbjct  6003  ..G.........................................................  6062

Query  6001  GCGGCCACCAAAAGAAACTGTAATGTCACACAGATGAGAGAACTACCAACATATGATTCT  6060
Sbjct  6063  ...............................................G............  6122

Query  6061  GCAGTGCTAAATGTAGAGGCCTTCAGGAAATATGCGTGCAAGCCAGACGTATGGGATGAG  6120
Sbjct  6123  ............................................................  6182

Query  6121  TACAGGGATAATCCGATTTGCATAACCACCGAAAATGTCACCACTTACGTCGCCAAGTTG  6180
Sbjct  6183  ..............A...................................T..T......  6242

Query  6181  AAAGGACCGAAAGCTGCGGCCTTGTTTGCAAAAACACATAACCTGATACCACTACACCAA  6240
Sbjct  6243  ............................................................  6302

Query  6241  GTTCCTATGGACAAATTCACGGTAGATATGAAGAGAGATGTCAAAGTCACGCCCGGAACC  6300
Sbjct  6303  ............................................................  6362

Query  6301  AAGCACACCGAAGAGAGACCAAAGGTACAGGTGATTCAAGCGGCAGAGCCACTAGCCACT  6360
Sbjct  6363  ............................................................  6422

Query  6361  GCCTACCTCTGCGGAATTCACCGTGAATTGGTGCGCCGTCTCAACAACGCGCTTTTCCCA  6420
Sbjct  6423  .....................................................C.....T  6482

Query  6421  AACATCCACACTTTGTTTGATATGTCCGCAGAGGATTTCGATGCAATCATAGCGGAACAT  6480
Sbjct  6483  ..T...................................T.....................  6542

Query  6481  TTTAAGCACGGTGACCATGTGTTGGAAACGGATATAGCCTCTTTTGACAAAAGTCAAGAT  6540
Sbjct  6543  .................C..........................................  6602

Query  6541  GATTCCATGGCACTCACTGCGTTAATGATCCTTGAGGACCTGGGAGTAGACCAAAACCTA  6600
Sbjct  6603  ............................................................  6662

Query  6601  ATGAATTTGATAGAGGCTGCATTCGGGGAAATCGTGAGTACACACTTGCCCACAGGTACT  6660
Sbjct  6663  ............................................................  6722

Query  6661  AGATTCAAATTTGGAGCTATGATGAAGTCTGGAATGTTTTTGACGCTGTTCGTCAATACA  6720
Sbjct  6723  .......G.....................C...............T..............  6782
```

```
Query  6721  ATTCTTAATGTGGTTATTGCGTGCCGAGTGTTGGAGGATCAATTGGCGCAGTCGCCGTGG  6780
Sbjct  6783  ........C..................................................C  6842

Query  6781  CCTGCTTTCATAGGAGATGACAACATAATCCATGGTATAATATCAGACAAATTGATGGCA  6840
Sbjct  6843  G...............................C..........................G  6902

Query  6841  GATAGATGTGCCACCTGGATGAACATGGAGGTCAAGATACTGGACTCTATAGTTGGAATA  6900
Sbjct  6903  ............................................T..C............  6962

Query  6901  CGGCCACCTTACTTCTGTGGAGGATTTATTGTATGTGACGATGTAACAGGTACAGCCTGC  6960
Sbjct  6963  ...........T................................................  7022

Query  6961  CGCGTCGCAGACCCACTGAAGAGATTGTTCAAGCTAGGTAAGCCATTGCCACTTGACGAT  7020
Sbjct  7023  ........................C...................................  7082

Query  7021  GGCCAAGATGAAGACAGAAGACGTGCATTACATGATGAAGTGAAAACCTGGTCGCGCGTA  7080
Sbjct  7083  .............................G..............................  7142

Query  7081  GGGCTGCGACACAGAGTGTGTGAAGCCATCGAAGACCGTTATGCCGTCCACTCATCAGAA  7140
Sbjct  7143  ..................................................T.........  7202

Query  7141  CTAGTTTTATTGGCACTGACTACTCTGTCTAAGAACTTGAAGTCCTTCAGAAACATAAGA  7200
Sbjct  7203  ..............G.............................................  7262

Query  7201  GGGAAACCAATACATCTCTACGGTGGTCCTAAATAG  7236
Sbjct  7263  ....................................  7298
```

>Barmah Forest virus isolate MIDIWB78.2018, complete genome
Sequence ID: MK697274.1 Length: 11503
Range 1: 63 to 7298

Score:12578 bits(6811), Expect:0.0,
Identities:7094/7236(98%), Gaps:0/7236(0%), Strand: Plus/Plus

```
Query  1      ATGGCGAAACCAGTTGTGAAGATCGACGTGGAACCTGAAAGCCATTTCGCTAAGCAGGTC  60
Sbjct  63     ...........G...................................T............  122

Query  61     CAGAGTTGCTTCCCGCAGTTTGAGATCGAAGCAGTGCAGACCACACCAAACGATCATGCA  120
Sbjct  123    .........................................G..................  182

Query  121    CACGCGAGGGCGTTTTCGCACCTTGCTACGAAGCTCATAGAAATGGAGACAGCAAAAGAT  180
Sbjct  183    ............................................................  242

Query  181    CAGATCATCCTCGATATCGGAAGTGCACCCGCGAGGAGACTGTATTCAGAACACAAGTAC  240
Sbjct  243    ............................................................  302

Query  241    CACTGTGTTTGCCCAATGAAGTGCACGGAAGATCCAGAGAGAATGCTAGGATATGCACGT  300
Sbjct  303    ............................................................  362

Query  301    AAGTTGATCGCAGGCTCTGCGAAAGGGAAGGCAGAAAAGTTACGCGATCTCAGGGATGTC  360
```

```
Sbjct  363   ............................................................  422

Query  361   TTGGCTACGCCAGACATCGAGACGCAGTCGCTATGTCTCCACACAGACGCATCCTGCAGA  420
Sbjct  423   ................................G...........................  482

Query  421   TACCGCGGTGATGTTGCCGTGTATCAAGACGTGTATGCCATTGACGCACCTACCACGCTG  480
Sbjct  483   ..................................................C.........  542

Query  481   TACCACCAAGCGTTAAAGGGCGTCAGGACCGCATATTGGATAGGCTTTGATACAACGCCG  540
Sbjct  543   ...........................................................A  602

Query  541   TTCATGTACGATGCACTAGCAGGAGCTTACCCGCTCTACTCCACAAACTGGGCTGATGAG  600
Sbjct  603   .....................................................C......  662

Query  601   CAAGTGCTCGAGTCCAGAAACATTGGGCTATGTTCAGACAAAGTTTCTGAAGGGGGAAAG  660
Sbjct  663   ............................................................  722

Query  661   AAAGGGAGATCAATCCTCAGGAAGAAGTTCTTGAAGCAGTCAGACAGAGTCATGTTCTCT  720
Sbjct  723   ............................................................  782

Query  721   GTCGGCTCGACGTTGTATACGGAAAGCCGTAAATTACTGCAAAGTTGGCACCTGCCATCC  780
Sbjct  783   ............................................C...............  842

Query  781   ACATTCCATCTCAAAGGCAAATCTTCGTTCACGTGCCGCTGCGACACTATCGTCAGCTGC  840
Sbjct  843   ................................A..............C............  902

Query  841   GAAGGGTATGTTCTGAAGAAAATTACAATGTGTCCTGGAGTGACAGGCAAACCGATAGGA  900
Sbjct  903   ................................C..C........................  962

Query  901   TATGCCGTCACCCATCACAAAGAAGGATTCGTAGTCGGAAAAGTCACAGATACCATTCGC  960
Sbjct  963   ..............C.....G.............................C.........  1022

Query  961   GGCGAGAGAGTCTCCTTCGCCGTGTGTACTTATGTACCAACAACACTCTGCGACCAGATG  1020
Sbjct  1023  ............................................................  1082

Query  1021  ACCGGGATCCTAGCAACAGAAGTAACAGCCGATGATGCCCAGAAACTGCTGGTGGGTTTG  1080
Sbjct  1083  ........T...................................................  1142

Query  1081  AACCAGAGAATAGTAGTTAATGGTAGGACCCAGAGAAATACCAATACTATGAAGAACTAC  1140
Sbjct  1143  ............................................C...............  1202

Query  1141  CTGCTACCACTGGTTGCACAAGCGCTAGCAAAATGGGCGAAGGAAGCAAAACAGGATATG  1200
Sbjct  1203  ......................................A.....................  1262

Query  1201  GAAGATGAAAGACCCCTGAACGAACGCCAACGAACGCTAACGTGCCTCTGCTGCTGGGCA  1260
Sbjct  1263  ............................................................  1322

Query  1261  TTTAAGCGAAACAAACGCCACGCCATTTACAAGAGACCAGACACACAGAGTATAGTCAAG  1320
Sbjct  1323  ............................................................  1382

Query  1321  GTCCCTTGCGAATTCACAAGCTTTCCTTTGGTCAGCCTGTGGTCCGCTGGGATGTCTATA  1380
Sbjct  1383  ............................................................  1442

Query  1381  TCTCTTAGGCAGAAGTTGAAGATGATGCTGCAGGCGAGGCAGCCCACACAAATAGCAGCA  1440
```

```
Sbjct  1443  ............................................................  1502

Query  1441  GTGACTGAGGAACTCATACAAGAAGCAGCTGCAGTAGAGCAAGAGGCCGTGGATACGGCC  1500
Sbjct  1503  ..................................C.........................  1562

Query  1501  AATGCCGAGCTGGACCACGCCGCATGGCCCTCCATTGTGGATACGACAGAGCGCCATGTT  1560
Sbjct  1563  ...............................................G............  1622

Query  1561  GAGGTCGAAGTGGAAGAACTCGACCAGCGTGCAGGGGAAGGGGTAGTGGAAACACCTCGA  1620
Sbjct  1623  ............................................................  1682

Query  1621  AACTCTATCAAAGTTTCAACACAGATCGGGGACGCGTTAATCGGCAGTTACCTGATCCTA  1680
Sbjct  1683  ........T........T..........................................  1742

Query  1681  TCACCCCAAGCAGTCCTACGCAGCGAAAAATTAGCCTGCATACATGATCTTGCAGAGCAG  1740
Sbjct  1743  ............................................................  1802

Query  1741  GTTAAGTTGGTCACACACTCTGGCCGTAGTGGTAGGTACGCCGTCGACAAATACNACGGA  1800
Sbjct  1803  ..C.................C..T..............................G.....  1862

Query  1801  AGAGTACTAGTCCCTACAGGAGTGGCTATAGACATTCAATCGTTCCAGGCTCTCAGTGAG  1860
Sbjct  1863  ...................................C........................  1922

Query  1861  AGCGCGACCCTTGTGTACAACGAACGCGAGTTCGTTAACAGGAAGCTGTGGCACATAGCA  1920
Sbjct  1923  ..T..........................................T..............  1982

Query  1921  GTATACGGGGCAGCACTCAATACTGATGAAGAAGGATACGAGAAGGTCCCGGTAGAGAGA  1980
Sbjct  1983  ............................................................  2042

Query  1981  GCAGAATCAGATTATGTGTTTGATGTAGACCAAAAAATGTGCCTaaaaaaaGAGCAGGCA  2040
Sbjct  2043  .................A............A.............C...............  2102

Query  2041  TCAGGTTGGGTACTCTGTGGCGAACTAGTCAACCCCCCATTCCACGAATTCGCATATGAA  2100
Sbjct  2103  .....A..................T...................................  2162

Query  2101  GGGCTCCGCACGAGACCGTCAGCACCCTACAAGGTTCATACAGTAGGTGTGTACGGAGTG  2160
Sbjct  2163  ........T..T......G.........................................  2222

Query  2161  CCAGGATCAGGCAAATCCGCAATAATCAAGAACACGGTCACCATGTCTGACCTAGTATTG  2220
Sbjct  2223  ............................................................  2282

Query  2221  AGTGGTAAGAAAGAGAACTGCTTAGAAATTATGAACGATGTACTTAAACACAGAGCTCTA  2280
Sbjct  2283  ............................................................  2342

Query  2281  CGTATCACAGCGAAGACCGTAGACTCAGTGTTATTAAACGGCGTGAAACACACGCCTAAC  2340
Sbjct  2343  .....T....................G........G........................  2402

Query  2341  ATACTATACATCGACGAAGCGTTCTCATGCCATGCAGGGACTCTGTTGGCCACTATAGCC  2400
Sbjct  2403  ............................................................  2462

Query  2401  ATAGTCAGGCCCAAACAGAAAGTGGTACTGTGCGGAGACCCGAAACAATGCGGATTCTTC  2460
Sbjct  2463  T...........................................................  2522

Query  2461  AATATGATGCAACTGAAAGTTAATTACAATCATGACATCTGCTCAGAAGTCTTCCACAAA  2520
```

```
Sbjct  2523  .......................C....................................  2582

Query  2521  AGTATCTCTAGACGGTGCACCCAGGATATCACGGCCATCGTTTCCAAATTACATTACCAG  2580
Sbjct  2583  ............................................T...............  2642

Query  2581  GACCGAATGAGGACCACAAACCCCGAAAAGGAGACATCATTATAGACACTACCGGCACT  2640
Sbjct  2643  ..................................................C.........  2702

Query  2641  ACCAAACCAGCCAAAACAGATCTGATTCTGACGTGCTTCAGGGGATGGGTGAAACAGTTG  2700
Sbjct  2703  ....................C.......................................  2762

Query  2701  CAGCAAGACTACAGAGGTAACGAAGTAATGACGGCTGCAGCGTCCCAAGGACTGACGAGG  2760
Sbjct  2763  ............................................................  2822

Query  2761  GCCTCCGTATATGCGGTTCGAACTAAAGTCAATGAGAACCCGCTATATGCACAGACCTCC  2820
Sbjct  2823  ............................................................  2882

Query  2821  GAGCACGTGAACGTGTTGTTAACACGCACAGAAAACAAGCTAGTATGGAAGACCTTGTCA  2880
Sbjct  2883  .......................................T....................  2942

Query  2881  ACAGATCCCTGGATTAAAACACTGACTAACCCACCTAGAGGGCACTATACCGCCACCATA  2940
Sbjct  2943  ............................................................  3002

Query  2941  GCAGAATGGGAAGCGGAACACCAGGGTATAATGAAGGCCATACAAGGGTATGCACCGCCC  3000
Sbjct  3003  ..............A...........C.................................  3062

Query  3001  GTGAACACCTTCATGAACAAAGTAAATGTGTGCTGGGCAAAGACACTTACGCCTGTGCTG  3060
Sbjct  3063  ...........T................................G..C..A.........  3122

Query  3061  GAAACTGCGGGTATCTCCCTGTCAGCAGAAGACTGGTCTGAACTGCTGCCCCCGTTTGCC  3120
Sbjct  3123  ..................T.........................................  3182

Query  3121  CAGGACGTGGCGTACTCACCCGAGGTGGCATTAAACATCATATGCACGAAAATGTATGGG  3180
Sbjct  3183  ...............................................A............  3242

Query  3181  TTTGACTTAGACACTGGTCTTTTTTCCAGGCCATCAGTGCCAATGACATACACCAAAGAC  3240
Sbjct  3243  ........G.....C.............A.........A.....................  3302

Query  3241  CATTGGGATAACAGAGTTGGAGGGAAAATGTATGGATTCAGCCAACAAGCATACGATCAG  3300
Sbjct  3303  ............................................................  3362

Query  3301  CTGGCAAGACGACATCCGTACCTTCGAGGTAGAGAGAAATCAGGAATGCAGATCGTAGTC  3360
Sbjct  3363  ...................................A........................  3422

Query  3361  ACTGAAATGCGTATCCAGCGCCCAAGATCGGATGCCAACATCATCCCGATCAACCGCAGG  3420
Sbjct  3423  .....G......................................................  3482

Query  3421  CTCCCTCACTCACTCGTAGCCACACACGAGTATAGGCGAGCTGCACGGGCCGAGGAATTC  3480
Sbjct  3483  ............................................G...............  3542

Query  3481  TTCACCACGACACGAGGGTACACTATGCTGCTGGTCTCTGAGTATAACATGAACTTACCA  3540
Sbjct  3543  ..........T.................................................  3602

Query  3541  AACAAGAAGATCACCTGGCTGGCTCCGATAGGGACGCAGGGGGCCCATCACACCGCCAAC  3600
```

```
Sbjct  3603  ............................................................  3662

Query  3601  CTAAACTTGGGGATACCACCTCTGCTGGGCAGTTTTGATGCGGTGGTTGTGAACATGCCG  3660
Sbjct  3663  ........................................T...................  3722

Query  3661  ACTCCATTCCGGAACCATCACTACCAGCAATGTGAAGACCACGCGATGAAACTCCAGATG  3720
Sbjct  3723  ................................C...........................  3782

Query  3721  CTGGCAGGCGACGCACTGAGGCACATTAAACCTGGCGGATCATTGTGGGTCAAGGCATAC  3780
Sbjct  3783  .............................G..............................  3842

Query  3781  GGCTACGCAGACCGGCACAGCGAGCACGTGGTCTTGGCATTGGCTAGAAAGTTTAAAAGC  3840
Sbjct  3843  .......................................C....................  3902

Query  3841  TTCAGAGTCACACAACCCTCATGCGTGACTTCCAACACCGAGGTGTTTCTCCACTTCTCA  3900
Sbjct  3903  ..T........G........G.....A................................G  3962

Query  3901  ATTTTTGACAATGGCAAACGCGCGATAGCCCTGCATTCAGCTAATAGGAAGGCTAACAGT  3960
Sbjct  3963  ............................................................  4022

Query  3961  ATCTTCCAAAACACCTTCTTACCGGCGGGCAGTGCACCGGCGTACAGAGTCAAACGTGGA  4020
Sbjct  4023  ..........................A....A............................  4082

Query  4021  GACATTTCGAACGCCCCAGAGGATGCAGTGGTCAATGCAGCAAACCAACAGGGAGTGAAG  4080
Sbjct  4083  ............................................................  4142

Query  4081  GGTGCTGGAGTTTGCGGTGCAATTTACCGTAAGTGGCCGGACGCTTTCGGTGATGTCGCT  4140
Sbjct  4143  ............................................................  4202

Query  4141  ACTCCAACCGGAACAGCAGTTTCGAAATCCGTCCAAGATAAATTGGTGATCCACGCTGTC  4200
Sbjct  4203  ............................................................  4262

Query  4201  GGCCCGAATTTCTCAAAATGTTCAGAAGAGGAAGGGGACAGAGACCTAGCATCTGCTTAC  4260
Sbjct  4263  ...........T................................................  4322

Query  4261  AGAGCTGCAGCAGAAATAGTGATGGATaaaaaaaTTACAACAGTGGCCGTCCCCTTACTC  4320
Sbjct  4323  .............C............A...........................C.....  4382

Query  4321  TCCACCGGCATTTATGCCGGAGGAAAAAACAGAGTAGAACAGTCACTCAACCATCTCTTC  4380
Sbjct  4383  ..............C........G....................................  4442

Query  4381  ACGGCATTCGACAATACTGATGCAGATGTGACCATATATTGCATGGACAAAACATGGGAA  4440
Sbjct  4443  ........T...................................................  4502

Query  4441  AAGAAGATTAAGGAGGCAATCGATCACCGGACTTCGGTTGAGATGGTGCAGGATGACGTG  4500
Sbjct  4503  ............................................................  4562

Query  4501  CAGTTGGAGGAGGAACTGGTACGAGTACACCCTTTGAGTAGTTTAGCAGGTAGGAAGGGT  4560
Sbjct  4563  ......................................C.................A...  4622

Query  4561  TACAGTACGGACAGCGGCCGAGTGTTTTCCTACCTGGAAGGTACCAAATTCCATCAGACT  4620
Sbjct  4623  ............................................................  4682

Query  4621  GCGGTGGACATAGCCGAAATGCAAGTGCTGTGGCCCGCCCTCAAAGAGTCTAATGAGCAA  4680
```

```
Sbjct  4683  ........T............................T.....................  4742

Query  4681  ATAGTGGCATACACCTTAGGAGAATCAATGGACCAGATACGTGGCAAGTGCCCGACAGAA  4740
Sbjct  4743  ............................................................  4802

Query  4741  GATACTGACGCCTCCACACCTCCACGGACTGTGCCGTGCCTCTGTCGATACGCCATGACA  4800
Sbjct  4803  ............................................................  4862

Query  4801  CCAGAGAGAGTGTACCGACTTAAATGCACGAACACTACCCAATTTACGGTTTGCTCATCT  4860
Sbjct  4863  ............................................C.....C.........  4922

Query  4861  TTTGAGTTGCCAAAGTATCACATTCAGGGAGTGCAGAGAGTAAAATGTGAAAGAATCATC  4920
Sbjct  4923  ............................................................  4982

Query  4921  ATCTTAGATCCCACTGTTCCACCAACTTACAAACGGCCATGCATCAGACGGTACCCCTCC  4980
Sbjct  4983  ............................................................  5042

Query  4981  ACAATCTCTTGTAACTCCTCTGAGGACTCCAGGAGCTTGTCTACTTTTTCTGTCAGCTCC  5040
Sbjct  5043  ........C...................................C...........  5102

Query  5041  GACTCCTCGATTGGTTCTCTGCCGGTCGGAGACACGAGACCCATTCCAGCCCCGAGGACC  5100
Sbjct  5103  .................CG..A..................T...........A......  5162

Query  5101  ATTTTCAGACCCGTCCCTGCCCCGAGAGCACCCGTGCTCAGAACCACACCGCCTCCTAAA  5160
Sbjct  5163  G...........................T.........T..T.............  5222

Query  5161  CCACCGCGCACATTCACCGTGCGTGCAGAAGTGCACCAAGCACCCCCTACACCTGTACCT  5220
Sbjct  5223  ..................................T........................  5282

Query  5221  CCACCCAGACCGAAGAGGGCTGCAAAGTTGGCTCGTGAGATGCACCCCGGGTTCACCTTC  5280
Sbjct  5283  .....T......................................................  5342

Query  5281  GGGGACTTCGGAGAGCACGAGGTTGAGGAGCTTACGGCCTCTCCCTTAACCTTCGGAGAT  5340
Sbjct  5343  ..A.................A.................G....................  5402

Query  5341  TTTGCTGAAGGAGAGATCCAGGGGATGGGAGTGGAGTTTGAATGACTAGGCAGAGCCGGC  5400
Sbjct  5403  ...................A....C.................................  5462

Query  5401  GGGTACATTTTTTCGTCAGACACGGGTCCAGGCCACCTACAGCAGAGATCCGTTTTACAA  5460
Sbjct  5463  ........................................A.....G............  5522

Query  5461  AATTGCACGGCAGAATGTATCTACGAACCGGCAAAACTAGAAAAAATTCATGCACCAAAG  5520
Sbjct  5523  ............................................................  5582

Query  5521  TTGGATAAAACCAAGGAAGATATCTTAAGGAGCAAGTACCAAATGAAACCGTCTGAAGCA  5580
Sbjct  5583  ............................................................  5642

Query  5581  AACAAAAGCAGGTACCAATCTAGAAAAGTAGAAAATATGAAAGCAGAGATCGTAGGTAGA  5640
Sbjct  5643  ........T......................................T.........  5702

Query  5641  CTCTTGGACGGACTGGGGGAGTATCTGGGCACCGAGCATCCAGTTGAATGCTACCGAATA  5700
Sbjct  5703  ..............A.............................................  5762

Query  5701  ACGTACCCGGTGCCTATATACTCAACTAGTGACCTCAGAGGTCTGTCTAGTGCCAAAACA  5760
```

```
Sbjct  5763  .....T....C...................AT..G.........................  5822

Query  5761  GCTGTTAGAGCTTGCAATGCATTTTTGGAAGCTAATTTTCCATCAGTCACTTCATATAAA  5820
Sbjct  5823  .....C......................................................  5882

Query  5821  ATTACTGATGAATACGACGCATACCTAGATATGGTAGATGGATCAGAGAGCTGTCTGGAC  5880
Sbjct  5883  ............................................................  5942

Query  5881  AGATCCTCCTTTTCGCCGTCTAGATTGCGTAGCTTTCCAAAAACACACTCATACTTGGAC  5940
Sbjct  5943  .................A...........C..............................  6002

Query  5941  CCACAGATCAACAGTGCGGTACCGTCACCATTCCAAAACACCTTACAAAATGTATTGGCA  6000
Sbjct  6003  ..G.........................................................  6062

Query  6001  GCGGCCACCAAAAGAAACTGTAATGTCACACAGATGAGAGAACTACCAACATATGATTCT  6060
Sbjct  6063  ...............................................G............  6122

Query  6061  GCAGTGCTAAATGTAGAGGCCTTCAGGAAATATGCGTGCAAGCCAGACGTATGGGATGAG  6120
Sbjct  6123  ............................................................  6182

Query  6121  TACAGGGATAATCCGATTTGCATAACCACCGAAAATGTCACCACTTACGTCGCCAAGTTG  6180
Sbjct  6183  ..............A...................................T..T......  6242

Query  6181  AAAGGACCGAAAGCTGCGGCCTTGTTTGCAAAAACACATAACCTGATACCACTACACCAA  6240
Sbjct  6243  ............................................................  6302

Query  6241  GTTCCTATGGACAAATTCACGGTAGATATGAAGAGAGATGTCAAAGTCACGCCCGGAACC  6300
Sbjct  6303  ............................................................  6362

Query  6301  AAGCACACCGAAGAGAGACCAAAGGTACAGGTGATTCAAGCGGCAGAGCCACTAGCCACT  6360
Sbjct  6363  ............................................................  6422

Query  6361  GCCTACCTCTGCGGAATTCACCGTGAATTGGTGCGCCGTCTCAACAACGCGCTTTTCCCA  6420
Sbjct  6423  .....................................................C.....T  6482

Query  6421  AACATCCACACTTTGTTTGATATGTCCGCAGAGGATTTCGATGCAATCATAGCGGAACAT  6480
Sbjct  6483  ..T...................................T.....................  6542

Query  6481  TTTAAGCACGGTGACCATGTGTTGGAAACGGATATAGCCTCTTTTGACAAAAGTCAAGAT  6540
Sbjct  6543  .................C..........................................  6602

Query  6541  GATTCCATGGCACTCACTGCGTTAATGATCCTTGAGGACCTGGGAGTAGACCAAAACCTA  6600
Sbjct  6603  ............................................................  6662

Query  6601  ATGAATTTGATAGAGGCTGCATTCGGGGAAATCGTGAGTACACACTTGCCCACAGGTACT  6660
Sbjct  6663  ............................................................  6722

Query  6661  AGATTCAAATTTGGAGCTATGATGAAGTCTGGAATGTTTTTGACGCTGTTCGTCAATACA  6720
Sbjct  6723  .......G.....................C...............T..............  6782

Query  6721  ATTCTTAATGTGGTTATTGCGTGCCGAGTGTTGGAGGATCAATTGGCGCAGTCGCCGTGG  6780
Sbjct  6783  ........C..................................................C  6842

Query  6781  CCTGCTTTCATAGGAGATGACAACATAATCCATGGTATAATATCAGACAAATTGATGGCA  6840
```

```
Sbjct  6843  G...............................C..........................G  6902

Query  6841  GATAGATGTGCCACCTGGATGAACATGGAGGTCAAGATACTGGACTCTATAGTTGGAATA  6900
Sbjct  6903  ............................................T..C............  6962

Query  6901  CGGCCACCTTACTTCTGTGGAGGATTTATTGTATGTGACGATGTAACAGGTACAGCCTGC  6960
Sbjct  6963  ...........T................................................  7022

Query  6961  CGCGTCGCAGACCCACTGAAGAGATTGTTCAAGCTAGGTAAGCCATTGCCACTTGACGAT  7020
Sbjct  7023  ........................C...................................  7082

Query  7021  GGCCAAGATGAAGACAGAAGACGTGCATTACATGATGAAGTGAAAACCTGGTCGCGCGTA  7080
Sbjct  7083  .............................G..............................  7142

Query  7081  GGGCTGCGACACAGAGTGTGTGAAGCCATCGAAGACCGTTATGCCGTCCACTCATCAGAA  7140
Sbjct  7143  ..................................................T.........  7202

Query  7141  CTAGTTTTATTGGCACTGACTACTCTGTCTAAGAACTTGAAGTCCTTCAGAAACATAAGA  7200
Sbjct  7203  ..............G.............................................  7262

Query  7201  GGGAAACCAATACATCTCTACGGTGGTCCTAAATAG  7236
Sbjct  7263  ....................................  7298



>Barmah Forest virus isolate ARBO318, complete genome
Sequence ID: MW556197.1 Length: 11550
Range 1: 50 to 7285

Score:12578 bits(6811), Expect:0.0,
Identities:7094/7236(98%),  Gaps:0/7236(0%), Strand: Plus/Plus

Query  1     ATGGCGAAACCAGTTGTGAAGATCGACGTGGAACCTGAAAGCCATTTCGCTAAGCAGGTC  60
Sbjct  50    ...........G...................................T............  109

Query  61    CAGAGTTGCTTCCCGCAGTTTGAGATCGAAGCAGTGCAGACCACACCAAACGATCATGCA  120
Sbjct  110   .........................................G..................  169

Query  121   CACGCGAGGGCGTTTTCGCACCTTGCTACGAAGCTCATAGAAATGGAGACAGCAAAAGAT  180
Sbjct  170   ............................................................  229

Query  181   CAGATCATCCTCGATATCGGAAGTGCACCCGCGAGGAGACTGTATTCAGAACACAAGTAC  240
Sbjct  230   ............................................................  289

Query  241   CACTGTGTTTGCCCAATGAAGTGCACGGAAGATCCAGAGAGAATGCTAGGATATGCACGT  300
Sbjct  290   ............................................................  349

Query  301   AAGTTGATCGCAGGCTCTGCGAAAGGGAAGGCAGAAAAGTTACGCGATCTCAGGGATGTC  360
Sbjct  350   ............................................................  409

Query  361   TTGGCTACGCCAGACATCGAGACGCAGTCGCTATGTCTCCACACAGACGCATCCTGCAGA  420
Sbjct  410   ............................................................  469
```

```
Query  421   TACCGCGGTGATGTTGCCGTGTATCAAGACGTGTATGCCATTGACGCACCTACCACGCTG  480
Sbjct  470   ..................................................C.........  529

Query  481   TACCACCAAGCGTTAAAGGGCGTCAGGACCGCATATTGGATAGGCTTTGATACAACGCCG  540
Sbjct  530   ...........................................................A  589

Query  541   TTCATGTACGATGCACTAGCAGGAGCTTACCCGCTCTACTCCACAAACTGGGCTGATGAG  600
Sbjct  590   .........A...........................................C......  649

Query  601   CAAGTGCTCGAGTCCAGAAACATTGGGCTATGTTCAGACAAAGTTTCTGAAGGGGGAAAG  660
Sbjct  650   ............................................................  709

Query  661   AAAGGGAGATCAATCCTCAGGAAGAAGTTCTTGAAGCAGTCAGACAGAGTCATGTTCTCT  720
Sbjct  710   ............................................................  769

Query  721   GTCGGCTCGACGTTGTATACGGAAAGCCGTAAATTACTGCAAAGTTGGCACCTGCCATCC  780
Sbjct  770   ............................................C...............  829

Query  781   ACATTCCATCTCAAAGGCAAATCTTCGTTCACGTGCCGCTGCGACACTATCGTCAGCTGC  840
Sbjct  830   ................................A..............C............  889

Query  841   GAAGGGTATGTTCTGAAGAAAATTACAATGTGTCCTGGAGTGACAGGCAAACCGATAGGA  900
Sbjct  890   ................................C..C........................  949

Query  901   TATGCCGTCACCCATCACAAAGAAGGATTCGTAGTCGGAAAAGTCACAGATACCATTCGC  960
Sbjct  950   ....................G.............................C.........  1009

Query  961   GGCGAGAGAGTCTCCTTCGCCGTGTGTACTTATGTACCAACAACACTCTGCGACCAGATG  1020
Sbjct  1010  ............................................................  1069

Query  1021  ACCGGGATCCTAGCAACAGAAGTAACAGCCGATGATGCCCAGAAACTGCTGGTGGGTTTG  1080
Sbjct  1070  ........T...................................................  1129

Query  1081  AACCAGAGAATAGTAGTTAATGGTAGGACCCAGAGAAATACCAATACTATGAAGAACTAC  1140
Sbjct  1130  ............................................C...............  1189

Query  1141  CTGCTACCACTGGTTGCACAAGCGCTAGCAAAATGGGCGAAGGAAGCAAAACAGGATATG  1200
Sbjct  1190  ......................................A.....................  1249

Query  1201  GAAGATGAAAGACCCCTGAACGAACGCCAACGAACGCTAACGTGCCTCTGCTGCTGGGCA  1260
Sbjct  1250  ............................................................  1309

Query  1261  TTTAAGCGAAACAAACGCCACGCCATTTACAAGAGACCAGACACACAGAGTATAGTCAAG  1320
Sbjct  1310  ............................................................  1369

Query  1321  GTCCCTTGCGAATTCACAAGCTTTCCTTTGGTCAGCCTGTGGTCCGCTGGGATGTCTATA  1380
Sbjct  1370  ............................................................  1429

Query  1381  TCTCTTAGGCAGAAGTTGAAGATGATGCTGCAGGCGAGGCAGCCCACACAAATAGCAGCA  1440
Sbjct  1430  ............................................................  1489

Query  1441  GTGACTGAGGAACTCATACAAGAAGCAGCTGCAGTAGAGCAAGAGGCCGTGGATACGGCC  1500
Sbjct  1490  ..................................C.........................  1549
```

```
Query  1501  AATGCCGAGCTGGACCACGCCGCATGGCCCTCCATTGTGGATACGACAGAGCGCCATGTT  1560
Sbjct  1550  ...............................................G............  1609

Query  1561  GAGGTCGAAGTGGAAGAACTCGACCAGCGTGCAGGGGAAGGGGTAGTGGAAACACCTCGA  1620
Sbjct  1610  ............................................................  1669

Query  1621  AACTCTATCAAAGTTTCAACACAGATCGGGGACGCGTTAATCGGCAGTTACCTGATCCTA  1680
Sbjct  1670  ........T........T..........................................  1729

Query  1681  TCACCCCAAGCAGTCCTACGCAGCGAAAAATTAGCCTGCATACATGATCTTGCAGAGCAG  1740
Sbjct  1730  ............................................................  1789

Query  1741  GTTAAGTTGGTCACACACTCTGGCCGTAGTGGTAGGTACGCCGTCGACAAATACNACGGA  1800
Sbjct  1790  ..C.................C..T..............................G.....  1849

Query  1801  AGAGTACTAGTCCCTACAGGAGTGGCTATAGACATTCAATCGTTCCAGGCTCTCAGTGAG  1860
Sbjct  1850  ...................................C........................  1909

Query  1861  AGCGCGACCCTTGTGTACAACGAACGCGAGTTCGTTAACAGGAAGCTGTGGCACATAGCA  1920
Sbjct  1910  ..T..........................................T..............  1969

Query  1921  GTATACGGGGCAGCACTCAATACTGATGAAGAAGGATACGAGAAGGTCCCGGTAGAGAGA  1980
Sbjct  1970  ............................................................  2029

Query  1981  GCAGAATCAGATTATGTGTTTGATGTAGACCAAAAAATGTGCCTaaaaaaaGAGCAGGCA  2040
Sbjct  2030  .................A............A.............C...............  2089

Query  2041  TCAGGTTGGGTACTCTGTGGCGAACTAGTCAACCCCCCATTCCACGAATTCGCATATGAA  2100
Sbjct  2090  .....A..................T...................................  2149

Query  2101  GGGCTCCGCACGAGACCGTCAGCACCCTACAAGGTTCATACAGTAGGTGTGTACGGAGTG  2160
Sbjct  2150  ........T..T................................................  2209

Query  2161  CCAGGATCAGGCAAATCCGCAATAATCAAGAACACGGTCACCATGTCTGACCTAGTATTG  2220
Sbjct  2210  ............................................................  2269

Query  2221  AGTGGTAAGAAAGAGAACTGCTTAGAAATTATGAACGATGTACTTAAACACAGAGCTCTA  2280
Sbjct  2270  ............................................................  2329

Query  2281  CGTATCACAGCGAAGACCGTAGACTCAGTGTTATTAAACGGCGTGAAACACACGCCTAAC  2340
Sbjct  2330  .....T.............................G........................  2389

Query  2341  ATACTATACATCGACGAAGCGTTCTCATGCCATGCAGGGACTCTGTTGGCCACTATAGCC  2400
Sbjct  2390  ............................................................  2449

Query  2401  ATAGTCAGGCCCAAACAGAAAGTGGTACTGTGCGGAGACCCGAAACAATGCGGATTCTTC  2460
Sbjct  2450  T...........................................................  2509

Query  2461  AATATGATGCAACTGAAAGTTAATTACAATCATGACATCTGCTCAGAAGTCTTCCACAAA  2520
Sbjct  2510  .......................C....................................  2569

Query  2521  AGTATCTCTAGACGGTGCACCCAGGATATCACGGCCATCGTTTCCAAATTACATTACCAG  2580
Sbjct  2570  ............................................T...............  2629
```

```
Query  2581  GACCGAATGAGGACCACAAACCCCCGAAAAGGAGACATCATTATAGACACTACCGGCACT  2640
Sbjct  2630  ..................................................C.........  2689

Query  2641  ACCAAACCAGCCAAAACAGATCTGATTCTGACGTGCTTCAGGGGATGGGTGAAACAGTTG  2700
Sbjct  2690  ....................CT......................................  2749

Query  2701  CAGCAAGACTACAGAGGTAACGAAGTAATGACGGCTGCAGCGTCCCAAGGACTGACGAGG  2760
Sbjct  2750  ............................................................  2809

Query  2761  GCCTCCGTATATGCGGTTCGAACTAAAGTCAATGAGAACCCGCTATATGCACAGACCTCC  2820
Sbjct  2810  .........................................A..................  2869

Query  2821  GAGCACGTGAACGTGTTGTTAACACGCACAGAAAACAAGCTAGTATGGAAGACCTTGTCA  2880
Sbjct  2870  .......................................T....................  2929

Query  2881  ACAGATCCCTGGATTAAAACACTGACTAACCCACCTAGAGGGCACTATACCGCCACCATA  2940
Sbjct  2930  ............................................................  2989

Query  2941  GCAGAATGGGAAGCGGAACACCAGGGTATAATGAAGGCCATACAAGGGTATGCACCGCCC  3000
Sbjct  2990  ..............A.............................................  3049

Query  3001  GTGAACACCTTCATGAACAAAGTAAATGTGTGCTGGGCAAAGACACTTACGCCTGTGCTG  3060
Sbjct  3050  ...........T................................G..C..A.........  3109

Query  3061  GAAACTGCGGGTATCTCCCTGTCAGCAGAAGACTGGTCTGAACTGCTGCCCCCGTTTGCC  3120
Sbjct  3110  ..................T.........................................  3169

Query  3121  CAGGACGTGGCGTACTCACCCGAGGTGGCATTAAACATCATATGCACGAAAATGTATGGG  3180
Sbjct  3170  ...............................................A............  3229

Query  3181  TTTGACTTAGACACTGGTCTTTTTTCCAGGCCATCAGTGCCAATGACATACACCAAAGAC  3240
Sbjct  3230  ........G.....C.............A.........A.....................  3289

Query  3241  CATTGGGATAACAGAGTTGGAGGGAAAATGTATGGATTCAGCCAACAAGCATACGATCAG  3300
Sbjct  3290  .....................................................T......  3349

Query  3301  CTGGCAAGACGACATCCGTACCTTCGAGGTAGAGAGAAATCAGGAATGCAGATCGTAGTC  3360
Sbjct  3350  ...................................A........................  3409

Query  3361  ACTGAAATGCGTATCCAGCGCCCAAGATCGGATGCCAACATCATCCCGATCAACCGCAGG  3420
Sbjct  3410  .....G......................................................  3469

Query  3421  CTCCCTCACTCACTCGTAGCCACACACGAGTATAGGCGAGCTGCACGGCCGAGGAATTC  3480
Sbjct  3470  ............................................G...............  3529

Query  3481  TTCACCACGACACGAGGGTACACTATGCTGCTGGTCTCTGAGTATAACATGAACTTACCA  3540
Sbjct  3530  ..........T.................................................  3589

Query  3541  AACAAGAAGATCACCTGGCTGGCTCCGATAGGGACGCAGGGGGCCCATCACACCGCCAAC  3600
Sbjct  3590  ............................................................  3649

Query  3601  CTAAACTTGGGGATACCACCTCTGCTGGGCAGTTTTGATGCGGTGGTTGTGAACATGCCG  3660
Sbjct  3650  ........................................T...................  3709
```

```
Query  3661  ACTCCATTCCGGAACCATCACTACCAGCAATGTGAAGACCACGCGATGAAACTCCAGATG  3720
Sbjct  3710  ................................C...........................  3769

Query  3721  CTGGCAGGCGACGCACTGAGGCACATTAAACCTGGCGGATCATTGTGGGTCAAGGCATAC  3780
Sbjct  3770  .............................G..............................  3829

Query  3781  GGCTACGCAGACCGGCACAGCGAGCACGTGGTCTTGGCATTGGCTAGAAAGTTTAAAAGC  3840
Sbjct  3830  .......................................C.............C......  3889

Query  3841  TTCAGAGTCACACAACCCTCATGCGTGACTTCCAACACCGAGGTGTTTCTCCACTTCTCA  3900
Sbjct  3890  ...........G........G.....A................................G  3949

Query  3901  ATTTTTGACAATGGCAAACGCGCGATAGCCCTGCATTCAGCTAATAGGAAGGCTAACAGT  3960
Sbjct  3950  ............................................................  4009

Query  3961  ATCTTCCAAAACACCTTCTTACCGGCGGGCAGTGCACCGGCGTACAGAGTCAAACGTGGA  4020
Sbjct  4010  ..........................A....A............................  4069

Query  4021  GACATTTCGAACGCCCCAGAGGATGCAGTGGTCAATGCAGCAAACCAACAGGGAGTGAAG  4080
Sbjct  4070  ............................................................  4129

Query  4081  GGTGCTGGAGTTTGCGGTGCAATTTACCGTAAGTGGCCGGACGCTTTCGGTGATGTCGCT  4140
Sbjct  4130  ............................................................  4189

Query  4141  ACTCCAACCGGAACAGCAGTTTCGAAATCCGTCCAAGATAAATTGGTGATCCACGCTGTC  4200
Sbjct  4190  ............................................................  4249

Query  4201  GGCCCGAATTTCTCAAAATGTTCAGAAGAGGAAGGGGACAGAGACCTAGCATCTGCTTAC  4260
Sbjct  4250  ...........T................................................  4309

Query  4261  AGAGCTGCAGCAGAAATAGTGATGGATaaaaaaaTTACAACAGTGGCCGTCCCCTTACTC  4320
Sbjct  4310  ..........................A...........................C.....  4369

Query  4321  TCCACCGGCATTTATGCCGGAGGAAAAAACAGAGTAGAACAGTCACTCAACCATCTCTTC  4380
Sbjct  4370  ..............C........G....................................  4429

Query  4381  ACGGCATTCGACAATACTGATGCAGATGTGACCATATATTGCATGGACAAAACATGGGAA  4440
Sbjct  4430  ........T...................................................  4489

Query  4441  AAGAAGATTAAGGAGGCAATCGATCACCGGACTTCGGTTGAGATGGTGCAGGATGACGTG  4500
Sbjct  4490  ............................................................  4549

Query  4501  CAGTTGGAGGAGGAACTGGTACGAGTACACCCTTTGAGTAGTTTAGCAGGTAGGAAGGGT  4560
Sbjct  4550  ......................................C.................A...  4609

Query  4561  TACAGTACGGACAGCGGCCGAGTGTTTTCCTACCTGGAAGGTACCAAATTCCATCAGACT  4620
Sbjct  4610  ............................................................  4669

Query  4621  GCGGTGGACATAGCCGAAATGCAAGTGCTGTGGCCCGCCCTCAAAGAGTCTAATGAGCAA  4680
Sbjct  4670  ........T.............................T.....................  4729

Query  4681  ATAGTGGCATACACCTTAGGAGAATCAATGGACCAGATACGTGGCAAGTGCCCGACAGAA  4740
Sbjct  4730  ............................................................  4789
```

```
Query  4741  GATACTGACGCCTCCACACCTCCACGGACTGTGCCGTGCCTCTGTCGATACGCCATGACA  4800
Sbjct  4790  ............................................................  4849

Query  4801  CCAGAGAGAGTGTACCGACTTAAATGCACGAACACTACCCAATTTACGGTTTGCTCATCT  4860
Sbjct  4850  ............................................C.....C.........  4909

Query  4861  TTTGAGTTGCCAAAGTATCACATTCAGGGAGTGCAGAGAGTAAAATGTGAAAGAATCATC  4920
Sbjct  4910  ............................................................  4969

Query  4921  ATCTTAGATCCCACTGTTCCACCAACTTACAAACGGCCATGCATCAGACGGTACCCCTCC  4980
Sbjct  4970  ............................................................  5029

Query  4981  ACAATCTCTTGTAACTCCTCTGAGGACTCCAGGAGCTTGTCTACTTTTTCTGTCAGCTCC  5040
Sbjct  5030  ........C......................................C............  5089

Query  5041  GACTCCTCGATTGGTTCTCTGCCGGTCGGAGACACGAGACCCATTCCAGCCCCGAGGACC  5100
Sbjct  5090  .................CG..A...................T...........A......  5149

Query  5101  ATTTTCAGACCCGTCCCTGCCCCGAGAGCACCCGTGCTCAGAACCACACCGCCTCCTAAA  5160
Sbjct  5150  G...............................T..........T................  5209

Query  5161  CCACCGCGCACATTCACCGTGCGTGCAGAAGTGCACCAAGCACCCCCTACACCTGTACCT  5220
Sbjct  5210  ...................................T........................  5269

Query  5221  CCACCCAGACCGAAGAGGGCTGCAAAGTTGGCTCGTGAGATGCACCCCGGGTTCACCTTC  5280
Sbjct  5270  .....T......................................................  5329

Query  5281  GGGGACTTCGGAGAGCACGAGGTTGAGGAGCTTACGGCCTCTCCCTTAACCTTCGGAGAT  5340
Sbjct  5330  ..A..................A.................G....................  5389

Query  5341  TTTGCTGAAGGAGAGATCCAGGGGATGGGAGTGGAGTTTGAATGACTAGGCAGAGCCGGC  5400
Sbjct  5390  ....................A....C..................................  5449

Query  5401  GGGTACATTTTTTCGTCAGACACGGGTCCAGGCCACCTACAGCAGAGATCCGTTTTACAA  5460
Sbjct  5450  .........................................A.....G............  5509

Query  5461  AATTGCACGGCAGAATGTATCTACGAACCGGCAAAACTAGAAAAAAATTCATGCACCAAAG  5520
Sbjct  5510  ............................................................  5569

Query  5521  TTGGATAAAACCAAGGAAGATATCTTAAGGAGCAAGTACCAAATGAAACCGTCTGAAGCA  5580
Sbjct  5570  ............................................................  5629

Query  5581  AACAAAAGCAGGTACCAATCTAGAAAAGTAGAAAATATGAAAGCAGAGATCGTAGGTAGA  5640
Sbjct  5630  ........T.........................................T.........  5689

Query  5641  CTCTTGGACGGACTGGGGGAGTATCTGGGCACCGAGCATCCAGTTGAATGCTACCGAATA  5700
Sbjct  5690  ..............A.............................................  5749

Query  5701  ACGTACCCGGTGCCTATATACTCAACTAGTGACCTCAGAGGTCTGTCTAGTGCCAAAACA  5760
Sbjct  5750  ...C.T....C...................AT..G.........................  5809

Query  5761  GCTGTTAGAGCTTGCAATGCATTTTTGGAAGCTAATTTTCCATCAGTCACTTCATATAAA  5820
Sbjct  5810  ................................G...........................  5869
```

```
Query  5821  ATTACTGATGAATACGACGCATACCTAGATATGGTAGATGGATCAGAGAGCTGTCTGGAC  5880
Sbjct  5870  ..................T.........................................  5929

Query  5881  AGATCCTCCTTTTCGCCGTCTAGATTGCGTAGCTTTCCAAAAACACACTCATACTTGGAC  5940
Sbjct  5930  .................A...........C..............................  5989

Query  5941  CCACAGATCAACAGTGCGGTACCGTCACCATTCCAAAACACCTTACAAAATGTATTGGCA  6000
Sbjct  5990  ..G.........................................................  6049

Query  6001  GCGGCCACCAAAAGAAACTGTAATGTCACACAGATGAGAGAACTACCAACATATGATTCT  6060
Sbjct  6050  ...............................................G............  6109

Query  6061  GCAGTGCTAAATGTAGAGGCCTTCAGGAAATATGCGTGCAAGCCAGACGTATGGGATGAG  6120
Sbjct  6110  ............................................................  6169

Query  6121  TACAGGGATAATCCGATTTGCATAACCACCGAAAATGTCACCACTTACGTCGCCAAGTTG  6180
Sbjct  6170  ..............A...................................T..T......  6229

Query  6181  AAAGGACCGAAAGCTGCGGCCTTGTTTGCAAAAACACATAACCTGATACCACTACACCAA  6240
Sbjct  6230  ............................................................  6289

Query  6241  GTTCCTATGGACAAATTCACGGTAGATATGAAGAGAGATGTCAAAGTCACGCCCGGAACC  6300
Sbjct  6290  ............................................................  6349

Query  6301  AAGCACACCGAAGAGAGACCAAAGGTACAGGTGATTCAAGCGGCAGAGCCACTAGCCACT  6360
Sbjct  6350  ............................................................  6409

Query  6361  GCCTACCTCTGCGGAATTCACCGTGAATTGGTGCGCCGTCTCAACAACGCGCTTTTCCCA  6420
Sbjct  6410  .....................................................C.....T  6469

Query  6421  AACATCCACACTTTGTTTGATATGTCCGCAGAGGATTTCGATGCAATCATAGCGGAACAT  6480
Sbjct  6470  ..T...................................T.....................  6529

Query  6481  TTTAAGCACGGTGACCATGTGTTGGAAACGGATATAGCCTCTTTTGACAAAAGTCAAGAT  6540
Sbjct  6530  .................C..........................................  6589

Query  6541  GATTCCATGGCACTCACTGCGTTAATGATCCTTGAGGACCTGGGAGTAGACCAAAACCTA  6600
Sbjct  6590  ............................................................  6649

Query  6601  ATGAATTTGATAGAGGCTGCATTCGGGGAAATCGTGAGTACACACTTGCCCACAGGTACT  6660
Sbjct  6650  ............................................................  6709

Query  6661  AGATTCAAATTTGGAGCTATGATGAAGTCTGGAATGTTTTTGACGCTGTTCGTCAATACA  6720
Sbjct  6710  .......G.....................C...............T..............  6769

Query  6721  ATTCTTAATGTGGTTATTGCGTGCCGAGTGTTGGAGGATCAATTGGCGCAGTCGCCGTGG  6780
Sbjct  6770  ........C..................................................C  6829

Query  6781  CCTGCTTTCATAGGAGATGACAACATAATCCATGGTATAATATCAGACAAATTGATGGCA  6840
Sbjct  6830  G......................T...................................G  6889

Query  6841  GATAGATGTGCCACCTGGATGAACATGGAGGTCAAGATACTGGACTCTATAGTTGGAATA  6900
Sbjct  6890  ............................................T..C............  6949
```

```
Query  6901  CGGCCACCTTACTTCTGTGGAGGATTTATTGTATGTGACGATGTAACAGGTACAGCCTGC  6960
Sbjct  6950  ...........T................................................  7009

Query  6961  CGCGTCGCAGACCCACTGAAGAGATTGTTCAAGCTAGGTAAGCCATTGCCACTTGACGAT  7020
Sbjct  7010  ........................C...................................  7069

Query  7021  GGCCAAGATGAAGACAGAAGACGTGCATTACATGATGAAGTGAAAACCTGGTCGCGCGTA  7080
Sbjct  7070  .............................G..............................  7129

Query  7081  GGGCTGCGACACAGAGTGTGTGAAGCCATCGAAGACCGTTATGCCGTCCACTCATCAGAA  7140
Sbjct  7130  ..................................................T.........  7189

Query  7141  CTAGTTTTATTGGCACTGACTACTCTGTCTAAGAACTTGAAGTCCTTCAGAAACATAAGA  7200
Sbjct  7190  .........C....G.............................................  7249

Query  7201  GGGAAACCAATACATCTCTACGGTGGTCCTAAATAG  7236
Sbjct  7250  ....................................  7285
```

>Barmah Forest virus isolate SW105961, complete genome
Sequence ID: MN689047.1 Length: 11486
Range 1: 54 to 7289

Score:12578 bits(6811), Expect:0.0,
Identities:7094/7236(98%),  Gaps:0/7236(0%), Strand: Plus/Plus

```
Query  1     ATGGCGAAACCAGTTGTGAAGATCGACGTGGAACCTGAAAGCCATTTCGCTAAGCAGGTC  60
Sbjct  54    ...........G...................................T............  113

Query  61    CAGAGTTGCTTCCCGCAGTTTGAGATCGAAGCAGTGCAGACCACACCAAACGATCATGCA  120
Sbjct  114   .........................................G..................  173

Query  121   CACGCGAGGGCGTTTTCGCACCTTGCTACGAAGCTCATAGAAATGGAGACAGCAAAAGAT  180
Sbjct  174   ............................................................  233

Query  181   CAGATCATCCTCGATATCGGAAGTGCACCCGCGAGGAGACTGTATTCAGAACACAAGTAC  240
Sbjct  234   ............................................................  293

Query  241   CACTGTGTTTGCCCAATGAAGTGCACGGAAGATCCAGAGAGAATGCTAGGATATGCACGT  300
Sbjct  294   ............................................................  353

Query  301   AAGTTGATCGCAGGCTCTGCGAAAGGGAAGGCAGAAAAGTTACGCGATCTCAGGGATGTC  360
Sbjct  354   ............................................................  413

Query  361   TTGGCTACGCCAGACATCGAGACGCAGTCGCTATGTCTCCACACAGACGCATCCTGCAGA  420
Sbjct  414   ............................................................  473

Query  421   TACCGCGGTGATGTTGCCGTGTATCAAGACGTGTATGCCATTGACGCACCTACCACGCTG  480
Sbjct  474   ..................................................C.........  533

Query  481   TACCACCAAGCGTTAAAGGGCGTCAGGACCGCATATTGGATAGGCTTTGATACAACGCCG  540
Sbjct  534   ...........................................................A  593
```

```
Query  541   TTCATGTACGATGCACTAGCAGGAGCTTACCCGCTCTACTCCACAAACTGGGCTGATGAG  600
Sbjct  594   .........A...........................................C......  653

Query  601   CAAGTGCTCGAGTCCAGAAACATTGGGCTATGTTCAGACAAAGTTTCTGAAGGGGGAAAG  660
Sbjct  654   ............................................................  713

Query  661   AAAGGGAGATCAATCCTCAGGAAGAAGTTCTTGAAGCAGTCAGACAGAGTCATGTTCTCT  720
Sbjct  714   ............................................................  773

Query  721   GTCGGCTCGACGTTGTATACGGAAAGCCGTAAATTACTGCAAAGTTGGCACCTGCCATCC  780
Sbjct  774   ............................................C...............  833

Query  781   ACATTCCATCTCAAAGGCAAATCTTCGTTCACGTGCCGCTGCGACACTATCGTCAGCTGC  840
Sbjct  834   ................................A..............C............  893

Query  841   GAAGGGTATGTTCTGAAGAAAATTACAATGTGTCCTGGAGTGACAGGCAAACCGATAGGA  900
Sbjct  894   ................................C..C........................  953

Query  901   TATGCCGTCACCCATCACAAAGAAGGATTCGTAGTCGGAAAAGTCACAGATACCATTCGC  960
Sbjct  954   ..............C.....G.............................C.........  1013

Query  961   GGCGAGAGAGTCTCCTTCGCCGTGTGTACTTATGTACCAACAACACTCTGCGACCAGATG  1020
Sbjct  1014  ............................................................  1073

Query  1021  ACCGGGATCCTAGCAACAGAAGTAACAGCCGATGATGCCCAGAAACTGCTGGTGGGTTTG  1080
Sbjct  1074  ........T...................................................  1133

Query  1081  AACCAGAGAATAGTAGTTAATGGTAGGACCCAGAGAAATACCAATACTATGAAGAACTAC  1140
Sbjct  1134  ............................................C...............  1193

Query  1141  CTGCTACCACTGGTTGCACAAGCGCTAGCAAAATGGGCGAAGGAAGCAAAACAGGATATG  1200
Sbjct  1194  ......................................A.....................  1253

Query  1201  GAAGATGAAAGACCCCTGAACGAACGCCAACGAACGCTAACGTGCCTCTGCTGCTGGGCA  1260
Sbjct  1254  ............................................................  1313

Query  1261  TTTAAGCGAAACAAACGCCACGCCATTTACAAGAGACCAGACACACAGAGTATAGTCAAG  1320
Sbjct  1314  ............................................................  1373

Query  1321  GTCCCTTGCGAATTCACAAGCTTTCCTTTGGTCAGCCTGTGGTCCGCTGGGATGTCTATA  1380
Sbjct  1374  ............................................................  1433

Query  1381  TCTCTTAGGCAGAAGTTGAAGATGATGCTGCAGGCGAGGCAGCCCACACAAATAGCAGCA  1440
Sbjct  1434  ............................................................  1493

Query  1441  GTGACTGAGGAACTCATACAAGAAGCAGCTGCAGTAGAGCAAGAGGCCGTGGATACGGCC  1500
Sbjct  1494  ..................................C.........................  1553

Query  1501  AATGCCGAGCTGGACCACGCCGCATGGCCCTCCATTGTGGATACGACAGAGCGCCATGTT  1560
Sbjct  1554  ...............................................G............  1613

Query  1561  GAGGTCGAAGTGGAAGAACTCGACCAGCGTGCAGGGGAAGGGGTAGTGGAAACACCTCGA  1620
Sbjct  1614  ............................................................  1673
```

```
Query  1621  AACTCTATCAAAGTTTCAACACAGATCGGGGACGCGTTAATCGGCAGTTACCTGATCCTA  1680
Sbjct  1674  ........T........T..........................................  1733

Query  1681  TCACCCCAAGCAGTCCTACGCAGCGAAAAATTAGCCTGCATACATGATCTTGCAGAGCAG  1740
Sbjct  1734  ............................................................  1793

Query  1741  GTTAAGTTGGTCACACACTCTGGCCGTAGTGGTAGGTACGCCGTCGACAAATACNACGGA  1800
Sbjct  1794  ..C.................C..T..............................G.....  1853

Query  1801  AGAGTACTAGTCCCTACAGGAGTGGCTATAGACATTCAATCGTTCCAGGCTCTCAGTGAG  1860
Sbjct  1854  ...................................C........................  1913

Query  1861  AGCGCGACCCTTGTGTACAACGAACGCGAGTTCGTTAACAGGAAGCTGTGGCACATAGCA  1920
Sbjct  1914  ..T..........................................T..............  1973

Query  1921  GTATACGGGGCAGCACTCAATACTGATGAAGAAGGATACGAGAAGGTCCCGGTAGAGAGA  1980
Sbjct  1974  ............................................................  2033

Query  1981  GCAGAATCAGATTATGTGTTTGATGTAGACCAAAAAATGTGCCTaaaaaaaGAGCAGGCA  2040
Sbjct  2034  .................A............A.............C...............  2093

Query  2041  TCAGGTTGGGTACTCTGTGGCGAACTAGTCAACCCCCCATTCCACGAATTCGCATATGAA  2100
Sbjct  2094  .....A..................T...................................  2153

Query  2101  GGGCTCCGCACGAGACCGTCAGCACCCTACAAGGTTCATACAGTAGGTGTGTACGGAGTG  2160
Sbjct  2154  ........T..T......G.........................................  2213

Query  2161  CCAGGATCAGGCAAATCCGCAATAATCAAGAACACGGTCACCATGTCTGACCTAGTATTG  2220
Sbjct  2214  ............................................................  2273

Query  2221  AGTGGTAAGAAAGAGAACTGCTTAGAAATTATGAACGATGTACTTAAACACAGAGCTCTA  2280
Sbjct  2274  ............................................................  2333

Query  2281  CGTATCACAGCGAAGACCGTAGACTCAGTGTTATTAAACGGCGTGAAACACACGCCTAAC  2340
Sbjct  2334  .....T.............................G........................  2393

Query  2341  ATACTATACATCGACGAAGCGTTCTCATGCCATGCAGGGACTCTGTTGGCCACTATAGCC  2400
Sbjct  2394  ............................................................  2453

Query  2401  ATAGTCAGGCCCAAACAGAAAGTGGTACTGTGCGGAGACCCGAAACAATGCGGATTCTTC  2460
Sbjct  2454  T...........................................................  2513

Query  2461  AATATGATGCAACTGAAAGTTAATTACAATCATGACATCTGCTCAGAAGTCTTCCACAAA  2520
Sbjct  2514  .......................C....................................  2573

Query  2521  AGTATCTCTAGACGGTGCACCCAGGATATCACGGCCATCGTTTCCAAATTACATTACCAG  2580
Sbjct  2574  ............................................T...............  2633

Query  2581  GACCGAATGAGGACCACAAACCCCCGAAAAGGAGACATCATTATAGACACTACCGGCACT  2640
Sbjct  2634  ..................................................C.........  2693

Query  2641  ACCAAACCAGCCAAAACAGATCTGATTCTGACGTGCTTCAGGGGATGGGTGAAACAGTTG  2700
Sbjct  2694  ....................C.......................................  2753
```

```
Query  2701  CAGCAAGACTACAGAGGTAACGAAGTAATGACGGCTGCAGCGTCCCAAGGACTGACGAGG  2760
Sbjct  2754  ............................................................  2813

Query  2761  GCCTCCGTATATGCGGTTCGAACTAAAGTCAATGAGAACCCGCTATATGCACAGACCTCC  2820
Sbjct  2814  ............................................................  2873

Query  2821  GAGCACGTGAACGTGTTGTTAACACGCACAGAAAACAAGCTAGTATGGAAGACCTTGTCA  2880
Sbjct  2874  .......................................T....................  2933

Query  2881  ACAGATCCCTGGATTAAAACACTGACTAACCCACCTAGAGGGCACTATACCGCCACCATA  2940
Sbjct  2934  ............................................................  2993

Query  2941  GCAGAATGGGAAGCGGAACACCAGGGTATAATGAAGGCCATACAAGGGTATGCACCGCCC  3000
Sbjct  2994  ..............A.............................................  3053

Query  3001  GTGAACACCTTCATGAACAAAGTAAATGTGTGCTGGGCAAAGACACTTACGCCTGTGCTG  3060
Sbjct  3054  ...........T................................G..C..A.........  3113

Query  3061  GAAACTGCGGGTATCTCCCTGTCAGCAGAAGACTGGTCTGAACTGCTGCCCCCGTTTGCC  3120
Sbjct  3114  ..................T.........................................  3173

Query  3121  CAGGACGTGGCGTACTCACCCGAGGTGGCATTAAACATCATATGCACGAAAATGTATGGG  3180
Sbjct  3174  .......C.......................................A............  3233

Query  3181  TTTGACTTAGACACTGGTCTTTTTTCCAGGCCATCAGTGCCAATGACATACACCAAAGAC  3240
Sbjct  3234  ........G.....C.............A.........A.....................  3293

Query  3241  CATTGGGATAACAGAGTTGGAGGGAAAATGTATGGATTCAGCCAACAAGCATACGATCAG  3300
Sbjct  3294  ............................................................  3353

Query  3301  CTGGCAAGACGACATCCGTACCTTCGAGGTAGAGAGAAATCAGGAATGCAGATCGTAGTC  3360
Sbjct  3354  ...................................A........................  3413

Query  3361  ACTGAAATGCGTATCCAGCGCCCAAGATCGGATGCCAACATCATCCCGATCAACCGCAGG  3420
Sbjct  3414  .....G......................................................  3473

Query  3421  CTCCCTCACTCACTCGTAGCCACACACGAGTATAGGCGAGCTGCACGGGCCGAGGAATTC  3480
Sbjct  3474  ............................................G...............  3533

Query  3481  TTCACCACGACACGAGGGTACACTATGCTGCTGGTCTCTGAGTATAACATGAACTTACCA  3540
Sbjct  3534  ..........T.................................................  3593

Query  3541  AACAAGAAGATCACCTGGCTGGCTCCGATAGGGACGCAGGGGGCCCATCACACCGCCAAC  3600
Sbjct  3594  ............................................................  3653

Query  3601  CTAAACTTGGGGATACCACCTCTGCTGGGCAGTTTTGATGCGGTGGTTGTGAACATGCCG  3660
Sbjct  3654  ........................................T...................  3713

Query  3661  ACTCCATTCCGGAACCATCACTACCAGCAATGTGAAGACCACGCGATGAAACTCCAGATG  3720
Sbjct  3714  ................................C...........................  3773

Query  3721  CTGGCAGGCGACGCACTGAGGCACATTAAACCTGGCGGATCATTGTGGGTCAAGGCATAC  3780
Sbjct  3774  .............................G..............................  3833
```

```
Query  3781  GGCTACGCAGACCGGCACAGCGAGCACGTGGTCTTGGCATTGGCTAGAAAGTTTAAAAGC  3840
Sbjct  3834  .......................................C....................  3893

Query  3841  TTCAGAGTCACACAACCCTCATGCGTGACTTCCAACACCGAGGTGTTTCTCCACTTCTCA  3900
Sbjct  3894  ..T........G........G.....A................................G  3953

Query  3901  ATTTTTGACAATGGCAAACGCGCGATAGCCCTGCATTCAGCTAATAGGAAGGCTAACAGT  3960
Sbjct  3954  ............................................................  4013

Query  3961  ATCTTCCAAAACACCTTCTTACCGGCGGGCAGTGCACCGGCGTACAGAGTCAAACGTGGA  4020
Sbjct  4014  ..........................A....A............................  4073

Query  4021  GACATTTCGAACGCCCCAGAGGATGCAGTGGTCAATGCAGCAAACCAACAGGGAGTGAAG  4080
Sbjct  4074  ............................................................  4133

Query  4081  GGTGCTGGAGTTTGCGGTGCAATTTACCGTAAGTGGCCGGACGCTTTCGGTGATGTCGCT  4140
Sbjct  4134  ............................................................  4193

Query  4141  ACTCCAACCGGAACAGCAGTTTCGAAATCCGTCCAAGATAAATTGGTGATCCACGCTGTC  4200
Sbjct  4194  ............................................................  4253

Query  4201  GGCCCGAATTTCTCAAAATGTTCAGAAGAGGAAGGGGACAGAGACCTAGCATCTGCTTAC  4260
Sbjct  4254  ...........T................................................  4313

Query  4261  AGAGCTGCAGCAGAAATAGTGATGGATaaaaaaaTTACAACAGTGGCCGTCCCCTTACTC  4320
Sbjct  4314  ..........................A...........................C.....  4373

Query  4321  TCCACCGGCATTTATGCCGGAGGAAAAAACAGAGTAGAACAGTCACTCAACCATCTCTTC  4380
Sbjct  4374  ..........C...C........G....................................  4433

Query  4381  ACGGCATTCGACAATACTGATGCAGATGTGACCATATATTGCATGGACAAAACATGGGAA  4440
Sbjct  4434  ........T...................................................  4493

Query  4441  AAGAAGATTAAGGAGGCAATCGATCACCGGACTTCGGTTGAGATGGTGCAGGATGACGTG  4500
Sbjct  4494  ............................................................  4553

Query  4501  CAGTTGGAGGAGGAACTGGTACGAGTACACCCTTTGAGTAGTTTAGCAGGTAGGAAGGGT  4560
Sbjct  4554  ......................................C.................A...  4613

Query  4561  TACAGTACGGACAGCGGCCGAGTGTTTTCCTACCTGGAAGGTACCAAATTCCATCAGACT  4620
Sbjct  4614  ............................................................  4673

Query  4621  GCGGTGGACATAGCCGAAATGCAAGTGCTGTGGCCCGCCCTCAAAGAGTCTAATGAGCAA  4680
Sbjct  4674  ........T.............................T.....................  4733

Query  4681  ATAGTGGCATACACCTTAGGAGAATCAATGGACCAGATACGTGGCAAGTGCCCGACAGAA  4740
Sbjct  4734  ............................................................  4793

Query  4741  GATACTGACGCCTCCACACCTCCACGGACTGTGCCGTGCCTCTGTCGATACGCCATGACA  4800
Sbjct  4794  ............................................................  4853

Query  4801  CCAGAGAGAGTGTACCGACTTAAATGCACGAACACTACCCAATTTACGGTTTGCTCATCT  4860
Sbjct  4854  ............................................C.....C.........  4913
```

```
Query  4861  TTTGAGTTGCCAAAGTATCACATTCAGGGAGTGCAGAGAGTAAAATGTGAAAGAATCATC  4920
Sbjct  4914  ............................................................  4973

Query  4921  ATCTTAGATCCCACTGTTCCACCAACTTACAAACGGCCATGCATCAGACGGTACCCCTCC  4980
Sbjct  4974  ............................................................  5033

Query  4981  ACAATCTCTTGTAACTCCTCTGAGGACTCCAGGAGCTTGTCTACTTTTTCTGTCAGCTCC  5040
Sbjct  5034  ........C......................................C............  5093

Query  5041  GACTCCTCGATTGGTTCTCTGCCGGTCGGAGACACGAGACCCATTCCAGCCCCGAGGACC  5100
Sbjct  5094  .........G.......CG..A...................T...........A......  5153

Query  5101  ATTTTCAGACCCGTCCCTGCCCCGAGAGCACCCGTGCTCAGAACCACACCGCCTCCTAAA  5160
Sbjct  5154  G...............................T..........T............C...  5213

Query  5161  CCACCGCGCACATTCACCGTGCGTGCAGAAGTGCACCAAGCACCCCCTACACCTGTACCT  5220
Sbjct  5214  ...................................T........................  5273

Query  5221  CCACCCAGACCGAAGAGGGCTGCAAAGTTGGCTCGTGAGATGCACCCCGGGTTCACCTTC  5280
Sbjct  5274  .....T.............................C........................  5333

Query  5281  GGGGACTTCGGAGAGCACGAGGTTGAGGAGCTTACGGCCTCTCCCTTAACCTTCGGAGAT  5340
Sbjct  5334  ..A..................A.................G....................  5393

Query  5341  TTTGCTGAAGGAGAGATCCAGGGGATGGGAGTGGAGTTTGAATGACTAGGCAGAGCCGGC  5400
Sbjct  5394  ....................A....C..................................  5453

Query  5401  GGGTACATTTTTTCGTCAGACACGGGTCCAGGCCACCTACAGCAGAGATCCGTTTTACAA  5460
Sbjct  5454  .........................................A.....G............  5513

Query  5461  AATTGCACGGCAGAATGTATCTACGAACCGGCAAAACTAGAAAAAATTCATGCACCAAAG  5520
Sbjct  5514  ............................................................  5573

Query  5521  TTGGATAAAACCAAGGAAGATATCTTAAGGAGCAAGTACCAAATGAAACCGTCTGAAGCA  5580
Sbjct  5574  ............................................................  5633

Query  5581  AACAAAAGCAGGTACCAATCTAGAAAAGTAGAAAATATGAAAGCAGAGATCGTAGGTAGA  5640
Sbjct  5634  ........T.........................................T.........  5693

Query  5641  CTCTTGGACGGACTGGGGGAGTATCTGGGCACCGAGCATCCAGTTGAATGCTACCGAATA  5700
Sbjct  5694  ..............A.............................................  5753

Query  5701  ACGTACCCGGTGCCTATATACTCAACTAGTGACCTCAGAGGTCTGTCTAGTGCCAAAACA  5760
Sbjct  5754  .....T....C...................AT..G.........................  5813

Query  5761  GCTGTTAGAGCTTGCAATGCATTTTTGGAAGCTAATTTTCCATCAGTCACTTCATATAAA  5820
Sbjct  5814  ............................................................  5873

Query  5821  ATTACTGATGAATACGACGCATACCTAGATATGGTAGATGGATCAGAGAGCTGTCTGGAC  5880
Sbjct  5874  ............................................................  5933

Query  5881  AGATCCTCCTTTTCGCCGTCTAGATTGCGTAGCTTTCCAAAAACACACTCATACTTGGAC  5940
Sbjct  5934  .................A...........C..............................  5993
```

```
Query  5941  CCACAGATCAACAGTGCGGTACCGTCACCATTCCAAAACACCTTACAAAATGTATTGGCA  6000
Sbjct  5994  ..G.........................................................  6053

Query  6001  GCGGCCACCAAAAGAAACTGTAATGTCACACAGATGAGAGAACTACCAACATATGATTCT  6060
Sbjct  6054  ...............................................G............  6113

Query  6061  GCAGTGCTAAATGTAGAGGCCTTCAGGAAATATGCGTGCAAGCCAGACGTATGGGATGAG  6120
Sbjct  6114  ............................................................  6173

Query  6121  TACAGGGATAATCCGATTTGCATAACCACCGAAAATGTCACCACTTACGTCGCCAAGTTG  6180
Sbjct  6174  ..............A...................................T..T......  6233

Query  6181  AAAGGACCGAAAGCTGCGGCCTTGTTTGCAAAAACACATAACCTGATACCACTACACCAA  6240
Sbjct  6234  ............................................................  6293

Query  6241  GTTCCTATGGACAAATTCACGGTAGATATGAAGAGAGATGTCAAAGTCACGCCCGGAACC  6300
Sbjct  6294  ............................................................  6353

Query  6301  AAGCACACCGAAGAGAGACCAAAGGTACAGGTGATTCAAGCGGCAGAGCCACTAGCCACT  6360
Sbjct  6354  ............................................................  6413

Query  6361  GCCTACCTCTGCGGAATTCACCGTGAATTGGTGCGCCGTCTCAACAACGCGCTTTTCCCA  6420
Sbjct  6414  .....................................................C.....T  6473

Query  6421  AACATCCACACTTTGTTTGATATGTCCGCAGAGGATTTCGATGCAATCATAGCGGAACAT  6480
Sbjct  6474  ..T...................................T.....................  6533

Query  6481  TTTAAGCACGGTGACCATGTGTTGGAAACGGATATAGCCTCTTTTGACAAAAGTCAAGAT  6540
Sbjct  6534  .................C..........................................  6593

Query  6541  GATTCCATGGCACTCACTGCGTTAATGATCCTTGAGGACCTGGGAGTAGACCAAAACCTA  6600
Sbjct  6594  ............................................................  6653

Query  6601  ATGAATTTGATAGAGGCTGCATTCGGGGAAATCGTGAGTACACACTTGCCCACAGGTACT  6660
Sbjct  6654  ............................................................  6713

Query  6661  AGATTCAAATTTGGAGCTATGATGAAGTCTGGAATGTTTTTGACGCTGTTCGTCAATACA  6720
Sbjct  6714  .......G.....................C...............T..............  6773

Query  6721  ATTCTTAATGTGGTTATTGCGTGCCGAGTGTTGGAGGATCAATTGGCGCAGTCGCCGTGG  6780
Sbjct  6774  ........C..................................................C  6833

Query  6781  CCTGCTTTCATAGGAGATGACAACATAATCCATGGTATAATATCAGACAAATTGATGGCA  6840
Sbjct  6834  G...............................C..........................G  6893

Query  6841  GATAGATGTGCCACCTGGATGAACATGGAGGTCAAGATACTGGACTCTATAGTTGGAATA  6900
Sbjct  6894  ............................................T..C............  6953

Query  6901  CGGCCACCTTACTTCTGTGGAGGATTTATTGTATGTGACGATGTAACAGGTACAGCCTGC  6960
Sbjct  6954  ...........T................................................  7013

Query  6961  CGCGTCGCAGACCCACTGAAGAGATTGTTCAAGCTAGGTAAGCCATTGCCACTTGACGAT  7020
Sbjct  7014  ........................C...................................  7073
```

```
Query  7021   GGCCAAGATGAAGACAGAAGACGTGCATTACATGATGAAGTGAAAACCTGGTCGCGCGTA  7080
Sbjct  7074   ............................G...............................  7133

Query  7081   GGGCTGCGACACAGAGTGTGTGAAGCCATCGAAGACCGTTATGCCGTCCACTCATCAGAA  7140
Sbjct  7134   ..................................................T.........  7193

Query  7141   CTAGTTTTATTGGCACTGACTACTCTGTCTAAGAACTTGAAGTCCTTCAGAAACATAAGA  7200
Sbjct  7194   ..............G.............................................  7253

Query  7201   GGGAAACCAATACATCTCTACGGTGGTCCTAAATAG  7236
Sbjct  7254   ....................................  7289
```

```
>Barmah Forest virus isolate SW94457, complete genome
Sequence ID: MN689044.1 Length: 11366
Range 1: 51 to 7259

Score:12491 bits(6764), Expect:0.0,
Identities:7083/7236(98%),  Gaps:27/7236(0%), Strand: Plus/Plus

Query  1      ATGGCGAAACCAGTTGTGAAGATCGACGTGGAACCTGAAAGCCATTTCGCTAAGCAGGTC  60
Sbjct  51     ...............................................T............  110

Query  61     CAGAGTTGCTTCCCGCAGTTTGAGATCGAAGCAGTGCAGACCACACCAAACGATCATGCA  120
Sbjct  111    ........................................G...................  170

Query  121    CACGCGAGGGCGTTTTCGCACCTTGCTACGAAGCTCATAGAAATGGAGACAGCAAAAGAT  180
Sbjct  171    ............................................................  230

Query  181    CAGATCATCCTCGATATCGGAAGTGCACCCGCGAGGAGACTGTATTCAGAACACAAGTAC  240
Sbjct  231    ............................................................  290

Query  241    CACTGTGTTTGCCCAATGAAGTGCACGGAAGATCCAGAGAGAATGCTAGGATATGCACGT  300
Sbjct  291    ............................................................  350

Query  301    AAGTTGATCGCAGGCTCTGCGAAAGGGAAGGCAGAAAAGTTACGCGATCTCAGGGATGTC  360
Sbjct  351    ............................................................  410

Query  361    TTGGCTACGCCAGACATCGAGACGCAGTCGCTATGTCTCCACACAGACGCATCCTGCAGA  420
Sbjct  411    ............................................................  470

Query  421    TACCGCGGTGATGTTGCCGTGTATCAAGACGTGTATGCCATTGACGCACCTACCACGCTG  480
Sbjct  471    ..................................................C.........  530

Query  481    TACCACCAAGCGTTAAAGGGCGTCAGGACCGCATATTGGATAGGCTTTGATACAACGCCG  540
Sbjct  531    ...........................................................A  590

Query  541    TTCATGTACGATGCACTAGCAGGAGCTTACCCGCTCTACTCCACAAACTGGGCTGATGAG  600
Sbjct  591    .........A.......................................C......  650

Query  601    CAAGTGCTCGAGTCCAGAAACATTGGGCTATGTTCAGACAAAGTTTCTGAAGGGGGAAAG  660
```

```
Sbjct  651   ............................................................  710

Query  661   AAAGGGAGATCAATCCTCAGGAAGAAGTTCTTGAAGCAGTCAGACAGAGTCATGTTCTCT  720
Sbjct  711   ............................................................  770

Query  721   GTCGGCTCGACGTTGTATACGGAAAGCCGTAAATTACTGCAAAGTTGGCACCTGCCATCC  780
Sbjct  771   ............................................C...............  830

Query  781   ACATTCCATCTCAAAGGCAAATCTTCGTTCACGTGCCGCTGCGACACTATCGTCAGCTGC  840
Sbjct  831   ................................A..............C............  890

Query  841   GAAGGGTATGTTCTGAAGAAAATTACAATGTGTCCTGGAGTGACAGGCAAACCGATAGGA  900
Sbjct  891   ................................C..C........................  950

Query  901   TATGCCGTCACCCATCACAAAGAAGGATTCGTAGTCGGAAAAGTCACAGATACCATTCGC  960
Sbjct  951   ....................G.............................C.........  1010

Query  961   GGCGAGAGAGTCTCCTTCGCCGTGTGTACTTATGTACCAACAACACTCTGCGACCAGATG  1020
Sbjct  1011  ............................................................  1070

Query  1021  ACCGGGATCCTAGCAACAGAAGTAACAGCCGATGATGCCCAGAAACTGCTGGTGGGTTTG  1080
Sbjct  1071  ........T...................................................  1130

Query  1081  AACCAGAGAATAGTAGTTAATGGTAGGACCCAGAGAAATACCAATACTATGAAGAACTAC  1140
Sbjct  1131  ............................................C...............  1190

Query  1141  CTGCTACCACTGGTTGCACAAGCGCTAGCAAAATGGGCGAAGGAAGCAAAACAGGATATG  1200
Sbjct  1191  ......................................A.....................  1250

Query  1201  GAAGATGAAAGACCCCTGAACGAACGCCAACGAACGCTAACGTGCCTCTGCTGCTGGGCA  1260
Sbjct  1251  ............................................................  1310

Query  1261  TTTAAGCGAAACAAACGCCACGCCATTTACAAGAGACCAGACACACAGAGTATAGTCAAG  1320
Sbjct  1311  ............................................................  1370

Query  1321  GTCCCTTGCGAATTCACAAGCTTTCCTTTGGTCAGCCTGTGGTCCGCTGGGATGTCTATA  1380
Sbjct  1371  ..................G.........................................  1430

Query  1381  TCTCTTAGGCAGAAGTTGAAGATGATGCTGCAGGCGAGGCAGCCCACACAAATAGCAGCA  1440
Sbjct  1431  ............................................................  1490

Query  1441  GTGACTGAGGAACTCATACAAGAAGCAGCTGCAGTAGAGCAAGAGGCCGTGGATACGGCC  1500
Sbjct  1491  ..................................C.........................  1550

Query  1501  AATGCCGAGCTGGACCACGCCGCATGGCCCTCCATTGTGGATACGACAGAGCGCCATGTT  1560
Sbjct  1551  ...............................................G............  1610

Query  1561  GAGGTCGAAGTGGAAGAACTCGACCAGCGTGCAGGGGAAGGGGTAGTGGAAACACCTCGA  1620
Sbjct  1611  ............................................................  1670

Query  1621  AACTCTATCAAAGTTTCAACACAGATCGGGGACGCGTTAATCGGCAGTTACCTGATCCTA  1680
Sbjct  1671  ........T........T..........................................  1730

Query  1681  TCACCCCAAGCAGTCCTACGCAGCGAAAAATTAGCCTGCATACATGATCTTGCAGAGCAG  1740
```

```
Sbjct  1731  ............................................................  1790

Query  1741  GTTAAGTTGGTCACACACTCTGGCCGTAGTGGTAGGTACGCCGTCGACAAATACNACGGA  1800
Sbjct  1791  ..C.................C..T..............................G.....  1850

Query  1801  AGAGTACTAGTCCCTACAGGAGTGGCTATAGACATTCAATCGTTCCAGGCTCTCAGTGAG  1860
Sbjct  1851  ...................................C........................  1910

Query  1861  AGCGCGACCCTTGTGTACAACGAACGCGAGTTCGTTAACAGGAAGCTGTGGCACATAGCA  1920
Sbjct  1911  ..T..........................................T..............  1970

Query  1921  GTATACGGGGCAGCACTCAATACTGATGAAGAAGGATACGAGAAGGTCCCGGTAGAGAGA  1980
Sbjct  1971  ............................................................  2030

Query  1981  GCAGAATCAGATTATGTGTTTGATGTAGACCAAAAAATGTGCCTaaaaaaaGAGCAGGCA  2040
Sbjct  2031  .................A............A.............C...............  2090

Query  2041  TCAGGTTGGGTACTCTGTGGCGAACTAGTCAACCCCCCATTCCACGAATTCGCATATGAA  2100
Sbjct  2091  .....A..............T.......................................  2150

Query  2101  GGGCTCCGCACGAGACCGTCAGCACCCTACAAGGTTCATACAGTAGGTGTGTACGGAGTG  2160
Sbjct  2151  ........T..T................................................  2210

Query  2161  CCAGGATCAGGCAAATCCGCAATAATCAAGAACACGGTCACCATGTCTGACCTAGTATTG  2220
Sbjct  2211  ............................................................  2270

Query  2221  AGTGGTAAGAAAGAGAACTGCTTAGAAATTATGAACGATGTACTTAAACACAGAGCTCTA  2280
Sbjct  2271  ............................................................  2330

Query  2281  CGTATCACAGCGAAGACCGTAGACTCAGTGTTATTAAACGGCGTGAAACACACGCCTAAC  2340
Sbjct  2331  .....T.............................G........................  2390

Query  2341  ATACTATACATCGACGAAGCGTTCTCATGCCATGCAGGGACTCTGTTGGCCACTATAGCC  2400
Sbjct  2391  ............................................................  2450

Query  2401  ATAGTCAGGCCCAAACAGAAAGTGGTACTGTGCGGAGACCCGAAACAATGCGGATTCTTC  2460
Sbjct  2451  T...........................................................  2510

Query  2461  AATATGATGCAACTGAAAGTTAATTACAATCATGACATCTGCTCAGAAGTCTTCCACAAA  2520
Sbjct  2511  .......................C....................................  2570

Query  2521  AGTATCTCTAGACGGTGCACCCAGGATATCACGGCCATCGTTTCCAAATTACATTACCAG  2580
Sbjct  2571  ............................................T...............  2630

Query  2581  GACCGAATGAGGACCACAAACCCCCGAAAAGGAGACATCATTATAGACACTACCGGCACT  2640
Sbjct  2631  ..................................................C.........  2690

Query  2641  ACCAAACCAGCCAAAACAGATCTGATTCTGACGTGCTTCAGGGGATGGGTGAAACAGTTG  2700
Sbjct  2691  ....................C.......................................  2750

Query  2701  CAGCAAGACTACAGAGGTAACGAAGTAATGACGGCTGCAGCGTCCCAAGGACTGACGAGG  2760
Sbjct  2751  ............................................................  2810

Query  2761  GCCTCCGTATATGCGGTTCGAACTAAAGTCAATGAGAACCCGCTATATGCACAGACCTCC  2820
```

```
Sbjct  2811  ............................................................  2870

Query  2821  GAGCACGTGAACGTGTTGTTAACACGCACAGAAAACAAGCTAGTATGGAAGACCTTGTCA  2880
Sbjct  2871  .......................................T....................  2930

Query  2881  ACAGATCCCTGGATTAAAACACTGACTAACCCACCTAGAGGGCACTATACCGCCACCATA  2940
Sbjct  2931  ............................................................  2990

Query  2941  GCAGAATGGGAAGCGGAACACCAGGGTATAATGAAGGCCATACAAGGGTATGCACCGCCC  3000
Sbjct  2991  ..............A.............................................  3050

Query  3001  GTGAACACCTTCATGAACAAAGTAAATGTGTGCTGGGCAAAGACACTTACGCCTGTGCTG  3060
Sbjct  3051  ............................................G..C..A.........  3110

Query  3061  GAAACTGCGGGTATCTCCCTGTCAGCAGAAGACTGGTCTGAACTGCTGCCCCCGTTTGCC  3120
Sbjct  3111  ............................................................  3170

Query  3121  CAGGACGTGGCGTACTCACCCGAGGTGGCATTAAACATCATATGCACGAAAATGTATGGG  3180
Sbjct  3171  ...............................................A............  3230

Query  3181  TTTGACTTAGACACTGGTCTTTTTTCCAGGCCATCAGTGCCAATGACATACACCAAAGAC  3240
Sbjct  3231  ........G.....C.............A......T..A.....................  3290

Query  3241  CATTGGGATAACAGAGTTGGAGGGAAAATGTATGGATTCAGCCAACAAGCATACGATCAG  3300
Sbjct  3291  ............................................................  3350

Query  3301  CTGGCAAGACGACATCCGTACCTTCGAGGTAGAGAGAAATCAGGAATGCAGATCGTAGTC  3360
Sbjct  3351  ...................................A........................  3410

Query  3361  ACTGAAATGCGTATCCAGCGCCCAAGATCGGATGCCAACATCATCCCGATCAACCGCAGG  3420
Sbjct  3411  .....G......................................................  3470

Query  3421  CTCCCTCACTCACTCGTAGCCACACACGAGTATAGGCGAGCTGCACGGGCCGAGGAATTC  3480
Sbjct  3471  ........T...................................G...............  3530

Query  3481  TTCACCACGACACGAGGGTACACTATGCTGCTGGTCTCTGAGTATAACATGAACTTACCA  3540
Sbjct  3531  ..........T.................................................  3590

Query  3541  AACAAGAAGATCACCTGGCTGGCTCCGATAGGGACGCAGGGGGCCCATCACACCGCCAAC  3600
Sbjct  3591  ............................................................  3650

Query  3601  CTAAACTTGGGGATACCACCTCTGCTGGGCAGTTTTGATGCGGTGGTTGTGAACATGCCG  3660
Sbjct  3651  ........................................T...................  3710

Query  3661  ACTCCATTCCGGAACCATCACTACCAGCAATGTGAAGACCACGCGATGAAACTCCAGATG  3720
Sbjct  3711  ................................C...........................  3770

Query  3721  CTGGCAGGCGACGCACTGAGGCACATTAAACCTGGCGGATCATTGTGGGTCAAGGCATAC  3780
Sbjct  3771  .............................G..............................  3830

Query  3781  GGCTACGCAGACCGGCACAGCGAGCACGTGGTCTTGGCATTGGCTAGAAAGTTTAAAAGC  3840
Sbjct  3831  .......................................C....................  3890

Query  3841  TTCAGAGTCACACAACCCTCATGCGTGACTTCCAACACCGAGGTGTTTCTCCACTTCTCA  3900
```

```
Sbjct  3891  ...........G........G.....A.................................  3950

Query  3901  ATTTTTGACAATGGCAAACGCGCGATAGCCCTGCATTCAGCTAATAGGAAGGCTAACAGT  3960
Sbjct  3951  ............................................................  4010

Query  3961  ATCTTCCAAAACACCTTCTTACCGGCGGGCAGTGCACCGGCGTACAGAGTCAAACGTGGA  4020
Sbjct  4011  ..........................A....A............................  4070

Query  4021  GACATTTCGAACGCCCCAGAGGATGCAGTGGTCAATGCAGCAAACCAACAGGGAGTGAAG  4080
Sbjct  4071  ............................................................  4130

Query  4081  GGTGCTGGAGTTTGCGGTGCAATTTACCGTAAGTGGCCGGACGCTTTCGGTGATGTCGCT  4140
Sbjct  4131  ............................................................  4190

Query  4141  ACTCCAACCGGAACAGCAGTTTCGAAATCCGTCCAAGATAAATTGGTGATCCACGCTGTC  4200
Sbjct  4191  ............................................................  4250

Query  4201  GGCCCGAATTTCTCAAAATGTTCAGAAGAGGAAGGGGACAGAGACCTAGCATCTGCTTAC  4260
Sbjct  4251  ............................................................  4310

Query  4261  AGAGCTGCAGCAGAAATAGTGATGGATaaaaaaaTTACAACAGTGGCCGTCCCCTTACTC  4320
Sbjct  4311  ..........................A...........................C.....  4370

Query  4321  TCCACCGGCATTTATGCCGGAGGAAAAAACAGAGTAGAACAGTCACTCAACCATCTCTTC  4380
Sbjct  4371  ..............C........G....................................  4430

Query  4381  ACGGCATTCGACAATACTGATGCAGATGTGACCATATATTGCATGGACAAAACATGGGAA  4440
Sbjct  4431  ........T...................................................  4490

Query  4441  AAGAAGATTAAGGAGGCAATCGATCACCGGACTTCGGTTGAGATGGTGCAGGATGACGTG  4500
Sbjct  4491  ............................................................  4550

Query  4501  CAGTTGGAGGAGGAACTGGTACGAGTACACCCTTTGAGTAGTTTAGCAGGTAGGAAGGGT  4560
Sbjct  4551  ......................................C.....................  4610

Query  4561  TACAGTACGGACAGCGGCCGAGTGTTTTCCTACCTGGAAGGTACCAAATTCCATCAGACT  4620
Sbjct  4611  ............................................................  4670

Query  4621  GCGGTGGACATAGCCGAAATGCAAGTGCTGTGGCCCGCCCTCAAAGAGTCTAATGAGCAA  4680
Sbjct  4671  ........T.............................T.....................  4730

Query  4681  ATAGTGGCATACACCTTAGGAGAATCAATGGACCAGATACGTGGCAAGTGCCCGACAGAA  4740
Sbjct  4731  ............................................................  4790

Query  4741  GATACTGACGCCTCCACACCTCCACGGACTGTGCCGTGCCTCTGTCGATACGCCATGACA  4800
Sbjct  4791  ............................................................  4850

Query  4801  CCAGAGAGAGTGTACCGACTTAAATGCACGAACACTACCCAATTTACGGTTTGCTCATCT  4860
Sbjct  4851  ............................................C.....C.........  4910

Query  4861  TTTGAGTTGCCAAAGTATCACATTCAGGGAGTGCAGAGAGTAAAATGTGAAAGAATCATC  4920
Sbjct  4911  ............................................................  4970

Query  4921  ATCTTAGATCCCACTGTTCCACCAACTTACAAACGGCCATGCATCAGACGGTACCCCTCC  4980
```

```
Sbjct  4971  ............................................................  5030

Query  4981  ACAATCTCTTGTAACTCCTCTGAGGACTCCAGGAGCTTGTCTACTTTTTCTGTCAGCTCC  5040
Sbjct  5031  ........C...................................--.--..T.-..A  5085

Query  5041  GACTCCTCGATTGGTTCTCTGCCGGTCGGAGACACGAGACCCATTCCAGCCCCGAGGACC  5100
Sbjct  5086  .-..-.-..--------.-..--.--------......T..........A......  5123

Query  5101  ATTTTCAGACCCGTCCCTGCCCCGAGAGCACCCGTGCTCAGAACCACACCGCCTCCTAAA  5160
Sbjct  5124  G.............................T.........T...............  5183

Query  5161  CCACCGCGCACATTCACCGTGCGTGCAGAAGTGCACCAAGCACCCCCTACACCTGTACCT  5220
Sbjct  5184  .................................T........................  5243

Query  5221  CCACCCAGACCGAAGAGGGCTGCAAAGTTGGCTCGTGAGATGCACCCCGGGTTCACCTTC  5280
Sbjct  5244  .....T......................................................  5303

Query  5281  GGGGACTTCGGAGAGCACGAGGTTGAGGAGCTTACGGCCTCTCCCTTAACCTTCGGAGAT  5340
Sbjct  5304  ..A.................A................G....................  5363

Query  5341  TTTGCTGAAGGAGAGATCCAGGGGATGGGAGTGGAGTTTGAATGACTAGGCAGAGCCGGC  5400
Sbjct  5364  ....................A.......................................  5423

Query  5401  GGGTACATTTTTTCGTCAGACACGGGTCCAGGCCACCTACAGCAGAGATCCGTTTTACAA  5460
Sbjct  5424  ......................................A.....G...........  5483

Query  5461  AATTGCACGGCAGAATGTATCTACGAACCGGCAAAACTAGAAAAAATTCATGCACCAAAG  5520
Sbjct  5484  ............................................................  5543

Query  5521  TTGGATAAAACCAAGGAAGATATCTTAAGGAGCAAGTACCAAATGAAACCGTCTGAAGCA  5580
Sbjct  5544  ............................................................  5603

Query  5581  AACAAAAGCAGGTACCAATCTAGAAAAGTAGAAAATATGAAAGCAGAGATCGTAGGTAGA  5640
Sbjct  5604  ........T....................................T.........  5663

Query  5641  CTCTTGGACGGACTGGGGGAGTATCTGGGCACCGAGCATCCAGTTGAATGCTACCGAATA  5700
Sbjct  5664  ..............A.............................................  5723

Query  5701  ACGTACCCGGTGCCTATATACTCAACTAGTGACCTCAGAGGTCTGTCTAGTGCCAAAACA  5760
Sbjct  5724  .....T....C..................AT..G........................  5783

Query  5761  GCTGTTAGAGCTTGCAATGCATTTTTGGAAGCTAATTTTCCATCAGTCACTTCATATAAA  5820
Sbjct  5784  ............................................................  5843

Query  5821  ATTACTGATGAATACGACGCATACCTAGATATGGTAGATGGATCAGAGAGCTGTCTGGAC  5880
Sbjct  5844  ............................................................  5903

Query  5881  AGATCCTCCTTTTCGCCGTCTAGATTGCGTAGCTTTCCAAAAACACACTCATACTTGGAC  5940
Sbjct  5904  .................A..........C...............................  5963

Query  5941  CCACAGATCAACAGTGCGGTACCGTCACCATTCCAAAACACCTTACAAAATGTATTGGCA  6000
Sbjct  5964  ..G.........................................................  6023

Query  6001  GCGGCCACCAAAAGAAACTGTAATGTCACACAGATGAGAGAACTACCAACATATGATTCT  6060
```

```
Sbjct  6024  ...............................................G............  6083

Query  6061  GCAGTGCTAAATGTAGAGGCCTTCAGGAAATATGCGTGCAAGCCAGACGTATGGGATGAG  6120
Sbjct  6084  ............................................................  6143

Query  6121  TACAGGGATAATCCGATTTGCATAACCACCGAAAATGTCACCACTTACGTCGCCAAGTTG  6180
Sbjct  6144  ..............A...................................T..T......  6203

Query  6181  AAAGGACCGAAAGCTGCGGCCTTGTTTGCAAAAACACATAACCTGATACCACTACACCAA  6240
Sbjct  6204  ............................................................  6263

Query  6241  GTTCCTATGGACAAATTCACGGTAGATATGAAGAGAGATGTCAAAGTCACGCCCGGAACC  6300
Sbjct  6264  .....................................................G......  6323

Query  6301  AAGCACACCGAAGAGAGACCAAAGGTACAGGTGATTCAAGCGGCAGAGCCACTAGCCACT  6360
Sbjct  6324  ............................................................  6383

Query  6361  GCCTACCTCTGCGGAATTCACCGTGAATTGGTGCGCCGTCTCAACAACGCGCTTTTCCCA  6420
Sbjct  6384  .....................................................C......  6443

Query  6421  AACATCCACACTTTGTTTGATATGTCCGCAGAGGATTTCGATGCAATCATAGCGGAACAT  6480
Sbjct  6444  ..T...................................T.....................  6503

Query  6481  TTTAAGCACGGTGACCATGTGTTGGAAACGGATATAGCCTCTTTTGACAAAAGTCAAGAT  6540
Sbjct  6504  .................C..........................................  6563

Query  6541  GATTCCATGGCACTCACTGCGTTAATGATCCTTGAGGACCTGGGAGTAGACCAAAACCTA  6600
Sbjct  6564  ............................................................  6623

Query  6601  ATGAATTTGATAGAGGCTGCATTCGGGGAAATCGTGAGTACACACTTGCCCACAGGTACT  6660
Sbjct  6624  ............................................................  6683

Query  6661  AGATTCAAATTTGGAGCTATGATGAAGTCTGGAATGTTTTTGACGCTGTTCGTCAATACA  6720
Sbjct  6684  .......G.....................C...............T..............  6743

Query  6721  ATTCTTAATGTGGTTATTGCGTGCCGAGTGTTGGAGGATCAATTGGCGCAGTCGCCGTGG  6780
Sbjct  6744  ........C..................................................C  6803

Query  6781  CCTGCTTTCATAGGAGATGACAACATAATCCATGGTATAATATCAGACAAATTGATGGCA  6840
Sbjct  6804  G..........................................................G  6863

Query  6841  GATAGATGTGCCACCTGGATGAACATGGAGGTCAAGATACTGGACTCTATAGTTGGAATA  6900
Sbjct  6864  ............................................T..C............  6923

Query  6901  CGGCCACCTTACTTCTGTGGAGGATTTATTGTATGTGACGATGTAACAGGTACAGCCTGC  6960
Sbjct  6924  ...........T................................................  6983

Query  6961  CGCGTCGCAGACCCACTGAAGAGATTGTTCAAGCTAGGTAAGCCATTGCCACTTGACGAT  7020
Sbjct  6984  ........................C...................................  7043

Query  7021  GGCCAAGATGAAGACAGAAGACGTGCATTACATGATGAAGTGAAAACCTGGTCGCGCGTA  7080
Sbjct  7044  .............................G..............................  7103

Query  7081  GGGCTGCGACACAGAGTGTGTGAAGCCATCGAAGACCGTTATGCCGTCCACTCATCAGAA  7140
```

```
Sbjct  7104  ..................................................T.........  7163

Query  7141  CTAGTTTTATTGGCACTGACTACTCTGTCTAAGAACTTGAAGTCCTTCAGAAACATAAGA  7200
Sbjct  7164  ............................................................  7223

Query  7201  GGGAAACCAATACATCTCTACGGTGGTCCTAAATAG  7236
Sbjct  7224  ....................................  7259
```

>Barmah Forest virus isolate PNG, complete genome
Sequence ID: MN115377.1 Length: 11480
Range 1: 63 to 7298

Score:12152 bits(6580), Expect:0.0,
Identities:7019/7237(97%),  Gaps:2/7237(0%), Strand: Plus/Plus

```
Query  1     ATGGCGAAACCAGTTGTGAAGATCGACGTGGAACCTGAAAGCCATTTCGCTAAGCAGGTC  60
Sbjct  63    .......................T....................................  122

Query  61    CAGAGTTGCTTCCCGCAGTTTGAGATCGAAGCAGTGCAGACCACACCAAACGATCATGCA  120
Sbjct  123   .........................................G.....T............  182

Query  121   CACGCGAGGGCGTTTTCGCACCTTGCTACGAAGCTCATAGAAATGGAGACAGCAAAAGAT  180
Sbjct  183   ...........................................................C  242

Query  181   CAGATCATCCTCGATATCGGAAGTGCACCCGCGAGGAGACTGTATTCAGAACACAAGTAC  240
Sbjct  243   ............................................................  302

Query  241   CACTGTGTTTGCCCAATGAAGTGCACGGAAGATCCAGAGAGAATGCTAGGATATGCACGT  300
Sbjct  303   ........C..........................G........................  362

Query  301   AAGTTGATCGCAGGCTCTGCGAAAGGGAAGGCAGAAAAGTTACGCGATCTCAGGGATGTC  360
Sbjct  363   ............................................................  422

Query  361   TTGGCTACGCCAGACATCGAGACGCAGTCGCTATGTCTCCACACAGACGCATCCTGCAGA  420
Sbjct  423   .............................A..............................  482

Query  421   TACCGCGGTGATGTTGCCGTGTATCAAGACGTGTATGCCATTGACGCACCTACCACGCTG  480
Sbjct  483   ............................................................  542

Query  481   TACCACCAAGCGTTAAAGGGCGTCAGGACCGCATATTGGATAGGCTTTGATACAACGCCG  540
Sbjct  543   .............................T.......................T.....A  602

Query  541   TTCATGTACGATGCACTAGCAGGAGCTTACCCGCTCTACTCCACAAACTGGGCTGATGAG  600
Sbjct  603   .........A..................................................  662

Query  601   CAAGTGCTCGAGTCCAGAAACATTGGGCTATGTTCAGACAAAGTTTCTGAAGGGGGAAAG  660
Sbjct  663   ............................................................  722

Query  661   AAAGGGAGATCAATCCTCAGGAAGAAGTTCTTGAAGCAGTCAGACAGAGTCATGTTCTCT  720
Sbjct  723   ............................................................  782
```

```
Query  721   GTCGGCTCGACGTTGTATACGGAAAGCCGTAAATTACTGCAAAGTTGGCACCTGCCATCC  780
Sbjct  783   .......................G........G.......................G..T  842

Query  781   ACATTCCATCTCAAAGGCAAATCTTCGTTCACGTGCCGCTGCGACACTATCGTCAGCTGC  840
Sbjct  843   .......................A........A..............C............  902

Query  841   GAAGGGTATGTTCTGAAGAAAATTACAATGTGTCCTGGAGTGACAGGCAAACCGATAGGA  900
Sbjct  903   ..............A........C..T.................................  962

Query  901   TATGCCGTCACCCATCACAAAGAAGGATTCGTAGTCGGAAAAGTCACAGATACCATTCGC  960
Sbjct  963   ..................................................C.........  1022

Query  961   GGCGAGAGAGTCTCCTTCGCCGTGTGTACTTATGTACCAACAACACTCTGCGACCAGATG  1020
Sbjct  1023  ................................C.................T.........  1082

Query  1021  ACCGGGATCCTAGCAACAGAAGTAACAGCCGATGATGCCCAGAAACTGCTGGTGGGTTTG  1080
Sbjct  1083  .....................................................T..C...  1142

Query  1081  AACCAGAGAATAGTAGTTAATGGTAGGACCCAGAGAAATACCAATACTATGAAGAACTAC  1140
Sbjct  1143  .............................T..............C...............  1202

Query  1141  CTGCTACCACTGGTTGCACAAGCGCTAGCAAAATGGGCGAAGGAAGCAAAACAGGATATG  1200
Sbjct  1203  ...........A...........A..............A.....................  1262

Query  1201  GAAGATGAAAGACCCCTGAACGAACGCCAACGAACGCTAACGTGCCTCTGCTGCTGGGCA  1260
Sbjct  1263  ...............................................T..T.........  1322

Query  1261  TTTAAGCGAAACAAACGCCACGCCATTTACAAGAGACCAGACACACAGAGTATAGTCAAG  1320
Sbjct  1323  ..................................................C.........  1382

Query  1321  GTCCCTTGCGAATTCACAAGCTTTCCTTTGGTCAGCCTGTGGTCCGCTGGGATGTCTATA  1380
Sbjct  1383  .....G..................................................C...  1442

Query  1381  TCTCTTAGGCAGAAGTTGAAGATGATGCTGCAGGCGAGGCAGCCCACACAAATAGCAGCA  1440
Sbjct  1443  C............................A....AA.....A..................  1502

Query  1441  GTGACTGAGGAACTCATACAAGAAGCAGCTGCAGTAGAGCAAGAGGCCGTGGATACGGCC  1500
Sbjct  1503  ..................................C.........................  1562

Query  1501  AATGCCGAGCTGGACCACGCCGCATGGCCCTCCATTGTGGATACGACAGAGCGCCATGTT  1560
Sbjct  1563  ...............................................G............  1622

Query  1561  GAGGTCGAAGTGGAAGAACTCGACCAGCGTGCAGGGGAAGGGGTAGTGGAAACACCTCGA  1620
Sbjct  1623  ..T.........................................................  1682

Query  1621  AACTCTATCAAAGTTTCAACACAGATCGGGGACGCGTTAATCGGCAGTTACCTGATCCTA  1680
Sbjct  1683  ............................................................  1742

Query  1681  TCACCCCAAGCAGTCCTACGCAGCGAAAAATTAGCCTGCATACATGATCTTGCAGAGCAG  1740
Sbjct  1743  ..............................C.......T........C............  1802

Query  1741  GTTAAGTTGGTCACACACTCTGGCCGTAGTGGTAGGTACGCCGTCGACAAATACNACGGA  1800
Sbjct  1803  ....................C........C..............................  1862
```

```
Query  1801  AGAGTACTAGTCCCTACAGGAGTGGCTATAGACATTCAATCGTTCCAGGCTCTCAGTGAG  1860
Sbjct  1863  ............................................................  1922

Query  1861  AGCGCGACCCTTGTGTACAACGAACGCGAGTTCGTTAACAGGAAGCTGTGGCACATAGCA  1920
Sbjct  1923  .....A......................................................  1982

Query  1921  GTATACGGGGCAGCACTCAATACTGATGAAGAAGGATACGAGAAGGTCCCGGTAGAGAGA  1980
Sbjct  1983  ....................C.............................A.........  2042

Query  1981  GCAGAATCAGATTATGTGTTTGATGTAGACCAAAAAATGTGCCTaaaaaaaGAGCAGGCA  2040
Sbjct  2043  .................A.............................G............  2102

Query  2041  TCAGGTTGGGTACTCTGTGGCGAACTAGTCAACCCCCCATTCCACGAATTCGCATATGAA  2100
Sbjct  2103  ............................................T..G.....G......  2162

Query  2101  GGGCTCCGCACGAGACCGTCAGCACCCTACAAGGTTCATACAGTAGGTGTGTACGGAGTG  2160
Sbjct  2163  .................A..........................................  2222

Query  2161  CCAGGATCAGGCAAATCCGCAATAATCAAGAACACGGTCACCATGTCTGACCTAGTATTG  2220
Sbjct  2223  ............................................................  2282

Query  2221  AGTGGTAAGAAAGAGAACTGCTTAGAAATTATGAACGATGTACTTAAACACAGAGCTCTA  2280
Sbjct  2283  .............................C..............................  2342

Query  2281  CGTATCACAGCGAAGACCGTAGACTCAGTGTTATTAAACGGCGTGAAACACACGCCTAAC  2340
Sbjct  2343  .....T.............................G........................  2402

Query  2341  ATACTATACATCGACGAAGCGTTCTCATGCCATGCAGGGACTCTGTTGGCCACTATAGCC  2400
Sbjct  2403  .....................................................C......  2462

Query  2401  ATAGTCAGGCCCAAACAGAAAGTGGTACTGTGCGGAGACCCGAAACAATGCGGATTCTTC  2460
Sbjct  2463  .............................A.............................T  2522

Query  2461  AATATGATGCAACTGAAAGTTAATTACAATCATGACATCTGCTCAGAAGTCTTCCACAAA  2520
Sbjct  2523  .................G..C..C.....C..............................  2582

Query  2521  AGTATCTCTAGACGGTGCACCCAGGATATCACGGCCATCGTTTCCAAATTACATTACCAG  2580
Sbjct  2583  ............................................................  2642

Query  2581  GACCGAATGAGGACCACAAACCCCCGAAAAGGAGACATCATTATAGACACTACCGGCACT  2640
Sbjct  2643  .....G.............................T..............C.........  2702

Query  2641  ACCAAACCAGCCAAAACAGATCTGATTCTGACGTGCTTCAGGGGATGGGTGAAACAGTTG  2700
Sbjct  2703  ....................C.......................................  2762

Query  2701  CAGCAAGACTACAGAGGTAACGAAGTAATGACGGCTGCAGCGTCCCAAGGACTGACGAGG  2760
Sbjct  2763  ...................................................T........  2822

Query  2761  GCCTCCGTATATGCGGTTCGAACTAAAGTCAATGAGAACCCGCTATATGCACAGACCTCC  2820
Sbjct  2823  .................C........................T.................  2882

Query  2821  GAGCACGTGAACGTGTTGTTAACACGCACAGAAAACAAGCTAGTATGGAAGACCTTGTCA  2880
Sbjct  2883  .................A..........................................  2942
```

```
Query  2881  ACAGATCCCTGGATTAAAACACTGACTAACCCACCTAGAGGGCACTATACCGCCACCATA  2940
Sbjct  2943  ..............C......T....C.................................  3002

Query  2941  GCAGAATGGGAAGCGGAACACCAGGGTATAATGAAGGCCATACAAGGGTATGCACCGCCC  3000
Sbjct  3003  ..............A.............................G..............T  3062

Query  3001  GTGAACACCTTCATGAACAAAGTAAATGTGTGCTGGGCAAAGACACTTACGCCTGTGCTG  3060
Sbjct  3063  ...............................................C.T..........  3122

Query  3061  GAAACTGCGGGTATCTCCCTGTCAGCAGAAGACTGGTCTGAACTGCTGCCCCCGTTTGCC  3120
Sbjct  3123  ..................................................T.........  3182

Query  3121  CAGGACGTGGCGTACTCACCCGAGGTGGCATTAAACATCATATGCACGAAAATGTATGGG  3180
Sbjct  3183  ..............T.............................................  3242

Query  3181  TTTGACTTAGACACTGGTCTTTTTTCCAGGCCATCAGTGCCAATGACATACACCAAAGAC  3240
Sbjct  3243  ..............C..C..............T...........................  3302

Query  3241  CATTGGGATAACAGAGTTGGAGGGAAAATGTATGGATTCAGCCAACAAGCATACGATCAG  3300
Sbjct  3303  .................C..........................................  3362

Query  3301  CTGGCAAGACGACATCCGTACCTTCGAGGTAGAGAGAAATCAGGAATGCAGATCGTAGTC  3360
Sbjct  3363  ..................................................A.........  3422

Query  3361  ACTGAAATGCGTATCCAGCGCCCAAGATCGGATGCCAACATCATCCCGATCAACCGCAGG  3420
Sbjct  3423  .....G......................................................  3482

Query  3421  CTCCCTCACTCACTCGTAGCCACACACGAGTATAGGCGAGCTGCACGGGCCGAGGAATTC  3480
Sbjct  3483  ................................C.................T.........  3542

Query  3481  TTCACCACGACACGAGGGTACACTATGCTGCTGGTCTCTGAGTATAACATGAACTTACCA  3540
Sbjct  3543  ...............................................T........G...  3602

Query  3541  AACAAGAAGATCACCTGGCTGGCTCCGATAGGGACGCAGGGGGCCCATCACACCGCCAAC  3600
Sbjct  3603  ............................................................  3662

Query  3601  CTAAACTTGGGGATACCACCTCTGCTGGGCAGTTTTGATGCGGTGGTTGTGAACATGCCG  3660
Sbjct  3663  T.......A...................................................  3722

Query  3661  ACTCCATTCCGGAACCATCACTACCAGCAATGTGAAGACCACGCGATGAAACTCCAGATG  3720
Sbjct  3723  .....T..T..............T....................................  3782

Query  3721  CTGGCAGGCGACGCACTGAGGCACATTAAACCTGGCGGATCATTGTGGGTCAAGGCATAC  3780
Sbjct  3783  .....C..........................C...........................  3842

Query  3781  GGCTACGCAGACCGGCACAGCGAGCACGTGGTCTTGGCATTGGCTAGAAAGTTTAAAAGC  3840
Sbjct  3843  ............................................................  3902

Query  3841  TTCAGAGTCACACAACCCTCATGCGTGACTTCCAACACCGAGGTGTTTCTCCACTTCTCA  3900
Sbjct  3903  ...........G........G.....A..A..................T...........  3962

Query  3901  ATTTTTGACAATGGCAAACGCGCGATAGCCCTGCATTCAGCTAATAGGAAGGCTAACAGT  3960
Sbjct  3963  ...........C................................................  4022
```

```
Query  3961  ATCTTCCAAAACACCTTCTTACCGGCGGGCAGTGCACCGGCGTACAGAGTCAAACGTGGA  4020
Sbjct  4023  ..T.......................A.................................  4082

Query  4021  GACATTTCGAACGCCCCAGAGGATGCAGTGGTCAATGCAGCAAACCAACAGGGAGTGAAG  4080
Sbjct  4083  .................G..............T...........................  4142

Query  4081  GGTGCTGGAGTTTGCGGTGCAATTTACCGTAAGTGGCCGGACGCTTTCGGTGATGTCGCT  4140
Sbjct  4143  .....A......................................................  4202

Query  4141  ACTCCAACCGGAACAGCAGTTTCGAAATCCGTCCAAGATAAATTGGTGATCCACGCTGTC  4200
Sbjct  4203  .....G........C.............................................  4262

Query  4201  GGCCCGAATTTCTCAAAATGTTCAGAAGAGGAAGGGGACAGAGACCTAGCATCTGCTTAC  4260
Sbjct  4263  ............................................TT.......C......  4322

Query  4261  AGAGCTGCAGCAGAAATAGTGATGGATaaaaaaTTACAACAGTGGCCGTCCCCTTACTC  4320
Sbjct  4323  ..........................C.................T...............  4382

Query  4321  TCCACCGGCATTTATGCCGGAGGAAAAAACAGAGTAGAACAGTCACTCAACCATCTCTTC  4380
Sbjct  4383  ..............C.............................G...............  4442

Query  4381  ACGGCATTCGACAATACTGATGCAGATGTGACCATATATTGCATGGACAAAACATGGGAA  4440
Sbjct  4443  ......................................C.....................  4502

Query  4441  AAGAAGATTAAGGAGGCAATCGATCACCGGACTTCGGTTGAGATGGTGCAGGATGACGTG  4500
Sbjct  4503  ....................T..........................A........T...  4562

Query  4501  CAGTTGGAGGAGGAACTGGTACGAGTACACCCTTTGAGTAGTTTAGCAGGTAGGAAGGGT  4560
Sbjct  4563  .....A................................C..C.....G...........A  4622

Query  4561  TACAGTACGGACAGCGGCCGAGTGTTTTCCTACCTGGAAGGTACCAAATTCCATCAGACT  4620
Sbjct  4623  ..............T.............................................  4682

Query  4621  GCGGTGGACATAGCCGAAATGCAAGTGCTGTGGCCCGCCCTCAAAGAGTCTAATGAGCAA  4680
Sbjct  4683  ......................................T..T..................  4742

Query  4681  ATAGTGGCATACACCTTAGGAGAATCAATGGACCAGATACGTGGCAAGTGCCCGACAGAA  4740
Sbjct  4743  ..........................................A.......T.........  4802

Query  4741  GATACTGACGCCTCCACACCTCCACGGACTGTGCCGTGCCTCTGTCGATACGCCATGACA  4800
Sbjct  4803  .....A.....T................................................  4862

Query  4801  CCAGAGAGAGTGTACCGACTTAAATGCACGAACACTACCCAATTTACGGTTTGCTCATCT  4860
Sbjct  4863  ............................................C.....G.........  4922

Query  4861  TTTGAGTTGCCAAAGTATCACATTCAGGGAGTGCAGAGAGTAAAATGTGAAAGAATCATC  4920
Sbjct  4923  ............................................................  4982

Query  4921  ATCTTAGATCCCACTGTTCCACCAACTTACAAACGGCCATGCATCAGACGGTACCCCTCC  4980
Sbjct  4983  ............................................................  5042

Query  4981  ACAATCTCTTGTAACTCCTCTGAGGACTCCAGGAGCTTGTCTACTTTTTCTGTCAGCTCC  5040
Sbjct  5043  ........C...........C..........................C..C.........  5102
```

```
Query  5041  GACTCCTCGATTGGTTCTCTGCCGGTCGGAGACACGAGACCCATTCCAGCCCCGAGGACC  5100
Sbjct  5103  ............................T..............C.........A......  5162

Query  5101  ATTTTCAGACCCGTCCCTGCCCCGAGAGCACCCGTGCTCAGAACCACACCGCCTCCTAAA  5160
Sbjct  5163  ...........T................................................  5222

Query  5161  CCACCGCGCACATTCACCGTGCGTGCAGAAGTGCACCAAGCACCCCCTACACCTGTACCT  5220
Sbjct  5223  ............................................T...............  5282

Query  5221  CCACCCAGACCGAAGAGGGCTGCAAAGTTGGCTCGTGAGATGCACCCCGGGTTCACCTTC  5280
Sbjct  5283  ............................................................  5342

Query  5281  GGGGACTTCGGAGAGCACGAGGTTGAGGAGCTTACGGCCTCTCCCTTAACCTTCGGAGAT  5340
Sbjct  5343  .......................C.......................G............  5402

Query  5341  TTTGCTGAAGGAGAGATCCAGGGGATGGGAGTGGAGTTTGAATGACTAGGCAGAGCCGGC  5400
Sbjct  5403  ..C..............T..........................................  5462

Query  5401  GGGTACATTTTTTCGTCAGACACGGGTCCAGGCCACCTACAGCAGAGATCCGTTTTACAA  5460
Sbjct  5463  ...........C......................................T...C.....  5522

Query  5461  AATTGCACGGCAGAATGTATCTACGAACCGGCAAAACTAGAAAAAATTCATGCACCAAAG  5520
Sbjct  5523  ...........T................................................  5582

Query  5521  TTGGATAAAACCAAGGAAGATATCTTAAGGAGCAAGTACCAAATGAAACCGTCTGAAGCA  5580
Sbjct  5583  ....................C.......................................  5642

Query  5581  AACAAAAGCAGGTACCAATCTAGAAAAGTAGAAAATATGAAAGCAGAGATCGTAGGTAGA  5640
Sbjct  5643  ..............T...................................T.........  5702

Query  5641  CTCTTGGACGGACTGGGGGAGTATCTGGGCACCGAGCATCCAGTTGAATGCTACCGAATA  5700
Sbjct  5703  .....................................G.T....................  5762

Query  5701  ACGTACCCGGTGCCTATATACTCAACTAGTGACCTCAGAGGTCTGTCTAGTGCCAAAACA  5760
Sbjct  5763  ..........CA..................AT..G...................G.....  5822

Query  5761  GCTGTTAGAGCTTGCAATGCATTTTTGGAAGCTAATTTTCCATCAGTCACTTCATATAAA  5820
Sbjct  5823  ............................................................  5882

Query  5821  ATTACTGATGAATACGACGCATACCTAGATATGGTAGATGGATCAGAGAGCTGTCTGGAC  5880
Sbjct  5883  ..............T.............................................  5942

Query  5881  AGATCCTCCTTTTCGCCGTCTAGATTGCGTAGCTTTCCAAAAACACACTCATACTTGGAC  5940
Sbjct  5943  .................A..C.A......C.......................T......  6002

Query  5941  CCACAGATCAACAGTGCGGTACCGTCACCATTCCAAAACACCTTACAAAATGTATTGGCA  6000
Sbjct  6003  .............................T.....G........................  6062

Query  6001  GCGGCCACCAAAAGAAACTGTAATGTCACACAGATGAGAGAACTACCAACATATGATTCT  6060
Sbjct  6063  ..A..............T...................................C......  6122

Query  6061  GCAGTGCTAAATGTAGAGGCCTTCAGGAAATATGCGTGCAAGCCAGACGTATGGGATGAG  6120
Sbjct  6123  ............................................................  6182
```

```
Query  6121  TACAGGGATAATCCGATTTGCATAACCACCGAAAATGTCACCACTTACGTCGCCAAGTTG  6180
Sbjct  6183  .................C...................................T......  6242

Query  6181  AAAGGACCGAAAGCTGCGGCCTTGTTTGCAAAAACACATAACCTGATACCACTACACCAA  6240
Sbjct  6243  ........................................................T...  6302

Query  6241  GTTCCTATGGACAAATTCACGGTAGATATGAAGAGAGATGTCAAAGTCACGCCCGGAACC  6300
Sbjct  6303  .....C......................................................  6362

Query  6301  AAGCACACCGAAGAGAGACCAAAGGTACAGGTGATTCAAGCGGCAGAGCCACTAGCCACT  6360
Sbjct  6363  ........T...................................................  6422

Query  6361  GCCTACCTCTGCGGAATTCACCGTGAATTGGTGCGCCGTCTCAACAACGCGCTTTTCCCA  6420
Sbjct  6423  ..............T......................................C......  6482

Query  6421  AACATCCACACTTTGTTTGATATGTCCGCAGAGGATTTCGATGCAATCATAGCGGAACAT  6480
Sbjct  6483  ...........C.....C....................T.....................  6542

Query  6481  TTTAAGCACGGTGACCATGTGTTGGAAACGGATATAGCCTCTTTTGACAAAAGTCAAGAT  6540
Sbjct  6543  .................C.......................A..................  6602

Query  6541  GATTCCATGGCACTCACTGCGTTAATGATCCTTGAGGACCTGGGAGTAGACCAAAACCTA  6600
Sbjct  6603  ....................A...........C........................T..  6662

Query  6601  ATGAATTTGATAGAGGCTGCATTCGGGGAAATCGTGAGTACACACTTGCCCACAGGTACT  6660
Sbjct  6663  ..................................................A........C  6722

Query  6661  AGATTCAAATTTGGAGCTATGATGAAGTCTGGAATGTTTTTGACGCTGTTCGTCAATACA  6720
Sbjct  6723  ................................G...........A.....T.........  6782

Query  6721  ATTCTTAATGTGGTTATTGCGTGCCGAGTGTTGGAGGATCAATTGGCGCAGTCGCCGTG-  6779
Sbjct  6783  ..............C...........................C................T  6842

Query  6780  GCCTGCTTTCATAGGAGATGACAACATAATCCATGGTATAATATCAGACAAATTGATGGC  6839
Sbjct  6843  ...-.....T......................................T...........  6901

Query  6840  AGATAGATGTGCCACCTGGATGAACATGGAGGTCAAGATACTGGACTCTATAGTTGGAAT  6899
Sbjct  6902  G................................T..........................  6961

Query  6900  ACGGCCACCTTACTTCTGTGGAGGATTTATTGTATGTGACGATGTAACAGGTACAGCCTG  6959
Sbjct  6962  .........A..T..............C.....G........C..............T..  7021

Query  6960  CCGCGTCGCAGACCCACTGAAGAGATTGTTCAAGCTAGGTAAGCCATTGCCACTTGACGA  7019
Sbjct  7022  ............T............C..................................  7081

Query  7020  TGGCCAAGATGAAGACAGAAGACGTGCATTACATGATGAAGTGAAAACCTGGTCGCGCGT  7079
Sbjct  7082  ............................................................  7141

Query  7080  AGGGCTGCGACACAGAGTGTGTGAAGCCATCGAAGACCGTTATGCCGTCCACTCATCAGA  7139
Sbjct  7142  ...................................................T........  7201

Query  7140  ACTAGTTTTATTGGCACTGACTACTCTGTCTAAGAACTTGAAGTCCTTCAGAAACATAAG  7199
Sbjct  7202  .......C....................................................  7261
```

```
Query  7200  AGGGAAACCAATACATCTCTACGGTGGTCCTAAATAG  7236
Sbjct  7262  .....................................  7298
```
