# Supplementary figures and images for: Exploring Barmah Forest virus pathogenesis: molecular tools to investigate non-structural protein 3 nuclear localization and viral genomic determinants of replication
Source: mBio. 2024 Jul 2;15(8):e00993-24. doi: 10.1128/mbio.00993-24 (PMC11323547; doi:10.1128/mbio.00993-24)

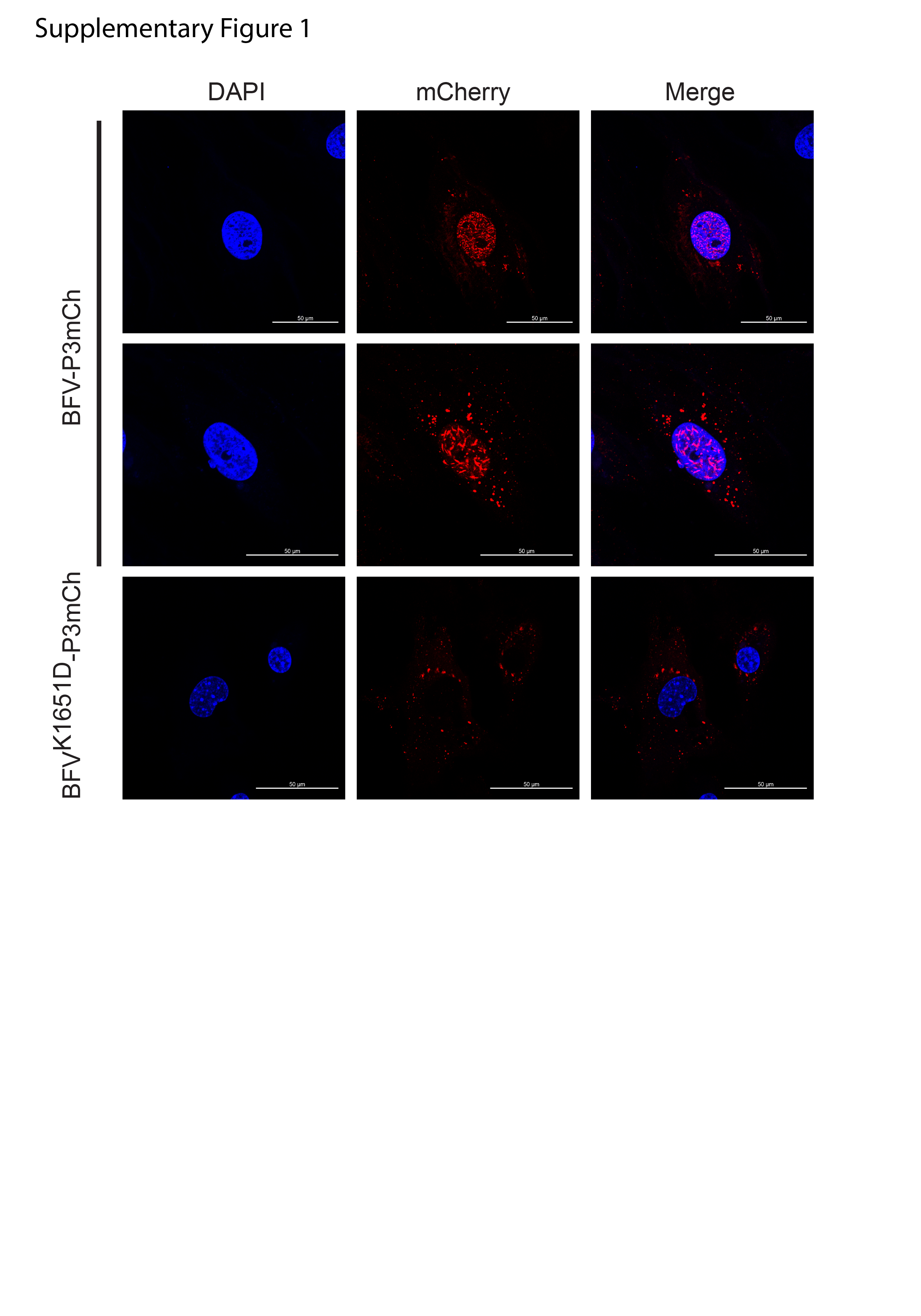

Supplement: Figure S1 — MEF cells infected with BFV-P3mCh or BFVK1651D-P3mCh. [file mbio.00993-24-s0003.tiff]

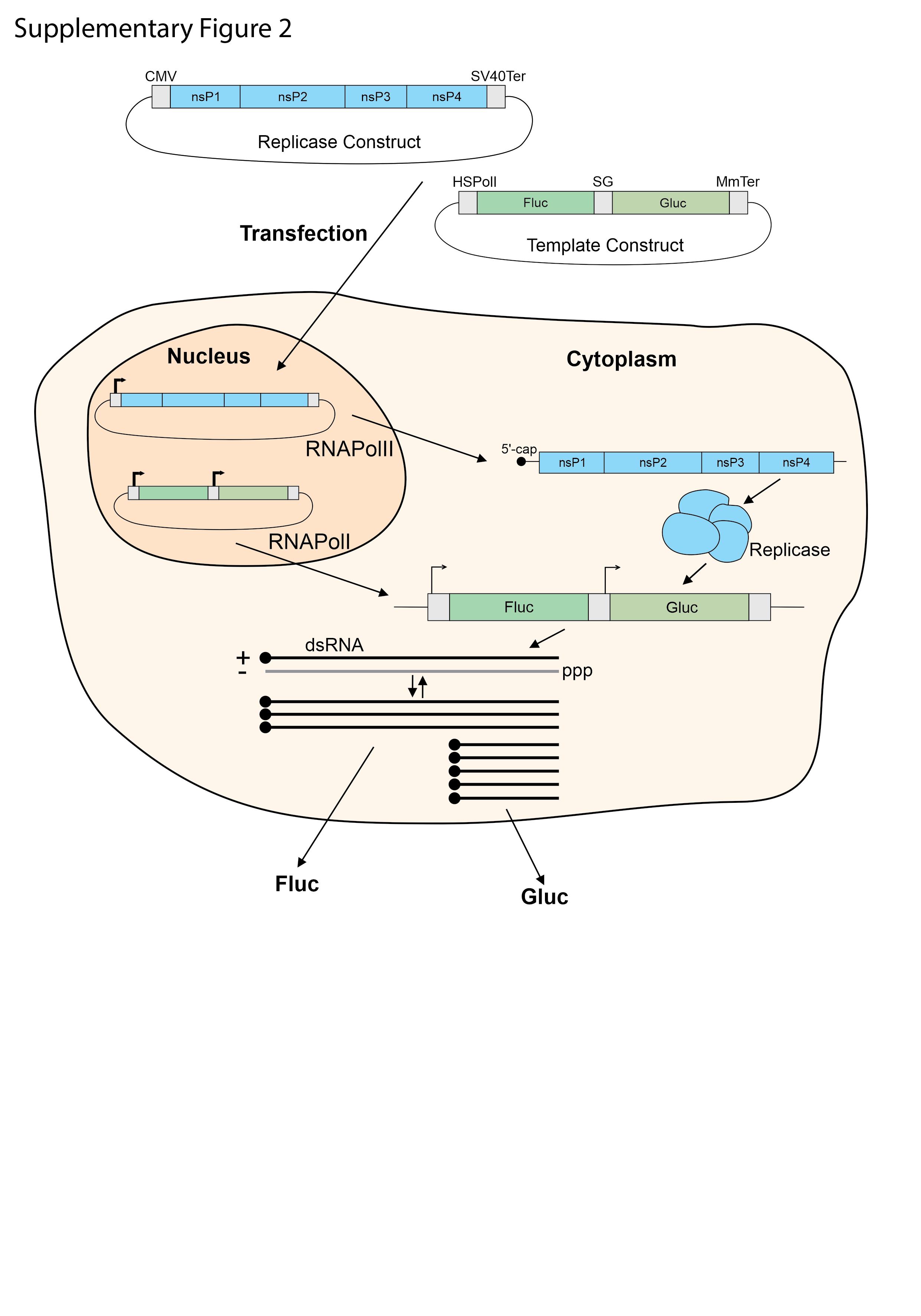

Supplement: Figure S2 — Components and principle of BFV trans-replicase system. [file mbio.00993-24-s0004.tiff]
