# Supplementary material for: Exploring Barmah Forest virus pathogenesis: molecular tools to investigate non-structural protein 3 nuclear localization and viral genomic determinants of replication
Source: mBio. 2024 Jul 2;15(8):e00993-24. doi: 10.1128/mbio.00993-24 (PMC11323547; doi:10.1128/mbio.00993-24)
Supplement: Supplemental legends — Legends for supplemental movies, figures, and data. [file mbio.00993-24-s0005.docx]

**Supplementary Figure 1.** MEF cells were infected with BFV-P3mCh or BFVK1651D-P3mCh at an MOI of 1.0. The cells were fixed at 24 h post-infection. Nuclei were counterstained with DAPI. Cells were analysed for mCherry fluorescence using a Zeiss LSM710 confocal microscope. Scale bar = 50 μm.

**Supplementary Figure 2. Components and principle of BFV trans-replicase system**

**Supplementary movie 1. BFV nsP3-mCherry localization during BFV-P3mCh infection.**
Vero-E6 cells were infected with BFV-P3mCh at an MOI of 1.0 and subsequently analyzed for mCherry fluorescence with the EVOS^TM^ Imaging System. Images were taken every 30 min and shown here from the timepoints of clear fluorescence signal appearance at 5.5 h up to 24.5 h.

**Supplementary movie 2. BFV nsP3-mCherry localization during BFV^K1651D^-P3mCh infection.**
Vero-E6 cells were infected with BFV^K1651D^-P3mCh at an MOI of 1.0 and subsequently analyzed for mCherry fluorescence with the EVOS^TM^ Imaging System. Images were taken every 30 min and shown here from the timepoints of clear fluorescence signal appearance at 5.5 h up to 24.5 h.

**Supplementary data 1. NGS sequencing data for BFV2193-FI.**

Vero cells were infected with BFV2193-FI at an MOI of 1.0. The culture medium was collected at 24 h p.i., and RNA extracted with TRIzol® reagent. The purified RNA was sent for NGS analysis. Obtained datasets were analyzed by de novo assembly using the Trinity Transcriptome Assembly. The 5’- and 3‘-UTR of BFV2193-FI were amplified with the SMARTer® RACE 5'/3' Kit. The obtained fragments were cloned and analyzed using Sanger sequencing. The obtained complete sequence of BFV2193-FI is shown.

**Supplementary data 2. BFV non-structural polyprotein alignment for mutation position comparison.**

Genome sequences of 38 BFV isolates were obtained from GenBank database. Sequence alignment over the non-structural protein region was performed using Standard Nucleotide BLAST.
